# Supplementary material for: Transcriptome-Wide Identification of Coding and Noncoding RNA-Binding Proteins Defines the Comprehensive RNA Interactome of Leishmania mexicana
Source: Microbiol Spectr. 2022 Feb 9;10(1):e02422-21. doi: 10.1128/spectrum.02422-21 (PMC8826732; doi:10.1128/spectrum.02422-21)
Supplement: SUPPLEMENTAL FILE 1 — Supplemental material. Download SPECTRUM02422-21_Supp_1_seq3.pdf, PDF file, 2.1 MB [file spectrum02422-21_supp_1_seq3.pdf]

Supplemental Material

Transcriptome-wide identification of coding and noncoding RNA-binding proteins defines the comprehensive RNA interactome of *Leishmania mexicana*

Karunakaran Kalesh,[a*] Wenbin Wei,[b] Brian S. Mantilla,[b] Theodoros I. Roumeliotis,[c] Jyoti Choudhary,[c] Paul W. Denny[b]

[a]Department of Chemistry, Durham University, Durham, United Kingdom

[b]Department of Biosciences, Durham University, Durham, United Kingdom

[c]Functional Proteomics Group, The Institute of Cancer Research, London, United Kingdom

*Address correspondence to Karunakaran Kalesh, kalesh.karunakaran@durham.ac.uk

FIG S1

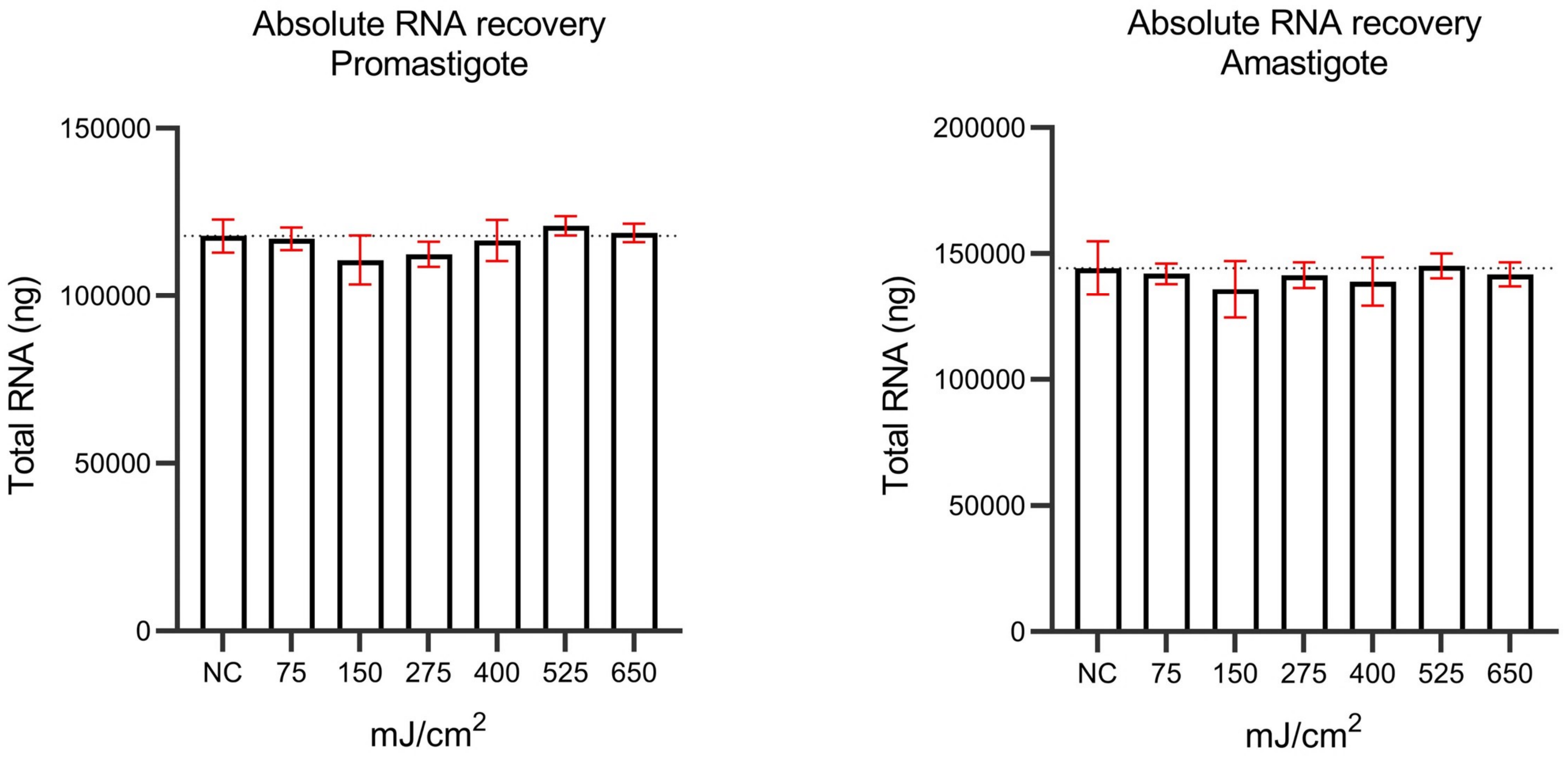
Absolute RNA recovery
Promastigote
150000
100000
50000
0
Total RNA (ng)
NC
75
150
275
400
525
650
mJ/cm2
Absolute RNA recovery
Amastigote
200000
150000
100000
50000
0
Total RNA (ng)
NC
75
150
275
400
525
650
mJ/cm2

FIG S2

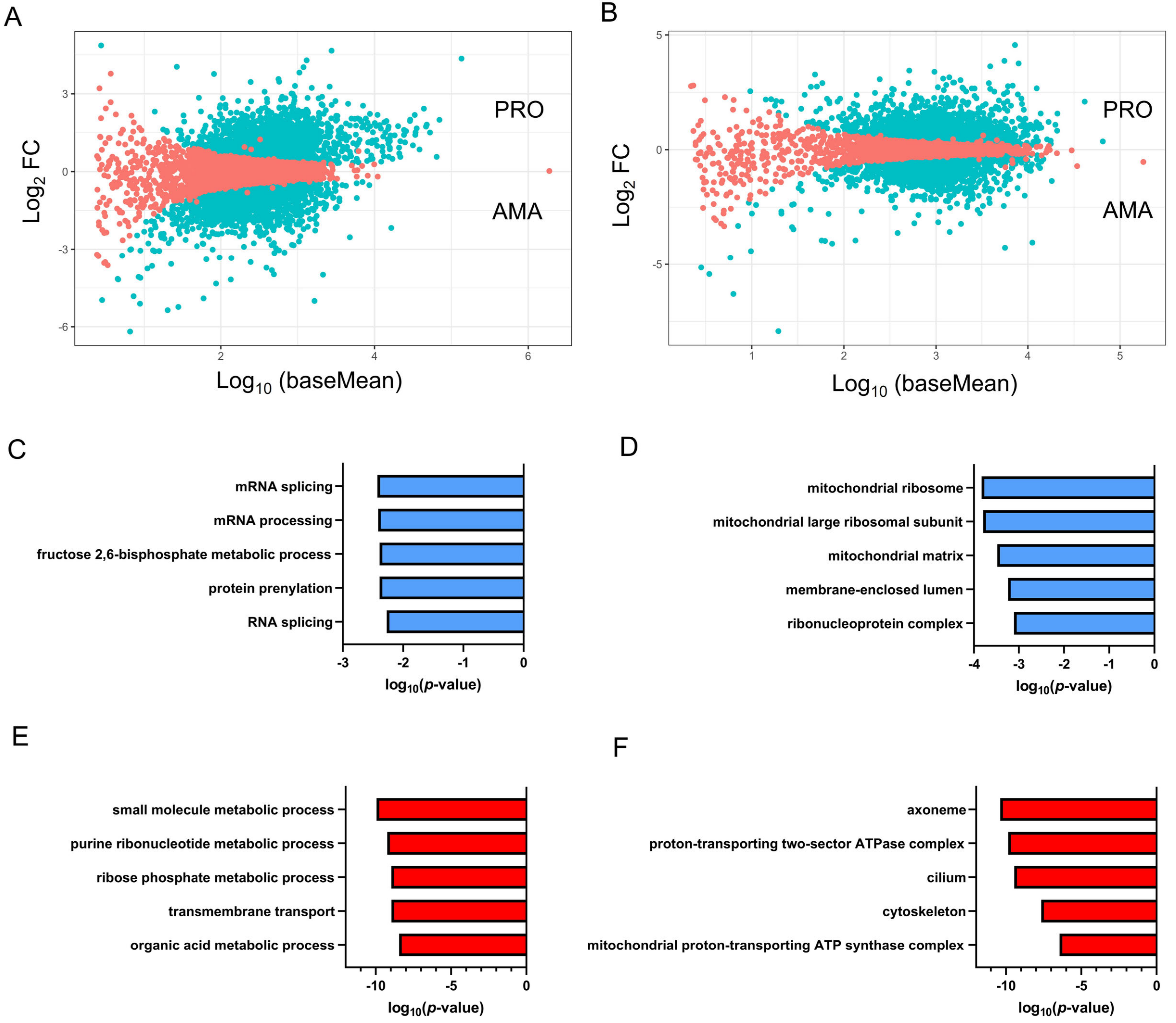

A
PRO
AMA
Log2 FC
Log10 (baseMean)
B
PRO
AMA
Log2 FC
Log10 (baseMean)
C
mRNA splicing
mRNA processing
fructose 2,6-bisphosphate metabolic process
protein prenylation
RNA splicing
log10(p-value)
D
mitochondrial ribosome
mitochondrial large ribosomal subunit
mitochondrial matrix
membrane-enclosed lumen
ribonucleoprotein complex
log10(p-value)
E
small molecule metabolic process
purine ribonucleotide metabolic process
ribose phosphate metabolic process
transmembrane transport
organic acid metabolic process
log10(p-value)
F
axoneme
proton-transporting two-sector ATPase complex
cilium
cytoskeleton
mitochondrial proton-transporting ATP synthase complex
log10(p-value)

FIG S3

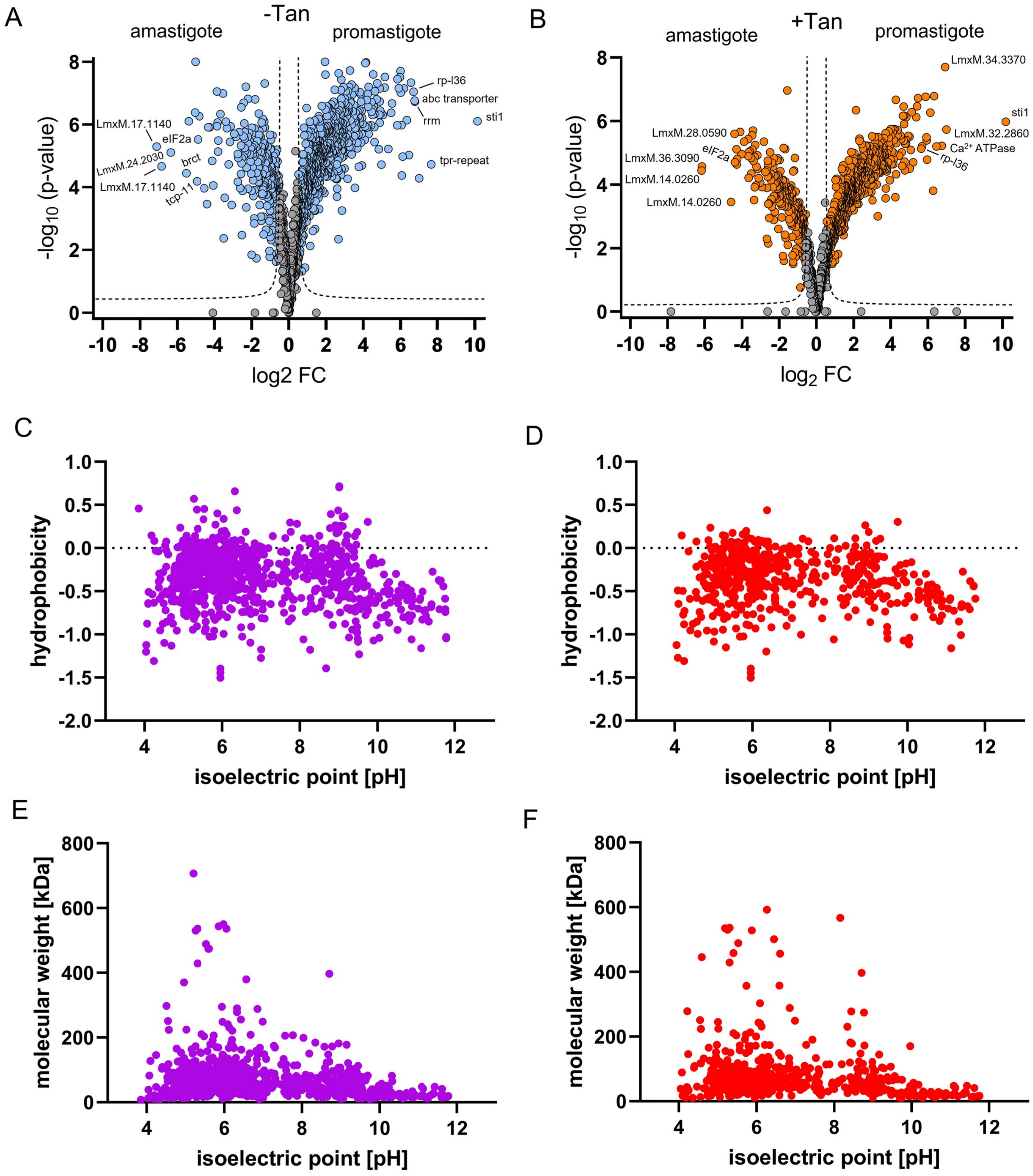
A
-Tan
amastigote
promastigote
LmxM.17.1140
eIF2a
LmxM.24.2030
brct
LmxM.17.1140
tcp-11
rp-l36
abc transporter
rrm
sti1
tpr-repeat
-log10 (p-value)
log2 FC
B
+Tan
amastigote
promastigote
LmxM.34.3370
sti1
LmxM.32.2860
Ca2+ ATPase
rp-l36
LmxM.28.0590
eIF2a
LmxM.36.3090
LmxM.14.0260
LmxM.14.0260
-log10 (p-value)
log2 FC
C
D
hydrophobicity
isoelectric point [pH]
E
F
molecular weight [kDa]
isoelectric point [pH]

SUPPLEMENTAL FIGURE LEGEND

FIG. S1 Absolute quantification of the total recovered RNA (PBR + free RNA) in *L. mexicana* promastigotes and axenic amastigotes. Data shown as mean +/- SD of three independent experiments.

FIG. S2 Differential gene expression analysis of RNA-seq data sets in *L. mexicana*. Scatter plots of mean of normalised read counts in log-scale (log_{10} baseMean) versus log2 fold change (log_2 FC) for promastigote (PRO) versus axenic amastigote (AMA) samples of NC (A) and CL (B) conditions with statistically significant hits ($P_{adj}<0.05$) shown in blue. BP and CC GO-Term enrichment analyses of the transcripts with significant ($P_{adj}<0.05$) differential expression between the two life cycle stages in the CL samples of axenic amastigotes (C and D) and promastigotes (E and F) respectively.

FIG. S3 Tanespimycin affects the RBPs of *L. mexicana*. Volcano plots showing differential enrichment of RBPs in the promastigote and axenic amastigote life cycle stages of *L. mexicana* with vehicle (DMSO) treatment (A) and with tanespimycin (1 µM for 16 h) treatment (B). A modified *t*-test with permutation based FDR statistics (250 permutations, FDR = 0.05) was applied to compare the +Tan and -Tan groups. (C) and (D), Scatter plots comparing isoelectric points and hydrophobicity of proteins in promastigotes and amastigotes respectively. (E) and (F) Scatter plots comparing isoelectric points and molecular weight of proteins in promastigotes and amastigotes respectively.

Table Legends

TABLE S1 Total RBPs identified with two or more unique peptides in *L. mexicana* promastigotes. The fold change (FC) of CL samples versus NC samples in log2 scale and statistical significance of the reporter intensity quantifications across three independent replicates of CL versus NC samples as negative log of *t*-test P-values are also presented.

TABLE S2. Total RBPs identified with two or more unique peptides in *L. mexicana* axenic amastigotes. The fold change (FC) of CL samples versus NC samples in log2 scale and statistical significance of the reporter intensity quantifications across three independent replicates of CL versus NC samples as negative log of *t*-test P-values are also presented.

TABLE S3 *L. mexicana* poly(A) interactomes.

TABLE S4 *L. mexicana* non-poly(A) interactomes.

TABLE S5 Protein kinase genes identified in the CL transcripts of *L. mexicana* axenic amastigotes.

TABLE S6 Protein kinase genes identified in the CL transcripts of *L. mexicana* promastigotes.

TABLE S7 High-confidence tanespimycin-affected RBPs of *L. mexicana* promastigotes. Ribosomal proteins, proteasomal proteins and translation factors are highlighted in red, blue, and green respectively.

TABLE S8 High-confidence tanespimycin-affected RBPs of *L. mexicana* axenic amastigotes. Ribosomal proteins, proteasomal proteins and translation factors are highlighted in red, blue, and green respectively.

TABLE S9 Top-10 MF, CC and BP GO-Terms enriched in the tanespimycin-affected RBPs of *L. mexicana*.

TABLE S10 Effect of tanespimycin on the RBPs of *L. mexicana* promastigotes compared with the effect on the synthesis of the nascent proteins revealed by BONCAT-iTRAQ quantitative proteomic MS (ref. 32). An increase (positive value of log2 FC) or decrease (negative value of log2 FC) of RNA-binding upon tanespimycin-treatment quantified by the MS3 dataset of the OOPS-TMTsixplex-LC/MS/MS is compared with the change in abundance of the nascent proteins revealed by the BONCAT-iTRAQ quantitative proteomic MS data set. Positively and negatively correlated RBPs are highlighted in blue and orange respectively.

Table S1. RBPs in *L. mexicana* promastigotes

[-Log(P-value)]	log2 FC	Protein IDs	Fasta headers	Unique peptides
1.961848106	7.299253	E9AZ49	tr|E9AZ49|E9AZ49_LEIMU Uncharacterized protein OS=Leishm	2
4.878329476	7.008324	E9AST6	tr|E9AST6|E9AST6_LEIMU Putative universal minicircle sequen	7
2.301493551	6.887898	E9AUE0	tr|E9AUE0|E9AUE0_LEIMU DNA-directed RNA polymerase sub	2
3.656637374	6.581095	E9ATA6	tr|E9ATA6|E9ATA6_LEIMU Putative 60S ribosomal protein L22	6
2.571398557	6.517256	E9ASI0;E9AI	tr|E9ASI0|E9ASI0_LEIMU Putative ubiquitin/ribosomal protein	2
2.832939773	6.438545	E9AXF0	sp|E9AXF0|IF5A_LEIMU Eukaryotic translation initiation factor	4
2.142478368	6.391259	E9AKC3	tr|E9AKC3|E9AKC3_LEIMU Uncharacterized protein OS=Leishm	2
3.368120282	6.368167	E9AXI7	tr|E9AXI7|E9AXI7_LEIMU Uncharacterized protein OS=Leishma	3
3.998190677	6.309788	E9AZ22;E9A	tr|E9AZ22|E9AZ22_LEIMU Putative histone H1 OS=Leishmania	3
3.163120188	6.146287	E9B259	tr|E9B259|E9B259_LEIMU Putative calreticulin OS=Leishmania	4
3.026044848	6.09926	E9AQA3	tr|E9AQA3|E9AQA3_LEIMU Tryparedoxin peroxidase OS=Leish	2
3.268432814	6.057908	E9ARS6	tr|E9ARS6|E9ARS6_LEIMU Putative pumilio protein OS=Leishm	3
3.501259919	5.960386	E9ARF9	tr|E9ARF9|E9ARF9_LEIMU Putative RNA-binding protein OS=Le	7
3.183339046	5.886726	E9AMC0	tr|E9AMC0|E9AMC0_LEIMU Uncharacterized protein OS=Leish	3
3.822504621	5.851223	E9B665	tr|E9B665|E9B665_LEIMU Putative 60S ribosomal protein L5 O	13
3.467393289	5.788977	E9B721	tr|E9B721|E9B721_LEIMU Uncharacterized protein OS=Leishm	4
3.104835434	5.723198	E9B1A6	tr|E9B1A6|E9B1A6_LEIMU Uncharacterized protein OS=Leishm	5
2.746713821	5.721594	E9AQ02	tr|E9AQ02|E9AQ02_LEIMU NADH-cytochrome b5 reductase OS	2
3.80266801	5.675918	E9B4X4	tr|E9B4X4|E9B4X4_LEIMU Uncharacterized protein OS=Leishm	8
3.130564033	5.667205	E9AQE5;B8I	tr|E9AQE5|E9AQE5_LEIMU Phosphodiesterase OS=Leishmania	2
2.924017189	5.655875	E9B6F3	tr|E9B6F3|E9B6F3_LEIMU Galactokinase-like protein OS=Leishi	5
4.007541933	5.621325	E9B3G8	tr|E9B3G8|E9B3G8_LEIMU Putative 60S ribosomal protein L2 C	12
3.128585319	5.616306	E9AN31	tr|E9AN31|E9AN31_LEIMU Uncharacterized protein OS=Leishn	2
3.461752469	5.605793	E9AK09	tr|E9AK09|E9AK09_LEIMU Uncharacterized protein OS=Leishm	2
3.978033564	5.60487	E9AU61	tr|E9AU61|E9AU61_LEIMU Putative chaperone protein DNAj C	2
3.527433485	5.587635	E9B303	tr|E9B303|E9B303_LEIMU Uncharacterized protein OS=Leishm	3
4.097576667	5.569937	E9AW05	tr|E9AW05|E9AW05_LEIMU Peptidyl-prolyl cis-trans isomerase	3
3.607217518	5.553515	E9AL77	tr|E9AL77|E9AL77_LEIMU Uncharacterized protein OS=Leishm	6
3.676200758	5.529841	E9B612	tr|E9B612|E9B612_LEIMU Uncharacterized protein OS=Leishm	2
3.726326694	5.525297	E9AVM6	tr|E9AVM6|E9AVM6_LEIMU Putative 40S ribosomal protein S1	3
3.352228018	5.520797	E9B3B7	tr|E9B3B7|E9B3B7_LEIMU Uncharacterized protein OS=Leishm	2
3.845791121	5.50551	E9APW9	tr|E9APW9|E9APW9_LEIMU Uncharacterized protein OS=Leish	2
3.887356828	5.48731	E9B469	tr|E9B469|E9B469_LEIMU Putative udp-glc 4-epimerase OS=Le	4
4.097149434	5.485994	E9B6W8	tr|E9B6W8|E9B6W8_LEIMU Uncharacterized protein OS=Leish	3
3.64863245	5.464754	E9AY76	tr|E9AY76|E9AY76_LEIMU Uncharacterized protein OS=Leishm	2
3.868565312	5.411219	E9ARW1	tr|E9ARW1|E9ARW1_LEIMU Putative fibrillarin OS=Leishmania	6
3.824897764	5.409145	E9AK40;A0A	tr|E9AK40|E9AK40_LEIMU Putative spermidine synthase OS=Le	2
3.514571721	5.409109	E9AS19	tr|E9AS19|E9AS19_LEIMU Putative U3 snoRNA-associated prot	2
4.153912396	5.392574	E9AVY4	tr|E9AVY4|E9AVY4_LEIMU Putative 40S ribosomal protein L14	6
4.071001088	5.388751	E9AKE4	tr|E9AKE4|E9AKE4_LEIMU Putative microtubule-associated pro	8
4.041881701	5.377602	E9AYI0	tr|E9AYI0|E9AYI0_LEIMU Uncharacterized protein OS=Leishma	2
3.330216081	5.372909	E9AXW8	tr|E9AXW8|E9AXW8_LEIMU Putative serine/threonine-protein	4
3.646984762	5.369988	E9B6T1	tr|E9B6T1|E9B6T1_LEIMU RNA helicase OS=Leishmania mexica	2
4.044189534	5.365204	E9ARG7	tr|E9ARG7|E9ARG7_LEIMU Uncharacterized protein OS=Leishn	2
3.758191067	5.35443	E9APY5	tr|E9APY5|E9APY5_LEIMU Putative tyrosyl-tRNA synthetase OS	3
3.576533476	5.339412	E9B5Y1;E9B	tr|E9B5Y1|E9B5Y1_LEIMU Putative casein kinase OS=Leishman	6
3.187094803	5.30895	E9B600	tr|E9B600|E9B600_LEIMU Uncharacterized protein OS=Leishm	4
3.890759562	5.306946	E9B4K7	tr|E9B4K7|E9B4K7_LEIMU Uncharacterized protein (Fragment)	3

4.750000244	5.291805	E9B2L1	tr|E9B2L1|E9B2L1_LEIMU Phenylalanyl-tRNA synthetase alpha	4
3.666692982	5.291691	E9ANS8	tr|E9ANS8|E9ANS8_LEIMU Proteasome subunit beta OS=Leishı	2
3.533555353	5.291658	E9AUR8	tr|E9AUR8|E9AUR8_LEIMU Putative calpain-like cysteine pepti	3
3.978648038	5.286392	E9ATX7	tr|E9ATX7|E9ATX7_LEIMU Putative branch point binding prote	3
4.488930376	5.280318	E9ALD1	tr|E9ALD1|E9ALD1_LEIMU Proteasome regulatory non-ATP-ase	3
4.247058987	5.279893	E9AMI1	tr|E9AMI1|E9AMI1_LEIMU Stress-induced protein sti1 OS=Leis	11
3.843594817	5.275613	E9AWI0	tr|E9AWI0|E9AWI0_LEIMU Pyridoxal phosphate homeostasis p	3
3.617907431	5.275589	E9AZ74	tr|E9AZ74|E9AZ74_LEIMU Uncharacterized protein OS=Leishm	3
4.082518479	5.272299	E9AV53	tr|E9AV53|E9AV53_LEIMU Uncharacterized protein OS=Leishm	2
3.920527618	5.269508	E8NHK6	tr|E8NHK6|E8NHK6_LEIMU WGS CADB00000000 data, contig 5	4
4.1299361	5.266336	E9AMY4	tr|E9AMY4|E9AMY4_LEIMU Putative paraflagellar rod compon	3
4.605822151	5.25968	E9B0I7	tr|E9B0I7|E9B0I7_LEIMU Ribosome biogenesis regulatory prot	4
4.083532584	5.256313	E8NHE3	tr|E8NHE3|E8NHE3_LEIMU Putative 60S ribosomal protein L28	6
4.518419851	5.249299	E9ARF8	tr|E9ARF8|E9ARF8_LEIMU Uncharacterized protein OS=Leishm	5
4.317122354	5.246743	E9ASM7	tr|E9ASM7|E9ASM7_LEIMU Putative ribosomal protein L24 OS	4
2.928252176	5.237494	E9B207	tr|E9B207|E9B207_LEIMU Uncharacterized protein OS=Leishm	4
3.724542359	5.228003	E9ASR3	tr|E9ASR3|E9ASR3_LEIMU Putative translation elongation fact	7
3.936369798	5.222464	E9ALB6	tr|E9ALB6|E9ALB6_LEIMU Putative nucleolar RNA-binding prot	7
4.560724006	5.22188	E9B0V3	tr|E9B0V3|E9B0V3_LEIMU Uncharacterized protein OS=Leishm	4
4.198893413	5.206586	E9AV21	tr|E9AV21|E9AV21_LEIMU Putative la RNA binding protein OS=	8
4.427065644	5.201057	E9ASC2	tr|E9ASC2|E9ASC2_LEIMU Putative PUF1 OS=Leishmania mexic	5
3.50851126	5.196866	E9AKJ8	tr|E9AKJ8|E9AKJ8_LEIMU Putative paraflagellar rod protein OS	2
3.756026663	5.194477	E9AQL0	tr|E9AQL0|E9AQL0_LEIMU Uncharacterized protein OS=Leishm	4
4.158798399	5.194172	E9AJK8	tr|E9AJK8|E9AJK8_LEIMU Uncharacterized protein OS=Leishma	2
3.738681102	5.191606	E9B0C5	tr|E9B0C5|E9B0C5_LEIMU Uncharacterized protein LMXM_28_	10
4.260478406	5.189936	E9ALY9	tr|E9ALY9|E9ALY9_LEIMU Guanine deaminase OS=Leishmania	3
3.583958698	5.189722	E9AQ39	tr|E9AQ39|E9AQ39_LEIMU Putative 60S acidic ribosomal prote	2
4.592335279	5.186861	E9AV37	tr|E9AV37|E9AV37_LEIMU Putative ribonuclease L inhibitor OS	3
3.895340556	5.176087	E9AWM7	tr|E9AWM7|E9AWM7_LEIMU Putative aspartate aminotransfe	3
4.000965525	5.173402	E9AT36	tr|E9AT36|E9AT36_LEIMU Putative membrane-bound acid pho	9
3.887678101	5.168729	E9AR18	tr|E9AR18|E9AR18_LEIMU Uncharacterized protein OS=Leishm	2
4.155726504	5.166524	E9ARZ6	tr|E9ARZ6|E9ARZ6_LEIMU Putative nucleosome assembly prot	9
4.586434751	5.158922	E9AZL5	tr|E9AZL5|E9AZL5_LEIMU 40S ribosomal protein S26 OS=Leishı	8
4.246735094	5.157225	E9AQS3	tr|E9AQS3|E9AQS3_LEIMU Tyrosyl or methionyl-tRNA syntheta	4
4.115383177	5.151414	E9AMM0	tr|E9AMM0|E9AMM0_LEIMU Uncharacterized protein OS=Leis	2
3.783832104	5.147222	E9B602	tr|E9B602|E9B602_LEIMU Putative short chain dehydrogenase	4
3.918387097	5.140332	E9AZP7	tr|E9AZP7|E9AZP7_LEIMU Putative RNA binding protein rbp16	3
4.460590846	5.121853	E9ANS3	tr|E9ANS3|E9ANS3_LEIMU Uncharacterized protein OS=Leishm	3
4.287780618	5.12184	E9B6J0	tr|E9B6J0|E9B6J0_LEIMU Uncharacterized protein OS=Leishma	2
4.199276917	5.120982	E9B5P1;Q27	tr|E9B5P1|E9B5P1_LEIMU Pyruvate kinase OS=Leishmania mex	8
3.848392249	5.115545	E9APM8	tr|E9APM8|E9APM8_LEIMU Putative threonine synthase OS=L	4
4.323606951	5.114866	E9AM64	tr|E9AM64|E9AM64_LEIMU Uncharacterized protein OS=Leish	2
4.249655984	5.110454	E9AKP0	tr|E9AKP0|E9AKP0_LEIMU Peptidyl-prolyl cis-trans isomerase (	5
3.770498192	5.105175	E9AVA8	tr|E9AVA8|E9AVA8_LEIMU Uncharacterized protein OS=Leishn	3
4.104110563	5.101669	E9B2G1	tr|E9B2G1|E9B2G1_LEIMU Putative ATP-dependent RNA helica	25
3.752098844	5.08529	E9B3U1;E9A	tr|E9B3U1|E9B3U1_LEIMU Uncharacterized protein OS=Leishm	2
3.953527303	5.081966	E9B5Y8	tr|E9B5Y8|E9B5Y8_LEIMU Uncharacterized protein OS=Leishm	4
4.073967018	5.081666	E9AUU6	tr|E9AUU6|E9AUU6_LEIMU Putative 40S ribosomal protein S1	12
3.376333407	5.073589	E9AYS1	tr|E9AYS1|E9AYS1_LEIMU Proteasome endopeptidase complex	4
4.065646552	5.066343	E9ARX5	tr|E9ARX5|E9ARX5_LEIMU Uncharacterized protein OS=Leishm	6
4.162697104	5.061596	E9B687	tr|E9B687|E9B687_LEIMU Putative kinesin OS=Leishmania mex	3
3.405917997	5.054957	E9AKN1;Q6	tr|E9AKN1|E9AKN1_LEIMU Uncharacterized protein OS=Leishn	4

4.307172169	5.048908	E9AQ06	tr|E9AQ06|E9AQ06_LEIMU Heat shock protein HslVU, ATPase s	3
3.824538645	5.046888	E9ALI7	tr|E9ALI7|E9ALI7_LEIMU Putative 60S ribosomal protein L39 O	2
4.15596028	5.046	E9AN29	tr|E9AN29|E9AN29_LEIMU Putative nucleolar protein OS=Leish	20
4.523905366	5.043848	E9B479	tr|E9B479|E9B479_LEIMU Putative GTPase protein OS=Leishm	2
4.915040033	5.040745	E9AZ87	tr|E9AZ87|E9AZ87_LEIMU Paraflagellar rod protein-like protein	2
4.34582652	5.038066	E9AZZ3	tr|E9AZZ3|E9AZZ3_LEIMU Uncharacterized protein OS=Leishm	3
4.285283362	5.027549	E9B379	tr|E9B379|E9B379_LEIMU ATP binding protein-like protein OS=	2
3.662978239	5.022456	E9ALX2	tr|E9ALX2|E9ALX2_LEIMU Uncharacterized protein OS=Leishm	4
3.687566601	5.020907	E9B701	tr|E9B701|E9B701_LEIMU Glycine cleavage system H protein C	2
3.977236312	5.01287	Q95NJ3;E8N	tr|Q95NJ3|Q95NJ3_LEIAM Activated C kinase protein OS=Leish	9
4.021747268	5.011844	E9B163	tr|E9B163|E9B163_LEIMU Putative aldehyde dehydrogenase C	9
4.304645309	5.009508	E9AZ16	tr|E9AZ16|E9AZ16_LEIMU Putative intraflagellar transport prot	2
4.190669139	5.007537	E9APS5	tr|E9APS5|E9APS5_LEIMU Uncharacterized protein OS=Leishm	4
4.238616268	5.004729	E9AP12	tr|E9AP12|E9AP12_LEIMU Uncharacterized protein OS=Leishm	9
4.501567933	5.004177	E9AQ75	tr|E9AQ75|E9AQ75_LEIMU Uncharacterized protein OS=Leishn	4
3.86007851	5.003963	E9AUD1;A7	tr|E9AUD1|E9AUD1_LEIMU Protein disulfide-isomerase OS=Lei	18
3.716055788	5.002199	E9AL45	tr|E9AL45|E9AL45_LEIMU Putative ATP-dependent DEAD/H RN	11
4.222572164	5.001174	E9B201;E9B	tr|E9B201|E9B201_LEIMU Succinyl-diaminopimelate desucciny	4
3.811061228	4.999202	E9AVX7	tr|E9AVX7|E9AVX7_LEIMU Peptidyl-prolyl cis-trans isomerase (	2
3.689804475	4.995602	E9B5M4	tr|E9B5M4|E9B5M4_LEIMU H/ACA ribonucleoprotein complex	3
4.349384635	4.993931	E9AWJ4	tr|E9AWJ4|E9AWJ4_LEIMU Putative 60S ribosomal protein L17	10
4.251463452	4.991558	E9AYA3;Q2!	tr|E9AYA3|E9AYA3_LEIMU Heat shock protein 70-related prote	20
4.223360727	4.988526	E9B2C2	tr|E9B2C2|E9B2C2_LEIMU Uncharacterized protein OS=Leishm	14
3.963742903	4.988015	E9B5R8	tr|E9B5R8|E9B5R8_LEIMU Uncharacterized protein OS=Leishm	2
4.260972879	4.986053	E9AS20;E9A	tr|E9AS20|E9AS20_LEIMU Putative kinesin OS=Leishmania mex	3
4.117147702	4.982308	E9AVQ7	tr|E9AVQ7|E9AVQ7_LEIMU Uncharacterized protein OS=Leishn	15
3.320567701	4.977921	E9B4L7	tr|E9B4L7|E9B4L7_LEIMU Putative importin beta-1 subunit OS	3
4.415838227	4.976337	E9AN72	tr|E9AN72|E9AN72_LEIMU Uncharacterized protein OS=Leishn	5
4.386471838	4.974044	E9AYP5	tr|E9AYP5|E9AYP5_LEIMU Uncharacterized protein OS=Leishm	4
4.148556769	4.971588	E9ALH7	tr|E9ALH7|E9ALH7_LEIMU Putative 60S ribosomal protein L13	16
4.973464879	4.971583	E9B5C7	tr|E9B5C7|E9B5C7_LEIMU Glucose-6-phosphate 1-epimerase (	4
4.44676729	4.969713	E9APK7;A0/	tr|E9APK7|E9APK7_LEIMU Nucleoside hydrolase-like protein O	5
3.854008556	4.966382	E9B5R2	tr|E9B5R2|E9B5R2_LEIMU 60S ribosomal protein L30 OS=Leish	8
3.879850052	4.964643	E9B1X5	tr|E9B1X5|E9B1X5_LEIMU Nucleosome assembly protein-like p	7
4.140163336	4.962327	E9B533;E9A	tr|E9B533|E9B533_LEIMU Putative ribosomal protein l35a OS=	7
4.00172119	4.962106	E9B072	tr|E9B072|E9B072_LEIMU Eukaryotic translation initiation fact	3
4.048886136	4.9513	E9ASX8	tr|E9ASX8|E9ASX8_LEIMU Chaperonin HSP60, mitochondrial O	10
4.682723219	4.947998	E9B2G7	tr|E9B2G7|E9B2G7_LEIMU Putative prostaglandin f synthase O	2
3.870579971	4.946743	E9B6H7	tr|E9B6H7|E9B6H7_LEIMU Putative chaperone protein DNAj O	6
3.766786548	4.945303	E9AL62;E9A	tr|E9AL62|E9AL62_LEIMU Putative 60S ribosomal protein L7a (	22
4.533531932	4.945115	E9B0K5	tr|E9B0K5|E9B0K5_LEIMU Mitochondrial oligo_U binding prote	3
4.670639953	4.944896	E9AZN5	tr|E9AZN5|E9AZN5_LEIMU Uncharacterized protein OS=Leishn	3
4.552747276	4.943446	E9ATF7	tr|E9ATF7|E9ATF7_LEIMU Putative 40S ribosomal protein S27-	6
4.315095328	4.941509	E9B473	tr|E9B473|E9B473_LEIMU Succinyl-CoA:3-ketoacid-coenzyme /	9
4.37007184	4.934802	E9ANP0	tr|E9ANP0|E9ANP0_LEIMU 40S ribosomal protein S5 OS=Leishi	8
3.478027071	4.933559	E9AQE0	tr|E9AQE0|E9AQE0_LEIMU Proliferating cell nuclear antigen O	6
4.178227756	4.933235	E9AQD3	tr|E9AQD3|E9AQD3_LEIMU Putative nucleolar RNA binding pro	17
3.724735036	4.933114	E9ARX0	tr|E9ARX0|E9ARX0_LEIMU Uncharacterized protein OS=Leishm	4
4.132377333	4.928553	E9AZT9	tr|E9AZT9|E9AZT9_LEIMU Putative glucose-regulated protein 7	24
4.222322313	4.923381	E9AQ19	tr|E9AQ19|E9AQ19_LEIMU Putative 60S ribosomal protein L13	6
4.356449947	4.917143	E9AL78	tr|E9AL78|E9AL78_LEIMU Calcium-transporting ATPase OS=Lei	4
4.121919073	4.91675	E9B4I1;A7U	tr|E9B4I1|E9B4I1_LEIMU Malate dehydrogenase OS=Leishman	11

3.555658476	4.915267	E9AK98	tr|E9AK98|E9AK98_LEIMU Putative RNA-binding protein OS=Le	4
4.643970996	4.915213	E9AWK3	tr|E9AWK3|E9AWK3_LEIMU Ankyrin/TPR repeat protein OS=Le	4
4.257242149	4.910129	E9B1Y7;E9B	tr|E9B1Y7|E9B1Y7_LEIMU Mannosyltransferase-like protein (F	4
3.869091276	4.908611	E9AJD4	tr|E9AJD4|E9AJD4_LEIMU Putative fatty acyl CoA syntetase 1 C	5
3.557218765	4.906328	E9B659	tr|E9B659|E9B659_LEIMU Uncharacterized protein OS=Leishm	2
4.116474831	4.904662	E9AUA1;Q8	tr|E9AUA1|E9AUA1_LEIMU 2,3-bisphosphoglycerate-independ	11
4.460503283	4.902499	E9AUF4	tr|E9AUF4|E9AUF4_LEIMU Uncharacterized protein OS=Leishm	3
4.189375816	4.900973	E9AUN5	tr|E9AUN5|E9AUN5_LEIMU 1,2-dihydroxy-3-keto-5-methylthic	2
4.430441694	4.899571	E9ALU2	tr|E9ALU2|E9ALU2_LEIMU Putative carnitine/choline acetyltra	7
4.065912006	4.895404	E9AW84	tr|E9AW84|E9AW84_LEIMU 3-ketoacyl-CoA thiolase-like prote	10
4.817574526	4.894423	E9B2J1	tr|E9B2J1|E9B2J1_LEIMU Putative OSM3-like kinesin OS=Leishi	4
3.736819548	4.894353	E9ARV9	tr|E9ARV9|E9ARV9_LEIMU Uncharacterized protein OS=Leishm	4
4.04704754	4.89185	E9B6S6	tr|E9B6S6|E9B6S6_LEIMU Uncharacterized protein OS=Leishm	4
4.47677158	4.891267	E9B139	tr|E9B139|E9B139_LEIMU Uncharacterized protein OS=Leishm	3
4.010853742	4.887659	E9AZ13	tr|E9AZ13|E9AZ13_LEIMU Mitochondrial RNA binding protein	2
3.956920253	4.88659	E9ASN4	tr|E9ASN4|E9ASN4_LEIMU Short chain 3-hydroxyacyl-CoA deh	3
4.280778958	4.883558	E9B6U6	tr|E9B6U6|E9B6U6_LEIMU Uncharacterized protein OS=Leishm	2
4.492932504	4.883326	E9AXP1	tr|E9AXP1|E9AXP1_LEIMU Uncharacterized protein OS=Leishm	4
4.331599825	4.880981	E9AMV3	tr|E9AMV3|E9AMV3_LEIMU Uncharacterized protein OS=Leish	7
4.05758338	4.878036	E9AMW3	tr|E9AMW3|E9AMW3_LEIMU Mitochondrial RNA binding prot	6
4.452800042	4.87787	E9B1Q4	tr|E9B1Q4|E9B1Q4_LEIMU Biotin/lipoate protein ligase-like pr	7
4.637768532	4.875647	E9AZ04	tr|E9AZ04|E9AZ04_LEIMU Uncharacterized protein OS=Leishm	3
4.452550041	4.872516	E9B146	tr|E9B146|E9B146_LEIMU Conserved TPR domain protein OS=I	13
4.113569397	4.871903	E9ARV4	tr|E9ARV4|E9ARV4_LEIMU Uncharacterized protein OS=Leishm	5
4.209839282	4.871797	E9AM03	tr|E9AM03|E9AM03_LEIMU Putative U3 small nucleolar ribonu	8
4.127534175	4.871164	E9AN99	tr|E9AN99|E9AN99_LEIMU Peptidylprolyl isomerase OS=Leishr	4
4.438006843	4.870943	E9APF9	tr|E9APF9|E9APF9_LEIMU Uncharacterized protein OS=Leishm	4
4.45099943	4.852258	E9AL96	tr|E9AL96|E9AL96_LEIMU Uncharacterized protein OS=Leishm	2
4.214131908	4.850253	E9ATK5	tr|E9ATK5|E9ATK5_LEIMU Uncharacterized protein OS=Leishm	3
3.68846551	4.845647	E9ALX8	tr|E9ALX8|E9ALX8_LEIMU Uncharacterized protein OS=Leishm	2
3.740033297	4.845043	E8NHI9;E9A	tr|E8NHI9|E8NHI9_LEIMU WGS CADB00000000 data, contig 40	2
4.24468878	4.844882	E9AMG3	tr|E9AMG3|E9AMG3_LEIMU DNA polymerase OS=Leishmania	5
3.710466106	4.843551	E9AM76	tr|E9AM76|E9AM76_LEIMU Ribosomal protein L3-like protein	3
4.335373303	4.840233	E9ALU9	tr|E9ALU9|E9ALU9_LEIMU Uncharacterized protein OS=Leishm	9
5.107721323	4.836508	E9ATP0	tr|E9ATP0|E9ATP0_LEIMU Uncharacterized protein OS=Leishm	3
3.396566313	4.827153	E9ALI5;E9AI	tr|E9ALI5|E9ALI5_LEIMU Putative kinesin OS=Leishmania mexi	2
4.052502906	4.826559	E9B064	tr|E9B064|E9B064_LEIMU Putative vacuolar ATP synthase subu	10
4.345679618	4.826451	E9B1G4	tr|E9B1G4|E9B1G4_LEIMU DNA-directed RNA polymerase subu	6
4.26509077	4.82471	E9AVW5	tr|E9AVW5|E9AVW5_LEIMU Uncharacterized protein OS=Leish	3
4.589168769	4.824518	E9AM67	tr|E9AM67|E9AM67_LEIMU Proteasome regulatory non-ATPas	5
3.998772115	4.823161	E9AR04	tr|E9AR04|E9AR04_LEIMU Uncharacterized protein OS=Leishm	3
4.843961766	4.820549	E9AW21	tr|E9AW21|E9AW21_LEIMU Putative endoribonuclease L-PSP (	2
4.316082115	4.819971	E9B4H8	tr|E9B4H8|E9B4H8_LEIMU Putative adenylate kinase OS=Leish	5
4.034788583	4.819025	E9AQ14	tr|E9AQ14|E9AQ14_LEIMU Uncharacterized protein OS=Leishm	4
4.803301596	4.818797	E9AV56	tr|E9AV56|E9AV56_LEIMU Methionine aminopeptidase 2 OS=L	5
4.308410674	4.817174	E9B6U3	tr|E9B6U3|E9B6U3_LEIMU Polyadenylate-binding protein OS=I	20
3.816656566	4.816447	E9AVR2	tr|E9AVR2|E9AVR2_LEIMU NADH-cytochrome b5 reductase OS	4
4.507252971	4.816055	E9B0Y0	tr|E9B0Y0|E9B0Y0_LEIMU Uncharacterized protein OS=Leishm	2
4.159890598	4.815881	E9APG0	tr|E9APG0|E9APG0_LEIMU Uncharacterized protein OS=Leishm	9
4.497071359	4.812813	E9B049	tr|E9B049|E9B049_LEIMU DNA topoisomerase 2 OS=Leishman	8
4.0472879	4.812383	E9AP92	tr|E9AP92|E9AP92_LEIMU Uncharacterized protein OS=Leishm	2
3.967928306	4.808016	E9ARJ2	tr|E9ARJ2|E9ARJ2_LEIMU Uncharacterized protein OS=Leishm	8

4.270319582	4.807805	E9AZ36	tr|E9AZ36|E9AZ36_LEIMU Uncharacterized protein OS=Leishm	17
5.161041422	4.807398	E9ANW9	tr|E9ANW9|E9ANW9_LEIMU Uncharacterized protein OS=Leisl	2
4.158125437	4.805698	E9ASX7	tr|E9ASX7|E9ASX7_LEIMU Chaperonin HSP60, mitochondrial O	10
4.235983953	4.803598	E9AMA8	tr|E9AMA8|E9AMA8_LEIMU Superoxide dismutase OS=Leishm	2
4.704479833	4.802929	E9B018	tr|E9B018|E9B018_LEIMU Uncharacterized protein OS=Leishm	9
4.418886755	4.802166	E9ASL3	tr|E9ASL3|E9ASL3_LEIMU Putative 40S ribosomal protein S18 (	22
3.912925874	4.801332	E9B400	tr|E9B400|E9B400_LEIMU Peptidyl-prolyl cis-trans isomerase (	4
4.328833073	4.800188	E9AM77	tr|E9AM77|E9AM77_LEIMU Transcription factor-like protein O	29
4.273141542	4.799866	E9AUQ7;E9/	tr|E9AUQ7|E9AUQ7_LEIMU Calpain-like cysteine peptidase OS	9
4.180301877	4.799721	E9AZR7	tr|E9AZR7|E9AZR7_LEIMU Putative ribosomal protein S20 OS=	5
4.453654857	4.798772	E9AZ28	tr|E9AZ28|E9AZ28_LEIMU Uncharacterized protein OS=Leishm	6
3.990394951	4.79822	E9B2M5	tr|E9B2M5|E9B2M5_LEIMU Chaperonin containing t-complex |	16
4.455658549	4.798162	E9APJ2	tr|E9APJ2|E9APJ2_LEIMU Pyrroline-5-carboxylate reductase OS	2
4.645386083	4.792133	E9B609	tr|E9B609|E9B609_LEIMU Putative ubiquitin-conjugating enzyı	2
4.117007169	4.791853	E9AQV6;H6'	tr|E9AQV6|E9AQV6_LEIMU Paraflagellar rod protein 2C OS=Le	2
3.726842399	4.785479	E9AWL4	tr|E9AWL4|E9AWL4_LEIMU Putative ATP-dependent RNA helic	3
4.69096878	4.784026	Q8MTW1;E!	tr|Q8MTW1|Q8MTW1_LEIAM Meta 2 protein OS=Leishmania a	5
5.137824634	4.777753	E9ARY1	tr|E9ARY1|E9ARY1_LEIMU Putative RNA binding protein OS=Le	4
4.248034813	4.776988	E9AVE2	tr|E9AVE2|E9AVE2_LEIMU Uncharacterized protein OS=Leishm	8
4.050729281	4.773046	E9AZE9	tr|E9AZE9|E9AZE9_LEIMU Putative heat shock protein DNAJ OS	11
4.130179722	4.770829	E9AWM1	tr|E9AWM1|E9AWM1_LEIMU Putative fumarate hydratase OS	3
5.557499299	4.770423	E9AUQ2	tr|E9AUQ2|E9AUQ2_LEIMU DNAj-like protein OS=Leishmania r	2
4.506284598	4.767295	E9AXQ3	tr|E9AXQ3|E9AXQ3_LEIMU Pyruvate dehydrogenase E1 compc	7
4.290607706	4.767033	E9B544	tr|E9B544|E9B544_LEIMU Uncharacterized protein OS=Leishm	2
4.132812739	4.765162	E9AUV1	tr|E9AUV1|E9AUV1_LEIMU Uncharacterized protein OS=Leishr	3
3.455861803	4.761429	E9B1E3	tr|E9B1E3|E9B1E3_LEIMU Uncharacterized protein OS=Leishm	3
3.807954584	4.760477	E9AZA7;E9A	tr|E9AZA7|E9AZA7_LEIMU Branched-chain-amino-acid aminotı	4
5.755390779	4.757569	E9B1C1	tr|E9B1C1|E9B1C1_LEIMU Uncharacterized protein OS=Leishm	2
4.31506708	4.755695	E9AJY3;O09	tr|E9AJY3|E9AJY3_LEIMU Calcium-transporting ATPase OS=Lei:	8
4.312851669	4.754278	E9ASQ6	tr|E9ASQ6|E9ASQ6_LEIMU Transitional endoplasmic reticulum	19
4.32069055	4.751569	E9ARL1	tr|E9ARL1|E9ARL1_LEIMU Putative elongation factor Tu OS=Le	7
3.989121734	4.750888	E9ASJ4	tr|E9ASJ4|E9ASJ4_LEIMU Uncharacterized protein OS=Leishma	8
4.163751935	4.749356	E9B3P3	tr|E9B3P3|E9B3P3_LEIMU Uncharacterized protein OS=Leishm	2
4.583997083	4.748606	E9APP0	tr|E9APP0|E9APP0_LEIMU Uncharacterized protein OS=Leishm	5
4.472295356	4.748371	E9B361	tr|E9B361|E9B361_LEIMU Proteasome regulatory non-ATP-ase	3
4.352030393	4.747399	E9AZ37	tr|E9AZ37|E9AZ37_LEIMU Putative arginyl-tRNA synthetase OS	14
4.373579468	4.74623	E9ARI4	tr|E9ARI4|E9ARI4_LEIMU Uncharacterized protein OS=Leishma	2
4.008028998	4.745887	E9B0E2	tr|E9B0E2|E9B0E2_LEIMU Putative 2-hydroxy-3-oxopropionate	2
4.59234154	4.744819	E9AP18	tr|E9AP18|E9AP18_LEIMU Uncharacterized protein OS=Leishm	2
4.451566289	4.743181	E9ATS5	tr|E9ATS5|E9ATS5_LEIMU Uncharacterized protein OS=Leishm	3
4.796998591	4.741402	Q25310	tr|Q25310|Q25310_LEIME Paraflagellar rod protein 2C OS=Leis	2
4.411757141	4.740721	E9ALK3	tr|E9ALK3|E9ALK3_LEIMU Putative GTP-binding protein OS=Lei	8
4.468890962	4.7401	E9AK75	tr|E9AK75|E9AK75_LEIMU Uncharacterized protein OS=Leishm	4
4.281610217	4.737068	E9ALI1	tr|E9ALI1|E9ALI1_LEIMU Uncharacterized protein OS=Leishmaı	3
3.949644842	4.733994	E9B5P0	tr|E9B5P0|E9B5P0_LEIMU Pyruvate kinase (Fragment) OS=Leis	2
4.131132804	4.733792	E9ASP6;Q9U	tr|E9ASP6|E9ASP6_LEIMU Fructose-bisphosphate aldolase OS=	19
4.210599153	4.728694	E9B2X4	tr|E9B2X4|E9B2X4_LEIMU Putative 1-acyl-sn-glycerol-3-phospl	5
3.726285463	4.728356	E9APE3	tr|E9APE3|E9APE3_LEIMU Adenylosuccinate synthetase OS=Le	9
3.995485466	4.719959	E9AM02	tr|E9AM02|E9AM02_LEIMU Putative lipophosphoglycan biosyı	8
4.241761834	4.719142	E9B297	tr|E9B297|E9B297_LEIMU Putative acetyl-CoA carboxylase OS=	24
4.720620709	4.717098	E9B573	tr|E9B573|E9B573_LEIMU Regulatory subunit of protein kinase	13
4.45685294	4.71664	E9B178	tr|E9B178|E9B178_LEIMU Eukaryotic translation initiation fact	10

4.180188875	4.715325	E9AK23	tr|E9AK23|E9AK23_LEIMU Uncharacterized protein OS=Leishm	2
4.171894828	4.714101	E9AUX6	tr|E9AUX6|E9AUX6_LEIMU Uncharacterized protein OS=Leishn	2
4.099138416	4.713525	E9AMZ4	tr|E9AMZ4|E9AMZ4_LEIMU Uncharacterized protein OS=Leish	4
4.298055191	4.712246	E9B170;Q27	tr|E9B170|E9B170_LEIMU Glyceraldehyde-3-phosphate dehyd	17
4.475707189	4.709979	E9AVH4	tr|E9AVH4|E9AVH4_LEIMU 40S ribosomal protein S6 OS=Leish	14
4.653104719	4.708751	E9AQL3	tr|E9AQL3|E9AQL3_LEIMU Putative orotidine-5-phosphate dec	3
4.995987345	4.708531	E9AYV9	tr|E9AYV9|E9AYV9_LEIMU Uncharacterized protein OS=Leishm	2
4.19844227	4.707336	E9B384	tr|E9B384|E9B384_LEIMU Uncharacterized protein OS=Leishm	11
4.186671723	4.706168	E9ASC5	tr|E9ASC5|E9ASC5_LEIMU Stress-inducible protein STI1 homol	6
4.767854983	4.705355	E9B0P0	tr|E9B0P0|E9B0P0_LEIMU Putative RNA-binding protein OS=Le	7
4.134399725	4.70444	Q5MY89;E9	tr|Q5MY89|Q5MY89_LEIAM Replication protein A subunit OS=	9
4.308213219	4.702472	E9ANM6	tr|E9ANM6|E9ANM6_LEIMU Uncharacterized protein OS=Leish	9
4.0492794	4.701459	E9AZ73	tr|E9AZ73|E9AZ73_LEIMU Putative eukaryotic translation relea	9
4.274742506	4.699359	E9AN57;E9A	tr|E9AN57|E9AN57_LEIMU GP63, leishmanolysin OS=Leishmar	5
4.241523675	4.697689	E9AK49;E9A	tr|E9AK49|E9AK49_LEIMU Uncharacterized protein OS=Leishm	2
4.778622729	4.695471	E9B007	tr|E9B007|E9B007_LEIMU Uncharacterized protein OS=Leishm	6
4.373305388	4.694984	E9B376;A0A	tr|E9B376|E9B376_LEIMU Nucleoside diphosphate kinase OS=I	9
4.277804521	4.694686	E9B2K8	tr|E9B2K8|E9B2K8_LEIMU Uncharacterized protein OS=Leishm	10
3.824507085	4.694287	E9AR65	tr|E9AR65|E9AR65_LEIMU Putative ATP-dependent RNA helica	2
4.863135481	4.690069	E9AT16;E9A	tr|E9AT16|E9AT16_LEIMU Methyltransferase OS=Leishmania n	5
4.895252159	4.68851	E9ALE1	tr|E9ALE1|E9ALE1_LEIMU Uncharacterized protein OS=Leishma	4
4.131378536	4.687196	E9B686	tr|E9B686|E9B686_LEIMU Putative calcium motive P-type ATPa	6
3.668242595	4.686811	E9B4N7	tr|E9B4N7|E9B4N7_LEIMU Uncharacterized protein OS=Leishm	4
4.308695384	4.686609	E9AQ01	tr|E9AQ01|E9AQ01_LEIMU Uncharacterized protein OS=Leishn	2
4.475384478	4.684926	E9ATX0	tr|E9ATX0|E9ATX0_LEIMU 2-methoxy-6-polyprenyl-1,4-benzoc	2
3.695544242	4.684751	E9AW96	tr|E9AW96|E9AW96_LEIMU Actin interacting protein-like prot	2
4.76419821	4.684366	E9B5X5	tr|E9B5X5|E9B5X5_LEIMU Uncharacterized protein OS=Leishm	4
5.090347859	4.684258	E9ASG7	tr|E9ASG7|E9ASG7_LEIMU Putative DNAJ domain protein OS=L	3
5.082354563	4.679578	E9B733	tr|E9B733|E9B733_LEIMU Uncharacterized protein OS=Leishm	2
4.167474861	4.678015	E9AWR7	tr|E9AWR7|E9AWR7_LEIMU Malic enzyme OS=Leishmania me:	9
4.422039682	4.677468	E9B3G5	tr|E9B3G5|E9B3G5_LEIMU Putative myosin heavy chain OS=Le	4
4.758030765	4.674883	E9ATD2;E9E	tr|E9ATD2|E9ATD2_LEIMU Putative polyubiquitin (Fragment) (	6
5.009621339	4.673807	E9AVB3	tr|E9AVB3|E9AVB3_LEIMU Putative vesicule-associated memb	3
4.41701483	4.669634	E9AXD1	tr|E9AXD1|E9AXD1_LEIMU Uncharacterized protein OS=Leishn	9
4.530186592	4.668844	E9B0I8	tr|E9B0I8|E9B0I8_LEIMU Putative histidyl-tRNA synthetase OS=	5
4.173037155	4.667344	E9B4K3	tr|E9B4K3|E9B4K3_LEIMU Eukaryotic translation initiation fact	14
4.379299552	4.666704	E9APV6	tr|E9APV6|E9APV6_LEIMU Putative kinesin K39 OS=Leishmania	4
4.113477618	4.665544	E9AJZ0	tr|E9AJZ0|E9AJZ0_LEIMU DNA topoisomerase type IB small sub	11
4.830315828	4.662228	E9AR51	tr|E9AR51|E9AR51_LEIMU Ysine decarboxylase-like protein OS	5
4.378623841	4.655605	E9B1B3	tr|E9B1B3|E9B1B3_LEIMU Phosphoglycerate kinase OS=Leishn	2
4.388779262	4.654675	E9ALD8;E9B	tr|E9ALD8|E9ALD8_LEIMU 40S ribosomal protein S19-like prot	7
4.386970168	4.652517	E9B4A1	tr|E9B4A1|E9B4A1_LEIMU Mitochondrial processing peptidase	2
4.18977966	4.651601	E9ALK4	tr|E9ALK4|E9ALK4_LEIMU Uncharacterized protein OS=Leishm	8
4.172099425	4.647217	E9ATC6	tr|E9ATC6|E9ATC6_LEIMU 2-oxoglutarate dehydrogenase E1 c	8
4.182302173	4.645184	E9B1F7	tr|E9B1F7|E9B1F7_LEIMU Uncharacterized protein OS=Leishm	2
5.287144113	4.644109	E9AKW6	tr|E9AKW6|E9AKW6_LEIMU Putative acyl-coenzyme a dehydrc	3
4.370785281	4.643485	E9AT02;A0A	tr|E9AT02|E9AT02_LEIMU Developmentally regulated GTP-bin	6
4.090157167	4.642432	E9AZ71	tr|E9AZ71|E9AZ71_LEIMU Uncharacterized protein OS=Leishm	5
4.230054302	4.642331	E9ALM8	tr|E9ALM8|E9ALM8_LEIMU Putative fumarate hydratase (Frag	2
4.239918453	4.642256	E9AZE0	tr|E9AZE0|E9AZE0_LEIMU Obg-like ATPase 1 OS=Leishmania m	4
3.827937239	4.641953	E9AZV4	tr|E9AZV4|E9AZV4_LEIMU Haloacid dehalogenase-like hydrola	2
4.316564547	4.641394	Q86PL4;E9A	tr|Q86PL4|Q86PL4_LEIME Paraflagellar rod protein 1D OS=Leis	22

3.847213884	4.639051	E9AQ51	tr|E9AQ51|E9AQ51_LEIMU Uncharacterized protein OS=Leishr	3
4.781985743	4.638579	E9AKP2	tr|E9AKP2|E9AKP2_LEIMU Proteasome subunit beta OS=Leishr	2
5.444502683	4.634933	E9B0P1	tr|E9B0P1|E9B0P1_LEIMU Importin subunit alpha OS=Leishma	3
4.606347611	4.633944	E9B617	tr|E9B617|E9B617_LEIMU Mitochondrial processing peptidase	12
3.463468788	4.633626	E9B496	tr|E9B496|E9B496_LEIMU Putative aminopeptidase OS=Leishm	3
4.098074126	4.631641	E9APL2	tr|E9APL2|E9APL2_LEIMU Uncharacterized protein OS=Leishm	6
4.40697876	4.630817	E9API9	tr|E9API9|E9API9_LEIMU Putative dynein heavy chain OS=Leish	18
4.044959927	4.629406	E9B3K6	tr|E9B3K6|E9B3K6_LEIMU Uncharacterized protein OS=Leishm	2
4.352045518	4.626033	E9B2V1	tr|E9B2V1|E9B2V1_LEIMU Uncharacterized protein OS=Leishm	7
4.552039988	4.62569	E9B528	tr|E9B528|E9B528_LEIMU Glycosyltransferase-like protein OS=	2
5.016306329	4.622611	E9AP67	tr|E9AP67|E9AP67_LEIMU Putative flagellar radial spoke prote	2
4.574620285	4.622574	E9AKB4	tr|E9AKB4|E9AKB4_LEIMU Serine/threonine-protein phosphata	4
5.019657317	4.622531	E9AWL9	tr|E9AWL9|E9AWL9_LEIMU Uncharacterized protein OS=Leishi	2
4.531673384	4.622035	E9AJY2;A0A	tr|E9AJY2|E9AJY2_LEIMU Elongation initiation factor 2 alpha su	14
4.457453919	4.621164	E9ARK4	tr|E9ARK4|E9ARK4_LEIMU Uncharacterized protein OS=Leishm	4
4.090127315	4.618827	E9B3Z8	tr|E9B3Z8|E9B3Z8_LEIMU Putative peptidase M20/M25/M40 (	7
4.701009415	4.617833	E9AZQ3	tr|E9AZQ3|E9AZQ3_LEIMU Uncharacterized protein OS=Leishn	2
3.482446215	4.61736	E9ALC3	tr|E9ALC3|E9ALC3_LEIMU Cation-transporting ATPase OS=Leis	4
5.290253359	4.617279	E9AL69	tr|E9AL69|E9AL69_LEIMU Uncharacterized protein OS=Leishm	3
4.258702664	4.616615	E9AVY6	tr|E9AVY6|E9AVY6_LEIMU Alanine--tRNA ligase OS=Leishmania	17
4.452341703	4.615842	E9B099;Q07	tr|E9B099|E9B099_LEIMU Putative heat-shock protein hsp70 C	46
4.453979527	4.615142	E9B2V7	tr|E9B2V7|E9B2V7_LEIMU Uncharacterized protein OS=Leishm	3
4.47231514	4.615122	E9ANZ4	tr|E9ANZ4|E9ANZ4_LEIMU Cytochrome c oxidase subunit IV OS	11
4.642062489	4.615051	E9AZA6	tr|E9AZA6|E9AZA6_LEIMU D-lactate dehydrogenase-like prote	2
4.131261831	4.614142	E9ASL8;E9A	tr|E9ASL8|E9ASL8_LEIMU Putative 40S ribosomal protein S10 (	7
4.970636567	4.613015	E9AMV4	tr|E9AMV4|E9AMV4_LEIMU Uncharacterized protein OS=Leish	2
4.395036051	4.612586	E9ATF3	tr|E9ATF3|E9ATF3_LEIMU Uncharacterized protein OS=Leishm	2
3.892430128	4.612327	E9B1R0	tr|E9B1R0|E9B1R0_LEIMU Putative N-acyl-L-amino acid amidol	3
5.271827108	4.612264	E9AST1	tr|E9AST1|E9AST1_LEIMU Proteasome subunit alpha type OS=	6
3.841407452	4.609692	E9AS04	tr|E9AS04|E9AS04_LEIMU Uncharacterized protein OS=Leishm	3
4.109706831	4.60931	E9AW71	tr|E9AW71|E9AW71_LEIMU Kinesin-like protein OS=Leishmani	11
4.359268533	4.608993	E9AS67	tr|E9AS67|E9AS67_LEIMU Uncharacterized protein OS=Leishm	11
4.628913559	4.605492	E9B1D9	tr|E9B1D9|E9B1D9_LEIMU Ribosomal protein L15 OS=Leishma	2
4.470368179	4.605452	E9AZ83;E9A	tr|E9AZ83|E9AZ83_LEIMU Glycosomal phosphoenolpyruvate ca	15
4.378582912	4.602144	E9AW04;Q4	tr|E9AW04|E9AW04_LEIMU Peroxidoxin (Tryparedoxin peroxic	5
4.324335318	4.600704	V5RIA5;E9A	tr|V5RIA5|V5RIA5_LEIAM Calmodulin-like protein OS=Leishmai	8
4.023490083	4.598209	E9B3P7	tr|E9B3P7|E9B3P7_LEIMU Uncharacterized protein OS=Leishm	4
3.972575032	4.596599	P36400;Q05	sp|P36400|LMCPB_LEIME Cysteine proteinase B OS=Leishmani	3
4.571287216	4.592189	E9AN60	tr|E9AN60|E9AN60_LEIMU Uncharacterized protein OS=Leishn	10
4.673679082	4.590341	E9AKK2	tr|E9AKK2|E9AKK2_LEIMU Dipeptidyl peptidase 3 OS=Leishmai	13
4.298772399	4.589561	E9AML1	tr|E9AML1|E9AML1_LEIMU Uncharacterized protein OS=Leishi	10
4.310421868	4.589346	E9B226	tr|E9B226|E9B226_LEIMU Putative 3,2-trans-enoyl-CoA isomei	7
4.534353081	4.583811	E9ATH3	tr|E9ATH3|E9ATH3_LEIMU Adenosylhomocysteinase OS=Leish	23
4.228264941	4.581954	E9B6I9	tr|E9B6I9|E9B6I9_LEIMU Putative ATP-dependent RNA helicase	27
4.265343592	4.581874	E9AQH3	tr|E9AQH3|E9AQH3_LEIMU Putative 50S ribosomal protein L17	2
4.0503584	4.580009	E9AJD6	tr|E9AJD6|E9AJD6_LEIMU Putative long-chain-fatty-acid-CoA li	4
4.775038194	4.579861	E9AMD0	tr|E9AMD0|E9AMD0_LEIMU Putative ribose-phosphate pyropł	2
4.48312826	4.579268	E9AL72	tr|E9AL72|E9AL72_LEIMU Putative electron transfer flavoprote	3
4.214225965	4.578158	E9AUA3	tr|E9AUA3|E9AUA3_LEIMU Uncharacterized protein OS=Leishr	3
4.154820142	4.576345	E9ATU4	tr|E9ATU4|E9ATU4_LEIMU Uncharacterized protein OS=Leishn	31
5.044471868	4.574388	E9ANP7	tr|E9ANP7|E9ANP7_LEIMU Uncharacterized protein OS=Leishn	9
4.333426305	4.571874	E9AT99	tr|E9AT99|E9AT99_LEIMU 14-3-3 protein-like protein OS=Leish	7

4.406229742	4.571036	E9AST7	tr|E9AST7|E9AST7_LEIMU Proteasome subunit beta OS=Leishn	2
4.511206903	4.570394	E9AL39	tr|E9AL39|E9AL39_LEIMU Uncharacterized protein OS=Leishma	3
4.776030541	4.569565	E9AQA6;Q4	tr|E9AQA6|E9AQA6_LEIMU Tryparedoxin peroxidase OS=Leish	3
4.199134314	4.56807	E9AY65	tr|E9AY65|E9AY65_LEIMU Uncharacterized protein OS=Leishm	2
4.373039279	4.567862	Q9NGK2;E9	tr|Q9NGK2|Q9NGK2_LEIAM 40S ribosomal protein S24 OS=Lei	7
4.895654366	4.56665	E9AY45;A7Y	tr|E9AY45|E9AY45_LEIMU Uncharacterized protein OS=Leishm	7
4.769681683	4.566345	E9AXP2	tr|E9AXP2|E9AXP2_LEIMU Uncharacterized protein OS=Leishm	5
4.467012945	4.56483	E9AJG4	tr|E9AJG4|E9AJG4_LEIMU Uncharacterized protein OS=Leishm	24
4.352971835	4.564827	E9B3A4	tr|E9B3A4|E9B3A4_LEIMU Uncharacterized protein OS=Leishm	3
4.443717841	4.564226	E9B0G6	tr|E9B0G6|E9B0G6_LEIMU WD-40 repeat protein OS=Leishmai	2
4.865994454	4.563367	E9AL58	tr|E9AL58|E9AL58_LEIMU Uncharacterized protein OS=Leishm	3
4.938435709	4.56335	E9APY0	tr|E9APY0|E9APY0_LEIMU Serine hydroxymethyltransferase O	3
4.743693284	4.559905	E9B6Q4	tr|E9B6Q4|E9B6Q4_LEIMU Putative 60S ribosomal protein L27	6
4.150940071	4.556869	E9AV43	tr|E9AV43|E9AV43_LEIMU Uncharacterized protein OS=Leishm	3
3.83648692	4.55521	E9AZX3	tr|E9AZX3|E9AZX3_LEIMU Putative DEAD box RNA helicase OS	4
5.067965189	4.552566	E9B6J9	tr|E9B6J9|E9B6J9_LEIMU Uncharacterized protein OS=Leishma	2
4.398117041	4.550439	E9B6D4	tr|E9B6D4|E9B6D4_LEIMU Putative RNA binding protein OS=Le	2
3.966760099	4.550187	E9B2R2	tr|E9B2R2|E9B2R2_LEIMU Uncharacterized protein OS=Leishm	5
4.572267555	4.543656	E9B3L2;P27	tr|E9B3L2|E9B3L2_LEIMU Heat shock protein 83-1 OS=Leishma	50
4.155912501	4.543582	E9ANN2	tr|E9ANN2|E9ANN2_LEIMU Uncharacterized protein OS=Leishi	2
4.322635603	4.536472	E9AQ29	tr|E9AQ29|E9AQ29_LEIMU Ribonucleoprotein p18, mitochond	10
4.789584736	4.536442	E9AXU7;E9/	tr|E9AXU7|E9AXU7_LEIMU Succinate--CoA ligase [ADP-forming	9
4.506232142	4.534663	E9ASD6	tr|E9ASD6|E9ASD6_LEIMU Elongation factor 2 OS=Leishmania	50
4.430104568	4.53367	E9AQP7;E8I	tr|E9AQP7|E9AQP7_LEIMU Uncharacterized protein OS=Leishr	5
4.646500511	4.532225	E9B2L9	tr|E9B2L9|E9B2L9_LEIMU Uncharacterized protein OS=Leishma	13
4.604435802	4.531846	E9AYZ0	tr|E9AYZ0|E9AYZ0_LEIMU Putative 2-oxoglutarate dehydrogen	11
4.120642992	4.53111	E9AVJ6	tr|E9AVJ6|E9AVJ6_LEIMU Putative GMP synthase (Putative glu	7
4.760262085	4.531003	E9ANW2	tr|E9ANW2|E9ANW2_LEIMU Uncharacterized protein OS=Leisl	4
4.634180289	4.530163	E9AZS3	tr|E9AZS3|E9AZS3_LEIMU Uncharacterized protein OS=Leishm	10
4.321326273	4.529904	E9AX72	tr|E9AX72|E9AX72_LEIMU Putative notchless homolog OS=Leis	2
4.627986217	4.529893	E9B2G8	tr|E9B2G8|E9B2G8_LEIMU Uncharacterized protein OS=Leishm	3
4.679311589	4.528704	E9ATI7	tr|E9ATI7|E9ATI7_LEIMU Uncharacterized protein OS=Leishma	3
4.274778363	4.528516	E9B028	tr|E9B028|E9B028_LEIMU Putative mitochondrial DEAD box pr	4
3.916794663	4.52803	E9AL57	tr|E9AL57|E9AL57_LEIMU Acyl-CoA dehydrogenase, mitochon	4
4.300439674	4.528011	E9B183	tr|E9B183|E9B183_LEIMU Uncharacterized protein OS=Leishm	12
4.483876353	4.527052	E9B713	tr|E9B713|E9B713_LEIMU Proteasome subunit alpha type OS=	7
4.48805083	4.526699	E9AQC3	tr|E9AQC3|E9AQC3_LEIMU Putative cation transporter OS=Lei	3
5.09763158	4.524947	E9AZE8	tr|E9AZE8|E9AZE8_LEIMU Putative TPR-repeat protein OS=Leis	3
4.326845868	4.523338	E9AUC7	tr|E9AUC7|E9AUC7_LEIMU Putative T-complex protein 1, theta	18
4.30235345	4.521626	E9ARX7	tr|E9ARX7|E9ARX7_LEIMU Kinesin-like protein OS=Leishmania	4
4.569753831	4.520978	E9AJJ4	tr|E9AJJ4|E9AJJ4_LEIMU Uncharacterized protein OS=Leishmai	7
4.252855992	4.517017	E9AU76	tr|E9AU76|E9AU76_LEIMU Putative 40S ribosomal protein S8 (	3
4.362345566	4.51598	E9AQV4	tr|E9AQV4|E9AQV4_LEIMU Uncharacterized protein OS=Leishr	10
4.564144795	4.513886	E9B307	tr|E9B307|E9B307_LEIMU Uncharacterized protein OS=Leishm	4
3.741299096	4.513215	E9APT0	tr|E9APT0|E9APT0_LEIMU Putative calpain-like cysteine peptid	3
4.383589703	4.512806	E9AUV6;E9/	tr|E9AUV6|E9AUV6_LEIMU Putative aminoacylase OS=Leishma	4
4.28505352	4.51221	E9AU41	tr|E9AU41|E9AU41_LEIMU Uncharacterized protein OS=Leishn	12
3.993942242	4.510833	E9AQK9	tr|E9AQK9|E9AQK9_LEIMU Uncharacterized protein OS=Leishr	2
4.412774359	4.510586	E9AUQ0	tr|E9AUQ0|E9AUQ0_LEIMU Uncharacterized protein OS=Leishi	8
4.577137029	4.51048	E9ALZ2	tr|E9ALZ2|E9ALZ2_LEIMU High mobility group protein homolo	14
4.787542083	4.510458	E9AXR4	tr|E9AXR4|E9AXR4_LEIMU Uncharacterized protein OS=Leishm	8
4.355122782	4.510387	E9AV52	tr|E9AV52|E9AV52_LEIMU Uncharacterized protein OS=Leishm	9

4.388313453	4.509586	E9AK60;E9A	tr|E9AK60|E9AK60_LEIMU Uncharacterized protein OS=Leishm	4
4.142871198	4.508958	E9ASU7	tr|E9ASU7|E9ASU7_LEIMU Uncharacterized protein OS=Leishm	2
3.761378081	4.507796	E9B1K6	tr|E9B1K6|E9B1K6_LEIMU Mevalonate kinase OS=Leishmania r	3
4.565850364	4.507367	E9B2E2	tr|E9B2E2|E9B2E2_LEIMU Uncharacterized protein OS=Leishm	19
4.332435544	4.503531	E9B3B4	tr|E9B3B4|E9B3B4_LEIMU Dihydrolipoyl dehydrogenase OS=Le	18
3.965301979	4.503512	E9B549	tr|E9B549|E9B549_LEIMU Uncharacterized protein OS=Leishm	12
4.515036841	4.503093	E9AW01	tr|E9AW01|E9AW01_LEIMU Uncharacterized protein OS=Leish	3
5.063996469	4.500839	E9AXM1	tr|E9AXM1|E9AXM1_LEIMU GTP-binding nuclear protein OS=L	10
4.347831871	4.500175	E9B5M6	tr|E9B5M6|E9B5M6_LEIMU Coatomer subunit alpha OS=Leish	6
4.338029168	4.498854	E9APZ2	tr|E9APZ2|E9APZ2_LEIMU Uncharacterized protein OS=Leishm	30
4.38229603	4.497511	E9ALJ9	tr|E9ALJ9|E9ALJ9_LEIMU Aminopeptidase OS=Leishmania mex	2
3.857564154	4.497166	E9B2F2	tr|E9B2F2|E9B2F2_LEIMU Uncharacterized protein OS=Leishm	3
4.787096547	4.493857	E9B2E6	tr|E9B2E6|E9B2E6_LEIMU Uncharacterized protein OS=Leishm	2
4.307947472	4.493536	E9B5E9	tr|E9B5E9|E9B5E9_LEIMU Adenosine kinase-like protein OS=Le	2
4.678297868	4.492359	E9B215	tr|E9B215|E9B215_LEIMU Prostaglandin f2-alpha synthase OS=	3
4.581751745	4.492131	E9ASS0	tr|E9ASS0|E9ASS0_LEIMU Uncharacterized protein OS=Leishm	2
4.8708618	4.491774	E9AZU1	tr|E9AZU1|E9AZU1_LEIMU Uncharacterized protein OS=Leishm	11
4.944646776	4.489501	E9AYY9	tr|E9AYY9|E9AYY9_LEIMU Uncharacterized protein OS=Leishm	7
4.13486041	4.489487	E9AZM2	tr|E9AZM2|E9AZM2_LEIMU Putative dynein heavy chain OS=Le	3
4.616591595	4.487623	E9AM16	tr|E9AM16|E9AM16_LEIMU Putative ABC transporter OS=Leish	10
3.657166352	4.486621	E9B162	tr|E9B162|E9B162_LEIMU Uncharacterized protein OS=Leishm	2
4.682221003	4.485247	E9AWU0	tr|E9AWU0|E9AWU0_LEIMU Putative replication factor C, subu	9
4.455085822	4.484336	E9ATD8	tr|E9ATD8|E9ATD8_LEIMU Putative cysteine synthase OS=Leisl	11
4.392620836	4.479818	E9AVS9	tr|E9AVS9|E9AVS9_LEIMU Uncharacterized protein OS=Leishm	2
4.445492184	4.479291	E8NHG6;E8I	tr|E8NHG6|E8NHG6_LEIMU Uncharacterized protein LmxM_30	22
4.730807728	4.479164	E9B0M2	tr|E9B0M2|E9B0M2_LEIMU Uncharacterized protein OS=Leish	2
4.488317531	4.478458	E9AVP4	tr|E9AVP4|E9AVP4_LEIMU Putative proteasome regulatory ATI	7
3.981629281	4.477621	E9AM68	tr|E9AM68|E9AM68_LEIMU Uncharacterized protein OS=Leish	5
4.607612649	4.475669	E9B4Z0;P90	tr|E9B4Z0|E9B4Z0_LEIMU ATP-dependent RNA helicase OS=Le	4
4.267275889	4.475094	E9AYH5	tr|E9AYH5|E9AYH5_LEIMU Putative mitotubule-associated pro	3
4.45583343	4.47241	E9AXJ6;E9A	tr|E9AXJ6|E9AXJ6_LEIMU ATP synthase subunit beta OS=Leish	25
4.754131178	4.46966	E9B343	tr|E9B343|E9B343_LEIMU Putative cystathionine beta-lyase OS	4
4.366792198	4.468731	E9AV58	tr|E9AV58|E9AV58_LEIMU Hypoxanthine phosphoribosyltransf	2
4.308329543	4.467188	E9AKB8	tr|E9AKB8|E9AKB8_LEIMU Putative nucleolar RNA helicase II O	7
4.270628949	4.463538	E9B6Z8	tr|E9B6Z8|E9B6Z8_LEIMU Uncharacterized protein OS=Leishm	2
4.372022031	4.461118	E9AX96	tr|E9AX96|E9AX96_LEIMU Uncharacterized protein OS=Leishm	3
4.432936976	4.460939	E9ANC1	tr|E9ANC1|E9ANC1_LEIMU Uncharacterized protein OS=Leishm	7
4.482964121	4.459012	E9AK97	tr|E9AK97|E9AK97_LEIMU Putative fructose-1,6-bisphosphatas	4
4.503298373	4.452558	E9AWE0	tr|E9AWE0|E9AWE0_LEIMU T-complex protein 1 subunit gamr	18
4.298516483	4.451755	E9APH0	tr|E9APH0|E9APH0_LEIMU Programmed cell death 6 protein-li	2
4.31811569	4.45104	E9AJY9	tr|E9AJY9|E9AJY9_LEIMU Uncharacterized protein OS=Leishma	7
4.182939566	4.450441	Q9BLW4;E9	tr|Q9BLW4|Q9BLW4_LEIME GDP-mannose pyrophosphorylase	3
4.720223382	4.450234	E9AU22	tr|E9AU22|E9AU22_LEIMU Uncharacterized protein OS=Leishm	4
4.534735569	4.44954	E9AM10	tr|E9AM10|E9AM10_LEIMU Putative RNA-binding protein OS=	6
4.841322177	4.447065	E9ATE6	tr|E9ATE6|E9ATE6_LEIMU Uncharacterized protein OS=Leishm	6
4.450553445	4.446656	E9AST4	tr|E9AST4|E9AST4_LEIMU Clathrin heavy chain OS=Leishmania	22
3.978076459	4.446415	E9AYU4	tr|E9AYU4|E9AYU4_LEIMU Ribokinase OS=Leishmania mexican	5
5.927804415	4.446279	E9ARJ9;E9A	tr|E9ARJ9|E9ARJ9_LEIMU Putative ATP-dependent zinc metallo	2
4.680738542	4.44444	E9ATM0	tr|E9ATM0|E9ATM0_LEIMU Proteasome regulatory ATPase sul	6
4.628928765	4.442332	E9B189	tr|E9B189|E9B189_LEIMU Uncharacterized protein OS=Leishm	3
4.380819534	4.441414	E9AR50	tr|E9AR50|E9AR50_LEIMU GMP reductase OS=Leishmania mex	6
4.433993678	4.439243	E9AZA2	tr|E9AZA2|E9AZA2_LEIMU Putative rRNA methyltransferase OS	7

4.573300109	4.438265	E9B191	tr|E9B191|E9B191_LEIMU Uncharacterized protein OS=Leishm	3
4.155536887	4.436174	E9AR29	tr|E9AR29|E9AR29_LEIMU Putative P-type ATPase OS=Leishma	3
3.714803495	4.435421	E9AMW9	tr|E9AMW9|E9AMW9_LEIMU Uncharacterized protein OS=Lei	3
4.498254275	4.433536	E9APE7;E9A	tr|E9APE7|E9APE7_LEIMU 40S ribosomal protein S4 OS=Leishn	20
4.377871967	4.432763	E9AK64	tr|E9AK64|E9AK64_LEIMU Pescadillo homolog OS=Leishmania	6
4.955571494	4.429458	E9AW66	tr|E9AW66|E9AW66_LEIMU Uncharacterized protein OS=Leish	2
4.520879363	4.429301	Q9GRU2;E9	tr|Q9GRU2|Q9GRU2_LEIME Mitogen-activated protein kinase	2
4.514404255	4.429047	E9AV93	tr|E9AV93|E9AV93_LEIMU T-complex protein 1 subunit delta C	15
4.563708181	4.427856	E9AUS9	tr|E9AUS9|E9AUS9_LEIMU Putative axoneme central apparatu	2
4.421099371	4.427815	E9ARY6	tr|E9ARY6|E9ARY6_LEIMU Uncharacterized protein OS=Leishm	2
4.689431517	4.427182	E9ANK8;E9A	tr|E9ANK8|E9ANK8_LEIMU Putative aminopeptidase (Metallo-	5
4.528836032	4.426241	E9AYH6	tr|E9AYH6|E9AYH6_LEIMU Uncharacterized protein OS=Leishm	22
4.635307465	4.42449	E9AT24	tr|E9AT24|E9AT24_LEIMU Putative acid phosphatase OS=Leish	4
4.254599811	4.422241	E9AT49	tr|E9AT49|E9AT49_LEIMU Putative mitochondrial ATP-depend	3
4.490653212	4.421295	E9ARK6;E9A	tr|E9ARK6|E9ARK6_LEIMU Citrate synthase OS=Leishmania me	8
4.641568724	4.419774	E9AJI7	tr|E9AJI7|E9AJI7_LEIMU Uncharacterized protein OS=Leishmar	2
4.30385079	4.418518	E9AVW9	tr|E9AVW9|E9AVW9_LEIMU Intraflagellar transport protein-lik	2
4.322960456	4.417589	E9AJY1	tr|E9AJY1|E9AJY1_LEIMU Uncharacterized protein OS=Leishma	3
5.29494103	4.417089	E9AQU6	tr|E9AQU6|E9AQU6_LEIMU DNA-directed rna polymerase I lar	7
4.476706692	4.416758	E9AUG6	tr|E9AUG6|E9AUG6_LEIMU Midasin OS=Leishmania mexicana	50
4.125678788	4.415422	E9AP30	tr|E9AP30|E9AP30_LEIMU Uncharacterized protein OS=Leishm	2
4.479545184	4.415207	E8NHJ8;E8N	tr|E8NHJ8|E8NHJ8_LEIMU 60S acidic ribosomal protein P0 OS=	12
4.329016325	4.414466	E9B063	tr|E9B063|E9B063_LEIMU Dihydrolipoamide acetyltransferase	8
4.43348004	4.413774	E9B3E6	tr|E9B3E6|E9B3E6_LEIMU 3-hydroxyisobutyryl-CoA hydrolase,	2
5.321301974	4.410081	E9AWI3	tr|E9AWI3|E9AWI3_LEIMU V-type proton ATPase subunit a OS	5
4.437039655	4.409282	E9APC4	tr|E9APC4|E9APC4_LEIMU Uncharacterized protein OS=Leishm	2
4.908251754	4.409128	E9B0H0	tr|E9B0H0|E9B0H0_LEIMU Eukaryotic translation initiation fact	3
4.386186739	4.408287	Q867J9;E9A	tr|Q867J9|Q867J9_LEIME Histone H2A OS=Leishmania mexicar	3
4.329887203	4.408197	E9AXQ9	tr|E9AXQ9|E9AXQ9_LEIMU Putative 3-oxo-5-alpha-steroid 4-d	6
4.395339362	4.406196	E9AX71	tr|E9AX71|E9AX71_LEIMU Putative fatty-acid desaturase OS=L	5
3.954451377	4.405593	E9B3T7	tr|E9B3T7|E9B3T7_LEIMU Uncharacterized protein OS=Leishm	3
4.573339046	4.405397	E9B4N8	tr|E9B4N8|E9B4N8_LEIMU Uncharacterized protein OS=Leishm	8
4.445576161	4.399104	E9AP80;E9A	tr|E9AP80|E9AP80_LEIMU Putative 60S ribosomal protein L18	8
4.355783038	4.398801	E9B350;E9B	tr|E9B350|E9B350_LEIMU Putative ribosomal protein L27 OS=I	10
5.233413193	4.396739	E9B6S3;A0A	tr|E9B6S3|E9B6S3_LEIMU Putative PRP8 protein homologue (L	5
4.036166657	4.396021	E9AXF3	tr|E9AXF3|E9AXF3_LEIMU Uncharacterized protein OS=Leishm	10
4.590765911	4.395027	E9ANI1	tr|E9ANI1|E9ANI1_LEIMU Putative 14-3-3 protein OS=Leishmar	5
4.642486646	4.393151	E9AT08	tr|E9AT08|E9AT08_LEIMU Uncharacterized protein OS=Leishm	3
4.610022635	4.392486	E9AZM9	tr|E9AZM9|E9AZM9_LEIMU Uncharacterized protein OS=Leish	2
3.558616049	4.390036	E9AJZ6	tr|E9AJZ6|E9AJZ6_LEIMU Putative exosome complex exonucle	3
4.464900349	4.389818	Q7Z031;E9A	tr|Q7Z031|Q7Z031_LEIAM Acidocalcisomal pyrophosphatase C	4
4.354049154	4.389596	E9AYU0	tr|E9AYU0|E9AYU0_LEIMU Putative nucleoporin OS=Leishman	8
4.226048175	4.387573	E9AW91;Q5	tr|E9AW91|E9AW91_LEIMU Putative mitochondrial RNA bindi	6
4.15706624	4.386764	E9B1G2	tr|E9B1G2|E9B1G2_LEIMU Putative ubiquitin hydrolase OS=Lei	2
5.129683093	4.386232	E9AWK0	tr|E9AWK0|E9AWK0_LEIMU Uncharacterized protein OS=Leish	3
4.479386682	4.385478	E9ART6	tr|E9ART6|E9ART6_LEIMU Plasma membrane ATPase OS=Leish	15
4.738453993	4.383259	E9AW42	tr|E9AW42|E9AW42_LEIMU ABC transporter-like protein OS=L	10
4.462875219	4.382646	Q95PT4;E9A	tr|Q95PT4|Q95PT4_LEIME Myo-inositol-1-phosphate synthase	8
4.323318588	4.3821	E9AKR8	tr|E9AKR8|E9AKR8_LEIMU Putative 60S ribosomal protein L19	12
4.664174407	4.380975	E9ANF6	tr|E9ANF6|E9ANF6_LEIMU Putative seryl-tRNA synthetase OS=	10
4.411762896	4.377092	E9B423	tr|E9B423|E9B423_LEIMU Nucleolar GTP-binding protein 1 OS=	11
4.102575524	4.375415	E9AQD9	tr|E9AQD9|E9AQD9_LEIMU Putative glutaminyl-tRNA syntheta	5

4.504543927	4.374321	E9AK89	tr|E9AK89|E9AK89_LEIMU Uncharacterized protein OS=Leishm	5
4.490340816	4.373672	E9ARS2	tr|E9ARS2|E9ARS2_LEIMU Putative pyruvate dehydrogenase E	6
4.257526325	4.373346	E9AKI1	tr|E9AKI1|E9AKI1_LEIMU Uncharacterized protein OS=Leishma	3
4.601544834	4.372769	E9AWN5;E9	tr|E9AWN5|E9AWN5_LEIMU Uncharacterized protein LMXM_2	7
4.581204461	4.372341	E9AMI0	tr|E9AMI0|E9AMI0_LEIMU Uncharacterized protein OS=Leishm	15
3.47317929	4.371387	E9ALY1	tr|E9ALY1|E9ALY1_LEIMU Uncharacterized protein OS=Leishm	2
4.790972707	4.370804	E9AKE1;Q0(	tr|E9AKE1|E9AKE1_LEIMU Trypanothione reductase OS=Leishm	10
4.756191316	4.369925	E9ARW2	tr|E9ARW2|E9ARW2_LEIMU Clathrin light chain OS=Leishmani	2
4.704201863	4.3698	E9B0L7	tr|E9B0L7|E9B0L7_LEIMU Putative adenosine kinase OS=Leishr	8
4.397110695	4.367035	E9AQJ6;O76	tr|E9AQJ6|E9AQJ6_LEIMU Uncharacterized protein OS=Leishm	6
4.95980164	4.366692	E9AZ99	tr|E9AZ99|E9AZ99_LEIMU Uncharacterized protein OS=Leishm	6
4.182639455	4.364806	E9AZH5	tr|E9AZH5|E9AZH5_LEIMU Putative pantothenate kinase subu	4
4.217947897	4.363154	E9AJQ8	tr|E9AJQ8|E9AJQ8_LEIMU Putative long chain fatty Acyl CoA sy	2
5.521174702	4.362964	E8NHS4;E8N	tr|E8NHS4|E8NHS4_LEIMU WGS CADB00000000 data, contig 9	3
4.519489386	4.361781	E9AQI0	tr|E9AQI0|E9AQI0_LEIMU Protein tyrosine phosphatase-like pr	5
4.50432575	4.36067	E9ARD0;I3V	tr|E9ARD0|E9ARD0_LEIMU Elongation factor 1-alpha OS=Leish	26
4.540139387	4.359393	E9ATM4	tr|E9ATM4|E9ATM4_LEIMU ATP-dependent RNA helicase-like	5
4.746585379	4.357753	E9AT19	tr|E9AT19|E9AT19_LEIMU Dipeptidyl-peptidase 8-like serine pe	6
3.867693331	4.357639	E9AVK1	tr|E9AVK1|E9AVK1_LEIMU Uncharacterized protein OS=Leishm	4
4.396545734	4.356114	E9B3H7	tr|E9B3H7|E9B3H7_LEIMU Putative GIPL galf transferase OS=Le	16
4.559171643	4.354874	E9B6L0	tr|E9B6L0|E9B6L0_LEIMU Uncharacterized protein OS=Leishma	7
4.411447405	4.354275	E9APW3;Q3	tr|E9APW3|E9APW3_LEIMU Enolase OS=Leishmania mexicana	23
4.362278028	4.354148	E9AYA1	tr|E9AYA1|E9AYA1_LEIMU Uncharacterized protein OS=Leishm	2
3.7358792	4.35295	E9AQP2	tr|E9AQP2|E9AQP2_LEIMU Uncharacterized protein OS=Leishm	2
4.490700831	4.350309	E9AXG9	tr|E9AXG9|E9AXG9_LEIMU Peptidyl-prolyl cis-trans isomerase	12
4.396666306	4.349349	E9AMD5	tr|E9AMD5|E9AMD5_LEIMU Translation initiation factor-like p	4
4.259192292	4.348836	E9ATF9	tr|E9ATF9|E9ATF9_LEIMU Nascent polypeptide-associated con	7
4.869749801	4.346368	E9AN80	tr|E9AN80|E9AN80_LEIMU Uncharacterized protein OS=Leishm	4
4.447764951	4.345979	E8NHI0	tr|E8NHI0|E8NHI0_LEIMU 40S ribosomal protein S3a OS=Leish	18
4.860186037	4.34306	E9AJV2	tr|E9AJV2|E9AJV2_LEIMU Uncharacterized protein OS=Leishma	13
4.704151769	4.342545	P16126;E9A	sp|P16126|DRTS_LEIAM Bifunctional dihydrofolate reductase-t	4
4.987380783	4.340873	E9B703	tr|E9B703|E9B703_LEIMU Glyceraldehyde-3-phosphate dehyd	3
4.183098278	4.340106	E9AST5	tr|E9AST5|E9AST5_LEIMU Putative poly-zinc finger protein 2 O	6
4.284327189	4.339221	E9AUU5	tr|E9AUU5|E9AUU5_LEIMU Phosphatase-like protein OS=Leish	4
5.042065795	4.334729	E9ALW2	tr|E9ALW2|E9ALW2_LEIMU Uncharacterized protein OS=Leishm	2
4.471304047	4.333689	E9ARB0	tr|E9ARB0|E9ARB0_LEIMU Protein SDA1 OS=Leishmania mexic	9
4.537596603	4.330687	E9B2C5	tr|E9B2C5|E9B2C5_LEIMU Uncharacterized protein OS=Leishm	5
4.22895905	4.329699	Q9GU60;E9	tr|Q9GU60|Q9GU60_LEIAM Kinetoplast membrane protein 11	9
4.848173638	4.328427	E9ANA1	tr|E9ANA1|E9ANA1_LEIMU Putative small GTP-binding protein	4
4.90848489	4.327483	E9AM73	tr|E9AM73|E9AM73_LEIMU Putative tryptophanyl-tRNA synth	4
4.677901997	4.326282	E9B3B0	tr|E9B3B0|E9B3B0_LEIMU Putative chaperonin alpha subunit (	12
4.519917089	4.32547	E9APU4	tr|E9APU4|E9APU4_LEIMU ADP/ATP mitochondrial carrier-like	5
4.664736605	4.323398	E9AVY2	tr|E9AVY2|E9AVY2_LEIMU Putative ATP-dependent DEAD/H RI	5
4.559169239	4.322961	E9B5F8	tr|E9B5F8|E9B5F8_LEIMU Vacuolar ATP synthase catalytic subu	9
5.125972865	4.321648	E9B0G8	tr|E9B0G8|E9B0G8_LEIMU Sulfhydryl oxidase OS=Leishmania r	2
4.243483652	4.318453	E9AV59	tr|E9AV59|E9AV59_LEIMU Xanthine phosphoribosyltransferase	4
4.498426211	4.31819	E9AX84	tr|E9AX84|E9AX84_LEIMU Putative poly(A)-binding protein OS	18
4.301181497	4.317601	E9AKJ5	tr|E9AKJ5|E9AKJ5_LEIMU Uncharacterized protein OS=Leishma	7
4.530329356	4.316114	E8NHJ5;E9A	tr|E8NHJ5|E8NHJ5_LEIMU WGS CADB00000000 data, contig 43	9
3.721408382	4.314204	Q9GNZ4;F6I	tr|Q9GNZ4|Q9GNZ4_LEIME 3-nucleotidase/nuclease OS=Leish	2
4.398056939	4.313627	E9B4G0	tr|E9B4G0|E9B4G0_LEIMU Coatomer subunit beta OS=Leishma	2
4.49633777	4.3135	E9B5S0	tr|E9B5S0|E9B5S0_LEIMU Putative serine palmitoyltransferase	2

5.048554478	4.312972	E9B506;A7Y	tr|E9B506|E9B506_LEIMU Uncharacterized protein OS=Leishm	3
4.697625752	4.312806	E9ARS1	tr|E9ARS1|E9ARS1_LEIMU Putative heat shock protein OS=Leis	39
4.355166098	4.308777	E9AME1	tr|E9AME1|E9AME1_LEIMU Uncharacterized protein OS=Leish	4
4.426697665	4.306828	E9AV89	tr|E9AV89|E9AV89_LEIMU Putative 60S ribosomal protein L9 C	12
4.462225928	4.305661	E9AS06	tr|E9AS06|E9AS06_LEIMU Methionine aminopeptidase OS=Lei	4
4.548188887	4.305485	E9AQB1	tr|E9AQB1|E9AQB1_LEIMU 60S acidic ribosomal protein P2 OS	7
3.951471654	4.305338	E9B1E9	tr|E9B1E9|E9B1E9_LEIMU Putative 5-methyltetrahydropteroyl	5
4.744880868	4.304094	E9B699	tr|E9B699|E9B699_LEIMU Putative RNA-binding protein OS=Le	18
4.479322123	4.303351	E9AQA0	tr|E9AQA0|E9AQA0_LEIMU NAD-specific glutamate dehydroge	33
4.022114486	4.30165	E9APM1	tr|E9APM1|E9APM1_LEIMU Uncharacterized protein OS=Leish	2
4.64127	4.299084	E9B405;A0A	tr|E9B405|E9B405_LEIMU Thiamine biosynthesis-like protein C	3
4.894086636	4.298455	E9ALL7	tr|E9ALL7|E9ALL7_LEIMU Uncharacterized protein OS=Leishma	3
5.390603815	4.297575	E9B0Q0	tr|E9B0Q0|E9B0Q0_LEIMU Small nuclear ribonucleoprotein E C	3
4.603582889	4.297204	E9AT09	tr|E9AT09|E9AT09_LEIMU Glucokinase 1-like protein OS=Leish	2
4.94918191	4.29398	E9ARY2	tr|E9ARY2|E9ARY2_LEIMU RNA binding protein OS=Leishmania	2
4.646337104	4.293507	E9AYD6	tr|E9AYD6|E9AYD6_LEIMU Putative thimet oligopeptidase (Me	9
4.137183131	4.292901	E9AR41	tr|E9AR41|E9AR41_LEIMU Uncharacterized protein OS=Leishm	2
4.861618568	4.291593	E9B1E7	tr|E9B1E7|E9B1E7_LEIMU Putative 60S acidic ribosomal protei	5
4.946099205	4.288986	E9B078	tr|E9B078|E9B078_LEIMU Putative 40S ribosomal protein S17	12
4.454958327	4.286884	E9AWK5	tr|E9AWK5|E9AWK5_LEIMU Uncharacterized protein OS=Leish	3
4.708632173	4.286208	E9AWR8	tr|E9AWR8|E9AWR8_LEIMU Putative malic enzyme OS=Leishm	10
4.678798314	4.284525	E9ATT5;E9A	tr|E9ATT5|E9ATT5_LEIMU 40S ribosomal protein SA OS=Leishm	13
5.738550753	4.283511	E9B081	tr|E9B081|E9B081_LEIMU Putative splicing factor 3B subunit 1	2
6.180479657	4.28234	E9AZH7	tr|E9AZH7|E9AZH7_LEIMU Putative RNA binding protein OS=Le	2
4.075531433	4.282051	E9ARN1	tr|E9ARN1|E9ARN1_LEIMU Uncharacterized protein OS=Leishr	3
4.242595703	4.281824	E9AZF7	tr|E9AZF7|E9AZF7_LEIMU Uncharacterized protein OS=Leishm	4
4.452038646	4.279998	E9ARI2	tr|E9ARI2|E9ARI2_LEIMU Carboxypeptidase OS=Leishmania me	5
4.085719358	4.279871	E9AQQ7	tr|E9AQQ7|E9AQQ7_LEIMU Uncharacterized protein OS=Leish	2
4.633389742	4.27908	E9ARD9	tr|E9ARD9|E9ARD9_LEIMU Diphosphomevalonate decarboxyla	3
4.327339677	4.276823	E9ATU9	tr|E9ATU9|E9ATU9_LEIMU Uncharacterized protein OS=Leishm	12
4.425858588	4.275005	E9B5F7	tr|E9B5F7|E9B5F7_LEIMU Transmembrane 9 superfamily mem	3
4.51372415	4.273684	E9AUG4	tr|E9AUG4|E9AUG4_LEIMU Uncharacterized protein OS=Leishi	2
3.793061761	4.272842	E9AYF0	tr|E9AYF0|E9AYF0_LEIMU Putative cytochrome c oxidase subu	3
4.66828532	4.271143	E9ALG1	tr|E9ALG1|E9ALG1_LEIMU Uncharacterized protein OS=Leishm	6
5.146588409	4.269538	E9AXI5	tr|E9AXI5|E9AXI5_LEIMU Uncharacterized protein OS=Leishma	3
4.840803368	4.267678	E9AV28	tr|E9AV28|E9AV28_LEIMU Putative RNA helicase OS=Leishmar	4
4.889638864	4.264493	E9ANJ3	tr|E9ANJ3|E9ANJ3_LEIMU Putative pumilio-repeat, RNA-bindir	8
4.850048718	4.264327	E9ALY6;Q9U	tr|E9ALY6|E9ALY6_LEIMU Putative ADP ribosylation factor 3 OS	11
5.51985851	4.263415	E9AUN0	tr|E9AUN0|E9AUN0_LEIMU Uncharacterized protein OS=Leishi	2
4.631222783	4.261697	Q8MPM3;E9	tr|Q8MPM3|Q8MPM3_LEIME Transketolase OS=Leishmania m	8
4.159841845	4.26096	E9AQC4	tr|E9AQC4|E9AQC4_LEIMU Uncharacterized protein OS=Leishr	2
4.436902133	4.260145	E9B6R4	tr|E9B6R4|E9B6R4_LEIMU T-complex protein 1 subunit eta OS=	19
4.294343718	4.259554	E9B552	tr|E9B552|E9B552_LEIMU RuvB-like helicase OS=Leishmania m	9
4.589408617	4.253597	E9ASK9	tr|E9ASK9|E9ASK9_LEIMU Eukaryotic translation initiation fact	2
5.084352871	4.251351	E9ATX3	tr|E9ATX3|E9ATX3_LEIMU Putative NADH dehydrogenase OS=I	9
4.384045209	4.249657	E9ALW5;E9A	tr|E9ALW5|E9ALW5_LEIMU Putative ribosomal protein L1a OS	14
4.064653837	4.246875	E9B5I1	tr|E9B5I1|E9B5I1_LEIMU Putative dynein heavy chain OS=Leish	5
4.797955809	4.246707	E9AQL1;Q9I	tr|E9AQL1|E9AQL1_LEIMU Dihydroorotate dehydrogenase (fur	3
4.609125153	4.246606	E9AKT6	tr|E9AKT6|E9AKT6_LEIMU Putative 60S ribosomal protein L23a	9
4.803899422	4.246218	E9AXE9	tr|E9AXE9|E9AXE9_LEIMU Uncharacterized protein OS=Leishm	5
4.81475679	4.24403	E9ATH0	tr|E9ATH0|E9ATH0_LEIMU Eukaryotic translation initiation fact	7
5.076164907	4.243891	E9AT43	tr|E9AT43|E9AT43_LEIMU Dihydrolipoamide acetyltransferase	6

4.770197002	4.243636	E9B632	tr|E9B632|E9B632_LEIMU Putative reiske iron-sulfur protein O	3
4.222213114	4.243266	E9AT78	tr|E9AT78|E9AT78_LEIMU Putative ATP-dependent RNA helica	4
4.591758333	4.241166	E9ALV7	tr|E9ALV7|E9ALV7_LEIMU Tryparedoxin OS=Leishmania mexica	6
5.463667758	4.240902	E9AMI9	tr|E9AMI9|E9AMI9_LEIMU Uncharacterized protein OS=Leishm	3
4.574043615	4.237132	E9APJ0	tr|E9APJ0|E9APJ0_LEIMU Putative chaperonin TCP20 OS=Leish	12
4.609232289	4.236643	E9B6K2	tr|E9B6K2|E9B6K2_LEIMU Putative cystathione gamma lyase C	14
4.150937053	4.236375	E9AU90	tr|E9AU90|E9AU90_LEIMU Similarity to endo-1-like protein OS	5
6.399349307	4.236069	Q8T381;E9A	tr|Q8T381|Q8T381_LEIME Mercaptopyruvate sulfurtransferase	4
4.545842777	4.23214	E9AN39	tr|E9AN39|E9AN39_LEIMU Isocitrate dehydrogenase [NADP] C	15
4.751328746	4.230022	E9AVE1	tr|E9AVE1|E9AVE1_LEIMU Putative RNA helicase OS=Leishmar	11
4.322058745	4.229974	E9AU26	tr|E9AU26|E9AU26_LEIMU Uncharacterized protein OS=Leishn	2
4.384850476	4.22939	E9B6C6	tr|E9B6C6|E9B6C6_LEIMU Uncharacterized protein OS=Leishm	4
4.882179344	4.228798	E9APB2	tr|E9APB2|E9APB2_LEIMU Uncharacterized protein OS=Leishm	16
4.605129086	4.227731	E9B483	tr|E9B483|E9B483_LEIMU Uncharacterized protein OS=Leishm	16
4.773992447	4.227601	E9ASB9	tr|E9ASB9|E9ASB9_LEIMU Histone H4 OS=Leishmania mexican	4
4.065392894	4.225651	E9APU2	tr|E9APU2|E9APU2_LEIMU Uncharacterized protein OS=Leishn	2
4.366821935	4.225566	E9AXI8	tr|E9AXI8|E9AXI8_LEIMU Uncharacterized protein OS=Leishma	3
4.689965553	4.223897	E9AT73	tr|E9AT73|E9AT73_LEIMU Succinate--CoA ligase [ADP-forming]	12
4.610497702	4.22219	E9B478	tr|E9B478|E9B478_LEIMU Putative heat shock protein OS=Leis	27
4.683378702	4.221973	E9AT13	tr|E9AT13|E9AT13_LEIMU Putative tyrosine aminotransferase	7
4.569379371	4.221841	E9B5D9	tr|E9B5D9|E9B5D9_LEIMU RuvB-like helicase OS=Leishmania n	4
4.485136971	4.218553	E9AZQ5	tr|E9AZQ5|E9AZQ5_LEIMU Ribonucleoside-diphosphate reduct	4
4.357822232	4.216757	E9ANI6	tr|E9ANI6|E9ANI6_LEIMU Tubulin-tyrsoine ligase-like protein C	3
4.5128421	4.215613	E9AWJ0	tr|E9AWJ0|E9AWJ0_LEIMU Uncharacterized protein OS=Leishr	3
5.334225101	4.214588	E9B264	tr|E9B264|E9B264_LEIMU Acetoin dehydrogenase e3 compone	2
4.468584966	4.213154	E9B2I6	tr|E9B2I6|E9B2I6_LEIMU Uncharacterized protein OS=Leishma	3
4.204896365	4.212838	E9AVH3	tr|E9AVH3|E9AVH3_LEIMU Putative ATP synthase F1 subunit g	6
4.957346667	4.210697	E9B010	tr|E9B010|E9B010_LEIMU Uncharacterized protein OS=Leishm	3
4.841634225	4.208288	E9AJU1	tr|E9AJU1|E9AJU1_LEIMU Uncharacterized protein OS=Leishm	3
4.197599405	4.204196	E9B1S5	tr|E9B1S5|E9B1S5_LEIMU p-glycoprotein e OS=Leishmania me	2
4.784723945	4.201804	E9B494	tr|E9B494|E9B494_LEIMU Isocitrate dehydrogenase [NADP] OS	3
4.940194165	4.200603	E9ANP4	tr|E9ANP4|E9ANP4_LEIMU Pyruvate, phosphate dikinase OS=L	11
4.566433527	4.200523	E9AS22	tr|E9AS22|E9AS22_LEIMU Putative kinesin OS=Leishmania mex	6
4.720486523	4.199375	E9AVE7	tr|E9AVE7|E9AVE7_LEIMU Uncharacterized protein OS=Leishm	4
4.71162467	4.198147	E9ARC2	tr|E9ARC2|E9ARC2_LEIMU Uncharacterized protein OS=Leishm	20
4.495476032	4.19565	E9AZD6	tr|E9AZD6|E9AZD6_LEIMU Transcription elongation regulator-l	6
4.900405017	4.193182	E9AZK8	tr|E9AZK8|E9AZK8_LEIMU Carbonic anhydrase-like protein OS=	5
5.014130687	4.192996	E9AU73	tr|E9AU73|E9AU73_LEIMU Uncharacterized protein OS=Leishn	3
5.164678417	4.192703	E9ARY5	tr|E9ARY5|E9ARY5_LEIMU Uncharacterized protein OS=Leishm	3
4.583079286	4.191442	E9ANU8	tr|E9ANU8|E9ANU8_LEIMU Proteasome regulatory ATPase sub	5
5.149845107	4.190928	E9AXR6	tr|E9AXR6|E9AXR6_LEIMU Uncharacterized protein OS=Leishm	3
4.013362955	4.190516	Q4TZP8;E9E	tr|Q4TZP8|Q4TZP8_LEIME Putative mitogen-activated protein	2
4.479120307	4.18767	E9ASE5	tr|E9ASE5|E9ASE5_LEIMU Uncharacterized protein OS=Leishm	2
4.265635568	4.187265	E9AYV3	tr|E9AYV3|E9AYV3_LEIMU Putative radial spoke protein 3 OS=l	3
5.192226095	4.18491	E9B5D3	tr|E9B5D3|E9B5D3_LEIMU DNA topoisomerase I OS=Leishman	9
4.258245013	4.183767	E9ANC5	tr|E9ANC5|E9ANC5_LEIMU Uncharacterized protein OS=Leishn	5
4.728116878	4.18358	E9B660	tr|E9B660|E9B660_LEIMU Uncharacterized protein OS=Leishm	3
4.623940834	4.183223	E9AJC9	tr|E9AJC9|E9AJC9_LEIMU 40S ribosomal protein S7 OS=Leishm	10
4.889839984	4.183153	E9B6I5	tr|E9B6I5|E9B6I5_LEIMU Putative ubiquitin-activating enzyme	6
4.601106259	4.183043	E9B2E8	tr|E9B2E8|E9B2E8_LEIMU Uncharacterized protein OS=Leishm	11
4.958085624	4.179914	E9AJU3	tr|E9AJU3|E9AJU3_LEIMU Putative arginine N-methyltransfera	4
5.110737029	4.178759	E9AKQ4	tr|E9AKQ4|E9AKQ4_LEIMU Putative 5-3 exonuclease OS=Leish	8

5.268938879	4.178293	E9B4X7	tr|E9B4X7|E9B4X7_LEIMU Uncharacterized protein OS=Leishm	3
3.868245113	4.178242	E9ATM9	tr|E9ATM9|E9ATM9_LEIMU Putative mitochondrial intermedia	6
4.929500431	4.177617	E9AW59	tr|E9AW59|E9AW59_LEIMU Putative 5-3 exonuclease XRNC OS	2
4.241790485	4.175285	E9B340	tr|E9B340|E9B340_LEIMU Uncharacterized protein OS=Leishm	2
4.375912606	4.17481	E9ARA1	tr|E9ARA1|E9ARA1_LEIMU Putative translation initiation factor	11
5.430603791	4.173894	E9ARJ6	tr|E9ARJ6|E9ARJ6_LEIMU Putative RNA binding protein OS=Lei	2
4.763572592	4.173634	Q4KXA7;E9A	tr|Q4KXA7|Q4KXA7_LEIME Cytoplasmic dynein heavy chain 2.2	5
4.672916637	4.173228	E9AJH6	tr|E9AJH6|E9AJH6_LEIMU Uncharacterized protein OS=Leishm	2
4.766688537	4.172762	E9AW40	tr|E9AW40|E9AW40_LEIMU Putative NADP-dependent alcoho	12
4.670977183	4.172743	E9AXH6	tr|E9AXH6|E9AXH6_LEIMU Putative dynein heavy chain OS=Lei	16
4.60341456	4.168922	E9AV90;Q8	tr|E9AV90|E9AV90_LEIMU Putative 40S ribosomal protein S23	4
3.912037069	4.167286	E9ALT4	tr|E9ALT4|E9ALT4_LEIMU Uncharacterized protein OS=Leishm	2
4.087461166	4.166427	E9AQC1	tr|E9AQC1|E9AQC1_LEIMU DNA topoisomerase 2 OS=Leishma	2
4.59362843	4.165991	E9AZR2	tr|E9AZR2|E9AZR2_LEIMU 40S ribosomal protein S14 OS=Leish	5
4.085573765	4.164075	E9B1T9	tr|E9B1T9|E9B1T9_LEIMU Uncharacterized protein OS=Leishm	2
4.621615685	4.163196	E9AMH0	tr|E9AMH0|E9AMH0_LEIMU Uncharacterized protein OS=Leish	6
5.730657611	4.160995	E9AYX1	tr|E9AYX1|E9AYX1_LEIMU Uncharacterized protein OS=Leishm	5
4.579470346	4.160739	E9B1D4	tr|E9B1D4|E9B1D4_LEIMU Putative ATP synthase, epsilon chai	4
4.947148921	4.160733	E9AW86	tr|E9AW86|E9AW86_LEIMU Acetyl-coenzyme A synthetase OS	8
4.642216551	4.160393	E9ANH0	tr|E9ANH0|E9ANH0_LEIMU Proteasome subunit alpha type OS	2
3.530460357	4.160236	E9AV30	tr|E9AV30|E9AV30_LEIMU Putative phosphoglucomutase OS=L	2
5.088818504	4.1593	E9B5N7	tr|E9B5N7|E9B5N7_LEIMU Uncharacterized protein OS=Leishm	3
4.838608552	4.1576	O46368;E9E	tr|O46368|O46368_LEIME Surface protein amastin OS=Leishm	2
4.63362482	4.15553	E9AQU2;A0	tr|E9AQU2|E9AQU2_LEIMU Putative cytochrome c OS=Leishma	3
4.826370937	4.154951	E9AJS7;E9A	tr|E9AJS7|E9AJS7_LEIMU Putative 60S acidic ribosomal protein	4
5.34596858	4.153853	E9AM90	tr|E9AM90|E9AM90_LEIMU Uncharacterized protein OS=Leish	5
5.792417834	4.153403	E9B601	tr|E9B601|E9B601_LEIMU Putative short chain dehydrogenase	2
4.770953492	4.151824	E9AUZ2;E9A	tr|E9AUZ2|E9AUZ2_LEIMU Phosphotransferase OS=Leishmania	30
4.752166845	4.151677	E9AQL8;Q9I	tr|E9AQL8|E9AQL8_LEIMU Putative carbamoyl-phosphate synt	32
4.954872689	4.151448	E9AKI8	tr|E9AKI8|E9AKI8_LEIMU Uncharacterized protein OS=Leishma	3
5.063067918	4.149425	E9B2F7	tr|E9B2F7|E9B2F7_LEIMU Uncharacterized protein OS=Leishm	7
5.052378632	4.147141	E9AZQ9	tr|E9AZQ9|E9AZQ9_LEIMU Uncharacterized protein OS=Leishm	5
5.143430404	4.147026	E9B714	tr|E9B714|E9B714_LEIMU Uncharacterized protein OS=Leishm	6
4.693668294	4.146379	E9B545	tr|E9B545|E9B545_LEIMU Uncharacterized protein OS=Leishm	11
4.376804373	4.144608	E9B258	tr|E9B258|E9B258_LEIMU Uncharacterized protein OS=Leishm	3
4.094374699	4.143764	E9AKF2	tr|E9AKF2|E9AKF2_LEIMU Nucleolar GTP-binding protein 2 OS=	5
3.849283536	4.143093	E9B4I7	tr|E9B4I7|E9B4I7_LEIMU Uncharacterized protein OS=Leishma	3
4.135917595	4.139071	E9B012	tr|E9B012|E9B012_LEIMU Putative zinc transporter OS=Leishm	3
4.631211586	4.138833	E9AP13	tr|E9AP13|E9AP13_LEIMU NADH:flavin oxidoreductase/NADH	10
4.676725059	4.138083	E9AM47	tr|E9AM47|E9AM47_LEIMU Uncharacterized protein OS=Leish	4
5.397404525	4.136553	Q0QW09;E9	tr|Q0QW09|Q0QW09_LEIME Malate dehydrogenase OS=Leish	2
4.875064979	4.134963	E9AXN4	tr|E9AXN4|E9AXN4_LEIMU Uncharacterized protein OS=Leishm	8
4.778892058	4.131978	E9B2G6;E9A	tr|E9B2G6|E9B2G6_LEIMU 40S ribosomal protein S2 OS=Leishm	11
4.506185669	4.13121	E9B6N3	tr|E9B6N3|E9B6N3_LEIMU Uncharacterized protein OS=Leishm	2
4.792035678	4.130556	E9APT6	tr|E9APT6|E9APT6_LEIMU Glutathione synthetase OS=Leishma	2
4.458633111	4.129048	E9ARI8	tr|E9ARI8|E9ARI8_LEIMU Aconitate hydratase OS=Leishmania	18
5.148050182	4.128405	E9AKU4	tr|E9AKU4|E9AKU4_LEIMU Putative lanosterol synthase OS=Le	3
4.079573116	4.128014	E9B6Q0	tr|E9B6Q0|E9B6Q0_LEIMU Uncharacterized protein OS=Leishm	6
5.577650589	4.127724	E9B0Q4;A0	tr|E9B0Q4|E9B0Q4_LEIMU Putative Pyridoxal kinase OS=Leishm	3
4.779969786	4.125448	E9AS23	tr|E9AS23|E9AS23_LEIMU Glycosomal malate dehydrogenase	15
5.147499882	4.12399	E9AXK5	tr|E9AXK5|E9AXK5_LEIMU Uncharacterized protein OS=Leishm	2
4.847596855	4.123145	E9B1E2	tr|E9B1E2|E9B1E2_LEIMU Uncharacterized protein OS=Leishm	14

4.60243871	4.120336	E9ATG7	tr|E9ATG7|E9ATG7_LEIMU Uncharacterized protein OS=Leishm	2
4.714828547	4.119899	E9B0D0	tr|E9B0D0|E9B0D0_LEIMU Uncharacterized protein OS=Leishm	3
4.983080045	4.119805	E9ARJ0	tr|E9ARJ0|E9ARJ0_LEIMU Uncharacterized protein OS=Leishma	3
4.733373566	4.118867	E9AUK8	tr|E9AUK8|E9AUK8_LEIMU rRNA biogenesis protein-like protei	4
3.754551528	4.118788	E9AJX4	tr|E9AJX4|E9AJX4_LEIMU Putative peter pan protein OS=Leishi	2
4.513012572	4.117922	E9ARW7	tr|E9ARW7|E9ARW7_LEIMU Putative aminopeptidase OS=Leisl	11
4.437827898	4.116673	E9B2N1	tr|E9B2N1|E9B2N1_LEIMU Putative dynein OS=Leishmania me	6
4.11351191	4.115729	E9AL20	tr|E9AL20|E9AL20_LEIMU Pitrilysin-like metalloprotease (Meta	2
5.627006233	4.112806	E9AUT3	tr|E9AUT3|E9AUT3_LEIMU Uncharacterized protein OS=Leishm	3
5.019007475	4.111107	E9AS17	tr|E9AS17|E9AS17_LEIMU Uncharacterized protein OS=Leishm	2
4.967429829	4.110908	E9B1G7	tr|E9B1G7|E9B1G7_LEIMU Putative nucleolar protein OS=Leish	6
4.125815431	4.110104	E9AN53;E9/	tr|E9AN53|E9AN53_LEIMU GP63, leishmanolysin OS=Leishmar	2
4.309074076	4.108772	E9B1B4	tr|E9B1B4|E9B1B4_LEIMU Putative CAS/CSE/importin domain	2
4.721180441	4.108329	E9B0H1;E9/	tr|E9B0H1|E9B0H1_LEIMU Putative aspartyl-tRNA synthetase (	9
5.010246277	4.107738	E9B073	tr|E9B073|E9B073_LEIMU Putative acyl-CoA dehydrogenase O	7
3.823659175	4.104739	E9AMB3	tr|E9AMB3|E9AMB3_LEIMU Uncharacterized protein OS=Leish	2
4.488587835	4.104374	E9ASN7	tr|E9ASN7|E9ASN7_LEIMU Uncharacterized protein OS=Leishm	4
4.584872111	4.103311	E9ARW8	tr|E9ARW8|E9ARW8_LEIMU Uncharacterized protein OS=Leish	9
4.705661698	4.102575	E9AX57	tr|E9AX57|E9AX57_LEIMU Uncharacterized protein OS=Leishm	11
4.662430618	4.101624	Q86PL7;E9A	tr|Q86PL7|Q86PL7_LEIME Putative small GTP-binding protein (	2
4.826529485	4.101616	E9B6L6	tr|E9B6L6|E9B6L6_LEIMU Uncharacterized protein OS=Leishma	2
4.996882244	4.099674	E9AUS4	tr|E9AUS4|E9AUS4_LEIMU Coatomer subunit beta OS=Leishma	2
4.55508227	4.095538	E9ALY7	tr|E9ALY7|E9ALY7_LEIMU Uncharacterized protein OS=Leishma	4
4.880573929	4.095078	E9ALH3	tr|E9ALH3|E9ALH3_LEIMU ATP-dependent 6-phosphofructokir	11
4.474538299	4.094961	E9AZE5	tr|E9AZE5|E9AZE5_LEIMU Uncharacterized protein OS=Leishm	3
4.596706651	4.091661	E9B578;E9B	tr|E9B578|E9B578_LEIMU Putative ribosomal protein L3 OS=Le	12
4.757682577	4.089052	E9AZ29	tr|E9AZ29|E9AZ29_LEIMU Putative T-complex protein 1, beta s	17
4.823480139	4.087936	E9AVT0	tr|E9AVT0|E9AVT0_LEIMU Uncharacterized protein OS=Leishm	2
4.653035713	4.080778	E9AK58	tr|E9AK58|E9AK58_LEIMU Putative 60S ribosomal protein L10	14
4.33597353	4.079774	E9AVS5;E9A	tr|E9AVS5|E9AVS5_LEIMU Uncharacterized protein OS=Leishm	2
4.34060589	4.079068	E9AMQ3	tr|E9AMQ3|E9AMQ3_LEIMU Uncharacterized protein OS=Leisl	5
4.609432841	4.078252	E9ASQ3	tr|E9ASQ3|E9ASQ3_LEIMU Putative N-acetyltransferase subun	9
4.629683072	4.076005	E9ANF3	tr|E9ANF3|E9ANF3_LEIMU Uncharacterized protein OS=Leishm	9
4.547327886	4.075115	Q2TUW2;E9	tr|Q2TUW2|Q2TUW2_LEIAM Inosine-5-monophosphate dehyc	7
4.58581574	4.072902	E9AR70	tr|E9AR70|E9AR70_LEIMU Uncharacterized protein OS=Leishm	2
5.302752814	4.071939	E9APN6	tr|E9APN6|E9APN6_LEIMU Uncharacterized protein OS=Leishn	9
5.643211159	4.07001	E9B4B4	tr|E9B4B4|E9B4B4_LEIMU Putative translation initiation factor	6
4.737165428	4.069283	E9AX88	tr|E9AX88|E9AX88_LEIMU Putative electron transfer flavoprot	4
4.754751383	4.068411	E9B476	tr|E9B476|E9B476_LEIMU Uncharacterized protein OS=Leishm	2
5.077934046	4.066804	E9ARL9	tr|E9ARL9|E9ARL9_LEIMU Uncharacterized protein OS=Leishm	23
4.79843644	4.06634	E9ANJ4	tr|E9ANJ4|E9ANJ4_LEIMU Uncharacterized protein OS=Leishm	7
4.435344102	4.066075	E9AZU3	tr|E9AZU3|E9AZU3_LEIMU Putative p450 reductase OS=Leishm	2
6.168411861	4.064124	E9B584	tr|E9B584|E9B584_LEIMU Uncharacterized protein OS=Leishm	2
4.397198858	4.063594	E9AK29	tr|E9AK29|E9AK29_LEIMU 60S ribosomal protein L11 (L5, L16)	9
4.538472565	4.062717	E9AM28	tr|E9AM28|E9AM28_LEIMU Cofilin-like protein OS=Leishmania	6
4.659198674	4.062222	E9AJD9	tr|E9AJD9|E9AJD9_LEIMU Putative long-chain-fatty-acid-CoA li	10
4.635142102	4.061834	E9B155	tr|E9B155|E9B155_LEIMU Putative chaperonin HSP60/CNP60 (	5
4.061595636	4.061659	E9AMM1	tr|E9AMM1|E9AMM1_LEIMU Uncharacterized protein OS=Leis	3
4.106163422	4.061101	E9AQG1	tr|E9AQG1|E9AQG1_LEIMU Uncharacterized protein OS=Leishi	2
4.855069818	4.060434	E9AMJ4	tr|E9AMJ4|E9AMJ4_LEIMU Uncharacterized protein OS=Leishn	2
5.370916146	4.05913	E9B4W6;E9	tr|E9B4W6|E9B4W6_LEIMU D-isomer specific 2-hydroxyacid de	6
4.849934674	4.058836	E9B683	tr|E9B683|E9B683_LEIMU 60S ribosomal protein L32 OS=Leish	3

4.956578109	4.057016	E9AN73	tr|E9AN73|E9AN73_LEIMU Flagellar glycoprotein-like protein (	7
4.596482978	4.056966	E9AKH0	tr|E9AKH0|E9AKH0_LEIMU Uncharacterized protein OS=Leishn	2
4.75345573	4.052226	E9AT76	tr|E9AT76|E9AT76_LEIMU Uncharacterized protein OS=Leishm	2
4.387369073	4.051739	E9B2Z5	tr|E9B2Z5|E9B2Z5_LEIMU Uncharacterized protein OS=Leishm	8
6.09465134	4.050217	E9AVE4	tr|E9AVE4|E9AVE4_LEIMU Uncharacterized protein OS=Leishm	2
4.73370078	4.048598	E9AM37	tr|E9AM37|E9AM37_LEIMU Uncharacterized protein OS=Leish	2
4.983310632	4.048317	E9AVH9	tr|E9AVH9|E9AVH9_LEIMU Proteasome subunit alpha type OS	5
4.986786597	4.047498	E9B047	tr|E9B047|E9B047_LEIMU Putative glycosomal membrane prot	2
4.690703614	4.046009	E9AVH2	tr|E9AVH2|E9AVH2_LEIMU Centromere/microtubule binding p	9
5.197617017	4.045705	E9B2W2	tr|E9B2W2|E9B2W2_LEIMU Chaperonin HSP60, mitochondrial	2
5.163377702	4.045561	E9APD3	tr|E9APD3|E9APD3_LEIMU Putative proteasome regulatory AT	6
5.187505924	4.045263	E9APT8	tr|E9APT8|E9APT8_LEIMU Putative immunodominant antigen	7
4.871358184	4.045032	E9AW70	tr|E9AW70|E9AW70_LEIMU Putative ubiquitin-activating enzy	6
5.425162267	4.043655	E9AXZ5	tr|E9AXZ5|E9AXZ5_LEIMU Adenine phosphoribosyltransferase	2
4.890635239	4.038624	E9AVG6	tr|E9AVG6|E9AVG6_LEIMU Proteasome subunit alpha type OS	6
3.634691716	4.038263	E9ATD6	tr|E9ATD6|E9ATD6_LEIMU Putative signal recognition particle	2
4.933781743	4.036706	E9AYL9	tr|E9AYL9|E9AYL9_LEIMU Uncharacterized protein OS=Leishma	5
4.770553917	4.036265	E9AKY4;A7Y	tr|E9AKY4|E9AKY4_LEIMU Protein disulfide isomerase OS=Leis	3
4.889999034	4.03326	E9AZK7	tr|E9AZK7|E9AZK7_LEIMU Uncharacterized protein OS=Leishm	2
4.582392768	4.032574	E9AUN1	tr|E9AUN1|E9AUN1_LEIMU Uncharacterized protein OS=Leishi	20
4.838594684	4.03105	E9B013	tr|E9B013|E9B013_LEIMU Uncharacterized protein OS=Leishm	5
4.893698772	4.030637	E9B730	tr|E9B730|E9B730_LEIMU Polyadenylate-binding protein OS=L	17
4.712931047	4.027733	E9B1C6	tr|E9B1C6|E9B1C6_LEIMU S-adenosylmethionine synthase OS=	12
4.775850208	4.027638	E9B508	tr|E9B508|E9B508_LEIMU Uncharacterized protein OS=Leishm	2
4.957113337	4.027464	E9AUL9	tr|E9AUL9|E9AUL9_LEIMU Putative vesicle-fusing ATPase OS=L	5
4.40873186	4.025496	E9AQ80	tr|E9AQ80|E9AQ80_LEIMU Uncharacterized protein OS=Leishn	2
4.252820663	4.02401	E9ATN0	tr|E9ATN0|E9ATN0_LEIMU Uncharacterized protein OS=Leishn	4
3.92947531	4.023628	E9AZV0	tr|E9AZV0|E9AZV0_LEIMU Putative ATP-dependent RNA helica	2
4.676927208	4.023285	E9B2U4	tr|E9B2U4|E9B2U4_LEIMU Uncharacterized protein OS=Leishm	2
3.958377889	4.022379	E9AXB9	tr|E9AXB9|E9AXB9_LEIMU Uncharacterized protein OS=Leishm	2
4.400342763	4.020901	E9AYN0	tr|E9AYN0|E9AYN0_LEIMU Uncharacterized protein OS=Leishn	2
5.624422001	4.02031	E9ATB8	tr|E9ATB8|E9ATB8_LEIMU 60S ribosomal protein L29 OS=Leish	3
4.400857628	4.019273	E9AJN6	tr|E9AJN6|E9AJN6_LEIMU Putative peptidyl dipeptidase OS=Le	3
4.505179443	4.018773	E9AXH8	tr|E9AXH8|E9AXH8_LEIMU Uncharacterized protein OS=Leishn	3
4.709858785	4.018494	E9AP50	tr|E9AP50|E9AP50_LEIMU Uncharacterized protein OS=Leishm	4
5.307965919	4.017132	E9B3L1	tr|E9B3L1|E9B3L1_LEIMU Putative ATP-binding cassette protei	5
4.678499724	4.017121	E9ANG1	tr|E9ANG1|E9ANG1_LEIMU Uncharacterized protein OS=Leishi	8
4.70292899	4.014951	E9ANI7	tr|E9ANI7|E9ANI7_LEIMU Ribosome biogenesis protein BOP1 h	3
4.807780023	4.014874	E9AT85	tr|E9AT85|E9AT85_LEIMU Fibrillarin OS=Leishmania mexicana	8
4.670576458	4.014721	E9B193	tr|E9B193|E9B193_LEIMU 3-hydroxy-3-methylglutaryl coenzyn	5
4.755404785	4.007758	E9ARW6	tr|E9ARW6|E9ARW6_LEIMU Uncharacterized protein OS=Leish	7
4.889733773	4.005431	E9AQ10	tr|E9AQ10|E9AQ10_LEIMU Putative ATP-dependent RNA helic	4
5.102272722	4.004009	E9B174	tr|E9B174|E9B174_LEIMU Putative Lsm5p OS=Leishmania mex	2
4.601970638	4.002697	E9AS18	tr|E9AS18|E9AS18_LEIMU DNA-directed RNA polymerase, alph	5
4.497345129	4.001471	E9B4Q3;E9E	tr|E9B4Q3|E9B4Q3_LEIMU Elongation factor 1-beta OS=Leishn	6
4.802141554	3.997688	E9B199	tr|E9B199|E9B199_LEIMU Putative glutamyl-tRNA synthetase (	8
5.416753352	3.997546	E9B234	tr|E9B234|E9B234_LEIMU Putative p-nitrophenylphosphatase	3
4.493574839	3.996218	E9B187	tr|E9B187|E9B187_LEIMU Putative valyl-tRNA synthetase OS=L	5
4.86021483	3.992205	E9ARY4	tr|E9ARY4|E9ARY4_LEIMU Intraflagellar transport protein com	2
4.079459318	3.991401	E9APD5	tr|E9APD5|E9APD5_LEIMU Uncharacterized protein OS=Leishn	2
4.529864819	3.991051	E9ARQ8	tr|E9ARQ8|E9ARQ8_LEIMU Putative prolyl-tRNA synthetase OS	3
5.012026147	3.990458	E9APD4;A0/	tr|E9APD4|E9APD4_LEIMU Putative leucyl-tRNA synthetase OS	12

4.726764128	3.989556	E9AV01	tr|E9AV01|E9AV01_LEIMU Mitochondrial processing peptidase	9
5.626806735	3.987444	E9ANE2	tr|E9ANE2|E9ANE2_LEIMU Uncharacterized protein OS=Leishn	3
4.676479435	3.985146	E9AV48	tr|E9AV48|E9AV48_LEIMU Uncharacterized protein OS=Leishm	21
5.240623681	3.983334	E9B6L3;B0F	tr|E9B6L3|E9B6L3_LEIMU 6-phosphogluconate dehydrogenase	7
4.831746009	3.982151	E9ANZ0	tr|E9ANZ0|E9ANZ0_LEIMU Putative alanine aminotransferase (	4
4.350728718	3.982032	E9AMI2	tr|E9AMI2|E9AMI2_LEIMU Uncharacterized protein OS=Leishm	2
4.444115686	3.981233	E9AUS1	tr|E9AUS1|E9AUS1_LEIMU Putative calpain-like cysteine peptic	3
4.740689401	3.980772	E9AXT8	tr|E9AXT8|E9AXT8_LEIMU Uncharacterized protein OS=Leishm	2
5.027140901	3.98063	E9B2K7	tr|E9B2K7|E9B2K7_LEIMU Uncharacterized protein OS=Leishm	2
5.03790636	3.97914	E9AX44	tr|E9AX44|E9AX44_LEIMU Uncharacterized protein OS=Leishm	5
4.162154876	3.978292	E9AY81	tr|E9AY81|E9AY81_LEIMU Putative dynein heavy chain OS=Leis	4
4.182461419	3.978062	Q2PQD2;E9	tr|Q2PQD2|Q2PQD2_LEIME MPK10 OS=Leishmania mexicana (	2
4.228100185	3.975541	E9AN97;Q9I	tr|E9AN97|E9AN97_LEIMU Histone H3 OS=Leishmania mexicar	5
4.685315947	3.974966	E9ANL6	tr|E9ANL6|E9ANL6_LEIMU Uncharacterized protein OS=Leishm	2
4.465814737	3.973782	E9AU33	tr|E9AU33|E9AU33_LEIMU Uncharacterized protein OS=Leishn	4
4.061786278	3.97378	E9ATG0	tr|E9ATG0|E9ATG0_LEIMU Uncharacterized protein OS=Leishm	5
4.636283176	3.973271	E9APC3	tr|E9APC3|E9APC3_LEIMU Uncharacterized protein OS=Leishm	2
4.117002733	3.972381	E9B4I2	tr|E9B4I2|E9B4I2_LEIMU Putative malate dehydrogenase OS=L	2
4.652002349	3.969245	E9B4K6	tr|E9B4K6|E9B4K6_LEIMU Uncharacterized protein (Fragment)	3
5.786240359	3.967183	E9B0Q7	tr|E9B0Q7|E9B0Q7_LEIMU Uncharacterized protein OS=Leishn	2
4.605712883	3.966057	E9B700	tr|E9B700|E9B700_LEIMU Uncharacterized protein OS=Leishm	14
4.76005447	3.96283	E9AVR1	tr|E9AVR1|E9AVR1_LEIMU Putative NADH-cytochrome b5 redu	7
4.858317368	3.962476	E9B0G2	tr|E9B0G2|E9B0G2_LEIMU Putative phosphatase 2C OS=Leishr	3
4.654219832	3.961707	E9B3W9	tr|E9B3W9|E9B3W9_LEIMU Cysteine conjugate beta-lyase,ami	4
4.669605624	3.961304	E9AJT7	tr|E9AJT7|E9AJT7_LEIMU Putative 26S protease regulatory sub	4
4.296476408	3.960821	E9AUB1	tr|E9AUB1|E9AUB1_LEIMU Uncharacterized protein OS=Leishr	5
4.692100299	3.960817	E9ALK7	tr|E9ALK7|E9ALK7_LEIMU Rab GDP dissociation inhibitor OS=L	4
5.417887732	3.959734	E9AU34	tr|E9AU34|E9AU34_LEIMU Uncharacterized protein OS=Leishn	3
4.446524132	3.952987	E9B1B8	tr|E9B1B8|E9B1B8_LEIMU Uncharacterized protein OS=Leishm	6
5.284048531	3.95199	E9B263	tr|E9B263|E9B263_LEIMU 2-oxoglutarate dehydrogenase, e3 c	2
4.299986401	3.951207	E9AX02	tr|E9AX02|E9AX02_LEIMU Uncharacterized protein OS=Leishm	2
4.668990465	3.949835	E9AQK4	tr|E9AQK4|E9AQK4_LEIMU Putative 60S ribosomal protein L21	7
4.555949811	3.948825	E9B5Z7;E9B	tr|E9B5Z7|E9B5Z7_LEIMU Putative NADH-dependent fumarate	7
5.62993991	3.946679	E9AQS9	tr|E9AQS9|E9AQS9_LEIMU Coatomer subunit delta OS=Leishm	6
4.660579427	3.946393	E8NHQ7;E9.	tr|E8NHQ7|E8NHQ7_LEIMU Putative ATPase alpha subunit OS=	18
4.84962204	3.944696	E9AZU7	tr|E9AZU7|E9AZU7_LEIMU Putative phenylalanine-4-hydroxyla	4
4.043133976	3.943151	E9ARU5	tr|E9ARU5|E9ARU5_LEIMU Uncharacterized protein OS=Leishr	2
4.670788483	3.943065	E9B3K3	tr|E9B3K3|E9B3K3_LEIMU Thiol-dependent reductase 1 OS=Le	3
3.946307604	3.942149	E9AYL8	tr|E9AYL8|E9AYL8_LEIMU Uncharacterized protein OS=Leishma	2
4.37954886	3.94161	E9AYI1	tr|E9AYI1|E9AYI1_LEIMU Uncharacterized protein OS=Leishma	5
4.313329009	3.940532	E9AQ55	tr|E9AQ55|E9AQ55_LEIMU Uncharacterized protein OS=Leishr	2
5.128451734	3.940353	E9AVX3	tr|E9AVX3|E9AVX3_LEIMU Putative centrin (Ca2+-binding ef-ha	5
4.716973459	3.93955	E9ANV2	tr|E9ANV2|E9ANV2_LEIMU Putative cysteinyl-tRNA synthetase	9
4.628150454	3.938524	E9AXE4	tr|E9AXE4|E9AXE4_LEIMU Putative epsin OS=Leishmania mexi	7
3.977019652	3.938249	E9AWM2	tr|E9AWM2|E9AWM2_LEIMU Uncharacterized protein OS=Leis	3
4.65235109	3.938218	E9AQJ5	tr|E9AQJ5|E9AQJ5_LEIMU Putative tubulin tyrosine ligase OS=I	2
4.462781558	3.938128	E9AVN0	tr|E9AVN0|E9AVN0_LEIMU Uncharacterized protein OS=Leishr	4
4.764605712	3.937897	E9AZ89;A5J	tr|E9AZ89|E9AZ89_LEIMU Putative trypanothione synthetase (	4
5.194446395	3.937004	E9AMU9	tr|E9AMU9|E9AMU9_LEIMU Elongation factor-1 gamma OS=Le	8
4.44890903	3.935202	E9AUN2	tr|E9AUN2|E9AUN2_LEIMU Uncharacterized protein OS=Leishı	3
4.523131232	3.933611	E9AZG6	tr|E9AZG6|E9AZG6_LEIMU Uncharacterized protein OS=Leishm	3
4.556669151	3.932363	E9B4S1;Q9X	tr|E9B4S1|E9B4S1_LEIMU p-glycoprotein OS=Leishmania mexi	8

4.967902548	3.931527	E9B2A8	tr|E9B2A8|E9B2A8_LEIMU Putative peptidase OS=Leishmania r	4
4.891012062	3.931062	E9AJE9	tr|E9AJE9|E9AJE9_LEIMU Uncharacterized protein OS=Leishma	2
5.122380456	3.927659	E9ALL2	tr|E9ALL2|E9ALL2_LEIMU Uncharacterized protein OS=Leishma	7
5.573300408	3.9269	E9B1W4;E9	tr|E9B1W4|E9B1W4_LEIMU Thiolase protein-like protein OS=L	4
5.02297911	3.92323	E9ASW6	tr|E9ASW6|E9ASW6_LEIMU Putative 60S ribosomal protein L3	2
4.907323211	3.921328	E9B2F6	tr|E9B2F6|E9B2F6_LEIMU Uncharacterized protein OS=Leishm	8
5.443407694	3.920776	E9B2A6;E9E	tr|E9B2A6|E9B2A6_LEIMU Iron/zinc transporter protein-like pr	2
5.383313596	3.920771	E9B5J3	tr|E9B5J3|E9B5J3_LEIMU Nucleolar protein family a member-li	6
4.63349561	3.920763	E9B6L7	tr|E9B6L7|E9B6L7_LEIMU Uncharacterized protein OS=Leishma	2
4.427422435	3.920558	E9AMV9;E9	tr|E9AMV9|E9AMV9_LEIMU Putative eukaryotic translation ini	9
4.913594447	3.919469	E9AP62;E8N	tr|E9AP62|E9AP62_LEIMU Tubulin alpha chain OS=Leishmania	37
4.639713357	3.918029	E9AYB7	tr|E9AYB7|E9AYB7_LEIMU Prefoldin subunit 3 OS=Leishmania r	4
4.863929057	3.917586	E9B0J8	tr|E9B0J8|E9B0J8_LEIMU GrpE protein homolog OS=Leishmani	8
5.090409109	3.917311	E9B0S3	tr|E9B0S3|E9B0S3_LEIMU Putative kinesin OS=Leishmania mex	3
4.292940114	3.916954	E9ALV8;A5J	tr|E9ALV8|E9ALV8_LEIMU Putative tryparedoxin OS=Leishman	3
4.956841907	3.916562	E9AWB3	tr|E9AWB3|E9AWB3_LEIMU Uncharacterized protein OS=Leish	4
4.585547344	3.914568	E9AK81	tr|E9AK81|E9AK81_LEIMU Uncharacterized protein OS=Leishm	14
5.128911307	3.914	E9AX08	tr|E9AX08|E9AX08_LEIMU Succinate dehydrogenase [ubiquino	12
4.627200129	3.91282	E9AV44	tr|E9AV44|E9AV44_LEIMU Proteasome regulatory non-ATP-as	3
4.224711401	3.912214	E9B5M9	tr|E9B5M9|E9B5M9_LEIMU Proteasome subunit beta OS=Leish	2
4.518307273	3.910712	E9AR16	tr|E9AR16|E9AR16_LEIMU Uncharacterized protein OS=Leishm	4
4.931298699	3.90814	E9AZB9	tr|E9AZB9|E9AZB9_LEIMU Uncharacterized protein OS=Leishm	3
4.246881398	3.902327	E9AU28	tr|E9AU28|E9AU28_LEIMU Uncharacterized protein OS=Leishn	2
5.468888867	3.899726	E9AJM8	tr|E9AJM8|E9AJM8_LEIMU Uncharacterized protein OS=Leishn	5
4.73889497	3.899369	E9ALE4	tr|E9ALE4|E9ALE4_LEIMU Uncharacterized protein OS=Leishma	4
3.92518319	3.897258	E9AJD5	tr|E9AJD5|E9AJD5_LEIMU Beta eliminating lyase,putative OS=L	3
5.009105205	3.896212	E9B493	tr|E9B493|E9B493_LEIMU Putative carboxypeptidase OS=Leish	10
4.716195268	3.892809	E9AWW4	tr|E9AWW4|E9AWW4_LEIMU Uncharacterized protein OS=Lei	2
4.980978701	3.892804	Q6TUJ5;E9E	tr|Q6TUJ5|Q6TUJ5_LEIME Arginase OS=Leishmania mexicana (	8
4.460759924	3.89241	E9B6Q7	tr|E9B6Q7|E9B6Q7_LEIMU Putative 60S ribosomal protein L23	7
4.503330089	3.892346	E9B344	tr|E9B344|E9B344_LEIMU Uncharacterized protein OS=Leishm	2
4.651049873	3.890501	E9AVA9	tr|E9AVA9|E9AVA9_LEIMU Putative adenylate kinase OS=Leish	7
4.485278858	3.888812	E9ARD8	tr|E9ARD8|E9ARD8_LEIMU Uncharacterized protein OS=Leishn	8
4.848945817	3.888259	E9AXZ8;E9A	tr|E9AXZ8|E9AXZ8_LEIMU Putative 60S ribosomal protein L7 O	12
4.369777384	3.887773	E9AXG0	tr|E9AXG0|E9AXG0_LEIMU Prefoldin subunit 4 OS=Leishmania	3
3.199316218	3.887572	E9AZG5	tr|E9AZG5|E9AZG5_LEIMU Uncharacterized protein OS=Leishm	3
4.024647444	3.887023	E9AYE2	tr|E9AYE2|E9AYE2_LEIMU Putative 40S ribosomal protein S33 (	3
5.364636773	3.886075	E9ANQ5	tr|E9ANQ5|E9ANQ5_LEIMU Uncharacterized protein OS=Leishi	7
5.218374543	3.884272	E9AZ50	tr|E9AZ50|E9AZ50_LEIMU Uncharacterized protein OS=Leishm	4
4.69937929	3.88305	E9ASH6;O0(	tr|E9ASH6|E9ASH6_LEIMU Elongation factor G, mitochondrial (	6
3.58643992	3.88177	E9AS31	tr|E9AS31|E9AS31_LEIMU Putative signal recognition particle p	2
4.89415085	3.880177	E9AMS8;A7	tr|E9AMS8|E9AMS8_LEIMU Oligopeptidase b OS=Leishmania r	5
5.095203346	3.879864	E9B669;E9A	tr|E9B669|E9B669_LEIMU Putative 60S Ribosomal protein L36	7
4.765305154	3.879016	E9ANQ3;C7	tr|E9ANQ3|E9ANQ3_LEIMU Putative lanosterol 14-alpha-deme	5
4.871576804	3.878402	E9B182	tr|E9B182|E9B182_LEIMU Putative homoserine kinase OS=Leis	2
4.683426067	3.876645	E9ATZ6	tr|E9ATZ6|E9ATZ6_LEIMU Putative isoleucyl-tRNA synthetase (	11
4.823385379	3.875745	E9ATW8	tr|E9ATW8|E9ATW8_LEIMU Uncharacterized protein OS=Leish	6
5.376248752	3.875367	E9B5H9	tr|E9B5H9|E9B5H9_LEIMU Small nuclear ribonucleoprotein Sm	2
5.658149395	3.875222	E9B1S0	tr|E9B1S0|E9B1S0_LEIMU Putative vacuolar-type proton transl	5
4.543315972	3.873174	E9AYP1	tr|E9AYP1|E9AYP1_LEIMU Uncharacterized protein OS=Leishm	2
4.821142152	3.872102	E9ATG6	tr|E9ATG6|E9ATG6_LEIMU Putative glycyl tRNA synthetase OS	13
4.883172781	3.866894	E9ATA7	tr|E9ATA7|E9ATA7_LEIMU Uncharacterized protein OS=Leishm	7

4.214783696	3.865961	E9AKR1	tr|E9AKR1|E9AKR1_LEIMU Uncharacterized protein OS=Leishm	3
5.193676496	3.864197	E9AZT2	tr|E9AZT2|E9AZT2_LEIMU Putative electron-transfer-flavoprot	5
4.909963991	3.863464	E9ASV1	tr|E9ASV1|E9ASV1_LEIMU Putative exoribonuclease 2 OS=Leisl	4
5.069589328	3.862456	E9B369	tr|E9B369|E9B369_LEIMU Uncharacterized protein OS=Leishm	2
5.428228031	3.86187	E9B529	tr|E9B529|E9B529_LEIMU DNAj-like protein OS=Leishmania m	3
5.486811014	3.853285	E9ANY1;P42	tr|E9ANY1|E9ANY1_LEIMU Glucose-6-phosphate isomerase OS	7
4.521837235	3.85004	E9B0V2	tr|E9B0V2|E9B0V2_LEIMU Putative queuine tRNA-ribosyltrans	6
4.752892023	3.844936	E9AUD5	tr|E9AUD5|E9AUD5_LEIMU Putative eukaryotic translation init	15
3.815342284	3.843708	E9AMV0	tr|E9AMV0|E9AMV0_LEIMU Uncharacterized protein OS=Leish	2
4.909690159	3.839574	E9B466	tr|E9B466|E9B466_LEIMU Uncharacterized protein OS=Leishm	4
4.787975359	3.835709	E9AY41	tr|E9AY41|E9AY41_LEIMU Putative 10 kDa heat shock protein	4
4.90445252	3.833887	E9AW08	tr|E9AW08|E9AW08_LEIMU Uncharacterized protein OS=Leish	14
5.013963341	3.828831	E9AST0	tr|E9AST0|E9AST0_LEIMU Uncharacterized protein OS=Leishm	2
5.831141867	3.828497	E9AU45	tr|E9AU45|E9AU45_LEIMU Uncharacterized protein OS=Leishm	2
5.376776152	3.827591	E9B2X2	tr|E9B2X2|E9B2X2_LEIMU Putative heat shock protein-like pro	3
4.709354481	3.827513	E9AYA9	tr|E9AYA9|E9AYA9_LEIMU Uncharacterized protein OS=Leishm	25
5.331035569	3.826853	E9B6R2	tr|E9B6R2|E9B6R2_LEIMU Proteasome subunit beta OS=Leishr	3
4.682329918	3.825996	E9AKW5	tr|E9AKW5|E9AKW5_LEIMU Uncharacterized protein OS=Leish	2
4.974610326	3.824035	E9AX53	tr|E9AX53|E9AX53_LEIMU 40S ribosomal protein S8 OS=Leishn	9
4.735486738	3.822934	E9B5S5	tr|E9B5S5|E9B5S5_LEIMU Putative ATP-dependent DEAD-box I	7
5.606007653	3.82283	E9B2X1	tr|E9B2X1|E9B2X1_LEIMU Uncharacterized protein OS=Leishm	8
4.713408867	3.819468	E9AY83	tr|E9AY83|E9AY83_LEIMU Uncharacterized protein OS=Leishm	3
5.271683271	3.816902	E9B492	tr|E9B492|E9B492_LEIMU Uncharacterized protein OS=Leishm	3
4.124295033	3.816831	E9ATF1	tr|E9ATF1|E9ATF1_LEIMU Uncharacterized protein OS=Leishm	2
5.280180465	3.815642	E9ANY7	tr|E9ANY7|E9ANY7_LEIMU Uncharacterized protein OS=Leishn	3
4.855065714	3.814173	E9AXD9	tr|E9AXD9|E9AXD9_LEIMU DNA-directed RNA polymerase sub	2
5.016493803	3.813162	E9ASE2	tr|E9ASE2|E9ASE2_LEIMU eIF3-interacting protein-like protein	9
5.160863567	3.810977	E9ATK7	tr|E9ATK7|E9ATK7_LEIMU Uncharacterized protein OS=Leishm	11
5.216014079	3.807854	E9B038	tr|E9B038|E9B038_LEIMU Uncharacterized protein OS=Leishm	12
4.65191377	3.805648	Q4TZP6;E9A	tr|Q4TZP6|Q4TZP6_LEIME Glutamine synthetase OS=Leishman	10
4.627431069	3.798865	E9AYD4	tr|E9AYD4|E9AYD4_LEIMU Trifunctional enzyme alpha subunit	2
4.414040713	3.797199	E9B705	tr|E9B705|E9B705_LEIMU Putative peptidyl-prolyl cis-trans iso	4
5.142874331	3.791908	E9AMD6	tr|E9AMD6|E9AMD6_LEIMU Uncharacterized protein OS=Leish	4
5.478257066	3.789674	E9B3U9	tr|E9B3U9|E9B3U9_LEIMU Putative pumilio protein OS=Leishm	11
5.467000047	3.787272	E9AWH9	tr|E9AWH9|E9AWH9_LEIMU Uncharacterized protein OS=Leish	5
5.913568164	3.787107	E9AUL2	tr|E9AUL2|E9AUL2_LEIMU Uncharacterized protein OS=Leishm	2
5.2765197	3.78671	E9B645	tr|E9B645|E9B645_LEIMU Putative 60S ribosomal protein L26	2
5.062264082	3.785912	E9B677	tr|E9B677|E9B677_LEIMU Uncharacterized protein OS=Leishm	6
6.039656275	3.785133	E9ANB6	tr|E9ANB6|E9ANB6_LEIMU Uncharacterized protein OS=Leishr	2
5.195603964	3.782846	E9B0D7	tr|E9B0D7|E9B0D7_LEIMU Alkyl dihydroxyacetonephosphate s	6
5.362972141	3.782113	E9B1P8	tr|E9B1P8|E9B1P8_LEIMU Uncharacterized protein OS=Leishm	6
5.31093676	3.781922	E9AV22	tr|E9AV22|E9AV22_LEIMU Dihydrolipoamide acetyltransferase	3
4.683742298	3.781487	E9ALS3;E8N	tr|E9ALS3|E9ALS3_LEIMU Uncharacterized protein OS=Leishm	3
3.917529633	3.779873	E9ASD1	tr|E9ASD1|E9ASD1_LEIMU Uncharacterized protein OS=Leishm	2
5.230279193	3.779117	E9AXJ1	tr|E9AXJ1|E9AXJ1_LEIMU Aldehyde dehydrogenase, mitochon	10
5.186484636	3.778769	E9ATB0	tr|E9ATB0|E9ATB0_LEIMU Putative actin-like protein OS=Leish	2
4.266080818	3.772958	E9AT88	tr|E9AT88|E9AT88_LEIMU Putative ATP synthase OS=Leishmar	3
4.349505119	3.770957	E9APZ6	tr|E9APZ6|E9APZ6_LEIMU Putative glutathione-S-transferase/	3
4.186255925	3.770187	E9AWA7;Q9	tr|E9AWA7|E9AWA7_LEIMU Cytosolic leucyl aminopeptidase (	3
5.054674829	3.769315	E9AZ84	tr|E9AZ84|E9AZ84_LEIMU Uncharacterized protein OS=Leishm	8
4.909657775	3.765752	E9AYZ5	tr|E9AYZ5|E9AYZ5_LEIMU Putative isovaleryl-coA dehydrogena	5
5.347010472	3.765585	E9APB1	tr|E9APB1|E9APB1_LEIMU Mitochondrial processing peptidase	8

4.852606994	3.761787	E9B052	tr|E9B052|E9B052_LEIMU Eukaryotic translation initiation fact	5
5.187535683	3.758348	E9AJW8	tr|E9AJW8|E9AJW8_LEIMU Uncharacterized protein OS=Leishr	7
4.445292919	3.757989	E9B1R9	tr|E9B1R9|E9B1R9_LEIMU Uncharacterized protein OS=Leishm	3
5.02438489	3.752437	E9ASA6	tr|E9ASA6|E9ASA6_LEIMU Peptidylprolyl isomerase OS=Leishm	7
4.737837571	3.747609	E9AY61	tr|E9AY61|E9AY61_LEIMU Putative asparagine synthetase a (Pu	7
4.595286249	3.747593	E9B2E4	tr|E9B2E4|E9B2E4_LEIMU Dynein light chain OS=Leishmania m	2
5.174313006	3.747526	E9B1B6	tr|E9B1B6|E9B1B6_LEIMU Uncharacterized protein OS=Leishm	3
4.673036841	3.747321	E9B398	tr|E9B398|E9B398_LEIMU Uncharacterized protein OS=Leishm	3
5.350600414	3.74562	E9AZ25	tr|E9AZ25|E9AZ25_LEIMU Uncharacterized protein OS=Leishm	9
5.066229449	3.741154	E9AQN5	tr|E9AQN5|E9AQN5_LEIMU Transaldolase OS=Leishmania mex	5
4.628716377	3.738142	E9APD0	tr|E9APD0|E9APD0_LEIMU NADH-cytochrome b5 reductase OS	2
4.220376605	3.737502	E9B5K8	tr|E9B5K8|E9B5K8_LEIMU Uncharacterized protein OS=Leishm	2
4.232713807	3.734085	E9B5Z2;E9B	tr|E9B5Z2|E9B5Z2_LEIMU Oligosaccharyl transferase-like prote	3
5.139846361	3.73182	E9ATK1;A1Y	tr|E9ATK1|E9ATK1_LEIMU Putative oxidoreductase OS=Leishm	7
5.1475509	3.731137	E9ASF6	tr|E9ASF6|E9ASF6_LEIMU Uncharacterized protein OS=Leishma	4
4.551478459	3.730113	E9AQ99;E9E	tr|E9AQ99|E9AQ99_LEIMU 60S ribosomal protein L6 OS=Leishi	11
4.327356438	3.730058	E9AZH2	tr|E9AZH2|E9AZH2_LEIMU Proteasome subunit beta OS=Leishi	2
4.360806291	3.726575	E9AP69	tr|E9AP69|E9AP69_LEIMU Uncharacterized protein OS=Leishm	6
4.710356855	3.716446	E9B560	tr|E9B560|E9B560_LEIMU Uncharacterized protein OS=Leishm	5
4.760334672	3.716432	E9ATU7	tr|E9ATU7|E9ATU7_LEIMU Uncharacterized protein OS=Leishn	2
4.355595603	3.712297	E9AWL7	tr|E9AWL7|E9AWL7_LEIMU Dynein intermediate-chain-like pro	3
4.317574752	3.709748	E9AZY4	tr|E9AZY4|E9AZY4_LEIMU Uncharacterized protein OS=Leishm	2
4.21817367	3.708354	E9AZZ2	tr|E9AZZ2|E9AZZ2_LEIMU Proteasome regulatory non-ATP-ase	5
4.7549718	3.70811	E9API2	tr|E9API2|E9API2_LEIMU Ubiquitin-conjugating enzyme-like pr	3
4.994148144	3.707471	E9AVK4	tr|E9AVK4|E9AVK4_LEIMU Uncharacterized protein OS=Leishm	20
4.150714501	3.70615	E9B158	tr|E9B158|E9B158_LEIMU Uncharacterized protein OS=Leishm	2
4.717634973	3.705839	O61059;E9A	tr|O61059|O61059_LEIME Glucose transporter OS=Leishmania	5
4.221294773	3.700783	E9AZB6	tr|E9AZB6|E9AZB6_LEIMU Putative RNA-binding protein OS=Le	6
5.80616239	3.694184	E9B485	tr|E9B485|E9B485_LEIMU Uncharacterized protein OS=Leishm	6
4.7090433	3.693969	E9B1M1	tr|E9B1M1|E9B1M1_LEIMU Putative ATP-dependent zinc meta	2
5.455264927	3.693897	E9AQY7	tr|E9AQY7|E9AQY7_LEIMU Uncharacterized protein OS=Leishn	3
6.066195919	3.688719	E9AKI9	tr|E9AKI9|E9AKI9_LEIMU S-methyl-5-thioadenosine phosphory	2
5.305382223	3.687896	E9B760	tr|E9B760|E9B760_LEIMU Uncharacterized protein OS=Leishm	6
5.003351468	3.687547	E9B0V4	tr|E9B0V4|E9B0V4_LEIMU Putative splicing factor 3a OS=Leish	3
4.703562696	3.687438	E9ASZ0	tr|E9ASZ0|E9ASZ0_LEIMU Putative DEAD box RNA helicase OS=	7
4.770750083	3.687358	E9AJT5	tr|E9AJT5|E9AJT5_LEIMU Uncharacterized protein OS=Leishma	5
4.708251541	3.677104	E9AK26	tr|E9AK26|E9AK26_LEIMU Putative calpain-like cysteine peptic	5
4.906835243	3.668107	E9AXX5	tr|E9AXX5|E9AXX5_LEIMU Uncharacterized protein OS=Leishm	7
5.352997054	3.664499	E9ANQ6	tr|E9ANQ6|E9ANQ6_LEIMU Putative eukaryotic release factor	7
5.356544105	3.663555	E9AXX0	tr|E9AXX0|E9AXX0_LEIMU Uncharacterized protein OS=Leishm	3
5.22652991	3.661301	E9AV10	tr|E9AV10|E9AV10_LEIMU Uncharacterized protein OS=Leishm	8
4.757038173	3.659657	E9AXH4	tr|E9AXH4|E9AXH4_LEIMU Tubulin gamma chain OS=Leishmar	3
3.744200089	3.658121	E9AZB2	tr|E9AZB2|E9AZB2_LEIMU Uncharacterized protein OS=Leishm	2
5.804905186	3.657656	E9B2F8	tr|E9B2F8|E9B2F8_LEIMU Protein SEY1 homolog OS=Leishman	2
4.81776419	3.656165	E9B6B5	tr|E9B6B5|E9B6B5_LEIMU Putative aminopeptidase P OS=Leisl	3
5.125976108	3.653711	E9AYX8	tr|E9AYX8|E9AYX8_LEIMU Putative small GTP-binding protein I	5
5.075823132	3.649798	E9B5U7	tr|E9B5U7|E9B5U7_LEIMU Uncharacterized protein OS=Leishm	8
5.273715687	3.649065	E9B4C7	tr|E9B4C7|E9B4C7_LEIMU Uncharacterized protein OS=Leishm	6
4.505134972	3.647882	E9ANM5	tr|E9ANM5|E9ANM5_LEIMU Uncharacterized protein OS=Leisl	7
5.142493021	3.646413	E9B759	tr|E9B759|E9B759_LEIMU Putative isomerase (Isopentenyl-dip	4
4.472858974	3.644298	E9B378	tr|E9B378|E9B378_LEIMU Tubulin-specific chaperone A OS=Le	2
5.26387181	3.643989	E9AX67	tr|E9AX67|E9AX67_LEIMU Putative 60S ribosomal protein L12	2

5.034982913	3.640759	E9AL86	tr|E9AL86|E9AL86_LEIMU Uncharacterized protein OS=Leishm	2
5.477420858	3.640547	E9AVJ1	tr|E9AVJ1|E9AVJ1_LEIMU Transcription factor-like protein OS=	2
4.908755457	3.637644	E9AP81	tr|E9AP81|E9AP81_LEIMU 40S ribosomal protein S12 OS=Leish	7
5.22648906	3.63747	Q71PB4;E9E	tr|Q71PB4|Q71PB4_LEIME Broad specificity aminotransferase	6
4.434726476	3.637227	E9AP97	tr|E9AP97|E9AP97_LEIMU Ubiquitin-like protein OS=Leishman	2
4.672656223	3.63648	E9B2J9	tr|E9B2J9|E9B2J9_LEIMU Putative RNA binding protein OS=Lei:	11
3.258747708	3.636145	E9B6C8	tr|E9B6C8|E9B6C8_LEIMU Uncharacterized protein OS=Leishm	2
6.244127931	3.636123	E9AT66	tr|E9AT66|E9AT66_LEIMU Uncharacterized protein OS=Leishm	2
4.972684214	3.635275	E9AUK0	tr|E9AUK0|E9AUK0_LEIMU CTP synthase OS=Leishmania mexic	5
5.14026192	3.634692	E9AWS4	tr|E9AWS4|E9AWS4_LEIMU Uncharacterized protein OS=Leish	2
5.156170011	3.634431	E9AU86	tr|E9AU86|E9AU86_LEIMU Uncharacterized protein OS=Leishn	2
4.557155864	3.63356	E9B2S6	tr|E9B2S6|E9B2S6_LEIMU Putative ATP-dependent zinc metall	3
4.605031963	3.630113	E9B414	tr|E9B414|E9B414_LEIMU Uncharacterized protein OS=Leishm	8
5.272922232	3.630087	E9AJL7	tr|E9AJL7|E9AJL7_LEIMU Uncharacterized protein OS=Leishma	3
4.825481183	3.628848	E9ASP5;E9A	tr|E9ASP5|E9ASP5_LEIMU Putative 40S ribosomal protein S9 O	7
4.61714563	3.625839	E9B1S3	tr|E9B1S3|E9B1S3_LEIMU Uncharacterized protein OS=Leishm	2
4.839127111	3.624257	E9B520	tr|E9B520|E9B520_LEIMU Putative asparaginyl-tRNA synthetas	9
4.771993721	3.620057	E9AXI9	tr|E9AXI9|E9AXI9_LEIMU DNAj-like protein OS=Leishmania me	3
4.567514656	3.616625	E9AJR2	tr|E9AJR2|E9AJR2_LEIMU Uncharacterized protein OS=Leishma	3
5.052224472	3.616265	E9AJR0	tr|E9AJR0|E9AJR0_LEIMU Putative ribosomal protein L38 OS=L	5
4.334160153	3.614684	E9APH2	tr|E9APH2|E9APH2_LEIMU Putative Ran-binding protein 1 OS=	2
5.044403028	3.613212	E9B1Z2	tr|E9B1Z2|E9B1Z2_LEIMU Putative dihydrouridine synthase (D	2
5.401927271	3.611268	E9B074	tr|E9B074|E9B074_LEIMU Uncharacterized protein OS=Leishm	2
5.110947323	3.606343	E9AXK0	tr|E9AXK0|E9AXK0_LEIMU Uncharacterized protein OS=Leishm	6
4.364270677	3.60369	E9AMU6	tr|E9AMU6|E9AMU6_LEIMU Uncharacterized protein OS=Leish	5
4.701672593	3.602744	E9B6Z9	tr|E9B6Z9|E9B6Z9_LEIMU Putative MCAK-like kinesin OS=Leish	5
4.773901248	3.601375	E9AQ77	tr|E9AQ77|E9AQ77_LEIMU Uncharacterized protein OS=Leishn	13
4.244302912	3.599925	E9AQL2;Q9I	tr|E9AQL2|E9AQL2_LEIMU Putative aspartate carbamoyltransf	4
4.449026241	3.599174	E9AVT1	tr|E9AVT1|E9AVT1_LEIMU Uncharacterized protein OS=Leishm	3
4.909774469	3.598224	E9AKL5;E8N	tr|E9AKL5|E9AKL5_LEIMU Uncharacterized protein OS=Leishm	5
5.163511464	3.594256	E9ALV6	tr|E9ALV6|E9ALV6_LEIMU Uncharacterized protein OS=Leishm	2
4.650251526	3.591768	E9B5X8;E9B	tr|E9B5X8|E9B5X8_LEIMU Aldose 1-epimerase-like protein OS=	3
4.898273507	3.583246	E9AL83	tr|E9AL83|E9AL83_LEIMU Topoisomerase-related function pro	4
4.861057088	3.58257	E9B2V9;E9E	tr|E9B2V9|E9B2V9_LEIMU Superoxide dismutase OS=Leishmar	3
5.545995881	3.577925	E9ATL1	tr|E9ATL1|E9ATL1_LEIMU Uncharacterized protein OS=Leishm	5
4.831825154	3.57511	E9AX62	tr|E9AX62|E9AX62_LEIMU Uncharacterized protein OS=Leishm	4
4.874282896	3.574859	E9AT87	tr|E9AT87|E9AT87_LEIMU Uncharacterized protein OS=Leishm	3
3.785548924	3.572887	E9AY79	tr|E9AY79|E9AY79_LEIMU Uncharacterized protein OS=Leishm	2
4.826358686	3.570502	E9B2Z9	tr|E9B2Z9|E9B2Z9_LEIMU Uncharacterized protein OS=Leishm	5
5.282931007	3.570046	E9B6Z2	tr|E9B6Z2|E9B6Z2_LEIMU Uncharacterized protein OS=Leishm	3
5.828944639	3.5667	E9ALH0	tr|E9ALH0|E9ALH0_LEIMU Uncharacterized protein OS=Leishm	5
5.081562348	3.564918	E9APM4	tr|E9APM4|E9APM4_LEIMU Proteasome subunit alpha type OS	3
4.031736989	3.560864	E9B2V2	tr|E9B2V2|E9B2V2_LEIMU Uncharacterized protein OS=Leishm	2
5.23121764	3.560352	E9ATU0	tr|E9ATU0|E9ATU0_LEIMU Uncharacterized protein OS=Leishn	4
4.203208028	3.559825	E9B1A1	tr|E9B1A1|E9B1A1_LEIMU Uncharacterized protein OS=Leishm	2
4.443358211	3.559701	E9AWF1	tr|E9AWF1|E9AWF1_LEIMU Putative dynein heavy chain OS=Le	4
4.830204209	3.556686	E9AQH1	tr|E9AQH1|E9AQH1_LEIMU Eukaryotic translation initiation fac	5
4.865465824	3.556137	E9B5P8	tr|E9B5P8|E9B5P8_LEIMU Uncharacterized protein OS=Leishm	4
4.745169851	3.549092	E9B0U8	tr|E9B0U8|E9B0U8_LEIMU Uncharacterized protein OS=Leishm	2
4.963973353	3.548466	E9ARZ1	tr|E9ARZ1|E9ARZ1_LEIMU Putative 40S ribosomal protein S13	8
3.996728476	3.546169	E9B357	tr|E9B357|E9B357_LEIMU Uncharacterized protein OS=Leishm	2
4.553327808	3.542215	E9ASM6	tr|E9ASM6|E9ASM6_LEIMU Uncharacterized protein OS=Leishi	5

5.088928276	3.534271	E9AQ94	tr|E9AQ94|E9AQ94_LEIMU Putative 40S ribosomal protein S3 (	5
4.820166185	3.533384	E9AZ67	tr|E9AZ67|E9AZ67_LEIMU Uncharacterized protein OS=Leishm	4
5.214424821	3.533139	E9AMB1	tr|E9AMB1|E9AMB1_LEIMU Putative mitochondrial associated	3
4.607424625	3.528509	E9AJV8	tr|E9AJV8|E9AJV8_LEIMU Uncharacterized protein OS=Leishma	3
4.772899272	3.522369	E9AMX3	tr|E9AMX3|E9AMX3_LEIMU Putative AAA family ATPase OS=Le	4
4.7739801	3.515034	E9AKM1	tr|E9AKM1|E9AKM1_LEIMU V-type proton ATPase subunit OS=	2
5.146598093	3.514084	E9AVN9	tr|E9AVN9|E9AVN9_LEIMU Putative proteasome regulatory AT	2
4.582753689	3.507886	E9AQX4	tr|E9AQX4|E9AQX4_LEIMU Putative prohibitin OS=Leishmania	3
4.741596337	3.50737	E9AMC2	tr|E9AMC2|E9AMC2_LEIMU Uncharacterized protein OS=Leish	7
4.644757428	3.505534	E9ANG0	tr|E9ANG0|E9ANG0_LEIMU Uncharacterized protein OS=Leishı	3
4.415898479	3.500182	E9AVC8	tr|E9AVC8|E9AVC8_LEIMU Uncharacterized protein OS=Leishm	2
5.172339964	3.489302	E9B620	tr|E9B620|E9B620_LEIMU Putative threonyl-tRNA synthetase (	15
4.876175001	3.48726	E9B260;Q25	tr|E9B260|E9B260_LEIMU DNA-directed RNA polymerase subu	7
4.883234219	3.482195	E9AV88	tr|E9AV88|E9AV88_LEIMU Putative kinesin OS=Leishmania me	3
5.269415532	3.473055	E9ANM2;E9	tr|E9ANM2|E9ANM2_LEIMU Putative 40S ribosomal protein S2	3
4.709719936	3.471505	E9B6X6	tr|E9B6X6|E9B6X6_LEIMU Uncharacterized protein OS=Leishm	4
5.163210426	3.469893	E9B2N8	tr|E9B2N8|E9B2N8_LEIMU Uncharacterized protein OS=Leishm	2
4.240631963	3.468148	E9B694	tr|E9B694|E9B694_LEIMU Uncharacterized protein OS=Leishm	2
4.998078995	3.467073	E9B547	tr|E9B547|E9B547_LEIMU Uncharacterized protein OS=Leishm	4
5.163368087	3.465377	E9B6X2	tr|E9B6X2|E9B6X2_LEIMU Putative mitochondrial phosphate tı	5
4.200241969	3.462568	E9B360	tr|E9B360|E9B360_LEIMU Putative RNA-binding protein OS=Le	2
4.40019318	3.462121	E9AKY6	tr|E9AKY6|E9AKY6_LEIMU Putative deoxyribose-phosphate ald	2
4.369822865	3.457567	E9B4E9	tr|E9B4E9|E9B4E9_LEIMU Uncharacterized protein OS=Leishm	3
4.610741097	3.448931	O76285;E9A	tr|O76285|O76285_LEIAM Ribonucleotide reductase M2 subur	4
4.945842701	3.448687	E9ANN4	tr|E9ANN4|E9ANN4_LEIMU Putative 60S ribosomal protein L24	2
4.646623798	3.446906	E9AV16	tr|E9AV16|E9AV16_LEIMU Putative DnaJ protein OS=Leishman	6
4.572938209	3.443773	E9APA5	tr|E9APA5|E9APA5_LEIMU Uncharacterized protein OS=Leishm	2
4.573597912	3.437887	E9AKA4;C6k	tr|E9AKA4|E9AKA4_LEIMU Actin OS=Leishmania mexicana (stra	14
5.475413569	3.431223	E9APQ1	tr|E9APQ1|E9APQ1_LEIMU Uncharacterized protein OS=Leishr	5
4.7404212	3.430161	E9AKM5	tr|E9AKM5|E9AKM5_LEIMU Uncharacterized protein OS=Leish	2
5.398136564	3.429595	E9AZ08	tr|E9AZ08|E9AZ08_LEIMU Putative cysteine desulfurase OS=Le	6
5.196162899	3.426702	E9B0B0	tr|E9B0B0|E9B0B0_LEIMU Putative dynein heavy chain OS=Lei:	5
5.304626929	3.423057	E9B397	tr|E9B397|E9B397_LEIMU Pyrroline-5-carboxylate synthetase-	2
4.860292282	3.419999	E9AVC7	tr|E9AVC7|E9AVC7_LEIMU 2-oxoisovalerate dehydrogenase su	4
4.653507393	3.414946	E9B4N4	tr|E9B4N4|E9B4N4_LEIMU Proteasome regulatory non-ATP-as	2
4.683851871	3.411289	E9AN96	tr|E9AN96|E9AN96_LEIMU Uncharacterized protein OS=Leishn	2
5.471825393	3.408538	E9AMC1	tr|E9AMC1|E9AMC1_LEIMU Uncharacterized protein OS=Leish	5
4.872016181	3.407445	E9AW77	tr|E9AW77|E9AW77_LEIMU Uncharacterized protein OS=Leish	2
4.416633859	3.403603	E9AKC2	tr|E9AKC2|E9AKC2_LEIMU Dihydrolipoamide acetyltransferase	2
4.384719132	3.398496	E9AXT6	tr|E9AXT6|E9AXT6_LEIMU Uncharacterized protein OS=Leishm	2
4.830928628	3.394872	E9AW79	tr|E9AW79|E9AW79_LEIMU Uncharacterized protein OS=Leish	5
5.297507614	3.393244	E9B722	tr|E9B722|E9B722_LEIMU Uncharacterized protein OS=Leishm	3
4.358765995	3.39286	E9AV20	tr|E9AV20|E9AV20_LEIMU Uncharacterized protein OS=Leishm	2
5.15107825	3.391143	E9ALB7	tr|E9ALB7|E9ALB7_LEIMU RNA binding protein-like protein OS	14
5.006592522	3.387983	E9AV49;A0/	tr|E9AV49|E9AV49_LEIMU Putative methionyl-tRNA synthetase	8
5.224660291	3.383366	E9AVM8	tr|E9AVM8|E9AVM8_LEIMU Uncharacterized protein OS=Leish	2
4.666660423	3.379459	E9AQ24	tr|E9AQ24|E9AQ24_LEIMU Lysine--tRNA ligase OS=Leishmania	8
4.580218978	3.376836	Q86PL6;E9A	tr|Q86PL6|Q86PL6_LEIME Paraflagellar rod protein 4 OS=Leish	2
4.252126178	3.37118	E9AU70	tr|E9AU70|E9AU70_LEIMU Uncharacterized protein OS=Leishn	2
4.82052355	3.368586	E9B4G7	tr|E9B4G7|E9B4G7_LEIMU Uncharacterized protein OS=Leishm	6
5.050324271	3.365063	E9AVW8	tr|E9AVW8|E9AVW8_LEIMU Putative farnesyl pyrophosphate s	2
5.43027655	3.362785	E9B1Q2	tr|E9B1Q2|E9B1Q2_LEIMU Uncharacterized protein OS=Leishn	6

5.230951522	3.361893	E9AQX3	tr|E9AQX3|E9AQX3_LEIMU Uncharacterized protein OS=Leishr	6
5.400652788	3.355206	E9AU12	tr|E9AU12|E9AU12_LEIMU tRNA (adenine(58)-N(1))-methyltra	3
5.074083381	3.350886	E9ANB3	tr|E9ANB3|E9ANB3_LEIMU RNA-binding protein-like protein O	5
5.162012577	3.348813	E9ARK0	tr|E9ARK0|E9ARK0_LEIMU Putative 60S ribosomal protein L10a	6
5.747158347	3.347594	E9AQM8	tr|E9AQM8|E9AQM8_LEIMU Uncharacterized protein OS=Leisl	3
4.535135067	3.341085	E9AP43	tr|E9AP43|E9AP43_LEIMU Putative carboxypeptidase (Metallo	2
4.923372877	3.332406	E9AVL4	tr|E9AVL4|E9AVL4_LEIMU Uncharacterized protein OS=Leishm	10
4.892696602	3.33127	E9AR48	tr|E9AR48|E9AR48_LEIMU Uncharacterized protein OS=Leishr	4
5.443283685	3.323018	E9B4J8	tr|E9B4J8|E9B4J8_LEIMU Uncharacterized protein OS=Leishma	7
4.672330521	3.321522	E9AU20	tr|E9AU20|E9AU20_LEIMU Uncharacterized protein OS=Leishn	10
4.444266698	3.317315	E9AK43	tr|E9AK43|E9AK43_LEIMU Uncharacterized protein OS=Leishm	3
6.273276399	3.300904	E9AR97	tr|E9AR97|E9AR97_LEIMU RNA cytidine acetyltransferase OS=l	4
4.472234189	3.298798	E9AZZ7	tr|E9AZZ7|E9AZZ7_LEIMU DNA topoisomerase OS=Leishmania	2
4.717014816	3.295271	E9AYV1	tr|E9AYV1|E9AYV1_LEIMU Putative calpain-like cysteine peptic	8
4.463899128	3.29449	E9AJV4	tr|E9AJV4|E9AJV4_LEIMU Uncharacterized protein OS=Leishma	2
5.256539952	3.28231	E9AYA4	tr|E9AYA4|E9AYA4_LEIMU Uncharacterized protein OS=Leishm	4
4.75069592	3.278465	E9AXM2	tr|E9AXM2|E9AXM2_LEIMU Uncharacterized protein OS=Leish	16
5.102262701	3.278154	E9ALG7	tr|E9ALG7|E9ALG7_LEIMU Putative serine/threonine-protein k	4
5.287702711	3.274379	E9B276	tr|E9B276|E9B276_LEIMU Uncharacterized protein OS=Leishm	4
4.663476646	3.272696	E9AUB0	tr|E9AUB0|E9AUB0_LEIMU Putative prolyl oligopeptidase OS=l	6
6.095810419	3.269946	E9AWD4	tr|E9AWD4|E9AWD4_LEIMU Putative coronin OS=Leishmania i	3
4.851840352	3.26629	E8NHN7;E9I	tr|E8NHN7|E8NHN7_LEIMU WGS CADB00000000 data, contig	4
5.10340543	3.266183	Q25332;Q2!	tr|Q25332|Q25332_LEIME Secreted acid phosphatase 1 (SAP1)	11
5.336868389	3.264125	E9AR33;E9A	tr|E9AR33|E9AR33_LEIMU Uncharacterized protein OS=Leishm	4
5.751810724	3.257864	E9B430	tr|E9B430|E9B430_LEIMU Putative phosphoribosylpyrophosph	2
5.54149575	3.255627	E9AV82	tr|E9AV82|E9AV82_LEIMU Uncharacterized protein OS=Leishm	8
4.687973406	3.25399	E9B2Q9	tr|E9B2Q9|E9B2Q9_LEIMU Cleavage and polyadenylation spec	5
4.555191585	3.252728	E9AZ92	tr|E9AZ92|E9AZ92_LEIMU Uncharacterized protein OS=Leishm	2
4.96344843	3.249777	E9ALA4	tr|E9ALA4|E9ALA4_LEIMU Splicing factor ptsr1-like protein OS=	21
5.426569672	3.245074	E9ARE6	tr|E9ARE6|E9ARE6_LEIMU Putative alpha glucosidase II subuni	2
6.992249506	3.240934	E9AXU5	tr|E9AXU5|E9AXU5_LEIMU Uncharacterized protein OS=Leishn	2
4.234364103	3.223638	E9B235	tr|E9B235|E9B235_LEIMU Uncharacterized protein OS=Leishm	2
4.907902624	3.215418	E9AYF9	tr|E9AYF9|E9AYF9_LEIMU Uncharacterized protein OS=Leishm	17
5.539805353	3.214923	E9B4H4;Q8I	tr|E9B4H4|E9B4H4_LEIMU Glucose-6-phosphate 1-dehydroger	7
5.713772845	3.212655	E9AWS6;A0	tr|E9AWS6|E9AWS6_LEIMU Triosephosphate isomerase OS=Le	2
4.114178579	3.20737	E9AY72	tr|E9AY72|E9AY72_LEIMU Uncharacterized protein OS=Leishm	2
3.654440686	3.206979	E9ANX1	tr|E9ANX1|E9ANX1_LEIMU Uncharacterized protein OS=Leishn	2
4.652933656	3.204494	E9AW60	tr|E9AW60|E9AW60_LEIMU Uncharacterized protein OS=Leish	2
4.976169847	3.199362	E9AWK2	tr|E9AWK2|E9AWK2_LEIMU Guanine nucleotide-binding prote	2
5.277805835	3.194966	E9AYP0	tr|E9AYP0|E9AYP0_LEIMU Uncharacterized protein OS=Leishm	2
5.011254391	3.194503	E9AQQ4;A0	tr|E9AQQ4|E9AQQ4_LEIMU Sucrose-phosphate synthase-like p	3
5.475512874	3.193815	E9B6P8	tr|E9B6P8|E9B6P8_LEIMU Putative Gim5A protein (Glycosoma	3
4.695658709	3.193683	E9B2W9	tr|E9B2W9|E9B2W9_LEIMU Uncharacterized protein OS=Leish	4
4.349118829	3.184145	E9AXP7	tr|E9AXP7|E9AXP7_LEIMU Uncharacterized protein OS=Leishm	2
4.909684955	3.180896	E9B278	tr|E9B278|E9B278_LEIMU Putative ADP-ribosylation factor OS=	2
5.05264027	3.180808	Q6T9E2;E9A	tr|Q6T9E2|Q6T9E2_LEIAM Uncharacterized protein OS=Leishm	2
4.981015202	3.17272	E9APV8;E8N	tr|E9APV8|E9APV8_LEIMU Putative kinesin K39 OS=Leishmania	5
5.592540496	3.172146	E9AXC4	tr|E9AXC4|E9AXC4_LEIMU Uncharacterized protein OS=Leishm	3
5.137325278	3.163223	E9AJN3	tr|E9AJN3|E9AJN3_LEIMU Putative ATP-dependent Clp proteas	8
4.74189293	3.143224	E9AYK0	tr|E9AYK0|E9AYK0_LEIMU Uncharacterized protein OS=Leishm	2
5.689169539	3.139943	E9ARF2	tr|E9ARF2|E9ARF2_LEIMU Serine/threonine protein phosphata	4
4.466355518	3.129149	E9AS74	tr|E9AS74|E9AS74_LEIMU Uncharacterized protein OS=Leishm	3

5.381602127	3.124825	E9B0Z8	tr|E9B0Z8|E9B0Z8_LEIMU Conserved zinc-finger protein OS=Le	3
5.165697817	3.120547	Q25327;E9A	tr|Q25327|Q25327_LEIME Membrane-bound acid phosphatase	9
4.802031188	3.116186	E9AZN4	tr|E9AZN4|E9AZN4_LEIMU Uncharacterized protein OS=Leishm	3
4.822097903	3.102993	E9AT28	tr|E9AT28|E9AT28_LEIMU Nuclear pore protein OS=Leishmania	3
4.791916106	3.095139	E9B1D2	tr|E9B1D2|E9B1D2_LEIMU Putative serine/threonine-protein k	11
4.007540076	3.090742	E9AJE1	tr|E9AJE1|E9AJE1_LEIMU Uncharacterized protein OS=Leishma	3
4.795129158	3.089192	E9B4S8	tr|E9B4S8|E9B4S8_LEIMU Putative mitochondrial ATP-depende	2
4.895260888	3.088432	E9AQE3	tr|E9AQE3|E9AQE3_LEIMU Putative ribosomal protein S6 (Nph	2
4.927975999	3.083321	E9AX01	tr|E9AX01|E9AX01_LEIMU Uncharacterized protein OS=Leishm	8
4.917112125	3.055813	E9B3C6;E9B	tr|E9B3C6|E9B3C6_LEIMU Uncharacterized protein (Fragment)	5
5.732265685	3.049799	E9B4J1	tr|E9B4J1|E9B4J1_LEIMU Uncharacterized protein OS=Leishma	3
4.676183403	3.045488	E9AVZ3	tr|E9AVZ3|E9AVZ3_LEIMU Uncharacterized protein OS=Leishm	7
5.022486891	3.038962	E9AKL0;Q86	tr|E9AKL0|E9AKL0_LEIMU Stomatin-like protein OS=Leishmania	5
5.018531568	3.016406	E9B548	tr|E9B548|E9B548_LEIMU Uncharacterized protein OS=Leishm	5
5.570732669	3.013945	E8NHL4	tr|E8NHL4|E8NHL4_LEIMU Brix domain containing-like protein	5
5.306355485	3.008605	E9AXT3	tr|E9AXT3|E9AXT3_LEIMU Uncharacterized protein OS=Leishm	3
5.02947255	2.999018	E9B3Y6	tr|E9B3Y6|E9B3Y6_LEIMU Uncharacterized protein OS=Leishm	2
5.116469035	2.998301	E9B2B1	tr|E9B2B1|E9B2B1_LEIMU Uncharacterized protein OS=Leishm	8
5.36184668	2.988921	E9AVG8	tr|E9AVG8|E9AVG8_LEIMU 60S ribosomal protein L32 OS=Leisl	3
5.275404834	2.970933	E9B6K7	tr|E9B6K7|E9B6K7_LEIMU Putative 60S ribosomal subunit prot	10
4.59807765	2.967065	E9ATR2	tr|E9ATR2|E9ATR2_LEIMU Uncharacterized protein OS=Leishm	3
4.564426639	2.96693	E9B228	tr|E9B228|E9B228_LEIMU Uncharacterized protein OS=Leishm	3
5.138027001	2.96421	E9B6R8	tr|E9B6R8|E9B6R8_LEIMU Uncharacterized protein OS=Leishm	2
5.118054889	2.962158	E9AKC8;E9A	tr|E9AKC8|E9AKC8_LEIMU Putative viscerotropic leishmaniasis	2
6.118007142	2.958272	E9AUC8	tr|E9AUC8|E9AUC8_LEIMU Uncharacterized protein OS=Leishn	3
4.186130222	2.95767	E9AZ12	tr|E9AZ12|E9AZ12_LEIMU Uncharacterized protein OS=Leishm	6
5.29987572	2.947287	E9AQZ4	tr|E9AQZ4|E9AQZ4_LEIMU Cystathionine beta-synthase OS=Le	5
4.340597733	2.943685	E9B1H3	tr|E9B1H3|E9B1H3_LEIMU Uncharacterized protein OS=Leishm	2
4.339769299	2.943571	E9AN63	tr|E9AN63|E9AN63_LEIMU Uncharacterized protein OS=Leishn	3
5.031213942	2.938602	E9AK24	tr|E9AK24|E9AK24_LEIMU Uncharacterized protein OS=Leishm	8
5.698588913	2.932472	E9AQY8	tr|E9AQY8|E9AQY8_LEIMU Uncharacterized protein OS=Leishn	4
5.468555416	2.929178	E9AW99	tr|E9AW99|E9AW99_LEIMU Uncharacterized protein OS=Leish	2
4.363829786	2.925803	E9AVW4	tr|E9AVW4|E9AVW4_LEIMU Uncharacterized protein OS=Leish	6
4.548047944	2.924732	E9ALS6	tr|E9ALS6|E9ALS6_LEIMU Uncharacterized protein OS=Leishma	7
4.849703064	2.919084	E9AP27	tr|E9AP27|E9AP27_LEIMU Arginine N-methyltransferase-like p	3
4.150424412	2.917714	E9AVX8	tr|E9AVX8|E9AVX8_LEIMU I/6 autoantigen-like protein OS=Leis	6
4.247234142	2.895426	E9ATV9	tr|E9ATV9|E9ATV9_LEIMU Uncharacterized protein OS=Leishm	2
4.428054926	2.881882	E9B0K3	tr|E9B0K3|E9B0K3_LEIMU Uncharacterized protein OS=Leishm	3
5.567739738	2.875933	E9B2D5	tr|E9B2D5|E9B2D5_LEIMU Uncharacterized protein OS=Leishm	3
4.544979092	2.854872	E9ALP4	tr|E9ALP4|E9ALP4_LEIMU Putative 40S ribosomal protein S15A	4
4.599908804	2.853579	E9AL32	tr|E9AL32|E9AL32_LEIMU Uncharacterized protein OS=Leishma	4
4.627462554	2.811471	E9AY66;Q86	tr|E9AY66|E9AY66_LEIMU Putative 40S ribosomal protein S16	9
4.51252764	2.807166	E9AJ92	tr|E9AJ92|E9AJ92_LEIMU Uncharacterized protein OS=Leishma	3
4.505070979	2.802599	E9AYS6	tr|E9AYS6|E9AYS6_LEIMU Kinetoplast-associated protein-like p	2
5.449521223	2.771585	E9AS32	tr|E9AS32|E9AS32_LEIMU Putative ABC transporter OS=Leishm	7
5.169508245	2.771491	E9B4W0	tr|E9B4W0|E9B4W0_LEIMU Uncharacterized protein OS=Leish	4
4.248749993	2.749655	E9B2Z3	tr|E9B2Z3|E9B2Z3_LEIMU Uncharacterized protein OS=Leishm	2
4.890192674	2.722991	E9ATF6;E9A	tr|E9ATF6|E9ATF6_LEIMU Putative 60S ribosomal protein L34 (	6
4.649838528	2.674762	E9AM51	tr|E9AM51|E9AM51_LEIMU Uncharacterized protein OS=Leish	2
4.999453024	2.663591	E9AS55	tr|E9AS55|E9AS55_LEIMU Putative phenylalanyl-tRNA syntheta	2
5.175178061	2.630855	E9AQU8	tr|E9AQU8|E9AQU8_LEIMU Uncharacterized protein OS=Leishi	2
4.792658665	2.629485	E9ANM7	tr|E9ANM7|E9ANM7_LEIMU Uncharacterized protein OS=Leish	2

5.363423966	2.547973	E9AYN3	tr|E9AYN3|E9AYN3_LEIMU Uncharacterized protein OS=Leishn	2
5.229068014	2.501087	E9AWQ4;E9	tr|E9AWQ4|E9AWQ4_LEIMU Kinesin-like protein OS=Leishmar	4
4.840316994	2.440564	Q4KXA6;E9I	tr|Q4KXA6|Q4KXA6_LEIME Cytoplasmic dynein heavy chain 2.1	7
4.499561328	2.429992	E9ARN6	tr|E9ARN6|E9ARN6_LEIMU UTP--glucose-1-phosphate uridylylt	3
4.509381547	2.356462	Q0PKV3;E9/	tr|Q0PKV3|Q0PKV3_LEIME Putative glycogen synthase kinase 3	4
4.442143305	2.32498	E9AMT0	tr|E9AMT0|E9AMT0_LEIMU Uncharacterized protein OS=Leish	2
4.527856296	2.271343	E9AQ88	tr|E9AQ88|E9AQ88_LEIMU Putative ABC transporter OS=Leishı	2
4.573868156	2.126379	E9B2X8	tr|E9B2X8|E9B2X8_LEIMU Uncharacterized protein OS=Leishm	2
4.173792163	2.124404	E9ANN3	tr|E9ANN3|E9ANN3_LEIMU Uncharacterized protein OS=Leishı	4
5.303734683	2.117087	E9AP91	tr|E9AP91|E9AP91_LEIMU Putative lectin OS=Leishmania mexi	2
4.490107836	1.952153	E9B0E9	tr|E9B0E9|E9B0E9_LEIMU Putative ubiquitin hydrolase OS=Leis	2

Table S2. RBPs in *L. mexicana* axenic amastigotes

[-Log(P-value)]	log2 FC	Protein IDs	Fasta headers	Unique peptides
4.129283892	4.4868	E9B4D7	tr|E9B4D7|E9B4D7_LEIMU Uncharacterized protein (	2
4.867424935	3.6974	E9AN57;E9A	tr|E9AN57|E9AN57_LEIMU GP63, leishmanolysin OS=	4
4.769553812	3.6853	E9B600	tr|E9B600|E9B600_LEIMU Uncharacterized protein C	2
5.136589823	3.6298	E9AM02	tr|E9AM02|E9AM02_LEIMU Putative lipophosphogly	11
4.607237662	3.4678	E9AJC9	tr|E9AJC9|E9AJC9_LEIMU 40S ribosomal protein S7 C	7
4.964889836	3.2813	E9ASR3	tr|E9ASR3|E9ASR3_LEIMU Putative translation elong	4
4.184398215	3.2614	E9AP91	tr|E9AP91|E9AP91_LEIMU Putative lectin OS=Leishm	2
4.397830244	3.2327	E9B350;E9B	tr|E9B350|E9B350_LEIMU Putative ribosomal protein	5
4.594648105	3.2096	E9AL18	tr|E9AL18|E9AL18_LEIMU Putative Golgi/lysosome gl	2
4.434616824	3.1653	E9B078	tr|E9B078|E9B078_LEIMU Putative 40S ribosomal pro	4
4.270588588	3.1577	E9AWA7;Q9	tr|E9AWA7|E9AWA7_LEIMU Cytosolic leucyl aminop	2
4.649272969	3.132	E9AXU1	tr|E9AXU1|E9AXU1_LEIMU Uncharacterized protein (	2
4.659491177	3.109	E9ARJ2	tr|E9ARJ2|E9ARJ2_LEIMU Uncharacterized protein OS	2
4.626068418	3.0983	E9AW91;Q5	tr|E9AW91|E9AW91_LEIMU Putative mitochondrial F	5
4.446869606	3.0684	E9B714	tr|E9B714|E9B714_LEIMU Uncharacterized protein C	2
4.849581393	3.0605	E9B4Q3;E9B	tr|E9B4Q3|E9B4Q3_LEIMU Elongation factor 1-beta (	6
4.354295499	2.9856	O46368;E9B	tr|O46368|O46368_LEIME Surface protein amastin O	2
4.992450042	2.9713	E9AKP0	tr|E9AKP0|E9AKP0_LEIMU Peptidyl-prolyl cis-trans is	4
4.80810464	2.9711	E9AK98	tr|E9AK98|E9AK98_LEIMU Putative RNA-binding prot	3
4.73356256	2.9278	E9AY45;A7Y	tr|E9AY45|E9AY45_LEIMU Uncharacterized protein C	11
4.550747248	2.8976	E9AR33;E9A	tr|E9AR33|E9AR33_LEIMU Uncharacterized protein (	2
4.290695534	2.8846	E9AQ05	tr|E9AQ05|E9AQ05_LEIMU Uncharacterized protein (	3
4.620602607	2.8712	E9B170;Q27	tr|E9B170|E9B170_LEIMU Glyceraldehyde-3-phospha	15
4.869830786	2.8709	E9B5N7	tr|E9B5N7|E9B5N7_LEIMU Uncharacterized protein (	3
5.290132585	2.8536	E9B189	tr|E9B189|E9B189_LEIMU Uncharacterized protein C	3
4.920548023	2.8314	E9AM03	tr|E9AM03|E9AM03_LEIMU Putative U3 small nuclec	4
4.768822084	2.8187	E9AZT9	tr|E9AZT9|E9AZT9_LEIMU Putative glucose-regulatec	24
3.821320124	2.8112	E9AW79	tr|E9AW79|E9AW79_LEIMU Uncharacterized protein	6
4.498217421	2.809	E9AQY8	tr|E9AQY8|E9AQY8_LEIMU Uncharacterized protein (	5
4.422706147	2.8048	E9AYU4	tr|E9AYU4|E9AYU4_LEIMU Ribokinase OS=Leishmani	2
5.08943538	2.7902	E9AUB1	tr|E9AUB1|E9AUB1_LEIMU Uncharacterized protein (	11
4.582388089	2.7804	E9B1V7	tr|E9B1V7|E9B1V7_LEIMU Putative cytochrome c oxi	2
4.271056255	2.7403	E9AUZ2;E9A	tr|E9AUZ2|E9AUZ2_LEIMU Phosphotransferase OS=L	20
5.413434965	2.7362	E9AT36	tr|E9AT36|E9AT36_LEIMU Putative membrane-bound	3
4.639027831	2.7345	E9AR51	tr|E9AR51|E9AR51_LEIMU Ysine decarboxylase-like p	6
4.541879368	2.725	E9AVK9	tr|E9AVK9|E9AVK9_LEIMU Uncharacterized protein (	3
5.119057263	2.7178	E9ART5	tr|E9ART5|E9ART5_LEIMU Putative P-type H+-ATPase	2
4.16506619	2.7145	E9AWI3	tr|E9AWI3|E9AWI3_LEIMU V-type proton ATPase sub	6
4.450159833	2.7122	E9B5Z0	tr|E9B5Z0|E9B5Z0_LEIMU Uncharacterized protein O	3
4.280164332	2.7102	E9AWP2	tr|E9AWP2|E9AWP2_LEIMU Putative DNAJ domain p	2
4.291790976	2.6972	E9B716	tr|E9B716|E9B716_LEIMU Uncharacterized protein C	3
3.597875693	2.6883	E9B548	tr|E9B548|E9B548_LEIMU Uncharacterized protein C	3
3.985650553	2.6824	E9AQA9	tr|E9AQA9|E9AQA9_LEIMU Uncharacterized protein	4
4.383514431	2.6822	E9AXI5	tr|E9AXI5|E9AXI5_LEIMU Uncharacterized protein OS	2
4.170254393	2.6742	E9AMV6	tr|E9AMV6|E9AMV6_LEIMU Phospholipid:diacylglyce	3
4.499118852	2.6685	E9AU51	tr|E9AU51|E9AU51_LEIMU Uncharacterized protein (	2

4.51463173	2.6669	E9AS79	tr|E9AS79|E9AS79_LEIMU Uncharacterized protein O	2
4.980552148	2.6562	E9AK97	tr|E9AK97|E9AK97_LEIMU Putative fructose-1,6-bisp	3
4.376754374	2.6287	E9B5I2	tr|E9B5I2|E9B5I2_LEIMU Uncharacterized protein OS	2
4.511353054	2.6193	E9AS47	tr|E9AS47|E9AS47_LEIMU Peptidylprolyl isomerase C	2
4.663968302	2.6181	E9AKL0;Q86	tr|E9AKL0|E9AKL0_LEIMU Stomatin-like protein OS=L	10
3.761135618	2.6131	E9B5Z2;E9B	tr|E9B5Z2|E9B5Z2_LEIMU Oligosaccharyl transferase	6
4.851203164	2.607	E9AU66	tr|E9AU66|E9AU66_LEIMU Serine/threonine-protein	5
3.88667969	2.6024	E9AJK8	tr|E9AJK8|E9AJK8_LEIMU Uncharacterized protein OS	8
4.467553438	2.5949	E9ARW0	tr|E9ARW0|E9ARW0_LEIMU Uncharacterized protein	5
4.75346917	2.577	E9AZ96	tr|E9AZ96|E9AZ96_LEIMU Uncharacterized protein O	2
4.003731693	2.5644	E9ANW2	tr|E9ANW2|E9ANW2_LEIMU Uncharacterized protein	2
3.824511047	2.5632	E9B2V9;E9B	tr|E9B2V9|E9B2V9_LEIMU Superoxide dismutase OS=	2
3.232529957	2.5631	E9AUI1;E9A	tr|E9AUI1|E9AUI1_LEIMU Uncharacterized protein O	2
4.047066395	2.561	E9ALR2	tr|E9ALR2|E9ALR2_LEIMU Transmembrane 9 superfa	2
4.156283175	2.5598	E9AJM9	tr|E9AJM9|E9AJM9_LEIMU Putative mitochondrial ca	2
3.884414239	2.5583	E9AY83	tr|E9AY83|E9AY83_LEIMU Uncharacterized protein O	7
4.699770496	2.5409	E9AXI9	tr|E9AXI9|E9AXI9_LEIMU DNAj-like protein OS=Leishı	5
4.021328138	2.5358	E9ALS3;E8N	tr|E9ALS3|E9ALS3_LEIMU Uncharacterized protein O	2
5.375954618	2.5182	E9B560	tr|E9B560|E9B560_LEIMU Uncharacterized protein O	4
4.951062372	2.5142	E9ASU7	tr|E9ASU7|E9ASU7_LEIMU Uncharacterized protein O	3
3.881372059	2.503	E9AUJ5	tr|E9AUJ5|E9AUJ5_LEIMU Uncharacterized protein O	2
4.204360405	2.4692	E9B1S0	tr|E9B1S0|E9B1S0_LEIMU Putative vacuolar-type pro	12
4.300514304	2.4653	E9B612	tr|E9B612|E9B612_LEIMU Uncharacterized protein O	8
3.836603209	2.4545	E9B5E6	tr|E9B5E6|E9B5E6_LEIMU Uncharacterized protein O	2
4.459116643	2.4387	E9ASZ2	tr|E9ASZ2|E9ASZ2_LEIMU Putative COP-coated vesicl	2
4.47041267	2.437	E9B632	tr|E9B632|E9B632_LEIMU Putative reiske iron-sulfur	5
3.750183079	2.4337	E9ALD0	tr|E9ALD0|E9ALD0_LEIMU Uncharacterized protein O	2
4.279177508	2.4264	E9B2C7	tr|E9B2C7|E9B2C7_LEIMU Putative syntaxin 5 OS=Lei	2
3.606438935	2.4252	E9B259	tr|E9B259|E9B259_LEIMU Putative calreticulin OS=Le	4
3.299344148	2.4249	E9AR71	tr|E9AR71|E9AR71_LEIMU Uncharacterized protein O	2
4.472024826	2.4227	E9ARV5;E9A	tr|E9ARV5|E9ARV5_LEIMU Histone H2B OS=Leishmai	2
4.523601779	2.4216	E9AUD1;A7Y	tr|E9AUD1|E9AUD1_LEIMU Protein disulfide-isomera	19
4.577975306	2.4198	E9B3B4	tr|E9B3B4|E9B3B4_LEIMU Dihydrolipoyl dehydrogen	8
4.225107992	2.3995	E9ALL2	tr|E9ALL2|E9ALL2_LEIMU Uncharacterized protein OS	9
4.158901016	2.3958	E9AZI2	tr|E9AZI2|E9AZI2_LEIMU Histone H2B OS=Leishmania	2
4.281036315	2.3947	E9B700	tr|E9B700|E9B700_LEIMU Uncharacterized protein O	11
4.258216073	2.3946	E9AXJ2	tr|E9AXJ2|E9AXJ2_LEIMU Putative cytochrome c oxid	2
4.091593027	2.3894	E9ALH3	tr|E9ALH3|E9ALH3_LEIMU ATP-dependent 6-phosph	13
4.108036993	2.3886	E9ANS8	tr|E9ANS8|E9ANS8_LEIMU Proteasome subunit beta	2
4.734206294	2.3864	E9AR37;E9A	tr|E9AR37|E9AR37_LEIMU Uncharacterized protein O	2
3.109355858	2.3749	E9AP45	tr|E9AP45|E9AP45_LEIMU SURF1-like protein OS=Lei	2
2.72099939	2.3744	E9APE1	tr|E9APE1|E9APE1_LEIMU Uncharacterized protein O	2
3.834271451	2.3675	E9AYS3	tr|E9AYS3|E9AYS3_LEIMU Uncharacterized protein O	2
3.914394171	2.3645	E9AX53	tr|E9AX53|E9AX53_LEIMU 40S ribosomal protein S8 O	6
4.102105235	2.362	E9B047	tr|E9B047|E9B047_LEIMU Putative glycosomal meml	5
4.699031338	2.3611	E9AKY4;A7Y	tr|E9AKY4|E9AKY4_LEIMU Protein disulfide isomeras	6
4.31503506	2.3527	E9B158	tr|E9B158|E9B158_LEIMU Uncharacterized protein O	2
4.073984301	2.3507	E9ARR3;E9A	tr|E9ARR3|E9ARR3_LEIMU Uncharacterized protein O	3
5.035155089	2.3461	E9ALB7	tr|E9ALB7|E9ALB7_LEIMU RNA binding protein-like p	2
3.857571748	2.3408	E9B5F7	tr|E9B5F7|E9B5F7_LEIMU Transmembrane 9 superfa	4

2.865893882	2.3325	E9AN91	tr|E9AN91|E9AN91_LEIMU Uncharacterized protein (	2
4.544497557	2.33	E9APH2	tr|E9APH2|E9APH2_LEIMU Putative Ran-binding prot	3
4.299544501	2.3287	E9B0T3	tr|E9B0T3|E9B0T3_LEIMU Uncharacterized protein O	2
4.595449214	2.3243	E9AVK2	tr|E9AVK2|E9AVK2_LEIMU Uncharacterized protein (	2
2.780529322	2.32	E9AMB3	tr|E9AMB3|E9AMB3_LEIMU Uncharacterized protein	2
4.190592091	2.3177	E9APY1	tr|E9APY1|E9APY1_LEIMU Uncharacterized protein C	2
3.999283679	2.3169	E9B3W5	tr|E9B3W5|E9B3W5_LEIMU Putative ABC transporte	2
4.808393556	2.3142	E9B2E8	tr|E9B2E8|E9B2E8_LEIMU Uncharacterized protein O	10
4.571943547	2.3116	E9B506;A7Y	tr|E9B506|E9B506_LEIMU Uncharacterized protein C	2
3.92918048	2.3088	E9AK24	tr|E9AK24|E9AK24_LEIMU Uncharacterized protein (	3
3.943984992	2.3063	E9B4R2	tr|E9B4R2|E9B4R2_LEIMU Uncharacterized protein C	2
4.131502326	2.3033	E9B428	tr|E9B428|E9B428_LEIMU Uncharacterized protein C	2
3.102496205	2.3022	E9B065	tr|E9B065|E9B065_LEIMU Uncharacterized protein C	3
4.215895326	2.2998	E9AJK7	tr|E9AJK7|E9AJK7_LEIMU Uncharacterized protein OS	9
3.895416321	2.2988	E9ATF6;E9A	tr|E9ATF6|E9ATF6_LEIMU Putative 60S ribosomal pro	2
4.57253755	2.2958	E9B4N4	tr|E9B4N4|E9B4N4_LEIMU Proteasome regulatory no	2
4.356931633	2.2921	E9AMG3	tr|E9AMG3|E9AMG3_LEIMU DNA polymerase OS=Le	2
3.914653079	2.2918	E9B466	tr|E9B466|E9B466_LEIMU Uncharacterized protein C	2
3.946613935	2.2836	E9API4	tr|E9API4|E9API4_LEIMU Uncharacterized protein OS	2
3.276929454	2.2831	E9B444	tr|E9B444|E9B444_LEIMU Uncharacterized protein C	4
4.345817472	2.2797	E9AVH9	tr|E9AVH9|E9AVH9_LEIMU Proteasome subunit alph	3
3.829987167	2.2776	E9B4B3	tr|E9B4B3|E9B4B3_LEIMU Uncharacterized protein C	5
3.42388994	2.2736	E9B2Y0	tr|E9B2Y0|E9B2Y0_LEIMU Uncharacterized protein O	2
4.282070247	2.2718	E9AML1	tr|E9AML1|E9AML1_LEIMU Uncharacterized protein	6
2.601644272	2.2702	E9ASV7	tr|E9ASV7|E9ASV7_LEIMU Putative DEAD box RNA he	2
4.013246028	2.2606	E9AL77	tr|E9AL77|E9AL77_LEIMU Uncharacterized protein O	5
2.843807202	2.254	E9AKA5	tr|E9AKA5|E9AKA5_LEIMU Uncharacterized protein (	3
4.60201836	2.2416	E9AQE3	tr|E9AQE3|E9AQE3_LEIMU Putative ribosomal protei	2
3.381579468	2.2354	E9APS4	tr|E9APS4|E9APS4_LEIMU Uncharacterized protein C	2
4.156463391	2.2302	E9B3B3	tr|E9B3B3|E9B3B3_LEIMU Putative chaperone protei	2
3.691949672	2.23	E9AXQ9	tr|E9AXQ9|E9AXQ9_LEIMU Putative 3-oxo-5-alpha-st	6
4.37610563	2.226	E9ALL3	tr|E9ALL3|E9ALL3_LEIMU Uncharacterized protein OS	4
4.008617839	2.2207	E9B4H8	tr|E9B4H8|E9B4H8_LEIMU Putative adenylate kinase	6
4.070745688	2.2164	E9APM2	tr|E9APM2|E9APM2_LEIMU Uncharacterized protein	2
3.897797181	2.2135	E9B5N9	tr|E9B5N9|E9B5N9_LEIMU Uncharacterized protein (	8
4.133660863	2.2134	E9AVH4	tr|E9AVH4|E9AVH4_LEIMU 40S ribosomal protein S6	5
4.114599306	2.2094	E9B226	tr|E9B226|E9B226_LEIMU Putative 3,2-trans-enoyl-C	5
4.28788069	2.2093	E9AVL4	tr|E9AVL4|E9AVL4_LEIMU Uncharacterized protein C	12
4.096170899	2.2034	E9B4K6	tr|E9B4K6|E9B4K6_LEIMU Uncharacterized protein (I	2
3.628907029	2.1992	E9AN97;Q9I	tr|E9AN97|E9AN97_LEIMU Histone H3 OS=Leishman	4
4.090034138	2.1976	E9B630	tr|E9B630|E9B630_LEIMU Uncharacterized protein C	3
3.38587833	2.1932	E9AT12;Q01	tr|E9AT12|E9AT12_LEIMU Glyceraldehyde-3-phospha	3
3.806243243	2.1927	E9AYB0	tr|E9AYB0|E9AYB0_LEIMU Uncharacterized protein C	3
4.492908888	2.1909	E9ALJ3	tr|E9ALJ3|E9ALJ3_LEIMU Enoyl-CoA hydratase/isome	2
3.238789856	2.1829	E9ATT0	tr|E9ATT0|E9ATT0_LEIMU Uncharacterized protein C	3
4.031439785	2.1829	E9B126	tr|E9B126|E9B126_LEIMU Uncharacterized protein C	2
4.436419222	2.1802	E9AKM1	tr|E9AKM1|E9AKM1_LEIMU V-type proton ATPase su	2
3.99554468	2.1789	E9AKL1	tr|E9AKL1|E9AKL1_LEIMU Uncharacterized protein O	3
4.844753362	2.178	E9AUR9	tr|E9AUR9|E9AUR9_LEIMU Uncharacterized protein (	2
3.75747327	2.1766	E9AKU9	tr|E9AKU9|E9AKU9_LEIMU Uncharacterized protein (	6

3.998109209	2.1758	E9AQW6	tr|E9AQW6|E9AQW6_LEIMU Putative endoplasmic r	2
2.816064869	2.1755	E9ARR2	tr|E9ARR2|E9ARR2_LEIMU Uncharacterized protein C	2
4.128742675	2.1751	E9B5K8	tr|E9B5K8|E9B5K8_LEIMU Uncharacterized protein C	7
3.996561301	2.1732	E9ANC1	tr|E9ANC1|E9ANC1_LEIMU Uncharacterized protein	2
3.851506975	2.1719	E9AVE9	tr|E9AVE9|E9AVE9_LEIMU Uncharacterized protein C	2
3.499149844	2.1697	E9AQK4	tr|E9AQK4|E9AQK4_LEIMU Putative 60S ribosomal p	5
4.167517234	2.1685	E9AWT2	tr|E9AWT2|E9AWT2_LEIMU DNA helicase OS=Leishm	3
3.795604188	2.1665	E9AU24	tr|E9AU24|E9AU24_LEIMU Uncharacterized protein	7
3.713701782	2.1606	E9ATC9	tr|E9ATC9|E9ATC9_LEIMU Uncharacterized protein C	2
4.454048994	2.1577	E9AW08	tr|E9AW08|E9AW08_LEIMU Uncharacterized protein	10
3.818318777	2.1554	E9B6X6	tr|E9B6X6|E9B6X6_LEIMU Uncharacterized protein C	2
4.264262498	2.1536	E9ANG0	tr|E9ANG0|E9ANG0_LEIMU Uncharacterized protein	3
3.628248076	2.1532	E9ALP0	tr|E9ALP0|E9ALP0_LEIMU Uncharacterized protein O	3
4.256730281	2.1523	E9AKF5	tr|E9AKF5|E9AKF5_LEIMU Uncharacterized protein C	5
3.929262141	2.1494	E9B665	tr|E9B665|E9B665_LEIMU Putative 60S ribosomal pr	5
3.957653074	2.1481	E9B3A9	tr|E9B3A9|E9B3A9_LEIMU Glucosamine-6-phosphate	2
4.232415818	2.1406	E9AWU9	tr|E9AWU9|E9AWU9_LEIMU Putative DNAJ domain p	2
4.174776079	2.1369	E9AUE8	tr|E9AUE8|E9AUE8_LEIMU Uncharacterized protein	3
4.131583488	2.1355	E9AS15	tr|E9AS15|E9AS15_LEIMU Putative histone H3 varian	2
4.34250683	2.1333	V5RIA5;E9A	tr|V5RIA5|V5RIA5_LEIAM Calmodulin-like protein OS	2
3.84328802	2.1315	E9B617	tr|E9B617|E9B617_LEIMU Mitochondrial processing	11
2.433476208	2.1286	E9ASP4	tr|E9ASP4|E9ASP4_LEIMU Uncharacterized protein C	2
4.627463113	2.128	E9AL16	tr|E9AL16|E9AL16_LEIMU Putative cytochrome c1, h	4
3.827506172	2.1271	E9AWS6;A0	tr|E9AWS6|E9AWS6_LEIMU Triosephosphate isomer	2
4.631641669	2.1245	E9ASS9	tr|E9ASS9|E9ASS9_LEIMU Uncharacterized protein O	2
3.688647562	2.1222	E9AP80;E9A	tr|E9AP80|E9AP80_LEIMU Putative 60S ribosomal pr	2
4.32471427	2.1219	E9AX30	tr|E9AX30|E9AX30_LEIMU Hypothetical predicted m	3
3.41376112	2.1207	E9AYE1	tr|E9AYE1|E9AYE1_LEIMU Phosphatidate cytidylyltra	2
4.325498844	2.1201	E9ARZ1	tr|E9ARZ1|E9ARZ1_LEIMU Putative 40S ribosomal pr	3
3.722933508	2.1171	E9AUC8	tr|E9AUC8|E9AUC8_LEIMU Uncharacterized protein	4
3.646873293	2.1131	E9AQD7	tr|E9AQD7|E9AQD7_LEIMU Putative mismatch repai	3
4.121833834	2.1121	E9AXM2	tr|E9AXM2|E9AXM2_LEIMU Uncharacterized protein	2
3.037657575	2.1109	E9B2V2	tr|E9B2V2|E9B2V2_LEIMU Uncharacterized protein C	4
4.198362774	2.1099	E9ASB9	tr|E9ASB9|E9ASB9_LEIMU Histone H4 OS=Leishmani	3
4.12575727	2.1043	E9B649	tr|E9B649|E9B649_LEIMU Uncharacterized protein C	2
2.810970709	2.1025	E9ANM2;E9	tr|E9ANM2|E9ANM2_LEIMU Putative 40S ribosomal	2
3.865177739	2.1002	E9B6Q4	tr|E9B6Q4|E9B6Q4_LEIMU Putative 60S ribosomal p	3
4.553048686	2.0982	E9AY47	tr|E9AY47|E9AY47_LEIMU Uncharacterized protein C	2
3.982956974	2.0943	E9AYW6	tr|E9AYW6|E9AYW6_LEIMU Uncharacterized protein	3
3.144320955	2.0939	E9B263	tr|E9B263|E9B263_LEIMU 2-oxoglutarate dehydroge	3
3.678995228	2.0939	E9AQK9	tr|E9AQK9|E9AQK9_LEIMU Uncharacterized protein	2
4.950722902	2.0936	E9ALJ0	tr|E9ALJ0|E9ALJ0_LEIMU Uncharacterized protein OS	2
3.839562558	2.092	E9AR74	tr|E9AR74|E9AR74_LEIMU Uncharacterized protein C	5
3.56386285	2.0919	E9AN94	tr|E9AN94|E9AN94_LEIMU Uncharacterized protein	2
3.52647071	2.0918	E9APS3	tr|E9APS3|E9APS3_LEIMU Uncharacterized protein C	3
3.704610099	2.0918	E9ALL1	tr|E9ALL1|E9ALL1_LEIMU Uncharacterized protein OS	2
3.84353383	2.0895	E9ASN7	tr|E9ASN7|E9ASN7_LEIMU Uncharacterized protein	4
3.987546439	2.0885	E9AMK8	tr|E9AMK8|E9AMK8_LEIMU Putative RNA helicase OS	5
4.291995229	2.0847	E9AJT5	tr|E9AJT5|E9AJT5_LEIMU Uncharacterized protein OS	5
2.800227596	2.0843	E9AR16	tr|E9AR16|E9AR16_LEIMU Uncharacterized protein C	2

4.44944402	2.0798	E9AJU4	tr|E9AJU4|E9AJU4_LEIMU Kinase-like protein OS=Lei:	2
3.832819516	2.0777	E9B6Y0	tr|E9B6Y0|E9B6Y0_LEIMU Uncharacterized protein O	3
3.296319082	2.0704	E9AKC2	tr|E9AKC2|E9AKC2_LEIMU Dihydrolipoamide acetyltr	4
4.001846453	2.0695	Q867J9;E9A	tr|Q867J9|Q867J9_LEIME Histone H2A OS=Leishman	2
4.51256507	2.0689	E9APV6	tr|E9APV6|E9APV6_LEIMU Putative kinesin K39 OS=L	5
3.989325665	2.0684	E9AVG7	tr|E9AVG7|E9AVG7_LEIMU Putative cytochrome c ox	7
3.220781209	2.0644	E9B602	tr|E9B602|E9B602_LEIMU Putative short chain dehyc	3
4.016250044	2.0643	E9AM47	tr|E9AM47|E9AM47_LEIMU Uncharacterized protein	4
4.514576459	2.0636	E9APB2	tr|E9APB2|E9APB2_LEIMU Uncharacterized protein (	2
3.91699857	2.0635	E9AT08	tr|E9AT08|E9AT08_LEIMU Uncharacterized protein C	2
3.931560641	2.0632	E9AKR8	tr|E9AKR8|E9AKR8_LEIMU Putative 60S ribosomal pr	2
3.805733545	2.0629	E9AYQ6	tr|E9AYQ6|E9AYQ6_LEIMU CAAX prenyl protease OS:	4
4.096309185	2.0627	E9AL39	tr|E9AL39|E9AL39_LEIMU Uncharacterized protein O	3
5.001073779	2.0624	E9ATE1	tr|E9ATE1|E9ATE1_LEIMU Uncharacterized protein C	3
4.469088794	2.06	E9ART6	tr|E9ART6|E9ART6_LEIMU Plasma membrane ATPase	7
3.948021999	2.0586	E9B6X2	tr|E9B6X2|E9B6X2_LEIMU Putative mitochondrial ph	6
4.413228259	2.0558	E9ARF8	tr|E9ARF8|E9ARF8_LEIMU Uncharacterized protein C	9
4.044783833	2.0532	E9AJY0	tr|E9AJY0|E9AJY0_LEIMU Uncharacterized protein OS	3
4.203995033	2.0519	E9AVP7	tr|E9AVP7|E9AVP7_LEIMU Uncharacterized protein (	4
3.217573343	2.0517	E9B0Y3	tr|E9B0Y3|E9B0Y3_LEIMU Uncharacterized protein O	5
3.780525796	2.0503	E9AZ76	tr|E9AZ76|E9AZ76_LEIMU Uncharacterized protein C	2
3.614258219	2.0493	E9B4C7	tr|E9B4C7|E9B4C7_LEIMU Uncharacterized protein C	2
3.40837376	2.0474	E9AL69	tr|E9AL69|E9AL69_LEIMU Uncharacterized protein O	3
3.611987864	2.0465	E9AKR1	tr|E9AKR1|E9AKR1_LEIMU Uncharacterized protein (	2
4.664581793	2.0464	E9B0M2	tr|E9B0M2|E9B0M2_LEIMU Uncharacterized protein	2
3.161131598	2.046	E8NHJ5;E9A	tr|E8NHJ5|E8NHJ5_LEIMU WGS CADB00000000 data	8
3.567617274	2.0448	E9B304	tr|E9B304|E9B304_LEIMU RNA helicase OS=Leishmai	3
4.011371587	2.0446	E9AVW4	tr|E9AVW4|E9AVW4_LEIMU Uncharacterized proteir	12
3.229314466	2.0425	E9B127	tr|E9B127|E9B127_LEIMU Uncharacterized protein C	2
3.476885954	2.0425	E9AXA9	tr|E9AXA9|E9AXA9_LEIMU Uncharacterized protein (	2
4.19375771	2.0422	E9APT4	tr|E9APT4|E9APT4_LEIMU Uncharacterized protein C	2
3.990358644	2.0414	E9APM4	tr|E9APM4|E9APM4_LEIMU Proteasome subunit alpl	2
4.105219775	2.0399	E9AQY7	tr|E9AQY7|E9AQY7_LEIMU Uncharacterized protein (	6
4.110552588	2.039	E9AK06	tr|E9AK06|E9AK06_LEIMU Uncharacterized protein C	2
3.866992602	2.0362	E9B6G8	tr|E9B6G8|E9B6G8_LEIMU Uncharacterized protein (	3
4.184908937	2.0351	E9AKX9	tr|E9AKX9|E9AKX9_LEIMU Uncharacterized protein C	2
4.353075534	2.0336	E9B376;A0A	tr|E9B376|E9B376_LEIMU Nucleoside diphosphate ki	5
2.824660203	2.0328	E9AUZ6	tr|E9AUZ6|E9AUZ6_LEIMU Uncharacterized protein (	2
3.999944024	2.0315	E9B0N9	tr|E9B0N9|E9B0N9_LEIMU Putative DREV methyltrar	2
3.571762582	2.0294	E9ANW6	tr|E9ANW6|E9ANW6_LEIMU Uncharacterized proteir	2
4.233999471	2.029	E9B2E1	tr|E9B2E1|E9B2E1_LEIMU Uncharacterized protein O	2
3.86772471	2.029	E9B0I4	tr|E9B0I4|E9B0I4_LEIMU Uncharacterized protein OS	2
3.607624609	2.0287	E9ANG4	tr|E9ANG4|E9ANG4_LEIMU Uncharacterized protein	2
4.338775578	2.0221	E9AQ06	tr|E9AQ06|E9AQ06_LEIMU Heat shock protein HslVU	9
4.148760223	2.0217	E9ATD2;E9B	tr|E9ATD2|E9ATD2_LEIMU Putative polyubiquitin (Fr	2
3.909441145	2.0203	E9AW66	tr|E9AW66|E9AW66_LEIMU Uncharacterized protein	2
4.152715891	2.0191	E9B5K2	tr|E9B5K2|E9B5K2_LEIMU Uncharacterized protein C	5
3.571432461	2.019	E9AQR6	tr|E9AQR6|E9AQR6_LEIMU Uncharacterized protein	2
4.003705417	2.0183	E9ASL7	tr|E9ASL7|E9ASL7_LEIMU Uncharacterized protein O	2
4.441571883	2.0166	E9B370	tr|E9B370|E9B370_LEIMU Uncharacterized protein C	2

3.741484283	2.0157	E9ATG0	tr|E9ATG0|E9ATG0_LEIMU Uncharacterized protein (	2
4.082090313	2.0107	E9B2L1	tr|E9B2L1|E9B2L1_LEIMU Phenylalanyl-tRNA synthet	6
3.797273095	2.0104	E9B429	tr|E9B429|E9B429_LEIMU Uncharacterized protein C	2
4.606777904	2.0088	E9ATU7	tr|E9ATU7|E9ATU7_LEIMU Uncharacterized protein (	3
3.719999843	2.0086	E9ATN6	tr|E9ATN6|E9ATN6_LEIMU Uncharacterized protein (	7
3.693298864	2.008	E9ANM9	tr|E9ANM9|E9ANM9_LEIMU Uncharacterized proteir	4
4.157378496	2.0057	E9B1H3	tr|E9B1H3|E9B1H3_LEIMU Uncharacterized protein (	11
3.935992299	2.005	E9AJR0	tr|E9AJR0|E9AJR0_LEIMU Putative ribosomal protein	3
3.492795064	2.0043	E9AUV2	tr|E9AUV2|E9AUV2_LEIMU Uncharacterized protein	3
3.345361633	2.0042	E9ASK1	tr|E9ASK1|E9ASK1_LEIMU Uncharacterized protein C	4
3.687388643	2.0037	E9AKX0	tr|E9AKX0|E9AKX0_LEIMU Uncharacterized protein C	2
3.053425998	2.0036	E9B4M8	tr|E9B4M8|E9B4M8_LEIMU Uncharacterized protein	2
3.792545682	2.0027	E9B052	tr|E9B052|E9B052_LEIMU Eukaryotic translation initi	11
4.45145847	2.0022	E9B6H7	tr|E9B6H7|E9B6H7_LEIMU Putative chaperone prote	7
4.024819927	2.0015	E9AK81	tr|E9AK81|E9AK81_LEIMU Uncharacterized protein C	2
5.04986212	2.0013	E9APR1	tr|E9APR1|E9APR1_LEIMU Elongation of fatty acids p	2
3.683190177	2.001	E9B2P1	tr|E9B2P1|E9B2P1_LEIMU Elongation of fatty acids p	2
4.12605236	2.0009	E9B027	tr|E9B027|E9B027_LEIMU Threonylcarbamoyl-AMP s	3
4.274744143	1.9986	E9B3A4	tr|E9B3A4|E9B3A4_LEIMU Uncharacterized protein (	3
3.808172651	1.9978	E9AN60	tr|E9AN60|E9AN60_LEIMU Uncharacterized protein (	4
4.076755345	1.9976	E9AV01	tr|E9AV01|E9AV01_LEIMU Mitochondrial processing	13
4.068526693	1.9952	E9B0W0	tr|E9B0W0|E9B0W0_LEIMU Adenylate kinase isoenzy	2
4.615063911	1.995	E9ARE6	tr|E9ARE6|E9ARE6_LEIMU Putative alpha glucosidase	6
3.659895971	1.994	E9B3Z9	tr|E9B3Z9|E9B3Z9_LEIMU Uncharacterized protein O	4
3.864227326	1.9922	E9AVW9	tr|E9AVW9|E9AVW9_LEIMU Intraflagellar transport p	5
4.196271476	1.9921	E9ATV2	tr|E9ATV2|E9ATV2_LEIMU Putative aldehyde dehydr	8
3.46317233	1.9921	E9AKQ0	tr|E9AKQ0|E9AKQ0_LEIMU Uncharacterized protein (	5
3.467075565	1.9886	E9B3Y5	tr|E9B3Y5|E9B3Y5_LEIMU Uncharacterized protein O	3
3.988109703	1.9882	E9B533;E9A	tr|E9B533|E9B533_LEIMU Putative ribosomal proteir	5
3.811144396	1.986	E9AKG6	tr|E9AKG6|E9AKG6_LEIMU Uncharacterized protein (	2
3.466898421	1.9844	E9AKB0	tr|E9AKB0|E9AKB0_LEIMU Putative major vault prote	4
3.48902142	1.9838	E9B2I5	tr|E9B2I5|E9B2I5_LEIMU Uncharacterized protein OS	2
3.750541875	1.9838	E9AQ98	tr|E9AQ98|E9AQ98_LEIMU Putative succinate dehyd	3
3.86492979	1.9837	Q7Z031;E9A	tr|Q7Z031|Q7Z031_LEIAM Acidocalcisomal pyrophos	5
3.996669918	1.9836	E9AV86	tr|E9AV86|E9AV86_LEIMU Putative dynein arm light	4
3.729677873	1.9832	E9AZL7	tr|E9AZL7|E9AZL7_LEIMU Uncharacterized protein O	3
4.128908196	1.9829	E9AJD6	tr|E9AJD6|E9AJD6_LEIMU Putative long-chain-fatty-a	2
3.472056986	1.9829	E9B5G2	tr|E9B5G2|E9B5G2_LEIMU Uncharacterized protein (	2
3.885382464	1.9829	E9AUD3	tr|E9AUD3|E9AUD3_LEIMU Uncharacterized protein	3
3.680041331	1.982	E9AXC1	tr|E9AXC1|E9AXC1_LEIMU Putative transcription fact	2
4.17858307	1.981	E9AYS1	tr|E9AYS1|E9AYS1_LEIMU Proteasome endopeptidas	3
4.581839308	1.9803	E9AQB1	tr|E9AQB1|E9AQB1_LEIMU 60S acidic ribosomal prot	2
3.523367481	1.9785	E9AWY9	tr|E9AWY9|E9AWY9_LEIMU Uncharacterized protein	2
3.483113917	1.9767	E9ALG5	tr|E9ALG5|E9ALG5_LEIMU Uncharacterized protein C	2
4.349734376	1.9757	E9APB1	tr|E9APB1|E9APB1_LEIMU Mitochondrial processing	9
4.19405473	1.9752	E9AW71	tr|E9AW71|E9AW71_LEIMU Kinesin-like protein OS=	4
3.360635127	1.9751	E9AYI3	tr|E9AYI3|E9AYI3_LEIMU Uncharacterized protein OS	2
4.001606945	1.9744	E9AXP1	tr|E9AXP1|E9AXP1_LEIMU Uncharacterized protein C	6
4.069564502	1.9733	E9AKY9	tr|E9AKY9|E9AKY9_LEIMU Uncharacterized protein C	4
4.218390116	1.973	E9B2Q3	tr|E9B2Q3|E9B2Q3_LEIMU Putative RNA polymerase	2

3.852677976	1.9711	E9AWN5;E9	tr|E9AWN5|E9AWN5_LEIMU Uncharacterized protei	4
3.913505807	1.971	E9ANQ3;C7I	tr|E9ANQ3|E9ANQ3_LEIMU Putative lanosterol 14-al	9
3.426989922	1.9703	E9B584	tr|E9B584|E9B584_LEIMU Uncharacterized protein C	4
4.164929346	1.9702	E9AUE0	tr|E9AUE0|E9AUE0_LEIMU DNA-directed RNA polym	3
4.130217693	1.9701	E9AQB0	tr|E9AQB0|E9AQB0_LEIMU Uncharacterized protein	2
3.614521377	1.9699	E9ASJ1	tr|E9ASJ1|E9ASJ1_LEIMU Uncharacterized protein OS	2
2.981775734	1.9695	E9B1J8;E8NI	tr|E9B1J8|E9B1J8_LEIMU Uncharacterized protein OS	2
3.848445878	1.9693	E9B6K4	tr|E9B6K4|E9B6K4_LEIMU Putative replication factor	4
4.351910502	1.9692	E9B334	tr|E9B334|E9B334_LEIMU Putative AMP deaminase (	2
4.068756873	1.9692	E9B443	tr|E9B443|E9B443_LEIMU Uncharacterized protein C	2
3.90778376	1.9673	E9B033	tr|E9B033|E9B033_LEIMU Putative cullin 2 OS=Leishr	2
3.509167772	1.9672	E9B3U1;E9A	tr|E9B3U1|E9B3U1_LEIMU Uncharacterized protein (	7
3.407127398	1.9669	E9AX23	tr|E9AX23|E9AX23_LEIMU Fatty acid transporter pro	3
4.525267726	1.9651	E9ARZ6	tr|E9ARZ6|E9ARZ6_LEIMU Putative nucleosome asse	8
4.115208646	1.965	E9ATB7	tr|E9ATB7|E9ATB7_LEIMU Uncharacterized protein C	3
3.190787311	1.9643	E9B3P3	tr|E9B3P3|E9B3P3_LEIMU Uncharacterized protein C	3
3.159400852	1.9642	E9AV99	tr|E9AV99|E9AV99_LEIMU Uncharacterized protein (	2
3.728619974	1.9642	E9B5H5	tr|E9B5H5|E9B5H5_LEIMU Uncharacterized protein (	2
3.683578381	1.9622	E9ALI5;E9AL	tr|E9ALI5|E9ALI5_LEIMU Putative kinesin OS=Leishm	4
3.86500779	1.9597	E9AT49	tr|E9AT49|E9AT49_LEIMU Putative mitochondrial AT	3
3.919278633	1.9589	E9AWB2	tr|E9AWB2|E9AWB2_LEIMU Uncharacterized protei	3
4.438073276	1.9573	E9AT16;E9A	tr|E9AT16|E9AT16_LEIMU Methyltransferase OS=Lei	10
4.028066583	1.9572	E9AJG4	tr|E9AJG4|E9AJG4_LEIMU Uncharacterized protein O	20
3.987311579	1.957	E9B4H3	tr|E9B4H3|E9B4H3_LEIMU Putative ascorbate-depen	2
3.812711672	1.9567	E9B4M5	tr|E9B4M5|E9B4M5_LEIMU Uncharacterized protein	3
3.718686823	1.9566	E9AZB2	tr|E9AZB2|E9AZB2_LEIMU Uncharacterized protein C	3
4.438517671	1.9566	E9B369	tr|E9B369|E9B369_LEIMU Uncharacterized protein C	2
3.436316905	1.9566	E9B465	tr|E9B465|E9B465_LEIMU Glycosyl hydrolase-like pro	2
3.776895065	1.9565	E9AW42	tr|E9AW42|E9AW42_LEIMU ABC transporter-like pro	3
4.031229284	1.9559	E9AKK2	tr|E9AKK2|E9AKK2_LEIMU Dipeptidyl peptidase 3 OS	4
4.309670069	1.9547	E9ANK4	tr|E9ANK4|E9ANK4_LEIMU 3-methylcrotonoyl-CoA ca	3
3.999507849	1.9542	E9B686	tr|E9B686|E9B686_LEIMU Putative calcium motive P-	6
3.852182854	1.954	E9AMX7	tr|E9AMX7|E9AMX7_LEIMU Uncharacterized protein	2
4.293974621	1.9524	E9AS80	tr|E9AS80|E9AS80_LEIMU Uncharacterized protein C	3
3.685958134	1.9517	E9B041	tr|E9B041|E9B041_LEIMU Uncharacterized protein C	5
3.46068181	1.9516	E9B2B1	tr|E9B2B1|E9B2B1_LEIMU Uncharacterized protein C	5
3.622411695	1.9509	E9ASW0	tr|E9ASW0|E9ASW0_LEIMU Uncharacterized protein	3
4.054963314	1.9501	E9AQA3	tr|E9AQA3|E9AQA3_LEIMU Tryparedoxin peroxidase	2
3.586697844	1.9498	E9B5X9	tr|E9B5X9|E9B5X9_LEIMU Uncharacterized protein C	2
4.372749205	1.9495	E9B5N2	tr|E9B5N2|E9B5N2_LEIMU Cell differentiation protei	7
3.84034216	1.9487	E9AVY4	tr|E9AVY4|E9AVY4_LEIMU Putative 40S ribosomal pr	2
3.313266739	1.9486	E9ATF7	tr|E9ATF7|E9ATF7_LEIMU Putative 40S ribosomal pro	3
4.018545506	1.9471	E9AUV5	tr|E9AUV5|E9AUV5_LEIMU Uncharacterized protein	2
3.786961466	1.9471	E9AYD3	tr|E9AYD3|E9AYD3_LEIMU Uncharacterized protein (	2
3.46978413	1.9458	E9AJN6	tr|E9AJN6|E9AJN6_LEIMU Putative peptidyl dipeptid	3
4.043144701	1.9453	E9AZC8	tr|E9AZC8|E9AZC8_LEIMU Putative ribosomal protei	2
3.682215889	1.9452	E9B5M2	tr|E9B5M2|E9B5M2_LEIMU Defective in cullin neddy	2
4.352069006	1.9448	E9AQI6	tr|E9AQI6|E9AQI6_LEIMU Putative proteasome 26S r	4
3.257653754	1.9438	E9B0U3	tr|E9B0U3|E9B0U3_LEIMU Putative small GTP-bindin	2
3.980581719	1.9436	E9ANY7	tr|E9ANY7|E9ANY7_LEIMU Uncharacterized protein (	2

3.828729688	1.9434	E9AQV3	tr|E9AQV3|E9AQV3_LEIMU Uncharacterized protein	5
3.433563234	1.943	E9B013	tr|E9B013|E9B013_LEIMU Uncharacterized protein C	2
4.27193826	1.9425	E9B4S1;Q9X	tr|E9B4S1|E9B4S1_LEIMU p-glycoprotein OS=Leishma	9
3.772357027	1.9423	E9AKC3	tr|E9AKC3|E9AKC3_LEIMU Uncharacterized protein C	2
4.082258986	1.9421	E9AXP4	tr|E9AXP4|E9AXP4_LEIMU Uncharacterized protein C	3
5.267129153	1.942	E9AP69	tr|E9AP69|E9AP69_LEIMU Uncharacterized protein C	6
5.105429599	1.942	E9AM16	tr|E9AM16|E9AM16_LEIMU Putative ABC transporte	4
4.150193947	1.9415	E9B6R2	tr|E9B6R2|E9B6R2_LEIMU Proteasome subunit beta	2
4.130103383	1.9408	E9AS57	tr|E9AS57|E9AS57_LEIMU Uncharacterized protein C	4
4.140190489	1.9399	E9AV21	tr|E9AV21|E9AV21_LEIMU Putative la RNA binding pr	2
3.645101404	1.9397	E9AJR2	tr|E9AJR2|E9AJR2_LEIMU Uncharacterized protein OS	2
3.647784363	1.9395	E9ANC5	tr|E9ANC5|E9ANC5_LEIMU Uncharacterized protein (	2
4.056111968	1.9392	E9AKL7	tr|E9AKL7|E9AKL7_LEIMU Uncharacterized protein O	2
3.492588964	1.9389	E9B2D4	tr|E9B2D4|E9B2D4_LEIMU Uncharacterized protein C	5
3.502300765	1.9382	E9B1R1	tr|E9B1R1|E9B1R1_LEIMU Putative monoglyceride lip	2
4.261537649	1.938	E9AM67	tr|E9AM67|E9AM67_LEIMU Proteasome regulatory r	13
3.084460071	1.9378	E9B0E3	tr|E9B0E3|E9B0E3_LEIMU Uncharacterized protein O	3
3.610624057	1.9375	E9AWK5	tr|E9AWK5|E9AWK5_LEIMU Uncharacterized protein	2
3.682257499	1.9371	E9AJY9	tr|E9AJY9|E9AJY9_LEIMU Uncharacterized protein OS	2
3.926803904	1.9365	E9B430	tr|E9B430|E9B430_LEIMU Putative phosphoribosylpy	2
3.856495755	1.9351	E9B3J3	tr|E9B3J3|E9B3J3_LEIMU Uncharacterized protein OS	2
4.063784579	1.9338	E9AX57	tr|E9AX57|E9AX57_LEIMU Uncharacterized protein C	5
3.397811346	1.9329	E9B5Y8	tr|E9B5Y8|E9B5Y8_LEIMU Uncharacterized protein O	2
3.777211076	1.9328	E9B099;Q07	tr|E9B099|E9B099_LEIMU Putative heat-shock protei	28
4.383714417	1.9309	E9ASL3	tr|E9ASL3|E9ASL3_LEIMU Putative 40S ribosomal pro	10
3.441000758	1.9305	E9B415	tr|E9B415|E9B415_LEIMU Uncharacterized protein C	3
4.357765589	1.9285	E9AZ71	tr|E9AZ71|E9AZ71_LEIMU Uncharacterized protein C	4
4.156489964	1.9269	E9ATJ9	tr|E9ATJ9|E9ATJ9_LEIMU Uncharacterized protein OS	4
3.939306188	1.9269	E9B0F9	tr|E9B0F9|E9B0F9_LEIMU Kinesin-like protein OS=Lei	4
3.670997013	1.9267	E9AZY3	tr|E9AZY3|E9AZY3_LEIMU Uncharacterized protein O	3
4.345779399	1.9265	E9B3B7	tr|E9B3B7|E9B3B7_LEIMU Uncharacterized protein C	4
3.96120559	1.9256	E9AQU9	tr|E9AQU9|E9AQU9_LEIMU Putative ADP-ribosylatio	4
3.8722981	1.9246	E9B2U9	tr|E9B2U9|E9B2U9_LEIMU Coatomer subunit epsilon	4
5.261912638	1.924	E9B1M5	tr|E9B1M5|E9B1M5_LEIMU Uncharacterized protein	2
4.025435387	1.9233	E9AUX9	tr|E9AUX9|E9AUX9_LEIMU DNA topoisomerase 1A O	2
4.071803825	1.9225	E9APW9	tr|E9APW9|E9APW9_LEIMU Uncharacterized protein	6
3.067424124	1.922	E9AQI7	tr|E9AQI7|E9AQI7_LEIMU Uncharacterized protein O	2
3.909985719	1.9216	E9B1E9	tr|E9B1E9|E9B1E9_LEIMU Putative 5-methyltetrahyd	4
3.981164173	1.9213	E9ALU6	tr|E9ALU6|E9ALU6_LEIMU Putative ATP-dependent C	12
3.689598131	1.921	E9AZ79	tr|E9AZ79|E9AZ79_LEIMU Uncharacterized protein C	2
3.637347532	1.9209	E9AR29	tr|E9AR29|E9AR29_LEIMU Putative P-type ATPase OS	8
3.47368537	1.9208	E9AP56	tr|E9AP56|E9AP56_LEIMU Putative small Rab GTP bir	2
3.987116117	1.9202	E9AK89	tr|E9AK89|E9AK89_LEIMU Uncharacterized protein C	4
4.060525422	1.919	E9AZG1	tr|E9AZG1|E9AZG1_LEIMU Uncharacterized protein C	2
4.202249078	1.9189	E9B5D9	tr|E9B5D9|E9B5D9_LEIMU RuvB-like helicase OS=Leis	13
3.697531825	1.9188	E9AV90;Q86	tr|E9AV90|E9AV90_LEIMU Putative 40S ribosomal pr	4
3.504490599	1.9186	E9AM99;J7H	tr|E9AM99|E9AM99_LEIMU Uncharacterized protein	7
4.046299489	1.9179	E9AZH2	tr|E9AZH2|E9AZH2_LEIMU Proteasome subunit beta	2
3.85823011	1.9177	E9B2D6	tr|E9B2D6|E9B2D6_LEIMU Uncharacterized protein C	2
3.86129737	1.9174	E9AXJ1	tr|E9AXJ1|E9AXJ1_LEIMU Aldehyde dehydrogenase,	11

3.772770989	1.9173	E9AZL6	tr|E9AZL6|E9AZL6_LEIMU DNA repair protein RAD51	2
3.265111672	1.9163	E9AR77	tr|E9AR77|E9AR77_LEIMU Hydrolase-like protein (Es	2
4.207803924	1.9162	E9B3U3	tr|E9B3U3|E9B3U3_LEIMU Putative guanylate kinase	2
3.58213811	1.9157	E9B3Y6	tr|E9B3Y6|E9B3Y6_LEIMU Uncharacterized protein O	3
3.829542811	1.9152	E9ANN1	tr|E9ANN1|E9ANN1_LEIMU Putative kinesin OS=Leisl	2
3.984548023	1.915	E9AUA1;Q8(	tr|E9AUA1|E9AUA1_LEIMU 2,3-bisphosphoglycerate-	8
2.596371758	1.9146	E9ANE2	tr|E9ANE2|E9ANE2_LEIMU Uncharacterized protein (	2
3.04404178	1.9145	E9B479	tr|E9B479|E9B479_LEIMU Putative GTPase protein O	2
3.550860661	1.9144	E9AXP2	tr|E9AXP2|E9AXP2_LEIMU Uncharacterized protein C	2
3.725275479	1.9141	E9B3R5	tr|E9B3R5|E9B3R5_LEIMU Putative 2,4-dienoyl-coa r	2
3.971586103	1.9139	E9AVH3	tr|E9AVH3|E9AVH3_LEIMU Putative ATP synthase F1	5
4.064859546	1.9137	E9ANC9	tr|E9ANC9|E9ANC9_LEIMU Flagellar protofilament ri	2
3.690673443	1.9134	E9ALJ4	tr|E9ALJ4|E9ALJ4_LEIMU Ubiquitinyl hydrolase 1 OS=	2
3.78827272	1.9122	E9ATA7	tr|E9ATA7|E9ATA7_LEIMU Uncharacterized protein (	6
4.421971195	1.9121	E9AYV6	tr|E9AYV6|E9AYV6_LEIMU Putative replication factor	3
3.803840967	1.9113	E9AVE2	tr|E9AVE2|E9AVE2_LEIMU Uncharacterized protein (	12
4.361374733	1.911	E9B6B8	tr|E9B6B8|E9B6B8_LEIMU Uncharacterized protein C	2
4.033834676	1.9103	E9B6G4	tr|E9B6G4|E9B6G4_LEIMU Uncharacterized protein (	3
3.537785663	1.9101	E9ATT5;E9A	tr|E9ATT5|E9ATT5_LEIMU 40S ribosomal protein SA (	12
3.767757696	1.9094	E9B1M1	tr|E9B1M1|E9B1M1_LEIMU Putative ATP-dependent	4
3.121231644	1.9093	E9AS66	tr|E9AS66|E9AS66_LEIMU Uncharacterized protein O	3
4.151645049	1.9088	E9AMW3	tr|E9AMW3|E9AMW3_LEIMU Mitochondrial RNA bir	6
3.892843103	1.908	E9AVS5;E9A	tr|E9AVS5|E9AVS5_LEIMU Uncharacterized protein C	3
3.632353888	1.9073	E9AWR3	tr|E9AWR3|E9AWR3_LEIMU Signal recognition partic	5
3.549865955	1.907	E9B6F6	tr|E9B6F6|E9B6F6_LEIMU Uncharacterized protein O	2
3.76860894	1.9068	E9APL6	tr|E9APL6|E9APL6_LEIMU Uncharacterized protein O	2
3.857070394	1.9068	E9B007	tr|E9B007|E9B007_LEIMU Uncharacterized protein C	5
4.288442706	1.9065	E9B063	tr|E9B063|E9B063_LEIMU Dihydrolipoamide acetyltr	7
3.748565969	1.9062	E9B4N7	tr|E9B4N7|E9B4N7_LEIMU Uncharacterized protein (	3
4.118942215	1.9057	E9AKS5	tr|E9AKS5|E9AKS5_LEIMU ATP-NAD kinase-like prote	2
3.448082608	1.9042	E9ARB0	tr|E9ARB0|E9ARB0_LEIMU Protein SDA1 OS=Leishma	3
3.988557232	1.9041	E9APD0	tr|E9APD0|E9APD0_LEIMU NADH-cytochrome b5 rec	3
3.292335275	1.9037	E9AR26	tr|E9AR26|E9AR26_LEIMU Uncharacterized protein C	2
4.247046019	1.9032	E9AK94	tr|E9AK94|E9AK94_LEIMU Putative cytochrome c oxi	2
3.696330493	1.9031	E9B6H6	tr|E9B6H6|E9B6H6_LEIMU Uncharacterized protein (	3
3.142395685	1.9026	E9B5E1	tr|E9B5E1|E9B5E1_LEIMU Peroxisome biosynthesis p	2
4.119135936	1.9025	E9AXJ6;E9A:	tr|E9AXJ6|E9AXJ6_LEIMU ATP synthase subunit beta	26
4.306264494	1.9016	E9B0Q3	tr|E9B0Q3|E9B0Q3_LEIMU Putative calcium-binding	3
3.902432924	1.9015	E9B258	tr|E9B258|E9B258_LEIMU Uncharacterized protein C	6
3.797462682	1.9005	E9ALW5;E9/	tr|E9ALW5|E9ALW5_LEIMU Putative ribosomal prote	10
4.093107753	1.8998	E9B1E3	tr|E9B1E3|E9B1E3_LEIMU Uncharacterized protein O	8
3.737160465	1.8991	E9AWD5	tr|E9AWD5|E9AWD5_LEIMU Uncharacterized proteir	2
3.495710841	1.8988	E9B1H1;E9B	tr|E9B1H1|E9B1H1_LEIMU Uncharacterized protein (	2
3.755423464	1.8977	E9AZL2	tr|E9AZL2|E9AZL2_LEIMU Uncharacterized protein O	2
3.891835753	1.8975	E9AWL4	tr|E9AWL4|E9AWL4_LEIMU Putative ATP-dependent	5
3.324159552	1.8969	E9AU67	tr|E9AU67|E9AU67_LEIMU Uncharacterized protein (	2
3.582749115	1.8968	E9AYR8	tr|E9AYR8|E9AYR8_LEIMU Uncharacterized protein C	2
3.756908628	1.8963	E9AKZ7	tr|E9AKZ7|E9AKZ7_LEIMU Uncharacterized protein C	2
3.606441802	1.8956	E9AS17	tr|E9AS17|E9AS17_LEIMU Uncharacterized protein O	7
4.171648865	1.8953	E9B699	tr|E9B699|E9B699_LEIMU Putative RNA-binding prot	7

3.83046564	1.8949	E9AJX7	tr|E9AJX7|E9AJX7_LEIMU Uncharacterized protein OS	2
4.010212493	1.8949	E9ALA4	tr|E9ALA4|E9ALA4_LEIMU Splicing factor ptsr1-like p	7
4.107213958	1.8947	E8NHG6;E8I	tr|E8NHG6|E8NHG6_LEIMU Uncharacterized protein	26
4.101739024	1.8946	E9AJT7	tr|E9AJT7|E9AJT7_LEIMU Putative 26S protease regu	6
4.289694528	1.8946	E9AJ97	tr|E9AJ97|E9AJ97_LEIMU Glutaredoxin-like protein C	3
3.852032484	1.8945	E9AZP1	tr|E9AZP1|E9AZP1_LEIMU Uncharacterized protein C	3
3.764366747	1.8944	E9B593;Q0P	tr|E9B593|E9B593_LEIMU Uncharacterized protein C	3
4.040802051	1.8943	E9AYV4	tr|E9AYV4|E9AYV4_LEIMU Uncharacterized protein C	2
4.020988017	1.894	E9B0G8	tr|E9B0G8|E9B0G8_LEIMU Sulfhydryl oxidase OS=Lei	2
4.393214685	1.8938	E9AXD1	tr|E9AXD1|E9AXD1_LEIMU Uncharacterized protein C	4
3.603077232	1.8937	E9AZJ4	tr|E9AZJ4|E9AZJ4_LEIMU Putative rrp44p homologue	2
3.565770994	1.8931	E9AYS2	tr|E9AYS2|E9AYS2_LEIMU Uncharacterized protein O	2
4.07335371	1.8921	E9ASX7	tr|E9ASX7|E9ASX7_LEIMU Chaperonin HSP60, mitoch	12
4.311001904	1.8911	E9AXL1	tr|E9AXL1|E9AXL1_LEIMU Serine/threonine-protein p	2
3.595196023	1.8907	E8NHH1	tr|E8NHH1|E8NHH1_LEIMU WGS CADB00000000 dat	4
4.001310322	1.8905	E9AYP5	tr|E9AYP5|E9AYP5_LEIMU Uncharacterized protein C	2
4.140254441	1.8897	E9ASX8	tr|E9ASX8|E9ASX8_LEIMU Chaperonin HSP60, mitoch	11
4.212821776	1.8897	E9B3K3	tr|E9B3K3|E9B3K3_LEIMU Thiol-dependent reductase	2
4.247891787	1.8892	E9AMM2	tr|E9AMM2|E9AMM2_LEIMU Heat shock protein Hsl	8
4.147827458	1.8885	E8NHS4;E8N	tr|E8NHS4|E8NHS4_LEIMU WGS CADB00000000 data	2
3.882040523	1.8883	E9AK75	tr|E9AK75|E9AK75_LEIMU Uncharacterized protein C	3
4.030819314	1.8876	E9B361	tr|E9B361|E9B361_LEIMU Proteasome regulatory no	6
3.983571542	1.8873	E9B2L9	tr|E9B2L9|E9B2L9_LEIMU Uncharacterized protein O	9
3.635408206	1.8872	E9B4J8	tr|E9B4J8|E9B4J8_LEIMU Uncharacterized protein OS	7
4.07414572	1.8869	E9ALB6	tr|E9ALB6|E9ALB6_LEIMU Putative nucleolar RNA-bir	3
3.945040467	1.8868	E9B1C1	tr|E9B1C1|E9B1C1_LEIMU Uncharacterized protein C	4
3.88065306	1.8867	E9B4T9	tr|E9B4T9|E9B4T9_LEIMU Uncharacterized protein O	2
3.538029923	1.8866	E9B106	tr|E9B106|E9B106_LEIMU Uncharacterized protein C	3
3.968688959	1.8866	E9B3F9	tr|E9B3F9|E9B3F9_LEIMU Uncharacterized protein O	3
3.869842533	1.8864	E9B570	tr|E9B570|E9B570_LEIMU Uncharacterized protein C	5
3.762050076	1.8862	E9B656	tr|E9B656|E9B656_LEIMU Putative DNA polymerase	3
4.593887812	1.8859	E9AZ25	tr|E9AZ25|E9AZ25_LEIMU Uncharacterized protein C	11
4.23528803	1.8848	E9AQS3	tr|E9AQS3|E9AQS3_LEIMU Tyrosyl or methionyl-tRN	5
4.524502049	1.8846	E9AJI9	tr|E9AJI9|E9AJI9_LEIMU Small GTP binding protein ra	3
4.076776576	1.8831	E9AS67	tr|E9AS67|E9AS67_LEIMU Uncharacterized protein C	8
3.770576243	1.8824	E9B5L5	tr|E9B5L5|E9B5L5_LEIMU Uncharacterized protein O	2
3.616419862	1.8822	E9B4I1;A7UI	tr|E9B4I1|E9B4I1_LEIMU Malate dehydrogenase OS=	9
3.985690181	1.8821	E9ALH0	tr|E9ALH0|E9ALH0_LEIMU Uncharacterized protein C	4
3.798449094	1.8812	E9AKI8	tr|E9AKI8|E9AKI8_LEIMU Uncharacterized protein OS	7
4.041730184	1.8812	E9APG0	tr|E9APG0|E9APG0_LEIMU Uncharacterized protein C	11
4.075172054	1.8811	E9AJF8	tr|E9AJF8|E9AJF8_LEIMU Putative ubiquitin activatin	2
3.609881569	1.8795	E9AV52	tr|E9AV52|E9AV52_LEIMU Uncharacterized protein C	12
4.100301071	1.8789	E9B278	tr|E9B278|E9B278_LEIMU Putative ADP-ribosylation	7
3.480034877	1.8789	E9B4R3	tr|E9B4R3|E9B4R3_LEIMU Uncharacterized protein C	2
4.006293111	1.8784	E9B675	tr|E9B675|E9B675_LEIMU Uncharacterized protein C	3
3.871451307	1.8783	E9AZ26	tr|E9AZ26|E9AZ26_LEIMU Uncharacterized protein C	2
3.556057979	1.8783	E9APU4	tr|E9APU4|E9APU4_LEIMU ADP/ATP mitochondrial c	4
3.719887334	1.8775	E9B6R8	tr|E9B6R8|E9B6R8_LEIMU Uncharacterized protein C	7
4.092302515	1.8772	Q6Y8G9;E9A	tr|Q6Y8G9|Q6Y8G9_LEIME Beta adaptin OS=Leishma	2
3.82833255	1.8771	E9AZW2	tr|E9AZW2|E9AZW2_LEIMU Putative DNA polymeras	3

4.169636808	1.8769	E9AVB3	tr|E9AVB3|E9AVB3_LEIMU Putative vesicule-associat	2
4.339399226	1.8769	E9ATU9	tr|E9ATU9|E9ATU9_LEIMU Uncharacterized protein (	4
3.870960011	1.8763	E9B3N2	tr|E9B3N2|E9B3N2_LEIMU Uncharacterized protein (	2
3.953247106	1.8762	E9B5V6	tr|E9B5V6|E9B5V6_LEIMU Conserved CBS domain pr	3
3.636898169	1.8757	E9B406	tr|E9B406|E9B406_LEIMU DNA polymerase OS=Leish	6
3.940903506	1.8755	E9AYL3	tr|E9AYL3|E9AYL3_LEIMU Uncharacterized protein O	2
3.539605442	1.8755	E9AZ99	tr|E9AZ99|E9AZ99_LEIMU Uncharacterized protein C	4
3.215746021	1.8755	E9ASN8	tr|E9ASN8|E9ASN8_LEIMU Uncharacterized protein (	3
3.88582438	1.8748	E9ANK8;E9A	tr|E9ANK8|E9ANK8_LEIMU Putative aminopeptidase	4
3.86147902	1.8743	E9AV05	tr|E9AV05|E9AV05_LEIMU Uncharacterized protein (	3
3.659091454	1.8742	E9AMS6	tr|E9AMS6|E9AMS6_LEIMU Putative acyl-CoA bindin	2
3.715710078	1.8738	E9B397	tr|E9B397|E9B397_LEIMU Pyrroline-5-carboxylate sy	3
4.28043938	1.8738	E9AK40;A0A	tr|E9AK40|E9AK40_LEIMU Putative spermidine synth	3
4.148074929	1.8737	E9B1Y7;E9B	tr|E9B1Y7|E9B1Y7_LEIMU Mannosyltransferase-like	4
3.929707226	1.8736	E9B735	tr|E9B735|E9B735_LEIMU Uncharacterized protein C	2
3.783412616	1.8736	E9AZQ3	tr|E9AZQ3|E9AZQ3_LEIMU Uncharacterized protein (	2
3.871171059	1.8731	E9AVN8	tr|E9AVN8|E9AVN8_LEIMU Kinesin-like protein OS=L	2
4.018843206	1.873	E9ASZ4	tr|E9ASZ4|E9ASZ4_LEIMU Uncharacterized protein O	2
4.141845377	1.8729	E9ATM0	tr|E9ATM0|E9ATM0_LEIMU Proteasome regulatory A	9
3.825416621	1.8723	E9B081	tr|E9B081|E9B081_LEIMU Putative splicing factor 3B	7
3.613723961	1.8723	E9ATM8	tr|E9ATM8|E9ATM8_LEIMU Uncharacterized protein	2
3.8312262	1.8722	E9AT56	tr|E9AT56|E9AT56_LEIMU Uncharacterized protein C	4
4.218060682	1.8719	E9AZZ2	tr|E9AZZ2|E9AZZ2_LEIMU Proteasome regulatory no	12
4.043323537	1.8716	E9AQS6	tr|E9AQS6|E9AQS6_LEIMU Uncharacterized protein (	2
3.999368687	1.8704	E9ARQ0	tr|E9ARQ0|E9ARQ0_LEIMU Putative ras-related prote	3
3.353825557	1.87	E9AM37	tr|E9AM37|E9AM37_LEIMU Uncharacterized protein	2
3.71244501	1.8699	E9B6Z2	tr|E9B6Z2|E9B6Z2_LEIMU Uncharacterized protein O	7
3.952423458	1.8698	E9B3A6	tr|E9B3A6|E9B3A6_LEIMU Putative dynein light inter	3
4.042996055	1.8694	E9B313	tr|E9B313|E9B313_LEIMU Putative cytochrome-b5 re	3
3.996073867	1.8692	E9AM09	tr|E9AM09|E9AM09_LEIMU Uncharacterized protein	2
3.635961217	1.869	E9AYL9	tr|E9AYL9|E9AYL9_LEIMU Uncharacterized protein O	9
3.326332515	1.869	E9AJY3;O09	tr|E9AJY3|E9AJY3_LEIMU Calcium-transporting ATPa	17
4.049122578	1.8689	E9ATX7	tr|E9ATX7|E9ATX7_LEIMU Putative branch point binc	3
3.003646277	1.8667	E9APM1	tr|E9APM1|E9APM1_LEIMU Uncharacterized protein	2
3.529300707	1.8666	E9AV20	tr|E9AV20|E9AV20_LEIMU Uncharacterized protein (	5
3.334942725	1.8666	E9B398	tr|E9B398|E9B398_LEIMU Uncharacterized protein C	5
3.571165983	1.8662	E9AWA2	tr|E9AWA2|E9AWA2_LEIMU Quinone oxidoreductas	2
3.805765314	1.8662	E9ASF4	tr|E9ASF4|E9ASF4_LEIMU Phosphatidylinositol-4-pho	3
3.563726624	1.8657	E9B1F7	tr|E9B1F7|E9B1F7_LEIMU Uncharacterized protein O	4
3.930780665	1.8655	E9B4Z0;P90	tr|E9B4Z0|E9B4Z0_LEIMU ATP-dependent RNA helica	2
4.004184416	1.8654	E9AJU6	tr|E9AJU6|E9AJU6_LEIMU Uncharacterized protein O	3
3.850991979	1.8643	E9B1Z6	tr|E9B1Z6|E9B1Z6_LEIMU Tryparedoxin-like protein (	2
3.234607586	1.8637	E9B555	tr|E9B555|E9B555_LEIMU Uncharacterized protein C	2
3.442297756	1.8637	E9AQJ6;O76	tr|E9AQJ6|E9AQJ6_LEIMU Uncharacterized protein C	4
3.962703052	1.8634	E9AJB7	tr|E9AJB7|E9AJB7_LEIMU Putative acidocalcisomal e	4
3.758996052	1.8631	E9B1B6	tr|E9B1B6|E9B1B6_LEIMU Uncharacterized protein C	4
3.669711539	1.8624	E9AQG1	tr|E9AQG1|E9AQG1_LEIMU Uncharacterized protein	4
4.081880358	1.8623	E9ANK2	tr|E9ANK2|E9ANK2_LEIMU Transcription modulator/	2
3.589710032	1.8617	E9ASH9	tr|E9ASH9|E9ASH9_LEIMU Uncharacterized protein (	2
4.21309888	1.8617	E9ANV2	tr|E9ANV2|E9ANV2_LEIMU Putative cysteinyl-tRNA s	6

3.578577782	1.8614	E9AYH8	tr|E9AYH8|E9AYH8_LEIMU Uncharacterized protein (	2
3.908751579	1.8612	E9B0C5	tr|E9B0C5|E9B0C5_LEIMU Uncharacterized protein L	3
4.330628788	1.8611	E9AT43	tr|E9AT43|E9AT43_LEIMU Dihydrolipoamide acetyltr	15
4.567858439	1.8611	E9AV33	tr|E9AV33|E9AV33_LEIMU Metallo-beta-lactamase fa	2
3.799737573	1.8603	E9AZT8	tr|E9AZT8|E9AZT8_LEIMU Copine i-like protein OS=Le	8
3.58163143	1.8603	E9AK15	tr|E9AK15|E9AK15_LEIMU Putative mitochondrial ex	2
3.766509065	1.86	E9B1Q7	tr|E9B1Q7|E9B1Q7_LEIMU Uncharacterized protein (	2
3.458613894	1.8599	E9AWK8	tr|E9AWK8|E9AWK8_LEIMU Uncharacterized protein	2
3.617808418	1.8588	E9AJZ6	tr|E9AJZ6|E9AJZ6_LEIMU Putative exosome complex	3
3.78851059	1.8588	E9APZ6	tr|E9APZ6|E9APZ6_LEIMU Putative glutathione-S-tra	6
4.030466761	1.8588	E9AZN8	tr|E9AZN8|E9AZN8_LEIMU Putative DNA repair prote	2
3.292102573	1.8587	E9B543	tr|E9B543|E9B543_LEIMU Uncharacterized protein C	2
3.249179163	1.8586	E9AZ84	tr|E9AZ84|E9AZ84_LEIMU Uncharacterized protein C	15
4.042251364	1.8582	Q86PL4;E9A	tr|Q86PL4|Q86PL4_LEIME Paraflagellar rod protein 1	25
3.707163809	1.858	E9B529	tr|E9B529|E9B529_LEIMU DNAj-like protein OS=Leisl	3
3.317044913	1.8578	E9AKC8;E9A	tr|E9AKC8|E9AKC8_LEIMU Putative viscerotropic leis	2
3.894635912	1.8577	E9B6S8	tr|E9B6S8|E9B6S8_LEIMU Uncharacterized protein (N	2
3.659159278	1.8576	E9ALW2	tr|E9ALW2|E9ALW2_LEIMU Uncharacterized protein	5
4.031324834	1.8575	E9AJL7	tr|E9AJL7|E9AJL7_LEIMU Uncharacterized protein OS	4
3.735732655	1.8575	E9B4K9	tr|E9B4K9|E9B4K9_LEIMU Uncharacterized protein C	5
4.125575053	1.8569	E9ATU4	tr|E9ATU4|E9ATU4_LEIMU Uncharacterized protein (	22
3.402590014	1.8568	E9AX43	tr|E9AX43|E9AX43_LEIMU Uncharacterized protein C	6
3.647173984	1.8565	E9B2L7	tr|E9B2L7|E9B2L7_LEIMU Uncharacterized protein O	2
3.602424006	1.8553	E9AZ92	tr|E9AZ92|E9AZ92_LEIMU Uncharacterized protein C	5
3.906382072	1.8549	E9AY26	tr|E9AY26|E9AY26_LEIMU Uncharacterized protein C	2
3.824130391	1.8543	E9B4Y8	tr|E9B4Y8|E9B4Y8_LEIMU Uncharacterized protein O	2
3.645990308	1.8538	E9AQM5	tr|E9AQM5|E9AQM5_LEIMU Uncharacterized protein	2
3.529668721	1.8538	E9B469	tr|E9B469|E9B469_LEIMU Putative udp-glc 4-epimera	4
3.731457986	1.8538	E9AVS3	tr|E9AVS3|E9AVS3_LEIMU Uncharacterized protein C	2
3.835057542	1.8534	E9ASK7	tr|E9ASK7|E9ASK7_LEIMU Putative DEAH-box RNA he	2
4.111437467	1.8531	E9B5Y5	tr|E9B5Y5|E9B5Y5_LEIMU Uncharacterized protein O	2
3.957511628	1.853	E9B594	tr|E9B594|E9B594_LEIMU Uncharacterized protein C	2
3.936367829	1.8523	E9B2R1	tr|E9B2R1|E9B2R1_LEIMU Putative RNA guanylyltran	4
3.928673995	1.8522	E9ARU0	tr|E9ARU0|E9ARU0_LEIMU Uncharacterized protein (	2
3.943360984	1.8518	E9ANZ4	tr|E9ANZ4|E9ANZ4_LEIMU Cytochrome c oxidase sub	17
3.596151915	1.8516	E9APZ1	tr|E9APZ1|E9APZ1_LEIMU Uncharacterized protein C	3
4.320937377	1.8515	E9B0Q4;A0A	tr|E9B0Q4|E9B0Q4_LEIMU Putative Pyridoxal kinase	6
4.049842892	1.8512	E9B6Z8	tr|E9B6Z8|E9B6Z8_LEIMU Uncharacterized protein O	5
3.330075729	1.85	E9AXA3	tr|E9AXA3|E9AXA3_LEIMU Uncharacterized protein (	2
3.849993349	1.8498	E9ASD8	tr|E9ASD8|E9ASD8_LEIMU Putative SET domain prot	2
4.09761385	1.8496	E9B0C7	tr|E9B0C7|E9B0C7_LEIMU Peptidyl-prolyl cis-trans iso	2
3.518682113	1.8495	E9B2V8	tr|E9B2V8|E9B2V8_LEIMU Uncharacterized protein C	2
3.680878818	1.8493	E9ALY1	tr|E9ALY1|E9ALY1_LEIMU Uncharacterized protein O	6
3.801596276	1.8493	E9B5P9	tr|E9B5P9|E9B5P9_LEIMU Putative coatomer zeta su	2
3.825554254	1.8492	E9B2G6;E9A	tr|E9B2G6|E9B2G6_LEIMU 40S ribosomal protein S2	13
3.901712429	1.849	E9B2E4	tr|E9B2E4|E9B2E4_LEIMU Dynein light chain OS=Leis	2
3.758195352	1.8489	E9B379	tr|E9B379|E9B379_LEIMU ATP binding protein-like pr	4
3.477596086	1.8486	E9AP88	tr|E9AP88|E9AP88_LEIMU Uncharacterized protein C	4
3.701672351	1.8482	E9AQS9	tr|E9AQS9|E9AQS9_LEIMU Coatomer subunit delta C	6
4.054285597	1.8481	E9AMR1	tr|E9AMR1|E9AMR1_LEIMU Putative cyclin 1 OS=Leis	3

4.315388898	1.8473	E9B1G1	tr|E9B1G1|E9B1G1_LEIMU Uncharacterized protein (	5
3.656109143	1.8471	E9ANZ7	tr|E9ANZ7|E9ANZ7_LEIMU Uncharacterized protein (	3
3.920929133	1.8471	E9AVR2	tr|E9AVR2|E9AVR2_LEIMU NADH-cytochrome b5 red	4
3.643595816	1.8469	E9AVN9	tr|E9AVN9|E9AVN9_LEIMU Putative proteasome reg	7
3.918125015	1.8468	E9B508	tr|E9B508|E9B508_LEIMU Uncharacterized protein C	5
4.159217982	1.8468	E9AK77	tr|E9AK77|E9AK77_LEIMU Putative adenylate kinase	4
3.720831366	1.8467	E9AKU1	tr|E9AKU1|E9AKU1_LEIMU Uncharacterized protein (	2
4.357465208	1.8466	Q9Y079;Q9Y	tr|Q9Y079|Q9Y079_LEIIN Putative mitogen-activated	3
3.681903615	1.8465	Q4TZP8;E9B	tr|Q4TZP8|Q4TZP8_LEIME Putative mitogen-activate	3
2.969533063	1.8462	E9ALM6	tr|E9ALM6|E9ALM6_LEIMU Uncharacterized protein	3
3.770083629	1.8461	E9AUQ1	tr|E9AUQ1|E9AUQ1_LEIMU Putative phosphatidylinc	3
4.47928472	1.8458	E9APC9	tr|E9APC9|E9APC9_LEIMU Uncharacterized protein C	3
4.136859667	1.8454	E9B6C2	tr|E9B6C2|E9B6C2_LEIMU Putative phosphoinositide	2
4.147002252	1.8453	E9AUB2	tr|E9AUB2|E9AUB2_LEIMU AP complex subunit beta	6
4.223060403	1.8453	E9APT8	tr|E9APT8|E9APT8_LEIMU Putative immunodominan	12
3.881644402	1.8452	E9B0Q2	tr|E9B0Q2|E9B0Q2_LEIMU Uncharacterized protein (	3
3.470361741	1.8451	E9AJR6	tr|E9AJR6|E9AJR6_LEIMU Uncharacterized protein OS	4
3.378344133	1.845	E9B4U7	tr|E9B4U7|E9B4U7_LEIMU Uncharacterized protein (	2
3.969035093	1.8444	E9AUL6	tr|E9AUL6|E9AUL6_LEIMU Uncharacterized protein C	3
3.954040121	1.8443	E9AUY6	tr|E9AUY6|E9AUY6_LEIMU Uncharacterized protein (	3
3.572635363	1.8442	E9APK4	tr|E9APK4|E9APK4_LEIMU Uncharacterized protein C	4
4.161750568	1.8438	E9AQ51	tr|E9AQ51|E9AQ51_LEIMU Uncharacterized protein (	5
4.571805436	1.8433	E9B138	tr|E9B138|E9B138_LEIMU Uncharacterized protein C	2
2.988965152	1.8433	E9AS10	tr|E9AS10|E9AS10_LEIMU Uncharacterized protein C	2
4.398593737	1.8433	E9AM06	tr|E9AM06|E9AM06_LEIMU Elongation factor Ts, mit	2
3.656959579	1.8431	E9AR49	tr|E9AR49|E9AR49_LEIMU Uncharacterized protein C	3
4.131581145	1.8431	E9ALW3	tr|E9ALW3|E9ALW3_LEIMU Uncharacterized protein	5
4.238883985	1.8429	E9B3P0	tr|E9B3P0|E9B3P0_LEIMU Uncharacterized protein C	5
4.122784362	1.8426	E9B2J1	tr|E9B2J1|E9B2J1_LEIMU Putative OSM3-like kinesin	3
3.722742293	1.8425	E9AU95	tr|E9AU95|E9AU95_LEIMU Uncharacterized protein (	3
3.566715753	1.8422	E9B2M5	tr|E9B2M5|E9B2M5_LEIMU Chaperonin containing t-	14
4.268191519	1.8418	E9AYM9	tr|E9AYM9|E9AYM9_LEIMU CDP-diacylglycerol--inosi	2
3.713165192	1.8412	E9ALY7	tr|E9ALY7|E9ALY7_LEIMU Uncharacterized protein O	8
3.989000487	1.8403	E9B2J9	tr|E9B2J9|E9B2J9_LEIMU Putative RNA binding prote	5
3.673407591	1.8393	E9ARP1	tr|E9ARP1|E9ARP1_LEIMU Putative putative 5-oxopr	2
3.924557392	1.8392	E9B4A3	tr|E9B4A3|E9B4A3_LEIMU Putative dynein intermedi	7
3.577701706	1.8392	E9AV56	tr|E9AV56|E9AV56_LEIMU Methionine aminopeptida	3
4.046447741	1.8392	E9B146	tr|E9B146|E9B146_LEIMU Conserved TPR domain pro	9
4.01288989	1.8389	E9AVW5	tr|E9AVW5|E9AVW5_LEIMU Uncharacterized proteir	5
4.024001062	1.8378	E9AUL3	tr|E9AUL3|E9AUL3_LEIMU Uncharacterized protein C	2
3.865137997	1.8378	E9ARG5	tr|E9ARG5|E9ARG5_LEIMU Uncharacterized protein (	10
4.232546756	1.8377	E9AS31	tr|E9AS31|E9AS31_LEIMU Putative signal recognition	5
3.808814474	1.8377	E9B5A0	tr|E9B5A0|E9B5A0_LEIMU Uncharacterized protein C	3
3.666250083	1.8372	E9B303	tr|E9B303|E9B303_LEIMU Uncharacterized protein C	3
3.672467094	1.8364	E9B4U9	tr|E9B4U9|E9B4U9_LEIMU Putative NLI-interacting fa	2
4.353487513	1.8362	E9ARF9	tr|E9ARF9|E9ARF9_LEIMU Putative RNA-binding prot	3
4.414670024	1.8361	E9B191	tr|E9B191|E9B191_LEIMU Uncharacterized protein C	3
3.281805646	1.8361	E9ALP1	tr|E9ALP1|E9ALP1_LEIMU Putative dihydrolipoamide	2
3.299759507	1.836	E9AX75	tr|E9AX75|E9AX75_LEIMU Cullin-like protein-like pro	4
3.722018918	1.836	E9AR96	tr|E9AR96|E9AR96_LEIMU Uncharacterized protein C	9

3.481056231	1.8357	E9ATT6	tr|E9ATT6|E9ATT6_LEIMU Uncharacterized protein C	3
3.763496548	1.8355	E9AUQ4	tr|E9AUQ4|E9AUQ4_LEIMU Uncharacterized protein	2
3.828765831	1.8351	E9ATH0	tr|E9ATH0|E9ATH0_LEIMU Eukaryotic translation init	7
3.704212388	1.8346	E9AV16	tr|E9AV16|E9AV16_LEIMU Putative DnaJ protein OS=	7
4.305016345	1.8342	E9AP58	tr|E9AP58|E9AP58_LEIMU Uncharacterized protein C	2
3.78033713	1.8341	E9ARU5	tr|E9ARU5|E9ARU5_LEIMU Uncharacterized protein (	11
3.809368927	1.8334	E9AZN4	tr|E9AZN4|E9AZN4_LEIMU Uncharacterized protein (	3
3.91515464	1.8333	E9AMY4	tr|E9AMY4|E9AMY4_LEIMU Putative paraflagellar ro	9
3.293669304	1.8332	E9ASV9	tr|E9ASV9|E9ASV9_LEIMU Uncharacterized protein C	2
3.60033709	1.8327	E9AXT6	tr|E9AXT6|E9AXT6_LEIMU Uncharacterized protein C	2
3.428496698	1.8327	E9ASG3	tr|E9ASG3|E9ASG3_LEIMU Vacuolar protein sorting-a	2
3.779358293	1.8325	E8NHK6	tr|E8NHK6|E8NHK6_LEIMU WGS CADB00000000 dat	2
3.838134866	1.8323	E9B485	tr|E9B485|E9B485_LEIMU Uncharacterized protein C	4
4.131203899	1.8322	E9B629	tr|E9B629|E9B629_LEIMU Mitochondrial import inne	2
3.953052844	1.8318	E9ATW8	tr|E9ATW8|E9ATW8_LEIMU Uncharacterized protein	14
3.67512817	1.8311	E9AL22	tr|E9AL22|E9AL22_LEIMU Uncharacterized protein O	3
3.892384254	1.8307	E9AU38	tr|E9AU38|E9AU38_LEIMU Uncharacterized protein (	13
3.851177656	1.8303	E9B0H0	tr|E9B0H0|E9B0H0_LEIMU Eukaryotic translation init	4
3.20874029	1.8293	E9ART7	tr|E9ART7|E9ART7_LEIMU Putative kinesin OS=Leishı	2
4.439640905	1.8291	E9ARB4	tr|E9ARB4|E9ARB4_LEIMU Putative otubain (Otubair	3
3.670640549	1.829	E9B112	tr|E9B112|E9B112_LEIMU Uncharacterized protein C	8
3.950622898	1.829	E9AZC3	tr|E9AZC3|E9AZC3_LEIMU Uncharacterized protein C	3
3.677009056	1.8288	E9AKA9	tr|E9AKA9|E9AKA9_LEIMU Uncharacterized protein (	2
3.960460328	1.8287	E9B2F6	tr|E9B2F6|E9B2F6_LEIMU Uncharacterized protein O	4
4.127651908	1.8285	E9AWK2	tr|E9AWK2|E9AWK2_LEIMU Guanine nucleotide-binc	4
3.944544964	1.8284	E9B0A4	tr|E9B0A4|E9B0A4_LEIMU Putative heat shock prote	5
3.993711689	1.8281	E9B4S8	tr|E9B4S8|E9B4S8_LEIMU Putative mitochondrial ATI	7
3.409317557	1.828	E9AM35	tr|E9AM35|E9AM35_LEIMU Uncharacterized protein	2
3.421332408	1.8277	E9ANT9	tr|E9ANT9|E9ANT9_LEIMU Uncharacterized protein (	4
3.781379625	1.8277	E9B537	tr|E9B537|E9B537_LEIMU Uncharacterized protein C	5
3.8153878	1.8276	E9B096	tr|E9B096|E9B096_LEIMU Uncharacterized protein C	4
3.189883205	1.8273	E9ARK3	tr|E9ARK3|E9ARK3_LEIMU Uncharacterized protein (	2
3.73213355	1.8272	E9ATI4	tr|E9ATI4|E9ATI4_LEIMU Uncharacterized protein OS	2
4.382318358	1.8268	E9AXE4	tr|E9AXE4|E9AXE4_LEIMU Putative epsin OS=Leishma	6
3.731727541	1.8262	E9AT78	tr|E9AT78|E9AT78_LEIMU Putative ATP-dependent R	6
3.711229179	1.826	E9AJ92	tr|E9AJ92|E9AJ92_LEIMU Uncharacterized protein O!	2
4.064754737	1.8258	Q9GRT0;E9E	tr|Q9GRT0|Q9GRT0_LEIME Casein kinase II alpha sub	4
3.80116329	1.8252	E9B3H7	tr|E9B3H7|E9B3H7_LEIMU Putative GIPL galf transfeı	4
3.633764958	1.8249	E9B4B0	tr|E9B4B0|E9B4B0_LEIMU DNA helicase OS=Leishma	4
4.171821945	1.8248	E9AMH0	tr|E9AMH0|E9AMH0_LEIMU Uncharacterized proteir	7
4.518462903	1.8246	E9B549	tr|E9B549|E9B549_LEIMU Uncharacterized protein C	7
3.890303146	1.8243	E9AKD3	tr|E9AKD3|E9AKD3_LEIMU Uncharacterized protein (	3
3.687372799	1.8243	E9AJC6	tr|E9AJC6|E9AJC6_LEIMU Uncharacterized protein O!	2
4.179382503	1.8229	E9B722	tr|E9B722|E9B722_LEIMU Uncharacterized protein C	3
3.8349509	1.8227	E9APE7;E9A	tr|E9APE7|E9APE7_LEIMU 40S ribosomal protein S4 (	14
3.870100513	1.8224	E9AZL5	tr|E9AZL5|E9AZL5_LEIMU 40S ribosomal protein S26	2
3.980284015	1.8223	E9B1E2	tr|E9B1E2|E9B1E2_LEIMU Uncharacterized protein O	20
3.299884209	1.8221	E9AT48	tr|E9AT48|E9AT48_LEIMU Uncharacterized protein C	4
3.773320361	1.8221	E9B082	tr|E9B082|E9B082_LEIMU Putative leucine-rich repea	3
4.322878669	1.8218	E9B5F8	tr|E9B5F8|E9B5F8_LEIMU Vacuolar ATP synthase cat	11

3.762100355	1.8217	E9ATA6	tr|E9ATA6|E9ATA6_LEIMU Putative 60S ribosomal pr	2
3.752394283	1.8216	E9B344	tr|E9B344|E9B344_LEIMU Uncharacterized protein C	2
4.107740854	1.8216	E9AMQ7	tr|E9AMQ7|E9AMQ7_LEIMU Uncharacterized protei	3
4.101576103	1.8212	E9ANC6	tr|E9ANC6|E9ANC6_LEIMU Putative rab1 small GTP-k	3
3.722704346	1.821	E9AZY6	tr|E9AZY6|E9AZY6_LEIMU Uncharacterized protein O	2
3.693812771	1.821	E9AWS4	tr|E9AWS4|E9AWS4_LEIMU Uncharacterized protein	8
4.007541589	1.8208	E9AXC0	tr|E9AXC0|E9AXC0_LEIMU Uncharacterized protein C	6
3.902202356	1.8208	E9B2G7	tr|E9B2G7|E9B2G7_LEIMU Putative prostaglandin f s	3
3.908389683	1.8207	E9B597	tr|E9B597|E9B597_LEIMU Putative exosome subunit	6
3.963161871	1.8206	E9AYY9	tr|E9AYY9|E9AYY9_LEIMU Uncharacterized protein C	9
3.771159696	1.8205	E9AQP7;E8N	tr|E9AQP7|E9AQP7_LEIMU Uncharacterized protein	7
3.655333136	1.8199	E9AQD5	tr|E9AQD5|E9AQD5_LEIMU Uncharacterized protein	3
3.766389349	1.8198	E9B2C2	tr|E9B2C2|E9B2C2_LEIMU Uncharacterized protein C	11
4.309458968	1.8198	E9AR24	tr|E9AR24|E9AR24_LEIMU Putative RNA-binding prot	5
3.476561821	1.8195	E9AU65	tr|E9AU65|E9AU65_LEIMU Putative homocysteine S-	3
3.177883508	1.8192	E9AP92	tr|E9AP92|E9AP92_LEIMU Uncharacterized protein C	4
4.160505787	1.818	O76285;E9A	tr|O76285|O76285_LEIAM Ribonucleotide reductase	5
4.083583619	1.8179	E9B1B1	tr|E9B1B1|E9B1B1_LEIMU Putative calmodulin-relate	10
3.735710865	1.8179	E9AKC4	tr|E9AKC4|E9AKC4_LEIMU Uncharacterized protein C	3
4.117162301	1.8179	E9B2F7	tr|E9B2F7|E9B2F7_LEIMU Uncharacterized protein O	3
3.827741815	1.8176	E8NHJ8;E8N	tr|E8NHJ8|E8NHJ8_LEIMU 60S acidic ribosomal prote	12
3.708001165	1.817	E9ALK4	tr|E9ALK4|E9ALK4_LEIMU Uncharacterized protein O	4
3.484753937	1.8169	E9AZM0	tr|E9AZM0|E9AZM0_LEIMU Uncharacterized protein	3
3.945368768	1.8168	E9B705	tr|E9B705|E9B705_LEIMU Putative peptidyl-prolyl cis	6
4.174497905	1.8167	E9AVZ3	tr|E9AVZ3|E9AVZ3_LEIMU Uncharacterized protein C	2
4.921623072	1.8154	E9B5D1	tr|E9B5D1|E9B5D1_LEIMU Uncharacterized protein C	5
3.985151581	1.8153	E9APL4	tr|E9APL4|E9APL4_LEIMU Uncharacterized protein O	2
4.233760258	1.8149	E9ATX0	tr|E9ATX0|E9ATX0_LEIMU 2-methoxy-6-polyprenyl-1	4
3.764925461	1.8147	E9AJ89	tr|E9AJ89|E9AJ89_LEIMU Kinesin-like protein OS=Lei	2
3.297123103	1.8142	E9AJW8	tr|E9AJW8|E9AJW8_LEIMU Uncharacterized protein	2
3.412554892	1.8139	E9ANB4	tr|E9ANB4|E9ANB4_LEIMU Uncharacterized protein	3
3.895677582	1.8138	E9ALK7	tr|E9ALK7|E9ALK7_LEIMU Rab GDP dissociation inhib	4
3.726082178	1.8136	E9AZP7	tr|E9AZP7|E9AZP7_LEIMU Putative RNA binding prot	2
3.646393428	1.8128	E9ANX3	tr|E9ANX3|E9ANX3_LEIMU Uncharacterized protein	3
3.655389098	1.8125	E9APQ1	tr|E9APQ1|E9APQ1_LEIMU Uncharacterized protein	5
3.656148474	1.812	E9ANH2	tr|E9ANH2|E9ANH2_LEIMU Uncharacterized protein	4
3.614615519	1.8118	E9AT38	tr|E9AT38|E9AT38_LEIMU Folylpolyglutamate synthe	3
2.999386427	1.8104	E9ATY2	tr|E9ATY2|E9ATY2_LEIMU Uncharacterized protein C	2
3.220625987	1.8104	E9B6I1	tr|E9B6I1|E9B6I1_LEIMU 1-alkyl-2-acetylglycerophos	3
4.118679419	1.8104	E9ANU8	tr|E9ANU8|E9ANU8_LEIMU Proteasome regulatory A	10
3.805354429	1.8101	E9AM79	tr|E9AM79|E9AM79_LEIMU Structural maintenance	5
3.816127869	1.8094	E9B544	tr|E9B544|E9B544_LEIMU Uncharacterized protein C	12
4.040420423	1.8082	E9B552	tr|E9B552|E9B552_LEIMU RuvB-like helicase OS=Leis	12
4.384247772	1.8082	E9APV8;E8N	tr|E9APV8|E9APV8_LEIMU Putative kinesin K39 OS=L	7
3.947489769	1.808	E9AVU3;Q4I	tr|E9AVU3|E9AVU3_LEIMU Putative dynein heavy ch	9
3.493986449	1.8079	E9AR35	tr|E9AR35|E9AR35_LEIMU Uncharacterized protein C	3
4.833168362	1.8078	E9B6J5	tr|E9B6J5|E9B6J5_LEIMU Riboflavin kinase/fmn aden	3
4.236916505	1.8077	E9ALP2	tr|E9ALP2|E9ALP2_LEIMU Uncharacterized protein O	4
3.896326548	1.8076	E9AMD5	tr|E9AMD5|E9AMD5_LEIMU Translation initiation fac	4
3.932708332	1.8076	E9AZG6	tr|E9AZG6|E9AZG6_LEIMU Uncharacterized protein C	5

4.512836712	1.8076	E9B0J8	tr|E9B0J8|E9B0J8_LEIMU GrpE protein homolog OS=l	7
3.586596513	1.8075	E9AV88	tr|E9AV88|E9AV88_LEIMU Putative kinesin OS=Leish	9
3.875111806	1.807	E9ASB0	tr|E9ASB0|E9ASB0_LEIMU Uncharacterized protein C	2
3.51127029	1.8069	E9AM81	tr|E9AM81|E9AM81_LEIMU Putative Qc-SNARE prote	2
4.278198366	1.8069	E9AY81	tr|E9AY81|E9AY81_LEIMU Putative dynein heavy cha	12
3.707426185	1.8066	E9AQN7	tr|E9AQN7|E9AQN7_LEIMU Uncharacterized protein	7
4.215880082	1.8063	E9AQ03	tr|E9AQ03|E9AQ03_LEIMU Uncharacterized protein (	2
3.671056216	1.8059	E9ATM4	tr|E9ATM4|E9ATM4_LEIMU ATP-dependent RNA hel	7
4.024284435	1.8056	E9B2X8	tr|E9B2X8|E9B2X8_LEIMU Uncharacterized protein C	3
3.705235347	1.8049	E9B090	tr|E9B090|E9B090_LEIMU Serine/threonine-protein	4
3.875201355	1.8048	E9AZQ1	tr|E9AZQ1|E9AZQ1_LEIMU DNA helicase OS=Leishma	4
3.248319517	1.8045	E9B392	tr|E9B392|E9B392_LEIMU Putative ABC transporter (	3
4.026106585	1.8043	E9B2X2	tr|E9B2X2|E9B2X2_LEIMU Putative heat shock protei	8
4.25968904	1.8038	E9B4M3	tr|E9B4M3|E9B4M3_LEIMU Uncharacterized protein	2
4.368004033	1.8037	E9B228	tr|E9B228|E9B228_LEIMU Uncharacterized protein C	3
3.146358947	1.8036	E9AVV6	tr|E9AVV6|E9AVV6_LEIMU Putative translation initia	5
3.327417114	1.8034	E9B2P7	tr|E9B2P7|E9B2P7_LEIMU Uncharacterized protein C	2
3.859504223	1.8025	E9AJN9	tr|E9AJN9|E9AJN9_LEIMU D-3-phosphoglycerate deh	6
3.535233818	1.8024	E9AQI3	tr|E9AQI3|E9AQI3_LEIMU Uncharacterized protein O	2
3.864959358	1.8022	E9AT60	tr|E9AT60|E9AT60_LEIMU Putative pre-mRNA splicin	2
3.414362256	1.8009	E9AJY8	tr|E9AJY8|E9AJY8_LEIMU Uncharacterized protein OS	2
4.492540025	1.8007	E9AVB9	tr|E9AVB9|E9AVB9_LEIMU V-type proton ATPase sub	7
3.971004731	1.8004	E9AYM2	tr|E9AYM2|E9AYM2_LEIMU Uncharacterized protein	3
3.279273694	1.8004	Q9GRU1;E9A	tr|Q9GRU1|Q9GRU1_LEIME Mitogen-activated prote	2
3.931357121	1.8003	E9AV22	tr|E9AV22|E9AV22_LEIMU Dihydrolipoamide acetyltr	5
4.001822768	1.8002	E9B6L0	tr|E9B6L0|E9B6L0_LEIMU Uncharacterized protein O	5
3.805373488	1.8001	E9APW8	tr|E9APW8|E9APW8_LEIMU Uncharacterized protein	3
4.278848866	1.8001	E9ANH0	tr|E9ANH0|E9ANH0_LEIMU Proteasome subunit alph	4
4.351268829	1.7997	E9AT99	tr|E9AT99|E9AT99_LEIMU 14-3-3 protein-like protein	10
3.576423549	1.7995	E9AS33	tr|E9AS33|E9AS33_LEIMU Uncharacterized protein C	3
4.759742894	1.7993	Q95ZD7;E9A	tr|Q95ZD7|Q95ZD7_LEIME Phosphomannomutase O	2
3.425984459	1.7992	E9B5C7	tr|E9B5C7|E9B5C7_LEIMU Glucose-6-phosphate 1-ep	4
3.604592588	1.7977	E9ASM7	tr|E9ASM7|E9ASM7_LEIMU Putative ribosomal prote	3
3.931244202	1.7975	E9ATN0	tr|E9ATN0|E9ATN0_LEIMU Uncharacterized protein (	3
3.953137836	1.7971	E9B0H7	tr|E9B0H7|E9B0H7_LEIMU Uncharacterized protein (	3
3.977159487	1.797	E9AR52	tr|E9AR52|E9AR52_LEIMU Kinesin-like protein OS=Le	2
3.878261869	1.7969	E9APM8	tr|E9APM8|E9APM8_LEIMU Putative threonine synth	7
3.749145551	1.7969	E9ANM5	tr|E9ANM5|E9ANM5_LEIMU Uncharacterized protein	6
3.530075258	1.7967	E9B4W6;E9I	tr|E9B4W6|E9B4W6_LEIMU D-isomer specific 2-hydr	5
4.162792616	1.7964	E9AX95	tr|E9AX95|E9AX95_LEIMU Ubiquitin carboxyl-termina	4
3.967294927	1.7964	E9AU74	tr|E9AU74|E9AU74_LEIMU Uncharacterized protein (	8
3.867694999	1.7956	E9AVA9	tr|E9AVA9|E9AVA9_LEIMU Putative adenylate kinase	9
3.396905627	1.7953	E9AWU0	tr|E9AWU0|E9AWU0_LEIMU Putative replication fact	4
4.152216831	1.7944	E9AZV4	tr|E9AZV4|E9AZV4_LEIMU Haloacid dehalogenase-lik	4
3.760329405	1.7941	E9AZQ9	tr|E9AZQ9|E9AZQ9_LEIMU Uncharacterized protein (	7
3.950943917	1.7941	E9AX48	tr|E9AX48|E9AX48_LEIMU Putative tubulin folding co	5
3.505550632	1.7938	E9B1X2	tr|E9B1X2|E9B1X2_LEIMU Uncharacterized protein C	6
3.766134256	1.7937	E9AVN0	tr|E9AVN0|E9AVN0_LEIMU Uncharacterized protein	4
3.434153931	1.7936	E9AX32	tr|E9AX32|E9AX32_LEIMU Uncharacterized protein C	3
3.753374791	1.7928	E9AL41	tr|E9AL41|E9AL41_LEIMU Uncharacterized protein O	5

3.731308251	1.7923	E9AQP5	tr|E9AQP5|E9AQP5_LEIMU Uncharacterized protein (	2
4.36427924	1.7921	E9B4F8	tr|E9B4F8|E9B4F8_LEIMU Small nuclear ribonucleopı	2
3.592044201	1.792	E9AJD9	tr|E9AJD9|E9AJD9_LEIMU Putative long-chain-fatty-a	7
3.476788303	1.7914	E9AUV6;E9A	tr|E9AUV6|E9AUV6_LEIMU Putative aminoacylase OS	3
3.570498487	1.7912	E9B0P0	tr|E9B0P0|E9B0P0_LEIMU Putative RNA-binding prot	8
3.899720303	1.7912	E9B4I3	tr|E9B4I3|E9B4I3_LEIMU Malate dehydrogenase OS=	6
4.019981629	1.7912	E9B2L5	tr|E9B2L5|E9B2L5_LEIMU Protein phosphatase meth	3
3.605210855	1.7912	E9APH6	tr|E9APH6|E9APH6_LEIMU Uncharacterized protein (	4
3.868166856	1.7905	E9AMH4	tr|E9AMH4|E9AMH4_LEIMU Uncharacterized proteir	9
4.49038957	1.7905	E9ANK3	tr|E9ANK3|E9ANK3_LEIMU Putative cytochrome b5 (	2
4.062561155	1.7905	E9AU73	tr|E9AU73|E9AU73_LEIMU Uncharacterized protein (	3
3.615561877	1.7902	E9AV00	tr|E9AV00|E9AV00_LEIMU Uncharacterized protein (	3
3.470698453	1.7899	E9ATJ4	tr|E9ATJ4|E9ATJ4_LEIMU Uncharacterized protein OS	2
3.202946027	1.7897	E9ATC0	tr|E9ATC0|E9ATC0_LEIMU Short chain dehydrogenas	3
3.673006264	1.7893	E9B040	tr|E9B040|E9B040_LEIMU Uncharacterized protein C	2
4.056827318	1.7892	E9B6B3	tr|E9B6B3|E9B6B3_LEIMU Uncharacterized protein C	2
3.684768184	1.7889	E9AQT0	tr|E9AQT0|E9AQT0_LEIMU Uncharacterized protein (	3
3.804356231	1.7889	E9AXD0	tr|E9AXD0|E9AXD0_LEIMU Putative ABC transporter	6
3.991810839	1.7888	E9B1T9	tr|E9B1T9|E9B1T9_LEIMU Uncharacterized protein O	6
3.382970217	1.7888	E9B6J9	tr|E9B6J9|E9B6J9_LEIMU Uncharacterized protein OS	3
3.375326864	1.7887	E9APA5	tr|E9APA5|E9APA5_LEIMU Uncharacterized protein (	2
3.607019676	1.7884	E9AL58	tr|E9AL58|E9AL58_LEIMU Uncharacterized protein O	8
3.241753433	1.7884	E9AXA5	tr|E9AXA5|E9AXA5_LEIMU Uncharacterized protein (	2
3.777849915	1.7882	E9AUN1	tr|E9AUN1|E9AUN1_LEIMU Uncharacterized protein	2
4.093380901	1.7882	E9ATI7	tr|E9ATI7|E9ATI7_LEIMU Uncharacterized protein OS	8
3.29620508	1.7878	E9B5P4	tr|E9B5P4|E9B5P4_LEIMU Uncharacterized protein C	2
3.495933718	1.7873	E9ATP6	tr|E9ATP6|E9ATP6_LEIMU Uncharacterized protein C	8
3.606448404	1.7871	E9AN19	tr|E9AN19|E9AN19_LEIMU Uncharacterized protein (	2
5.454168125	1.7871	E9B1X5	tr|E9B1X5|E9B1X5_LEIMU Nucleosome assembly pro	5
3.741452522	1.7871	E9AMC2	tr|E9AMC2|E9AMC2_LEIMU Uncharacterized protein	5
3.734523918	1.7868	E9B6R6	tr|E9B6R6|E9B6R6_LEIMU Uncharacterized protein C	2
3.199728935	1.7867	E9B592	tr|E9B592|E9B592_LEIMU Uncharacterized protein C	2
3.560385398	1.7865	E9ALI0	tr|E9ALI0|E9ALI0_LEIMU Phosphodiesterase OS=Leisl	5
3.570049802	1.7859	E9AY76	tr|E9AY76|E9AY76_LEIMU Uncharacterized protein C	3
4.309260714	1.7858	E9AW04;Q4	tr|E9AW04|E9AW04_LEIMU Peroxidoxin (Tryparedox	7
3.672220547	1.7857	E9B134	tr|E9B134|E9B134_LEIMU Putative replication factor	2
3.979447485	1.7855	E9AKX7	tr|E9AKX7|E9AKX7_LEIMU Uncharacterized protein (	3
3.6914296	1.7853	E9AZK5	tr|E9AZK5|E9AZK5_LEIMU Putative ribonuclease II-lik	6
3.904042491	1.7852	E9ALM1	tr|E9ALM1|E9ALM1_LEIMU Non-specific serine/threc	6
4.217401083	1.785	E9AVE3	tr|E9AVE3|E9AVE3_LEIMU Uncharacterized protein (	3
3.710761667	1.7847	E9AM70	tr|E9AM70|E9AM70_LEIMU GTP-binding protein-like	2
3.531211562	1.7846	E9B510	tr|E9B510|E9B510_LEIMU Uncharacterized protein C	4
4.291687273	1.7846	E9AY44	tr|E9AY44|E9AY44_LEIMU Uncharacterized protein C	4
3.305257223	1.7843	E9B5K3	tr|E9B5K3|E9B5K3_LEIMU Uncharacterized protein C	3
3.61622893	1.7843	E9B3H1	tr|E9B3H1|E9B3H1_LEIMU Uncharacterized protein (	5
3.402743846	1.7841	E9ALG9	tr|E9ALG9|E9ALG9_LEIMU Uncharacterized protein (	6
3.875075868	1.7838	E9B1A0	tr|E9B1A0|E9B1A0_LEIMU Putative ATP-dependent F	4
3.79602292	1.7833	Q9NGK2;E9/	tr|Q9NGK2|Q9NGK2_LEIAM 40S ribosomal protein S:	4
3.82382547	1.7829	E9ARU3	tr|E9ARU3|E9ARU3_LEIMU Uncharacterized protein (	2
4.00624855	1.7823	E9AXI8	tr|E9AXI8|E9AXI8_LEIMU Uncharacterized protein OS	3

3.814322775	1.781	E9B5P1;Q27	tr|E9B5P1|E9B5P1_LEIMU Pyruvate kinase OS=Leishr	3
4.073470488	1.781	E9AJP8	tr|E9AJP8|E9AJP8_LEIMU Putative acetyltransferase	2
4.217149097	1.7809	E9APL9	tr|E9APL9|E9APL9_LEIMU Uncharacterized protein O	4
3.882601529	1.7807	E9AZU1	tr|E9AZU1|E9AZU1_LEIMU Uncharacterized protein (	3
3.526945042	1.7806	E9AMQ3	tr|E9AMQ3|E9AMQ3_LEIMU Uncharacterized protei	9
4.013675627	1.7804	E9B3V4	tr|E9B3V4|E9B3V4_LEIMU Uncharacterized protein C	2
3.823429805	1.7803	E9B6N0	tr|E9B6N0|E9B6N0_LEIMU Structural maintenance o	4
3.379773409	1.7801	E9AUX2	tr|E9AUX2|E9AUX2_LEIMU Uncharacterized protein (	2
4.588543366	1.7799	E9AXK9	tr|E9AXK9|E9AXK9_LEIMU Uncharacterized protein C	2
3.914674133	1.7796	E9AUY4	tr|E9AUY4|E9AUY4_LEIMU Uncharacterized protein (	3
4.047802339	1.7795	E9B0S3	tr|E9B0S3|E9B0S3_LEIMU Putative kinesin OS=Leishr	4
3.754991523	1.7794	E9AQC1	tr|E9AQC1|E9AQC1_LEIMU DNA topoisomerase 2 OS	5
3.855991298	1.7793	E9B6N3	tr|E9B6N3|E9B6N3_LEIMU Uncharacterized protein (	3
3.659355418	1.7792	E9AWQ4;E9	tr|E9AWQ4|E9AWQ4_LEIMU Kinesin-like protein OS=	6
3.683968903	1.779	E9B4N8	tr|E9B4N8|E9B4N8_LEIMU Uncharacterized protein (	15
3.625303771	1.7787	E9AJL8	tr|E9AJL8|E9AJL8_LEIMU Uncharacterized protein OS	2
3.611191209	1.7787	E9B018	tr|E9B018|E9B018_LEIMU Uncharacterized protein C	5
3.45236453	1.7783	E9AX64	tr|E9AX64|E9AX64_LEIMU Putative basal body comp	7
3.496127019	1.7778	E9B694	tr|E9B694|E9B694_LEIMU Uncharacterized protein C	8
4.019771647	1.7775	E9AJV9	tr|E9AJV9|E9AJV9_LEIMU Uncharacterized protein O	2
3.775953011	1.7771	E9AL62;E9A	tr|E9AL62|E9AL62_LEIMU Putative 60S ribosomal pro	6
3.801293462	1.777	E9B064	tr|E9B064|E9B064_LEIMU Putative vacuolar ATP synt	14
3.516436586	1.777	E9AW51	tr|E9AW51|E9AW51_LEIMU Uncharacterized protein	3
4.048079436	1.7769	E9APD3	tr|E9APD3|E9APD3_LEIMU Putative proteasome regu	11
3.526081424	1.7768	E9B2V3	tr|E9B2V3|E9B2V3_LEIMU Uncharacterized protein C	2
4.070982779	1.7768	E9B0J4	tr|E9B0J4|E9B0J4_LEIMU Putative nuclear movemen	3
3.926274973	1.7749	E9B4I2	tr|E9B4I2|E9B4I2_LEIMU Putative malate dehydroge	3
3.703945347	1.7748	E9AJU3	tr|E9AJU3|E9AJU3_LEIMU Putative arginine N-methy	7
3.799866869	1.7743	E9APJ0	tr|E9APJ0|E9APJ0_LEIMU Putative chaperonin TCP20	15
3.330161921	1.7742	E9AUK1	tr|E9AUK1|E9AUK1_LEIMU Uncharacterized protein (	2
3.809958734	1.7741	E9B1E5	tr|E9B1E5|E9B1E5_LEIMU Putative CPSF-domain pro	7
4.093361412	1.774	E9AN66	tr|E9AN66|E9AN66_LEIMU Zinc binding dehydrogena	2
3.515787157	1.7737	E9B3G8	tr|E9B3G8|E9B3G8_LEIMU Putative 60S ribosomal pr	6
3.747813005	1.7729	E9B4V7	tr|E9B4V7|E9B4V7_LEIMU Uncharacterized protein C	3
4.196763038	1.7729	E9B2P5	tr|E9B2P5|E9B2P5_LEIMU Proteasome regulatory no	7
3.674703357	1.7728	E9AM86	tr|E9AM86|E9AM86_LEIMU Putative ATP-dependent	2
3.196153807	1.7723	E9ATT7	tr|E9ATT7|E9ATT7_LEIMU Uncharacterized protein C	2
3.856506204	1.7722	E9AXP0	tr|E9AXP0|E9AXP0_LEIMU Uncharacterized protein C	6
4.023948068	1.7719	E9AT61	tr|E9AT61|E9AT61_LEIMU Uncharacterized protein C	3
3.831429308	1.7715	E9ATP0	tr|E9ATP0|E9ATP0_LEIMU Uncharacterized protein C	6
4.252223108	1.7709	E9AU26	tr|E9AU26|E9AU26_LEIMU Uncharacterized protein (	6
4.445443685	1.7705	E9B196	tr|E9B196|E9B196_LEIMU Uncharacterized protein C	3
3.762471976	1.7703	E9B2S6	tr|E9B2S6|E9B2S6_LEIMU Putative ATP-dependent zi	10
3.443749089	1.7698	E9AP32	tr|E9AP32|E9AP32_LEIMU Uncharacterized protein C	2
3.472557686	1.7697	E9AYZ5	tr|E9AYZ5|E9AYZ5_LEIMU Putative isovaleryl-coA del	11
4.436996952	1.7694	E9AT88	tr|E9AT88|E9AT88_LEIMU Putative ATP synthase OS=	4
3.412623046	1.7693	E9AVM2	tr|E9AVM2|E9AVM2_LEIMU Uncharacterized protei	6
3.588584072	1.7693	Q6Y8H0;E9A	tr|Q6Y8H0|Q6Y8H0_LEIME AP-2 complex subunit alp	3
3.611883493	1.7692	E9B5P3	tr|E9B5P3|E9B5P3_LEIMU Putative 2-oxoisovalerate	6
3.705678459	1.7692	E9AKS8	tr|E9AKS8|E9AKS8_LEIMU Uncharacterized protein C	2

4.197798906	1.7692	E9AKL5;E8N	tr|E9AKL5|E9AKL5_LEIMU Uncharacterized protein O	5
3.542994907	1.7691	E9AUG6	tr|E9AUG6|E9AUG6_LEIMU Midasin OS=Leishmania r	13
4.112681697	1.7691	E9ALG3	tr|E9ALG3|E9ALG3_LEIMU Uncharacterized protein C	2
3.844752216	1.7688	E9ATR2	tr|E9ATR2|E9ATR2_LEIMU Uncharacterized protein C	5
3.482911745	1.7686	E9AQQ7	tr|E9AQQ7|E9AQQ7_LEIMU Uncharacterized protein	5
3.882951737	1.7686	E9ARS6	tr|E9ARS6|E9ARS6_LEIMU Putative pumilio protein C	2
4.032259171	1.7685	E9AQD3	tr|E9AQD3|E9AQD3_LEIMU Putative nucleolar RNA b	15
3.932808724	1.7685	Q86PL6;E9A	tr|Q86PL6|Q86PL6_LEIME Paraflagellar rod protein 4	7
3.177887119	1.7684	E9AZK7	tr|E9AZK7|E9AZK7_LEIMU Uncharacterized protein C	5
3.369330277	1.768	E9AXD9	tr|E9AXD9|E9AXD9_LEIMU DNA-directed RNA polym	3
4.063007372	1.7676	E9AVP4	tr|E9AVP4|E9AVP4_LEIMU Putative proteasome regu	14
3.111716037	1.7675	E9AW84	tr|E9AW84|E9AW84_LEIMU 3-ketoacyl-CoA thiolase-	18
3.556879087	1.7674	E9B2F2	tr|E9B2F2|E9B2F2_LEIMU Uncharacterized protein O	10
3.394633104	1.7672	E9APN9	tr|E9APN9|E9APN9_LEIMU Uncharacterized protein (	3
3.88038761	1.7668	E9B085	tr|E9B085|E9B085_LEIMU Coatomer subunit gamma	17
3.960385422	1.7665	E9AY91	tr|E9AY91|E9AY91_LEIMU Uncharacterized protein C	4
3.614378725	1.7665	E9B2B7	tr|E9B2B7|E9B2B7_LEIMU Uncharacterized protein C	3
3.966599169	1.7663	E9B6Q0	tr|E9B6Q0|E9B6Q0_LEIMU Uncharacterized protein (	9
3.523310118	1.7662	Q9GP07;O0(	tr|Q9GP07|Q9GP07_LEIAM Mitogen-activated protei	2
3.815793204	1.7661	E9B6T5	tr|E9B6T5|E9B6T5_LEIMU Uncharacterized protein O	2
3.548789827	1.7661	E9B6Y8	tr|E9B6Y8|E9B6Y8_LEIMU Putative phosphatidylserir	3
3.912353525	1.7656	E9ATK5	tr|E9ATK5|E9ATK5_LEIMU Uncharacterized protein C	9
3.049213066	1.7652	E9AP42	tr|E9AP42|E9AP42_LEIMU Mitochondrial DNA polym	3
3.936823263	1.7651	E9B2I3	tr|E9B2I3|E9B2I3_LEIMU Uncharacterized protein OS	2
3.655434856	1.7651	E9B074	tr|E9B074|E9B074_LEIMU Uncharacterized protein C	3
4.367647426	1.7649	E9ALK3	tr|E9ALK3|E9ALK3_LEIMU Putative GTP-binding prote	25
3.979443271	1.7641	E9AM44	tr|E9AM44|E9AM44_LEIMU Uncharacterized protein	10
3.596093577	1.7639	E9ATV0	tr|E9ATV0|E9ATV0_LEIMU Uncharacterized protein C	4
4.031443985	1.7638	E9ANG2	tr|E9ANG2|E9ANG2_LEIMU Uncharacterized protein	2
4.080305592	1.7635	E9ARP5	tr|E9ARP5|E9ARP5_LEIMU Protein kinase A catalytic	4
3.549183574	1.7634	E9AZE5	tr|E9AZE5|E9AZE5_LEIMU Uncharacterized protein O	2
3.601955049	1.763	E9B108	tr|E9B108|E9B108_LEIMU Sphingosine phosphate lya	4
3.958403281	1.7619	E9AVQ7	tr|E9AVQ7|E9AVQ7_LEIMU Uncharacterized protein	13
3.903784324	1.7618	E9AJV8	tr|E9AJV8|E9AJV8_LEIMU Uncharacterized protein O	3
3.858162114	1.7616	E9AMY1	tr|E9AMY1|E9AMY1_LEIMU Uncharacterized protein	3
3.498815511	1.7609	E9B3C4	tr|E9B3C4|E9B3C4_LEIMU Uncharacterized protein C	4
3.734774986	1.7606	E9B5K7	tr|E9B5K7|E9B5K7_LEIMU Uncharacterized protein C	3
3.806742826	1.7605	E9AYV2	tr|E9AYV2|E9AYV2_LEIMU Putative calpain-like cyste	5
3.854936544	1.7605	E9B2J2	tr|E9B2J2|E9B2J2_LEIMU Uncharacterized protein OS	2
3.875671156	1.7603	E8NHE2	tr|E8NHE2|E8NHE2_LEIMU Uncharacterized protein I	3
3.286846429	1.76	E9ATS5	tr|E9ATS5|E9ATS5_LEIMU Uncharacterized protein O	2
3.599295799	1.7597	E9B155	tr|E9B155|E9B155_LEIMU Putative chaperonin HSP6(	10
3.887884984	1.7596	E9B5P8	tr|E9B5P8|E9B5P8_LEIMU Uncharacterized protein C	4
3.758313105	1.7596	E9AM64	tr|E9AM64|E9AM64_LEIMU Uncharacterized protein	8
4.322475689	1.7595	E9B5D3	tr|E9B5D3|E9B5D3_LEIMU DNA topoisomerase I OS=	5
4.220521736	1.7594	Q86PL7;E9A	tr|Q86PL7|Q86PL7_LEIME Putative small GTP-binding	6
3.780591292	1.7593	E9B760	tr|E9B760|E9B760_LEIMU Uncharacterized protein C	10
3.952649406	1.7583	E9ART0	tr|E9ART0|E9ART0_LEIMU Uncharacterized protein C	2
4.301757712	1.7579	E9AMI0	tr|E9AMI0|E9AMI0_LEIMU Uncharacterized protein (	13
3.742708123	1.7579	E9AVX7	tr|E9AVX7|E9AVX7_LEIMU Peptidyl-prolyl cis-trans is	2

3.647839869	1.7577	E9B5Z9	tr|E9B5Z9|E9B5Z9_LEIMU Putative pre-mRNA splicing	5
3.71714836	1.7576	E9B2I0	tr|E9B2I0|E9B2I0_LEIMU ATP-dependent RNA helicas	5
3.594412427	1.757	E9B2W2	tr|E9B2W2|E9B2W2_LEIMU Chaperonin HSP60, mito	8
4.168607663	1.7566	E9B039	tr|E9B039|E9B039_LEIMU Uncharacterized protein C	3
3.762367974	1.7566	E9B5D2	tr|E9B5D2|E9B5D2_LEIMU Cleavage and polyadenyla	5
3.741855759	1.7565	E9ANS3	tr|E9ANS3|E9ANS3_LEIMU Uncharacterized protein (	3
3.448536243	1.7565	E9AVS2	tr|E9AVS2|E9AVS2_LEIMU Uncharacterized protein C	2
3.913953388	1.7562	E9AXX1	tr|E9AXX1|E9AXX1_LEIMU Putative adenylate kinase	4
3.778850135	1.756	E8NHI0	tr|E8NHI0|E8NHI0_LEIMU 40S ribosomal protein S3a	13
3.414207565	1.7559	E9B6T6	tr|E9B6T6|E9B6T6_LEIMU Uncharacterized protein O	3
3.751465195	1.7554	E9AP12	tr|E9AP12|E9AP12_LEIMU Uncharacterized protein C	10
3.322405001	1.7552	E9B5A8	tr|E9B5A8|E9B5A8_LEIMU Uncharacterized protein C	5
3.604583049	1.7551	E9ASM0	tr|E9ASM0|E9ASM0_LEIMU Uncharacterized protein	7
3.720673298	1.7549	E9AW70	tr|E9AW70|E9AW70_LEIMU Putative ubiquitin-activa	7
3.788581232	1.7545	E9AXM7	tr|E9AXM7|E9AXM7_LEIMU Uncharacterized protein	8
4.347622935	1.7542	E9B3K9	tr|E9B3K9|E9B3K9_LEIMU Autophagy-related protein	2
3.756700455	1.754	E9B1C6	tr|E9B1C6|E9B1C6_LEIMU S-adenosylmethionine syn	16
3.422763389	1.7539	E9AXX0	tr|E9AXX0|E9AXX0_LEIMU Uncharacterized protein C	3
3.589236913	1.7536	E9ALT4	tr|E9ALT4|E9ALT4_LEIMU Uncharacterized protein O	3
3.570136031	1.7536	E9AYL0	tr|E9AYL0|E9AYL0_LEIMU Uncharacterized protein O	3
3.860665628	1.7534	E9B483	tr|E9B483|E9B483_LEIMU Uncharacterized protein C	7
4.185074734	1.7531	E9AXT4	tr|E9AXT4|E9AXT4_LEIMU Uncharacterized protein C	7
3.966319776	1.7527	E9B3B0	tr|E9B3B0|E9B3B0_LEIMU Putative chaperonin alpha	20
3.882418962	1.7523	E9AWK1	tr|E9AWK1|E9AWK1_LEIMU Uncharacterized protein	3
4.23594988	1.7522	E9ASK9	tr|E9ASK9|E9ASK9_LEIMU Eukaryotic translation initi	2
4.369077425	1.7519	E9AT87	tr|E9AT87|E9AT87_LEIMU Uncharacterized protein C	5
3.630991045	1.7519	E9AYP7	tr|E9AYP7|E9AYP7_LEIMU Uncharacterized protein C	5
3.784199175	1.7516	E9API5	tr|E9API5|E9API5_LEIMU Kinesin-like protein OS=Leis	6
3.992708468	1.7515	E9AKU0	tr|E9AKU0|E9AKU0_LEIMU Carbonic anhydrase OS=L	2
3.996886873	1.7514	E9AWN2	tr|E9AWN2|E9AWN2_LEIMU Ubiquitin carboxyl-term	5
3.913367579	1.7512	E9B5B2	tr|E9B5B2|E9B5B2_LEIMU Uncharacterized protein C	3
3.919611471	1.7512	E9B201;E9B	tr|E9B201|E9B201_LEIMU Succinyl-diaminopimelate	3
3.590586672	1.7511	E9AVC7	tr|E9AVC7|E9AVC7_LEIMU 2-oxoisovalerate dehydro	6
3.62119291	1.7506	E9AZ85	tr|E9AZ85|E9AZ85_LEIMU Putative GTP binding prote	2
4.240056443	1.7505	E9B489	tr|E9B489|E9B489_LEIMU Uncharacterized protein C	5
4.243584839	1.7502	E9B2B6	tr|E9B2B6|E9B2B6_LEIMU Uncharacterized protein C	3
3.762951781	1.7501	E9AM77	tr|E9AM77|E9AM77_LEIMU Transcription factor-like	13
3.151897428	1.7499	E9B676	tr|E9B676|E9B676_LEIMU Uncharacterized protein C	3
3.718243786	1.7496	E9AW96	tr|E9AW96|E9AW96_LEIMU Actin interacting protein	5
3.496269634	1.7493	E9B576	tr|E9B576|E9B576_LEIMU Putative serine acetyltrans	3
3.657835287	1.7492	E9AJV2	tr|E9AJV2|E9AJV2_LEIMU Uncharacterized protein O	20
3.894871464	1.7491	E9AQ24	tr|E9AQ24|E9AQ24_LEIMU Lysine--tRNA ligase OS=Le	17
3.784480051	1.7488	E9ATZ0	tr|E9ATZ0|E9ATZ0_LEIMU Uncharacterized protein O	2
3.856561114	1.7487	Q7YWC7;E9	tr|Q7YWC7|Q7YWC7_LEIME Mu adaptin OS=Leishma	3
4.122345068	1.7485	E9AWG1	tr|E9AWG1|E9AWG1_LEIMU Uncharacterized protein	5
3.829448894	1.7484	E9AJS8	tr|E9AJS8|E9AJS8_LEIMU Conserved TLD domain pro	2
3.803441393	1.7482	E9ALY6;Q9U	tr|E9ALY6|E9ALY6_LEIMU Putative ADP ribosylation f	8
3.531540559	1.7481	E9AR76	tr|E9AR76|E9AR76_LEIMU Hydrolase-like protein OS=	2
3.625364765	1.7477	E9AQ76	tr|E9AQ76|E9AQ76_LEIMU Uncharacterized protein (	4
3.902185778	1.7475	E9AYQ9	tr|E9AYQ9|E9AYQ9_LEIMU Uncharacterized protein (	2

3.81924058	1.7475	E9AN48	tr|E9AN48|E9AN48_LEIMU Dihydroxyacetone kinase	4
3.897179391	1.7473	E9ANJ3	tr|E9ANJ3|E9ANJ3_LEIMU Putative pumilio-repeat, R	10
3.352455592	1.7464	E9B6I7	tr|E9B6I7|E9B6I7_LEIMU Putative glycerol kinase, gly	2
3.97232823	1.7464	E9AN63	tr|E9AN63|E9AN63_LEIMU Uncharacterized protein (	4
4.034203191	1.7456	E9AYW0	tr|E9AYW0|E9AYW0_LEIMU Uncharacterized protein	2
3.59948865	1.7456	E9AWI7	tr|E9AWI7|E9AWI7_LEIMU Uncharacterized protein (	3
3.196865525	1.7455	E9B1L9	tr|E9B1L9|E9B1L9_LEIMU Uncharacterized protein O	5
3.736732622	1.745	E9AMM1	tr|E9AMM1|E9AMM1_LEIMU Uncharacterized prote	9
3.66968123	1.7448	E9ASC3	tr|E9ASC3|E9ASC3_LEIMU Putative L-ribulokinase OS	2
3.376029716	1.7447	E9B3A1	tr|E9B3A1|E9B3A1_LEIMU Uncharacterized protein (	2
3.824480226	1.7447	E9B5H0	tr|E9B5H0|E9B5H0_LEIMU Phosphomannomutase-lil	2
3.344569865	1.7443	E9B624	tr|E9B624|E9B624_LEIMU Uncharacterized protein C	7
3.842799162	1.7441	E9AXV3	tr|E9AXV3|E9AXV3_LEIMU Uncharacterized protein (	2
3.508269709	1.7438	E9AK58	tr|E9AK58|E9AK58_LEIMU Putative 60S ribosomal pr	8
3.210597676	1.7432	E9AYY3	tr|E9AYY3|E9AYY3_LEIMU Glutaredoxin-like protein (	2
3.90232722	1.7423	E9B159	tr|E9B159|E9B159_LEIMU Uncharacterized protein C	4
4.212227627	1.7422	E9ANB3	tr|E9ANB3|E9ANB3_LEIMU RNA-binding protein-like	3
3.387890337	1.7421	E9ALH7	tr|E9ALH7|E9ALH7_LEIMU Putative 60S ribosomal pr	6
3.863147422	1.7417	E9ARM2	tr|E9ARM2|E9ARM2_LEIMU Uncharacterized protein	2
3.514322614	1.7414	E9AVE5	tr|E9AVE5|E9AVE5_LEIMU Choline dehydrogenase, li	3
3.460861725	1.7413	E9AUN5	tr|E9AUN5|E9AUN5_LEIMU 1,2-dihydroxy-3-keto-5-r	2
3.720566347	1.7413	E9AZA2	tr|E9AZA2|E9AZA2_LEIMU Putative rRNA methyltran	6
3.937891791	1.7413	E9ARW7	tr|E9ARW7|E9ARW7_LEIMU Putative aminopeptidas	7
3.735499084	1.741	E9AJP0	tr|E9AJP0|E9AJP0_LEIMU 2-aminoethylphosphonate:	4
4.033009775	1.7409	E9B2I6	tr|E9B2I6|E9B2I6_LEIMU Uncharacterized protein OS	3
3.931148617	1.74	E9AL29	tr|E9AL29|E9AL29_LEIMU Serine/threonine-protein p	7
4.0062928	1.7399	E9AL40	tr|E9AL40|E9AL40_LEIMU Uncharacterized protein O	3
3.425539802	1.7395	E9AZI5	tr|E9AZI5|E9AZI5_LEIMU Glycerol-3-phosphate dehy	4
3.695424319	1.7382	E9ARW5	tr|E9ARW5|E9ARW5_LEIMU Uncharacterized protein	5
4.312122983	1.7382	E9B573	tr|E9B573|E9B573_LEIMU Regulatory subunit of prot	16
3.530718708	1.7381	E9AXY6	tr|E9AXY6|E9AXY6_LEIMU Glycine cleavage system P	4
4.080564419	1.7379	E9AJV4	tr|E9AJV4|E9AJV4_LEIMU Uncharacterized protein O	2
3.719243673	1.7377	E9AW60	tr|E9AW60|E9AW60_LEIMU Uncharacterized protein	6
4.116924359	1.7372	E9AZX3	tr|E9AZX3|E9AZX3_LEIMU Putative DEAD box RNA he	7
4.581458289	1.737	E9AR10	tr|E9AR10|E9AR10_LEIMU Uncharacterized protein (	6
4.249726189	1.7369	E9B110	tr|E9B110|E9B110_LEIMU ADP-ribosylation factor-lik	3
4.550048534	1.7368	E9AXG0	tr|E9AXG0|E9AXG0_LEIMU Prefoldin subunit 4 OS=Le	3
3.878330381	1.7361	E9ASB2	tr|E9ASB2|E9ASB2_LEIMU ATP-dependent zinc metal	2
3.747537371	1.7358	E9AYH0	tr|E9AYH0|E9AYH0_LEIMU Uncharacterized protein (	4
4.29433067	1.7357	E9AW01	tr|E9AW01|E9AW01_LEIMU Uncharacterized protein	11
3.62905805	1.7355	E9B163	tr|E9B163|E9B163_LEIMU Putative aldehyde dehydro	10
3.596080479	1.7353	E9B5J1	tr|E9B5J1|E9B5J1_LEIMU Uncharacterized protein OS	8
3.801447803	1.7351	E9AT85	tr|E9AT85|E9AT85_LEIMU Fibrillarin OS=Leishmania r	9
3.751100376	1.7349	Q9GRU2;E9/	tr|Q9GRU2|Q9GRU2_LEIME Mitogen-activated prote	2
4.23543463	1.7349	E9AMI2	tr|E9AMI2|E9AMI2_LEIMU Uncharacterized protein (	4
3.380793869	1.7346	E9AZL4	tr|E9AZL4|E9AZL4_LEIMU RAD50 DNA repair-like pro	6
4.091353222	1.7346	E9B3S9	tr|E9B3S9|E9B3S9_LEIMU Putative nicotinate phosph	2
3.795663249	1.7341	E9AQX9	tr|E9AQX9|E9AQX9_LEIMU Uncharacterized protein (	8
4.000351693	1.7338	E9AQG2	tr|E9AQG2|E9AQG2_LEIMU Putative peroxisome ass	3
3.702881562	1.7338	E9B677	tr|E9B677|E9B677_LEIMU Uncharacterized protein C	11

3.832850796	1.7336	E9AX93	tr|E9AX93|E9AX93_LEIMU Cleavage and polyadenyla	8
3.671211857	1.7336	E9AZU3	tr|E9AZU3|E9AZU3_LEIMU Putative p450 reductase (	3
3.592198158	1.733	E9AUK9	tr|E9AUK9|E9AUK9_LEIMU Putative serine/threonine	7
3.907224155	1.7326	E9AJJ9	tr|E9AJJ9|E9AJJ9_LEIMU Putative casein kinase II, alp	3
3.938138968	1.7323	E9B1D8	tr|E9B1D8|E9B1D8_LEIMU Uncharacterized protein (	4
3.780276931	1.7322	E9AU01	tr|E9AU01|E9AU01_LEIMU Putative RNA helicase OS	4
3.59130046	1.7318	E9AJ91	tr|E9AJ91|E9AJ91_LEIMU Putative carboxylase OS=Le	6
3.545441726	1.7314	E9AX02	tr|E9AX02|E9AX02_LEIMU Uncharacterized protein C	2
3.431235729	1.7314	E9AMI9	tr|E9AMI9|E9AMI9_LEIMU Uncharacterized protein (	5
3.681171181	1.7313	E9AY79	tr|E9AY79|E9AY79_LEIMU Uncharacterized protein C	2
3.67194183	1.7313	E9B5K1	tr|E9B5K1|E9B5K1_LEIMU Putative ubiquitin hydrola	8
3.648943862	1.7312	E9B689	tr|E9B689|E9B689_LEIMU Uncharacterized protein C	2
4.20757071	1.731	E9AXQ4	tr|E9AXQ4|E9AXQ4_LEIMU Uncharacterized protein (	4
3.101734302	1.7303	E9ARG9	tr|E9ARG9|E9ARG9_LEIMU Uncharacterized protein (	3
4.576315997	1.7303	E9ALD1	tr|E9ALD1|E9ALD1_LEIMU Proteasome regulatory nc	2
3.517182926	1.7303	E9AMU6	tr|E9AMU6|E9AMU6_LEIMU Uncharacterized protein	2
3.28858074	1.7301	E9AXA1	tr|E9AXA1|E9AXA1_LEIMU Uncharacterized protein (	3
3.493531595	1.7298	E9AQ34;E9A	tr|E9AQ34|E9AQ34_LEIMU Uncharacterized protein (	2
4.025928563	1.7298	E9AV10	tr|E9AV10|E9AV10_LEIMU Uncharacterized protein (	10
3.613566815	1.7289	E9AYA1	tr|E9AYA1|E9AYA1_LEIMU Uncharacterized protein (	3
4.492798331	1.7285	E9ALB1	tr|E9ALB1|E9ALB1_LEIMU Uncharacterized protein O	4
3.616289544	1.7284	E9B6K3	tr|E9B6K3|E9B6K3_LEIMU Uncharacterized protein C	2
3.884878088	1.7283	E9AN88	tr|E9AN88|E9AN88_LEIMU Uncharacterized protein (	3
3.983664914	1.7281	E9B1J0	tr|E9B1J0|E9B1J0_LEIMU Putative calpain-like cysteir	5
3.959615061	1.7272	E9AR41	tr|E9AR41|E9AR41_LEIMU Uncharacterized protein (	6
4.036168064	1.7271	E9B1B7	tr|E9B1B7|E9B1B7_LEIMU Uncharacterized protein C	8
3.550973225	1.727	E9AY52	tr|E9AY52|E9AY52_LEIMU Uncharacterized protein C	2
3.922246526	1.7264	E9B6C0	tr|E9B6C0|E9B6C0_LEIMU Uncharacterized protein C	8
3.489821694	1.7259	E9AU45	tr|E9AU45|E9AU45_LEIMU Uncharacterized protein (	5
3.798877228	1.7257	E9AQV4	tr|E9AQV4|E9AQV4_LEIMU Uncharacterized protein	5
4.08067758	1.7255	E9AJE9	tr|E9AJE9|E9AJE9_LEIMU Uncharacterized protein OS	3
3.888986617	1.7249	E9AZC6	tr|E9AZC6|E9AZC6_LEIMU Uncharacterized protein C	3
4.026622468	1.7249	E9ALG0	tr|E9ALG0|E9ALG0_LEIMU Uncharacterized protein (	2
3.556636782	1.7248	E9B3P7	tr|E9B3P7|E9B3P7_LEIMU Uncharacterized protein C	8
3.342562682	1.7243	E9ATL2	tr|E9ATL2|E9ATL2_LEIMU Uncharacterized protein O	5
3.791946858	1.7243	E9AZ12	tr|E9AZ12|E9AZ12_LEIMU Uncharacterized protein C	6
3.730494322	1.7242	E9B1L4	tr|E9B1L4|E9B1L4_LEIMU Uncharacterized protein O	3
3.371062724	1.7242	E9B1G7	tr|E9B1G7|E9B1G7_LEIMU Putative nucleolar proteir	4
3.699376079	1.724	E9B6U3	tr|E9B6U3|E9B6U3_LEIMU Polyadenylate-binding pr	14
3.923173686	1.7238	E9ANB6	tr|E9ANB6|E9ANB6_LEIMU Uncharacterized protein (	3
3.709946381	1.7236	E9B2Z7	tr|E9B2Z7|E9B2Z7_LEIMU Uncharacterized protein O	3
3.441244145	1.7235	E9B407	tr|E9B407|E9B407_LEIMU Transcription activator OS	10
3.980174617	1.7232	E9B1D2	tr|E9B1D2|E9B1D2_LEIMU Putative serine/threonine	4
3.952800776	1.7227	E9AM68	tr|E9AM68|E9AM68_LEIMU Uncharacterized protein	15
3.800467851	1.7225	E9AL38	tr|E9AL38|E9AL38_LEIMU Putative ubiquitin-protein	3
4.173582955	1.7222	E9B2W9	tr|E9B2W9|E9B2W9_LEIMU Uncharacterized protein	8
3.650441847	1.7222	E9ARK0	tr|E9ARK0|E9ARK0_LEIMU Putative 60S ribosomal pr	5
3.293318301	1.7222	E9B2X4	tr|E9B2X4|E9B2X4_LEIMU Putative 1-acyl-sn-glycerol	4
4.424348524	1.7221	E9AVZ5	tr|E9AVZ5|E9AVZ5_LEIMU Putative CCR4 associated	4
3.760615674	1.7219	E9ARP8	tr|E9ARP8|E9ARP8_LEIMU Putative methyltransferas	3

3.611854449	1.7218	E9ATB0	tr|E9ATB0|E9ATB0_LEIMU Putative actin-like protein	2
3.793330383	1.7218	E9AL86	tr|E9AL86|E9AL86_LEIMU Uncharacterized protein O	5
4.224173033	1.7218	E9AXF0	sp|E9AXF0|IF5A_LEIMU Eukaryotic translation initiati	3
4.384058676	1.7217	E9B2W1	tr|E9B2W1|E9B2W1_LEIMU Putative rab11B GTPase	2
3.800876746	1.7209	E9AUY5	tr|E9AUY5|E9AUY5_LEIMU Uncharacterized protein (	6
3.529593657	1.7208	E9AMC1	tr|E9AMC1|E9AMC1_LEIMU Uncharacterized protein	4
3.973724858	1.7201	E9AQL1;Q9I	tr|E9AQL1|E9AQL1_LEIMU Dihydroorotate dehydrog	4
3.776213579	1.72	E9AYW4	tr|E9AYW4|E9AYW4_LEIMU Uncharacterized protein	4
3.588417132	1.7199	E9AL60	tr|E9AL60|E9AL60_LEIMU Uncharacterized protein O	5
3.752787557	1.7199	E9B3F8	tr|E9B3F8|E9B3F8_LEIMU Uncharacterized protein O	2
3.760286789	1.7198	E9AR04	tr|E9AR04|E9AR04_LEIMU Uncharacterized protein C	2
3.63128779	1.7196	E9AV49;A0A	tr|E9AV49|E9AV49_LEIMU Putative methionyl-tRNA :	13
4.430440376	1.7195	E9B072	tr|E9B072|E9B072_LEIMU Eukaryotic translation initi	3
3.817829651	1.7193	E9B1W5	tr|E9B1W5|E9B1W5_LEIMU Uncharacterized protein	2
3.461792261	1.7191	E9AL42	tr|E9AL42|E9AL42_LEIMU Uncharacterized protein O	2
3.874217464	1.7186	E9AK09	tr|E9AK09|E9AK09_LEIMU Uncharacterized protein C	3
4.611190572	1.7185	E9B5E2	tr|E9B5E2|E9B5E2_LEIMU Uncharacterized protein O	2
3.032832428	1.7183	E9AYA4	tr|E9AYA4|E9AYA4_LEIMU Uncharacterized protein (	2
3.494392465	1.7182	E9ASG5	tr|E9ASG5|E9ASG5_LEIMU Uncharacterized protein (	2
3.931273545	1.7181	E9B2L3	tr|E9B2L3|E9B2L3_LEIMU Ethanolamine-phosphate c	6
3.803696313	1.7181	E9B1W1	tr|E9B1W1|E9B1W1_LEIMU Diphthine synthase-like	5
3.635788655	1.7181	E9B4P2	tr|E9B4P2|E9B4P2_LEIMU Uncharacterized protein C	3
4.244309921	1.718	E9AZ24	tr|E9AZ24|E9AZ24_LEIMU Putative translation initiat	4
3.840536818	1.7178	E9B3L1	tr|E9B3L1|E9B3L1_LEIMU Putative ATP-binding casse	8
3.602958161	1.7175	E9AWJ0	tr|E9AWJ0|E9AWJ0_LEIMU Uncharacterized protein	2
3.72933421	1.7168	E9ANI7	tr|E9ANI7|E9ANI7_LEIMU Ribosome biogenesis prote	5
3.244236783	1.7167	E9AZD1	tr|E9AZD1|E9AZD1_LEIMU Uncharacterized protein (	2
3.820900321	1.7166	E9B1P7	tr|E9B1P7|E9B1P7_LEIMU Uncharacterized protein C	6
3.719499715	1.7161	E9ANN3	tr|E9ANN3|E9ANN3_LEIMU Uncharacterized protein	5
3.720685433	1.7156	E9B0G6	tr|E9B0G6|E9B0G6_LEIMU WD-40 repeat protein OS:	3
3.639006185	1.7154	E9ANZ3	tr|E9ANZ3|E9ANZ3_LEIMU Serine/threonine-protein	2
3.476331052	1.7153	E9ALB0	tr|E9ALB0|E9ALB0_LEIMU Uncharacterized protein O	3
3.394857143	1.7153	E9B4W9	tr|E9B4W9|E9B4W9_LEIMU Uncharacterized protein	2
3.175129949	1.7148	E9AQT7	tr|E9AQT7|E9AQT7_LEIMU Uncharacterized protein (	2
3.732593836	1.7146	E9APK2	tr|E9APK2|E9APK2_LEIMU Uncharacterized protein C	4
3.571072535	1.714	E9ALM8	tr|E9ALM8|E9ALM8_LEIMU Putative fumarate hydra	2
3.620283657	1.714	E9AX91	tr|E9AX91|E9AX91_LEIMU Uncharacterized protein C	3
3.713425067	1.7137	E9AXI7	tr|E9AXI7|E9AXI7_LEIMU Uncharacterized protein OS	3
3.000790325	1.7136	E9AW83	tr|E9AW83|E9AW83_LEIMU Uncharacterized protein	4
3.907136052	1.7135	E9B754	tr|E9B754|E9B754_LEIMU Uncharacterized protein C	2
3.803851957	1.7135	E9B6I5	tr|E9B6I5|E9B6I5_LEIMU Putative ubiquitin-activating	16
3.560870318	1.7134	E9B207	tr|E9B207|E9B207_LEIMU Uncharacterized protein C	3
3.570606907	1.7128	E9AWL7	tr|E9AWL7|E9AWL7_LEIMU Dynein intermediate-cha	5
3.993376397	1.7127	E9AWV6	tr|E9AWV6|E9AWV6_LEIMU Uncharacterized protein	5
3.364991203	1.7125	E9AR50	tr|E9AR50|E9AR50_LEIMU GMP reductase OS=Leishr	13
3.76794226	1.7123	E9ASR8	tr|E9ASR8|E9ASR8_LEIMU Uncharacterized protein C	2
4.167710151	1.7122	E9AW16	tr|E9AW16|E9AW16_LEIMU Uncharacterized protein	3
3.692548764	1.7119	E9AKW6	tr|E9AKW6|E9AKW6_LEIMU Putative acyl-coenzyme	7
3.593382189	1.7117	E9ARJ4	tr|E9ARJ4|E9ARJ4_LEIMU Uncharacterized protein O:	3
3.516093411	1.7117	E9B6F1	tr|E9B6F1|E9B6F1_LEIMU Uncharacterized protein O	3

3.405930955	1.7116	E9AKY3	tr|E9AKY3|E9AKY3_LEIMU Uncharacterized protein C	2
3.600023114	1.7116	E9AT02;A0A	tr|E9AT02|E9AT02_LEIMU Developmentally regulate	8
3.700439351	1.7114	E9ARJ9;E9A	tr|E9ARJ9|E9ARJ9_LEIMU Putative ATP-dependent zi	3
3.814786488	1.7114	E9AMS7	tr|E9AMS7|E9AMS7_LEIMU Uncharacterized protein	8
3.658863424	1.7106	E9ATD6	tr|E9ATD6|E9ATD6_LEIMU Putative signal recognitio	7
3.386279616	1.7105	E9AW40	tr|E9AW40|E9AW40_LEIMU Putative NADP-depende	10
3.965459051	1.71	E9AKM7	tr|E9AKM7|E9AKM7_LEIMU Protein phosphatase typ	4
3.769080133	1.71	E9ARF2	tr|E9ARF2|E9ARF2_LEIMU Serine/threonine protein	2
3.209314186	1.7093	E9ALF6	tr|E9ALF6|E9ALF6_LEIMU Uncharacterized protein O	6
4.210989915	1.7088	E9AT45	tr|E9AT45|E9AT45_LEIMU Uncharacterized protein C	4
3.82768297	1.7088	E9ASR0	tr|E9ASR0|E9ASR0_LEIMU Uncharacterized protein C	3
3.765539303	1.7088	E9AXH4	tr|E9AXH4|E9AXH4_LEIMU Tubulin gamma chain OS=	3
2.903389293	1.7086	E9AVK5	tr|E9AVK5|E9AVK5_LEIMU Uncharacterized protein C	3
3.635114212	1.7084	E9AR65	tr|E9AR65|E9AR65_LEIMU Putative ATP-dependent F	10
3.497888802	1.7082	E9AYZ0	tr|E9AYZ0|E9AYZ0_LEIMU Putative 2-oxoglutarate de	16
3.589897671	1.7082	E9B2Q9	tr|E9B2Q9|E9B2Q9_LEIMU Cleavage and polyadenyla	10
4.086100756	1.7079	E9B187	tr|E9B187|E9B187_LEIMU Putative valyl-tRNA synthe	6
3.478080953	1.7076	E9AK86	tr|E9AK86|E9AK86_LEIMU Acyltransferase-like prote	4
4.189809053	1.7075	E9B217	tr|E9B217|E9B217_LEIMU Uncharacterized protein C	3
4.32524039	1.7072	E9AY61	tr|E9AY61|E9AY61_LEIMU Putative asparagine synth	10
3.834363208	1.7071	E9ASP5;E9A	tr|E9ASP5|E9ASP5_LEIMU Putative 40S ribosomal pr	7
3.280167422	1.7068	E9AW65	tr|E9AW65|E9AW65_LEIMU Uncharacterized protein	2
3.533928863	1.7067	E9B1T6	tr|E9B1T6|E9B1T6_LEIMU Uncharacterized protein O	6
3.359570713	1.7067	E9B076	tr|E9B076|E9B076_LEIMU Uncharacterized protein C	2
3.992927785	1.7067	E9B0B3	tr|E9B0B3|E9B0B3_LEIMU Glutamate dehydrogenase	11
3.866028947	1.7066	E9AYH5	tr|E9AYH5|E9AYH5_LEIMU Putative mitotubule-asso	18
4.191958121	1.7064	Q5MY89;E9	tr|Q5MY89|Q5MY89_LEIAM Replication protein A su	5
3.899569639	1.7063	E9AQ99;E9E	tr|E9AQ99|E9AQ99_LEIMU 60S ribosomal protein L6	5
4.711531228	1.7063	A0A0C5D11	tr|A0A0C5D115|A0A0C5D115_LEIAM Seryl-tRNA syn	2
3.55309794	1.7058	E9AQU6	tr|E9AQU6|E9AQU6_LEIMU DNA-directed rna polym	13
3.120259958	1.7057	E9AYN0	tr|E9AYN0|E9AYN0_LEIMU Uncharacterized protein C	3
4.036534865	1.7045	E9AQQ1;E9A	tr|E9AQQ1|E9AQQ1_LEIMU Putative flagellar calcium	4
4.189368641	1.7044	E9B262	tr|E9B262|E9B262_LEIMU Serine/threonine-protein	3
2.880913774	1.7039	E9AN90	tr|E9AN90|E9AN90_LEIMU Uncharacterized protein C	2
3.575176826	1.7037	E9B5D6	tr|E9B5D6|E9B5D6_LEIMU Uncharacterized protein C	3
4.01436453	1.7035	E9ARX7	tr|E9ARX7|E9ARX7_LEIMU Kinesin-like protein OS=Le	5
3.830659988	1.7027	E9ALJ6	tr|E9ALJ6|E9ALJ6_LEIMU Uncharacterized protein OS	2
3.659359168	1.7027	E9AWJ8	tr|E9AWJ8|E9AWJ8_LEIMU Uncharacterized protein	2
3.870434355	1.7023	E9AZU7	tr|E9AZU7|E9AZU7_LEIMU Putative phenylalanine-4-	8
3.351457178	1.7022	E9B442	tr|E9B442|E9B442_LEIMU Uncharacterized protein C	6
3.875785031	1.7022	E9AMC3	tr|E9AMC3|E9AMC3_LEIMU Uncharacterized protein	2
3.744095357	1.702	E9AJF2	tr|E9AJF2|E9AJF2_LEIMU Mitochondrial processing p	7
4.1187152	1.7016	E9ATU8	tr|E9ATU8|E9ATU8_LEIMU Uncharacterized protein C	7
4.005742874	1.7015	E9B0V4	tr|E9B0V4|E9B0V4_LEIMU Putative splicing factor 3a	5
3.784947208	1.7014	E9AZ50	tr|E9AZ50|E9AZ50_LEIMU Uncharacterized protein C	6
3.002134061	1.7013	E9AR64	tr|E9AR64|E9AR64_LEIMU Uncharacterized protein C	2
3.652486158	1.7012	E9AVY2	tr|E9AVY2|E9AVY2_LEIMU Putative ATP-dependent D	7
3.916715413	1.7012	E9AV44	tr|E9AV44|E9AV44_LEIMU Proteasome regulatory nc	15
3.796792332	1.7008	E9AR97	tr|E9AR97|E9AR97_LEIMU RNA cytidine acetyltransfe	15
3.858042253	1.7008	E9B0H1;E9A	tr|E9B0H1|E9B0H1_LEIMU Putative aspartyl-tRNA sy	17

4.058919928	1.7007	E9AZ73	tr|E9AZ73|E9AZ73_LEIMU Putative eukaryotic transla	16
4.41061396	1.7005	E9B2Z9	tr|E9B2Z9|E9B2Z9_LEIMU Uncharacterized protein O	8
3.707026945	1.7005	E9AT28	tr|E9AT28|E9AT28_LEIMU Nuclear pore protein OS=L	6
4.117273179	1.7005	E9B0N0	tr|E9B0N0|E9B0N0_LEIMU Putative ubiquitin fusion d	2
3.532471632	1.7004	E9ALQ4	tr|E9ALQ4|E9ALQ4_LEIMU Uncharacterized protein O	3
3.534217748	1.7002	E9B405;A0A	tr|E9B405|E9B405_LEIMU Thiamine biosynthesis-like	8
4.71625998	1.6999	E9ATP9	tr|E9ATP9|E9ATP9_LEIMU Ribosome assembly factor	5
3.54721785	1.6999	E9AU22	tr|E9AU22|E9AU22_LEIMU Uncharacterized protein O	9
3.581925412	1.6997	E9ASW4	tr|E9ASW4|E9ASW4_LEIMU Putative T-lymphocyte tr	3
3.356524826	1.6997	E9AT27	tr|E9AT27|E9AT27_LEIMU Chromatin assembly facto	2
4.083565883	1.6996	E9B545	tr|E9B545|E9B545_LEIMU Uncharacterized protein C	9
3.613617804	1.6992	E9ARD8	tr|E9ARD8|E9ARD8_LEIMU Uncharacterized protein O	14
3.95468071	1.6991	Q9GRU3;E9A	tr|Q9GRU3|Q9GRU3_LEIME Mitogen-activated prote	2
4.451848157	1.6986	E9B757	tr|E9B757|E9B757_LEIMU Putative GTP-binding prot	3
3.713092967	1.6984	E9AJY5	tr|E9AJY5|E9AJY5_LEIMU Putative cAMP-specific pho	2
3.599152518	1.6982	E9AV43	tr|E9AV43|E9AV43_LEIMU Uncharacterized protein O	2
3.658113054	1.6979	E9APC2	tr|E9APC2|E9APC2_LEIMU Putative AMP deaminase	3
3.15628655	1.6975	E9AJX2	tr|E9AJX2|E9AJX2_LEIMU Uncharacterized protein OS	5
4.120883977	1.6975	E9AYR1	tr|E9AYR1|E9AYR1_LEIMU Cytochrome p450-like pro	3
4.268224121	1.6975	E9B2N5	tr|E9B2N5|E9B2N5_LEIMU Putative exportin 1 OS=Le	4
3.776056938	1.6973	E9ARD0;I3V	tr|E9ARD0|E9ARD0_LEIMU Elongation factor 1-alpha	29
3.823768626	1.6972	E9AX49	tr|E9AX49|E9AX49_LEIMU 3-oxoacyl-(Acyl-carrier pro	4
3.525864022	1.6966	E9ARY6	tr|E9ARY6|E9ARY6_LEIMU Uncharacterized protein C	2
4.38020925	1.6966	E9AT17	tr|E9AT17|E9AT17_LEIMU Uncharacterized protein C	2
3.784521304	1.6965	E9B6F2	tr|E9B6F2|E9B6F2_LEIMU Putative acyl-CoA dehydro	5
3.550489851	1.6964	E9B346	tr|E9B346|E9B346_LEIMU Uncharacterized protein C	3
4.396286357	1.6961	E9APA2	tr|E9APA2|E9APA2_LEIMU Uncharacterized protein O	6
3.644134855	1.6961	E9B050	tr|E9B050|E9B050_LEIMU Uncharacterized protein C	3
3.555283624	1.696	E9AVR1	tr|E9AVR1|E9AVR1_LEIMU Putative NADH-cytochrom	11
3.932119981	1.6956	E9B5I1	tr|E9B5I1|E9B5I1_LEIMU Putative dynein heavy chair	34
3.507783633	1.6956	E9AY82	tr|E9AY82|E9AY82_LEIMU Uncharacterized protein C	2
3.276576657	1.6954	E9B3K7	tr|E9B3K7|E9B3K7_LEIMU Uncharacterized protein C	3
3.44458451	1.6953	O96795;E9A	tr|O96795|O96795_LEIME Dolichol-phosphate mann	4
3.544150651	1.6952	E9AVZ4	tr|E9AVZ4|E9AVZ4_LEIMU Uncharacterized protein O	7
4.146202717	1.6949	E9AYK8	tr|E9AYK8|E9AYK8_LEIMU Putative nitrilase OS=Leish	3
3.857398467	1.6949	Q2PQD2;E9A	tr|Q2PQD2|Q2PQD2_LEIME MPK10 OS=Leishmania r	9
3.544288434	1.6948	E9ARV1	tr|E9ARV1|E9ARV1_LEIMU Putative gamma-glutamyl	5
3.766078803	1.6946	E9AX41	tr|E9AX41|E9AX41_LEIMU Uncharacterized protein C	3
3.480974607	1.6944	E9B578;E9B	tr|E9B578|E9B578_LEIMU Putative ribosomal protein	15
3.826532987	1.6943	E9AL72	tr|E9AL72|E9AL72_LEIMU Putative electron transfer	9
3.813011283	1.6942	E9B494	tr|E9B494|E9B494_LEIMU Isocitrate dehydrogenase	8
3.322207996	1.6933	E9ANF3	tr|E9ANF3|E9ANF3_LEIMU Uncharacterized protein O	5
3.87176898	1.6932	E9AZT2	tr|E9AZT2|E9AZT2_LEIMU Putative electron-transfer-	8
3.813572318	1.6932	E9AL45	tr|E9AL45|E9AL45_LEIMU Putative ATP-dependent D	11
3.606402958	1.6931	E9APY0	tr|E9APY0|E9APY0_LEIMU Serine hydroxymethyltran	15
3.105203987	1.693	E9B6F3	tr|E9B6F3|E9B6F3_LEIMU Galactokinase-like protein	11
3.047881126	1.6929	E9B6B0	tr|E9B6B0|E9B6B0_LEIMU Uncharacterized protein C	2
3.692374084	1.6929	E9AMI7	tr|E9AMI7|E9AMI7_LEIMU Uncharacterized protein O	2
3.568744909	1.6927	E9B2F8	tr|E9B2F8|E9B2F8_LEIMU Protein SEY1 homolog OS=	5
3.706945344	1.6925	E9B4S2	tr|E9B4S2|E9B4S2_LEIMU Putative myosin IB heavy c	3

4.070876999	1.6924	E9AUS9	tr|E9AUS9|E9AUS9_LEIMU Putative axoneme central	11
3.778702853	1.6922	E9AS18	tr|E9AS18|E9AS18_LEIMU DNA-directed RNA polyme	2
3.900030404	1.6922	E9AQD9	tr|E9AQD9|E9AQD9_LEIMU Putative glutaminyl-tRN/	5
3.883156187	1.6921	E9AWF1	tr|E9AWF1|E9AWF1_LEIMU Putative dynein heavy ch	30
3.58558863	1.6918	E9AQU7	tr|E9AQU7|E9AQU7_LEIMU Uncharacterized protein	5
3.566618052	1.6917	E9ATL1	tr|E9ATL1|E9ATL1_LEIMU Uncharacterized protein O	8
3.628630273	1.6917	E9B296	tr|E9B296|E9B296_LEIMU Putative serine/threonine-	3
3.658709415	1.6915	E9B066	tr|E9B066|E9B066_LEIMU Uncharacterized protein C	5
4.499258677	1.6914	E9AN41	tr|E9AN41|E9AN41_LEIMU Protein transport protein	3
3.341332324	1.6914	E9B1W4;E9I	tr|E9B1W4|E9B1W4_LEIMU Thiolase protein-like pro	14
3.771494936	1.6913	E9ASJ4	tr|E9ASJ4|E9ASJ4_LEIMU Uncharacterized protein OS	2
4.078829184	1.6913	E9AYA3;Q25	tr|E9AYA3|E9AYA3_LEIMU Heat shock protein 70-rela	14
3.69606368	1.6908	E9AJD4	tr|E9AJD4|E9AJD4_LEIMU Putative fatty acyl CoA syn	9
3.45598993	1.6907	E9AUT2	tr|E9AUT2|E9AUT2_LEIMU Uncharacterized protein (	2
3.666010561	1.6903	E9ASE2	tr|E9ASE2|E9ASE2_LEIMU eIF3-interacting protein-lik	11
3.878726815	1.6903	E9B1G8	tr|E9B1G8|E9B1G8_LEIMU Uncharacterized protein (	2
3.918934585	1.6903	E9APV2	tr|E9APV2|E9APV2_LEIMU Putative dynein heavy cha	10
4.641377079	1.6899	E9B2G0	tr|E9B2G0|E9B2G0_LEIMU Proteasome regulatory nc	9
3.965486276	1.6897	E9AN04	tr|E9AN04|E9AN04_LEIMU Uncharacterized protein (	11
3.365313112	1.6896	E9ALD8;E9B	tr|E9ALD8|E9ALD8_LEIMU 40S ribosomal protein S19	2
4.030854846	1.6894	E9AV93	tr|E9AV93|E9AV93_LEIMU T-complex protein 1 subu	24
3.441851091	1.6893	E9ARW1	tr|E9ARW1|E9ARW1_LEIMU Putative fibrillarin OS=Le	6
5.156014434	1.6892	E9AN95	tr|E9AN95|E9AN95_LEIMU Putative nuclear transpor	2
4.361848495	1.6891	E9ATW0	tr|E9ATW0|E9ATW0_LEIMU Putative adaptin OS=Lei:	2
3.852794007	1.6891	E9APB6	tr|E9APB6|E9APB6_LEIMU Uncharacterized protein (	2
3.506392826	1.6889	E9AQT4	tr|E9AQT4|E9AQT4_LEIMU Uncharacterized protein (	7
3.90282965	1.6887	E9AXW8	tr|E9AXW8|E9AXW8_LEIMU Putative serine/threonir	3
4.021037763	1.6886	E9ARK4	tr|E9ARK4|E9ARK4_LEIMU Uncharacterized protein (	10
3.659861748	1.6886	E9B384	tr|E9B384|E9B384_LEIMU Uncharacterized protein C	21
3.568392016	1.6885	E9AL78	tr|E9AL78|E9AL78_LEIMU Calcium-transporting ATPa	2
3.780087658	1.6884	E9AQL0	tr|E9AQL0|E9AQL0_LEIMU Uncharacterized protein (	5
3.85091441	1.6883	E9AQ77	tr|E9AQ77|E9AQ77_LEIMU Uncharacterized protein (	4
3.495670268	1.6881	Q0PKV8;E9E	tr|Q0PKV8|Q0PKV8_LEIME Putative mitogen-activate	2
3.915674659	1.6874	E9B3U9	tr|E9B3U9|E9B3U9_LEIMU Putative pumilio protein (	8
3.7134912	1.6872	E9B0J0	tr|E9B0J0|E9B0J0_LEIMU Uncharacterized protein OS	2
3.587674576	1.6871	E9ASS0	tr|E9ASS0|E9ASS0_LEIMU Uncharacterized protein O	4
3.688469001	1.6863	E9AYS6	tr|E9AYS6|E9AYS6_LEIMU Kinetoplast-associated pro	2
3.898691582	1.6859	E9ATS7	tr|E9ATS7|E9ATS7_LEIMU Methylthioribose-1-phosp	5
3.877156629	1.6859	E9AV27	tr|E9AV27|E9AV27_LEIMU Uncharacterized protein (	4
3.802267119	1.6856	E9ANZ0	tr|E9ANZ0|E9ANZ0_LEIMU Putative alanine aminotra	6
4.4794613	1.6852	E9API2	tr|E9API2|E9API2_LEIMU Ubiquitin-conjugating enzyı	3
3.985256815	1.6849	E9AXH6	tr|E9AXH6|E9AXH6_LEIMU Putative dynein heavy cha	67
3.312544883	1.6843	E9API7	tr|E9API7|E9API7_LEIMU Mitochondrial DNA polyme	4
3.863238311	1.6837	E9ASC2	tr|E9ASC2|E9ASC2_LEIMU Putative PUF1 OS=Leishma	5
3.661205281	1.6837	E9AQN2	tr|E9AQN2|E9AQN2_LEIMU Putative ubiquitin hydrol	2
3.39455124	1.6836	E9B5S1	tr|E9B5S1|E9B5S1_LEIMU Putative short chain dehyc	7
3.501121888	1.6836	E9B6Y4	tr|E9B6Y4|E9B6Y4_LEIMU Histone-lysine N-methyltra	7
3.817456674	1.6833	E9AP13	tr|E9AP13|E9AP13_LEIMU NADH:flavin oxidoreducta	9
3.612562318	1.6832	E9AW77	tr|E9AW77|E9AW77_LEIMU Uncharacterized protein	3
3.051961113	1.6831	E9ANA0	tr|E9ANA0|E9ANA0_LEIMU Uncharacterized protein	3

3.782609164	1.6831	E9B0H3	tr|E9B0H3|E9B0H3_LEIMU Putative bystin OS=Leishn	4
3.143735701	1.6831	E9AXU7;E9A	tr|E9AXU7|E9AXU7_LEIMU Succinate--CoA ligase [AD	14
3.74054107	1.6828	E9AZ16	tr|E9AZ16|E9AZ16_LEIMU Putative intraflagellar tran	5
4.017019584	1.6828	E9AQ29	tr|E9AQ29|E9AQ29_LEIMU Ribonucleoprotein p18, n	8
3.801754869	1.6823	E9B453	tr|E9B453|E9B453_LEIMU Uncharacterized protein C	3
3.791592732	1.6822	E9ANV5	tr|E9ANV5|E9ANV5_LEIMU Putative ornithine decark	8
3.758843567	1.6817	Q8MTW1;E9	tr|Q8MTW1|Q8MTW1_LEIAM Meta 2 protein OS=Lei	9
3.885604632	1.6813	E9B2J6	tr|E9B2J6|E9B2J6_LEIMU Uncharacterized protein OS	7
3.750153224	1.6811	E9AWS5	tr|E9AWS5|E9AWS5_LEIMU Putative dynein arm ligh	4
4.414981973	1.6805	E9B0U8	tr|E9B0U8|E9B0U8_LEIMU Uncharacterized protein (	2
3.184389709	1.6803	E9B547	tr|E9B547|E9B547_LEIMU Uncharacterized protein C	9
3.983337435	1.6797	E9AZ28	tr|E9AZ28|E9AZ28_LEIMU Uncharacterized protein C	7
3.642335997	1.6795	E9AQ80	tr|E9AQ80|E9AQ80_LEIMU Uncharacterized protein (	4
3.90557807	1.6792	E9B3Z6	tr|E9B3Z6|E9B3Z6_LEIMU Uncharacterized protein O	6
4.157578905	1.6791	E9AK27	tr|E9AK27|E9AK27_LEIMU Adenylosuccinate lyase OS	2
3.788342717	1.6791	E9AV48	tr|E9AV48|E9AV48_LEIMU Uncharacterized protein (	28
3.915275626	1.6789	E9B5M6	tr|E9B5M6|E9B5M6_LEIMU Coatomer subunit alpha	19
3.103933833	1.6785	E9B104	tr|E9B104|E9B104_LEIMU Uncharacterized protein C	2
3.174738395	1.6784	E9AM90	tr|E9AM90|E9AM90_LEIMU Uncharacterized protein	2
4.582962319	1.6782	E9ALH4	tr|E9ALH4|E9ALH4_LEIMU Vacuolar protein sorting-a	6
4.304071516	1.6779	E9AT66	tr|E9AT66|E9AT66_LEIMU Uncharacterized protein C	5
3.797334978	1.6779	E9B5S5	tr|E9B5S5|E9B5S5_LEIMU Putative ATP-dependent D	16
3.610598678	1.6772	E9ARC2	tr|E9ARC2|E9ARC2_LEIMU Uncharacterized protein (	19
4.000965467	1.6769	E9B199	tr|E9B199|E9B199_LEIMU Putative glutamyl-tRNA sy	11
4.441137783	1.6767	Q95PT4;E9A	tr|Q95PT4|Q95PT4_LEIME Myo-inositol-1-phosphate	4
3.852243928	1.6764	E9AN29	tr|E9AN29|E9AN29_LEIMU Putative nucleolar protei	17
3.178623441	1.6761	Q06309;E9A	sp|Q06309|CRK1_LEIME Cell division protein kinase 2	2
3.767455733	1.6757	E9B5R2	tr|E9B5R2|E9B5R2_LEIMU 60S ribosomal protein L30	4
4.061595341	1.6756	E9AVE1	tr|E9AVE1|E9AVE1_LEIMU Putative RNA helicase OS=	10
3.666061141	1.6754	E9B709	tr|E9B709|E9B709_LEIMU Uncharacterized protein C	2
3.779505204	1.6752	E9B2R2	tr|E9B2R2|E9B2R2_LEIMU Uncharacterized protein C	8
4.210042366	1.675	E9ARB5	tr|E9ARB5|E9ARB5_LEIMU Putative zinc-finger protei	8
4.024709184	1.675	E9B0M9	tr|E9B0M9|E9B0M9_LEIMU Uncharacterized protein	2
3.556892952	1.6749	E9B490	tr|E9B490|E9B490_LEIMU Uncharacterized protein C	6
4.06779514	1.6748	E9APE3	tr|E9APE3|E9APE3_LEIMU Adenylosuccinate synthet	15
4.152420091	1.6742	E9B343	tr|E9B343|E9B343_LEIMU Putative cystathionine bet	5
3.689666369	1.674	E9AKI9	tr|E9AKI9|E9AKI9_LEIMU S-methyl-5-thioadenosine p	2
4.566565667	1.6739	E9AYL7	tr|E9AYL7|E9AYL7_LEIMU Putative sec1 family transp	3
3.712676746	1.6738	E9B0G2	tr|E9B0G2|E9B0G2_LEIMU Putative phosphatase 2C (	4
4.513638262	1.6735	E9AZK1	tr|E9AZK1|E9AZK1_LEIMU Katanin p60 ATPase-conta	2
4.257974286	1.6733	E9AUY8	tr|E9AUY8|E9AUY8_LEIMU Uncharacterized protein (	2
3.947303098	1.6732	E9B3L2;P27	tr|E9B3L2|E9B3L2_LEIMU Heat shock protein 83-1 OS	46
3.421655257	1.6728	E9AJP3	tr|E9AJP3|E9AJP3_LEIMU Glycerol-3-phosphate acyl	7
3.529355637	1.6727	E9B4E9	tr|E9B4E9|E9B4E9_LEIMU Uncharacterized protein O	8
3.679426764	1.6726	E9AS63	tr|E9AS63|E9AS63_LEIMU Proteasome regulatory no	13
3.504666673	1.6722	E9ANA1	tr|E9ANA1|E9ANA1_LEIMU Putative small GTP-bindir	8
4.121982328	1.672	E9B6I9	tr|E9B6I9|E9B6I9_LEIMU Putative ATP-dependent RN	25
4.036190518	1.6719	E9AXF3	tr|E9AXF3|E9AXF3_LEIMU Uncharacterized protein C	8
3.620151198	1.6718	E9AK37	tr|E9AK37|E9AK37_LEIMU Uncharacterized protein C	10
4.007134231	1.6717	E9AYQ2;A0A	tr|E9AYQ2|E9AYQ2_LEIMU Putative glutamate 5-kina	2

2.871042804	1.6716	E9AVQ5	tr|E9AVQ5|E9AVQ5_LEIMU Uncharacterized protein	2
3.612405276	1.6711	E9AL20	tr|E9AL20|E9AL20_LEIMU Pitrilysin-like metalloprote	8
3.794559063	1.6701	E9B4B4	tr|E9B4B4|E9B4B4_LEIMU Putative translation initiat	12
4.032079561	1.6691	E9ALU2	tr|E9ALU2|E9ALU2_LEIMU Putative carnitine/choline	8
4.234052542	1.6691	E9B2P6	tr|E9B2P6|E9B2P6_LEIMU Uncharacterized protein C	7
3.142252884	1.6689	E9ALL9;E9A	tr|E9ALL9|E9ALL9_LEIMU Uncharacterized protein OS	2
3.835549034	1.6689	E9AP62;E8N	tr|E9AP62|E9AP62_LEIMU Tubulin alpha chain OS=Le	26
3.942750438	1.6689	E9AVC8	tr|E9AVC8|E9AVC8_LEIMU Uncharacterized protein (	2
4.042752779	1.6687	E9AQV6;H6	tr|E9AQV6|E9AQV6_LEIMU Paraflagellar rod protein	2
2.410630757	1.6683	E9B452	tr|E9B452|E9B452_LEIMU Kinesin-like protein OS=Le	3
3.75541826	1.668	E9AY42	tr|E9AY42|E9AY42_LEIMU Uncharacterized protein C	3
3.37548014	1.6673	E9B2Y6	tr|E9B2Y6|E9B2Y6_LEIMU ABC transporter-like prote	4
4.221954317	1.6672	E9ARY4	tr|E9ARY4|E9ARY4_LEIMU Intraflagellar transport pr	10
3.963147644	1.6672	E9ATD8	tr|E9ATD8|E9ATD8_LEIMU Putative cysteine synthas	6
3.746219679	1.667	E9B2E2	tr|E9B2E2|E9B2E2_LEIMU Uncharacterized protein O	6
3.813922823	1.6669	E9AUW3	tr|E9AUW3|E9AUW3_LEIMU Uncharacterized protei	2
3.412991401	1.6668	E9AXH9	tr|E9AXH9|E9AXH9_LEIMU Uncharacterized protein (	2
4.053772583	1.6668	E9AKB4	tr|E9AKB4|E9AKB4_LEIMU Serine/threonine-protein	14
3.860365201	1.6661	E9AS95	tr|E9AS95|E9AS95_LEIMU Uncharacterized protein O	2
3.876601592	1.6658	E9APF9	tr|E9APF9|E9APF9_LEIMU Uncharacterized protein C	4
4.105579229	1.6658	E9AQF8	tr|E9AQF8|E9AQF8_LEIMU Uncharacterized protein (	2
3.57430139	1.6658	E9AVT0	tr|E9AVT0|E9AVT0_LEIMU Uncharacterized protein C	3
4.166555055	1.6656	E9ATG6	tr|E9ATG6|E9ATG6_LEIMU Putative glycyl tRNA synth	13
3.90910197	1.6655	E9B4C1	tr|E9B4C1|E9B4C1_LEIMU Transcription elongation f	7
3.230744006	1.6653	E9B162	tr|E9B162|E9B162_LEIMU Uncharacterized protein C	2
3.876129945	1.6645	E9B5L4	tr|E9B5L4|E9B5L4_LEIMU Serine/threonine-protein p	3
4.34584526	1.6642	E9AJK0	tr|E9AJK0|E9AJK0_LEIMU Proteasome regulatory nor	18
3.747167085	1.6639	E9AJW0	tr|E9AJW0|E9AJW0_LEIMU Uncharacterized protein	3
3.73650506	1.6638	E9AWI0	tr|E9AWI0|E9AWI0_LEIMU Pyridoxal phosphate hom	4
2.902569991	1.6629	E9AX71	tr|E9AX71|E9AX71_LEIMU Putative fatty-acid desatu	2
3.564698677	1.6629	E9ATU2	tr|E9ATU2|E9ATU2_LEIMU Uncharacterized protein (	4
3.486452752	1.6626	E9AU68	tr|E9AU68|E9AU68_LEIMU Putative ubiquitin-protei	3
3.547288563	1.6626	E9AMD0	tr|E9AMD0|E9AMD0_LEIMU Putative ribose-phospha	5
4.341378233	1.6623	E9B638	tr|E9B638|E9B638_LEIMU Uncharacterized protein C	3
3.218792566	1.6622	E9AJK4	tr|E9AJK4|E9AJK4_LEIMU Putative RNA-editing comp	3
3.292506081	1.662	E9B4N6	tr|E9B4N6|E9B4N6_LEIMU ABC transporter-like prot	4
3.741340805	1.6617	E9ALG1	tr|E9ALG1|E9ALG1_LEIMU Uncharacterized protein (	19
3.894735195	1.6616	E9AY72	tr|E9AY72|E9AY72_LEIMU Uncharacterized protein C	6
3.170132275	1.6614	E9B474	tr|E9B474|E9B474_LEIMU Uncharacterized protein C	2
3.733800402	1.6614	E9B6B5	tr|E9B6B5|E9B6B5_LEIMU Putative aminopeptidase I	4
3.62830612	1.6613	E9API0	tr|E9API0|E9API0_LEIMU Putative ras-family membe	3
4.236075597	1.6613	E9AZ29	tr|E9AZ29|E9AZ29_LEIMU Putative T-complex protei	17
3.86881653	1.6612	E8NHQ7;E9	tr|E8NHQ7|E8NHQ7_LEIMU Putative ATPase alpha su	20
3.91997376	1.6612	E9ANQ6	tr|E9ANQ6|E9ANQ6_LEIMU Putative eukaryotic relea	15
3.933964266	1.6611	E9ANT6	tr|E9ANT6|E9ANT6_LEIMU Uncharacterized protein (	3
3.851738284	1.6608	E9B2V1	tr|E9B2V1|E9B2V1_LEIMU Uncharacterized protein C	11
3.646753874	1.6606	E9AV82	tr|E9AV82|E9AV82_LEIMU Uncharacterized protein (	20
4.029967728	1.6605	E9AKE5	tr|E9AKE5|E9AKE5_LEIMU Uncharacterized protein C	3
3.617466429	1.6602	E9AX96	tr|E9AX96|E9AX96_LEIMU Uncharacterized protein C	9
3.804798755	1.6601	E9B1P3;E8N	tr|E9B1P3|E9B1P3_LEIMU Sodium stibogluconate res	8

3.823070898	1.6599	E9B2P8	tr|E9B2P8|E9B2P8_LEIMU Putative ADP-ribosylation	2
3.512026318	1.6596	E8NHF3;E8N	tr|E8NHF3|E8NHF3_LEIMU Uncharacterized protein I	2
3.75081013	1.6596	E9ASQ3	tr|E9ASQ3|E9ASQ3_LEIMU Putative N-acetyltransfera	13
4.124398351	1.6594	E9AYY8	tr|E9AYY8|E9AYY8_LEIMU Uncharacterized protein C	2
3.958318173	1.6593	E9AXL3	tr|E9AXL3|E9AXL3_LEIMU Uncharacterized protein O	5
4.05455309	1.659	E9B1B8	tr|E9B1B8|E9B1B8_LEIMU Uncharacterized protein C	11
3.88387769	1.6589	E9AMI1	tr|E9AMI1|E9AMI1_LEIMU Stress-induced protein sti	6
3.469599909	1.6584	E9AP74	tr|E9AP74|E9AP74_LEIMU Putative RNA helicase OS=	10
4.05611104	1.6581	E9ASL5	tr|E9ASL5|E9ASL5_LEIMU Putative dynein heavy chai	21
3.647645092	1.658	E9AS32	tr|E9AS32|E9AS32_LEIMU Putative ABC transporter C	7
3.587477318	1.6579	E9B2M0	tr|E9B2M0|E9B2M0_LEIMU Putative ubiquitin-conju	4
3.948068334	1.6575	E9ALA2	tr|E9ALA2|E9ALA2_LEIMU Putative ubiquitin-conjuga	2
3.045810986	1.6574	E9AZJ5	tr|E9AZJ5|E9AZJ5_LEIMU Uncharacterized protein OS	2
3.851312614	1.6573	E9AP27	tr|E9AP27|E9AP27_LEIMU Arginine N-methyltransfer	8
3.362424706	1.6568	E9AXD5	tr|E9AXD5|E9AXD5_LEIMU Uncharacterized protein C	2
3.415796141	1.6568	E9AV28	tr|E9AV28|E9AV28_LEIMU Putative RNA helicase OS=	9
3.716882897	1.6567	E9ATM7	tr|E9ATM7|E9ATM7_LEIMU Asparaginase-like protei	4
2.910920024	1.6565	E9AZ52	tr|E9AZ52|E9AZ52_LEIMU Uncharacterized protein C	2
3.736606452	1.6564	E9AYT3	tr|E9AYT3|E9AYT3_LEIMU Uncharacterized protein C	2
3.658353649	1.6563	E9AKM0	tr|E9AKM0|E9AKM0_LEIMU Uncharacterized protein	5
4.114032723	1.6556	E9AQX3	tr|E9AQX3|E9AQX3_LEIMU Uncharacterized protein	7
3.377851101	1.6555	E9ALD4	tr|E9ALD4|E9ALD4_LEIMU Uncharacterized protein C	8
4.04442878	1.6553	E9AVC0	tr|E9AVC0|E9AVC0_LEIMU Uncharacterized protein C	2
4.175069924	1.6551	E9AY66;Q86	tr|E9AY66|E9AY66_LEIMU Putative 40S ribosomal pr	6
4.119658217	1.6549	E9AP67	tr|E9AP67|E9AP67_LEIMU Putative flagellar radial sp	11
3.766484244	1.6548	E9AMC0	tr|E9AMC0|E9AMC0_LEIMU Uncharacterized protein	7
3.443122027	1.6546	E9AUK8	tr|E9AUK8|E9AUK8_LEIMU rRNA biogenesis protein-l	7
3.730331993	1.6545	E9AWZ5	tr|E9AWZ5|E9AWZ5_LEIMU Uncharacterized protein	3
3.460315575	1.654	E9AQV1	tr|E9AQV1|E9AQV1_LEIMU Uncharacterized protein	2
3.313283787	1.6538	E9APN6	tr|E9APN6|E9APN6_LEIMU Uncharacterized protein C	3
3.772320911	1.6538	E9B1R9	tr|E9B1R9|E9B1R9_LEIMU Uncharacterized protein C	14
4.234739423	1.6537	E9AN11	tr|E9AN11|E9AN11_LEIMU Uncharacterized protein C	2
3.693766415	1.6532	E9AJN3	tr|E9AJN3|E9AJN3_LEIMU Putative ATP-dependent C	18
3.66197545	1.6531	Q4KXA6;E9E	tr|Q4KXA6|Q4KXA6_LEIME Cytoplasmic dynein heavy	27
3.033602102	1.6529	E9AN17	tr|E9AN17|E9AN17_LEIMU Probable cytosolic iron-su	2
3.691554605	1.6528	E9B357	tr|E9B357|E9B357_LEIMU Uncharacterized protein C	6
3.894782896	1.6522	E9AV50	tr|E9AV50|E9AV50_LEIMU Uncharacterized protein C	5
5.101751976	1.6519	E9B314	tr|E9B314|E9B314_LEIMU Uncharacterized protein C	4
3.515240578	1.6519	E9APR7	tr|E9APR7|E9APR7_LEIMU Elongation of fatty acids p	4
3.609748299	1.6517	E9B1P8	tr|E9B1P8|E9B1P8_LEIMU Uncharacterized protein C	3
4.067295153	1.6516	E9AQC4	tr|E9AQC4|E9AQC4_LEIMU Uncharacterized protein	4
3.927200134	1.6514	E9AZG7	tr|E9AZG7|E9AZG7_LEIMU Uncharacterized protein C	3
3.657072544	1.6512	E9AVJ6	tr|E9AVJ6|E9AVJ6_LEIMU Putative GMP synthase (Pu	13
3.717508871	1.6512	E9APD4;A0A	tr|E9APD4|E9APD4_LEIMU Putative leucyl-tRNA synt	8
3.952155761	1.6511	E9ANP0	tr|E9ANP0|E9ANP0_LEIMU 40S ribosomal protein S5	4
3.673235231	1.651	E9AUN3	tr|E9AUN3|E9AUN3_LEIMU Uncharacterized protein	4
3.856085692	1.6504	E9AZ37	tr|E9AZ37|E9AZ37_LEIMU Putative arginyl-tRNA synt	20
3.567380191	1.6503	E9AUY1	tr|E9AUY1|E9AUY1_LEIMU Uncharacterized protein C	3
3.996106444	1.6502	E9APW3;Q3	tr|E9APW3|E9APW3_LEIMU Enolase OS=Leishmania	17
3.468529883	1.6501	E9AKI4	tr|E9AKI4|E9AKI4_LEIMU Uncharacterized protein OS	2

3.430437475	1.6501	E9AYF3	tr|E9AYF3|E9AYF3_LEIMU Uncharacterized protein O	2
3.901104357	1.6495	E9AZ66	tr|E9AZ66|E9AZ66_LEIMU Uncharacterized protein C	4
3.76105501	1.6494	E9B492	tr|E9B492|E9B492_LEIMU Uncharacterized protein C	11
3.732301253	1.649	E9B049	tr|E9B049|E9B049_LEIMU DNA topoisomerase 2 OS=	7
3.53576551	1.6485	E9AP79	tr|E9AP79|E9AP79_LEIMU Uncharacterized protein C	3
3.689397786	1.6481	E9B5V1	tr|E9B5V1|E9B5V1_LEIMU Uncharacterized protein C	3
3.911863494	1.6479	E9AQ59	tr|E9AQ59|E9AQ59_LEIMU Uncharacterized protein (	2
3.675835439	1.6477	E9AYX8	tr|E9AYX8|E9AYX8_LEIMU Putative small GTP-binding	9
3.448762749	1.6476	E9B028	tr|E9B028|E9B028_LEIMU Putative mitochondrial DE	8
3.40181491	1.6471	E9AZU2	tr|E9AZU2|E9AZU2_LEIMU Uncharacterized protein (	2
3.723332882	1.6471	E9AXX5	tr|E9AXX5|E9AXX5_LEIMU Uncharacterized protein C	15
4.029563748	1.647	E9B4S6	tr|E9B4S6|E9B4S6_LEIMU Putative uracil phosphorib	4
3.512322333	1.6469	E9AJJ2	tr|E9AJJ2|E9AJJ2_LEIMU Uncharacterized protein OS	3
3.455229404	1.6466	E9AQ19	tr|E9AQ19|E9AQ19_LEIMU Putative 60S ribosomal pr	8
3.688804042	1.6464	E9B204	tr|E9B204|E9B204_LEIMU Uncharacterized protein C	5
3.887351138	1.6461	E9AU29	tr|E9AU29|E9AU29_LEIMU Uncharacterized protein (	5
3.554979502	1.6461	E9B3L9	tr|E9B3L9|E9B3L9_LEIMU Putative DNA mismatch re	4
3.730598809	1.6455	E9AM28	tr|E9AM28|E9AM28_LEIMU Cofilin-like protein OS=L	8
3.977367491	1.6452	E9AVD1	tr|E9AVD1|E9AVD1_LEIMU Uncharacterized protein (	5
3.622073405	1.6449	E9AX84	tr|E9AX84|E9AX84_LEIMU Putative poly(A)-binding p	13
3.392252401	1.6448	E9ATM9	tr|E9ATM9|E9ATM9_LEIMU Putative mitochondrial in	9
3.769274654	1.6446	E9AK23	tr|E9AK23|E9AK23_LEIMU Uncharacterized protein C	5
4.124348648	1.6446	E9ASQ7	tr|E9ASQ7|E9ASQ7_LEIMU Uncharacterized protein (	4
4.192121463	1.6444	E9B605	tr|E9B605|E9B605_LEIMU Uncharacterized protein C	5
3.800988443	1.6442	E9ALU9	tr|E9ALU9|E9ALU9_LEIMU Uncharacterized protein C	18
3.401399319	1.6442	E9AZV0	tr|E9AZV0|E9AZV0_LEIMU Putative ATP-dependent F	4
3.411047352	1.6438	E9B5I7	tr|E9B5I7|E9B5I7_LEIMU Phosphatidylinositol 3-kinas	4
3.815144208	1.6436	E9B0B0	tr|E9B0B0|E9B0B0_LEIMU Putative dynein heavy cha	19
3.974252501	1.6436	E9AU23	tr|E9AU23|E9AU23_LEIMU Putative small GTPase OS	2
3.487959293	1.6431	E9AJA7	tr|E9AJA7|E9AJA7_LEIMU Uncharacterized protein O	2
3.764114607	1.6428	E9AQ94	tr|E9AQ94|E9AQ94_LEIMU Putative 40S ribosomal pr	12
3.92564188	1.6426	E9B4E6	tr|E9B4E6|E9B4E6_LEIMU Tyrosyl-DNA phosphodiest	2
5.014389813	1.6423	E9ARJ3	tr|E9ARJ3|E9ARJ3_LEIMU V-type proton ATPase subu	3
4.21651242	1.6422	E9AX72	tr|E9AX72|E9AX72_LEIMU Putative notchless homolo	2
3.51679835	1.6422	E9ANQ5	tr|E9ANQ5|E9ANQ5_LEIMU Uncharacterized protein	8
3.6166582	1.6416	E9AYV1	tr|E9AYV1|E9AYV1_LEIMU Putative calpain-like cyste	9
3.844959393	1.6411	E9AKE1;Q0C	tr|E9AKE1|E9AKE1_LEIMU Trypanothione reductase (	2
3.750090913	1.6406	E9B1B4	tr|E9B1B4|E9B1B4_LEIMU Putative CAS/CSE/importin	10
3.959862665	1.6401	E9B1A1	tr|E9B1A1|E9B1A1_LEIMU Uncharacterized protein C	2
4.152899504	1.6396	E9API9	tr|E9API9|E9API9_LEIMU Putative dynein heavy chain	69
3.582692465	1.6388	E9AVH1	tr|E9AVH1|E9AVH1_LEIMU Uncharacterized protein (	2
3.44188704	1.6387	E9AU20	tr|E9AU20|E9AU20_LEIMU Uncharacterized protein (	6
4.237643604	1.6384	E9AT90	tr|E9AT90|E9AT90_LEIMU Uncharacterized protein C	2
3.585632612	1.6382	E9AYA9	tr|E9AYA9|E9AYA9_LEIMU Uncharacterized protein C	2
3.719882067	1.6381	E9B4W0	tr|E9B4W0|E9B4W0_LEIMU Uncharacterized protein	9
3.544077104	1.6373	E9AYK5	tr|E9AYK5|E9AYK5_LEIMU Uncharacterized protein C	2
3.708210311	1.6364	E9B423	tr|E9B423|E9B423_LEIMU Nucleolar GTP-binding pro	12
3.950097569	1.6358	E9AWK3	tr|E9AWK3|E9AWK3_LEIMU Ankyrin/TPR repeat prot	6
3.277801539	1.6356	E9AKJ5	tr|E9AKJ5|E9AKJ5_LEIMU Uncharacterized protein OS	8
3.663161614	1.6355	E9AN24	tr|E9AN24|E9AN24_LEIMU Putative leucine-rich repe	4

3.96692308	1.6353	Q9BLW4;E9,	tr|Q9BLW4|Q9BLW4_LEIME GDP-mannose pyrophos	9
4.009622025	1.6348	E9ALG7	tr|E9ALG7|E9ALG7_LEIMU Putative serine/threonine	7
3.369558048	1.6347	E9AKN2	tr|E9AKN2|E9AKN2_LEIMU Uncharacterized protein (	2
4.284262636	1.6346	E9B6K5	tr|E9B6K5|E9B6K5_LEIMU Uncharacterized protein C	3
3.962619328	1.6344	E9AV58	tr|E9AV58|E9AV58_LEIMU Hypoxanthine phosphorib	6
3.485239722	1.6339	E9B730	tr|E9B730|E9B730_LEIMU Polyadenylate-binding pro	11
3.248746453	1.6339	E9B017	tr|E9B017|E9B017_LEIMU Uncharacterized protein C	3
3.764987038	1.6339	E9AJM8	tr|E9AJM8|E9AJM8_LEIMU Uncharacterized protein (	6
3.770586121	1.6338	E9ATK1;A1Y	tr|E9ATK1|E9ATK1_LEIMU Putative oxidoreductase C	8
4.211354336	1.6337	E9AS55	tr|E9AS55|E9AS55_LEIMU Putative phenylalanyl-tRN,	5
4.410215048	1.6337	E9B2N4	tr|E9B2N4|E9B2N4_LEIMU Uncharacterized protein (	7
4.098404212	1.6323	E9B4I7	tr|E9B4I7|E9B4I7_LEIMU Uncharacterized protein OS	4
3.430617719	1.6322	E9ANS0	tr|E9ANS0|E9ANS0_LEIMU Uncharacterized protein (	5
3.429048918	1.6316	E9B1G4	tr|E9B1G4|E9B1G4_LEIMU DNA-directed RNA polym	10
3.603009497	1.6316	E9AX89	tr|E9AX89|E9AX89_LEIMU Putative elongation factor	13
3.856294554	1.6316	E9B601	tr|E9B601|E9B601_LEIMU Putative short chain dehyc	3
3.828559368	1.6314	E9AZ67	tr|E9AZ67|E9AZ67_LEIMU Uncharacterized protein C	14
4.422371744	1.631	E9AZ89;A5J\	tr|E9AZ89|E9AZ89_LEIMU Putative trypanothione syı	4
3.767644872	1.6308	E9AXZ8;E9A	tr|E9AXZ8|E9AXZ8_LEIMU Putative 60S ribosomal pro	8
3.915392469	1.6306	E9B587	tr|E9B587|E9B587_LEIMU Uncharacterized protein C	2
3.725985005	1.6306	E9B5D5	tr|E9B5D5|E9B5D5_LEIMU Uncharacterized protein (	3
3.663410801	1.6304	E9B0K3	tr|E9B0K3|E9B0K3_LEIMU Uncharacterized protein C	12
2.95442041	1.63	E9B4A0	tr|E9B4A0|E9B4A0_LEIMU Putative enoyl-CoA hydrat	2
3.609810988	1.63	E9AUC7	tr|E9AUC7|E9AUC7_LEIMU Putative T-complex prote	14
3.638586279	1.6299	E9AKB8	tr|E9AKB8|E9AKB8_LEIMU Putative nucleolar RNA he	11
3.549944917	1.6298	E9B422	tr|E9B422|E9B422_LEIMU Putative ABC transporter (	8
3.512951016	1.6295	E9AQ02	tr|E9AQ02|E9AQ02_LEIMU NADH-cytochrome b5 rec	3
3.23865034	1.6294	E9AND8	tr|E9AND8|E9AND8_LEIMU Uncharacterized protein	2
3.806691736	1.6292	E9B6L3;B0F.	tr|E9B6L3|E9B6L3_LEIMU 6-phosphogluconate dehyc	10
3.910815632	1.6286	E9B0P1	tr|E9B0P1|E9B0P1_LEIMU Importin subunit alpha OS	8
3.797939883	1.6285	E9AM76	tr|E9AM76|E9AM76_LEIMU Ribosomal protein L3-lik	3
3.833597378	1.6284	E9AWE0	tr|E9AWE0|E9AWE0_LEIMU T-complex protein 1 sub	21
4.216767377	1.628	E9AZE0	tr|E9AZE0|E9AZE0_LEIMU Obg-like ATPase 1 OS=Leis	6
3.859460519	1.6279	E9AZV5	tr|E9AZV5|E9AZV5_LEIMU Haloacid dehalogenase-lik	2
3.73233259	1.6271	E9B687	tr|E9B687|E9B687_LEIMU Putative kinesin OS=Leishr	11
4.053369991	1.6268	E9AYF0	tr|E9AYF0|E9AYF0_LEIMU Putative cytochrome c oxic	7
3.541238956	1.6256	E9AKJ8	tr|E9AKJ8|E9AKJ8_LEIMU Putative paraflagellar rod p	2
3.254635462	1.6252	E9B3V2	tr|E9B3V2|E9B3V2_LEIMU 33 kDa inner dynein arm l	3
2.494206306	1.6248	E9AQN4	tr|E9AQN4|E9AQN4_LEIMU Uncharacterized protein	2
3.184139872	1.6248	E9AXS4	tr|E9AXS4|E9AXS4_LEIMU Uncharacterized protein C	2
4.285698581	1.6248	E9B229	tr|E9B229|E9B229_LEIMU Putative ADP-ribosylation	4
3.530315029	1.6246	E9ATV8	tr|E9ATV8|E9ATV8_LEIMU Putative glycosyl hydrolas	2
3.672562601	1.6241	E9AS23	tr|E9AS23|E9AS23_LEIMU Glycosomal malate dehydı	9
3.781000624	1.6238	E9ASG1	tr|E9ASG1|E9ASG1_LEIMU Uncharacterized protein (	9
3.600841185	1.6237	E9B4A1	tr|E9B4A1|E9B4A1_LEIMU Mitochondrial processing	6
3.908598457	1.6232	E9AMV9;E9,	tr|E9AMV9|E9AMV9_LEIMU Putative eukaryotic tran	10
3.350802317	1.6232	E9AV06	tr|E9AV06|E9AV06_LEIMU Uncharacterized protein (	2
3.48607948	1.6227	E9B5G5	tr|E9B5G5|E9B5G5_LEIMU Serine palmitoyltransfera:	9
3.398814395	1.6219	E9AXP7	tr|E9AXP7|E9AXP7_LEIMU Uncharacterized protein (	5
4.200832961	1.6218	E9B4Z4	tr|E9B4Z4|E9B4Z4_LEIMU Uncharacterized protein O	3

3.818393709	1.6201	E9AM57	tr|E9AM57|E9AM57_LEIMU Uncharacterized protein	2
3.640619231	1.6198	E9ALE8	tr|E9ALE8|E9ALE8_LEIMU Uncharacterized protein O	7
3.921575379	1.6198	E9AQ43	tr|E9AQ43|E9AQ43_LEIMU Uncharacterized protein (	4
4.383669822	1.6195	E9B0D0	tr|E9B0D0|E9B0D0_LEIMU Uncharacterized protein (	2
3.920562362	1.6192	E9AUU6	tr|E9AUU6|E9AUU6_LEIMU Putative 40S ribosomal p	4
4.004039147	1.6188	E9AR70	tr|E9AR70|E9AR70_LEIMU Uncharacterized protein (	5
3.843367645	1.6188	E9AKA4;C6K	tr|E9AKA4|E9AKA4_LEIMU Actin OS=Leishmania mex	11
3.331060923	1.6188	E9AL95	tr|E9AL95|E9AL95_LEIMU Flavoprotein subunit-like p	10
4.386924082	1.6184	E9AJQ4	tr|E9AJQ4|E9AJQ4_LEIMU Putative U2 splicing auxilia	5
3.895169089	1.6177	E9B435	tr|E9B435|E9B435_LEIMU RNA editing complex prote	2
3.622786425	1.6177	E9AZZ3	tr|E9AZZ3|E9AZZ3_LEIMU Uncharacterized protein O	3
3.821161709	1.6176	E9AUD5	tr|E9AUD5|E9AUD5_LEIMU Putative eukaryotic trans	17
3.724648939	1.6169	E9B6S2	tr|E9B6S2|E9B6S2_LEIMU Uncharacterized protein O	3
4.01221522	1.6168	E9B178	tr|E9B178|E9B178_LEIMU Eukaryotic translation initi	7
3.62836521	1.6168	E9AV89	tr|E9AV89|E9AV89_LEIMU Putative 60S ribosomal pr	7
3.176986448	1.6165	E9B2M1	tr|E9B2M1|E9B2M1_LEIMU Putative calpain-like cyst	3
3.339670806	1.6162	E9ASS3	tr|E9ASS3|E9ASS3_LEIMU Uncharacterized protein O	3
3.81435281	1.6158	E9AYH6	tr|E9AYH6|E9AYH6_LEIMU Uncharacterized protein (	27
3.913891088	1.6158	E9APY5	tr|E9APY5|E9APY5_LEIMU Putative tyrosyl-tRNA synt	6
3.35159955	1.6156	E9AY14	tr|E9AY14|E9AY14_LEIMU C-1-tetrahydrofolate synth	9
3.747314366	1.6155	E9AL32	tr|E9AL32|E9AL32_LEIMU Uncharacterized protein O	9
3.710983949	1.6154	E9AUB0	tr|E9AUB0|E9AUB0_LEIMU Putative prolyl oligopepti	11
3.603403095	1.6152	E9AZF7	tr|E9AZF7|E9AZF7_LEIMU Uncharacterized protein O	3
3.473930922	1.6151	E9AJQ1	tr|E9AJQ1|E9AJQ1_LEIMU Putative ABC transporter p	9
3.344876517	1.6142	E9AMV7	tr|E9AMV7|E9AMV7_LEIMU Uncharacterized protein	2
3.688069189	1.6133	E9B2T7	tr|E9B2T7|E9B2T7_LEIMU Uncharacterized protein O	2
3.895148754	1.6132	E9AZE9	tr|E9AZE9|E9AZE9_LEIMU Putative heat shock protei	10
3.95911252	1.613	E9AKN1;Q6S	tr|E9AKN1|E9AKN1_LEIMU Uncharacterized protein (	11
3.561385712	1.6129	E9AMX3	tr|E9AMX3|E9AMX3_LEIMU Putative AAA family ATP	13
3.785952909	1.6126	E9ASZ0	tr|E9ASZ0|E9ASZ0_LEIMU Putative DEAD box RNA he	10
3.653393621	1.6119	E9AZJ9	tr|E9AZJ9|E9AZJ9_LEIMU Uncharacterized protein OS	5
3.643957328	1.6114	E9AQ96	tr|E9AQ96|E9AQ96_LEIMU Putative NAD/FAD depen	3
3.99105348	1.6114	E9B5Y1;E9B	tr|E9B5Y1|E9B5Y1_LEIMU Putative casein kinase OS=	10
5.121354373	1.6111	Q0PKV3;E9A	tr|Q0PKV3|Q0PKV3_LEIME Putative glycogen synthas	7
2.938679714	1.6109	E9B1T5	tr|E9B1T5|E9B1T5_LEIMU Uncharacterized protein O	2
3.565287124	1.6105	E9B4H4;Q8I	tr|E9B4H4|E9B4H4_LEIMU Glucose-6-phosphate 1-de	5
3.896880452	1.6103	E9APW0	tr|E9APW0|E9APW0_LEIMU Uncharacterized protein	3
3.996490573	1.61	E9AK64	tr|E9AK64|E9AK64_LEIMU Pescadillo homolog OS=Le	8
3.798337055	1.6093	E9AYI0	tr|E9AYI0|E9AYI0_LEIMU Uncharacterized protein OS	6
4.422815741	1.6085	E9APV3	tr|E9APV3|E9APV3_LEIMU Uncharacterized protein (	4
3.550476646	1.6077	E9AQW2	tr|E9AQW2|E9AQW2_LEIMU Uncharacterized protei	6
3.730702446	1.6077	E9AQC2	tr|E9AQC2|E9AQC2_LEIMU Putative ubiquitin hydrol	6
4.094250508	1.6075	E9AX01	tr|E9AX01|E9AX01_LEIMU Uncharacterized protein (	12
4.433572066	1.6073	E9AQC9	tr|E9AQC9|E9AQC9_LEIMU Uncharacterized protein	3
3.609080585	1.6073	E9B235	tr|E9B235|E9B235_LEIMU Uncharacterized protein C	8
3.217133442	1.6072	E9ALL4	tr|E9ALL4|E9ALL4_LEIMU Uncharacterized protein OS	2
4.401985657	1.6066	E9AUQ2	tr|E9AUQ2|E9AUQ2_LEIMU DNAj-like protein OS=Lei	3
3.078763404	1.6065	E9AQU5	tr|E9AQU5|E9AQU5_LEIMU Uncharacterized protein	5
4.032817301	1.6056	E9AZ87	tr|E9AZ87|E9AZ87_LEIMU Paraflagellar rod protein-li	6
3.777282058	1.6055	E9ANP7	tr|E9ANP7|E9ANP7_LEIMU Uncharacterized protein (	4

3.959249567	1.6052	E9B3F4	tr|E9B3F4|E9B3F4_LEIMU Uncharacterized protein O	3
4.468938201	1.6049	E9ALV7	tr|E9ALV7|E9ALV7_LEIMU Tryparedoxin OS=Leishma	4
3.970017605	1.6046	E9B1A6	tr|E9B1A6|E9B1A6_LEIMU Uncharacterized protein C	9
3.326995661	1.604	E9API6	tr|E9API6|E9API6_LEIMU Squalene monooxygenase-l	4
4.069246713	1.6034	E9B0L0	tr|E9B0L0|E9B0L0_LEIMU Uncharacterized protein O	3
3.563499358	1.6031	E9AK29	tr|E9AK29|E9AK29_LEIMU 60S ribosomal protein L11	8
3.901599348	1.6029	E9ALE3	tr|E9ALE3|E9ALE3_LEIMU Uncharacterized protein O	2
3.599491147	1.6024	E9B620	tr|E9B620|E9B620_LEIMU Putative threonyl-tRNA syi	14
3.58460741	1.6023	E9B6Z6	tr|E9B6Z6|E9B6Z6_LEIMU Putative katanin OS=Leishı	3
2.196194194	1.6018	E9B682	tr|E9B682|E9B682_LEIMU Putative ankyrin repeat pr	2
3.769490739	1.6015	E9AXQ3	tr|E9AXQ3|E9AXQ3_LEIMU Pyruvate dehydrogenase	11
3.877992245	1.6012	E9B1Q4	tr|E9B1Q4|E9B1Q4_LEIMU Biotin/lipoate protein liga	4
3.993665455	1.6011	E9ARQ8	tr|E9ARQ8|E9ARQ8_LEIMU Putative prolyl-tRNA synt	5
3.363468349	1.5998	E9AMU9	tr|E9AMU9|E9AMU9_LEIMU Elongation factor-1 gam	11
3.871091595	1.5994	E9AXG9	tr|E9AXG9|E9AXG9_LEIMU Peptidyl-prolyl cis-trans is	6
3.05634339	1.5989	E9ATL8	tr|E9ATL8|E9ATL8_LEIMU Uncharacterized protein O	2
3.658864243	1.5977	E9B3J7	tr|E9B3J7|E9B3J7_LEIMU Uncharacterized protein OS	3
3.81717328	1.5977	E9ATZ6	tr|E9ATZ6|E9ATZ6_LEIMU Putative isoleucyl-tRNA syi	17
3.70653569	1.5975	E9AW86	tr|E9AW86|E9AW86_LEIMU Acetyl-coenzyme A synth	5
4.831275954	1.5974	E9AUV0	tr|E9AUV0|E9AUV0_LEIMU Soluble n-ethylmaleimide	5
3.561000594	1.5969	E9B4I0	tr|E9B4I0|E9B4I0_LEIMU Malate dehydrogenase OS=	3
3.735528334	1.5967	E9AXU0	tr|E9AXU0|E9AXU0_LEIMU Elongation factor 2-like p	5
3.863389224	1.5965	E9AQN5	tr|E9AQN5|E9AQN5_LEIMU Transaldolase OS=Leishn	7
3.672476833	1.5957	E9ARL9	tr|E9ARL9|E9ARL9_LEIMU Uncharacterized protein O	45
3.566330732	1.5957	E9AZ72	tr|E9AZ72|E9AZ72_LEIMU Uncharacterized protein C	3
3.216515215	1.5956	E9B5S0	tr|E9B5S0|E9B5S0_LEIMU Putative serine palmitoyltr	9
3.461553993	1.5942	E9AYT2	tr|E9AYT2|E9AYT2_LEIMU Putative methylmalonyl-co	8
3.798929104	1.5941	E9AZS9	tr|E9AZS9|E9AZS9_LEIMU Uncharacterized protein O	8
3.525362307	1.5934	E9AYC1	tr|E9AYC1|E9AYC1_LEIMU Uncharacterized protein C	2
4.149042967	1.5933	E9B2Y3	tr|E9B2Y3|E9B2Y3_LEIMU Putative ras-related protei	2
3.274601723	1.5933	E9AJU0	tr|E9AJU0|E9AJU0_LEIMU Putative quinone oxidored	3
3.72819766	1.593	E9B371	tr|E9B371|E9B371_LEIMU Putative ubiquitin hydrola	7
4.086517356	1.5928	E9ASA6	tr|E9ASA6|E9ASA6_LEIMU Peptidylprolyl isomerase C	9
3.711459835	1.5927	E9AVD4	tr|E9AVD4|E9AVD4_LEIMU Uncharacterized protein (	5
3.385043907	1.5925	E9ASH6;O0(	tr|E9ASH6|E9ASH6_LEIMU Elongation factor G, mitoc	13
4.359098038	1.5923	E9B215	tr|E9B215|E9B215_LEIMU Prostaglandin f2-alpha syn	9
3.988864631	1.5922	E9B352	tr|E9B352|E9B352_LEIMU Uncharacterized protein C	2
3.695640888	1.5917	E9AVW8	tr|E9AVW8|E9AVW8_LEIMU Putative farnesyl pyroph	4
4.130567762	1.5914	E9AUU5	tr|E9AUU5|E9AUU5_LEIMU Phosphatase-like protein	2
4.170283743	1.591	E9ARA9	tr|E9ARA9|E9ARA9_LEIMU Putative L-gulonolactone	2
2.96489605	1.5907	E9ALC3	tr|E9ALC3|E9ALC3_LEIMU Cation-transporting ATPas	8
3.478346122	1.5902	E9AVK4	tr|E9AVK4|E9AVK4_LEIMU Uncharacterized protein C	9
3.624241974	1.5899	E9ARS0	tr|E9ARS0|E9ARS0_LEIMU Uncharacterized protein C	3
3.345727677	1.5897	E9ANJ5	tr|E9ANJ5|E9ANJ5_LEIMU Tetratricopeptide repeat ('	3
3.091333642	1.5896	E9B3T0	tr|E9B3T0|E9B3T0_LEIMU Uncharacterized protein O	5
4.061429593	1.5891	E9ATK7	tr|E9ATK7|E9ATK7_LEIMU Uncharacterized protein C	23
3.965346672	1.589	E9B038	tr|E9B038|E9B038_LEIMU Uncharacterized protein C	18
3.723991608	1.5881	E9AWR8	tr|E9AWR8|E9AWR8_LEIMU Putative malic enzyme C	7
3.717944139	1.5872	E9B387	tr|E9B387|E9B387_LEIMU Serine/threonine-protein		5
3.771602533	1.5872	E9AJJ4	tr|E9AJJ4|E9AJJ4_LEIMU Uncharacterized protein OS	13

3.029130363	1.5866	E9AVJ1	tr|E9AVJ1|E9AVJ1_LEIMU Transcription factor-like pr	2
3.669082042	1.5862	E9ASQ6	tr|E9ASQ6|E9ASQ6_LEIMU Transitional endoplasmic	21
3.604270387	1.5862	E9AX88	tr|E9AX88|E9AX88_LEIMU Putative electron transfer	8
3.190909583	1.5859	E9B0K5	tr|E9B0K5|E9B0K5_LEIMU Mitochondrial oligo_U bin	5
3.368475761	1.5858	E9AYZ3	tr|E9AYZ3|E9AYZ3_LEIMU Uncharacterized protein O	2
4.045176805	1.585	E9AJY1	tr|E9AJY1|E9AJY1_LEIMU Uncharacterized protein OS	3
3.975659653	1.585	E9AS20;E9A	tr|E9AS20|E9AS20_LEIMU Putative kinesin OS=Leishr	7
3.06024738	1.5848	E9B5U2	tr|E9B5U2|E9B5U2_LEIMU Uncharacterized protein (	2
3.690245589	1.5846	E9B3G5	tr|E9B3G5|E9B3G5_LEIMU Putative myosin heavy ch	14
4.129535524	1.5836	E9ASV6	tr|E9ASV6|E9ASV6_LEIMU Putative DEAD box RNA he	2
3.752763069	1.5833	E9B6S3;A0A	tr|E9B6S3|E9B6S3_LEIMU Putative PRP8 protein hom	10
4.342767583	1.5824	E9AK52	tr|E9AK52|E9AK52_LEIMU Uncharacterized protein C	2
3.391461759	1.582	E9B6T1	tr|E9B6T1|E9B6T1_LEIMU RNA helicase OS=Leishmar	5
4.184303754	1.5817	E9B5X5	tr|E9B5X5|E9B5X5_LEIMU Uncharacterized protein C	7
3.359629256	1.5815	E9AYF9	tr|E9AYF9|E9AYF9_LEIMU Uncharacterized protein O	22
4.525653393	1.5814	E9AXZ5	tr|E9AXZ5|E9AXZ5_LEIMU Adenine phosphoribosyltr	3
4.29687879	1.5813	E9ALV8;A5J'	tr|E9ALV8|E9ALV8_LEIMU Putative tryparedoxin OS=	2
4.742804819	1.5812	E9B3I3	tr|E9B3I3|E9B3I3_LEIMU Uncharacterized protein OS	2
3.658168667	1.5812	E9B6A7	tr|E9B6A7|E9B6A7_LEIMU ATP-dependent RNA helic	3
3.38150211	1.5811	E9B119	tr|E9B119|E9B119_LEIMU Putative heat shock 70-rel	2
3.067359181	1.5804	E9ALP4	tr|E9ALP4|E9ALP4_LEIMU Putative 40S ribosomal prc	6
3.627255482	1.5799	E9B260;Q25	tr|E9B260|E9B260_LEIMU DNA-directed RNA polyme	15
3.379877421	1.5796	E9AUC5	tr|E9AUC5|E9AUC5_LEIMU Putative nuclear pore cor	8
3.556435565	1.5793	E9ASC6	tr|E9ASC6|E9ASC6_LEIMU Leucine carboxyl methyltr	4
3.692910734	1.5792	E9ARN1	tr|E9ARN1|E9ARN1_LEIMU Uncharacterized protein (	9
3.569288699	1.5788	Q9GRS9;E9E	tr|Q9GRS9|Q9GRS9_LEIME Mannose-6-phosphate isc	4
3.724167204	1.5788	E9ANS9	tr|E9ANS9|E9ANS9_LEIMU Uncharacterized protein (	3
3.97246858	1.5788	E9AZA7;E9A	tr|E9AZA7|E9AZA7_LEIMU Branched-chain-amino-ac	10
3.278795493	1.5786	E9B6R9	tr|E9B6R9|E9B6R9_LEIMU Uncharacterized protein C	4
3.56345661	1.5786	E9AQE4	tr|E9AQE4|E9AQE4_LEIMU Phosphodiesterase OS=Le	2
3.592247154	1.5778	E9AW05	tr|E9AW05|E9AW05_LEIMU Peptidyl-prolyl cis-trans	2
3.65384121	1.5777	E9ARA1	tr|E9ARA1|E9ARA1_LEIMU Putative translation initia	16
3.072152167	1.5777	E9AYK3	tr|E9AYK3|E9AYK3_LEIMU Putative GDP-mannose 4,(	2
4.42523577	1.5774	E9APT0	tr|E9APT0|E9APT0_LEIMU Putative calpain-like cyste	2
3.883771036	1.5762	E9AZM2	tr|E9AZM2|E9AZM2_LEIMU Putative dynein heavy ch	29
3.249537066	1.5761	E9B2U5	tr|E9B2U5|E9B2U5_LEIMU Uncharacterized protein (	3
4.080893506	1.5756	E9B2C5	tr|E9B2C5|E9B2C5_LEIMU Uncharacterized protein C	9
3.967854844	1.5753	E9AW29;A0.	tr|E9AW29|E9AW29_LEIMU Putative argininosuccina	6
3.401344903	1.5752	E9B2B3	tr|E9B2B3|E9B2B3_LEIMU Methylcrotonoyl-coa carb	10
3.737589064	1.5745	E9AVB6	tr|E9AVB6|E9AVB6_LEIMU Uncharacterized protein (	3
4.139046432	1.5743	E9AP81	tr|E9AP81|E9AP81_LEIMU 40S ribosomal protein S12	9
3.144197435	1.5743	E9B4T0	tr|E9B4T0|E9B4T0_LEIMU Dihydroxyacetonephospha	7
3.379845808	1.5738	Q8MPM3;E!	tr|Q8MPM3|Q8MPM3_LEIME Transketolase OS=Leis	9
3.764870241	1.5732	E9AUL9	tr|E9AUL9|E9AUL9_LEIMU Putative vesicle-fusing ATI	11
3.218958256	1.573	E9AS92	tr|E9AS92|E9AS92_LEIMU Putative cation transporte	2
3.813000158	1.5729	E9AZ36	tr|E9AZ36|E9AZ36_LEIMU Uncharacterized protein C	14
3.791963095	1.5724	E9AT50	tr|E9AT50|E9AT50_LEIMU Uncharacterized protein C	5
2.932324457	1.5722	E9B3N4	tr|E9B3N4|E9B3N4_LEIMU Uncharacterized protein (	3
3.486949073	1.5719	E9AUQ7;E9/	tr|E9AUQ7|E9AUQ7_LEIMU Calpain-like cysteine pep	12
3.713607395	1.5718	E9B1H5	tr|E9B1H5|E9B1H5_LEIMU Putative ATP-dependent I	11

3.899543406	1.5717	E9AN05	tr|E9AN05|E9AN05_LEIMU Uncharacterized protein (	2
3.84660835	1.5717	E9ATH3	tr|E9ATH3|E9ATH3_LEIMU Adenosylhomocysteinase	7
3.138274168	1.5711	E9AVB8	tr|E9AVB8|E9AVB8_LEIMU Structural maintenance o	2
3.17238606	1.5692	E9AKV2	tr|E9AKV2|E9AKV2_LEIMU Threonine ammonia-lyase	4
3.441067134	1.5686	E9AT19	tr|E9AT19|E9AT19_LEIMU Dipeptidyl-peptidase 8-like	4
4.195028177	1.5685	E9B520	tr|E9B520|E9B520_LEIMU Putative asparaginyl-tRNA	14
3.868394427	1.5685	E9AQ01	tr|E9AQ01|E9AQ01_LEIMU Uncharacterized protein (	5
3.83387523	1.5682	E9AZQ5	tr|E9AZQ5|E9AZQ5_LEIMU Ribonucleoside-diphosph	8
3.395070989	1.5676	E9B037	tr|E9B037|E9B037_LEIMU Uncharacterized protein C	3
3.528313906	1.5674	E9ASD6	tr|E9ASD6|E9ASD6_LEIMU Elongation factor 2 OS=Le	36
3.173679062	1.5674	E9ARL1	tr|E9ARL1|E9ARL1_LEIMU Putative elongation factor	19
3.160794661	1.5663	E9AW23	tr|E9AW23|E9AW23_LEIMU Putative multidrug resist	2
5.127843345	1.5658	E9B354	tr|E9B354|E9B354_LEIMU Uncharacterized protein C	2
4.012777075	1.5648	E9B1W8	tr|E9B1W8|E9B1W8_LEIMU Uncharacterized protein	3
3.30464291	1.564	E9AZB9	tr|E9AZB9|E9AZB9_LEIMU Uncharacterized protein C	3
3.463130064	1.5638	E9B1K6	tr|E9B1K6|E9B1K6_LEIMU Mevalonate kinase OS=Lei	5
3.310522103	1.5636	E9B5N0	tr|E9B5N0|E9B5N0_LEIMU Serine/threonine-protein	5
3.735888728	1.5627	E9AQ75	tr|E9AQ75|E9AQ75_LEIMU Uncharacterized protein (	5
3.449252681	1.5625	E9B083	tr|E9B083|E9B083_LEIMU Uncharacterized protein C	3
3.145356241	1.5624	E9AUZ5	tr|E9AUZ5|E9AUZ5_LEIMU Uncharacterized protein (	3
3.541832069	1.5624	E9AXI1	tr|E9AXI1|E9AXI1_LEIMU Dehydrogenase-like proteir	2
3.952795319	1.5622	E9AKP2	tr|E9AKP2|E9AKP2_LEIMU Proteasome subunit beta	2
3.199156981	1.5619	E9AK26	tr|E9AK26|E9AK26_LEIMU Putative calpain-like cyste	6
3.736897701	1.5612	E9APL2	tr|E9APL2|E9APL2_LEIMU Uncharacterized protein O	5
3.770497726	1.561	E9B2Z5	tr|E9B2Z5|E9B2Z5_LEIMU Uncharacterized protein O	15
4.243953541	1.5609	E9AS94	tr|E9AS94|E9AS94_LEIMU Uncharacterized protein C	4
4.169579272	1.5607	E9AJQ8	tr|E9AJQ8|E9AJQ8_LEIMU Putative long chain fatty A	5
3.293950508	1.5606	E9ANJ4	tr|E9ANJ4|E9ANJ4_LEIMU Uncharacterized protein O	6
3.349143677	1.5602	E9AZH5	tr|E9AZH5|E9AZH5_LEIMU Putative pantothenate kir	8
3.671459318	1.5596	E9B0I8	tr|E9B0I8|E9B0I8_LEIMU Putative histidyl-tRNA synth	7
4.040229601	1.5586	E8NHL4	tr|E8NHL4|E8NHL4_LEIMU Brix domain containing-lik	4
4.080993765	1.5585	E9B2N1	tr|E9B2N1|E9B2N1_LEIMU Putative dynein OS=Leish	9
3.763546232	1.5585	E9B288	tr|E9B288|E9B288_LEIMU Uncharacterized protein C	4
3.462528793	1.5585	Q2TUW2;E9	tr|Q2TUW2|Q2TUW2_LEIAM Inosine-5-monophosph	11
3.357226743	1.5571	E9B0N3	tr|E9B0N3|E9B0N3_LEIMU Uncharacterized protein (	5
3.607909328	1.5559	E9ATB5	tr|E9ATB5|E9ATB5_LEIMU Uncharacterized protein C	2
3.626144974	1.5558	E9B4L7	tr|E9B4L7|E9B4L7_LEIMU Putative importin beta-1 su	8
3.50181324	1.5557	E9ALC7	tr|E9ALC7|E9ALC7_LEIMU Uncharacterized protein O	3
3.685368014	1.5551	Q6TUJ5;E9B	tr|Q6TUJ5|Q6TUJ5_LEIME Arginase OS=Leishmania n	5
4.113926999	1.5548	E9B5E5	tr|E9B5E5|E9B5E5_LEIMU Uncharacterized protein O	4
3.280443733	1.5545	E9B528	tr|E9B528|E9B528_LEIMU Glycosyltransferase-like pr	5
3.382094826	1.5543	E9AKI1	tr|E9AKI1|E9AKI1_LEIMU Uncharacterized protein OS	4
3.956289479	1.5542	E9B2G1	tr|E9B2G1|E9B2G1_LEIMU Putative ATP-dependent I	17
3.779569234	1.5535	E9AVR3	tr|E9AVR3|E9AVR3_LEIMU Uncharacterized protein (	2
3.643608942	1.5533	E9AST4	tr|E9AST4|E9AST4_LEIMU Clathrin heavy chain OS=Le	44
3.456066289	1.5528	E9AMV3	tr|E9AMV3|E9AMV3_LEIMU Uncharacterized proteir	15
3.643854463	1.5526	E9B6K2	tr|E9B6K2|E9B6K2_LEIMU Putative cystathione gamr	12
3.065660563	1.552	E9AUX8	tr|E9AUX8|E9AUX8_LEIMU Putative calpain OS=Leish	4
3.881834884	1.5515	E9AP50	tr|E9AP50|E9AP50_LEIMU Uncharacterized protein C	11
3.395782465	1.5513	E9B478	tr|E9B478|E9B478_LEIMU Putative heat shock protei	26

3.86385567	1.5512	E9AQE5;B8k	tr|E9AQE5|E9AQE5_LEIMU Phosphodiesterase OS=Le	6
3.829653364	1.5501	E9ARS1	tr|E9ARS1|E9ARS1_LEIMU Putative heat shock protei	32
3.379887033	1.549	E9ALX8	tr|E9ALX8|E9ALX8_LEIMU Uncharacterized protein O	14
3.572813451	1.5476	E9B3W9	tr|E9B3W9|E9B3W9_LEIMU Cysteine conjugate beta-	7
4.110367498	1.5475	E9AZN0	tr|E9AZN0|E9AZN0_LEIMU Serine/threonine-protein	8
3.789558504	1.5463	E9AU41	tr|E9AU41|E9AU41_LEIMU Uncharacterized protein (	20
3.725859504	1.5462	E9AUS4	tr|E9AUS4|E9AUS4_LEIMU Coatomer subunit beta OS	19
3.907591842	1.5447	E9B6C8	tr|E9B6C8|E9B6C8_LEIMU Uncharacterized protein C	3
3.653824497	1.5445	E9AN39	tr|E9AN39|E9AN39_LEIMU Isocitrate dehydrogenase	20
4.138588647	1.5443	E9AWR7	tr|E9AWR7|E9AWR7_LEIMU Malic enzyme OS=Leishr	11
3.966361957	1.5439	E9AWM1	tr|E9AWM1|E9AWM1_LEIMU Putative fumarate hyd	9
3.646134298	1.5434	E9ALL7	tr|E9ALL7|E9ALL7_LEIMU Uncharacterized protein OS	6
4.115040432	1.5434	E9B6Q7	tr|E9B6Q7|E9B6Q7_LEIMU Putative 60S ribosomal pr	4
3.492568638	1.5426	E9AQL8;Q9N	tr|E9AQL8|E9AQL8_LEIMU Putative carbamoyl-phosp	54
3.637430552	1.5424	E9AU34	tr|E9AU34|E9AU34_LEIMU Uncharacterized protein (	2
3.299112069	1.5413	E9AYU0	tr|E9AYU0|E9AYU0_LEIMU Putative nucleoporin OS=	4
3.849287698	1.5409	Q4KXA7;E9A	tr|Q4KXA7|Q4KXA7_LEIME Cytoplasmic dynein heavy	28
3.634959321	1.5407	E9AV59	tr|E9AV59|E9AV59_LEIMU Xanthine phosphoribosylt	4
3.796559553	1.5404	E9AWL9	tr|E9AWL9|E9AWL9_LEIMU Uncharacterized protein	6
3.911723494	1.5403	E9B6R4	tr|E9B6R4|E9B6R4_LEIMU T-complex protein 1 subur	17
3.483579214	1.5398	E9AKU4	tr|E9AKU4|E9AKU4_LEIMU Putative lanosterol synth	7
3.891920694	1.5396	E9ARW6	tr|E9ARW6|E9ARW6_LEIMU Uncharacterized protein	9
4.004048011	1.5394	E9AZ98	tr|E9AZ98|E9AZ98_LEIMU Protein kinase-like protein	2
3.765099106	1.5378	E9B5J2	tr|E9B5J2|E9B5J2_LEIMU AP-1 complex subunit gami	6
3.940934308	1.5367	E9AWZ3	tr|E9AWZ3|E9AWZ3_LEIMU Uncharacterized protein	3
3.601358585	1.5366	E9ARS2	tr|E9ARS2|E9ARS2_LEIMU Putative pyruvate dehydro	11
4.628714378	1.5359	E9B417	tr|E9B417|E9B417_LEIMU Uncharacterized protein C	2
3.743159426	1.5352	E9AVY6	tr|E9AVY6|E9AVY6_LEIMU Alanine--tRNA ligase OS=L	26
3.704282203	1.5351	E9AKF2	tr|E9AKF2|E9AKF2_LEIMU Nucleolar GTP-binding pro	7
3.589523753	1.5351	E9AQ25	tr|E9AQ25|E9AQ25_LEIMU Putative nonspecific lipid-	2
3.795298723	1.535	E9ANI6	tr|E9ANI6|E9ANI6_LEIMU Tubulin-tyrsoine ligase-like	6
3.567435049	1.5343	E9AL37	tr|E9AL37|E9AL37_LEIMU Acetylornithine deacetylas	5
3.792947772	1.5339	E9AVT1	tr|E9AVT1|E9AVT1_LEIMU Uncharacterized protein C	6
4.041083741	1.5331	E9B4T2	tr|E9B4T2|E9B4T2_LEIMU Uncharacterized protein O	2
3.513175212	1.533	E9AS51	tr|E9AS51|E9AS51_LEIMU 4-coumarate:coa ligase-lik	10
3.552015828	1.5329	E9AM36	tr|E9AM36|E9AM36_LEIMU Uncharacterized protein	5
3.903360182	1.5324	E9B0V3	tr|E9B0V3|E9B0V3_LEIMU Uncharacterized protein C	10
4.03452519	1.5313	E9AV37	tr|E9AV37|E9AV37_LEIMU Putative ribonuclease L in	3
3.548036948	1.5311	E9AXM1	tr|E9AXM1|E9AXM1_LEIMU GTP-binding nuclear pro	9
3.536432023	1.5292	E9B4G0	tr|E9B4G0|E9B4G0_LEIMU Coatomer subunit beta OS	14
3.686682752	1.5286	E9B0Y2;A0A	tr|E9B0Y2|E9B0Y2_LEIMU Putative alcohol dehydrog	2
4.222639539	1.5283	E9ANB8	tr|E9ANB8|E9ANB8_LEIMU Uncharacterized protein (	8
3.280994373	1.5275	E9B6P8	tr|E9B6P8|E9B6P8_LEIMU Putative Gim5A protein (G	12
4.141675872	1.5268	E9AS22	tr|E9AS22|E9AS22_LEIMU Putative kinesin OS=Leishr	12
3.228802596	1.5267	E9ALR7	tr|E9ALR7|E9ALR7_LEIMU Putative glutamamyl carbo	2
3.556316471	1.5264	E9B6L6	tr|E9B6L6|E9B6L6_LEIMU Uncharacterized protein O	3
3.778343446	1.5264	E9B6Z9	tr|E9B6Z9|E9B6Z9_LEIMU Putative MCAK-like kinesir	8
3.730806496	1.5256	E9B5R1	tr|E9B5R1|E9B5R1_LEIMU Uncharacterized protein C	2
3.279151024	1.5252	E9AS62	tr|E9AS62|E9AS62_LEIMU Putative mitochondrial car	2
3.642604117	1.5239	E9B0L7	tr|E9B0L7|E9B0L7_LEIMU Putative adenosine kinase	11

4.144400724	1.5233	E9AU04	tr|E9AU04|E9AU04_LEIMU Uncharacterized protein (	3
3.528898517	1.5228	E9AKX3	tr|E9AKX3|E9AKX3_LEIMU Putative glucosamine-fruc	8
3.690882103	1.5222	E9ARX5	tr|E9ARX5|E9ARX5_LEIMU Uncharacterized protein (	4
3.275497147	1.522	E9B458	tr|E9B458|E9B458_LEIMU Uncharacterized protein C	2
3.286141198	1.5204	E9AZ08	tr|E9AZ08|E9AZ08_LEIMU Putative cysteine desulfura	7
3.60011154	1.5192	E9AZA4	tr|E9AZA4|E9AZA4_LEIMU Uncharacterized protein (	2
3.621091598	1.519	P16126;E9A	sp|P16126|DRTS_LEIAM Bifunctional dihydrofolate re	11
3.375663999	1.5161	E9AX08	tr|E9AX08|E9AX08_LEIMU Succinate dehydrogenase	17
3.306171619	1.5145	E9AMQ4	tr|E9AMQ4|E9AMQ4_LEIMU Putative leucine-rich rep	4
3.519409771	1.5144	E9AL57	tr|E9AL57|E9AL57_LEIMU Acyl-CoA dehydrogenase, r	7
4.924709749	1.512	E9AX98	tr|E9AX98|E9AX98_LEIMU Uncharacterized protein (	3
3.148043399	1.511	E9AZ83;E9A	tr|E9AZ83|E9AZ83_LEIMU Glycosomal phosphoenolp	22
3.502715109	1.5102	E9ARN6	tr|E9ARN6|E9ARN6_LEIMU UTP--glucose-1-phosphat	5
3.45081594	1.5084	E9B5U7	tr|E9B5U7|E9B5U7_LEIMU Uncharacterized protein (	18
4.062178829	1.5082	E9ASX5	tr|E9ASX5|E9ASX5_LEIMU Uncharacterized protein C	2
3.372829879	1.5071	E9AKL2;E9A	tr|E9AKL2|E9AKL2_LEIMU Putative ATPase OS=Leishr	5
3.41021872	1.5066	E9B0H2	tr|E9B0H2|E9B0H2_LEIMU Uncharacterized protein (	2
3.119484668	1.5066	E8NHE3	tr|E8NHE3|E8NHE3_LEIMU Putative 60S ribosomal pı	3
3.31942292	1.5062	E9AY75	tr|E9AY75|E9AY75_LEIMU 60S ribosomal export prot	6
3.666546393	1.5062	E9AP89	tr|E9AP89|E9AP89_LEIMU Palmitoyltransferase OS=L	2
3.676307315	1.5057	E9AWM7	tr|E9AWM7|E9AWM7_LEIMU Putative aspartate ami	5
3.470677543	1.5049	E9AZD6	tr|E9AZD6|E9AZD6_LEIMU Transcription elongation r	7
3.575239191	1.5048	E9AYE0;F8SI	tr|E9AYE0|E9AYE0_LEIMU Proline dehydrogenase OS	9
3.858878178	1.5044	E9B1P5	tr|E9B1P5|E9B1P5_LEIMU Uncharacterized protein C	3
3.990613537	1.503	E9AZX7	tr|E9AZX7|E9AZX7_LEIMU Putative hydrolase, alpha/	4
3.424946906	1.5021	E9B473	tr|E9B473|E9B473_LEIMU Succinyl-CoA:3-ketoacid-co	10
3.60230895	1.5013	E9AQZ4	tr|E9AQZ4|E9AQZ4_LEIMU Cystathionine beta-syntha	5
3.980779241	1.5009	E9AQ10	tr|E9AQ10|E9AQ10_LEIMU Putative ATP-dependent	5
3.160118317	1.4986	E9B640	tr|E9B640|E9B640_LEIMU Uncharacterized protein C	2
4.425359396	1.4982	E9B6N5	tr|E9B6N5|E9B6N5_LEIMU Uncharacterized protein (	2
3.272022366	1.4972	E9AQL3	tr|E9AQL3|E9AQL3_LEIMU Putative orotidine-5-phos	3
3.617585699	1.496	E9ATX3	tr|E9ATX3|E9ATX3_LEIMU Putative NADH dehydroge	11
3.174927524	1.4959	E9B0D7	tr|E9B0D7|E9B0D7_LEIMU Alkyl dihydroxyacetoneph	12
3.695319238	1.4949	E9AY02	tr|E9AY02|E9AY02_LEIMU Protein pelota homolog O:	3
3.482571559	1.4938	E9AQ41	tr|E9AQ41|E9AQ41_LEIMU Cytoplasmic l-asparagina:	2
3.516375553	1.4938	E9ATC6	tr|E9ATC6|E9ATC6_LEIMU 2-oxoglutarate dehydroge	21
3.98208099	1.4906	E9AQW8	tr|E9AQW8|E9AQW8_LEIMU Uncharacterized protei	4
3.136261762	1.4906	E9AT09	tr|E9AT09|E9AT09_LEIMU Glucokinase 1-like protein	2
3.200122002	1.4903	E9AKR3	tr|E9AKR3|E9AKR3_LEIMU NAD(P)-dependent steroi	6
3.901315233	1.4873	E9ANM7	tr|E9ANM7|E9ANM7_LEIMU Uncharacterized protei	4
4.044843798	1.4861	E9B3Z8	tr|E9B3Z8|E9B3Z8_LEIMU Putative peptidase M20/M	7
3.882586768	1.4858	E9AS04	tr|E9AS04|E9AS04_LEIMU Uncharacterized protein C	3
3.409392289	1.4851	E9AQM8	tr|E9AQM8|E9AQM8_LEIMU Uncharacterized protei	4
3.675034083	1.4848	E9B2B4	tr|E9B2B4|E9B2B4_LEIMU Uncharacterized protein C	2
3.66431041	1.4847	E9AM00	tr|E9AM00|E9AM00_LEIMU Putative U-box domain p	6
3.735113649	1.4846	E9B2K8	tr|E9B2K8|E9B2K8_LEIMU Uncharacterized protein C	6
3.266073604	1.484	E9AQA0	tr|E9AQA0|E9AQA0_LEIMU NAD-specific glutamate c	41
3.719766804	1.4833	E9AYX1	tr|E9AYX1|E9AYX1_LEIMU Uncharacterized protein C	3
3.434492864	1.4833	E9ANP4	tr|E9ANP4|E9ANP4_LEIMU Pyruvate, phosphate dikiı	35
3.693050137	1.4826	E9AQI0	tr|E9AQI0|E9AQI0_LEIMU Protein tyrosine phosphata	7

4.215247963	1.4812	E9ANI1	tr|E9ANI1|E9ANI1_LEIMU Putative 14-3-3 protein OS	7
3.416068582	1.4811	E9AS74	tr|E9AS74|E9AS74_LEIMU Uncharacterized protein C	3
3.456516109	1.4803	E9AVG1	tr|E9AVG1|E9AVG1_LEIMU Putative mitochondrial st	2
3.577785549	1.4802	E9B5Q7	tr|E9B5Q7|E9B5Q7_LEIMU Uncharacterized protein (	5
3.938149917	1.4793	Q95NJ3;E8N	tr|Q95NJ3|Q95NJ3_LEIAM Activated C kinase protein	11
3.983755167	1.479	E9B604	tr|E9B604|E9B604_LEIMU Thioredoxin-like protein O	4
3.865722055	1.4777	E9AW98	tr|E9AW98|E9AW98_LEIMU Na/H antiporter-like pro	5
3.403942819	1.4762	E9B2E0	tr|E9B2E0|E9B2E0_LEIMU Uncharacterized protein O	2
3.745872704	1.476	E9AZV7	tr|E9AZV7|E9AZV7_LEIMU Haloacid dehalogenase-lik	2
3.42952895	1.4757	E9AKQ4	tr|E9AKQ4|E9AKQ4_LEIMU Putative 5-3 exonuclease	4
4.004922533	1.4754	E9ARU9	tr|E9ARU9|E9ARU9_LEIMU Uncharacterized protein (	2
4.640743964	1.4723	E9B2K9	tr|E9B2K9|E9B2K9_LEIMU Uncharacterized protein C	2
3.861641273	1.4721	E9AKY6	tr|E9AKY6|E9AKY6_LEIMU Putative deoxyribose-pho:	2
3.207359686	1.4716	E9B1K4	tr|E9B1K4|E9B1K4_LEIMU Putative ATP-binding casse	7
3.721099967	1.4713	E9ALS6	tr|E9ALS6|E9ALS6_LEIMU Uncharacterized protein O	12
3.577393187	1.4704	E9AR57	tr|E9AR57|E9AR57_LEIMU Uncharacterized protein C	5
3.592918094	1.4695	E9AXU4	tr|E9AXU4|E9AXU4_LEIMU Uncharacterized protein (	4
3.8207985	1.4677	E9B6F9	tr|E9B6F9|E9B6F9_LEIMU Uncharacterized protein O	2
3.112309866	1.4675	E9AWV7	tr|E9AWV7|E9AWV7_LEIMU Mitochondrial DNA-dire	2
3.545602543	1.4651	E9ARK6;E9A	tr|E9ARK6|E9ARK6_LEIMU Citrate synthase OS=Leish	13
4.087696314	1.4643	E9AK93	tr|E9AK93|E9AK93_LEIMU Uncharacterized protein C	2
3.012701553	1.4594	E9AZA6	tr|E9AZA6|E9AZA6_LEIMU D-lactate dehydrogenase-	8
2.868468388	1.4579	E9AQP9	tr|E9AQP9|E9AQP9_LEIMU Uncharacterized protein (	2
4.98478661	1.4578	E9ANR0	tr|E9ANR0|E9ANR0_LEIMU Putative S-phase kinase-a	3
3.652466614	1.457	E9ASS2	tr|E9ASS2|E9ASS2_LEIMU Uncharacterized protein O	6
3.410329023	1.4561	E9AYD4	tr|E9AYD4|E9AYD4_LEIMU Trifunctional enzyme alph	21
3.921960893	1.4561	E9B0I3	tr|E9B0I3|E9B0I3_LEIMU Uncharacterized protein OS	6
3.290979498	1.4501	E9ARY5	tr|E9ARY5|E9ARY5_LEIMU Uncharacterized protein C	3
3.508063666	1.4461	E9AP70	tr|E9AP70|E9AP70_LEIMU Uncharacterized protein C	6
4.010903794	1.444	E9AK51	tr|E9AK51|E9AK51_LEIMU Putative ubiquitin-conjuga	3
3.851058467	1.444	E8NHK0;E9A	tr|E8NHK0|E8NHK0_LEIMU WGS CADB00000000 dat.	3
3.659932795	1.4435	E9AVH2	tr|E9AVH2|E9AVH2_LEIMU Centromere/microtubule	9
3.615236818	1.4424	E9AVJ3	tr|E9AVJ3|E9AVJ3_LEIMU Putative heat shock protei	6
3.26497173	1.442	E9AK11	tr|E9AK11|E9AK11_LEIMU Uncharacterized protein C	2
3.363181447	1.4417	E9B4K3	tr|E9B4K3|E9B4K3_LEIMU Eukaryotic translation initi	6
2.771249525	1.4417	E9AJN2	tr|E9AJN2|E9AJN2_LEIMU Uncharacterized protein O	2
4.219059215	1.4412	E9B0K7	tr|E9B0K7|E9B0K7_LEIMU Putative rac serine-threon	3
3.424603459	1.4384	E9B2K6	tr|E9B2K6|E9B2K6_LEIMU Cyclin OS=Leishmania mex	2
3.910851847	1.4364	E9B4M4	tr|E9B4M4|E9B4M4_LEIMU Putative cysteine-leucine	2
3.424824815	1.4361	E9APZ2	tr|E9APZ2|E9APZ2_LEIMU Uncharacterized protein C	15
3.480484726	1.4358	E9ANY1;P42	tr|E9ANY1|E9ANY1_LEIMU Glucose-6-phosphate isor	10
3.933604508	1.4314	E9AR17	tr|E9AR17|E9AR17_LEIMU Uncharacterized protein C	2
3.606848818	1.4307	E9ANF6	tr|E9ANF6|E9ANF6_LEIMU Putative seryl-tRNA synth	11
3.134557764	1.4287	E9B073	tr|E9B073|E9B073_LEIMU Putative acyl-CoA dehydrc	15
3.25482459	1.428	E9AUF4	tr|E9AUF4|E9AUF4_LEIMU Uncharacterized protein (	2
3.714546426	1.4277	E9ARI8	tr|E9ARI8|E9ARI8_LEIMU Aconitate hydratase OS=Le	22
3.50208669	1.4258	E9B297	tr|E9B297|E9B297_LEIMU Putative acetyl-CoA carbo:	51
3.965285514	1.4243	E9AUR8	tr|E9AUR8|E9AUR8_LEIMU Putative calpain-like cyste	3
3.013618801	1.4236	E9AX62	tr|E9AX62|E9AX62_LEIMU Uncharacterized protein C	5
3.784893137	1.4234	E9APU2	tr|E9APU2|E9APU2_LEIMU Uncharacterized protein (	2

4.280153914	1.4223	E9AKF1	tr|E9AKF1|E9AKF1_LEIMU Uncharacterized protein C	7
3.929366551	1.4222	Q4TZP6;E9A	tr|Q4TZP6|Q4TZP6_LEIME Glutamine synthetase OS=	7
3.830447488	1.4206	E9ARD9	tr|E9ARD9|E9ARD9_LEIMU Diphosphomevalonate de	5
3.632704279	1.4185	E9AQQ4;A0	tr|E9AQQ4|E9AQQ4_LEIMU Sucrose-phosphate synt	8
3.278575223	1.4176	E9AXP3	tr|E9AXP3|E9AXP3_LEIMU Uncharacterized protein C	2
3.562818695	1.4166	E9B1L5	tr|E9B1L5|E9B1L5_LEIMU Uncharacterized protein O	5
3.287136235	1.4111	E9AZ53	tr|E9AZ53|E9AZ53_LEIMU Uncharacterized protein C	2
3.88996697	1.4105	E9AJY2;A0A	tr|E9AJY2|E9AJY2_LEIMU Elongation initiation factor	13
4.078139528	1.407	E9B400	tr|E9B400|E9B400_LEIMU Peptidyl-prolyl cis-trans is	7
3.205191977	1.4041	E9B5Z7;E9B	tr|E9B5Z7|E9B5Z7_LEIMU Putative NADH-dependent	25
2.743804551	1.4029	E9AU14	tr|E9AU14|E9AU14_LEIMU Uncharacterized protein (	2
3.041110666	1.4021	E9ATF1	tr|E9ATF1|E9ATF1_LEIMU Uncharacterized protein O	3
3.315835302	1.402	E9AT73	tr|E9AT73|E9AT73_LEIMU Succinate--CoA ligase [ADI	20
3.373413644	1.397	E9B4S7	tr|E9B4S7|E9B4S7_LEIMU Uncharacterized protein O	2
4.008882503	1.3969	E9B715	tr|E9B715|E9B715_LEIMU Uncharacterized protein C	2
2.742448565	1.3867	E9AMM4	tr|E9AMM4|E9AMM4_LEIMU DNA helicase OS=Leish	2
3.560035529	1.3859	E9AMK2	tr|E9AMK2|E9AMK2_LEIMU Uncharacterized protein	2
3.585995979	1.3848	E9B5N5	tr|E9B5N5|E9B5N5_LEIMU Uncharacterized protein (	2
3.979372698	1.377	E9AYG6	tr|E9AYG6|E9AYG6_LEIMU Uncharacterized protein (	3
3.896440723	1.3764	E9B180	tr|E9B180|E9B180_LEIMU Putative kinesin OS=Leishr	5
3.163378773	1.3761	E9B4G7	tr|E9B4G7|E9B4G7_LEIMU Uncharacterized protein (	8
3.663585982	1.3759	E9B4N0	tr|E9B4N0|E9B4N0_LEIMU Putative enoyl-[acyl-carri	2
3.065443761	1.3755	E9AM50	tr|E9AM50|E9AM50_LEIMU Uncharacterized protein	6
3.397552236	1.3714	E9B1F3	tr|E9B1F3|E9B1F3_LEIMU Peptidyl-prolyl cis-trans is	4
3.240112263	1.3704	E9AXN7	tr|E9AXN7|E9AXN7_LEIMU Uncharacterized protein (	2
3.127707078	1.3652	E9AQG7	tr|E9AQG7|E9AQG7_LEIMU Uncharacterized protein	11
3.241993554	1.3616	E9AZR1	tr|E9AZR1|E9AZR1_LEIMU Uncharacterized protein C	2
3.329756516	1.3611	E9AYH4	tr|E9AYH4|E9AYH4_LEIMU Uncharacterized protein (	4
3.660312624	1.3609	E9AP96	tr|E9AP96|E9AP96_LEIMU Oxidoreductase-like prote	3
3.696090444	1.3601	E9AV32	tr|E9AV32|E9AV32_LEIMU Cytosolic Fe-S cluster asse	2
2.881652098	1.3599	E9ASP6;Q9L	tr|E9ASP6|E9ASP6_LEIMU Fructose-bisphosphate ald	22
3.163844788	1.3588	E9B386	tr|E9B386|E9B386_LEIMU Putative chaperone protei	2
3.26015715	1.3586	E9AZ51	tr|E9AZ51|E9AZ51_LEIMU Proteasome regulatory no	4
3.520993639	1.3564	E9ATG3;E9A	tr|E9ATG3|E9ATG3_LEIMU Aminomethyltransferase	2
3.974771759	1.3545	E9ARP6	tr|E9ARP6|E9ARP6_LEIMU Phosphodiesterase OS=Le	3
3.359599945	1.349	E9B193	tr|E9B193|E9B193_LEIMU 3-hydroxy-3-methylglutar	10
4.268600668	1.3468	E9AY25	tr|E9AY25|E9AY25_LEIMU Cytosolic Fe-S cluster asse	2
3.348636895	1.3452	E9AQT2	tr|E9AQT2|E9AQT2_LEIMU Uncharacterized protein (	3
3.144492062	1.3433	E9B1B3	tr|E9B1B3|E9B1B3_LEIMU Phosphoglycerate kinase (	10
3.507160986	1.3386	E9B759	tr|E9B759|E9B759_LEIMU Putative isomerase (Isope	8
3.388654814	1.3336	E9AT13	tr|E9AT13|E9AT13_LEIMU Putative tyrosine aminotra	14
2.859654197	1.3334	E9B301	tr|E9B301|E9B301_LEIMU Small nuclear ribonucleop	4
2.966950132	1.3269	E9AVT6	tr|E9AVT6|E9AVT6_LEIMU Uncharacterized protein C	2
3.49545571	1.323	E9AZZ7	tr|E9AZZ7|E9AZZ7_LEIMU DNA topoisomerase OS=Le	4
3.311302566	1.3117	E9AQY9	tr|E9AQY9|E9AQY9_LEIMU Putative peptidase t (Pep	9
3.830822536	1.3097	E9ARZ9	tr|E9ARZ9|E9ARZ9_LEIMU Uncharacterized protein C	2
3.42113135	1.3023	E9AN37	tr|E9AN37|E9AN37_LEIMU Uncharacterized protein (	2
3.824458691	1.2957	E9AQ78	tr|E9AQ78|E9AQ78_LEIMU Peroxisomal membrane p	3
4.617429674	1.2857	E9AKT5	tr|E9AKT5|E9AKT5_LEIMU Putative deoxyuridine trip	2
3.303958836	1.2732	E9B098	tr|E9B098|E9B098_LEIMU Spindle pole body compor	2

3.978334008	1.2407	E9AXC4	tr|E9AXC4|E9AXC4_LEIMU Uncharacterized protein (	2
3.157253749	1.2238	E9AX09	tr|E9AX09|E9AX09_LEIMU Fatty acyl-CoA reductase (	6
2.784568807	1.2149	E9B5X8;E9B	tr|E9B5X8|E9B5X8_LEIMU Aldose 1-epimerase-like p	7
3.417184321	1.2137	E9ALD2	tr|E9ALD2|E9ALD2_LEIMU Uncharacterized protein (	2
3.504702608	1.2135	E8NHQ8	tr|E8NHQ8|E8NHQ8_LEIMU 3-hydroxyisobutyryl-CoA	2
2.761628483	1.2091	E9B4G6	tr|E9B4G6|E9B4G6_LEIMU Putative fatty acid desatu	4
3.11846664	1.2046	E9B2H5	tr|E9B2H5|E9B2H5_LEIMU Profilin OS=Leishmania m	6
2.645412147	1.1791	E9AQA1	tr|E9AQA1|E9AQA1_LEIMU Putative farnesyl synthet	2
2.124658496	1.0951	E9B299	tr|E9B299|E9B299_LEIMU Uncharacterized protein C	2
3.441679525	1.0491	E9B765	tr|E9B765|E9B765_LEIMU Uncharacterized protein C	4
2.950866495	1.0321	E9AL30	tr|E9AL30|E9AL30_LEIMU CDP-diacylglycerol--glycero	2
2.915697821	1.0038	E9AVX8	tr|E9AVX8|E9AVX8_LEIMU I/6 autoantigen-like prote	6
3.113271395	0.9826	E9AMS8;A7	tr|E9AMS8|E9AMS8_LEIMU Oligopeptidase b OS=Lei	2
2.614330862	0.8921	E9AMS5	tr|E9AMS5|E9AMS5_LEIMU Putative ubiquitin ligase	3

Table S3. *L. mexicana* poly(A) interactome.

Gene ID	Protein name
LmxM.01.0050	carboxylase, putative
LmxM.01.0410	hypothetical protein
LmxM.01.0470	fatty acyl CoA syntetase 1, putative
LmxM.01.0480	beta eliminating lyase,putative
LmxM.01.0490	long-chain-fatty-acid-CoA ligase, putative
LmxM.01.0520	long-chain-fatty-acid-CoA ligase, putative
LmxM.01.0540	hypothetical protein, conserved
LmxM.01.0620	Beat-regulation protein
LmxM.01.0770	Eukaryotic initiation factor 4A-1
LmxM.02.0240	glycosyltransferase (GlcNAc), putative
LmxM.02.0310	paraflagellar rod component, putative
LmxM.02.0370	proteasome regulatory non-ATPase subunit 6, putative
LmxM.02.0450	voltage-dependent anion-selective channel, putative
LmxM.02.0460	Mitochondrial outer membrane protein porin, putative
LmxM.02.0550	hypothetical protein, unknown function
LmxM.02.0670	mitochondrial carrier protein, putative
LmxM.02.0710	serine peptidase, putative
LmxM.02.0740	peptidyl dipeptidase, putative
LmxM.03.0030	D-3-phosphoglycerate dehydrogenase-like protein
LmxM.03.0040	2-aminoethylphosphonate:pyruvateaminotransferas e-like protein
LmxM.03.0230	long chain fatty Acyl CoA synthetase, putative
LmxM.03.0250	ribosomal protein L38, putative
LmxM.03.0270	FYVE zinc finger/Apolipoprotein A1/A4/E domain containing protein, putative
LmxM.03.0430	60S acidic ribosomal protein P2, putative
LmxM.03.0520	hypothetical protein, conserved
LmxM.03.0540	serine peptidase, Clan SJ, family S16, putative
LmxM.03.0600	arginine N-methyltransferase, putative
LmxM.03.0630	hypothetical protein, conserved
LmxM.03.0690	DEAD/DEAH box helicase, putative
LmxM.03.0960	hypothetical protein, conserved
LmxM.03.0970	MORN repeat, putative
LmxM.03.0980	eukaryotic translation initiation factor 2 subunit alpha
LmxM.04.0010	organelle-type calcium ATPase
LmxM.04.0030	cAMP-specific phosphodiesterase, putative
LmxM.04.0050	hypothetical protein, conserved
LmxM.04.0240	GRAM domain containing protein, putative
LmxM.04.0420	Tetratricopeptide repeat, putative
LmxM.04.0450	cysteine peptidase, Clan CA, family C2, putative
LmxM.04.0460	adenylosuccinate lyase, putative
LmxM.04.0470	60S ribosomal protein L11 (L5, L16)
LmxM.04.0550	Intraflagellar transport protein 139, putative
LmxM.04.0580	spermidine synthase, putative
LmxM.04.0660	hypothetical protein
LmxM.04.0670	hypothetical protein, conserved
LmxM.04.0680	ubiquitin-conjugating enzyme E2, putative
LmxM.04.0750	60S ribosomal protein L10, putative
LmxM.04.0810	Pescadillo N-terminus/BRCA1 C Terminus (BRCT) domain containing protein, putative
LmxM.04.0940	hypothetical protein
LmxM.04.0990	hypothetical protein, conserved
LmxM.04.1050	acyltransferase-like protein, copy 2
LmxM.04.1160	fructose-1,6-bisphosphatase, cytosolic, putative
LmxM.04.1170	Double RNA binding domain protein 3
LmxM.05.0030	small GTP-binding protein, putative

LmxM.05.0040	paraflagellar rod component par4, putative
LmxM.05.0100	phosphoprotein phosphatase, putative
LmxM.05.0140	nucleolar RNA helicase II, putative
LmxM.05.0180	dihydrolipoamide branched chain transacylase, putative
LmxM.05.0280	protein tyrosine phosphatase, putative
LmxM.05.0350	trypanothione reductase
LmxM.05.0460	GTPase, putative
LmxM.05.0490	hypothetical protein, conserved
LmxM.05.0500	ATP synthase F1, alpha subunit, putative
LmxM.05.0780	hypothetical protein, conserved
LmxM.05.0830	methylthioadenosine phosphorylase, putative
LmxM.05.0890	CPSF A subunit region, putative
LmxM.05.0960	metallo-peptidase, Clan M-, Family M49
LmxM.05.0970	mercaptopyruvate sulfurtransferase
LmxM.05.1040	stomatin-like protein
LmxM.05.1080	Phosphatidylinositol 4-phosphate 5-kinase, putative
LmxM.05.1100	methyltransferase domain containing protein, putative
LmxM.05.1140	V-type proton ATPase subunit D, putative
LmxM.05.1210	protein phosphatase type 1 regulator-like protein
LmxM.06.0030	WD domain, G-beta repeat, putative
LmxM.06.0120	cyclophilin
LmxM.06.0140	proteasome beta 6 subunit, putative
LmxM.06.0220	hypothetical protein, conserved
LmxM.06.0260	5'-3' exoribonuclease A, putative
LmxM.06.0350	NAD(P)-dependent steroid dehydrogenase protein, putative
LmxM.06.0370	glutamine synthetase, putative
LmxM.06.0410	60S ribosomal protein L19, putative
LmxM.06.0570	60S ribosomal protein L23a, putative
LmxM.06.0690	hypothetical protein, conserved
LmxM.06.0730	threonine ammonia-lyase
LmxM.06.0860	dihydrofolate reductase-thymidylate synthase
LmxM.06.0880	acyl-coenzyme a dehydrogenase, putative
LmxM.06.0950	glucosamine-fructose-6-phosphate aminotransferase, putative
LmxM.06.1050	protein disulfide isomerase
LmxM.06.1100	peripherally associated ATOM36
LmxM.07.0060	cytochrome c1, heme protein, mitochondrial, putative
LmxM.07.0190	protein phosphatase 2A, regulatory subunit B, putative
LmxM.07.0220	hypothetical protein, conserved
LmxM.07.0270	acetylornithine deacetylase-like protein
LmxM.07.0310	paraflagellar rod protein, putative
LmxM.07.0340	ATP-dependent RNA helicase DBP2B, putative
LmxM.07.0500	60S ribosomal protein L7a, putative
LmxM.07.0570	Nucleolar protein 54, putative
LmxM.07.0600	electron transfer flavoprotein-ubiquinone oxidoreductase, putative
LmxM.07.0640	eukaryotic translation initiation factor 3 subunit h
LmxM.07.0680	40S ribosomal protein S9, putative
LmxM.07.0700	topoisomerase-related function protein-like protein
LmxM.07.0800	flavoprotein subunit-like protein
LmxM.07.0870	splicing factor ptsr1-like protein
LmxM.07.0900	serine/threonine kinase, putative
LmxM.07.0930	hypothetical protein, conserved
LmxM.07.0990	nucleolar RNA-binding protein, putative
LmxM.07.1000	RNA binding protein-like protein
LmxM.07.1030	peptidyl-prolyl cis-trans isomerase/rotamase, putative
LmxM.07.1050	cation-transporting ATPase, putative
LmxM.08.0030	Qc-SNARE protein, putative

LmxM.08.0200	Inositol phosphosphingolipids phospholipase C
LmxM.08.0290	iron superoxide dismutase A, mitochondrial
LmxM.08.0320	mitochondrial associated ribonuclease, putative
LmxM.08.0410	WD domain, G-beta repeat, putative
LmxM.08.0420	hypothetical protein,conserved
LmxM.08.0550	translation initiation factor-like protein
LmxM.08.0890	mitochondrial DNA polymerase beta
LmxM.08.0960	Dpy-30 motif containing protein, putative
LmxM.08.1040b	hypothetical protein
LmxM.08.1040c	hypothetical protein
LmxM.08.1070	cathepsin L-like protease, putative
LmxM.08.1070pa	hypothetical protein
LmxM.08.1100	hypothetical protein, conserved
LmxM.08.1110	stress-induced protein sti1
LmxM.08.1120	Repeat of unknown function (DUF1126), putative
LmxM.08.1222	hypothetical protein, conserved
LmxM.08_29.002	transcription factor-like protein
LmxM.08_29.003	ribosomal protein L3-like protein
LmxM.08_29.006	tryptophanyl-tRNA synthetase, putative
LmxM.08_29.011	C2 domain in Dock180 and Zizimin proteins, putative
LmxM.08_29.012	proteasome regulatory non-ATPase subunit, putative
LmxM.08_29.015	Putative intraflagellar transport protein B2
LmxM.08_29.027	hypothetical protein
LmxM.08_29.028	hypothetical protein
LmxM.08_29.032	TPR repeat, putative
LmxM.08_29.035	hypothetical protein, conserved
LmxM.08_29.051	cofilin-like protein
LmxM.08_29.062	ABC transporter, putative
LmxM.08_29.066	flagellar radial spoke protein-like, putative
LmxM.08_29.068	Triple RNA binding domain protein 3
LmxM.08_29.076	heat shock protein 90, putative
LmxM.08_29.078	U-box domain protein, putative
LmxM.08_29.085	High mobility group protein TDP1
LmxM.08_29.086	guanine deaminase, putative
LmxM.08_29.087	hypothetical protein, conserved
LmxM.08_29.088	ADP-ribosylation factor-like protein 3A, putative
LmxM.08_29.092	LETM1 and EF-hand domain-containing protein 1, putative
LmxM.08_29.094	hypothetical protein, conserved
LmxM.08_29.100	hypothetical protein, conserved
LmxM.08_29.107	ribosomal protein L1a, putative
LmxM.08_29.110	Ankyrin repeats (3 copies), putative
LmxM.08_29.115	tryparedoxin 2, putative
LmxM.08_29.116	tryparedoxin 1, putative
LmxM.08_29.117	hypothetical protein, conserved
LmxM.08_29.124	Hsp70 protein/TPR repeat, putative
LmxM.08_29.127	serine peptidase, putative
LmxM.08_29.131	carnitine/choline acetyltransferase, putative
LmxM.08_29.138	hypothetical protein, conserved
LmxM.08_29.147	hypothetical protein, conserved
LmxM.08_29.150	Nodulin-like, putative
LmxM.08_29.160	endomembrane protein 70, putative
LmxM.08_29.172	histone H2A, putative
LmxM.08_29.175	paraflagellar rod protein 1D, putative
LmxM.08_29.180	40S ribosomal protein S15A, putative
LmxM.08_29.183	dihydrolipoamide dehydrogenase, putative
LmxM.08_29.196	fumarate hydratase, putative

LmxM.08_29.209	AAA domain containing protein, putative
LmxM.08_29.210	Present in the outer mitochondrial membrane proteome 37
LmxM.08_29.211	UDP-glucose:glycoprotein glucosyltransferase, putative
LmxM.08_29.216	rab-GDP dissociation inhibitor, putative
LmxM.08_29.220	dynamin-1-like protein
LmxM.08_29.224	Aminopeptidase M1, putative
LmxM.08_29.231	enoyl-CoA hydratase/isomerase-like protein
LmxM.08_29.234	nucleosome assembly protein (NAP), putative
LmxM.08_29.244	3'5'-cyclic nucleotide phosphodiesterase, putative
LmxM.08_29.246	60S ribosomal protein L13, putative
LmxM.08_29.250	vacuolar protein sorting-associated protein 4
LmxM.08_29.251	ATP-dependent 6-phosphofructokinase, glycosomal
LmxM.08_29.257	serine/threonine-protein kinase, putative
LmxM.08_29.263	Present in the outer mitochondrial membrane proteome 24
LmxM.08_29.280	inosine-adenosine-guanosine-nucleosidehydrolase, putative
LmxM.08_29.283	RNA recognition motif. (a.k.a. RRM, RBD, or RNP domain), putative
LmxM.08_29.286	40S ribosomal protein S19-like protein
LmxM.09.0010	hypothetical protein, conserved
LmxM.09.0100	flagellar pocket cytoskeletal protein bilbo1
LmxM.09.0220	hypothetical protein, conserved
LmxM.09.0230	ATP-dependent protease ATPase subunit HslU2
LmxM.09.0520	Flagellum attachment zone protein 3
LmxM.09.0740	ubiquitin ligase, putative
LmxM.09.0770	oligopeptidase b
LmxM.09.0891	polyubiquitin, putative
LmxM.09.0910	calmodulin, putative
LmxM.09.0940	hypothetical protein, conserved
LmxM.09.0970	elongation factor-1 gamma
LmxM.09.1010	Present in the outer mitochondrial membrane proteome 10
LmxM.09.1050	MSP (Major sperm protein) domain containing protein, putative
LmxM.09.1070	eukaryotic translation initiation factor 2 subunit, putative
LmxM.09.1120	mitochondrial RNA binding protein 2
LmxM.09.1320	paraflagellar rod component, putative
LmxM.09.1490	cytochrome b5-like protein, putative
LmxM.09.1510	hypothetical protein, conserved
LmxM.10.0070	ribosomal protein l35a, putative
LmxM.10.0130	hypothetical protein, conserved
LmxM.10.0180	leucine-rich repeat protein, putative
LmxM.10.0200	Mitogen-activated protein kinase 10, putative
LmxM.10.0210	nucleolar protein 56, putative
LmxM.10.0219	hypothetical protein, unknown function
LmxM.10.0290	isocitrate dehydrogenase [NADP]
LmxM.10.0390	GP63, leishmanolysin
LmxM.10.0420	dihydroxyacetone kinase 1-like protein
LmxM.10.0470	GP63, leishmanolysin
LmxM.10.0490	mitogen-activated protein kinase
LmxM.10.0510	glycerol-3-phosphate dehydrogenase [NAD+], glycosomal/mitochondrial
LmxM.10.0780	WD domain, G-beta repeat, putative
LmxM.10.0850	nuclear transport factor 2, putative
LmxM.10.0870	histone H3
LmxM.10.0890	FKBP-type peptidyl-prolyl cis-trans isomerase, putative
LmxM.10.0910	small GTP-binding protein Rab11, putative
LmxM.10.1030	RNA-binding protein-like protein
LmxM.10.1080	Eukaryotic translation initiation factor 4 gamma 5
LmxM.10.1110	PAB1-binding protein , putative
LmxM.10.1150	Cilia- and flagella-associated protein 45, putative

LmxM.10.1160	Ras-related protein RabX1, putative
LmxM.10.1190	flagellar protofilament ribbon protein-like protein
LmxM.10.1240	Beta-1,2 mannosyltransferase
LmxM.11.0070	GRIP domain containing protein, putative
LmxM.11.0100	seryl-tRNA synthetase
LmxM.11.0180	hypothetical protein, conserved
LmxM.11.0210	acidocalcisomal pyrophosphatase
LmxM.11.0240	proteasome alpha 7 subunit, putative
LmxM.11.0350	14-3-3 protein 2, putative
LmxM.11.0400	tubulin-tyrsoine ligase-like protein
LmxM.11.0470	pumilio-repeat, RNA-binding protein, putative
LmxM.11.0480	mRNA cap guanine-N7 methyltransferase, putative
LmxM.11.0490	tetratricopeptide repeat (TPR) protein
LmxM.11.0560	transcription modulator/accessory protein, putative
LmxM.11.0590	3-methylcrotonoyl-CoA carboxylase beta subunit, putative
LmxM.11.0620	metallo-peptidase, Clan MF, Family M17
LmxM.11.0630	metallo-peptidase, Clan MF, Family M17
LmxM.11.0810	hypothetical protein, conserved
LmxM.11.0820	hypothetical protein, conserved
LmxM.11.0830	hypothetical protein, conserved
LmxM.11.0870	kinesin, putative
LmxM.11.0880	START domain containing protein, putative
LmxM.11.0890	hypothetical protein, conserved
LmxM.11.0960	40S ribosomal protein S5
LmxM.11.1000	pyruvate phosphate dikinase, putative
LmxM.11.1030	hypothetical protein, conserved
LmxM.11.1100	Lanosterol 14-alpha demethylase
LmxM.11.1130	60S ribosomal protein L28, putative
LmxM.11.1160	protein transport protein SEC31, putative
LmxM.11.1170	eukaryotic release factor 3, putative
LmxM.11.1360	ribosomal protein L4/L1 family, putative
LmxM.12.0030	proteasome beta-1 subunit, putative
LmxM.12.0040	Mitochondrial SSU ribosomal protein 29, putative
LmxM.12.0210	proteasome regulatory ATPase subunittcc1l8.3, putative
LmxM.12.0250	cysteinyl-tRNA synthetase, putative
LmxM.12.0350	CCR4-NOT transcription complex subunit 2, putative
LmxM.12.0390	hypothetical protein, conserved
LmxM.12.0410	Eukaryotic protein of unknown function (DUF866), putative
LmxM.12.0450	hypothetical protein, unknown function
LmxM.12.0530	glucose-6-phosphate isomerase
LmxM.12.0630	alanine aminotransferase, putative
LmxM.12.0660	ser/thr protein phosphatase, putative
LmxM.12.0670	cytochrome oxidase subunit IV, putative
LmxM.12.1120	Flagellum attachment zone protein 2
LmxM.12.1130	NADH:flavin oxidoreductase/NADH oxidase, putative
LmxM.12.1270	arginine N-methyltransferase-like protein
LmxM.13.0090	metallo-peptidase, Clan MA(E) Family M32
LmxM.13.0160	protein kinase A regulatory subunit
LmxM.13.0280	alpha tubulin
LmxM.13.0300	alpha tubulin, putative, (fragment)
LmxM.13.0430	Flagellar radial spoke protein 4/6
LmxM.13.0450	ALBA-domain protein 1
LmxM.13.0460	hypothetical protein, conserved
LmxM.13.0500	ATP-dependent RNA helicase, putative
LmxM.13.0560	60S ribosomal protein L18, putative
LmxM.13.0570	40S ribosomal protein S12, putative

LmxM.13.0670	mannose-specific lectin, putative
LmxM.13.0680	Metallopeptidase family M24, putative
LmxM.13.0780	protein kinase, putative
LmxM.13.0870	mitochondrial processing peptidase alpha subunit, putative
LmxM.13.0880	hypothetical protein, conserved
LmxM.13.1050	Dpy-30 motif containing protein, putative
LmxM.13.1060	NADH-cytochrome b5 reductase, putative
LmxM.13.1090	proteasome regulatory ATPase subunit 2, putative
LmxM.13.1100	leucyl-tRNA synthetase, putative
LmxM.13.1190	adenylosuccinate synthetase, putative
LmxM.13.1220	40S ribosomal protein S4, putative
LmxM.13.1350	AAA domain/Dpy-30 motif containing protein, putative
LmxM.13.1360	hypothetical protein, conserved
LmxM.13.1480	Ran-binding protein 1, putative
LmxM.13.1580	ubiquitin-conjugating enzyme, putative
LmxM.13.1610	Kinesin-13 5, putative
LmxM.13.1650	dynein heavy chain, putative
LmxM.13.1660	chaperonin TCP20, putative
LmxM.14.0100	hypothetical protein, conserved
LmxM.14.0130	nucleoside hydrolase-like protein
LmxM.14.0190	Thioredoxin-like, putative
LmxM.14.0260	von Willebrand factor type A domain containing protein, putative
LmxM.14.0310	proteasome alpha 3 subunit, putative
LmxM.14.0450	N-terminal conserved domain of Nudc./CS domain containing protein, putative
LmxM.14.0660	fatty acid elongase, putative
LmxM.14.0705	fatty acid elongase, putative
LmxM.14.0750	hypothetical protein, conserved
LmxM.14.0800	hypothetical protein, conserved
LmxM.14.0850	small myristoylated protein-3, putative
LmxM.14.0930	tc40 antigen-like
LmxM.14.0990	ADP/ATP mitochondrial carrier-like protein
LmxM.14.1100	kinesin K39, putative
LmxM.14.1140	hypothetical protein, conserved
LmxM.14.1160	enolase
LmxM.14.1210	NLI interacting factor-like phosphatase, putative
LmxM.14.1220	hypothetical protein, conserved
LmxM.14.1270	ubiquitin/ribosomal protein S27a, putative
LmxM.14.1320	serine hydroxymethyltransferase, putative
LmxM.14.1360	myo-inositol-1-phosphate synthase
LmxM.14.1370	tyrosyl-tRNA synthetase, putative
LmxM.14.1440	cytoskeleton associated protein, putative
LmxM.15.0040	Possible lysine decarboxylase, putative
LmxM.15.0050	cytochrome-b5 reductase, putative
LmxM.15.0090	ATP-dependent protease ATPase subunit HslU1, putative
LmxM.15.0130	ATP-dependent RNA helicase, putative
LmxM.15.0170	protein phosphatase 2C, putative
LmxM.15.0200	60S ribosomal protein L13a, putative
LmxM.15.0230	lysyl-tRNA synthetase, putative
LmxM.15.0240	sterol carrier protein, putative
LmxM.15.0270	replication Factor A 28 kDa subunit, putative
LmxM.15.0275	ribonucleoprotein p18, mitochondrial precursor, putative
LmxM.15.0440b	hypothetical protein
LmxM.15.0540	Distal docking complex protein 1
LmxM.15.0770	protein kinase, putative
LmxM.15.0950	40S ribosomal protein S3, putative
LmxM.15.0990	succinate dehydrogenase, putative

LmxM.15.1000	60S ribosomal protein L6, putative
LmxM.15.1010	glutamate dehydrogenase
LmxM.15.1040	tryparedoxin peroxidase
LmxM.15.1160	tryparedoxin peroxidase
LmxM.15.1203	60S acidic ribosomal protein P2
LmxM.15.1320	Eukaryotic translation initiation factor 4 gamma 2
LmxM.15.1330	actin-like protein, putative
LmxM.15.1380	nucleolar RNA binding protein, putative
LmxM.15.1440	glutaminyl-tRNA synthetase, putative
LmxM.15.1450	proliferative cell nuclear antigen (PCNA), putative
LmxM.15.1470	ribosomal protein S6, putative
LmxM.15.1481	cAMP specific phosphodiesterase, putative
LmxM.16.0110	Phosphotransferase enzyme family, putative
LmxM.16.0140	eukaryotic translation initiation factor 1A, putative
LmxM.16.0230	protein tyrosine phosphatase-like protein
LmxM.16.0260	Ankyrin repeats (many copies)/Alpha/beta hydrolase family, putative
LmxM.16.0460	60S ribosomal protein L21, putative
LmxM.16.0520	hypothetical protein, conserved
LmxM.16.0530	dihydroorotate dehydrogenase, putative
LmxM.16.0540	aspartate carbamoyltransferase, putative
LmxM.16.0550	orotidine-5-phosphate decarboxylase/orotate phosphoribosyltransferase, putative
LmxM.16.0570	histone H3
LmxM.16.0590	carbamoyl-phosphate synthase, putative
LmxM.16.0690	eukaryotic translation initiation factor eIF2A, putative
LmxM.16.0760	transaldolase, putative
LmxM.16.0890	Nucleolar protein,Nop52, putative
LmxM.16.0900	hypothetical protein, conserved
LmxM.16.0950	sucrose-phosphate synthase-like protein
LmxM.16.1005	Protein of unknown function (DUF3184), putative
LmxM.16.1020	Protein of unknown function (DUF3184), putative
LmxM.16.1130	tyrosyl or methionyl-tRNA synthetase-like protein
LmxM.16.1180	delta-coat protein, putative
LmxM.16.1310	cytochrome c, putative
LmxM.16.1420	hypothetical protein, conserved
LmxM.16.1530	endoplasmic reticulum oxidoreductin, putative
LmxM.16.1600	eukaryotic translation initiation factor 4 gamma 3
LmxM.16.1610	prohibitin, putative
LmxM.16.1660	Flagellar Member 3
LmxM.17.0010	eukaryotic translation initiation factor 3 subunit a
LmxM.17.0080	elongation factor 1-alpha
LmxM.17.0085	elongation factor 1-alpha
LmxM.17.0120	hypothetical protein, conserved
LmxM.17.0140	peptidase t, putative
LmxM.17.0250	cystathionine beta-synthase
LmxM.17.0410	hypothetical protein, conserved
LmxM.17.0470	hypothetical protein, conserved
LmxM.17.0480	Phenazine biosynthesis-like protein, putative
LmxM.17.0550	RNA-binding protein, putative
LmxM.17.0600	P-type ATPase, putative
LmxM.17.0725	GMP reductase
LmxM.17.0735	ysine decarboxylase-like protein
LmxM.17.0800	OSM3-like kinesin, putative
LmxM.17.0870	hypothetical protein, conserved
LmxM.17.0910	acyltransferase, putative
LmxM.17.0980	Mak10 subunit, NatC N(alpha)-terminal acetyltransferase, putative
LmxM.17.1050	hydrolase-like protein

LmxM.17.1220	histone H2B
LmxM.17.1290	eukaryotic translation initiation factor 3 subunit b
LmxM.17.1400	otubain cysteine peptidase, Clan CA, family C65, putative
LmxM.18.0010	kinetoplast polyadenylation/uridylation factor 1
LmxM.18.0020	diphosphomevalonate decarboxylase, putative
LmxM.18.0150	serine/threonine protein phosphatase type 5, putative
LmxM.18.0210	Activator of Hsp90 ATPase, N-terminal/Activator of Hsp90 ATPase homolog 1-like protein, putative
LmxM.18.0220	RNA-binding protein 29, putative
LmxM.18.0270	protein kinase, putative
LmxM.18.0280	hypothetical protein, conserved
LmxM.18.0300	hypothetical protein, conserved
LmxM.18.0510	aconitase, putative
LmxM.18.0530	tubulin cofactor C domain-containing protein RP2, putative
LmxM.18.0560	vacuolar ATP synthase subunit c, putative
LmxM.18.0590	RNA binding protein, putative
LmxM.18.0620	60S ribosomal protein L10a, putative
LmxM.18.0670	citrate synthase, putative
LmxM.18.0680	citrate synthase, putative
LmxM.18.0700	HEAT repeats, putative
LmxM.18.0740	Elongation factor Tu, mitochondrial, putative
LmxM.18.0820	hypothetical protein, conserved
LmxM.18.0990	UTP-glucose-1-phosphate uridylyltransferase 2, putative
LmxM.18.1080	protein kinase A catalytic subunit 3
LmxM.18.1090	phosphodiesterase, putative
LmxM.18.1130	Ras-related protein Rab5A, putative
LmxM.18.1210	prolyl-tRNA synthetase, putative
LmxM.18.1290	mitochondrial carrier protein, putative
LmxM.18.1300	mitochondrial carrier protein, putative
LmxM.18.1370	heat shock protein, putative
LmxM.18.1380	pyruvate dehydrogenase E1 component alpha subunit, putative
LmxM.18.1400	60S ribosomal protein L34, putative
LmxM.18.1420	pumilio protein, putative
LmxM.18.1520	P-type H+-ATPase, putative
LmxM.18.1640	hypothetical protein, conserved
LmxM.18.1660	gamma-glutamylcysteine synthetase, putative
LmxM.19.0020	hypothetical protein, conserved
LmxM.19.0030	histone H2B
LmxM.19.0060	40S ribosomal protein S2
LmxM.19.0090	hypothetical protein, conserved
LmxM.19.0100	fibrillarin, putative
LmxM.19.0140	Associated kinase of Tb14-3-3, putative
LmxM.19.0150	mitogen-activated protein kinase kinase kinase, putative
LmxM.19.0160	metallo-peptidase, Clan MG, Family M24
LmxM.19.0190	multiprotein-bridging factor 1, putative
LmxM.19.0240	hypothetical protein, conserved
LmxM.19.0260	C-terminal motor kinesin, putative
LmxM.19.0295	RNA binding protein
LmxM.19.0300	RNA binding protein, putative
LmxM.19.0320	intraflagellar transport protein 52
LmxM.19.0340	hypothetical protein, conserved
LmxM.19.0390	40S ribosomal protein S13, putative
LmxM.19.0440	nucleosome assembly protein, putative
LmxM.19.0490	hypothetical protein, conserved
LmxM.19.0520	paraflagellar rod component, putative
LmxM.19.0550	metallo- peptidase, Clan MG, Family M24
LmxM.19.0630	histone H3 variant V

LmxM.19.0650	hypothetical protein, conserved
LmxM.19.0710	glycosomal malate dehydrogenase
LmxM.19.0760	transporter, putative
LmxM.19.0790	signal recognition particle protein, putative
LmxM.19.0800	ABC transporter, putative
LmxM.19.1005	4-coumarate:coa ligase-like protein
LmxM.19.1040	phenylalanyl-tRNA synthetase, putative
LmxM.19.1120	proteasome regulatory non-ATP-ase subunit 9, putative
LmxM.19.1160	hypothetical protein, conserved
LmxM.19.1230	hypothetical protein, conserved
LmxM.19.1280	SPFH domain / Band 7 family, putative
LmxM.19.1290	SPFH domain / Band 7 family, putative
LmxM.19.1400	hypothetical protein, conserved
LmxM.19.1420	cysteine peptidase A (CPA)
LmxM.19.1530	peptidylprolyl isomerase-like protein
LmxM.19.1560	inosine-5'-monophosphate dehydrogenase
LmxM.19.1590	ATP-dependent zinc metallopeptidase
LmxM.20.0080	hypothetical protein, conserved
LmxM.20.0240	transmembrane protein, putative
LmxM.20.0560	cytidine triphosphate synthase, putative
LmxM.20.0660	serine/threonine protein phosphatase 2A regulatory subunit, putative
LmxM.20.0810	vesicular-fusion protein nsf, putative
LmxM.20.0950	conserved protein, unknown function
LmxM.20.0970	1,2-Dihydroxy-3-keto-5-methylthiopentene dioxygenase, putative
LmxM.20.1180	calpain-like cysteine peptidase
LmxM.20.1185	calpain-like cysteine peptidase, putative
LmxM.20.1280	calpain-like cysteine peptidase, putative
LmxM.20.1290	nucleosome assembly protein (NAP), putative
LmxM.20.1310	small myristoylated protein 1
LmxM.20.1350	coatomer beta subunit, putative
LmxM.20.1400	paralyzed flagella protein 16
LmxM.20.1550	amidohydrolase, putative
LmxM.20.1560	amidohydrolase, putative
LmxM.20.1570	amidohydrolase, putative
LmxM.20.1640	Suppressor of G2 allele of SKP1, putative
LmxM.20.1650	ribosomal protein S11 homolog
LmxM.20.1690	soluble n-ethylmaleimide sensitive factor, putative
LmxM.21.0100	hypothetical protein, conserved
LmxM.21.0125	DNA topoisomerase 1A
LmxM.21.0210	hypothetical protein, conserved
LmxM.21.0340	mitochondrial processing peptidase alpha subunit, putative
LmxM.21.0380	Protein of unknown function (DUF667), putative
LmxM.21.0430	hypothetical protein, conserved
LmxM.21.0490	DnaJ protein, putative
LmxM.21.0530	hypothetical protein, conserved
LmxM.21.0540	Lupus La protein homolog, putative
LmxM.21.0550	dihydrolipoamide acetyltransferase precursorlike protein
LmxM.21.0610	RNA helicase, putative
LmxM.21.0710	ribonuclease L inhibitor, putative
LmxM.21.0730	60S ribosomal protein L36, putative
LmxM.21.0760	proteasome regulatory non-ATP-ase subunit 5, putative
LmxM.21.0800	CCR4-NOT transcription complex subunit 1
LmxM.21.0810	methionyl-tRNA synthetase, putative
LmxM.21.0820	Temperature dependent protein affecting M2 dsRNA replication, putative
LmxM.21.0825	plectin, putative
LmxM.21.0840	metallo-peptidase, Clan MG, Family M24

LmxM.21.0845	hypoxanthine-guanine phosphoribosyltransferase
LmxM.21.0850	xanthine phosphoribosyltransferase
LmxM.21.0980	intraflagellar transport protein 172, putative
LmxM.21.1050	60S ribosomal protein L9, putative
LmxM.21.1060	40S ribosomal protein S23, putative
LmxM.21.1080	cell division protein kinase 2
LmxM.21.1090	T-complex protein 1, delta subunit, putative
LmxM.21.1250	adenylate kinase, putative
LmxM.21.1340	ATP synthase, putative
LmxM.21.1430	2-oxoisovalerate dehydrogenase alpha subunit, putative
LmxM.21.1552	ATP-dependent RNA helicase SUB2, putative
LmxM.21.1555	hypothetical protein, conserved
LmxM.21.1566	Microtubule-binding protein MIP-T3, putative
LmxM.21.1700	proteasome alpha 2 subunit, putative
LmxM.21.1710	cytochrome c oxidase subunit VI, putative
LmxM.21.1720	60S ribosomal protein L32
LmxM.21.1760	centromere/microtubule binding protein cbf5, putative
LmxM.21.1770	ATP synthase F1 subunit gamma protein, putative
LmxM.21.1780	40S ribosomal protein S6, putative
LmxM.21.1820	60S ribosomal protein L37a, putative
LmxM.21.1830	proteasome subunit alpha type-5, putative
LmxM.22.0060	transcription factor-like protein
LmxM.22.0110	GMP synthase [glutamine-hydrolysing]
LmxM.22.0180	hypothetical protein, conserved
LmxM.22.0210	Nucleolar protein 168, putative
LmxM.22.0260	translocon-associated protein (TRAP), alpha subunit, putative
LmxM.22.0300	hypothetical protein, conserved
LmxM.22.0420	40S ribosomal protein S15, putative
LmxM.22.0470	Putative tRNA binding domain containing protein, putative
LmxM.22.0570	proteasome regulatory ATPase subunit 1, putative
LmxM.22.0620	proteasome regulatory ATPase subunit 5, putative
LmxM.22.0730	cytoskeleton associated protein, putative
LmxM.22.0770	NADH-cytochrome b5 reductase, putative
LmxM.22.0990	hypothetical protein, conserved
LmxM.22.1110	dynein heavy chain, cytosolic, putative
LmxM.22.1140	adaptor complex AP-1 medium subunit, putative
LmxM.22.1290	ribonucleoside-diphosphate reductase small chain, putative
LmxM.22.1330	hypothetical protein, conserved
LmxM.22.1360	farnesyl pyrophosphate synthase, putative
LmxM.22.1370	intraflagellar transport protein-like protein
LmxM.22.1460	i/6 autoantigen-like protein
LmxM.22.1500	ATP-dependent DEAD/H RNA helicase, putative
LmxM.22.1520	40S ribosomal protein L14, putative
LmxM.22.1540	alanyl-tRNA synthetase, putative
LmxM.23.0040	tryparedoxin peroxidase
LmxM.23.0050	peptidyl-prolyl cis-trans isomerase, putative
LmxM.23.0080	hypothetical protein, conserved
LmxM.23.0110	mannose-1-phosphate guanyltransferase
LmxM.23.0200	endoribonuclease L-PSP (pb5), putative
LmxM.23.0220	p-glycoprotein, putative
LmxM.23.0260	argininosuccinate synthase, putative
LmxM.23.0360	NADP-dependent alcohol dehydrogenase, putative
LmxM.23.0370	cytochrome c oxidase subunit 10, putative
LmxM.23.0547	hypothetical protein, conserved
LmxM.23.0550	ubiquitin-activating enzyme E1, putative
LmxM.23.0560	kinesin-C

LmxM.23.0640	hypothetical protein, conserved
LmxM.23.0680	mitochondrial DNA primase, putative
LmxM.23.0690	3-ketoacyl-CoA thiolase-like protein
LmxM.23.0710	acetyl-CoA synthetase, putative
LmxM.23.0760	mitochondrial RNA binding protein, putative
LmxM.23.0860	quinone oxidoreductase-like protein
LmxM.23.0950	metallo-peptidase, Clan MF, Family M17
LmxM.23.1000	hypothetical protein, unknown function
LmxM.23.1020	hypothetical protein, unknown function
LmxM.23.1165	coronin, putative
LmxM.23.1220	T-complex protein 1, gamma subunit, putative
LmxM.23.1310	dynein heavy chain, putative
LmxM.23.1410	succinate dehydrogenase and fumarate reductase iron-sulfur protein, putative
LmxM.23.1470	hypothetical protein, conserved
LmxM.23.1480	alanine racemase, putative
LmxM.23.1510	V-type proton ATPase subunit a, putative
LmxM.23.1550	hypothetical protein, conserved
LmxM.23.1580	Nucleoside 2-deoxyribosyltransferase, putative
LmxM.23.1640	Galactose oxidase, central domain containing protein, putative
LmxM.23.1750	hypothetical protein, conserved
LmxM.24.0040	60S ribosomal protein L17, putative
LmxM.24.0100	Calmodulin-binding, putative
LmxM.24.0140	ankyrin/TPR repeat protein
LmxM.24.0280	Dynein intermediate chain 1, axonemal
LmxM.24.0320	fumarate hydratase, putative
LmxM.24.0370	aspartate aminotransferase, putative
LmxM.24.0600	Ribosome 60S biogenesis N-terminal, putative
LmxM.24.0640	Kinesin-13 3, putative
LmxM.24.0761	malic enzyme
LmxM.24.0770	malic enzyme, putative
LmxM.24.0840	dynein light chain p28, axonemal, putative
LmxM.24.0850	triosephosphate isomerase
LmxM.24.1500	IgE-dependent histamine-releasing factor, putative
LmxM.24.1560	hypothetical protein, conserved
LmxM.24.1570	RNA recognition motif. (a.k.a. RRM, RBD, or RNP domain)/RNA recognition motif (a.k.a. RRM, RBD, o
LmxM.24.1630	Succinate dehydrogenase [ubiquinone] flavoprotein subunit, mitochondrial
LmxM.24.1640	Male sterility protein, putative
LmxM.24.1840	lysophospholipase, putative
LmxM.24.1980	hypothetical protein, conserved
LmxM.24.2030	3-oxoacyl-(acyl-carrier protein) reductase, putative
LmxM.24.2060	transketolase, putative
LmxM.24.2070	40S ribosomal protein S8, putative
LmxM.24.2110	3-hydroxy-3-methylglutaryl-CoA synthase, putative
LmxM.24.2160	Glycerophosphoryl diester phosphodiesterase family, putative
LmxM.24.2180	Trypanosome basal body component protein
LmxM.24.2210	60S ribosomal protein L12, putative
LmxM.24.2320	mitogen-activated protein kinase
LmxM.25.0080	poly(A)-binding protein, putative
LmxM.25.0120	electron transfer flavoprotein, putative
LmxM.25.0130	elongation factor, putative
LmxM.25.0190	ubiquitin carboxyl-terminal hydrolase, putative
LmxM.25.0530	ABC transporter, mitochondrial, putative
LmxM.25.0540	hypothetical protein, conserved
LmxM.25.0620	RNA polymerase I second largest subunit, putative
LmxM.25.0670	epsin, putative
LmxM.25.0715	hypothetical protein, conserved

LmxM.25.0720	eukaryotic initiation factor 5a, putative
LmxM.25.0750	protein phosphatase, putative
LmxM.25.0910	cyclophilin a, putative
LmxM.25.0980	dynein heavy chain, putative
LmxM.25.1000	hypothetical protein, conserved
LmxM.25.1060	hypothetical protein, conserved
LmxM.25.1080	hypothetical protein, conserved
LmxM.25.1090	magnesium transporter protein 2
LmxM.25.1120	aldehyde dehydrogenase, mitochondrial precursor
LmxM.25.1130	cytochrome oxidase subunit VII
LmxM.25.1170	ATP synthase subunit beta, mitochondrial, putative
LmxM.25.1180	ATP synthase subunit beta, mitochondrial, putative
LmxM.25.1210	hypothetical protein, conserved
LmxM.25.1340	protein transport protein Sec24C, putative
LmxM.25.1420	GTP-binding protein, putative
LmxM.25.1460	cAMP response protein, putative
LmxM.25.1540	hypothetical protein, conserved
LmxM.25.1600	Flagella-associated protein 251, putative
LmxM.25.1610	Eukaryotic translation initiation factor 3 subunit F
LmxM.25.1620	hypothetical protein, conserved
LmxM.25.1640	hypothetical protein, conserved
LmxM.25.1710	pyruvate dehydrogenase E1 beta subunit, putative
LmxM.25.1770	3-oxo-5-alpha-steroid 4-dehydrogenase, putative
LmxM.25.1820	hypothetical protein, conserved
LmxM.25.1840	General negative regulator of transcription subunit 5
LmxM.25.2010	2,4-dihydroxyhept-2-ene-1,7-dioic acid aldolase, putative
LmxM.25.2020	hypothetical protein, conserved
LmxM.25.2090	hypothetical protein, conserved
LmxM.25.2130	succinyl-CoA synthetase alpha subunit, putative
LmxM.25.2340	AGC essential kinase 1
LmxM.25.2360	Pumilio homology domain family member 8
LmxM.25.2410	hypothetical protein, conserved
LmxM.25.2450	histone H4
LmxM.26.0010	brix domain containing-like protein
LmxM.26.0030	glycine dehydrogenase, putative
LmxM.26.0140	adenine phosphoribosyltransferase
LmxM.26.0170	60S ribosomal protein L7, putative
LmxM.26.0320	C-1-tetrahydrofolate synthase, cytoplasmic, putative
LmxM.26.0430	nucleotide binding protein-like protein
LmxM.26.0620	10 kDa heat shock protein, putative
LmxM.26.0650	Flagella-associated protein 61, putative
LmxM.26.0660	protein disulfide isomerase, putative
LmxM.26.0680	Protein of unknown function (DUF2012), putative
LmxM.26.0830	aspartate--ammonia ligase, putative
LmxM.26.0880	40S ribosomal protein S16, putative
LmxM.26.1020	Inner arm dynein 5-1
LmxM.26.1040	hypothetical protein, conserved
LmxM.26.1240	heat shock protein 70-related protein
LmxM.26.1380	prefoldin-like protein
LmxM.26.1550	trifunctional enzyme alpha subunit, mitochondrial precursor-like protein
LmxM.26.1570	thimet oligopeptidase, putative
LmxM.26.1610	Proline dehydrogenase, mitochondrial
LmxM.26.1630	40S ribosomal protein S33, putative
LmxM.26.1710	cytochrome c oxidase subunit V, putative
LmxM.26.1790	Nucleolar protein 287, putative
LmxM.26.1900	hypothetical protein, conserved

LmxM.26.1940	Thioesterase-like superfamily, putative
LmxM.26.1960	hypothetical protein, conserved
LmxM.26.2000	methyltransferase domain containing protein, putative
LmxM.26.2250	hypothetical protein, conserved
LmxM.26.2640	hypothetical protein, conserved
LmxM.26.2660	Nucleoporin NUP92
LmxM.26.2710	glutamate 5-kinase, putative
LmxM.27.0040	metallo- peptidase, Clan M- Family M48
LmxM.27.0070	hypothetical protein, conserved
LmxM.27.0160	SET domain containing protein, putative
LmxM.27.0190	proteasome alpha 7 subunit, putative
LmxM.27.0210	Vacuolar iron transporter 1, putative
LmxM.27.0240	kinetoplast-associated protein-like protein
LmxM.27.0300	methylmalonyl-coenzyme a mutase, putative
LmxM.27.0420	ribokinase, putative
LmxM.27.0490	cysteine peptidase, Clan CA, family C2, putative
LmxM.27.0510	cysteine peptidase, Clan CA, family C2, putative
LmxM.27.0520	radial spoke protein 3, putative
LmxM.27.0650	hypothetical protein, conserved
LmxM.27.0760	ras-related protein RAB1A, putative
LmxM.27.0810	glutaredoxin-like protein
LmxM.27.0840	hypothetical protein, conserved
LmxM.27.0870	Repeat of unknown function (DUF1126)/EF-hand domain pair, putative
LmxM.27.0880	2-oxoglutarate dehydrogenase subunit, putative
LmxM.27.0930	isovaleryl-coA dehydrogenase, putative
LmxM.27.1030	hypothetical protein, conserved
LmxM.27.1060	cysteine desulfurase, putative
LmxM.27.1100	hypothetical protein, conserved
LmxM.27.1110	guide RNA-binding protein of 21 kDa
LmxM.27.1220	hypothetical protein, conserved
LmxM.27.1250	Serine-threonine kinase receptor-associated protein, putative
LmxM.27.1260	T-complex protein 1, beta subunit, putative
LmxM.27.1300	KH domain containing protein, putative
LmxM.27.1310	arginyl-tRNA synthetase, putative
LmxM.27.1380	60S acidic ribosomal protein P0, putative
LmxM.27.1380a	hypothetical protein
LmxM.27.1450	CBF/Mak21 family, putative
LmxM.27.1460	proteasome regulatory non-ATP-ase subunit 3, putative
LmxM.27.1640	BRO1-like domain/ALIX V-shaped domain binding to HIV, putative
LmxM.27.1680	hypothetical protein, conserved
LmxM.27.1710	eukaryotic translation release factor, putative
LmxM.27.1730	Flabarin, putative
LmxM.27.1805	Phosphoenolpyruvate carboxykinase [ATP], glycosomal
LmxM.27.1807	Phosphoenolpyruvate carboxykinase [ATP], glycosomal
LmxM.27.1820	hypothetical protein, unknown function
LmxM.27.1870	trypanothione synthetase
LmxM.27.1980	FtsJ cell division protein, putative
LmxM.27.2000	GMP-PDE, delta subunit, putative
LmxM.27.2020	D-lactate dehydrogenase-like protein
LmxM.27.2030	branched-chain amino acid aminotransferase, putative
LmxM.27.2100	RNA-binding protein, putative
LmxM.27.2300	transcription elongation regulator-like protein
LmxM.27.2330	GTP binding protein, putative
LmxM.27.2360	hypothetical protein, conserved
LmxM.27.2390	TPR-repeat protein, putative
LmxM.27.2400	heat shock protein DNAJ, putative

LmxM.27.2480	hypothetical protein, conserved
LmxM.28.0040	hypothetical protein, conserved
LmxM.28.0110	proteasome beta 3 subunit, putative
LmxM.28.0140	pantothenate kinase subunit, putative
LmxM.28.0210	Histone H2B variant V
LmxM.28.0340	hypothetical protein, conserved
LmxM.28.0380	saccharopine dehydrogenase, putative
LmxM.28.0480	carbonic anhydrase-like protein
LmxM.28.0540	ribosomal protein S26, putative
LmxM.28.0560	hypothetical protein, conserved
LmxM.28.0690	serine/threonine protein phosphatase catalytic subunit, putative
LmxM.28.0730	hypothetical protein, conserved
LmxM.28.0825	RNA binding protein rbp16, putative
LmxM.28.0890	ribonucleoside-diphosphate reductase large chain, putative
LmxM.28.0930	Mitochondrial import receptor subunit ATOM46, putative
LmxM.28.0960	40S ribosomal protein S14
LmxM.28.1010	ribosomal protein S20, putative
LmxM.28.1060	hypothetical protein, conserved
LmxM.28.1110	WD domain, G-beta repeat, putative
LmxM.28.1140	electron-transfer-flavoprotein, alpha polypeptide, putative
LmxM.28.1200	luminal binding protein 1 (BiP), putative
LmxM.28.1230	SMP-30/Gluconolaconase/LRE-like region, putative
LmxM.28.1420	DNA polymerase kappa, putative
LmxM.28.1570	hydrolase, alpha/beta fold family, putative
LmxM.28.1730	proteasome regulatory non-ATP-ase subunit 2, putative
LmxM.28.1740	NOL1/NOP2/sun family, putative
LmxM.28.1780	DNA topoisomerase III, putative
LmxM.28.1820	Replication factor A protein 1
LmxM.28.1880	Nucleolar protein 136, putative
LmxM.28.1990	Leucine-rich repeat protein 1, putative
LmxM.28.2080	DEAD-box ATP-dependent RNA helicase, mitochondrial
LmxM.28.2170	Mitochondrial import receptor subunit ATOM69, putative
LmxM.28.2190	hypothetical protein, conserved
LmxM.28.2260	glycosomal membrane protein, putative
LmxM.28.2310	Eukaryotic translation initiation factor 3 subunit E
LmxM.28.2420	2-oxoglutarate dehydrogenase, E2 component, dihydrolipoamide succinyltransferase, putative
LmxM.28.2430	Vacuolar proton pump subunit B, putative
LmxM.28.2445	Putative serine esterase (DUF676), putative
LmxM.28.2510	acyl-CoA dehydrogenase, putative
LmxM.28.2555	40S ribosomal protein S17, putative
LmxM.28.2620	Coatomer subunit gamma
LmxM.28.2740	activated protein kinase c receptor (LACK)
LmxM.28.2770	heat-shock protein hsp70, putative
LmxM.28.2820	heat shock protein 70, putative
LmxM.28.2860	cytosolic malate dehydrogenase, putative
LmxM.28.2910	glutamate dehydrogenase, putative
LmxM.28.3030	Nucleoporin NUP149
LmxM.29.0120	alkyl dihydroxyacetonephosphate synthase
LmxM.29.0180	2-hydroxy-3-oxopropionate reductase, putative
LmxM.29.0350	kinesin, putative
LmxM.29.0380	phosphatase 2C, putative
LmxM.29.0430	quiescin sulfhydryl oxidase, putative
LmxM.29.0440	Ribosome recycling factor, putative
LmxM.29.0460	aspartyl-tRNA synthetase, putative
LmxM.29.0480	bystin, putative
LmxM.29.0580	AMP-binding enzyme, putative

LmxM.29.0630	histidyl-tRNA synthetase, putative
LmxM.29.0660	pdz domain containing protein, putative
LmxM.29.0730	co-chaperone GrpE, putative
LmxM.29.0760	hypothetical protein, conserved
LmxM.29.0780	mitochondrial oligo_U binding protein TBRGG1, putative
LmxM.29.0850	surface protein amastin, putative
LmxM.29.0880	adenosine kinase, putative
LmxM.29.0970	p22 protein precursor, putative
LmxM.29.1040	succinate dehydrogenase subunit 7
LmxM.29.1110	RNA-binding protein, putative
LmxM.29.1120	importin alpha, putative
LmxM.29.1250	pyridoxal kinase, putative
LmxM.29.2330	hypothetical protein, unknown function
LmxM.29.2350	sphingosine phosphate lyase-like protein, putative
LmxM.29.2370	ADP-ribosylation factor-like protein
LmxM.29.2580	reticulon domain protein, 22 kDa potentially aggravating protein (paple22)
LmxM.29.2740	TPR domain protein, conserved
LmxM.29.2820	chaperonin HSP60/CNP60, putative
LmxM.29.2850	hypothetical protein, conserved
LmxM.29.2900	aldehyde dehydrogenase, putative
LmxM.29.2980	glyceraldehyde 3-phosphate dehydrogenase, glycosomal
LmxM.29.3040	eukaryotic translation initiation factor 3 subunit d
LmxM.29.3090	RNA-binding protein 42 (RNA-binding motif protein 42), putative
LmxM.29.3130	valyl-tRNA synthetase, putative
LmxM.29.3150	Translocon-associated protein beta (TRAPB), putative
LmxM.29.3190	3-hydroxy-3-methylglutaryl-CoA reductase, putative
LmxM.29.3240	glutamyl-tRNA synthetase, putative
LmxM.29.3310	MORN repeat-containing protein 1
LmxM.29.3360	calmodulin-related protein, putative
LmxM.29.3380	PAS-domain containing phosphoglycerate kinase, putative
LmxM.29.3430	protein mkt1, putative
LmxM.29.3500	S-adenosylmethionine synthetase
LmxM.29.3580	serine/threonine-protein kinase, putative
LmxM.29.3600	ATP synthase, epsilon chain, putative
LmxM.29.3650	ribosomal protein L15, putative
LmxM.29.3680	hypothetical protein, conserved
LmxM.29.3690	Protein of unknown function (DUF3808), putative
LmxM.30.0010	5-methyltetrahydropteroyltriglutamate--homocyst ei nemethyltransferase, putative
LmxM.30.0050	cyclophilin, putative
LmxM.30.0140	ubiquitin carboxyl-terminal hydrolase, putative
LmxM.30.0190	nucleolar protein, putative
LmxM.30.0250	ATP-dependent RNA helicase, putative
LmxM.30.0390	Calpain-like protein 2
LmxM.30.0440	cytoskeleton-associated protein CAP5.5, putative
LmxM.30.0440b	hypothetical protein
LmxM.30.0560	mevalonate kinase, putative
LmxM.30.0630	hypothetical protein, conserved
LmxM.30.0640	mitochondrial RNA binding protein, putative
LmxM.30.0680	C2 domain containing protein, putative
LmxM.30.0930	sodium stibogluconate resistance protein, putative
LmxM.30.0960	hypothetical protein
LmxM.30.1010	hypothetical protein, conserved
LmxM.30.1050	hypothetical protein, unknown function
LmxM.30.1070	biotin/lipoate protein ligase-like protein
LmxM.30.1130	amidohydrolase, putative
LmxM.30.1140	monoglyceride lipase, putative

LmxM.30.1220	Pyrophosphate-energized vacuolar membrane proton pump 1, putative
LmxM.30.1570	cytochrome c oxidase VIII (COX VIII), putative
LmxM.30.1630	3-ketoacyl-CoA thiolase-like protein, putative
LmxM.30.1640	thiolase protein-like protein
LmxM.30.1680	hypothetical protein, conserved
LmxM.30.1750	nucleosome assembly protein-like protein
LmxM.30.1900	ubiquitin-fusion protein
LmxM.30.2020	succinyl-diaminopimelate desuccinylase-like protein
LmxM.30.2050	CRAL/TRIO domain containing protein, putative
LmxM.30.2080	hypothetical protein, conserved
LmxM.30.2150	prostaglandin f2-alpha synthase
LmxM.30.2250	3,2-trans-enoyl-CoA isomerase, mitochondrial precursor, putative
LmxM.30.2280	ADP-ribosylation factor, putative
LmxM.30.2340	p-nitrophenylphosphatase, putative
LmxM.30.2350	Symplekin tight junction protein C terminal, putative
LmxM.30.2600	calreticulin, putative
LmxM.30.2610	RNA polymerase ii largest subunit
LmxM.30.2630	serine/threonine protein phosphatase pp1(5.9), putative
LmxM.30.2640	2-oxoglutarate dehydrogenase, e3 component, lipoamidedehydrogenase-like protein
LmxM.30.2650	acetoin dehydrogenase e3 component-like protein
LmxM.30.2790	ADP-ribosylation factor, putative
LmxM.30.2960	Repressor of differentiation kinase 2, putative
LmxM.30.2970	acetyl-CoA carboxylase
LmxM.30.3090	peptidase, putative
LmxM.30.3110	Flagellum attachment zone protein 14
LmxM.30.3130	methylcrotonoyl-coa carboxylase biotinylated subunitprotein-like protein
LmxM.30.3160	protein kinase, putative
LmxM.31.0020	nuclear segregation protein, putative
LmxM.31.0050	protein transport protein SEC13
LmxM.31.0230	dynein light chain, flagellar outer arm, putative
LmxM.31.0270	hypothetical protein, conserved
LmxM.31.0350	hypothetical protein, conserved
LmxM.31.0360	hypothetical protein, conserved
LmxM.31.0390	19S proteasome non-atpase subunit 8
LmxM.31.0400	ATP-dependent RNA helicase HEL67
LmxM.31.0520	profilin, putative
LmxM.31.0630	AmmeMemoRadiSam system protein A/uncharacterized protein, PH0010 family, putative
LmxM.31.0680	OSM3-like kinesin, putative
LmxM.31.0720	hypothetical protein, conserved
LmxM.31.0750	nuclear RNA binding domain
LmxM.31.0830	hypothetical protein, unknown function
LmxM.31.0840	hypothetical protein, conserved
LmxM.31.0870	phenylalanyl-tRNA synthetase alpha chain, putative
LmxM.31.0950	Staphylococcal nuclease homologue/Tudor domain containing protein, putative
LmxM.31.1000	chaperonin containing t-complex protein, putative
LmxM.31.1060	Dynein intermediate chain, axonemal
LmxM.31.1160	long chain polyunsaturated fatty acid elongation enzyme-like protein
LmxM.31.1200	proteasome regulatory non-ATP-ase subunit, putative
LmxM.31.1280	RNA polymerase-like protein, putative
LmxM.31.1500	Metalloprotease M41 FtsH, putative
LmxM.31.1580	phosphomannose isomerase, putative
LmxM.31.1730	coatomer subunit epsilon
LmxM.31.1750	pumilio/PUF RNA binding protein 7, putative
LmxM.31.1760	hypothetical protein, conserved
LmxM.31.1770	phosphatidylethanolamine-binding protein, putative
LmxM.31.1820	iron superoxide dismutase, putative

LmxM.31.1830	iron superoxide dismutase, putative
LmxM.31.1850	Chaperonin HSP60, mitochondrial
LmxM.31.1910	hypothetical protein, conserved
LmxM.31.1930	hypothetical protein, conserved
LmxM.31.1940	DnaJ homolog, putative
LmxM.31.1960	1-acyl-sn-glycerol-3-phosphateacyltransferase-like protein, putative
LmxM.31.1980	Flagella-associated protein 100, putative
LmxM.31.2020	cop-coated vesicle membrane protein p24 precursor, putative
LmxM.31.2150	hypothetical protein, conserved
LmxM.31.2180	eukaryotic translation initiation factor 3 subunit k
LmxM.31.2220	hypothetical protein, unknown function
LmxM.31.2260	heat shock protein Hsp20, putative
LmxM.31.2640	cystathionine beta-lyase, putative
LmxM.31.2690	ribosomal protein L27, putative
LmxM.31.2710	ribosomal protein L27, putative
LmxM.31.2780	Nucleoporin NUP109
LmxM.31.2810	RNA-binding protein, putative
LmxM.31.2820	proteasome regulatory non-ATP-ase subunit, putative
LmxM.31.2900	Outer dynein arm docking complex protein 2, putative
LmxM.31.2950	nucleoside diphosphate kinase b
LmxM.31.2970	tubulin binding cofactor A-like protein
LmxM.31.2980	ATP binding protein-like protein
LmxM.31.3010	leucine-rich repeat protein, putative
LmxM.31.3130	ribosomal protein L3, putative
LmxM.31.3140	pyrroline-5-carboxylate synthetase-like protein
LmxM.31.3150	Flagellar Member 7
LmxM.31.3210	Miro-like protein, putative
LmxM.31.3230	dynein light intermediate chain, putative
LmxM.31.3260	glucosamine-6-phosphate isomerase, putative
LmxM.31.3270	chaperonin alpha subunit, putative
LmxM.31.3310	dihydrolipoamide dehydrogenase, putative
LmxM.31.3870	myosin XXI, putative
LmxM.31.3900	60S ribosomal protein L8, putative
LmxM.32.0240	thiol-dependent reductase 1
LmxM.32.0260	RGG-containing protein 2, putative
LmxM.32.0310	ATP-binding cassette sub-family F member 1, putative
LmxM.32.0312	heat shock protein 83-1
LmxM.32.0590	Coiled-coil domain-containing protein 40 homolog
LmxM.32.0610	paraflagellar rod component, putative
LmxM.32.0830	2,4-dienoyl-coa reductase fadh1, putative
LmxM.32.1035	hypothetical protein, conserved
LmxM.32.1070	hypothetical protein, unknown function
LmxM.32.1150	pumilio protein, putative
LmxM.32.1330	glutamine aminotransferase, putative
LmxM.32.1380	mitogen activated protein kinase, putative
LmxM.32.1590	3-ketoacyl-CoA reductase, putative
LmxM.32.1610	cytosolic nonspecific dipeptidase, putative
LmxM.32.1630	cyclophilin, putative
LmxM.32.1690	DNA polymerase delta catalytic subunit, putative
LmxM.32.1750	macrophage migration inhibitory factor-like protein
LmxM.32.1870	nucleolar GTP-binding protein, putative
LmxM.32.1920	Phosphate transport (Pho88), putative
LmxM.32.2260	glycosyl hydrolase-like protein
LmxM.32.2270	hypothetical protein, conserved
LmxM.32.2300	udp-glc 4'-epimerase, putative
LmxM.32.2340	succinyl-coA:3-ketoacid-coenzyme A transferase, mitochondrial precursor, putative

LmxM.32.2350	Rab-GTPase-TBC domain containing protein, putative
LmxM.32.2390	heat shock protein, putative
LmxM.32.2440	KH-associated protein 1
LmxM.32.2460	Flagellum attachment zone protein 12
LmxM.32.2500	Radial spokehead-like protein, putative
LmxM.32.2540	metallo-peptidase, Clan MA(E) Family M32
LmxM.32.2550	isocitrate dehydrogenase (NAD+)
LmxM.32.2570	metallo-peptidase, Clan MF, Family M17
LmxM.32.2600	enoyl-CoA hydratase/Enoyl-CoA isomerase/3- hydroxyacyl-CoA dehydrogenase, putative
LmxM.32.2730	guide RNA associated protein 1, putative
LmxM.32.2740	translation initiation factor IF-2, putative
LmxM.32.2810	transcription elongation factor-like protein
LmxM.32.2860	hypothetical protein, conserved
LmxM.32.3190	small nuclear ribonucleoprotein SmD2
LmxM.32.3210	beta prime cop protein, putative
LmxM.32.3270	fatty acid desaturase, putative
LmxM.33.0010	short chain dehydrogenase, putative
LmxM.33.0070	ascorbate peroxidase, putative
LmxM.33.0080	glucose-6-phosphate dehydrogenase
LmxM.33.0110	adenylate kinase, putative
LmxM.33.0140	malate dehydrogenase
LmxM.33.0230	intraflagellar transport protein 81, putative
LmxM.33.0350	eukaryotic translation initiation factor 5, putative
LmxM.33.0380	hypothetical protein, unknown function
LmxM.33.0390	hypothetical protein, unknown function
LmxM.33.0410	hypothetical protein, conserved
LmxM.33.0490	importin beta-1 subunit, putative
LmxM.33.0550	Cysteine leucine rich protein
LmxM.33.0650	proteasome regulatory non-ATPase subunit 11, putative
LmxM.33.0690	flagellar attachment zone protein 1
LmxM.33.0820	elongation factor 1-beta
LmxM.33.0910	hypothetical protein, conserved
LmxM.33.0990	p-glycoprotein
LmxM.33.1040	uracil phosphoribosyltransferase, putative
LmxM.33.1090	dihydroxyacetone phosphate acyltransferase, putative
LmxM.33.1170	hypothetical protein, conserved
LmxM.33.1400	d-isomer specific 2-hydroxyacid dehydrogenase- like protein
LmxM.33.1410	d-isomer specific 2-hydroxyacid dehydrogenase- like protein
LmxM.33.1415	d-isomer specific 2-hydroxyacid dehydrogenase- like protein
LmxM.33.1490	hypothetical protein, conserved
LmxM.33.1520	p25-alpha, putative
LmxM.33.2200	thioredoxin, putative
LmxM.33.2340	asparaginyl-tRNA synthetase, putative
LmxM.33.2420	glycosyltransferase-like protein
LmxM.33.2430	DNAj-like protein
LmxM.33.2470	ribosomal protein l35a, putative
LmxM.33.2530	flagellar attachment zone protein, putative (fragment)
LmxM.33.2540	Coiled-coil and C2 domain-containing protein
LmxM.33.2560	hypothetical protein, conserved
LmxM.33.2570	Flagellum attachment zone protein 8
LmxM.33.2580	ALBA-domain protein 3
LmxM.33.2610	ruvB-like DNA helicase, putative
LmxM.33.2700	eukaryotic translation initiation factor 3 subunit g
LmxM.33.2820	regulatory subunit of protein kinase a-like protein
LmxM.33.2850	serine acetyltransferase, putative
LmxM.33.2950	EF-hand domain pair, putative

LmxM.33.3050	cytochrome b5-like Heme/Steroid binding domain containing protein, putative
LmxM.33.3190	NAD dependent epimerase/dehydratase family, putative
LmxM.33.3380	aldose 1-epimerase-like protein
LmxM.33.3440	DNA topoisomerase IB, large subunit
LmxM.33.3470	amidinotransferase, putative
LmxM.33.3500	ruvb-like 1 DNA helicase, putative
LmxM.33.3560	Ankyrin repeats (3 copies)/Ankyrin repeats (many copies), putative
LmxM.33.3660	transmembrane/endomembrane-like protein
LmxM.33.3670	vacuolar ATP synthase catalytic subunit A, putative
LmxM.33.3740	serine palmitoyltransferase-like protein
LmxM.33.3820	TPR repeat, putative
LmxM.33.3880	dynein heavy chain, putative
LmxM.33.3890	hypothetical protein, conserved
LmxM.33.3970	AP-1 adapter complex gamma subunit, putative
LmxM.33.3980	nucleolar protein family a member-like protein
LmxM.33.4060	ubiquitin hydrolase, putative
LmxM.33.4120	Alpha/beta hydrolase family, putative
LmxM.33.4130	Protein of unknown function (DUF1620), putative
LmxM.33.4310	coatomer alpha subunit, putative
LmxM.33.4340	20s proteasome beta 7 subunit, (putative)
LmxM.33.4550	cell differentiation protein-like protein
LmxM.33.4600	1,2-Dihydroxy-3-keto-5-methylthiopentene dioxygenase, putative
LmxM.33.4620	cyclic nucleotide-binding domain containing protein, putative
LmxM.34.0030	pyruvate kinase, putative
LmxM.34.0050	2-oxoisovalerate dehydrogenase beta subunit, mitochondrial precursor, putative
LmxM.34.0100	ubiquinol-cytochrome c reductase complex 14kD subunit, putative
LmxM.34.0190	NLI interacting factor-like phosphatase, putative
LmxM.34.0240	60S ribosomal protein L30
LmxM.34.0320	serine palmitoyltransferase, putative
LmxM.34.0330	short chain dehydrogenase, putative
LmxM.34.0370	ATP-dependent DEAD-box RNA helicase, putative
LmxM.34.0400	40S ribosomal protein S3A, putative
LmxM.34.0820	aspartate aminotransferase, putative
LmxM.34.0950	cell cycle sequence binding phosphoprotein (RBP33), putative
LmxM.34.0980	aldose 1-epimerase-like protein
LmxM.34.1010	casein kinase, putative
LmxM.34.1050	protein kinase, putative
LmxM.34.1130	oligosaccharyl transferase-like protein
LmxM.34.1160	oligosaccharyl transferase subunit, putative
LmxM.34.1180	NADH-dependent fumarate reductase, putative
LmxM.34.1220	short chain dehydrogenase, putative
LmxM.34.1230	short chain dehydrogenase, putative
LmxM.34.1260	ATPase family associated with various cellular activities (AAA), putative
LmxM.34.1300	ubiquitin-conjugating enzyme E2, putative
LmxM.34.1330	thioredoxin, putative
LmxM.34.1380	mitochondrial processing peptidase, beta subunit, putative
LmxM.34.1410	threonyl-tRNA synthetase, putative
LmxM.34.1480	arginase
LmxM.34.1520	hypothetical protein, conserved
LmxM.34.1540	rieske iron-sulfur protein, mitochondrial precursor, putative
LmxM.34.1830	hypothetical protein, conserved
LmxM.34.1880	60S ribosomal protein L5, putative
LmxM.34.2050	60S ribosomal protein L32
LmxM.34.2080	calcium motive p-type ATPase, putative
LmxM.34.2090	kinesin, putative
LmxM.34.2150	short chain dehydrogenase, putative

LmxM.34.2200	RNA-binding protein, putative
LmxM.34.2210	kinetoplastid membrane protein-11
LmxM.34.2270	ATP-dependent RNA helicase-like protein
LmxM.34.2350	aminopeptidase P, putative
LmxM.34.2400	Flagellar Member 5
LmxM.34.2420	Autophagy-related protein 24
LmxM.34.2490	hypothetical protein, conserved
LmxM.34.2730	acyl-CoA dehydrogenase, putative
LmxM.34.2740	galactokinase-like protein
LmxM.34.2980	chaperone protein DnaJ, putative
LmxM.34.3020	phospholipase A2-like protein, putative
LmxM.34.3060	ubiquitin-activating enzyme E1, putative
LmxM.34.3080	glycerol kinase, glycosomal, putative
LmxM.34.3100	ATP-dependent RNA helicase, putative
LmxM.34.3110	Present in the outer mitochondrial membrane proteome 7
LmxM.34.3200	RNA-binding protein, putative
LmxM.34.3230	cystathione gamma lyase, putative
LmxM.34.3280	60S ribosomal subunit protein L31, putative
LmxM.34.3310	Leucine Rich repeats (2 copies), putative
LmxM.34.3340	6-phosphogluconate dehydrogenase, decarboxylating, putative
LmxM.34.3370	hypothetical protein, conserved
LmxM.34.3560	CRAL/TRIO, N-terminal domain/CRAL/TRIO domain containing protein, putative
LmxM.34.3700	glycosomal membrane protein
LmxM.34.3720	hypothetical protein, conserved
LmxM.34.3760	60S ribosomal protein L27A/L29, putative
LmxM.34.3790	60S ribosomal protein L23, putative
LmxM.34.3840	proteasome beta 2 subunit, putative
LmxM.34.3860	T-complex protein 1, eta subunit, putative
LmxM.34.3900	hypothetical protein, conserved
LmxM.34.4060	Regulatory subunit of type II PKA R-subunit, putative
LmxM.34.4130	polyadenylate-binding protein 2
LmxM.34.4380	hypothetical protein, conserved
LmxM.34.4430	mitochondrial phosphate transporter, putative
LmxM.34.4470	Co-chaperone protein P23
LmxM.34.4630	chaperone protein DnaJ, putative
LmxM.34.4690	PACRGB
LmxM.34.4700	Kinesin-13 6, putative
LmxM.34.4710	Hsp70 protein, putative
LmxM.34.4750	glyceraldehyde-3-phosphate dehydrogenase-like protein
LmxM.34.4770	peptidyl-prolyl cis-trans isomerase (cyclophilin- 40), putative
LmxM.34.4810	BRCA1 C Terminus (BRCT) domain containing protein, putative
LmxM.34.4850	proteasome alpha 1 subunit, putative
LmxM.34.4860	Mitochondrial import receptor subunit ATOM40, putative
LmxM.34.4880	TPR repeat, putative
LmxM.34.4940	hypothetical protein, conserved
LmxM.34.4950	hypothetical protein, conserved
LmxM.34.5040	polyadenylate-binding protein 1, putative
LmxM.34.5330	isopentenyl-diphosphate delta-isomerase (type II), putative
LmxM.34.5340	Dynein heavy chain and region D6 of dynein motor/Ankyrin repeats (3 copies), putative
LmxM.35.2470	hypothetical protein, conserved
LmxM.36.0050	PUF1, putative
LmxM.36.0070	stress-inducible protein STI1 homolog
LmxM.36.0180	elongation factor 2
LmxM.36.0250	eukaryotic translation initiation factor 3 subunit l
LmxM.36.0310	dolicholphosphate-mannose synthase, putative
LmxM.36.0370	phosphatidylinositol-4-phosphate 5-kinase-like protein

LmxM.36.0440	hypothetical protein, conserved
LmxM.36.0480	hypothetical protein, conserved
LmxM.36.0570	Elongation factor G1, mitochondrial, putative
LmxM.36.0720	mitogen-activated protein kinase 2
LmxM.36.0740	hypothetical protein, conserved
LmxM.36.0890	eukaryotic translation initiation factor 6 (eIF-6), putative
LmxM.36.0930	40S ribosomal protein S18, putative
LmxM.36.0950	dynein heavy chain, putative
LmxM.36.0970	hypothetical protein, conserved
LmxM.36.0980	40S ribosomal protein S10, putative
LmxM.36.0990	40S ribosomal protein S10, putative
LmxM.36.1070	ribosomal protein L24, putative
LmxM.36.1140	short chain 3-hydroxyacyl-CoA dehydrogenase, putative
LmxM.36.1260	fructose-1,6-bisphosphate aldolase
LmxM.36.1340	N-acetyltransferase subunit Nat1, putative
LmxM.36.1370	Valosin-containing protein, putative
LmxM.36.1380	hypothetical protein, conserved
LmxM.36.1430	translation elongation factor 1-beta, putative
LmxM.36.1520	Serine/threonine-protein kinase NEK17, putative
LmxM.36.1600	proteasome subunit alpha type-1, putative
LmxM.36.1630	clathrin heavy chain, putative
LmxM.36.1635	poly-zinc finger protein 2, putative
LmxM.36.1640	universal minicircle sequence binding protein, putative
LmxM.36.1650	proteasome subunit beta type-5, putative
LmxM.36.1750	hypothetical protein, conserved
LmxM.36.1850	DEAD box RNA helicase, putative
LmxM.36.1860	Ribosomal protein S5, C-terminal domain containing protein, putative
LmxM.36.1920	hypothetical protein, conserved
LmxM.36.1960	phosphomannomutase, putative
LmxM.36.2020	chaperonin HSP60, mitochondrial precursor
LmxM.36.2030	chaperonin HSP60, mitochondrial precursor
LmxM.36.2250	developmentally regulated GTP-binding protein 1, putative
LmxM.36.2310	Mitochondrial ribosomal protein S18, putative
LmxM.36.2320	glucokinase 1-like protein
LmxM.36.2350	glyceraldehyde 3-phosphate dehydrogenase, cytosolic, putative
LmxM.36.2360	tyrosine aminotransferase, putative
LmxM.36.2380	sterol 24-c-methyltransferase, putative
LmxM.36.2390	sterol 24-c-methyltransferase, putative
LmxM.36.2400	hypothetical protein, conserved
LmxM.36.2420	serine peptidase, Clan SC, Family S9B
LmxM.36.2570	membrane-bound acid phosphatase precursor
LmxM.36.2590	membrane-bound acid phosphatase 2, putative
LmxM.36.2660	dihydrolipoamide acetyltransferase precursor, putative
LmxM.36.2700	hypothetical protein, conserved
LmxM.36.2860	40S ribosomal protein S24e
LmxM.36.2950	succinyl-CoA ligase [GDP-forming] beta-chain, putative
LmxM.36.3000	ATP-dependent RNA helicase, putative
LmxM.36.3070	fibrillarin
LmxM.36.3090	hypothetical protein, conserved
LmxM.36.3100	ATP synthase, putative
LmxM.36.3210	14-3-3 protein 1, putative
LmxM.36.3270	60S ribosomal protein L22, putative
LmxM.36.3280	Enoyl-CoA hydratase/isomerase family/2-enoyl-CoA Hydratase C-terminal region, putative
LmxM.36.3380	hypothetical protein, conserved
LmxM.36.3390	ribosomal protein L29, putative
LmxM.36.3410	short chain dehydrogenase-like protein

LmxM.36.3470	2-oxoglutarate dehydrogenase E1 component, putative
LmxM.36.3530pa	polyubiquitin, putative
LmxM.36.3590	cysteine synthase, putative
LmxM.36.3620	EF-hand domain pair, putative
LmxM.36.3750	40S ribosomal protein S27-1, putative
LmxM.36.3770	transcription factor BTF3, putative
LmxM.36.3780	Spindle pole body protein 1, putative
LmxM.36.3840	glycyl tRNA synthetase, putative
LmxM.36.3880	eukaryotic translation initiation factor 3 subunit, putative
LmxM.36.3910	S-adenosylhomocysteine hydrolase
LmxM.36.4040	hypothetical protein, conserved
LmxM.36.4150	hypothetical protein, conserved
LmxM.36.4170	oxidoreductase, putative
LmxM.36.4230	paraflagellar rod component, putative
LmxM.36.4360	Regulatory particle triple-A ATPase subunit 6
LmxM.36.4450	mitochondrial intermediate peptidase, putative
LmxM.36.4460	hypothetical protein, conserved
LmxM.36.4500	Protein of unknown function (DUF2870), putative
LmxM.36.4560	PSP1 C-terminal conserved region, putative
LmxM.36.4650	60S acidic ribosomal protein, putative
LmxM.36.4780	paraflagellar rod component, putative
LmxM.36.4910	hypothetical protein, conserved
LmxM.36.5010	40S ribosomal protein SA, putative
LmxM.36.5100	hypothetical protein, conserved
LmxM.36.5120	40S ribosomal protein SA, putative
LmxM.36.5130	hypothetical protein, conserved
LmxM.36.5140	VHS domain containing protein, putative
LmxM.36.5150	kinesin-D
LmxM.36.5180	aldehyde dehydrogenase, putative
LmxM.36.5340	hypothetical protein, conserved
LmxM.36.5360	ubiquinone biosynthesis methyltransferase, putative
LmxM.36.5380	NADH dehydrogenase, putative
LmxM.36.5390	phosphoribosylpyrophosphate synthetase, putative
LmxM.36.5550	hypothetical protein, conserved
LmxM.36.5620	isoleucyl-tRNA synthetase, putative
LmxM.36.5850	flagellum targeting protein kharon1
LmxM.36.5870	paraflagellar rod component, putative
LmxM.36.5910	2,3-diketo-5-methylthio-1-phosphopentane phosphatase, putative
LmxM.36.6030	hypothetical protein, conserved
LmxM.36.6060	eukaryotic translation initiation factor 4 gamma 4
LmxM.36.6100	CCR4-NOT transcription complex subunit 10, putative
LmxM.36.6160	hypothetical protein, conserved
LmxM.36.6270	chaperone protein DnaJ, putative
LmxM.36.6290	glucose transporter 2
LmxM.36.6310	homocysteine S-methyltransferase, putative
LmxM.36.6340	ubiquitin-protein ligase, putative
LmxM.36.6370	GTPase activating protein, putative
LmxM.36.6380	rib72 protein-like protein
LmxM.36.6480	histidine secretory acid phosphatase, putative
LmxM.36.6540	similarity to endo-1-like protein
LmxM.36.6650	2,3-bisphosphoglycerate-independentphosphoglyce ra temutase
LmxM.36.6730	hypothetical protein, conserved
LmxM.36.6750	prolyl endopeptidase
LmxM.36.6760	ATP synthase delta (OSCP) subunit, putative
LmxM.36.6770	beta adaptin, putative
LmxM.36.6910	T-complex protein 1, theta subunit, putative

LmxM.36.6940	protein disulfide isomerase 2
LmxM.36.6980	eukaryotic translation initiation factor 3 subunit c
LmxM.36.6995	cytochrome oxidase subunit IX, putative

Table S4. *L. mexicana* non-poly(A) interactome. Deubiquitinases (DUBs) are highlighted in yellow

Gene ID	Protein Name
LmxM.01.0030	Kinesin-13 1, putative
LmxM.01.0060	hypothetical protein, conserved
LmxM.01.0110	glutaredoxin-like protein
LmxM.01.0140	hypothetical protein
LmxM.01.0210	CUE domain/Domain of unknown function (DUF1771)/Smr domain containing protein, putative
LmxM.01.0220	cyclophilin type peptidyl-prolyl cis-trans isomerase, putative
LmxM.01.0310	acidocalcisomal exopolyphosphatase, putative
LmxM.01.0380	Dyggve-Melchior-Clausen syndrome protein, putative
LmxM.01.0640	HSP70-like protein
LmxM.01.0650	mitochondrial-processing peptidase subunit beta, putative
LmxM.01.0690	PLAC8 family, putative
LmxM.01.0710	ubiquitin activating enzyme, putative
LmxM.02.0090	hypothetical protein, conserved
LmxM.02.0260	small GTP binding protein rab6-like protein
LmxM.02.0290	Mitogen-activated kinase kinase kinase, putative
LmxM.02.0360	casein kinase II, alpha chain, putative
LmxM.02.0410	Kinetoplastid RNA editing protein 1, putative
LmxM.02.0540	hypothetical protein, conserved
LmxM.02.0560	hypothetical protein, unknown function
LmxM.02.0660	Nop14-like family, putative
LmxM.02.0700	hypothetical protein, conserved
LmxM.03.0080	glycerol-3-phosphate acyl transferase
LmxM.03.0130	acetyltransferase, putative
LmxM.03.0160	ABC transporter protein, putative
LmxM.03.0190	U2 splicing auxiliary factor, putative
LmxM.03.0320	hypothetical protein, conserved
LmxM.03.0440	60S acidic ribosomal protein P2, putative
LmxM.03.0450	TLD domain protein, conserved
LmxM.03.0530	hypothetical protein, conserved
LmxM.03.0570	quinone oxidoreductase, putative
LmxM.03.0580	hypothetical protein, conserved
LmxM.03.0610	kinase-like protein
LmxM.03.0700	hypothetical protein, conserved
LmxM.03.0710	Aminoacyl-tRNA editing domain containing protein, putative
LmxM.03.0750	TatD related DNase, putative
LmxM.03.0760	hypothetical protein, conserved
LmxM.03.0770	Ubiquitin-like domain containing protein, putative
LmxM.03.0840	hypothetical protein, conserved
LmxM.03.0880	hypothetical protein, conserved
LmxM.03.0900	peter pan protein, putative
LmxM.03.0930	Amidohydrolase family, putative
LmxM.04.0045	hypothetical protein, conserved
LmxM.04.0060	DNA topoisomerase type IB small subunit, putative
LmxM.04.0120	exosome complex exonuclease RRP40, putative
LmxM.04.0270	Mitochondrial 39-S ribosomal protein L47 (MRP-L47), putative
LmxM.04.0290	Alcohol dehydrogenase GroES-like domain containing protein, putative
LmxM.04.0330	mitochondrial exoribonuclease DSS-1, putative
LmxM.04.0430	hypothetical protein, conserved
LmxM.04.0610	hypothetical protein, conserved
LmxM.04.0690	axonemal dynein light chain, putative
LmxM.04.0760	hypothetical protein
LmxM.04.0770	hypothetical protein
LmxM.04.0950	hypothetical protein
LmxM.04.0960	adenylate kinase, putative
LmxM.04.1080	Transcriptional repressor TCF25, putative
LmxM.04.1120	hypothetical protein
LmxM.04.1130	cytochrome c oxidase assembly factor, putative
LmxM.04.1220	hypothetical protein, conserved
LmxM.05.0010	Kinetochore interacting protein 1, putative
LmxM.05.0050	hypothetical protein, conserved

LmxM.05.0060	major vault protein, putative
LmxM.05.0190	Fip1 motif/Zinc finger C-x8-C-x5-C-x3-H type (and similar), putative
LmxM.05.0200	50S ribosome-binding GTPase, putative
LmxM.05.0240	viscerotropic leishmaniasis antigen, putative
LmxM.05.0241	viscerotropic leishmaniasis antigen, putative
LmxM.05.0390	protein kinase, putative
LmxM.05.0450	hypothetical protein, conserved
LmxM.05.0510	ATP synthase F1, alpha subunit, putative
LmxM.05.0510b	hypothetical protein
LmxM.05.0600	COG4 transport protein, putative
LmxM.05.0640	Ankyrin repeats (many copies)/Ankyrin repeats (3 copies), putative
LmxM.05.0750	Flavodoxin/Radical SAM superfamily/Wyosine base formation, putative
LmxM.05.0820	hypothetical protein, conserved
LmxM.05.0920	paraflagellar rod protein, putative
LmxM.05.1050	hypothetical protein, unknown function
LmxM.05.1060	ATPase, putative
LmxM.05.1061	ATPase, putative
LmxM.05.1130	hypothetical protein, conserved
LmxM.05.1190	hypothetical protein, conserved
LmxM.05.1200	prefoldin subunit, putative
LmxM.06.0040	39S mitochondrial ribosomal protein L46, putative
LmxM.06.0250	hypothetical protein, conserved
LmxM.06.0330	hypothetical protein, conserved
LmxM.06.0415	60S ribosomal protein L19, putative
LmxM.06.0440	hypothetical protein, conserved
LmxM.06.0460	ATP-NAD kinase-like protein
LmxM.06.0490	hypothetical protein, conserved
LmxM.06.0560	deoxyuridine triphosphatase, putative
LmxM.06.0580	60S ribosomal protein L23a, putative
LmxM.06.0610	carbonic anhydrase family protein, putative
LmxM.06.0620	Putative Phosphatase/Protein of unknown function DUF89, putative
LmxM.06.0650	lanosterol synthase, putative
LmxM.06.0700	C2 domain/Ankyrin repeats (3 copies), putative
LmxM.06.0870	protein arginine n-methyltransferase 7
LmxM.06.0920	hypothetical protein, conserved
LmxM.06.0990	hypothetical protein, conserved
LmxM.06.1000	SET domain containing protein, putative
LmxM.06.1040	Outer dynein arm docking complex protein 2, putative
LmxM.06.1070	deoxyribose-phosphate aldolase, putative
LmxM.06.1180	protein kinase, putative
LmxM.07.0050	alpha-adaptin-like protein
LmxM.07.0080	Golgi/lysosome glycoprotein 1, putative
LmxM.07.0090	cobalamin-dependent methionine synthase, putative
LmxM.07.0100	pitrilysin-like metalloprotease
LmxM.07.0120	Sec7 domain containing protein, putative
LmxM.07.0200	phosphatidylglycerolphosphate synthase, mitochondrial, putative
LmxM.07.0280	ubiquitin-protein ligase-like, putative
LmxM.07.0290	Uncharacterised protein family (UPF0172), putative
LmxM.07.0300	hypothetical protein, conserved
LmxM.07.0320	hypothetical protein, conserved
LmxM.07.0460	acyl-CoA dehydrogenase, mitochondrial precursor, putative
LmxM.07.0470	pre-rrna-processing protein tsr1 homolog
LmxM.07.0480	Dynein heavy chain, N-terminal region 2, putative
LmxM.07.0510	60S ribosomal protein L7a, putative
LmxM.07.0650	vacuolar-type Ca2+-ATPase, putative
LmxM.07.0720	hypothetical protein, conserved
LmxM.07.0802	hypothetical protein, conserved
LmxM.07.0850	ubiquitin-protein ligase, putative
LmxM.07.0940	Domain of unknown function (DUF3437), putative
LmxM.07.1080	hypothetical protein, conserved
LmxM.07.1110	hypothetical protein, conserved
LmxM.07.1120	proteasome regulatory non-ATP-ase subunit, putative
LmxM.07.1130	hypothetical protein, conserved
LmxM.07.1150	N-terminal region of Chorein, a TM vesicle-mediated sorter, putative

LmxM.08.0010	adaptor complex protein (AP) 3 delta subunit 1, putative
LmxM.08.0080	ATP-dependent DEAD/H RNA helicase, putative
LmxM.08.0120	hypothetical protein, conserved
LmxM.08.0340	hypothetical protein, conserved
LmxM.08.0430	hypothetical protein, conserved
LmxM.08.0440	hypothetical protein, conserved
LmxM.08.0510	ribose-phosphate pyrophosphokinase, putative
LmxM.08.0560	hypothetical protein, conserved
LmxM.08.0620	hypothetical protein, conserved
LmxM.08.0920	hypothetical protein, conserved
LmxM.08.0930	protein kinase, putative
LmxM.08.1000	AAA domain (dynein-related subfamily)/von Willebrand factor type A domain containing protein, putative
LmxM.08.1030a	hypothetical protein
LmxM.08.1040	hypothetical protein
LmxM.08.1060	cathepsin L-like protease, putative
LmxM.08.1080	cathepsin L-like protease, putative
LmxM.08.1170	MRB1 complex subunit MRB3010
LmxM.08.1190	intraflagellar transport protein 122B, putative
LmxM.08_29.0090	intraflagellar transport protein 27
LmxM.08_29.0220	protein transport protein Sec24A, putative
LmxM.08_29.0390	RNA binding protein, putative
LmxM.08_29.0420	hypothetical protein, conserved
LmxM.08_29.0430	hypothetical protein, conserved
LmxM.08_29.0440	hypothetical protein, conserved
LmxM.08_29.0610	hypothetical protein, conserved
LmxM.08_29.0690	NADH-ubiquinone oxidoreductase complex I subunit, putative
LmxM.08_29.0710	bardet-biedl syndrome 7 protein
LmxM.08_29.0720	Elongation factor Ts, mitochondrial, putative
LmxM.08_29.0750	U3 small nucleolar ribonucleoprotein protein MPP10, putative
LmxM.08_29.1090	ribosomal protein L1a, putative
LmxM.08_29.1110	hypothetical protein, conserved
LmxM.08_29.1570	metallo-peptidase, Clan MH, Family M18
LmxM.08_29.1690	Acyl-coenzyme A thioesterase, putative
LmxM.08_29.1730	histone H2A, putative
LmxM.08_29.1740	histone H2A, putative
LmxM.08_29.1760	paraflagellar rod protein 1D, putative
LmxM.08_29.1820	hypothetical protein, conserved
LmxM.08_29.1840	hypothetical protein, conserved
LmxM.08_29.1980	hypothetical protein, conserved
LmxM.08_29.2020	5'-AMP-activated protein kinase catalytic subunit alpha, putative
LmxM.08_29.2040	hypothetical protein, conserved
LmxM.08_29.2060	hypothetical protein, conserved
LmxM.08_29.2120	VIT family, putative
LmxM.08_29.2190	hypothetical protein, conserved
LmxM.08_29.2210	leucine-rich repeat-containing protein
LmxM.08_29.2230	hypothetical protein, conserved
LmxM.08_29.2280	acyltransferase, putative
LmxM.08_29.2300	**DUB2**; ubiquitin carboxyl-terminal hydrolase, putative
LmxM.08_29.2320	mitogen-activated protein kinase kinase 1
LmxM.08_29.2370	60S ribosomal protein L39, putative
LmxM.08_29.2380	kinesin, putative
LmxM.08_29.2390	kinesin, putative
LmxM.08_29.2430	hypothetical protein, unknown function
LmxM.08_29.2461	60S ribosomal protein L13, putative
LmxM.08_29.2540	NOL1/NOP2/sun family, putative
LmxM.08_29.2550	RNA polymerase-associated protein CTR9, putative
LmxM.08_29.2590	hypothetical protein, conserved
LmxM.08_29.2610	hypothetical protein, conserved
LmxM.08_29.2640	hypothetical protein, conserved
LmxM.08_29.2680	hypothetical protein, conserved
LmxM.08_29.2760	Putative intraflagellar transport protein C3
LmxM.08_29.2810	hypothetical protein
LmxM.09.0070	RNA helicase, putative
LmxM.09.0210	Domain of unknown function (DUF4586), putative

LmxM.09.0250	DNA replication licensing factor MCM4
LmxM.09.0400	kinetoplastid kinetochore protein 10, putative
LmxM.09.0410	kinetoplastid kinetochore protein 19, putative
LmxM.09.0530	leucine-rich repeat protein, putative
LmxM.09.0560	Histidine phosphatase superfamily (branch 1), putative
LmxM.09.0600	cyclin 1
LmxM.09.0720	cleavage and polyadenylation specificity factor 30 kDa subunit, putative
LmxM.09.0750	acyl-CoA binding protein, putative
LmxM.09.0760	hypothetical protein, conserved
LmxM.09.0790	hypothetical protein, conserved
LmxM.09.0900	ef-hand protein 5, putative
LmxM.09.0920	calmodulin, putative
LmxM.09.0930	calmodulin, putative
LmxM.09.0960	hypothetical protein, conserved
LmxM.09.0980	hypothetical protein, conserved
LmxM.09.1020	LEM3 (ligand-effect modulator 3) family / CDC50 family, putative
LmxM.09.1040	phospholipid:diacylglycerol acyltransferase, putative
LmxM.09.1180	hypothetical protein, conserved
LmxM.09.1220	AAA family ATPase, putative
LmxM.09.1230	hypothetical protein, conserved
LmxM.09.1260	Bromodomain, putative
LmxM.09.1300	PPPDE putative peptidase domain containing protein, putative
LmxM.09.1420	hypothetical protein, conserved
LmxM.09.1520	hypothetical protein, conserved
LmxM.10.0050	Dos2-interacting transcription regulator of RNA-Pol-II, putative
LmxM.10.0110	hypothetical protein, conserved
LmxM.10.0270	exonuclease, putative
LmxM.10.0310	protein transport protein SEC23, putative
LmxM.10.0460	GP63, leishmanolysin
LmxM.10.0465	GP63, leishmanolysin
LmxM.10.0500	hypothetical protein, conserved
LmxM.10.0530	hypothetical protein, conserved
LmxM.10.0560	zinc binding dehydrogenase-like protein
LmxM.10.0620	FLA1-binding protein
LmxM.10.0630	Flagellum adhesion protein 1
LmxM.10.0700	RNA recognition motif. (a.k.a. RRM, RBD, or RNP domain), putative
LmxM.10.0800	hypothetical protein, conserved
LmxM.10.0810	hypothetical protein, conserved
LmxM.10.0840	hypothetical protein, conserved
LmxM.10.0860	Ribosome biogenesis protein Nop16, putative
LmxM.10.0900	hypothetical protein, conserved
LmxM.10.0950	translation initiation factor eif-2b beta subunit, putative
LmxM.10.0970	histone H3, putative
LmxM.10.0990	histone H3
LmxM.10.1040	Domain of Unknown Function (DUF1042), putative
LmxM.10.1060	hypothetical protein, conserved
LmxM.10.1280	Beta-1,2 mannoside phosphorylase
LmxM.11.0140	hypothetical protein, conserved
LmxM.11.0150	hypothetical protein, conserved
LmxM.11.0160	hypothetical protein, conserved
LmxM.11.0260	hypothetical protein, conserved
LmxM.11.0410	Nucleolar protein 89, putative
LmxM.11.0580	cytochrome b5, putative
LmxM.11.0710	hypothetical protein, conserved
LmxM.11.0740	hypothetical protein, conserved
LmxM.11.0760	40S ribosomal protein S21, putative
LmxM.11.0770	40S ribosomal protein S21, putative
LmxM.11.0850	Alpha/beta hydrolase family, putative
LmxM.11.0900	60S ribosomal protein L24, putative
LmxM.11.0970	40S ribosomal protein S5
LmxM.11.0990	adaptin-related protein-like protein
LmxM.11.1120	hypothetical protein
LmxM.11.1130partial	hypothetical protein
LmxM.11.1190	40S ribosomal protein S15A, putative

LmxM.11.1210	SKP1-like protein
LmxM.11.1330	Flagellar-associated PapD-like, putative
LmxM.12.0090	hypothetical protein, conserved
LmxM.12.0120	hypothetical protein, conserved
LmxM.12.0220	hydroxyacylglutathione hydrolase, putative
LmxM.12.0280	ornithine decarboxylase, putative
LmxM.12.0430	hypothetical protein, conserved
LmxM.12.0600	hypothetical protein, conserved
LmxM.12.0700	hypothetical protein, conserved
LmxM.12.1180	hypothetical protein, conserved
LmxM.12.1300	SET domain containing protein, putative
LmxM.12.1320	Vacuolar sorting protein 39 domain 1/Vacuolar sorting protein 39 domain 2, putative
LmxM.13.0080	mitochondrial DNA polymerase I protein B, putative
LmxM.13.0110	SURF1 family, putative
LmxM.13.0220	ras-related protein RAB5B, putative
LmxM.13.0240	Flagella-associated protein 73, putative
LmxM.13.0390	alpha tubulin
LmxM.13.0550	Uncharacterized ACR, COG1678, putative
LmxM.13.0640	hypothetical protein, conserved
LmxM.13.0650	hypothetical protein, conserved
LmxM.13.0720	oxidoreductase-like protein
LmxM.13.0730	ubiquitin-like protein
LmxM.13.0810	hypothetical protein, conserved
LmxM.13.0920	hypothetical protein, conserved
LmxM.13.0980	amp deaminase-like protein
LmxM.13.0990	hypothetical protein, conserved
LmxM.13.1000	hypothetical protein, conserved
LmxM.13.1040	subtilisin peptidase
LmxM.13.1110	hypothetical protein, conserved
LmxM.13.1170	hypothetical protein, conserved
LmxM.13.1230	40S ribosomal protein S4, putative
LmxM.13.1340	hypothetical protein, conserved
LmxM.13.1460	programmed cell death 6 protein-like protein
LmxM.13.1520	ATPase family associated with various cellular activities (AAA), putative
LmxM.13.1560	ras-family member, GTP-binding protein, putative
LmxM.13.1600	hypothetical protein, conserved
LmxM.13.1620	squalene monooxygenase-like protein
LmxM.13.1630	mitochondrial DNA polymerase I protein D, putative
LmxM.13.1680	pyrroline-5-carboxylate reductase
LmxM.14.0080	hypothetical protein, conserved
LmxM.14.0210	Domain of unknown function (DUF4201), putative
LmxM.14.0230	hypothetical protein, conserved
LmxM.14.0280	Nucleus and spindle associated protein 3, putative
LmxM.14.0290	hypothetical protein, conserved
LmxM.14.0350	threonine synthase, putative
LmxM.14.0410	Protein of unknown function (DUF1253), putative
LmxM.14.0440	pyridine nucleotide-disulphide oxidoreductase, putative
LmxM.14.0570	WD domain, G-beta repeat, putative
LmxM.14.0780	ribosomal protein L21, putative
LmxM.14.0790	hypothetical protein, conserved
LmxM.14.0851	cysteine peptidase, Clan CA, family C2, putative
LmxM.14.0890	hypothetical protein, conserved
LmxM.14.0910	glutathione synthetase, putative
LmxM.14.0970	hypothetical protein, conserved
LmxM.14.1060	dynein heavy chain, putative
LmxM.14.1120	kinesin K39, putative
LmxM.14.1330	DnaJ domain containing protein, putative
LmxM.14.1430	Cilia and flagella associated protein 44, putative
LmxM.14.1480	glutathione-S-transferase/glutaredoxin, putative
LmxM.15.0060	Eukaryotic translation initiation factor 4 gamma 1
LmxM.15.0070	hypothetical protein, conserved
LmxM.15.0080	hypothetical protein, conserved
LmxM.15.0281	ribonucleoprotein p18, mitochondrial precursor, putative
LmxM.15.0320	Isoprenylcysteine alpha-carbonyl methylesterase, putative

LmxM.15.0330	alpha/beta hydrolase fold, putative
LmxM.15.0370	60S acidic ribosomal protein, putative
LmxM.15.0380	60S acidic ribosomal protein, putative
LmxM.15.0390	cytoplasmic l-asparaginase i-like protein
LmxM.15.0410	hypothetical protein, conserved
LmxM.15.0440a	hypothetical protein
LmxM.15.0440c	hypothetical protein (fragment)
LmxM.15.0580	signal peptidase subunit, putative
LmxM.15.0610	CRAL/TRIO domain containing protein, putative
LmxM.15.0670	hypothetical protein, conserved
LmxM.15.0780	hypothetical protein, conserved
LmxM.15.0800	hypothetical protein, conserved
LmxM.15.0810	peroxisomal membrane protein (Pex16), putative
LmxM.15.0820	GDP dissociation inhibitor, putative
LmxM.15.0890	ABC transporter, putative
LmxM.15.0970	NAD/FAD dependent dehydrogenase, putative
LmxM.15.1020	farnesyl synthetase, putative
LmxM.15.1190	cyclic nucleotide-binding domain containing protein, putative
LmxM.15.1200	protein kinase, putative
LmxM.15.1207	60S acidic ribosomal protein P2
LmxM.15.1290	mitochondrial DNA topoisomerase II
LmxM.15.1300	**DUB9**; ubiquitin hydrolase, putative
LmxM.15.1310	cation transporter, putative
LmxM.15.1350	hypothetical protein, conserved
LmxM.15.1400	hypothetical protein, conserved
LmxM.15.1420	mismatch repair protein MSH3, putative
LmxM.15.1480	cAMP specific phosphodiesterase, putative
LmxM.16.0020	AAA domain containing protein, putative
LmxM.16.0050	DEAD/DEAH box helicase/Helicase conserved C-terminal domain containing protein, putative
LmxM.16.0060	peroxisome assembly protein, putative
LmxM.16.0080	Galactose oxidase, central domain/Domain of unknown function (DUF4110), putative
LmxM.16.0160	50S ribosomal protein L17, putative
LmxM.16.0290	proteasome 26S non-ATPase subunit 9, putative
LmxM.16.0300	protein kinase, putative
LmxM.16.0310	Met-10+ like-protein, putative
LmxM.16.0380	tubulin tyrosine ligase, putative
LmxM.16.0390	XRN 5'-3' exonuclease N-terminus, putative
LmxM.16.0510	hypothetical protein, conserved
LmxM.16.0575	histone H3
LmxM.16.0600	histone H3, putative
LmxM.16.0610	histone H3, putative
LmxM.16.0660	hypothetical protein, conserved
LmxM.16.0730	**DUB10**; ubiquitin hydrolase, putative
LmxM.16.0750	hypothetical protein, unknown function
LmxM.16.0780	N-terminal region of Chorein, a TM vesicle-mediated sorter, putative
LmxM.16.0830	Iron-sulfur assembly protein 1, putative
LmxM.16.0860	hypothetical protein, conserved
LmxM.16.0920	flagellar calcium-binding protein, putative
LmxM.16.0980	hypothetical protein, conserved
LmxM.16.1010partial	hypothetical protein
LmxM.16.1070	hypothetical protein, conserved
LmxM.16.1150	hypothetical protein, conserved
LmxM.16.1170	60S ribosomal protein L39, putative
LmxM.16.1190	Domain of unknown function DUF221, putative
LmxM.16.1210	hypothetical protein, conserved
LmxM.16.1230	hypothetical protein, conserved
LmxM.16.1260	hypothetical protein, conserved
LmxM.16.1320	cytochrome c, putative
LmxM.16.1340	hypothetical protein, conserved
LmxM.16.1350	DNA-directed rna polymerase I largest subunit, putative
LmxM.16.1360	amidase, putative
LmxM.16.1370	hypothetical protein, conserved
LmxM.16.1380	small GTPase, putative
LmxM.16.1390	EMG1/NEP1 methyltransferase, putative

LmxM.16.1410	hypothetical protein, conserved
LmxM.16.1490	hypothetical protein, conserved
LmxM.16.1550	Component of motile flagella 6, putative
LmxM.16.1560	hypothetical protein, conserved
LmxM.17.0081	elongation factor 1-alpha
LmxM.17.0082	elongation factor 1-alpha
LmxM.17.0130	hypothetical protein, unknown function
LmxM.17.0350	dihydrouridine synthase (Dus), putative
LmxM.17.0490	protein kinase, putative
LmxM.17.0570	hypothetical protein, conserved
LmxM.17.0620	Acyl CoA binding protein, putative
LmxM.17.0640	VID27 cytoplasmic protein, putative
LmxM.17.0710	Myb-like DNA-binding domain containing protein, putative
LmxM.17.0720	hypothetical protein, conserved
LmxM.17.0730	GRAM domain containing protein, putative
LmxM.17.0740	GRAM domain containing protein, putative
LmxM.17.0760	Alpha/beta hydrolase family, putative
LmxM.17.0780	Acyl CoA binding protein, putative
LmxM.17.0840	hypothetical protein, conserved
LmxM.17.0850	hypothetical protein, conserved
LmxM.17.0920	ATP-dependent RNA helicase, putative
LmxM.17.0990	hypothetical protein, conserved
LmxM.17.1020	glycosyltransferase (GlcNAc), putative
LmxM.17.1110	kinesin, putative
LmxM.17.1240	hypothetical protein, conserved
LmxM.17.1250	RNA cytidine acetyltransferase
LmxM.17.1360	L-galactonolactone oxidase, putative
LmxM.17.1370	NUC130/3NT domain/SDA1, putative
LmxM.17.1410	zinc-finger protein ZPR1, putative
LmxM.18.0090	alpha glucosidase II subunit, putative
LmxM.18.0140	Domain of unknown function DUF21, putative
LmxM.18.0320	Domain of unknown function (DUF4379), putative
LmxM.18.0380	hypothetical protein, conserved
LmxM.18.0450	serine peptidase, Clan SC, Family S10
LmxM.18.0470	paralyzed flagella protein 20
LmxM.18.0550	hypothetical protein, conserved
LmxM.18.0570	hypothetical protein, conserved
LmxM.18.0610	metallo-peptidase, Clan MA(E), Family M41
LmxM.18.0650	PA26 p53-induced protein (sestrin), putative
LmxM.18.0790	DNA-directed RNA polymerase I subunit RPB5z, putative
LmxM.18.0830	Periodic tryptophan protein 2 homolog, putative
LmxM.18.0850	hypothetical protein, conserved
LmxM.18.0940	Nrap protein, putative
LmxM.18.1040	putative 5-oxoprolinase, putative
LmxM.18.1110	Multisite-specific tRNA:(cytosine-C(5))-methyltransferase, putative
LmxM.18.1250	ttaggg binding factor, putative
LmxM.18.1310	Acetyltransferase (GNAT) family, putative
LmxM.18.1360	hypothetical protein, conserved
LmxM.18.1460	hypothetical protein, conserved
LmxM.18.1510	P-type H+-ATPase, putative
LmxM.18.1530	kinesin, putative
LmxM.18.1560	hypothetical protein, conserved
LmxM.18.1590	hypothetical protein, unknown function
LmxM.18.1610	Noc2p family, putative
LmxM.19.0050	histone H2B
LmxM.19.0080	DnaJ domain containing protein, putative
LmxM.19.0110	hypothetical protein, conserved
LmxM.19.0170	hypothetical protein, conserved
LmxM.19.0270	hypothetical protein, conserved
LmxM.19.0330	intraflagellar transport protein 80, putative
LmxM.19.0470	nuclear cap binding complex subunit CBP30, putative
LmxM.19.0580	tubulin binding cofactor c, putative
LmxM.19.0660	DNA-directed RNA polymerases I and III subunit RPAC1, putative
LmxM.19.0670	U3 snoRNA-associated protein UTP11, putative

LmxM.19.0680	Flagellum attachment zone protein 7
LmxM.19.0700	kinesin, putative
LmxM.19.0810	vps53-like domain protein
LmxM.19.0970	peptidyl-prolyl cis-trans isomerase, macrophage infectivity potentiator precursor, putative
LmxM.19.1010	WD domain, G-beta repeat, putative
LmxM.19.1060	hypothetical protein, conserved
LmxM.19.1110	mitochondrial carrier protein, putative
LmxM.19.1150	hypothetical protein, conserved
LmxM.19.1380	cation transporter, putative
LmxM.19.1410	Cid1 family poly A polymerase, putative
LmxM.19.1440	mitogen-activated protein kinase 4
LmxM.19.1570	ELMO/CED-12 family, putative
LmxM.20.0010	DNA-directed RNA polymerase III, putative
LmxM.20.0140	hypothetical protein, conserved
LmxM.20.0260	Midasin, putative
LmxM.20.0370	Metallo-dependent phosphatase-like domain-containing protein
LmxM.20.0375	Metallo-dependent phosphatase-like domain-containing protein
LmxM.20.0510	SSU ribosomal protein, mitochondrial, putative
LmxM.20.0570	hypothetical protein, unknown function
LmxM.20.0650	rRNA biogenesis protein-like protein
LmxM.20.0690	hypothetical protein, conserved
LmxM.20.0700	conserved protein, unknown function
LmxM.20.0780	Tubulin/FtsZ family, putative
LmxM.20.0870	ATP-dependent RNA helicase, putative
LmxM.20.0920	AATF protein, putative
LmxM.20.0930	transmembrane protein, conserved
LmxM.20.0940	cyclophilin 15, putative
LmxM.20.1110	hypothetical protein, conserved
LmxM.20.1120	phosphatidylinositol 3-kinase, putative
LmxM.20.1130	DNAj-like protein
LmxM.20.1150	Pre-mRNA-splicing factor 38, putative
LmxM.20.1360	exosome-associated protein 1, putative
LmxM.20.1365	hypothetical protein
LmxM.20.1370	pumilio protein 9, putative
LmxM.20.1370a	hypothetical protein
LmxM.20.1430	hypothetical protein, conserved
LmxM.20.1440	hypothetical protein, conserved
LmxM.20.1620	CHORD, putative
LmxM.20.1700	hypothetical protein, conserved
LmxM.20.1710	tRNA (Uracil-5-)-methyltransferase, putative
LmxM.20.1740	hypothetical protein, conserved
LmxM.20_36.6470	mitogen-activated protein kinase
LmxM.21.0060	WD domain, G-beta repeat, putative
LmxM.21.0120	cysteine peptidase, Clan CA, family C2, putative
LmxM.21.0140	hypothetical protein, conserved
LmxM.21.0170	hypothetical protein, conserved
LmxM.21.0180	CHAT domain containing protein, putative
LmxM.21.0190	hypothetical protein, conserved
LmxM.21.0240	hexokinase, putative
LmxM.21.0250	hexokinase, putative
LmxM.21.0280	hypothetical protein, conserved
LmxM.21.0290	hypothetical protein, conserved
LmxM.21.0330	Vps51/Vps67, putative
LmxM.21.0390	hypothetical protein, conserved
LmxM.21.0470	vacuolar sorting-associated-like protein
LmxM.21.0600	hypothetical protein, conserved
LmxM.21.0640	phosphoglucomutase, putative
LmxM.21.0660	nucleotide-binding protein, putative
LmxM.21.0670	metallo-beta-lactamase family-like protein
LmxM.21.0750	Nucleolar protein 91, putative
LmxM.21.0827	hypothetical protein, conserved
LmxM.21.1020	dynein arm light chain, putative
LmxM.21.1040	kinesin, putative
LmxM.21.1070	40S ribosomal protein S23, putative

LmxM.21.1150	hypothetical protein, conserved
LmxM.21.1240	Flagellum attachment zone protein 6
LmxM.21.1270	hypothetical protein, conserved
LmxM.21.1290	vesicle-associated membrane protein, putative
LmxM.21.1310	hypothetical protein, conserved
LmxM.21.1330partial	RecF/RecN/SMC N terminal domain/SMC proteins Flexible Hinge Domain, putative
LmxM.21.1350	PB1 domain containing protein, putative
LmxM.21.1440	cytochrome b5-like Heme/Steroid binding domain containing protein, putative
LmxM.21.1470	hypothetical protein, conserved
LmxM.21.1500	hypothetical protein, conserved
LmxM.21.1550	40S ribosomal protein S11, putative
LmxM.21.1558	Present in the outer mitochondrial membrane proteome 19
LmxM.21.1561	hypothetical protein, conserved
LmxM.21.1563	choline dehydrogenase, like protein
LmxM.21.1567	peroxidase, putative
LmxM.21.1569	Exocyst complex component EXO99
LmxM.21.1660	mitochondrial structure specific endonuclease I (SSE-1), putative
LmxM.21.1750	hypothetical protein, conserved
LmxM.22.0030	60S ribosomal protein L11 (L5, L16)
LmxM.22.0080	heat shock protein DNAJ, putative
LmxM.22.0140	hypothetical protein, conserved
LmxM.22.0160	hypothetical protein, conserved
LmxM.22.0170	hypothetical protein, conserved
LmxM.22.0220	Domain of unknown function (DUF1736), putative
LmxM.22.0380	Nucleoporin NUP132
LmxM.22.0450	Acetyltransferase (GNAT) family, putative
LmxM.22.0460	40S ribosomal protein S15, putative
LmxM.22.0560	kinesin, putative
LmxM.22.0650	guide RNA associated protein, GAP2, putative
LmxM.22.0710	hypothetical protein, conserved
LmxM.22.0780	NADH-cytochrome b5 reductase, putative
LmxM.22.0790	translation initiation factor SUI1, putative
LmxM.22.0860	Protein of unknown function (DUF3184), putative
LmxM.22.0900	Component of motile flagella 10, putative
LmxM.22.0910	Prp18 domain containing protein, putative
LmxM.22.0930	mitochondrial chaperone, putative
LmxM.22.0940	mitochondrial chaperone, putative
LmxM.22.0970	hypothetical protein, conserved
LmxM.22.0980	F-box and WD40 domain containing protein
LmxM.22.1040	hypothetical protein, conserved
LmxM.22.1200	ChaC-like protein, putative
LmxM.22.1240	translation initiation factor IF-2, putative
LmxM.22.1320	flagellar attachment zone protein, putative (fragment)
LmxM.22.1410	centrin-4, putative
LmxM.22.1450	cyclophilin, putative
LmxM.22.1560	40S ribosomal protein L14, putative
LmxM.22.1580	exosome complex exonuclease RRP45 homolog, putative
LmxM.22.1600	ser/thr protein phosphatase, putative
LmxM.22.1610	hypothetical protein, conserved
LmxM.22.1620	Cilia and flagella associated protein 47, putative
LmxM.22.1630	ccr4-not transcription complex subunit
LmxM.23.0010	hypothetical protein, conserved
LmxM.23.0020	hypothetical protein, conserved
LmxM.23.0030	Beta propeller protein 1
LmxM.23.0090	Domain of unknown function (DUF1767), putative
LmxM.23.0150	hypothetical protein, conserved
LmxM.23.0380	ATP-binding cassette protein subfamily G, member 5
LmxM.23.0480	hypothetical protein, conserved
LmxM.23.0543	5'-3' exoribonuclease C, putative
LmxM.23.0620	WD domain, G-beta repeat/BING4CT (NUC141) domain containing protein, putative
LmxM.23.0810	actin interacting protein-like protein
LmxM.23.0830	Na/H antiporter-like protein
LmxM.23.0840	hypothetical protein, unknown function
LmxM.23.0870	hypothetical protein

LmxM.23.0880a	hypothetical protein
LmxM.23.1010	hypothetical protein, conserved
LmxM.23.1065	hypothetical protein, conserved
LmxM.23.1190	hypothetical protein, conserved
LmxM.23.1290	hypothetical protein, unknown function
LmxM.23.1450	Tumour suppressor, Mitostatin, putative
LmxM.23.1630	hypothetical protein, conserved
LmxM.24.0080	Ring finger domain containing protein, putative
LmxM.24.0110	poly A polymerase regulatory subunit, putative
LmxM.24.0130	WD40/YVTN repeat-like-containing protein
LmxM.24.0160	hypothetical protein, conserved
LmxM.24.0190	hypothetical protein, conserved
LmxM.24.0250	ATP-dependent DEAD/H RNA helicase, putative
LmxM.24.0300	hypothetical protein, conserved
LmxM.24.0330	Mitochondrial small ribosomal subunit Rsm22, putative
LmxM.24.0420	**DUB15**; ubiquitin carboxyl-terminal hydrolase, putative,cysteine peptidase, Clan CA, family C12, putative
LmxM.24.0440	Protein of unknown function (DUF3184), putative
LmxM.24.0450	Protein of unknown function (DUF3184), putative
LmxM.24.0520	DNAJ domain protein, putative
LmxM.24.0620	**DUB12**; ubiquitin carboxyl-terminal hydrolase, putative
LmxM.24.0730	signal recognition particle, putative
LmxM.24.0830	Mitochondrial SSU ribosomal protein, putative
LmxM.24.0910	minchromosome maintenance (MCM) complex subunit, putative
LmxM.24.0990	replication factor C, subunit 1, putative
LmxM.24.1080	DNAJ domain protein, putative
LmxM.24.1140	hypothetical protein, conserved
LmxM.24.1150	DNA-directed RNA polymerase, mitochondrial
LmxM.24.1220	RNA-binding protein 38, putative
LmxM.24.1440	HpcH/HpaI aldolase/citrate lyase family, putative
LmxM.24.1470	hypothetical protein, conserved
LmxM.24.1490	WD domain, G-beta repeat, putative
LmxM.24.1500a	hypothetical protein
LmxM.24.1770	inhibitor of cysteine peptidase
LmxM.24.1780	fatty acid transporter protein-like protein
LmxM.24.1790	RNA recognition motif. (a.k.a. RRM, RBD, or RNP domain), putative
LmxM.24.1850	hypothetical predicted multi-pass transmembrane protein
LmxM.24.1870	hypothetical protein, conserved
LmxM.24.1950	hypothetical protein, conserved
LmxM.24.1970	Nucleoporin NUP76
LmxM.24.2020	tubulin folding cofactor D, putative
LmxM.24.2080	40S ribosomal protein S8, putative
LmxM.24.2250	fatty-acid desaturase, putative
LmxM.24.2260	notchless homolog, putative
LmxM.24.2290	cullin-like protein-like protein
LmxM.25.0150	hypothetical protein, conserved
LmxM.25.0170	cleavage and polyadenylation specificity factor, putative
LmxM.25.0200	Putative intraflagellar transport protein F6
LmxM.25.0220	hypothetical protein, conserved
LmxM.25.0250	hypothetical protein, conserved
LmxM.25.0270	Nucleoporin NUP110
LmxM.25.0290	RNA-binding protein, putative
LmxM.25.0320	B9 domain-containing protein 2
LmxM.25.0410	translation initiation factor SUI1, putative
LmxM.25.0420	hypothetical protein, conserved
LmxM.25.0430	WD domain, G-beta repeat/Utp13 specific WD40 associated domain containing protein, putative
LmxM.25.0440	transcription factor IIIb, putative
LmxM.25.0470	Kringle domain containing protein, putative
LmxM.25.0580	hypothetical protein, conserved
LmxM.25.0730	eukaryotic initiation factor 5a, putative
LmxM.25.0820	prefoldin subunit, putative
LmxM.25.0960	tubulin gamma chain
LmxM.25.1010	2OG-Fe(II) oxygenase superfamily, putative
LmxM.25.1020	dehydrogenase-like protein
LmxM.25.1100	DNAj-like protein

LmxM.25.1270	hypothetical protein, conserved
LmxM.25.1310	hypothetical protein, conserved
LmxM.25.1320	serine/threonine-protein phosphatase
LmxM.25.1430	Putative intraflagellar transport protein A1
LmxM.25.1570	ER-golgi trafficking TRAPP I complex 85 kDa subunit, putative
LmxM.25.1630	diacylglycerol acyltransferase, putative
LmxM.25.1660	hypothetical protein, conserved
LmxM.25.1720	hypothetical protein, conserved
LmxM.25.1920	tetratricopeptide repeat protein 29
LmxM.25.2040	hypothetical protein, conserved
LmxM.25.2060	Stage II sporulation protein E (SpoIIE), putative
LmxM.25.2080	elongation factor 2-like protein
LmxM.25.2110	KIF-1 binding protein C terminal, putative
LmxM.25.2120	eukaryotic translation initiation factor 3 subunit j
LmxM.25.2140	succinyl-CoA synthetase alpha subunit, putative
LmxM.25.2190	DnaJ domain/Zinc-finger double-stranded RNA-binding, putative
LmxM.25.2370	adenylate kinase, putative
LmxM.26.0010a	hypothetical protein
LmxM.26.0180	60S ribosomal protein L7, putative
LmxM.26.0200	hypothetical protein, conserved
LmxM.26.0440	hypothetical protein, conserved
LmxM.26.0630	hypothetical protein, conserved
LmxM.26.0640	10 kDa heat shock protein, putative
LmxM.26.0730	hypothetical protein, conserved
LmxM.26.0840	hypothetical protein, conserved
LmxM.26.0870	ISWI complex protein
LmxM.26.0890	40S ribosomal protein S16, putative
LmxM.26.0940	DnaJ domain/Domain of unknown function (DUF3395), putative
LmxM.26.0970	NMD3 family, putative
LmxM.26.0980	protein kinase, putative
LmxM.26.1010	hypothetical protein, conserved
LmxM.26.1030	hypothetical protein, conserved
LmxM.26.1120	axonemal dynein light chain, putative
LmxM.26.1220	hypothetical protein, conserved
LmxM.26.1250	hypothetical protein, conserved
LmxM.26.1300	Nucleolar protein 105, putative
LmxM.26.1310	hypothetical protein, conserved
LmxM.26.1420	FYVE zinc finger, putative
LmxM.26.1540	glutaredoxin, putative
LmxM.26.1620	CDP-DAG synthase, putative
LmxM.26.1640	40S ribosomal protein S33, putative
LmxM.26.1750	hypothetical protein, conserved
LmxM.26.1860	Present in the outer mitochondrial membrane proteome 2
LmxM.26.1950	microtubule-associated protein, putative
LmxM.26.1980	hypothetical protein, conserved
LmxM.26.2010	hypothetical protein, conserved
LmxM.26.2030	hypothetical protein, conserved
LmxM.26.2200	hypothetical protein, conserved
LmxM.26.2220	ribosomal protein L38, putative
LmxM.26.2230	GDP-mannose 4,6 dehydratase, putative
LmxM.26.2280	nitrilase, putative
LmxM.26.2300	hypothetical protein, conserved
LmxM.26.2320	hypothetical protein, conserved
LmxM.26.2360	sec1 family transport protein, putative
LmxM.26.2370	SPRY domain/HECT-domain (ubiquitin-transferase), putative
LmxM.26.2380	Fibronectin type III domain containing protein, putative
LmxM.26.2410	hypothetical protein, conserved
LmxM.26.2480	phosphatidyltransferase, putative
LmxM.26.2490	hypothetical protein, conserved
LmxM.26.2520	hypothetical protein, conserved
LmxM.26.2590	Paraquat-inducible protein A, putative
LmxM.26.2600	hypothetical protein, conserved
LmxM.27.0090	cytochrome p450-like protein
LmxM.27.0200	hypothetical protein, conserved

LmxM.27.0310	TATA-binding protein interacting (TIP20), putative
LmxM.27.0380	Nuclear pore complex protein 158
LmxM.27.0530	hypothetical protein, conserved
LmxM.27.0550	replication factor C, subunit 4, putative
LmxM.27.0580	hypothetical protein, conserved
LmxM.27.0590	Domain of unknown function (DUF4470)/Domain of unknown function (DUF4471), putative
LmxM.27.0630	Mitochondrial SSU ribosomal protein, putative
LmxM.27.0700	hypothetical protein, conserved
LmxM.27.0820	parallel beta-helix repeat, putative
LmxM.27.0860	WD domain, G-beta repeat, putative
LmxM.27.0910	Pep3/Vps18/deep orange family/Region in Clathrin and VPS, putative
LmxM.27.1050	vesicular transport protein (CDC48 homologue), putative
LmxM.27.1070	vesicular-fusion ATPase-like protein, putative
LmxM.27.1130	intraflagellar transport protein 88, putative
LmxM.27.1190	histone H1, putative
LmxM.27.1210	translation initiation factor eIF2B delta subunit, putative
LmxM.27.1230	hypothetical protein, conserved
LmxM.27.1240	histone H1, putative
LmxM.27.1290	predicted leucine-rich repeat protein
LmxM.27.1390	hypothetical protein
LmxM.27.1440	Snf7, putative
LmxM.27.1480	cyclic nucleotide-binding domain containing protein, putative
LmxM.27.1490	hypothetical protein, conserved
LmxM.27.1630	inner dynein arm I1 intermediate chain, axonemal
LmxM.27.1690	mRNA export factor MEX67
LmxM.27.1750	dynein heavy chain, putative
LmxM.27.1760	Tubulin cofactor C domain-containing protein 1, putative
LmxM.27.1790	hypothetical protein, conserved
LmxM.27.1810	Phosphoenolpyruvate carboxykinase [ATP], glycosomal
LmxM.27.1830	GTP binding protein, putative
LmxM.27.1850	paraflagellar rod protein 5, putative
LmxM.27.1895	hypothetical protein, conserved
LmxM.27.1930	hypothetical protein, unknown function
LmxM.27.1940	cdc2-related kinase 9
LmxM.27.1950	hypothetical protein, conserved
LmxM.27.1990	Rab-GTPase-TBC domain containing protein, putative
LmxM.27.2031	branched-chain amino acid aminotransferase, putative
LmxM.27.2032	branched-chain amino acid aminotransferase, putative
LmxM.27.2050	ribonucleoside-diphosphate reductase small chain, putative
LmxM.27.2060	hypothetical protein, conserved
LmxM.27.2130	hypothetical protein, conserved
LmxM.27.2170	hypothetical protein, conserved
LmxM.27.2200	thioredoxin, putative
LmxM.27.2220	ribosomal protein L11, putative
LmxM.27.2250	EMG1/NEP1 methyltransferase, putative
LmxM.27.2470	protein kinase, putative
LmxM.27.2600	hypothetical protein, conserved
LmxM.28.0050	nexin-dynein regulatory complex 2
LmxM.28.0060	hypothetical protein, conserved
LmxM.28.0160	RNA binding protein, putative
LmxM.28.0240	glycerol-3-phosphate dehydrogenase, putative
LmxM.28.0330	rrp44p homologue, putative
LmxM.28.0400	serine peptidase, Clan SJ, family S16, putative
LmxM.28.0450	DIS3-like exonuclease, putative
LmxM.28.0470	hypothetical protein, conserved
LmxM.28.0510	hypothetical protein, conserved
LmxM.28.0530	RAD50 DNA repair-like protein
LmxM.28.0550	DNA repair protein RAD51, putative
LmxM.28.0590	hypothetical protein, unknown function
LmxM.28.0610	dynein heavy chain, putative
LmxM.28.0680	Ankyrin repeats (3 copies), putative
LmxM.28.0720	Sas10/Utp3/C1D family/Sas10 C-terminal domain containing protein, putative
LmxM.28.0760	DNA repair protein, putative
LmxM.28.0780	kinetoplast poly(A) polymerase 1

LmxM.28.0850	minichromosome maintenance (MCM) complex subunit, putative
LmxM.28.0870	hypothetical protein, conserved
LmxM.28.0950	Tctex-1 family, putative
LmxM.28.1011	ribosomal protein S20, putative
LmxM.28.1030	ribosomal protein S20, putative
LmxM.28.1040	hypothetical protein, unknown function
LmxM.28.1190	copine i-like protein
LmxM.28.1220	hypothetical protein, conserved
LmxM.28.1240	p450 reductase, putative
LmxM.28.1280	phenylalanine-4-hydroxylase, putative
LmxM.28.1310	ATP-dependent RNA helicase, putative
LmxM.28.1350	haloacid dehalogenase-like hydrolase-like protein
LmxM.28.1360	haloacid dehalogenase-like hydrolase-like protein
LmxM.28.1380	haloacid dehalogenase-like hydrolase-like protein
LmxM.28.1530	DEAD box RNA helicase, putative
LmxM.28.1630	Domain of unknown function (DUF3342), putative
LmxM.28.1640	hypothetical protein, conserved
LmxM.28.1660	hypothetical protein, conserved
LmxM.28.1800	hypothetical protein, conserved
LmxM.28.1910	p21-C-terminal region-binding protein, putative
LmxM.28.1930	Zinc transporter 3, putative
LmxM.28.1940	divalent cation transporter, putative
LmxM.28.1980	hypothetical protein, conserved
LmxM.28.2040	DPCD protein family, putative
LmxM.28.2070	tRNA threonylcarbamoyl adenosine modification protein, Sua5/YciO/YrdC/YwlC family, putative
LmxM.28.2120	cullin 2, putative
LmxM.28.2160	methyltransferase domain containing protein, putative
LmxM.28.2180	hypothetical protein, conserved
LmxM.28.2200	Mannosyl oligosaccharide glucosidase, putative
LmxM.28.2280	DNA topoisomerase ii
LmxM.28.2290	Translation initiation factor eIF-2B subunit epsilon, putative
LmxM.28.2330	Cactus-binding C-terminus of cactin protein, putative
LmxM.28.2440	hypothetical protein, conserved
LmxM.28.2470	MRP protein-like protein
LmxM.28.2500	Eukaryotic translation initiation factor 4E-3
LmxM.28.2520	R3H domain containing protein, putative
LmxM.28.2540	hypothetical protein, conserved
LmxM.28.2560	40S ribosomal protein S17, putative
LmxM.28.2570	splicing factor 3B subunit 1, putative
LmxM.28.2580	leucine-rich repeat protein, putative
LmxM.28.2590	ADP-ribosylglycohydrolase, putative
LmxM.28.2670	protein phosphatase 4 catalytic subunit, putative
LmxM.28.2730	hypothetical protein, conserved
LmxM.28.2740a	hypothetical protein
LmxM.28.2760	gamma-tubulin complex component 3
LmxM.28.2880	dynein heavy chain, putative
LmxM.29.0020	cyclophilin, putative
LmxM.29.0050	MATH domain containing protein, putative
LmxM.29.0200	hypothetical protein, conserved
LmxM.29.0210	ribosomal RNA adenine dimethylase family protein, putative
LmxM.29.0250	**DUB13**; ubiquitin hydrolase, putative
LmxM.29.0410	WD-40 repeat protein
LmxM.29.0450	Eukaryotic translation initiation factor 4E-4
LmxM.29.0470	hypothetical protein, conserved
LmxM.29.0520	N-acetyltransferase B complex (NatB) non catalytic subunit, putative
LmxM.29.0590	Bardet-Biedl syndrome 2 protein
LmxM.29.0620	ribosome biogenesis regulatory protein (RRS1), putative
LmxM.29.0650	hypothetical protein, conserved
LmxM.29.0700	NUDC-like protein
LmxM.29.0800	rac serine-threonine kinase, putative
LmxM.29.0805	hypothetical protein, conserved
LmxM.29.0810	AdoMet dependent proline di-methyltransferase, putative
LmxM.29.0860	surface protein amastin, putative
LmxM.29.0930	hypothetical protein, conserved

LmxM.29.1000	protein kinase, putative
LmxM.29.1010	ubiquitin fusion degradation protein 2, putative
LmxM.29.1070	Flavinator of succinate dehydrogenase, putative
LmxM.29.1100	DREV methyltransferase, putative
LmxM.29.1205	small nuclear ribonucleoprotein polypeptide e, putative
LmxM.29.1230	Zinc finger, C3HC4 type (RING finger), putative
LmxM.29.1240	calcium-binding protein, putative
LmxM.29.1270	hypothetical protein, conserved
LmxM.29.1360	serine/threonine protein kinase RIO2, putative
LmxM.29.1450	kinesin, putative
LmxM.29.1590	hypothetical protein, conserved
LmxM.29.1710	small GTP-binding protein Rab28, putative
LmxM.29.1730	hypothetical protein, conserved
LmxM.29.1760	microtubule associated protein xmap215
LmxM.29.1800	queuine tRNA-ribosyltransferase, putative
LmxM.29.1810	Hydin, putative
LmxM.29.1830	splicing factor 3a, putative
LmxM.29.1890	Adenylate kinase, nuclear
LmxM.29.2070	Domain of unknown function (DUF814), putative
LmxM.29.2090	alcohol dehydrogenase, putative
LmxM.29.2100	hypothetical protein, conserved
LmxM.29.2250	zinc-finger protein, conserved
LmxM.29.2310	hypothetical protein, conserved
LmxM.29.2390	hypothetical protein, conserved
LmxM.29.2460	heat shock 70-related protein 1, mitochondrial precursor, putative
LmxM.29.2560	hypothetical protein, conserved
LmxM.29.2570	hypothetical protein, conserved
LmxM.29.2630	replication factor C, subunit 2, putative
LmxM.29.2660	hypothetical protein, conserved
LmxM.29.2670	hypothetical protein, conserved
LmxM.29.2750	hypothetical protein, conserved
LmxM.29.2845	succinate dehydrogenase subunit 3
LmxM.29.2890	hypothetical protein, conserved
LmxM.29.3015	Lsm5p, putative
LmxM.29.3060	kinesin, putative
LmxM.29.3080	homoserine kinase
LmxM.29.3170	Tumour suppressor, Mitostatin, putative
LmxM.29.3200	ribosomal protein S26, putative
LmxM.29.3220	hypothetical protein, conserved
LmxM.29.3250	ATP-dependent RNA helicase, putative
LmxM.29.3260	Ankyrin repeats (3 copies), putative
LmxM.29.3340	60S ribosomal protein L9, putative
LmxM.29.3390	CAS/CSE/importin domain protein, putative
LmxM.29.3410	hypothetical protein, conserved
LmxM.29.3420	T-complex protein 11, putative
LmxM.29.3450	hypothetical protein, conserved
LmxM.29.3590	40S ribosomal protein S14
LmxM.29.3640	Component of motile flagella protein 46
LmxM.29.3710	CPSF-domain protein, putative
LmxM.29.3730	60S acidic ribosomal protein P2, putative
LmxM.30.0090	hypothetical protein, conserved
LmxM.30.0130	hypothetical protein, unknown function
LmxM.30.0160	DNA-directed RNA polymerase II subunit 2, putative
LmxM.30.0200	hypothetical protein, conserved
LmxM.30.0210	hypothetical protein, conserved
LmxM.30.0211	hypothetical protein, conserved
LmxM.30.0230	hypothetical protein, conserved
LmxM.30.0480	hypothetical protein, conserved
LmxM.30.0480b	hypothetical protein
LmxM.30.0540	ATP-binding cassette protein subfamily D, member 2, putative
LmxM.30.0550	hypothetical protein, conserved
LmxM.30.0700	metallo-peptidase, Clan MA(E), Family M41
LmxM.30.0720	major facilitator superfamily, putative
LmxM.30.0740	Tir chaperone protein (CesT) family, putative

LmxM.30.0950	hypothetical protein
LmxM.30.0980	hypothetical protein, conserved
LmxM.30.1000	hypothetical protein, conserved
LmxM.30.1100	Domain of unknown function (DUF4498), putative
LmxM.30.1210	Protein of unknown function (DUF3638)/Protein of unknown function (DUF3645)/Zn-finger in Ran binding protei
LmxM.30.1250	hypothetical protein, conserved
LmxM.30.1270	p-glycoprotein e
LmxM.30.1370	hypothetical protein, conserved
LmxM.30.1380	hypothetical protein, unknown function
LmxM.30.1400	hypothetical protein, conserved
LmxM.30.1610	diphthine synthase-like protein
LmxM.30.1720	Cullin protein neddylation domain containing protein, putative
LmxM.30.1870	mannosyltransferase-like protein
LmxM.30.1880	mannosyltransferase-like protein
LmxM.30.1885	mannosyltransferase-like protein
LmxM.30.1930	dihydrouridine synthase (Dus), putative
LmxM.30.1970	tryparedoxin 4, putative
LmxM.30.2030	ubiquitin-fusion protein
LmxM.30.2170	ARM-like helical domain-containing protein
LmxM.30.2270	hypothetical protein, conserved
LmxM.30.2310	3'-nucleotidase/nuclease
LmxM.30.2590	TerD domain containing protein, putative
LmxM.30.2700	hypothetical protein, conserved
LmxM.30.2770	RNA recognition motif. (a.k.a. RRM, RBD, or RNP domain), putative
LmxM.30.2780	hypothetical protein, unknown function
LmxM.30.2890	Region in Clathrin and VPS, putative
LmxM.30.2900	Nucleoporin NUP62
LmxM.30.2940	farnesyltransferase, putative
LmxM.30.2990	hypothetical protein, conserved
LmxM.30.3060	ferrous iron transport protein
LmxM.30.3070	ferrous iron transport protein
LmxM.30.3140	hypothetical protein, conserved
LmxM.30.3150	Cilia and flagella associated protein 43, putative
LmxM.30.3180	histone H4
LmxM.31.0070	syntaxin 5, putative
LmxM.31.0130	hypothetical protein, conserved
LmxM.31.0140	Flagellum attachment zone protein 9
LmxM.31.0150	Utp14 protein, putative
LmxM.31.0160	hypothetical protein, unknown function
LmxM.31.0190	hypothetical protein, conserved
LmxM.31.0200	hypothetical protein, conserved
LmxM.31.0210	Histone chaperone Rttp106-like, putative
LmxM.31.0250	hypothetical protein, conserved
LmxM.31.0310	intraflagellar transport protein 140, putative
LmxM.31.0370	root hair defective 3 GTP-binding protein (RHD3), putative
LmxM.31.0410	hypothetical protein, conserved
LmxM.31.0430	60S ribosomal protein L17, putative
LmxM.31.0450	40S ribosomal protein S2
LmxM.31.0460	prostaglandin f synthase, putative
LmxM.31.0470	hypothetical protein, conserved
LmxM.31.0570	ATP- dependent RNA helicase, putative
LmxM.31.0600	hypothetical protein, conserved
LmxM.31.0620	hypothetical protein, conserved
LmxM.31.0690	hypothetical protein, conserved
LmxM.31.0820	CYC2-like cyclin, putative
LmxM.31.0850	polypyrimidine tract-binding protein, putative
LmxM.31.0890	ethanolamine-phosphate cytidylyltransferase
LmxM.31.0910	Alpha/beta hydrolase family, putative
LmxM.31.0930	FAD dependent oxidoreductase, putative
LmxM.31.0960	ubiquitin-conjugating enzyme protein, putative
LmxM.31.0970	cysteine peptidase, Clan CA, family C2, putative
LmxM.31.1070	small nuclear ribonucleoprotein, putative
LmxM.31.1090	HECT-domain (ubiquitin-transferase), putative
LmxM.31.1100	exportin 1, putative

LmxM.31.1130	hypothetical protein, conserved
LmxM.31.1210	pentatricopeptide repeat domain containing protein, putative
LmxM.31.1220	hypothetical protein, conserved
LmxM.31.1230	ADP-ribosylation factor GTPase activating protein 1, putative
LmxM.31.1340	cleavage and polyadenylation specificity factor- like protein
LmxM.31.1360	RNA capping enzyme, cytoplasmic
LmxM.31.1370	G5-interacting protein, putative
LmxM.31.1430	hypothetical protein, conserved
LmxM.31.1610	hypothetical protein, conserved
LmxM.31.1680	Paraflagellar Rod Proteome Component 9, putative
LmxM.31.1690	protein phosphatase 2C, putative
LmxM.31.1802	hypothetical protein, conserved
LmxM.31.1810	protein kinase, putative
LmxM.31.1840	rab11B GTPase, putative
LmxM.31.1870	Cation efflux family, putative
LmxM.31.2000	TLR4 regulator and MIR-interacting MSAP, putative
LmxM.31.2030	Ras-related protein RAB2B, putative
LmxM.31.2060	ABC transporter-like protein
LmxM.31.2130	RNA recognition motif. (a.k.a. RRM, RBD, or RNP domain), putative
LmxM.31.2160	R-SNARE protein, putative
LmxM.31.2200	U5 small nuclear ribonucleoprotein component, putative
LmxM.31.2230	ATP-dependent RNA helicase, putative
LmxM.31.2340	cytochrome-b5 reductase, putative
LmxM.31.2350	Nucleoporin NUP53a
LmxM.31.2530	cyclic nucleotide-binding domain containing protein, putative
LmxM.31.2550	adenosine monophosphate deaminase-like protein
LmxM.31.2610	Tip Of Extending FAZ protein 1
LmxM.31.2650	SpoU rRNA Methylase family, putative
LmxM.31.2670	kinteoplast poly(A) polymerase complex 1 subunit, putative
LmxM.31.2730	hypothetical protein, conserved
LmxM.31.2750	Guanine nucleotide exchange factor in Golgi transport N-terminal/Sec7 domain containing protein, putative
LmxM.31.2890	SNAP protein, putative
LmxM.31.2910	**DUB19**; ubiquitin hydrolase, putative
LmxM.31.2951	nucleoside diphosphate kinase b
LmxM.31.3030	chaperone protein DnaJ, putative
LmxM.31.3040	protein phosphatase 2A catalytic subunit, putative
LmxM.31.3180	mitochondrial RNA binding protein, putative
LmxM.31.3300	chaperone protein DnaJ, putative
LmxM.31.3340	hypothetical protein, conserved
LmxM.31.3380	tRNA pseudouridine synthase D (TruD), putative
LmxM.31.3410	Dopey, N-terminal, putative
LmxM.31.3430	Uncharacterised protein family UPF0564, putative
LmxM.31.3431	Uncharacterised protein family UPF0564, putative
LmxM.31.3650	hypothetical protein
LmxM.31.3680	enoyl-CoA hydratase/isomerase family protein, putative
LmxM.31.3760	enolase, putative
LmxM.31.3800	hypothetical protein, conserved
LmxM.31.3810	SHQ1 protein, putative
LmxM.31.3930	SPRY domain/HECT-domain (ubiquitin-transferase), putative
LmxM.31.3990	GIPL galf transferase, putative
LmxM.32.0060	WD domain, G-beta repeat, putative
LmxM.32.0150	hypothetical protein, conserved
LmxM.32.0190	hypothetical protein, conserved
LmxM.32.0270	Mpv17 / PMP22 family, putative
LmxM.32.0280	hypothetical protein, conserved
LmxM.32.0295	autophagocytosis protein, putative
LmxM.32.0314	heat shock protein 83-1
LmxM.32.0316	heat shock protein 83-1
LmxM.32.0410	DNA mismatch repair protein MSH2, putative
LmxM.32.0535	hypothetical protein, conserved
LmxM.32.0550	hypothetical protein, conserved
LmxM.32.0650	hypothetical protein, conserved
LmxM.32.0720	60S ribosomal protein L6, putative
LmxM.32.0765	hypothetical protein, conserved

LmxM.32.0920	40S ribosomal protein S3, putative
LmxM.32.0960	nicotinate phosphoribosyltransferase, putative
LmxM.32.0970	hypothetical protein, conserved
LmxM.32.1030	Protein prenyltransferase alpha subunit repeat, putative
LmxM.32.1090	guanylate kinase, putative
LmxM.32.1140	hypothetical protein, conserved
LmxM.32.1170	hypothetical protein, conserved
LmxM.32.1180	Axonemal inner arm dynein light chain, putative
LmxM.32.1200	ATPase family associated with various cellular activities (AAA), putative
LmxM.32.1300	ABC1 family protein, putative
LmxM.32.1490	hypothetical protein, conserved
LmxM.32.1500	hypothetical protein, conserved
LmxM.32.1620	hypothetical protein, conserved
LmxM.32.1680	thiamine biosynthesis-like protein
LmxM.32.1700	chromatin-remodeling complex atpase chain iswi
LmxM.32.1780	hypothetical protein, conserved
LmxM.32.1790	Histone methylation protein DOT1, putative
LmxM.32.1810	hypothetical protein, conserved
LmxM.32.1860	ABC transporter, putative
LmxM.32.1910	ribosome assembly protein RRB1, putative
LmxM.32.1930	phosphoribosylpyrophosphate synthetase, putative
LmxM.32.1970	mitochondrial
LmxM.32.2040	Nucleoporin NUP89
LmxM.32.2050	hypothetical protein, conserved
LmxM.32.2060	hypothetical protein, conserved
LmxM.32.2070	mitogen-activated protein kinase, putative
LmxM.32.2140	kinesin, putative
LmxM.32.2150	hypothetical protein, conserved
LmxM.32.2200	hypothetical protein, unknown function
LmxM.32.2220	Flagella-associated protein 91, putative
LmxM.32.2370	Nucleoporin NUP59
LmxM.32.2400	GTPase protein, putative
LmxM.32.2510	Mitochondrial SSU ribosomal protein, putative
LmxM.32.2530	NUC173 domain containing protein, putative
LmxM.32.2610	Mitochondrial-processing peptidase subunit alpha
LmxM.32.2630	dynein intermediate chain, putative
LmxM.32.2700	minichromosome maintenance (MCM) complex subunit, putative
LmxM.32.2960	hypothetical protein, conserved
LmxM.32.3050	tyrosyl-DNA phosphodiesterase-like protein
LmxM.32.3070partial	hypothetical protein
LmxM.32.3090	Holliday-junction resolvase-like of SPT6/SH2 domain containing protein, putative
LmxM.32.3150	40S ribosomal protein S13, putative
LmxM.32.3160	hypothetical protein, conserved
LmxM.33.0130	malate dehydrogenase, putative
LmxM.33.0150	malate dehydrogenase, putative
LmxM.33.0160	malate dehydrogenase, putative
LmxM.33.0190	Domain of unknown function (DUF1935), putative
LmxM.33.0250	hypothetical protein, conserved
LmxM.33.0300	hypothetical protein, conserved
LmxM.33.0340	ATPase family associated with various cellular activities (AAA), putative
LmxM.33.0540	Gamma-soluble NSF attachment protein (SNAP-gamma) (N-ethylmaleimide-sensitive factor attachment protein,
LmxM.33.0560	hypothetical protein, conserved
LmxM.33.0590	nuclear cap binding complex subunit CBP110, putative
LmxM.33.0610	enoyl-[acyl-carrier-protein] reductase, putative
LmxM.33.0660	hypothetical protein, unknown function
LmxM.33.0670	ABC transporter-like protein
LmxM.33.0680	Flagellar attachment zone protein 1, putative
LmxM.33.0720	hypothetical protein, conserved
LmxM.33.0815	Transcription factor Pcc1, putative
LmxM.33.0840	translation elongation factor 1-beta, putative
LmxM.33.0860	60S ribosomal protein L13a, putative
LmxM.33.0920	hypothetical protein, conserved
LmxM.33.1000	myosin IB heavy chain, putative
LmxM.33.1050	TPR repeat/SET domain containing protein, putative

LmxM.33.1060	mitochondrial ATP-dependent zinc metallopeptidase, putative
LmxM.33.1110	Shwachman-Bodian-Diamond syndrome (SBDS) protein/SBDS protein C-terminal domain containing protein, put
LmxM.33.1240	3'-5' exonuclease, putative
LmxM.33.1250	NLI-interacting factor, putative
LmxM.33.1330	BCS1 N terminal/ATPase family associated with various cellular activities (AAA), putative
LmxM.33.1360	Nucleoporin NUP225
LmxM.33.1440	hypothetical protein, conserved
LmxM.33.2030	hypothetical protein, conserved
LmxM.33.2050	ATP-dependent RNA helicase, putative
LmxM.33.2090	protein kinase, putative
LmxM.33.2220	Endonuclease/Exonuclease/phosphatase family, putative
LmxM.33.2240	Glycosyl hydrolase family 65 central catalytic domain/Glycosyl hydrolase family 65, C-terminal domain containin
LmxM.33.2360	hypothetical protein, conserved
LmxM.33.2480	Component of motile flagella 8, putative
LmxM.33.2520	Guanine nucleotide exchange factor in Golgi transport N-terminal/Sec7 domain/Domain of unknown function (D
LmxM.33.2650	Domain of unknown function DUF21, putative
LmxM.33.2780	40S ribosomal protein S19 protein, putative
LmxM.33.2790	hypothetical protein, conserved
LmxM.33.2870	ribosomal protein L3, putative
LmxM.33.2900	ribosomal protein L3, putative
LmxM.33.2980	Nucleoside diphosphate kinase 3
LmxM.33.3030	Retinal Maintenance, putative
LmxM.33.3040	hypothetical protein, conserved
LmxM.33.3080	exosome subunit rrp6p homologue, putative
LmxM.33.3100	hypothetical protein, conserved
LmxM.33.3110	AAA domain containing protein, putative
LmxM.33.3230	hypothetical protein, conserved
LmxM.33.3420	metallo-beta-lactamase superfamily, putative
LmxM.33.3430	cleavage and polyadenylation specificity factor, putative
LmxM.33.3460	hypothetical protein, conserved
LmxM.33.3520	peroxisome biosynthesis protein-like protein
LmxM.33.3530	hypothetical protein, conserved
LmxM.33.3570	hypothetical protein, conserved
LmxM.33.3600	adenosine kinase-like protein
LmxM.33.3650	60S ribosomal protein L21, putative
LmxM.33.3710	hypothetical protein, conserved
LmxM.33.3780	phosphomannomutase-like protein
LmxM.33.3860	small nuclear ribonucleoprotein sm d3
LmxM.33.3940	target of rapamycin kinase 3, putative
LmxM.33.3960	HECT-domain (ubiquitin-transferase), putative
LmxM.33.4070	hypothetical protein, conserved
LmxM.33.4080	DnaJ domain containing protein, putative
LmxM.33.4160	dynein heavy chain, putative
LmxM.33.4190	protein serine/threonine phosphatase, putativee
LmxM.33.4200	hypothetical protein, unknown function
LmxM.33.4270	Cullin binding, putative
LmxM.33.4290	nucleolar protein family a, putative
LmxM.33.4530	Phosphatidylinositol 3-kinase tor2
LmxM.33.4580	hypothetical protein, conserved
LmxM.34.0060	hypothetical protein, conserved
LmxM.34.0110	coatomer subunit zeta, putative
LmxM.34.0230	hypothetical protein, conserved
LmxM.34.0300	hypothetical protein, conserved
LmxM.34.0350	hypothetical protein, conserved
LmxM.34.0360	enoyl-CoA hydratase/isomerase family protein, conserved
LmxM.34.0400a	hypothetical protein
LmxM.34.0400b	hypothetical protein
LmxM.34.0610	hypothetical protein, conserved
LmxM.34.0670	hypothetical protein, conserved
LmxM.34.0690	Tetratricopeptide repeat, putative
LmxM.34.0710	Endonuclease/Exonuclease/phosphatase family, putative
LmxM.34.0760	AMP-activated protein kinase, gamma regulatory subunit
LmxM.34.0970	aldose 1-epimerase, putative
LmxM.34.0990	hypothetical protein, conserved

LmxM.34.1090	Nucleolar protein 111, putative
LmxM.34.1110	Rdx family, putative
LmxM.34.1150	oligosaccharyl transferase-like protein
LmxM.34.1200	pre-mRNA splicing factor ATP-dependent RNA helicase, putative
LmxM.34.1210	Degradation arginine-rich protein for mis-folding, putative
LmxM.34.1250	thioredoxin-like protein
LmxM.34.1310	histone H4
LmxM.34.1430	60S ribosomal protein L2, putative
LmxM.34.1440	60S ribosomal protein L2, putative
LmxM.34.1450	hypothetical protein, conserved
LmxM.34.1510	NLI interacting factor-like phosphatase/Zinc finger C-x8-C-x5-C-x3-H type (and similar), putative
LmxM.34.1600	Nucleoporin NUP82
LmxM.34.1620	Concanavalin A-like lectin/glucanases superfamily, putative
LmxM.34.1670	60S ribosomal protein L26, putative
LmxM.34.1720	cyclophilin 14, putative
LmxM.34.1730	casein kinase II, putative
LmxM.34.1740	**DUB6**; ubiquitin hydrolase, putative
LmxM.34.1790	DNA polymerase epsilon subunit B, putative
LmxM.34.1820	Eukaryotic rRNA processing protein EBP2, putative
LmxM.34.1890	60S ribosomal protein L5, putative
LmxM.34.1900	60S ribosomal protein L5, putative
LmxM.34.1920	60S ribosomal protein L36, putative
LmxM.34.1970	ubx domain containing protein, putative
LmxM.34.1980	hypothetical protein, conserved
LmxM.34.1990	U3 small nucleolar RNA-associated protein 6, putative
LmxM.34.2010	40S ribosomal protein S6, putative
LmxM.34.2040	ankyrin repeat protein, putative
LmxM.34.2110	hypothetical protein, conserved
LmxM.34.2120	R-SNARE protein, putative
LmxM.34.2190	60S ribosomal protein L12, putative
LmxM.34.2220	kinetoplastid membrane protein-11
LmxM.34.2221	kinetoplastid membrane protein-11
LmxM.34.2300	hypothetical protein, conserved
LmxM.34.2320	protein kinase, putative
LmxM.34.2330	Importin 1
LmxM.34.2380	hypothetical protein, unknown function
LmxM.34.2550	Double RNA binding domain protein 9
LmxM.34.2725	hypothetical protein, conserved
LmxM.34.2770	hypothetical protein, conserved
LmxM.34.2800	Alpha/beta hydrolase family, putative
LmxM.34.2850	RNA polymerase-associated protein LEO1, putative
LmxM.34.2890	SpoU rRNA Methylase family, putative
LmxM.34.2970	hypothetical protein, conserved
LmxM.34.3160	riboflavin kinase/fmn adenylyltransferase-like protein
LmxM.34.3210	EF-hand domain pair, putative
LmxM.34.3240	hypothetical protein, conserved
LmxM.34.3250	replication factor C, subunit 3, putative
LmxM.34.3260	Peroxin 19
LmxM.34.3290	60S ribosomal subunit protein L31, putative
LmxM.34.3360	Putative death-receptor fusion protein (DUF2428), putative
LmxM.34.3380	poly A polymerase regulatory subunit, putative
LmxM.34.3510	structural maintenance of chromosome (SMC) family protein, putative
LmxM.34.3540	pre-rRNA-processing protein PNO1, putative
LmxM.34.3780	60S ribosomal protein L27A/L29, putative
LmxM.34.3800	60S ribosomal protein L23, putative
LmxM.34.3880	WD domain, G-beta repeat/Sof1-like domain containing protein, putative
LmxM.34.3920	hypothetical protein, conserved
LmxM.34.3940	Chromosome passenger complex (CPC) protein INCENP N terminal, putative
LmxM.34.3950	U5 snRNA-associated splicing factor
LmxM.34.3980	4E-interacting protein, putative
LmxM.34.4000	protein kinase, putative
LmxM.34.4010	protein kinase A catalytic subunit isoform 1
LmxM.34.4030	ATP-dependent RNA helicase, putative
LmxM.34.4070	hypothetical protein, conserved

LmxM.34.4150	hsp70 subfamily B suppressor 1
LmxM.34.4160	Protein of unknown function (DUF2009), putative
LmxM.34.4510	hypothetical protein, conserved
LmxM.34.4550	hypothetical protein, conserved
LmxM.34.4590	phosphatidylserine decarboxylase, putative
LmxM.34.4670	katanin, putative
LmxM.34.4720	glycine cleavage system H protein, putative
LmxM.34.4870	hypothetical protein, conserved
LmxM.34.5070	hypothetical protein, conserved
LmxM.34.5090	hypothetical protein, conserved
LmxM.34.5280	Present in the outer mitochondrial membrane proteome 5
LmxM.34.5310	GTP-binding protein, putative
LmxM.34.5320	SAC3/GANP/Nin1/mts3/eIF-3 p25 family, putative
LmxM.34.5390	HECT-domain (ubiquitin-transferase), putative
LmxM.36.0020	histone H4
LmxM.36.0060	L-ribulokinase, putative
LmxM.36.0080	leucine carboxyl methyltransferase, putative
LmxM.36.0130	PUB domain containing protein, putative
LmxM.36.0210	SET domain protein, putative
LmxM.36.0220	dolichyl-phosphate beta-D-mannosyltransferase precursor, conserved
LmxM.36.0280	hypothetical protein, conserved
LmxM.36.0390	hypothetical protein, conserved
LmxM.36.0460	vacuolar protein sorting-associated protein, putative
LmxM.36.0500	TPR-repeat-containing chaperone protein DNAJ, putative
LmxM.36.0590	Eukaryotic translation initiation factor 4E type 5, putative
LmxM.36.0600	ubiquitin/ribosomal protein S27a, putative
LmxM.36.0710	Protein C21orf2 homolog, putative
LmxM.36.0810	hypothetical protein, conserved
LmxM.36.0870	pre-mRNA splicing factor ATP-dependent RNA helicase, putative
LmxM.36.0940	40S ribosomal protein S18, putative
LmxM.36.1000	intraflagellar transport protein 122, putative
LmxM.36.1030	hypothetical protein, conserved
LmxM.36.1060	ISWI complex protein
LmxM.36.1100	ribosomal protein L24, putative
LmxM.36.1170	hypothetical protein, conserved
LmxM.36.1180	hypothetical protein, conserved
LmxM.36.1240	hypothetical protein, conserved
LmxM.36.1250	40S ribosomal protein S9, putative
LmxM.36.1400	The ARF-like 2 binding protein BART, putative
LmxM.36.1440	N-terminal region of Chorein, a TM vesicle-mediated sorter, putative
LmxM.36.1480	hypothetical protein, conserved
LmxM.36.1500	hypothetical protein, conserved
LmxM.36.1530	protein kinase, putative,serine/threonine protein kinase, putative
LmxM.36.1590	STAG domain containing protein, putative
LmxM.36.1595	hypothetical protein, conserved
LmxM.36.1790	5'-3' exoribonuclease D, putative
LmxM.36.1840	DEAD box RNA helicase, putative
LmxM.36.1870	hypothetical protein, conserved
LmxM.36.1880	CBF/Mak21 family, putative
LmxM.36.1910	trypanin, putative
LmxM.36.1925	60S ribosomal protein L37a
LmxM.36.2000	hypothetical protein, conserved
LmxM.36.2130	ATP-dependent RNA helicase DBP2A, putative
LmxM.36.2150	transmembrane emp24 domain-containing protein
LmxM.36.2170	NOL1/NOP2/sun family, putative
LmxM.36.2470	acid phosphatase, putative
LmxM.36.2500	chromatin assembly factor 1 subunit b-like protein
LmxM.36.2510	Nucleoporin NUP96
LmxM.36.2610	folylpolyglutamate synthetase
LmxM.36.2680	hypothetical protein, conserved
LmxM.36.2710	mitochondrial ATP-dependent zinc metallopeptidase, putative
LmxM.36.2720	transportin2-like protein
LmxM.36.2790	Flagellar Member 1
LmxM.36.2830	pre-mRNA splicing factor ATP-dependent RNA helicase, putative

LmxM.36.2840	Flagellar Member 2
LmxM.36.2870	40S ribosomal protein S24e
LmxM.36.2880	hypothetical protein, conserved
LmxM.36.2980	predicted bromodomain protein
LmxM.36.3060	cullin-like protein
LmxM.36.3120	hypothetical protein, conserved
LmxM.36.3310	actin-like protein, putative
LmxM.36.3360	Bromodomain, putative
LmxM.36.3400	ribosomal protein L29, putative
LmxM.36.3500	hypothetical protein, conserved
LmxM.36.3570	signal recognition particle receptor like protein, putative
LmxM.36.3657	Protein of unknown function (DUF1619), putative
LmxM.36.3690	Nucleoporin NUP119
LmxM.36.3710	hypothetical protein, conserved
LmxM.36.3720	Protein of unknown function (DUF498/DUF598), putative
LmxM.36.3740	60S ribosomal protein L34, putative
LmxM.36.3760	60S ribosomal protein L10a, putative
LmxM.36.3800	aminomethyltransferase, mitochondrial, putative
LmxM.36.3810	aminomethyltransferase, mitochondrial, putative
LmxM.36.3850	Snf7, putative
LmxM.36.4010	hypothetical protein, conserved
LmxM.36.4110	hypothetical protein, conserved
LmxM.36.4210	WD domain, G-beta repeat/Utp21 specific WD40 associated putative domain containing protein, putative
LmxM.36.4270	Nucleoporin NUP152
LmxM.36.4280	hypothetical protein, conserved
LmxM.36.4340	hypothetical protein, unknown function
LmxM.36.4380	alternative oxidase, putative
LmxM.36.4400	ATP-dependent DEAD/H RNA helicase, putative
LmxM.36.4420	60S ribosomal protein L22, putative
LmxM.36.4430	asparaginase-like protein
LmxM.36.4440	hypothetical protein, conserved
LmxM.36.4510	60S ribosomal protein L18, putative
LmxM.36.4520	hypothetical protein, conserved
LmxM.36.4620	hypothetical protein, conserved
LmxM.36.4920	hypothetical protein, conserved
LmxM.36.4960	hypothetical protein, unknown function
LmxM.36.5020	hypothetical protein, conserved
LmxM.36.5030	hypothetical protein, conserved
LmxM.36.5060	Nin one binding (NOB1) Zn-ribbon like, putative
LmxM.36.5080	hypothetical protein, conserved
LmxM.36.5160	pre-mRNA-splicing factor CWC22, putative
LmxM.36.5240	glycosyl hydrolase, putative
LmxM.36.5250	hypothetical protein, conserved
LmxM.36.5260	Adaptor protein complex AP-3 subunit beta-3
LmxM.36.5420	branch point binding protein, putative
LmxM.36.5470	hypothetical protein, conserved
LmxM.36.5670	RNA helicase, putative
LmxM.36.5700	hypothetical protein, conserved
LmxM.36.5780	Gcd10p family, putative
LmxM.36.5800	hypothetical protein, conserved
LmxM.36.5880	small GTPase, putative
LmxM.36.5890	hypothetical protein, conserved
LmxM.36.5930	hypothetical protein, conserved
LmxM.36.5940	A distinct subfamily of CDD/CDA-like deaminases, putative
LmxM.36.5980	hypothetical protein, unknown function
LmxM.36.5990	hook complex protein, conserved
LmxM.36.6050	hypothetical protein, conserved
LmxM.36.6220	flagellum transition zone component, putative
LmxM.36.6320	Phosphatidylinositol 3-kinase tor1
LmxM.36.6330	hypothetical protein, conserved
LmxM.36.6347	hypothetical protein, conserved
LmxM.36.6400	40S ribosomal protein S8, putative
LmxM.36.6430	protein transport protein Sec23-like protein
LmxM.36.6520	hypothetical protein, conserved

LmxM.36.6590	Sperm tail/Sperm tail C-terminal domain containing protein, putative
LmxM.36.6670	cytochrome b5-like Heme/Steroid binding domain containing protein, putative
LmxM.36.6890	Nucleoporin NUP144
LmxM.36.6920	Ribosomal protein L1p/L10e family, putative
LmxM.36.6960	hypothetical protein, conserved

Table S5. Protein kinase genes identified in the CL transcript samples of *L. mexicana* amastigotes

gene ID	protein name	kinase group
LmxM.03.0780	serine/threonine-protein kinase, putative	AGC
LmxM.18.1080	protein kinase A catalytic subunit 3	AGC
LmxM.25.2340	AGC essential kinase 1	AGC
LmxM.28.1670	differentiation inhibitory kinase, putative	AGC
LmxM.29.0800	rac serine-threonine kinase, putative	AGC
LmxM.30.1530	protein kinase, putative	AGC
LmxM.34.3960	protein kinase A catalytic subunit isoform 2	AGC
LmxM.34.4010	protein kinase A catalytic subunit isoform 1	AGC
LmxM.05.0130	protein kinase, putative	CAMK
LmxM.07.0900	serine/threonine kinase, putative	CAMK
LmxM.08_29.2020	5'-AMP-activated protein kinase catalytic subunit alpha, putative	CAMK
LmxM.17.0060	mitogen-activated protein kinase kinase 3, putative	CAMK
LmxM.19.0140	Associated kinase of Tb14-3-3, putative	CAMK
LmxM.19.1470	protein kinase, putative	CAMK
LmxM.22.0810	serine/threonine protein kinase sos2, putative	CAMK
LmxM.22.1170	Protein kinase domain containing protein, putative	CAMK
LmxM.26.2510	protein kinase, putative	CAMK
LmxM.28.2000	phosphorylase kinase-like protein	CAMK
LmxM.32.1710	protein kinase, putative	CAMK
LmxM.34.0490	CAMK/CAMKL family protein kinase, putative	CAMK
LmxM.34.1050	protein kinase, putative	CAMK
LmxM.36.0900	SNF1-related protein kinases, putative (fragment)	CAMK
LmxM.04.1210	casein kinase I, putative	CK1
LmxM.25.1580	casein kinase I, putative	CK1
LmxM.27.1780	casein kinase I-like protein	CK1
LmxM.33.3020	protein kinase, putative	CK1
LmxM.34.1000	casein kinase I, putative	CK1
LmxM.34.1010	casein kinase, putative	CK1
LmxM.08_29.2150	cdc2-related kinase 10, putative	CMGC
LmxM.09.0400	kinetoplastid kinetochore protein 10, putative	CMGC
LmxM.09.0410	kinetoplastid kinetochore protein 19, putative	CMGC
LmxM.10.0200	Mitogen-activated protein kinase 10, putative	CMGC
LmxM.10.0490	mitogen-activated protein kinase	CMGC
LmxM.12.0130	Cell division protein kinase, putative	CMGC
LmxM.14.0830	protein kinase, putative	CMGC
LmxM.14.1070	protein kinase, putative	CMGC
LmxM.15.0180	serine/threonine-protein kinase, putative	CMGC
LmxM.16.0990	protein kinase, putative	CMGC
LmxM.18.0270	protein kinase, putative	CMGC
LmxM.19.0180	mitogen-activated protein kinase 9, putative	CMGC
LmxM.19.0360	protein kinase, putative	CMGC
LmxM.20_36.6470	mitogen-activated protein kinase	CMGC
LmxM.21.1080	cell division protein kinase 2	CMGC
LmxM.21.1650	protein kinase, putative	CMGC
LmxM.22.0490	protein kinase, putative	CMGC
LmxM.24.0670	protein kinase, putative	CMGC
LmxM.25.1560	protein kinase, putative	CMGC
LmxM.26.0980	protein kinase, putative	CMGC
LmxM.27.0100	protein kinase, putative	CMGC
LmxM.27.1800	protein kinase-like protein	CMGC
LmxM.28.0580	Mitogen-activated protein kinase 8, putative	CMGC
LmxM.29.3580	serine/threonine-protein kinase, putative	CMGC
LmxM.31.3250	mitogen-activated protein kinase, putative	CMGC
LmxM.32.1380	mitogen activated protein kinase, putative	CMGC
LmxM.32.1830	dual-specificity protein kinase, putative	CMGC
LmxM.32.2070	mitogen-activated protein kinase, putative	CMGC

LmxM.34.1860	protein kinase-like protein	CMGC
LmxM.34.5010	mitogen-activated protein kinase	CMGC
LmxM.36.0720	mitogen-activated protein kinase 2	CMGC
LmxM.36.4250	serine/threonine kinase, putative	CMGC
LmxM.07.0170	protein kinase, putative	NEK
LmxM.08_29.2570	serine/threonine-protein kinase, putative	NEK
LmxM.08_29.2670	serine/threonine-protein kinase Nek, putative	NEK
LmxM.14.1410	protein kinase, putative	NEK
LmxM.21.0853	Serine/threonine-protein kinase NEK19, putative	NEK
LmxM.21.1565	serine/threonine-protein kinase, putative	NEK
LmxM.22.0950	protein kinase, putative	NEK
LmxM.29.2130	protein kinase, putative	NEK
LmxM.30.2960	Repressor of differentiation kinase 2, putative	NEK
LmxM.02.0570	protein kinase, putative	Others
LmxM.08.0530	protein kinase , putative	Others
LmxM.08.0660	protein kinase, putative	Others
LmxM.08_29.0370	protein kinase-like protein	Others
LmxM.08_29.2490	protein kinase, putative	Others
LmxM.08_29.2720	serine/threonine protein kinase, putative	Others
LmxM.11.0060	protein kinase, putative	Others
LmxM.11.0250	serine/threonine protein kinase, putative	Others
LmxM.11.0510	protein kinase, putative	Others
LmxM.15.0770	protein kinase, putative	Others
LmxM.16.0870	protein kinase, putative	Others
LmxM.19.0590	protein kinase, putative	Others
LmxM.20.0960	protein kinase, putative	Others
LmxM.20.1330	kinase-like protein	Others
LmxM.24.1730	protein kinase, putative	Others
LmxM.25.1520	protein kinase, putative	Others
LmxM.26.2060	Protein kinase domain containing protein, putative	Others
LmxM.26.2110	protein kinase, putative	Others
LmxM.26.2440	protein kinase, putative	Others
LmxM.28.1760	protein kinase, putative	Others
LmxM.29.1560	protein kinase, putative	Others
LmxM.30.2860	protein kinase, putative	Others
LmxM.31.1290	Protein kinase domain containing protein, putative	Others
LmxM.33.0940	Wee1-like protein kinase, putative	Others
LmxM.33.2150	eukaryotic translation initiation factor 2-alpha kinase 2	Others
LmxM.33.2190	kinatase, putative	Others
LmxM.34.1730	casein kinase II, putative	Others
LmxM.34.2320	protein kinase, putative	Others
LmxM.34.2870	protein kinase, putative	Others
LmxM.36.2630	protein kinase, putative	Others
LmxM.36.5350	kinetoplastid kinetochore protein 2, putative	Others
LmxM.04.0440	serine/threonine protein kinase-like protein	STE
LmxM.05.0390	protein kinase, putative	STE
LmxM.07.0250	protein kinase, putative	STE
LmxM.07.0880	protein kinase, putative	STE
LmxM.08.1228	STE group serine/threonine-protein kinase, putative	STE
LmxM.14.1300	protein kinase, putative	STE
LmxM.15.1200	protein kinase, putative	STE
LmxM.17.0390	protein kinase, putative	STE
LmxM.20.0770	protein kinase, putative	STE
LmxM.21.0130	serine/threonine protein kinase, putative	STE
LmxM.24.1450	protein kinase, putative	STE
LmxM.24.2320	mitogen-activated protein kinase	STE
LmxM.26.1730	protein kinase, putative	STE
LmxM.27.1370	protein kinase, putative	STE
LmxM.29.0600	protein kinase, putative	STE
LmxM.29.3050	protein kinase-like protein	STE
LmxM.30.1830	protein kinase-like protein	STE

LmxM.30.1840	hypothetical protein, unknown function	STE
LmxM.31.0120	MEKK-related kinase 1, putative	STE
LmxM.31.0780	protein kinase, putative	STE
LmxM.31.0810	Repressor of differentiation kinase 1, putative	STE
LmxM.32.1400	protein kinase, putative	STE
LmxM.32.2290	protein kinase, putative	STE
LmxM.33.2090	protein kinase, putative	STE
LmxM.34.3170	protein kinase, putative	STE
LmxM.34.4000	protein kinase, putative	STE
LmxM.36.0860	mitogen-activated protein kinase kinase 5	STE
LmxM.36.0910	mitogen-activated protein kinase-like protein	STE
LmxM.36.3680	mitogen activated kinase-like protein	STE
LmxM.08_29.0240	serine/threonine protein kinase RIO1, putative	Unclassified
LmxM.17.0670	protein kinase, putative	Unclassified
LmxM.19.0150	mitogen-activated protein kinase kinase kinase, putative	Unclassified
LmxM.31.0260	Serine/threonine-protein kinase Nek1-related, putative	Unclassified
LmxM.31.1810	protein kinase, putative	Unclassified
LmxM.32.1980	serine/threonine-protein kinase a, putative	Unclassified
LmxM.34.5190	NIMA-related kinase, putative	Unclassified
LmxM.36.1530	protein kinase, putative,serine/threonine protein kinase, putative	Unclassified
LmxM.36.2290	serine/threonine protein kinase, putative	Unclassified

Table S6. Protein kinase genes identified in the CL transcript samples of *L. mexicana* promastigotes

gene ID	protein name	kinase group
LmxM.03.0780	serine/threonine-protein kinase, putative	AGC
LmxM.25.2340	AGC essential kinase 1	AGC
LmxM.29.0800	rac serine-threonine kinase, putative	AGC
LmxM.30.1530	protein kinase, putative	AGC
LmxM.34.3960	protein kinase A catalytic subunit isoform 2	AGC
LmxM.34.4010	protein kinase A catalytic subunit isoform 1	AGC
LmxM.05.0130	protein kinase, putative	CAMK
LmxM.07.0900	serine/threonine kinase, putative	CAMK
LmxM.08_29.2020	5'-AMP-activated protein kinase catalytic subunit alpha, putative	CAMK
LmxM.17.0060	mitogen-activated protein kinase kinase 3, putative	CAMK
LmxM.19.0140	Associated kinase of Tb14-3-3, putative	CAMK
LmxM.19.1470	protein kinase, putative	CAMK
LmxM.22.0810	serine/threonine protein kinase sos2, putative	CAMK
LmxM.28.2000	phosphorylase kinase-like protein	CAMK
LmxM.32.1710	protein kinase, putative	CAMK
LmxM.34.0490	CAMK/CAMKL family protein kinase, putative	CAMK
LmxM.34.1050	protein kinase, putative	CAMK
LmxM.36.0900	SNF1-related protein kinases, putative (fragment)	CAMK
LmxM.04.1210	casein kinase I, putative	CK1
LmxM.25.1580	casein kinase I, putative	CK1
LmxM.27.1780	casein kinase I-like protein	CK1
LmxM.33.3020	protein kinase, putative	CK1
LmxM.34.1010	casein kinase, putative	CK1
LmxM.09.0410	kinetoplastid kinetochore protein 19, putative	CMGC
LmxM.10.0490	mitogen-activated protein kinase	CMGC
LmxM.14.1070	protein kinase, putative	CMGC
LmxM.15.0180	serine/threonine-protein kinase, putative	CMGC
LmxM.16.0990	protein kinase, putative	CMGC
LmxM.18.0270	protein kinase, putative	CMGC
LmxM.19.0180	mitogen-activated protein kinase 9, putative	CMGC
LmxM.21.1080	cell division protein kinase 2	CMGC
LmxM.21.1650	protein kinase, putative	CMGC
LmxM.22.0490	protein kinase, putative	CMGC
LmxM.24.0670	protein kinase, putative	CMGC
LmxM.25.1560	protein kinase, putative	CMGC
LmxM.26.0980	protein kinase, putative	CMGC
LmxM.27.0100	protein kinase, putative	CMGC
LmxM.28.0580	Mitogen-activated protein kinase 8, putative	CMGC
LmxM.29.2910	mitogen-activated protein kinase, putative	CMGC
LmxM.29.3580	serine/threonine-protein kinase, putative	CMGC
LmxM.31.3250	mitogen-activated protein kinase, putative	CMGC
LmxM.32.1380	mitogen activated protein kinase, putative	CMGC
LmxM.32.2070	mitogen-activated protein kinase, putative	CMGC
LmxM.34.5010	mitogen-activated protein kinase	CMGC
LmxM.36.0720	mitogen-activated protein kinase 2	CMGC
LmxM.36.4250	serine/threonine kinase, putative	CMGC
LmxM.07.0170	protein kinase, putative	NEK
LmxM.08_29.2570	serine/threonine-protein kinase, putative	NEK

LmxM.14.1410	protein kinase, putative	NEK
LmxM.21.1565	serine/threonine-protein kinase, putative	NEK
LmxM.22.0950	protein kinase, putative	NEK
LmxM.29.2130	protein kinase, putative	NEK
LmxM.30.2960	Repressor of differentiation kinase 2, putative	NEK
LmxM.02.0570	protein kinase, putative	Others
LmxM.08.0530	protein kinase , putative	Others
LmxM.08_29.2490	protein kinase, putative	Others
LmxM.08_29.2720	serine/threonine protein kinase, putative	Others
LmxM.11.0060	protein kinase, putative	Others
LmxM.11.0250	serine/threonine protein kinase, putative	Others
LmxM.13.0440	mitogen-activated protein kinase kinase 2	Others
LmxM.15.0770	protein kinase, putative	Others
LmxM.17.0790	protein kinase, putative	Others
LmxM.20.0960	protein kinase, putative	Others
LmxM.24.1730	protein kinase, putative	Others
LmxM.25.1520	protein kinase, putative	Others
LmxM.26.2060	Protein kinase domain containing protein, putative	Others
LmxM.26.2110	protein kinase, putative	Others
LmxM.29.1560	protein kinase, putative	Others
LmxM.30.2860	protein kinase, putative	Others
LmxM.31.1290	Protein kinase domain containing protein, putative	Others
LmxM.33.0940	Wee1-like protein kinase, putative	Others
LmxM.33.2150	eukaryotic translation initiation factor 2-alpha kinase 2	Others
LmxM.33.2190	kinatase, putative	Others
LmxM.34.1730	casein kinase II, putative	Others
LmxM.34.2320	protein kinase, putative	Others
LmxM.36.2630	protein kinase, putative	Others
LmxM.36.5350	kinetoplastid kinetochore protein 2, putative	Others
LmxM.04.0440	serine/threonine protein kinase-like protein	STE
LmxM.05.0390	protein kinase, putative	STE
LmxM.07.0250	protein kinase, putative	STE
LmxM.07.0880	protein kinase, putative	STE
LmxM.14.1300	protein kinase, putative	STE
LmxM.15.1200	protein kinase, putative	STE
LmxM.17.0390	protein kinase, putative	STE
LmxM.20.0770	protein kinase, putative	STE
LmxM.21.0130	serine/threonine protein kinase, putative	STE
LmxM.24.2320	mitogen-activated protein kinase	STE
LmxM.27.1370	protein kinase, putative	STE
LmxM.29.3050	protein kinase-like protein	STE
LmxM.30.1830	protein kinase-like protein	STE
LmxM.30.1840	hypothetical protein, unknown function	STE
LmxM.31.0120	MEKK-related kinase 1, putative	STE
LmxM.31.0780	protein kinase, putative	STE
LmxM.31.0810	Repressor of differentiation kinase 1, putative	STE
LmxM.32.1400	protein kinase, putative	STE
LmxM.32.2290	protein kinase, putative	STE
LmxM.33.2090	protein kinase, putative	STE
LmxM.34.3170	protein kinase, putative	STE
LmxM.34.4000	protein kinase, putative	STE
LmxM.36.0910	mitogen-activated protein kinase-like protein	STE

LmxM.36.3680	mitogen activated kinase-like protein	STE
LmxM.10.0830	serine/threonine-protein kinase, putative	Unclassified
LmxM.17.0670	protein kinase, putative	Unclassified
LmxM.19.0150	mitogen-activated protein kinase kinase kinase, putative	Unclassified
LmxM.31.0260	Serine/threonine-protein kinase Nek1-related, putative	Unclassified
LmxM.31.1810	protein kinase, putative	Unclassified
LmxM.34.5190	NIMA-related kinase, putative	Unclassified
LmxM.36.1530	protein kinase, putative,serine/threonine protein kinase, putative	Unclassified
LmxM.36.2290	serine/threonine protein kinase, putative	Unclassified

Table S7. High-confidence tanespimycin affected RBPs of*L.mexicana* promastigote.

[-Log(P-value)]	Log2 FC	Protein IDs	Fasta headers	Unique peptides
6.269377902	4.16349	E9ATF4	tr|E9ATF4|E9ATF4_LEIMU Uncharacterized protein OS=Leishmania mexicana (s	2
5.086476262	3.11531	E9AK31	tr|E9AK31|E9AK31_LEIMU Uncharacterized protein OS=Leishmania mexicana (	3
5.347419469	2.92413	E9B6K7	tr|E9B6K7|E9B6K7_LEIMU Putative 60S ribosomal subunit protein L31 OS=Leish	5
5.486657911	2.72793	E9B012	tr|E9B012|E9B012_LEIMU Putative zinc transporter OS=Leishmania mexicana (	3
5.307605285	2.58173	E9B5P8	tr|E9B5P8|E9B5P8_LEIMU Uncharacterized protein OS=Leishmania mexicana (s	3
4.913396542	2.41913	E9AMA8	tr|E9AMA8|E9AMA8_LEIMU Superoxide dismutase OS=Leishmania mexicana (s	2
3.443121275	2.28862	E9AZ65	tr|E9AZ65|E9AZ65_LEIMU Eukaryotic translation initiation factor eIF-4E,putativ	2
4.824254384	2.22282	E9B106	tr|E9B106|E9B106_LEIMU Uncharacterized protein OS=Leishmania mexicana (s	2
4.804908894	2.21383	E9B4C7	tr|E9B4C7|E9B4C7_LEIMU Uncharacterized protein OS=Leishmania mexicana (s	6
4.806453312	2.20749	E9B645;E9A	tr|E9B645|E9B645_LEIMU Putative 60S ribosomal protein L26 OS=Leishmania r	2
4.812039485	2.17532	E9B0Z8	tr|E9B0Z8|E9B0Z8_LEIMU Conserved zinc-finger protein OS=Leishmania mexica	3
4.468215109	2.04841	E9ATL7	tr|E9ATL7|E9ATL7_LEIMU Uncharacterized protein OS=Leishmania mexicana (s	2
4.581239834	1.95494	E9AS66	tr|E9AS66|E9AS66_LEIMU Uncharacterized protein OS=Leishmania mexicana (s	3
4.905267512	1.95232	E9AM47	tr|E9AM47|E9AM47_LEIMU Uncharacterized protein OS=Leishmania mexicana	5
5.012648766	1.94218	E9AVK9	tr|E9AVK9|E9AVK9_LEIMU Uncharacterized protein OS=Leishmania mexicana (	5
4.361048879	1.93489	E9AKC8;E9A	tr|E9AKC8|E9AKC8_LEIMU Putative viscerotropic leishmaniasis antigen OS=Leis	2
5.158093474	1.89672	E9AV01	tr|E9AV01|E9AV01_LEIMU Mitochondrial processing peptidase alpha subunit,p	3
4.276434901	1.87502	E9ALD3	tr|E9ALD3|E9ALD3_LEIMU Peptide methionine sulfoxide reductase-like (Putativ	2
4.938531141	1.85994	E9AQ10	tr|E9AQ10|E9AQ10_LEIMU Putative ATP-dependent RNA helicase OS=Leishmar	2
4.517971563	1.81621	E9B474;E9B	tr|E9B474|E9B474_LEIMU Uncharacterized protein OS=Leishmania mexicana (s	3
4.37173809	1.79994	E9AQ75	tr|E9AQ75|E9AQ75_LEIMU Uncharacterized protein OS=Leishmania mexicana (	4
4.091768367	1.78349	E9AYC5	tr|E9AYC5|E9AYC5_LEIMU Uncharacterized protein OS=Leishmania mexicana (s	2
4.145741093	1.78148	E9B442	tr|E9B442|E9B442_LEIMU Uncharacterized protein OS=Leishmania mexicana (s	2
4.112647259	1.76748	E9AZK8	tr|E9AZK8|E9AZK8_LEIMU Carbonic anhydrase-like protein OS=Leishmania mex	6
4.247851286	1.74648	E9AN61	tr|E9AN61|E9AN61_LEIMU Glycerol-3-phosphate dehydrogenase [NAD(+)] OS=	3
4.367679012	1.72921	E9B1P8	tr|E9B1P8|E9B1P8_LEIMU Uncharacterized protein OS=Leishmania mexicana (s	5
4.204381205	1.71561	E9AK86	tr|E9AK86|E9AK86_LEIMU Acyltransferase-like protein, copy 2 OS=Leishmania	3
4.200840649	1.67738	E9AMQ3	tr|E9AMQ3|E9AMQ3_LEIMU Uncharacterized protein OS=Leishmania mexicana	5
4.133498272	1.66044	E9AS63	tr|E9AS63|E9AS63_LEIMU Proteasome regulatory non-ATP-ase subunit,putativ	4
5.15633423	1.66	E9AT16;E9A	tr|E9AT16|E9AT16_LEIMU Methyltransferase OS=Leishmania mexicana (strain	3
4.288908238	1.65236	E9B2K9	tr|E9B2K9|E9B2K9_LEIMU Uncharacterized protein OS=Leishmania mexicana (s	2
4.60083345	1.63465	E9AU20	tr|E9AU20|E9AU20_LEIMU Uncharacterized protein OS=Leishmania mexicana (	12
4.178451273	1.62976	E9AZ12	tr|E9AZ12|E9AZ12_LEIMU Uncharacterized protein OS=Leishmania mexicana (s	4
5.098073823	1.60241	E9AP69	tr|E9AP69|E9AP69_LEIMU Uncharacterized protein OS=Leishmania mexicana (s	5
4.219316521	1.56252	E9AK64	tr|E9AK64|E9AK64_LEIMU Pescadillo homolog OS=Leishmania mexicana (strain	2
4.259475238	1.55658	E9AJC9	tr|E9AJC9|E9AJC9_LEIMU 40S ribosomal protein S7 OS=Leishmania mexicana (s	10
3.953165767	1.54311	E9AMC1	tr|E9AMC1|E9AMC1_LEIMU Uncharacterized protein OS=Leishmania mexicana	7
3.981897843	1.53032	E9AT47	tr|E9AT47|E9AT47_LEIMU Uncharacterized protein OS=Leishmania mexicana (s	2
4.892544892	1.50434	E9AZP7	tr|E9AZP7|E9AZP7_LEIMU Putative RNA binding protein rbp16 OS=Leishmania	5
4.011215195	1.46157	E9AK29	tr|E9AK29|E9AK29_LEIMU 60S ribosomal protein L11 (L5, L16) OS=Leishmania r	6
4.223026912	1.46005	E9B049	tr|E9B049|E9B049_LEIMU DNA topoisomerase 2 OS=Leishmania mexicana (stra	8
4.499295107	1.42463	E9AZA1	tr|E9AZA1|E9AZA1_LEIMU Small nuclear ribonucleoprotein protein,putative OS	2
4.930981417	1.41324	Q25327;E9A	tr|Q25327|Q25327_LEIME Membrane-bound acid phosphatase OS=Leishmania	7
2.901080864	1.38597	E9AQU5	tr|E9AQU5|E9AQU5_LEIMU Uncharacterized protein OS=Leishmania mexicana	2
3.935209397	1.36663	E9AUX6	tr|E9AUX6|E9AUX6_LEIMU Uncharacterized protein OS=Leishmania mexicana (	2
3.847628902	1.35868	E9B1D2	tr|E9B1D2|E9B1D2_LEIMU Putative serine/threonine-protein kinase OS=Leishn	11
3.967698186	1.30052	E9AK54	tr|E9AK54|E9AK54_LEIMU Uncharacterized protein OS=Leishmania mexicana (s	2
4.13091393	1.28954	Q868H1;E9A	tr|Q868H1|Q868H1_LEIME Inhibitor of cysteine peptidases OS=Leishmania me	2
4.726094181	1.26993	E9AZJ9	tr|E9AZJ9|E9AZJ9_LEIMU Uncharacterized protein OS=Leishmania mexicana (st	3
3.488549237	1.26748	E9AP04;E9A	tr|E9AP04|E9AP04_LEIMU Promastigote surface antigen protein PSA (Fragmen	3
4.532926193	1.25925	E9ALB7	tr|E9ALB7|E9ALB7_LEIMU RNA binding protein-like protein OS=Leishmania me	7
3.987140275	1.21935	E9AM79	tr|E9AM79|E9AM79_LEIMU Structural maintenance of chromosomes protein C	3
4.150885739	1.20836	E9B549	tr|E9B549|E9B549_LEIMU Uncharacterized protein OS=Leishmania mexicana (s	5
4.030204919	1.15628	E9ALA4	tr|E9ALA4|E9ALA4_LEIMU Splicing factor ptsr1-like protein OS=Leishmania mex	15
2.793604677	1.15196	E9AUY6	tr|E9AUY6|E9AUY6_LEIMU Uncharacterized protein OS=Leishmania mexicana (	3
3.059888365	1.10737	E9B3C4	tr|E9B3C4|E9B3C4_LEIMU Uncharacterized protein OS=Leishmania mexicana (s	2
4.040616675	1.10131	E9AXM2	tr|E9AXM2|E9AXM2_LEIMU Uncharacterized protein OS=Leishmania mexicana	8
3.511246316	1.09908	E9AUU6	tr|E9AUU6|E9AUU6_LEIMU Putative 40S ribosomal protein S11 OS=Leishmania	5
3.565578811	1.07431	E9B3Z3	tr|E9B3Z3|E9B3Z3_LEIMU Ribulose-phosphate 3-epimerase OS=Leishmania me	2

4.292668861	1.07394	E9AKA9	tr|E9AKA9|E9AKA9_LEIMU Uncharacterized protein OS=Leishmania mexicana (	2
3.719297717	1.06799	E9AZN4	tr|E9AZN4|E9AZN4_LEIMU Uncharacterized protein OS=Leishmania mexicana (	3
3.379330456	1.05918	E9AL41	tr|E9AL41|E9AL41_LEIMU Uncharacterized protein OS=Leishmania mexicana (s	2
2.640719312	1.03507	E9AVM5	tr|E9AVM5|E9AVM5_LEIMU Uncharacterized protein OS=Leishmania mexicana	2
3.510208645	1.01183	E9AZB6	tr|E9AZB6|E9AZB6_LEIMU Putative RNA-binding protein OS=Leishmania mexica	3
3.249433526	1.01157	E9AV59	tr|E9AV59|E9AV59_LEIMU Xanthine phosphoribosyltransferase OS=Leishmania	4
3.940347401	0.99981	E9APZ2	tr|E9APZ2|E9APZ2_LEIMU Uncharacterized protein OS=Leishmania mexicana (s	24
3.686366429	0.9767	E9ATF6;E9A	tr|E9ATF6|E9ATF6_LEIMU Putative 60S ribosomal protein L34 OS=Leishmania r	3
2.893901962	0.96295	E9B4I3	tr|E9B4I3|E9B4I3_LEIMU Malate dehydrogenase OS=Leishmania mexicana (stra	2
3.518426224	0.95839	E9AJR6	tr|E9AJR6|E9AJR6_LEIMU Uncharacterized protein OS=Leishmania mexicana (s	2
3.224914686	0.95651	E9AQK4	tr|E9AQK4|E9AQK4_LEIMU Putative 60S ribosomal protein L21 OS=Leishmania	3
3.356097985	0.95299	E9AXC4	tr|E9AXC4|E9AXC4_LEIMU Uncharacterized protein OS=Leishmania mexicana (	3
3.688099222	0.95056	E9ASI0;E9AI	tr|E9ASI0|E9ASI0_LEIMU Putative ubiquitin/ribosomal protein S27a OS=Leishm	2
3.794331377	0.91709	E9AQ77	tr|E9AQ77|E9AQ77_LEIMU Uncharacterized protein OS=Leishmania mexicana (	9
2.78818461	0.91164	E9AMG5	tr|E9AMG5|E9AMG5_LEIMU Uncharacterized protein OS=Leishmania mexicana	2
4.072148806	0.89884	E9AXV0	tr|E9AXV0|E9AXV0_LEIMU Uncharacterized protein OS=Leishmania mexicana (	2
3.362790816	0.89725	E9B2E2	tr|E9B2E2|E9B2E2_LEIMU Uncharacterized protein OS=Leishmania mexicana (s	16
3.802032866	0.8943	E9AV16	tr|E9AV16|E9AV16_LEIMU Putative DnaJ protein OS=Leishmania mexicana (stra	3
3.656337928	0.89083	E9AQX9	tr|E9AQX9|E9AQX9_LEIMU Uncharacterized protein OS=Leishmania mexicana (	8
4.166036983	0.87441	E9APR7	tr|E9APR7|E9APR7_LEIMU Elongation of fatty acids protein OS=Leishmania me	2
3.157725141	0.8431	E9B1F3	tr|E9B1F3|E9B1F3_LEIMU Peptidyl-prolyl cis-trans isomerase OS=Leishmania m	3
2.613059015	0.84236	E9AZ16	tr|E9AZ16|E9AZ16_LEIMU Putative intraflagellar transport protein IFT88 OS=Le	2
3.45791519	0.83926	E9AL69	tr|E9AL69|E9AL69_LEIMU Uncharacterized protein OS=Leishmania mexicana (s	5
3.724814654	0.81805	E9ALP4	tr|E9ALP4|E9ALP4_LEIMU Putative 40S ribosomal protein S15A OS=Leishmania	5
3.10922099	0.81061	E9ARS0	tr|E9ARS0|E9ARS0_LEIMU Uncharacterized protein OS=Leishmania mexicana (s	2
3.170483291	0.80365	E9AWQ4	tr|E9AWQ4|E9AWQ4_LEIMU Kinesin-like protein OS=Leishmania mexicana (str	5
3.456671752	0.80038	E9B700	tr|E9B700|E9B700_LEIMU Uncharacterized protein OS=Leishmania mexicana (s	12
2.799089751	0.79977	E9AST5	tr|E9AST5|E9AST5_LEIMU Putative poly-zinc finger protein 2 OS=Leishmania m	3
3.462344693	0.79974	E9ARK0	tr|E9ARK0|E9ARK0_LEIMU Putative 60S ribosomal protein L10a OS=Leishmania	5
2.878113295	0.79525	E9AUV1	tr|E9AUV1|E9AUV1_LEIMU Uncharacterized protein OS=Leishmania mexicana	2
4.057967725	0.78045	E9B2N4	tr|E9B2N4|E9B2N4_LEIMU Uncharacterized protein OS=Leishmania mexicana (	3
3.069723532	0.77644	E9B5U1;E9E	tr|E9B5U1|E9B5U1_LEIMU 60S ribosomal protein L18a OS=Leishmania mexicar	8
3.328950608	0.77068	E9AT85	tr|E9AT85|E9AT85_LEIMU Fibrillarin OS=Leishmania mexicana (strain MHOM/(	3
2.540665338	0.76531	E9AS22	tr|E9AS22|E9AS22_LEIMU Putative kinesin OS=Leishmania mexicana (strain MH	5
2.645619061	0.75935	E9B5D6	tr|E9B5D6|E9B5D6_LEIMU Uncharacterized protein OS=Leishmania mexicana (	2
2.609530632	0.75693	E9B108	tr|E9B108|E9B108_LEIMU Sphingosine phosphate lyase-like protein,putative O	2
3.258382882	0.75548	E9AVK4	tr|E9AVK4|E9AVK4_LEIMU Uncharacterized protein OS=Leishmania mexicana (	17
3.413022291	0.75522	Q2HWD2;E9	tr|Q2HWD2|Q2HWD2_LEIAM Putative lanosterol C-14 demethylase OS=Leishm	5
3.046044032	0.75172	E9B0H7	tr|E9B0H7|E9B0H7_LEIMU Uncharacterized protein OS=Leishmania mexicana (	2
3.28266902	0.74281	E9ATU4	tr|E9ATU4|E9ATU4_LEIMU Uncharacterized protein OS=Leishmania mexicana (	29
3.162984694	0.74127	E9AQ29	tr|E9AQ29|E9AQ29_LEIMU Ribonucleoprotein p18, mitochondrial,putative OS=	9
3.046939971	0.73378	E9B0Q0	tr|E9B0Q0|E9B0Q0_LEIMU Small nuclear ribonucleoprotein E OS=Leishmania n	2
3.268650023	0.72749	E9ALF6	tr|E9ALF6|E9ALF6_LEIMU Uncharacterized protein OS=Leishmania mexicana (s	5
3.412160142	0.72057	E9B0E2	tr|E9B0E2|E9B0E2_LEIMU Putative 2-hydroxy-3-oxopropionate reductase OS=L	2
3.353617383	0.70951	E9AUF1;E9A	tr|E9AUF1|E9AUF1_LEIMU Phosphoglycerate kinase OS=Leishmania mexicana (	7
2.520221388	0.69684	E9AT28	tr|E9AT28|E9AT28_LEIMU Nuclear pore protein OS=Leishmania mexicana (stra	5
3.513413302	0.68217	E9AVG7;Q8	tr|E9AVG7|E9AVG7_LEIMU Putative cytochrome c oxidase subunit VI OS=Leish	3
2.713981613	0.65756	E9AZR2	tr|E9AZR2|E9AZR2_LEIMU 40S ribosomal protein S14 OS=Leishmania mexicana	5
2.823822769	0.65004	E9B6W8	tr|E9B6W8|E9B6W8_LEIMU Uncharacterized protein OS=Leishmania mexicana	3
1.94106109	0.63407	E9B1B7	tr|E9B1B7|E9B1B7_LEIMU Uncharacterized protein OS=Leishmania mexicana (s	2
3.776297554	0.62999	E9B6L6	tr|E9B6L6|E9B6L6_LEIMU Uncharacterized protein OS=Leishmania mexicana (s	4
3.007735651	0.62289	E9AVQ7	tr|E9AVQ7|E9AVQ7_LEIMU Uncharacterized protein OS=Leishmania mexicana	12
2.952642165	0.60534	E9AU73	tr|E9AU73|E9AU73_LEIMU Uncharacterized protein OS=Leishmania mexicana (	3
2.940718562	0.60437	E9AQJ6;O76	tr|E9AQJ6|E9AQJ6_LEIMU Uncharacterized protein OS=Leishmania mexicana (s	4
2.382173999	0.60209	E9AR29	tr|E9AR29|E9AR29_LEIMU Putative P-type ATPase OS=Leishmania mexicana (st	3
2.800593604	0.59344	E9AX53	tr|E9AX53|E9AX53_LEIMU 40S ribosomal protein S8 OS=Leishmania mexicana (	6
2.405492005	0.59197	E9ALU6	tr|E9ALU6|E9ALU6_LEIMU Putative ATP-dependent Clp protease subunit, heat	4
2.856950684	0.58674	E9ANT3	tr|E9ANT3|E9ANT3_LEIMU Uncharacterized protein OS=Leishmania mexicana (	3
2.312097592	0.58452	E9AXK9	tr|E9AXK9|E9AXK9_LEIMU Uncharacterized protein OS=Leishmania mexicana (s	2
2.9152792	0.58149	E9APB2	tr|E9APB2|E9APB2_LEIMU Uncharacterized protein OS=Leishmania mexicana (	10
3.498301767	0.57394	E9APL2	tr|E9APL2|E9APL2_LEIMU Uncharacterized protein OS=Leishmania mexicana (s	2
2.065682747	0.56674	E9APA9	tr|E9APA9|E9APA9_LEIMU Uncharacterized protein OS=Leishmania mexicana (	2
2.559106034	0.56104	E9APW0	tr|E9APW0|E9APW0_LEIMU Uncharacterized protein OS=Leishmania mexicana	2

2.930322153	0.55911	E9AMW3	tr|E9AMW3|E9AMW3_LEIMU Mitochondrial RNA binding protein 2 OS=Leishm	4
2.602595653	0.55527	E9ATS5	tr|E9ATS5|E9ATS5_LEIMU Uncharacterized protein OS=Leishmania mexicana (s	5
2.868497063	0.55409	E9AWS6;A0	tr|E9AWS6|E9AWS6_LEIMU Triosephosphate isomerase OS=Leishmania mexica	7
2.987436555	0.55143	E9ALH0	tr|E9ALH0|E9ALH0_LEIMU Uncharacterized protein OS=Leishmania mexicana (:	4
2.431254837	0.54769	E9B6D4	tr|E9B6D4|E9B6D4_LEIMU Putative RNA binding protein OS=Leishmania mexic	3
2.259045065	0.53818	E9B2R8	tr|E9B2R8|E9B2R8_LEIMU Uncharacterized protein OS=Leishmania mexicana (s	2
1.756381233	0.53761	E9B2F4	tr|E9B2F4|E9B2F4_LEIMU Uncharacterized protein OS=Leishmania mexicana (s	2
2.832241589	0.53485	E9B4F8	tr|E9B4F8|E9B4F8_LEIMU Small nuclear ribonucleoprotein Sm D2 OS=Leishmar	3
2.205888504	0.53365	E9B3T0	tr|E9B3T0|E9B3T0_LEIMU Uncharacterized protein OS=Leishmania mexicana (s	3
3.263199683	0.53087	E9B722	tr|E9B722|E9B722_LEIMU Uncharacterized protein OS=Leishmania mexicana (s	2
2.463317089	0.52989	E9ARU5	tr|E9ARU5|E9ARU5_LEIMU Uncharacterized protein OS=Leishmania mexicana (	9
2.512927682	0.50352	E9AJH4	tr|E9AJH4|E9AJH4_LEIMU Uncharacterized protein OS=Leishmania mexicana (s	2
1.60749384	0.49705	E9AQ28	tr|E9AQ28|E9AQ28_LEIMU Putative replication Factor A 28 kDa subunit OS=Lei	2
1.743773355	0.47907	E9B625	tr|E9B625|E9B625_LEIMU Uncharacterized protein OS=Leishmania mexicana (s	2
2.361613014	0.47611	E9AXC6;E9A	tr|E9AXC6|E9AXC6_LEIMU RNA-binding protein, putative, UPB1 OS=Leishmania	5
2.400272983	0.47593	E9AZ66	tr|E9AZ66|E9AZ66_LEIMU Uncharacterized protein OS=Leishmania mexicana (s	3
3.193530547	0.47281	E9B1Q2	tr|E9B1Q2|E9B1Q2_LEIMU Uncharacterized protein OS=Leishmania mexicana (	4
2.270558691	0.4691	E9B163	tr|E9B163|E9B163_LEIMU Putative aldehyde dehydrogenase OS=Leishmania m	3
2.341868859	0.46601	E9APY5	tr|E9APY5|E9APY5_LEIMU Putative tyrosyl-tRNA synthetase OS=Leishmania me	5
2.478789732	0.46224	E9AXG9	tr|E9AXG9|E9AXG9_LEIMU Peptidyl-prolyl cis-trans isomerase OS=Leishmania r	5
2.596574853	0.4592	E9B0F9	tr|E9B0F9|E9B0F9_LEIMU Kinesin-like protein OS=Leishmania mexicana (strain	2
3.114347309	0.45712	E9ALI8	tr|E9ALI8|E9ALI8_LEIMU Putative aspartyl aminopeptidase OS=Leishmania mex	2
2.242210726	0.43472	E9AWR8	tr|E9AWR8|E9AWR8_LEIMU Putative malic enzyme OS=Leishmania mexicana (:	6
1.716171006	0.43039	E9AUF8	tr|E9AUF8|E9AUF8_LEIMU Uncharacterized protein OS=Leishmania mexicana (	2
2.710064928	0.42976	E9AYZ5	tr|E9AYZ5|E9AYZ5_LEIMU Putative isovaleryl-coA dehydrogenase OS=Leishmar	4
2.380253227	0.41861	E9B3L2;P27	tr|E9B3L2|E9B3L2_LEIMU Heat shock protein 83-1 OS=Leishmania mexicana (st	32
2.652407542	0.41754	E9ANJ3	tr|E9ANJ3|E9ANJ3_LEIMU Putative pumilio-repeat, RNA-binding protein OS=Le	18
3.156718763	0.41663	E9ATD6	tr|E9ATD6|E9ATD6_LEIMU Putative signal recognition particle receptor like prc	3
3.109852808	0.41585	E9B3P3	tr|E9B3P3|E9B3P3_LEIMU Uncharacterized protein OS=Leishmania mexicana (s	2
2.221903929	0.40961	E9AMV3	tr|E9AMV3|E9AMV3_LEIMU Uncharacterized protein OS=Leishmania mexicana	10
2.506200526	0.40208	E9AN04	tr|E9AN04|E9AN04_LEIMU Uncharacterized protein OS=Leishmania mexicana (	8
2.416682141	0.40176	E9AJT5	tr|E9AJT5|E9AJT5_LEIMU Uncharacterized protein OS=Leishmania mexicana (st	7
2.944059342	-0.39995	E9AX62	tr|E9AX62|E9AX62_LEIMU Uncharacterized protein OS=Leishmania mexicana (:	2
2.715142401	-0.41008	E9B2F6	tr|E9B2F6|E9B2F6_LEIMU Uncharacterized protein OS=Leishmania mexicana (s	5
2.348135586	-0.41372	E9B2Z3	tr|E9B2Z3|E9B2Z3_LEIMU Uncharacterized protein OS=Leishmania mexicana (s	2
2.169656095	-0.41677	E9ATG6	tr|E9ATG6|E9ATG6_LEIMU Putative glycyl tRNA synthetase OS=Leishmania me:	6
2.938605169	-0.41729	E9B5Y6	tr|E9B5Y6|E9B5Y6_LEIMU Uncharacterized protein OS=Leishmania mexicana (s	2
2.798740005	-0.41861	E9AV21	tr|E9AV21|E9AV21_LEIMU Putative la RNA binding protein OS=Leishmania mex	4
2.522018052	-0.41991	E9ASU7	tr|E9ASU7|E9ASU7_LEIMU Uncharacterized protein OS=Leishmania mexicana (	4
2.110839835	-0.4222	Q2TUW2;E9	tr|Q2TUW2|Q2TUW2_LEIAM Inosine-5-monophosphate dehydrogenase OS=Le	5
2.587826834	-0.42351	E9AQK9	tr|E9AQK9|E9AQK9_LEIMU Uncharacterized protein OS=Leishmania mexicana (	2
2.138802252	-0.42359	E9AWF1	tr|E9AWF1|E9AWF1_LEIMU Putative dynein heavy chain OS=Leishmania mexic	11
2.044855536	-0.42535	E9AXD1	tr|E9AXD1|E9AXD1_LEIMU Uncharacterized protein OS=Leishmania mexicana (	6
1.89832921	-0.42636	E9B131	tr|E9B131|E9B131_LEIMU Putative RNA-binding protein OS=Leishmania mexica	3
2.226065786	-0.42714	E9AK89	tr|E9AK89|E9AK89_LEIMU Uncharacterized protein OS=Leishmania mexicana (:	5
2.162732686	-0.42755	E9APD0	tr|E9APD0|E9APD0_LEIMU NADH-cytochrome b5 reductase OS=Leishmania me	4
2.3582175	-0.4282	E9B4N8	tr|E9B4N8|E9B4N8_LEIMU Uncharacterized protein OS=Leishmania mexicana (	8
2.000951245	-0.42873	E9AT99	tr|E9AT99|E9AT99_LEIMU 14-3-3 protein-like protein OS=Leishmania mexicana	10
2.341549185	-0.4294	E9AS67	tr|E9AS67|E9AS67_LEIMU Uncharacterized protein OS=Leishmania mexicana (s	11
2.370266513	-0.42946	E9B4N7	tr|E9B4N7|E9B4N7_LEIMU Uncharacterized protein OS=Leishmania mexicana (	4
2.415474044	-0.42975	E9AK75	tr|E9AK75|E9AK75_LEIMU Uncharacterized protein OS=Leishmania mexicana (:	4
1.809899169	-0.4305	E9AZ71	tr|E9AZ71|E9AZ71_LEIMU Uncharacterized protein OS=Leishmania mexicana (s	7
1.863805736	-0.4311	E9AX98	tr|E9AX98|E9AX98_LEIMU Uncharacterized protein OS=Leishmania mexicana (:	2
2.416320056	-0.43142	E8NHE3	tr|E8NHE3|E8NHE3_LEIMU Putative 60S ribosomal protein L28 OS=Leishmania	3
2.498456242	-0.43344	E9B2F9	tr|E9B2F9|E9B2F9_LEIMU Uncharacterized protein OS=Leishmania mexicana (s	2
2.674206101	-0.43461	E9AMX3	tr|E9AMX3|E9AMX3_LEIMU Putative AAA family ATPase OS=Leishmania mexica	6
2.184461475	-0.43514	E9AY79	tr|E9AY79|E9AY79_LEIMU Uncharacterized protein OS=Leishmania mexicana (:	2
2.432496484	-0.43537	E9AP62;E8N	tr|E9AP62|E9AP62_LEIMU Tubulin alpha chain OS=Leishmania mexicana (strair	20
2.730459874	-0.43571	E9B2G6;E9A	tr|E9B2G6|E9B2G6_LEIMU 40S ribosomal protein S2 OS=Leishmania mexicana	9
2.00618192	-0.43686	E9B632	tr|E9B632|E9B632_LEIMU Putative reiske iron-sulfur protein OS=Leishmania m	4
2.540285148	-0.43823	E9ATW0	tr|E9ATW0|E9ATW0_LEIMU Putative adaptin OS=Leishmania mexicana (strain	3
2.478860038	-0.43912	E9AK98	tr|E9AK98|E9AK98_LEIMU Putative RNA-binding protein OS=Leishmania mexica	4
1.446385011	-0.43916	E9B4E9	tr|E9B4E9|E9B4E9_LEIMU Uncharacterized protein OS=Leishmania mexicana (s	6

2.548552058	-0.43992	E9AVS9	tr|E9AVS9|E9AVS9_LEIMU Uncharacterized protein OS=Leishmania mexicana (s	2
1.872904505	-0.44188	E9ATZ6	tr|E9ATZ6|E9ATZ6_LEIMU Putative isoleucyl-tRNA synthetase OS=Leishmania n	11
2.473755245	-0.44277	E9AMI7	tr|E9AMI7|E9AMI7_LEIMU Uncharacterized protein OS=Leishmania mexicana (	2
2.258234066	-0.44404	E9AQW8	tr|E9AQW8|E9AQW8_LEIMU Uncharacterized protein OS=Leishmania mexicana	4
1.822875193	-0.44476	E9AT24	tr|E9AT24|E9AT24_LEIMU Putative acid phosphatase OS=Leishmania mexicana	2
3.068062469	-0.44492	E9AXP9	tr|E9AXP9|E9AXP9_LEIMU Uncharacterized protein OS=Leishmania mexicana (s	2
1.960700261	-0.4457	E9AMC2	tr|E9AMC2|E9AMC2_LEIMU Uncharacterized protein OS=Leishmania mexicana	5
1.95809157	-0.44746	E9AVJ1	tr|E9AVJ1|E9AVJ1_LEIMU Transcription factor-like protein OS=Leishmania mex	5
1.918115285	-0.44876	E9AYU0	tr|E9AYU0|E9AYU0_LEIMU Putative nucleoporin OS=Leishmania mexicana (stra	7
1.431059433	-0.45057	E9AM81	tr|E9AM81|E9AM81_LEIMU Putative Qc-SNARE protein OS=Leishmania mexica	2
2.561275006	-0.45196	E9ANB3	tr|E9ANB3|E9ANB3_LEIMU RNA-binding protein-like protein OS=Leishmania m	5
2.123226484	-0.45244	E9B0P4	tr|E9B0P4|E9B0P4_LEIMU Uncharacterized protein OS=Leishmania mexicana (s	3
2.499148408	-0.45363	E9ALE1	tr|E9ALE1|E9ALE1_LEIMU Uncharacterized protein OS=Leishmania mexicana (s	2
2.244352858	-0.4547	E9AUK8	tr|E9AUK8|E9AUK8_LEIMU rRNA biogenesis protein-like protein OS=Leishmania	8
2.749649317	-0.45676	E9ALY6;Q9U	tr|E9ALY6|E9ALY6_LEIMU Putative ADP ribosylation factor 3 OS=Leishmania me	7
2.110646476	-0.45855	E9AZH5	tr|E9AZH5|E9AZH5_LEIMU Putative pantothenate kinase subunit OS=Leishman	5
2.158865366	-0.4622	E9ANQ5	tr|E9ANQ5|E9ANQ5_LEIMU Uncharacterized protein OS=Leishmania mexicana	4
2.0630423	-0.46327	E9AZX7	tr|E9AZX7|E9AZX7_LEIMU Putative hydrolase, alpha/beta fold family OS=Leishn	3
1.563848706	-0.46766	E9APV3	tr|E9APV3|E9APV3_LEIMU Uncharacterized protein OS=Leishmania mexicana (	3
2.16689145	-0.46772	E9AN41	tr|E9AN41|E9AN41_LEIMU Protein transport protein SEC23 OS=Leishmania me	5
1.770508807	-0.47084	E9B5N2	tr|E9B5N2|E9B5N2_LEIMU Cell differentiation protein-like protein OS=Leishma	3
1.989071222	-0.47452	E9AZ67	tr|E9AZ67|E9AZ67_LEIMU Uncharacterized protein OS=Leishmania mexicana (s	5
1.966328092	-0.47455	E9AZ92	tr|E9AZ92|E9AZ92_LEIMU Uncharacterized protein OS=Leishmania mexicana (s	2
2.230157587	-0.47504	E9AJY3;O09	tr|E9AJY3|E9AJY3_LEIMU Calcium-transporting ATPase OS=Leishmania mexican	9
2.601126246	-0.47576	E9AYN0	tr|E9AYN0|E9AYN0_LEIMU Uncharacterized protein OS=Leishmania mexicana (	4
1.600357532	-0.47597	E9AXI5	tr|E9AXI5|E9AXI5_LEIMU Uncharacterized protein OS=Leishmania mexicana (st	3
2.418961436	-0.47626	E9AMC6	tr|E9AMC6|E9AMC6_LEIMU Putative small ubiquitin protein OS=Leishmania me	2
1.949090979	-0.47668	E9ARJ3	tr|E9ARJ3|E9ARJ3_LEIMU V-type proton ATPase subunit C OS=Leishmania mexi	2
2.016141211	-0.47791	E9AU86	tr|E9AU86|E9AU86_LEIMU Uncharacterized protein OS=Leishmania mexicana (	2
2.244453148	-0.4795	E9AYP5	tr|E9AYP5|E9AYP5_LEIMU Uncharacterized protein OS=Leishmania mexicana (s	4
1.906223595	-0.47998	E9B2V9;E9B	tr|E9B2V9|E9B2V9_LEIMU Superoxide dismutase OS=Leishmania mexicana (str	3
2.589957903	-0.48155	E9B038	tr|E9B038|E9B038_LEIMU Uncharacterized protein OS=Leishmania mexicana (s	10
2.366311198	-0.48205	E9B423	tr|E9B423|E9B423_LEIMU Nucleolar GTP-binding protein 1 OS=Leishmania mex	9
2.324322785	-0.48429	E9B376;A0A	tr|E9B376|E9B376_LEIMU Nucleoside diphosphate kinase OS=Leishmania mexi	9
1.801505519	-0.48448	E9B2W2	tr|E9B2W2|E9B2W2_LEIMU Chaperonin HSP60, mitochondrial,putative OS=Lei	3
3.094520369	-0.48467	E9AQ14	tr|E9AQ14|E9AQ14_LEIMU Uncharacterized protein OS=Leishmania mexicana (	4
1.873314438	-0.48684	E9B1P3;E8N	tr|E9B1P3|E9B1P3_LEIMU Sodium stibogluconate resistance protein,putative C	3
2.187845072	-0.48745	E9ASZ0	tr|E9ASZ0|E9ASZ0_LEIMU Putative DEAD box RNA helicase OS=Leishmania mex	5
2.40642098	-0.48844	E9B0C5	tr|E9B0C5|E9B0C5_LEIMU Uncharacterized protein LMXM_28_3030 OS=Leishn	10
2.805976036	-0.48882	Q8MPM3;E9	tr|Q8MPM3|Q8MPM3_LEIME Transketolase OS=Leishmania mexicana mexican	6
2.213585234	-0.4894	E9AXF3	tr|E9AXF3|E9AXF3_LEIMU Uncharacterized protein OS=Leishmania mexicana (s	7
2.418529134	-0.49219	E9AWL7	tr|E9AWL7|E9AWL7_LEIMU Dynein intermediate-chain-like protein OS=Leishm	3
2.361567427	-0.49657	E9AR97	tr|E9AR97|E9AR97_LEIMU RNA cytidine acetyltransferase OS=Leishmania mexi	4
2.422090612	-0.49788	E9AY66;Q86	tr|E9AY66|E9AY66_LEIMU Putative 40S ribosomal protein S16 OS=Leishmania r	8
3.212827907	-0.49805	E9AWG1	tr|E9AWG1|E9AWG1_LEIMU Uncharacterized protein OS=Leishmania mexicana	2
2.61782969	-0.49809	E9AZ25	tr|E9AZ25|E9AZ25_LEIMU Uncharacterized protein OS=Leishmania mexicana (s	10
2.008413314	-0.49839	E9B489	tr|E9B489|E9B489_LEIMU Uncharacterized protein OS=Leishmania mexicana (s	4
2.911139885	-0.49926	E9AYQ6	tr|E9AYQ6|E9AYQ6_LEIMU CAAX prenyl protease OS=Leishmania mexicana (str	2
2.235463964	-0.50044	E9B1G7	tr|E9B1G7|E9B1G7_LEIMU Putative nucleolar protein OS=Leishmania mexicana	5
2.426166719	-0.50063	E9ASB9	tr|E9ASB9|E9ASB9_LEIMU Histone H4 OS=Leishmania mexicana (strain MHOM/	2
2.704299401	-0.5017	E9AUK2	tr|E9AUK2|E9AUK2_LEIMU Uncharacterized protein OS=Leishmania mexicana (	2
2.818487106	-0.50272	E9ASX8	tr|E9ASX8|E9ASX8_LEIMU Chaperonin HSP60, mitochondrial OS=Leishmania m	5
2.5961774	-0.50563	E9B6Q0	tr|E9B6Q0|E9B6Q0_LEIMU Uncharacterized protein OS=Leishmania mexicana (	10
2.319221801	-0.50955	E9AP27	tr|E9AP27|E9AP27_LEIMU Arginine N-methyltransferase-like protein OS=Leishn	4
2.187070412	-0.51007	E9B0H1;E9A	tr|E9B0H1|E9B0H1_LEIMU Putative aspartyl-tRNA synthetase OS=Leishmania n	11
2.479076224	-0.51068	E9B1C6	tr|E9B1C6|E9B1C6_LEIMU S-adenosylmethionine synthase OS=Leishmania mex	9
2.590650047	-0.51104	E9AKL5	tr|E9AKL5|E9AKL5_LEIMU Uncharacterized protein OS=Leishmania mexicana (s	3
2.635770151	-0.51182	E9AKP2	tr|E9AKP2|E9AKP2_LEIMU Proteasome subunit beta OS=Leishmania mexicana	4
2.017605664	-0.51216	E9ATE6	tr|E9ATE6|E9ATE6_LEIMU Uncharacterized protein OS=Leishmania mexicana (s	8
2.284714306	-0.51353	E9AL37	tr|E9AL37|E9AL37_LEIMU Acetylornithine deacetylase-like protein OS=Leishma	4
2.220184777	-0.51361	E9AR16	tr|E9AR16|E9AR16_LEIMU Uncharacterized protein OS=Leishmania mexicana (s	4
3.139945807	-0.51426	E9AZA4	tr|E9AZA4|E9AZA4_LEIMU Uncharacterized protein OS=Leishmania mexicana (s	2
2.383958704	-0.51511	E9AZU3	tr|E9AZU3|E9AZU3_LEIMU Putative p450 reductase OS=Leishmania mexicana (	2

2.91115854	-0.51587	E9AXN4	tr|E9AXN4|E9AXN4_LEIMU Uncharacterized protein OS=Leishmania mexicana (	3
2.087011167	-0.51699	E9AKX3	tr|E9AKX3|E9AKX3_LEIMU Putative glucosamine-fructose-6-phosphate aminotı	2
2.243129362	-0.51705	Q9GRT0;E9I	tr|Q9GRT0|Q9GRT0_LEIME Casein kinase II alpha subunit OS=Leishmania mexic	2
2.762125735	-0.51721	E9AN60	tr|E9AN60|E9AN60_LEIMU Uncharacterized protein OS=Leishmania mexicana (	20
2.881307575	-0.51809	E9AL83	tr|E9AL83|E9AL83_LEIMU Topoisomerase-related function protein-like protein	2
2.979071264	-0.52052	E9B6R8	tr|E9B6R8|E9B6R8_LEIMU Uncharacterized protein OS=Leishmania mexicana (s	2
1.96724332	-0.52229	E9B528	tr|E9B528|E9B528_LEIMU Glycosyltransferase-like protein OS=Leishmania mex	3
2.262426964	-0.52329	E9B1R9	tr|E9B1R9|E9B1R9_LEIMU Uncharacterized protein OS=Leishmania mexicana (s	2
2.759728837	-0.5236	E9B0B0	tr|E9B0B0|E9B0B0_LEIMU Putative dynein heavy chain OS=Leishmania mexicar	9
2.613513112	-0.52515	E9ATH3	tr|E9ATH3|E9ATH3_LEIMU Adenosylhomocysteinase OS=Leishmania mexicana	13
2.534104048	-0.52576	E9AWN5;E9	tr|E9AWN5|E9AWN5_LEIMU Uncharacterized protein LMXM_24_0450 OS=Leis	4
2.414012812	-0.52601	E9AM73	tr|E9AM73|E9AM73_LEIMU Putative tryptophanyl-tRNA synthetase OS=Leishm	7
2.791854625	-0.52867	E9AQN5	tr|E9AQN5|E9AQN5_LEIMU Transaldolase OS=Leishmania mexicana (strain MH	6
2.312583056	-0.53006	E9ARY3	tr|E9ARY3|E9ARY3_LEIMU GDT1 family protein OS=Leishmania mexicana (strai	2
2.417330743	-0.53197	E9AQA0	tr|E9AQA0|E9AQA0_LEIMU NAD-specific glutamate dehydrogenase OS=Leishm	23
2.544626077	-0.53237	E9ASK9	tr|E9ASK9|E9ASK9_LEIMU Eukaryotic translation initiation factor 6 OS=Leishma	3
2.628373904	-0.53361	E9B5H5	tr|E9B5H5|E9B5H5_LEIMU Uncharacterized protein OS=Leishmania mexicana (	3
2.434161345	-0.53395	E9AZQ9	tr|E9AZQ9|E9AZQ9_LEIMU Uncharacterized protein OS=Leishmania mexicana (	6
2.30796982	-0.53485	E9B052	tr|E9B052|E9B052_LEIMU Eukaryotic translation initiation factor 3 subunit E O:	9
2.02286562	-0.53602	E9AYV2	tr|E9AYV2|E9AYV2_LEIMU Putative calpain-like cysteine peptidase OS=Leishma	2
2.416732819	-0.53664	E9ASM7	tr|E9ASM7|E9ASM7_LEIMU Putative ribosomal protein L24 OS=Leishmania me	3
2.585920607	-0.53795	E9AZS9	tr|E9AZS9|E9AZS9_LEIMU Uncharacterized protein OS=Leishmania mexicana (s	7
2.375785361	-0.5385	E9ALI0	tr|E9ALI0|E9ALI0_LEIMU Phosphodiesterase OS=Leishmania mexicana (strain N	2
2.182113556	-0.53998	E9ALZ8	tr|E9ALZ8|E9ALZ8_LEIMU Uncharacterized protein OS=Leishmania mexicana (s	2
2.451543663	-0.54207	E9AUH1	tr|E9AUH1|E9AUH1_LEIMU Uncharacterized protein OS=Leishmania mexicana	2
2.626984286	-0.54271	E9AMC3	tr|E9AMC3|E9AMC3_LEIMU Uncharacterized protein OS=Leishmania mexicana	5
3.12320698	-0.54369	E9B4J8	tr|E9B4J8|E9B4J8_LEIMU Uncharacterized protein OS=Leishmania mexicana (st	6
2.657243122	-0.54381	E9B406	tr|E9B406|E9B406_LEIMU DNA polymerase OS=Leishmania mexicana (strain M	2
2.940252193	-0.5448	E9AXI9	tr|E9AXI9|E9AXI9_LEIMU DNAj-like protein OS=Leishmania mexicana (strain MI	3
2.673477162	-0.54485	E9B6K2	tr|E9B6K2|E9B6K2_LEIMU Putative cystathione gamma lyase OS=Leishmania m	11
2.506135057	-0.54784	E9ASQ3	tr|E9ASQ3|E9ASQ3_LEIMU Putative N-acetyltransferase subunit Nat1 OS=Leish	9
2.593064131	-0.54926	E9B6T1	tr|E9B6T1|E9B6T1_LEIMU RNA helicase OS=Leishmania mexicana (strain MHOI	3
2.656754354	-0.54947	E9ALW2	tr|E9ALW2|E9ALW2_LEIMU Uncharacterized protein OS=Leishmania mexicana	5
1.858327442	-0.54952	E9B4M8	tr|E9B4M8|E9B4M8_LEIMU Uncharacterized protein OS=Leishmania mexicana	2
2.94781028	-0.54976	E9B260;Q25	tr|E9B260|E9B260_LEIMU DNA-directed RNA polymerase subunit OS=Leishmar	8
2.171845746	-0.54996	E9ALG7	tr|E9ALG7|E9ALG7_LEIMU Putative serine/threonine-protein kinase OS=Leishm	3
2.863455899	-0.55089	E9AKM1	tr|E9AKM1|E9AKM1_LEIMU V-type proton ATPase subunit OS=Leishmania mex	3
2.943473851	-0.55207	E9AW96	tr|E9AW96|E9AW96_LEIMU Actin interacting protein-like protein OS=Leishmar	2
3.433073438	-0.55622	E9AX29	tr|E9AX29|E9AX29_LEIMU Putative lysophospholipase OS=Leishmania mexican	2
2.994730667	-0.55774	E9AJW8	tr|E9AJW8|E9AJW8_LEIMU Uncharacterized protein OS=Leishmania mexicana	2
1.939496179	-0.55961	E9AS02	tr|E9AS02|E9AS02_LEIMU Uncharacterized protein OS=Leishmania mexicana (s	2
2.830158875	-0.55979	E9ARX7	tr|E9ARX7|E9ARX7_LEIMU Kinesin-like protein OS=Leishmania mexicana (strair	6
2.397866344	-0.55989	E9AW21	tr|E9AW21|E9AW21_LEIMU Putative endoribonuclease L-PSP (Pb5) OS=Leishm	3
2.549602117	-0.5635	E9AP70	tr|E9AP70|E9AP70_LEIMU Uncharacterized protein OS=Leishmania mexicana (s	2
2.936860871	-0.56453	E9AJZ0	tr|E9AJZ0|E9AJZ0_LEIMU DNA topoisomerase type IB small subunit OS=Leishm	8
2.512828172	-0.5651	E9B2V7	tr|E9B2V7|E9B2V7_LEIMU Uncharacterized protein OS=Leishmania mexicana (s	2
3.176011488	-0.56603	E9AJK7	tr|E9AJK7|E9AJK7_LEIMU Uncharacterized protein OS=Leishmania mexicana (st	5
2.290839608	-0.56689	E9B1K4	tr|E9B1K4|E9B1K4_LEIMU Putative ATP-binding cassette protein subfamily D, r	3
3.405303342	-0.56782	E9B028	tr|E9B028|E9B028_LEIMU Putative mitochondrial DEAD box protein OS=Leishm	2
2.939488454	-0.56902	E9B1G4	tr|E9B1G4|E9B1G4_LEIMU DNA-directed RNA polymerase subunit beta OS=Lei:	4
2.920210822	-0.57197	E9ASS0	tr|E9ASS0|E9ASS0_LEIMU Uncharacterized protein OS=Leishmania mexicana (s	5
1.676631498	-0.57274	E9AJ91	tr|E9AJ91|E9AJ91_LEIMU Putative carboxylase OS=Leishmania mexicana (strair	2
2.679424228	-0.57335	E9AU61	tr|E9AU61|E9AU61_LEIMU Putative chaperone protein DNAj OS=Leishmania m	4
2.931411611	-0.57387	E9APV2	tr|E9APV2|E9APV2_LEIMU Putative dynein heavy chain OS=Leishmania mexica	2
2.258599999	-0.57414	E9AXX0	tr|E9AXX0|E9AXX0_LEIMU Uncharacterized protein OS=Leishmania mexicana (s	2
2.622180005	-0.57435	E9B2E8	tr|E9B2E8|E9B2E8_LEIMU Uncharacterized protein OS=Leishmania mexicana (s	13
2.715858061	-0.57523	E9AXJ1	tr|E9AXJ1|E9AXJ1_LEIMU Aldehyde dehydrogenase, mitochondrial OS=Leishma	10
2.950137027	-0.57962	E9B6S8	tr|E9B6S8|E9B6S8_LEIMU Uncharacterized protein (Map kinase kinase-like pro	2
2.375546538	-0.58146	E9B6I6	tr|E9B6I6|E9B6I6_LEIMU Uncharacterized protein OS=Leishmania mexicana (st	2
3.233867623	-0.58234	E9B1B8	tr|E9B1B8|E9B1B8_LEIMU Uncharacterized protein OS=Leishmania mexicana (s	4
2.425582013	-0.5832	E9AZC8	tr|E9AZC8|E9AZC8_LEIMU Putative ribosomal protein L11 OS=Leishmania mexi	2
2.076859238	-0.58356	E9B760	tr|E9B760|E9B760_LEIMU Uncharacterized protein OS=Leishmania mexicana (s	6
2.546404053	-0.58538	E9B0K3	tr|E9B0K3|E9B0K3_LEIMU Uncharacterized protein OS=Leishmania mexicana (s	6

3.061418993	-0.58801	E9B6A7	tr|E9B6A7|E9B6A7_LEIMU ATP-dependent RNA helicase-like protein OS=Leishr	3
2.861758928	-0.58859	E9B047	tr|E9B047|E9B047_LEIMU Putative glycosomal membrane protein OS=Leishma	3
2.208530692	-0.58912	E9B5K8	tr|E9B5K8|E9B5K8_LEIMU Uncharacterized protein OS=Leishmania mexicana (s	6
2.645619736	-0.59095	E9AXH6	tr|E9AXH6|E9AXH6_LEIMU Putative dynein heavy chain OS=Leishmania mexica	42
3.227466522	-0.59128	Q95PT4;E9A	tr|Q95PT4|Q95PT4_LEIME Myo-inositol-1-phosphate synthase OS=Leishmania	6
4.042447956	-0.59217	E9AXP0	tr|E9AXP0|E9AXP0_LEIMU Uncharacterized protein OS=Leishmania mexicana (s	2
2.457650434	-0.59313	E9B5C7	tr|E9B5C7|E9B5C7_LEIMU Glucose-6-phosphate 1-epimerase OS=Leishmania m	4
3.085817733	-0.59717	E9AYQ7	tr|E9AYQ7|E9AYQ7_LEIMU RNA helicase OS=Leishmania mexicana (strain MHC	2
2.678731746	-0.59846	E9ALL2	tr|E9ALL2|E9ALL2_LEIMU Uncharacterized protein OS=Leishmania mexicana (st	2
3.563822537	-0.59898	E9APR2	tr|E9APR2|E9APR2_LEIMU Elongation of fatty acids protein OS=Leishmania me	2
2.785276021	-0.59953	E9B478	tr|E9B478|E9B478_LEIMU Putative heat shock protein OS=Leishmania mexican	18
3.805643873	-0.60012	E9ALF3	tr|E9ALF3|E9ALF3_LEIMU Uncharacterized protein OS=Leishmania mexicana (s	2
2.921704451	-0.60016	E9APS7	tr|E9APS7|E9APS7_LEIMU Uncharacterized protein OS=Leishmania mexicana (s	2
2.382340392	-0.60094	E9AZZ2	tr|E9AZZ2|E9AZZ2_LEIMU Proteasome regulatory non-ATP-ase subunit 2,putat	6
2.907830938	-0.60117	E9B4W0	tr|E9B4W0|E9B4W0_LEIMU Uncharacterized protein OS=Leishmania mexicana	7
2.809452469	-0.60209	E9ASX7	tr|E9ASX7|E9ASX7_LEIMU Chaperonin HSP60, mitochondrial OS=Leishmania m	10
2.476424248	-0.60289	E9AUT3	tr|E9AUT3|E9AUT3_LEIMU Uncharacterized protein OS=Leishmania mexicana (	3
2.495502327	-0.60385	E9AX93	tr|E9AX93|E9AX93_LEIMU Cleavage and polyadenylation specificity factor subu	4
2.498683486	-0.60429	E9B191	tr|E9B191|E9B191_LEIMU Uncharacterized protein OS=Leishmania mexicana (s	3
2.71332436	-0.60498	E9AX57	tr|E9AX57|E9AX57_LEIMU Uncharacterized protein OS=Leishmania mexicana (s	9
3.332180366	-0.60563	E9B5D2	tr|E9B5D2|E9B5D2_LEIMU Cleavage and polyadenylation specificity factor,puta	3
2.301879827	-0.6059	E9AVY2	tr|E9AVY2|E9AVY2_LEIMU Putative ATP-dependent DEAD/H RNA helicase OS=L	7
2.327005562	-0.60946	E9B2Q9	tr|E9B2Q9|E9B2Q9_LEIMU Cleavage and polyadenylation specificity factor-like	7
2.503112685	-0.61145	E9AW08	tr|E9AW08|E9AW08_LEIMU Uncharacterized protein OS=Leishmania mexicana	11
2.140800617	-0.61206	E9AX44	tr|E9AX44|E9AX44_LEIMU Uncharacterized protein OS=Leishmania mexicana (s	2
2.947565705	-0.61526	E9B2V1	tr|E9B2V1|E9B2V1_LEIMU Uncharacterized protein OS=Leishmania mexicana (s	7
2.688327374	-0.61581	E9AL32	tr|E9AL32|E9AL32_LEIMU Uncharacterized protein OS=Leishmania mexicana (s	4
2.283537571	-0.61795	E9AXT4	tr|E9AXT4|E9AXT4_LEIMU Uncharacterized protein OS=Leishmania mexicana (s	2
2.290570435	-0.61802	E9AVM2	tr|E9AVM2|E9AVM2_LEIMU Uncharacterized protein OS=Leishmania mexicana	6
3.043929767	-0.61806	E9ASE2	tr|E9ASE2|E9ASE2_LEIMU eIF3-interacting protein-like protein OS=Leishmania	11
2.809687786	-0.61962	E9AUA1;Q8	tr|E9AUA1|E9AUA1_LEIMU 2,3-bisphosphoglycerate-independent phosphoglyc	9
2.520890832	-0.62049	E9AU90	tr|E9AU90|E9AU90_LEIMU Similarity to endo-1-like protein OS=Leishmania me	3
2.358095965	-0.6215	E9AVN0	tr|E9AVN0|E9AVN0_LEIMU Uncharacterized protein OS=Leishmania mexicana	4
3.59999097	-0.62214	E9B5Z2;E9B	tr|E9B5Z2|E9B5Z2_LEIMU Oligosaccharyl transferase-like protein OS=Leishman	3
3.21694525	-0.62468	E9APH6	tr|E9APH6|E9APH6_LEIMU Uncharacterized protein OS=Leishmania mexicana (	2
2.540335363	-0.6247	E9ANU8	tr|E9ANU8|E9ANU8_LEIMU Proteasome regulatory ATPase subunittcc1l8.3,put	3
4.049884025	-0.62535	E9AK09	tr|E9AK09|E9AK09_LEIMU Uncharacterized protein OS=Leishmania mexicana (s	2
2.966490753	-0.62828	E9AYZ0	tr|E9AYZ0|E9AYZ0_LEIMU Putative 2-oxoglutarate dehydrogenase subunit OS=	7
2.521235839	-0.62981	E9B6L0	tr|E9B6L0|E9B6L0_LEIMU Uncharacterized protein OS=Leishmania mexicana (s	6
3.306910269	-0.63014	E9ALJ3	tr|E9ALJ3|E9ALJ3_LEIMU Enoyl-CoA hydratase/isomerase-like protein OS=Leish	3
2.072218512	-0.63325	E9B1M3	tr|E9B1M3|E9B1M3_LEIMU Uncharacterized protein OS=Leishmania mexicana	3
2.5803723	-0.6346	E9AZ13	tr|E9AZ13|E9AZ13_LEIMU Mitochondrial RNA binding protein 1 OS=Leishmania	3
3.110420872	-0.63512	E9AVH3	tr|E9AVH3|E9AVH3_LEIMU Putative ATP synthase F1 subunit gamma protein O	6
1.838000505	-0.63538	E9B1B1	tr|E9B1B1|E9B1B1_LEIMU Putative calmodulin-related protein OS=Leishmania	5
2.64860536	-0.63629	E9AMU6	tr|E9AMU6|E9AMU6_LEIMU Uncharacterized protein OS=Leishmania mexicana	2
2.61583031	-0.6365	E9AKA4;C6K	tr|E9AKA4|E9AKA4_LEIMU Actin OS=Leishmania mexicana (strain MHOM/GT/2	6
2.94106327	-0.6365	E9ASP6;Q9U	tr|E9ASP6|E9ASP6_LEIMU Fructose-bisphosphate aldolase OS=Leishmania mex	11
2.970015039	-0.63681	E9AQX4	tr|E9AQX4|E9AQX4_LEIMU Putative prohibitin OS=Leishmania mexicana (strain	3
2.953933804	-0.63915	E9B520	tr|E9B520|E9B520_LEIMU Putative asparaginyl-tRNA synthetase OS=Leishmani	8
2.342008539	-0.63981	E9B4K7	tr|E9B4K7|E9B4K7_LEIMU Uncharacterized protein (Fragment) OS=Leishmania	3
2.017370766	-0.6406	E9AY60;E9A	tr|E9AY60|E9AY60_LEIMU Glutathione peroxidase OS=Leishmania mexicana (st	2
2.198720064	-0.644	E9ARY1	tr|E9ARY1|E9ARY1_LEIMU Putative RNA binding protein OS=Leishmania mexica	3
2.092586594	-0.64642	E9B6C8	tr|E9B6C8|E9B6C8_LEIMU Uncharacterized protein OS=Leishmania mexicana (s	3
2.411570806	-0.64718	E9AS04	tr|E9AS04|E9AS04_LEIMU Uncharacterized protein OS=Leishmania mexicana (s	2
2.949296133	-0.64758	E9ATW8	tr|E9ATW8|E9ATW8_LEIMU Uncharacterized protein OS=Leishmania mexicana	10
2.52152246	-0.64876	E9B258	tr|E9B258|E9B258_LEIMU Uncharacterized protein OS=Leishmania mexicana (s	5
2.701282828	-0.64993	E9ANZ4	tr|E9ANZ4|E9ANZ4_LEIMU Cytochrome c oxidase subunit IV OS=Leishmania me	6
2.398926868	-0.65244	E9AX96	tr|E9AX96|E9AX96_LEIMU Uncharacterized protein OS=Leishmania mexicana (s	4
2.939941276	-0.65443	E9AUV0	tr|E9AUV0|E9AUV0_LEIMU Soluble n-ethylmaleimide sensitive factor,putative	3
2.63818313	-0.65666	E9B6C2	tr|E9B6C2|E9B6C2_LEIMU Putative phosphoinositide-binding protein OS=Leish	2
2.845880666	-0.65716	E9APV8;E8N	tr|E9APV8|E9APV8_LEIMU Putative kinesin K39 OS=Leishmania mexicana (strai	4
3.252670701	-0.65727	E9ALL3	tr|E9ALL3|E9ALL3_LEIMU Uncharacterized protein OS=Leishmania mexicana (st	3
2.914746794	-0.66486	E9AJY9	tr|E9AJY9|E9AJY9_LEIMU Uncharacterized protein OS=Leishmania mexicana (st	4

2.880724135	-0.66567	E9B235	tr|E9B235|E9B235_LEIMU Uncharacterized protein OS=Leishmania mexicana (s	4
2.5501121	-0.66659	E9AL62;E9A	tr|E9AL62|E9AL62_LEIMU Putative 60S ribosomal protein L7a OS=Leishmania m	13
2.67232997	-0.66912	E9B0S3	tr|E9B0S3|E9B0S3_LEIMU Putative kinesin OS=Leishmania mexicana (strain MH	5
2.561276775	-0.67051	E9AUS4	tr|E9AUS4|E9AUS4_LEIMU Coatomer subunit beta OS=Leishmania mexicana (s	9
2.907689876	-0.67128	E9ATF1	tr|E9ATF1|E9ATF1_LEIMU Uncharacterized protein OS=Leishmania mexicana (s	2
3.451415824	-0.67224	E9ATL1	tr|E9ATL1|E9ATL1_LEIMU Uncharacterized protein OS=Leishmania mexicana (s	3
3.175141558	-0.6725	E9B405;A0A	tr|E9B405|E9B405_LEIMU Thiamine biosynthesis-like protein OS=Leishmania m	4
2.725948901	-0.67276	E9ARY4	tr|E9ARY4|E9ARY4_LEIMU Intraflagellar transport protein component,putative	5
2.972137159	-0.67569	E9B1S0	tr|E9B1S0|E9B1S0_LEIMU Putative vacuolar-type proton translocating pyropho	10
3.28038193	-0.67606	E9B178	tr|E9B178|E9B178_LEIMU Eukaryotic translation initiation factor 3 subunit 7-lik	4
2.470804031	-0.67682	E9AS17	tr|E9AS17|E9AS17_LEIMU Uncharacterized protein OS=Leishmania mexicana (s	10
2.971689115	-0.67866	Q25332;Q2!	tr|Q25332|Q25332_LEIME Secreted acid phosphatase 1 (SAP1) OS=Leishmania	7
3.630632443	-0.67918	E9AJF2	tr|E9AJF2|E9AJF2_LEIMU Mitochondrial processing peptide beta subunit,putati	2
3.14026444	-0.6794	E9ASC5	tr|E9ASC5|E9ASC5_LEIMU Stress-inducible protein STI1 homolog OS=Leishman	8
2.408043977	-0.68046	E9B5N7	tr|E9B5N7|E9B5N7_LEIMU Uncharacterized protein OS=Leishmania mexicana (	3
3.14472146	-0.68058	E9AVX3	tr|E9AVX3|E9AVX3_LEIMU Putative centrin (Ca2+-binding ef-hand protein) OS=	3
3.460420271	-0.6814	E9ARY5	tr|E9ARY5|E9ARY5_LEIMU Uncharacterized protein OS=Leishmania mexicana (s	5
3.070616376	-0.68181	E9APV6	tr|E9APV6|E9APV6_LEIMU Putative kinesin K39 OS=Leishmania mexicana (strai	2
3.014548445	-0.6821	E9B1L9	tr|E9B1L9|E9B1L9_LEIMU Uncharacterized protein OS=Leishmania mexicana (s	4
3.151005718	-0.68267	E9B4X4	tr|E9B4X4|E9B4X4_LEIMU Uncharacterized protein OS=Leishmania mexicana (s	5
3.18060621	-0.68444	E9AZ28	tr|E9AZ28|E9AZ28_LEIMU Uncharacterized protein OS=Leishmania mexicana (s	3
3.013250352	-0.68487	E9AMV9;E9.	tr|E9AMV9|E9AMV9_LEIMU Putative eukaryotic translation initiation factor 2 s	11
2.165698462	-0.68667	E9AT19	tr|E9AT19|E9AT19_LEIMU Dipeptidyl-peptidase 8-like serine peptidase,putative	2
2.671684511	-0.68668	E9B3A4	tr|E9B3A4|E9B3A4_LEIMU Uncharacterized protein OS=Leishmania mexicana (s	2
2.734647341	-0.68792	E9AMC0	tr|E9AMC0|E9AMC0_LEIMU Uncharacterized protein OS=Leishmania mexicana	2
3.072584907	-0.68821	E9AYD6	tr|E9AYD6|E9AYD6_LEIMU Putative thimet oligopeptidase (Metallo-peptidase,	7
2.917511444	-0.69053	E9AP50	tr|E9AP50|E9AP50_LEIMU Uncharacterized protein OS=Leishmania mexicana (s	9
3.193122482	-0.69101	E9APK7;A0A	tr|E9APK7|E9APK7_LEIMU Nucleoside hydrolase-like protein OS=Leishmania m	3
2.98561104	-0.69128	E9AQ19	tr|E9AQ19|E9AQ19_LEIMU Putative 60S ribosomal protein L13a OS=Leishmania	7
2.544238918	-0.69146	E9B3P4	tr|E9B3P4|E9B3P4_LEIMU Uncharacterized protein OS=Leishmania mexicana (s	2
3.278880738	-0.69236	E9AM17	tr|E9AM17|E9AM17_LEIMU Uncharacterized protein OS=Leishmania mexicana	2
3.002802381	-0.69253	E9B612	tr|E9B612|E9B612_LEIMU Uncharacterized protein OS=Leishmania mexicana (s	4
2.671078162	-0.69444	E9AUN0	tr|E9AUN0|E9AUN0_LEIMU Uncharacterized protein OS=Leishmania mexicana	2
3.204519993	-0.69447	E9AN72	tr|E9AN72|E9AN72_LEIMU Uncharacterized protein OS=Leishmania mexicana (	5
2.529795311	-0.69826	E9AVC7	tr|E9AVC7|E9AVC7_LEIMU 2-oxoisovalerate dehydrogenase subunit alpha OS=	3
3.323503486	-0.70431	E9AP95	tr|E9AP95|E9AP95_LEIMU Uncharacterized protein OS=Leishmania mexicana (s	3
2.767209628	-0.70447	E9ARN6	tr|E9ARN6|E9ARN6_LEIMU UTP--glucose-1-phosphate uridylyltransferase OS=L	2
3.189512243	-0.70454	E9API4	tr|E9API4|E9API4_LEIMU Uncharacterized protein OS=Leishmania mexicana (st	2
3.488841755	-0.70571	E9AZA2	tr|E9AZA2|E9AZA2_LEIMU Putative rRNA methyltransferase OS=Leishmania me	7
2.681528071	-0.70711	E9APF9	tr|E9APF9|E9APF9_LEIMU Uncharacterized protein OS=Leishmania mexicana (s	3
4.157560418	-0.70941	E9B602	tr|E9B602|E9B602_LEIMU Putative short chain dehydrogenase OS=Leishmania	2
3.290273362	-0.70968	E9B637	tr|E9B637|E9B637_LEIMU Uncharacterized protein OS=Leishmania mexicana (s	2
2.412946903	-0.71055	E9AL16	tr|E9AL16|E9AL16_LEIMU Putative cytochrome c1, heme protein, mitochondria	3
2.604660211	-0.713	E9AJH6	tr|E9AJH6|E9AJH6_LEIMU Uncharacterized protein OS=Leishmania mexicana (s	2
3.139858995	-0.71374	E9B4K3	tr|E9B4K3|E9B4K3_LEIMU Eukaryotic translation initiation factor 5,putative OS	5
2.570932086	-0.71463	E9AV43	tr|E9AV43|E9AV43_LEIMU Uncharacterized protein OS=Leishmania mexicana (	3
2.398334707	-0.7161	E9ATP9	tr|E9ATP9|E9ATP9_LEIMU Ribosome assembly factor mrt4 OS=Leishmania mex	4
2.445522667	-0.71721	E9ALS6	tr|E9ALS6|E9ALS6_LEIMU Uncharacterized protein OS=Leishmania mexicana (s	5
2.949913697	-0.71744	E9ATX7	tr|E9ATX7|E9ATX7_LEIMU Putative branch point binding protein OS=Leishmani	2
3.549314881	-0.71909	E9B428	tr|E9B428|E9B428_LEIMU Uncharacterized protein OS=Leishmania mexicana (s	4
3.473377874	-0.72016	E9ANZ0	tr|E9ANZ0|E9ANZ0_LEIMU Putative alanine aminotransferase OS=Leishmania r	4
3.034671818	-0.72031	E9B4Q3;E9E	tr|E9B4Q3|E9B4Q3_LEIMU Elongation factor 1-beta OS=Leishmania mexicana (	5
2.995944325	-0.72039	E9ALS3	tr|E9ALS3|E9ALS3_LEIMU Uncharacterized protein OS=Leishmania mexicana (s	3
4.464797794	-0.72111	E9AJI9	tr|E9AJI9|E9AJI9_LEIMU Small GTP binding protein rab6-like protein OS=Leishm	2
3.1875335	-0.72398	E9B493	tr|E9B493|E9B493_LEIMU Putative carboxypeptidase OS=Leishmania mexicana	5
2.449674401	-0.72405	E9ANI7	tr|E9ANI7|E9ANI7_LEIMU Ribosome biogenesis protein BOP1 homolog OS=Leis	2
3.081789487	-0.72603	E9AL58	tr|E9AL58|E9AL58_LEIMU Uncharacterized protein OS=Leishmania mexicana (s	6
4.081638062	-0.72622	E9B6F2	tr|E9B6F2|E9B6F2_LEIMU Putative acyl-CoA dehydrogenase OS=Leishmania me	3
3.002945029	-0.72922	E9AR24	tr|E9AR24|E9AR24_LEIMU Putative RNA-binding protein OS=Leishmania mexic	3
3.210740015	-0.73202	E9AUC5	tr|E9AUC5|E9AUC5_LEIMU Putative nuclear pore complex protein (NUP155) OS	3
3.266057292	-0.73214	E9AJY2;A0A	tr|E9AJY2|E9AJY2_LEIMU Elongation initiation factor 2 alpha subunit,putative (	12
3.178584181	-0.73749	E9B665	tr|E9B665|E9B665_LEIMU Putative 60S ribosomal protein L5 OS=Leishmania m	8
2.522741275	-0.73763	E9AQC4	tr|E9AQC4|E9AQC4_LEIMU Uncharacterized protein OS=Leishmania mexicana	4

3.134314629	-0.73841	E9B307	tr|E9B307|E9B307_LEIMU Uncharacterized protein OS=Leishmania mexicana (s	3
3.261750594	-0.73868	E9API5;E9A.	tr|E9API5|E9API5_LEIMU Kinesin-like protein OS=Leishmania mexicana (strain I	3
3.333822182	-0.73954	E9AZ89;A5J'	tr|E9AZ89|E9AZ89_LEIMU Putative trypanothione synthetase OS=Leishmania n	4
3.621690033	-0.74013	E9B552	tr|E9B552|E9B552_LEIMU RuvB-like helicase OS=Leishmania mexicana (strain M	3
3.252559319	-0.74028	E9AMI2	tr|E9AMI2|E9AMI2_LEIMU Uncharacterized protein OS=Leishmania mexicana (	4
2.780060408	-0.74058	E9B5F7	tr|E9B5F7|E9B5F7_LEIMU Transmembrane 9 superfamily member OS=Leishma	2
3.095051865	-0.74264	E9B5S0	tr|E9B5S0|E9B5S0_LEIMU Putative serine palmitoyltransferase OS=Leishmania	3
3.224058202	-0.74375	E9API9	tr|E9API9|E9API9_LEIMU Putative dynein heavy chain OS=Leishmania mexicana	43
2.512654782	-0.74407	E9AVX7	tr|E9AVX7|E9AVX7_LEIMU Peptidyl-prolyl cis-trans isomerase OS=Leishmania r	2
3.583445153	-0.74693	E9APW9	tr|E9APW9|E9APW9_LEIMU Uncharacterized protein OS=Leishmania mexicana	8
3.121316111	-0.74956	E9APE7;E9A	tr|E9APE7|E9APE7_LEIMU 40S ribosomal protein S4 OS=Leishmania mexicana (	10
2.839358031	-0.75209	E9B0G2	tr|E9B0G2|E9B0G2_LEIMU Putative phosphatase 2C OS=Leishmania mexicana (	6
2.788768164	-0.75281	E9AKE4	tr|E9AKE4|E9AKE4_LEIMU Putative microtubule-associated protein (Fragment)	3
3.310235152	-0.75354	E9B2X4	tr|E9B2X4|E9B2X4_LEIMU Putative 1-acyl-sn-glycerol-3-phosphateacyltransfera	3
3.092495791	-0.75683	Q9NGK2;E9.	tr|Q9NGK2|Q9NGK2_LEIAM 40S ribosomal protein S24 OS=Leishmania amazon	5
3.59500911	-0.75707	E9AX09	tr|E9AX09|E9AX09_LEIMU Fatty acyl-CoA reductase OS=Leishmania mexicana (	3
3.418103293	-0.75737	E9ATK7	tr|E9ATK7|E9ATK7_LEIMU Uncharacterized protein OS=Leishmania mexicana (s	10
3.041296608	-0.75869	E9ASA6	tr|E9ASA6|E9ASA6_LEIMU Peptidylprolyl isomerase OS=Leishmania mexicana (	4
2.916991315	-0.75902	E9B0J1	tr|E9B0J1|E9B0J1_LEIMU Uncharacterized protein OS=Leishmania mexicana (st	2
2.986501204	-0.75992	E9B3L9	tr|E9B3L9|E9B3L9_LEIMU Putative DNA mismatch repair protein OS=Leishmani	2
3.183077617	-0.76019	E9B3H7	tr|E9B3H7|E9B3H7_LEIMU Putative GIPL galf transferase OS=Leishmania mexic	9
2.669872924	-0.76081	E9AP43	tr|E9AP43|E9AP43_LEIMU Putative carboxypeptidase (Metallo-peptidase, clan	5
2.893742885	-0.76147	E9AVR1	tr|E9AVR1|E9AVR1_LEIMU Putative NADH-cytochrome b5 reductase OS=Leishn	6
2.957532247	-0.76151	E9AK37	tr|E9AK37|E9AK37_LEIMU Uncharacterized protein OS=Leishmania mexicana (s	3
3.220804265	-0.7625	E9ARE6	tr|E9ARE6|E9ARE6_LEIMU Putative alpha glucosidase II subunit OS=Leishmania	2
2.941212212	-0.76556	E9AQL0	tr|E9AQL0|E9AQL0_LEIMU Uncharacterized protein OS=Leishmania mexicana (	4
3.079942665	-0.7658	E9ATK5	tr|E9ATK5|E9ATK5_LEIMU Uncharacterized protein OS=Leishmania mexicana (s	8
3.318958302	-0.7669	E9AW71	tr|E9AW71|E9AW71_LEIMU Kinesin-like protein OS=Leishmania mexicana (stra	7
2.832812522	-0.76759	E9ANV2	tr|E9ANV2|E9ANV2_LEIMU Putative cysteinyl-tRNA synthetase OS=Leishmania	7
3.102746669	-0.76779	E9APJ0	tr|E9APJ0|E9APJ0_LEIMU Putative chaperonin TCP20 OS=Leishmania mexicana	10
3.254527132	-0.76894	E9AWI0	tr|E9AWI0|E9AWI0_LEIMU Pyridoxal phosphate homeostasis protein OS=Leish	7
3.057437971	-0.77378	E9B384	tr|E9B384|E9B384_LEIMU Uncharacterized protein OS=Leishmania mexicana (s	15
2.460382837	-0.77432	Q9GRU2;E9.	tr|Q9GRU2|Q9GRU2_LEIME Mitogen-activated protein kinase OS=Leishmania r	3
2.760809845	-0.77434	E9ANC5	tr|E9ANC5|E9ANC5_LEIMU Uncharacterized protein OS=Leishmania mexicana (	4
3.163652653	-0.77447	E9B2C2	tr|E9B2C2|E9B2C2_LEIMU Uncharacterized protein OS=Leishmania mexicana (s	13
3.227904514	-0.77511	E9AKE1;Q0(	tr|E9AKE1|E9AKE1_LEIMU Trypanothione reductase OS=Leishmania mexicana (	7
3.625794962	-0.77558	E9AV44	tr|E9AV44|E9AV44_LEIMU Proteasome regulatory non-ATP-ase subunit 5,puta	3
3.290933256	-0.77782	E9ANQ6	tr|E9ANQ6|E9ANQ6_LEIMU Putative eukaryotic release factor 3 OS=Leishmania	7
3.451558202	-0.77849	E9B494	tr|E9B494|E9B494_LEIMU Isocitrate dehydrogenase [NADP] OS=Leishmania me	4
2.95365532	-0.78	E9B078	tr|E9B078|E9B078_LEIMU Putative 40S ribosomal protein S17 OS=Leishmania r	6
3.057792968	-0.78447	E9AQ51	tr|E9AQ51|E9AQ51_LEIMU Uncharacterized protein OS=Leishmania mexicana (	4
3.087215746	-0.78455	E9AY83	tr|E9AY83|E9AY83_LEIMU Uncharacterized protein OS=Leishmania mexicana (s	5
2.550557858	-0.78652	E9AZ34	tr|E9AZ34|E9AZ34_LEIMU Uncharacterized protein OS=Leishmania mexicana (s	2
3.136406286	-0.78831	E9B4L7	tr|E9B4L7|E9B4L7_LEIMU Putative importin beta-1 subunit OS=Leishmania me:	3
3.065862849	-0.79199	E9AZ49	tr|E9AZ49|E9AZ49_LEIMU Uncharacterized protein OS=Leishmania mexicana (s	2
3.016364711	-0.79258	E9ALV8;A5J'	tr|E9ALV8|E9ALV8_LEIMU Putative tryparedoxin OS=Leishmania mexicana (stra	5
3.07236494	-0.79324	E9AVH4	tr|E9AVH4|E9AVH4_LEIMU 40S ribosomal protein S6 OS=Leishmania mexicana	9
2.872624819	-0.79496	E9ARW5	tr|E9ARW5|E9ARW5_LEIMU Uncharacterized protein OS=Leishmania mexicana	3
3.722846288	-0.79655	E9ARI8	tr|E9ARI8|E9ARI8_LEIMU Aconitate hydratase OS=Leishmania mexicana (strain	19
2.751054266	-0.7977	E9B4J1	tr|E9B4J1|E9B4J1_LEIMU Uncharacterized protein OS=Leishmania mexicana (st	5
2.883989012	-0.79895	E9B5Z0	tr|E9B5Z0|E9B5Z0_LEIMU Uncharacterized protein OS=Leishmania mexicana (s	2
3.071828087	-0.79911	E9ASP5;E9A	tr|E9ASP5|E9ASP5_LEIMU Putative 40S ribosomal protein S9 OS=Leishmania m	6
3.171989961	-0.80238	E9AY41	tr|E9AY41|E9AY41_LEIMU Putative 10 kDa heat shock protein OS=Leishmania r	4
3.255387805	-0.80318	E9AKW6	tr|E9AKW6|E9AKW6_LEIMU Putative acyl-coenzyme a dehydrogenase OS=Leisl	3
3.187245219	-0.80328	E9AY45;A7Y	tr|E9AY45|E9AY45_LEIMU Uncharacterized protein OS=Leishmania mexicana (s	6
2.687600298	-0.80476	E9ALM8	tr|E9ALM8|E9ALM8_LEIMU Putative fumarate hydratase (Fragment) OS=Leishn	2
2.919778112	-0.8048	E9APR0	tr|E9APR0|E9APR0_LEIMU Elongation of fatty acids protein OS=Leishmania me	3
3.04164485	-0.80527	E9AXX1	tr|E9AXX1|E9AXX1_LEIMU Putative adenylate kinase OS=Leishmania mexicana	2
2.363387249	-0.80562	E9AWM1	tr|E9AWM1|E9AWM1_LEIMU Putative fumarate hydratase OS=Leishmania me:	12
3.863365268	-0.80597	E9AQS3	tr|E9AQS3|E9AQS3_LEIMU Tyrosyl or methionyl-tRNA synthetase-like protein (	3
3.469857385	-0.80712	E9APD3	tr|E9APD3|E9APD3_LEIMU Putative proteasome regulatory ATPase subunit 2 C	4
2.661633671	-0.80926	E9B6S6	tr|E9B6S6|E9B6S6_LEIMU Uncharacterized protein OS=Leishmania mexicana (s	4
2.627943532	-0.80996	E9AQD9	tr|E9AQD9|E9AQD9_LEIMU Putative glutaminyl-tRNA synthetase OS=Leishman	6

2.982433365	-0.8101	E9B1S6;E9B	tr|E9B1S6|E9B1S6_LEIMU Putative p-glycoprotein e OS=Leishmania mexicana (	2
2.864697431	-0.81012	E9B259	tr|E9B259|E9B259_LEIMU Putative calreticulin OS=Leishmania mexicana (strair	2
3.463671036	-0.81054	E9B716	tr|E9B716|E9B716_LEIMU Uncharacterized protein OS=Leishmania mexicana (s	3
3.749331231	-0.81132	E9ALE4	tr|E9ALE4|E9ALE4_LEIMU Uncharacterized protein OS=Leishmania mexicana (s	3
3.417311568	-0.81435	E9AKP0	tr|E9AKP0|E9AKP0_LEIMU Peptidyl-prolyl cis-trans isomerase OS=Leishmania n	3
3.223156582	-0.81518	E9AT73	tr|E9AT73|E9AT73_LEIMU Succinate--CoA ligase [ADP-forming] subunit beta, m	10
3.049040398	-0.81552	E9B5P1;Q27	tr|E9B5P1|E9B5P1_LEIMU Pyruvate kinase OS=Leishmania mexicana (strain MH	6
2.955630773	-0.81575	E9AS06	tr|E9AS06|E9AS06_LEIMU Methionine aminopeptidase OS=Leishmania mexicar	2
3.570970201	-0.81625	E9AJY1	tr|E9AJY1|E9AJY1_LEIMU Uncharacterized protein OS=Leishmania mexicana (st	4
3.43613767	-0.81641	E9B378	tr|E9B378|E9B378_LEIMU Tubulin-specific chaperone A OS=Leishmania mexica	2
2.858835589	-0.81682	E9B1R0	tr|E9B1R0|E9B1R0_LEIMU Putative N-acyl-L-amino acid amidohydrolase OS=Le	2
2.41583887	-0.81739	E9B0K5	tr|E9B0K5|E9B0K5_LEIMU Mitochondrial oligo_U binding protein TBRGG1,puta	2
3.343930006	-0.82065	E9AUZ2;E9A	tr|E9AUZ2|E9AUZ2_LEIMU Phosphotransferase OS=Leishmania mexicana (strai	17
3.290684003	-0.8218	E9ALK3	tr|E9ALK3|E9ALK3_LEIMU Putative GTP-binding protein OS=Leishmania mexica	13
2.217174701	-0.82205	E9ARD7;E9A	tr|E9ARD7|E9ARD7_LEIMU Receptor-type adenylate cyclase a OS=Leishmania r	5
3.331668419	-0.82297	E9AVE1	tr|E9AVE1|E9AVE1_LEIMU Putative RNA helicase OS=Leishmania mexicana (str	9
3.048548852	-0.82525	E9ANH0	tr|E9ANH0|E9ANH0_LEIMU Proteasome subunit alpha type OS=Leishmania me	2
4.131788758	-0.826	E9B4S6	tr|E9B4S6|E9B4S6_LEIMU Putative uracil phosphoribosyltransferase OS=Leishn	3
3.273896884	-0.82866	E9B5Z9	tr|E9B5Z9|E9B5Z9_LEIMU Putative pre-mRNA splicing factor ATP-dependent RI	2
3.083332162	-0.8292	E9B620	tr|E9B620|E9B620_LEIMU Putative threonyl-tRNA synthetase OS=Leishmania n	6
3.673408034	-0.82959	E9AVB9	tr|E9AVB9|E9AVB9_LEIMU V-type proton ATPase subunit H OS=Leishmania me	3
2.38762938	-0.83189	E9ARI3	tr|E9ARI3|E9ARI3_LEIMU Putative tubulin-specific chaperone OS=Leishmania m	2
3.248275554	-0.83733	E9ALH7	tr|E9ALH7|E9ALH7_LEIMU Putative 60S ribosomal protein L13 OS=Leishmania i	8
2.795395464	-0.83733	E9B0I8	tr|E9B0I8|E9B0I8_LEIMU Putative histidyl-tRNA synthetase OS=Leishmania me:	4
3.017856443	-0.83966	E9B6T5	tr|E9B6T5|E9B6T5_LEIMU Uncharacterized protein OS=Leishmania mexicana (s	2
3.160364683	-0.84388	E9ALU9	tr|E9ALU9|E9ALU9_LEIMU Uncharacterized protein OS=Leishmania mexicana (	7
3.177107418	-0.84412	E9ARU2;Q6	tr|E9ARU2|E9ARU2_LEIMU Nonspecific nucleoside hydrolase OS=Leishmania m	2
3.114915809	-0.84692	E9AUS9	tr|E9AUS9|E9AUS9_LEIMU Putative axoneme central apparatus protein OS=Lei	8
2.791853704	-0.84711	E9B422	tr|E9B422|E9B422_LEIMU Putative ABC transporter OS=Leishmania mexicana (	3
2.373935716	-0.8479	E9AVX8	tr|E9AVX8|E9AVX8_LEIMU I/6 autoantigen-like protein OS=Leishmania mexicar	5
4.242867557	-0.84949	E9B4A1	tr|E9B4A1|E9B4A1_LEIMU Mitochondrial processing peptidase alpha subunit,p	2
3.592793146	-0.85036	E9AVE2	tr|E9AVE2|E9AVE2_LEIMU Uncharacterized protein OS=Leishmania mexicana (	7
2.778003583	-0.85125	E9ATM9	tr|E9ATM9|E9ATM9_LEIMU Putative mitochondrial intermediate peptidase OS	6
3.035090015	-0.85339	E9AR04	tr|E9AR04|E9AR04_LEIMU Uncharacterized protein OS=Leishmania mexicana (	3
3.236433071	-0.85434	E9AJS7;E9A	tr|E9AJS7|E9AJS7_LEIMU Putative 60S acidic ribosomal protein P2 OS=Leishma	6
3.33890329	-0.85496	E9ALV6	tr|E9ALV6|E9ALV6_LEIMU Uncharacterized protein OS=Leishmania mexicana (s	3
3.057674631	-0.85549	E9B705	tr|E9B705|E9B705_LEIMU Putative peptidyl-prolyl cis-trans isomerase (Cycloph	4
3.421553961	-0.85574	E9B1W4;E9	tr|E9B1W4|E9B1W4_LEIMU Thiolase protein-like protein OS=Leishmania mexic	7
3.1028291	-0.85777	E9ARQ8;E9A	tr|E9ARQ8|E9ARQ8_LEIMU Putative prolyl-tRNA synthetase OS=Leishmania me	7
2.95156582	-0.8593	E9B5S5	tr|E9B5S5|E9B5S5_LEIMU Putative ATP-dependent DEAD-box RNA helicase OS:	9
3.143002737	-0.86231	E9ATM0	tr|E9ATM0|E9ATM0_LEIMU Proteasome regulatory ATPase subunit OS=Leishm	3
3.691788674	-0.86555	E9B466	tr|E9B466|E9B466_LEIMU Uncharacterized protein OS=Leishmania mexicana (s	3
2.679647354	-0.86566	E9AU12	tr|E9AU12|E9AU12_LEIMU tRNA (adenine(58)-N(1))-methyltransferase non-cat	2
3.262070336	-0.86578	E9AW84	tr|E9AW84|E9AW84_LEIMU 3-ketoacyl-CoA thiolase-like protein OS=Leishman	6
3.508953738	-0.86723	E9AK58	tr|E9AK58|E9AK58_LEIMU Putative 60S ribosomal protein L10 OS=Leishmania i	5
3.028774689	-0.86755	E9AUC7	tr|E9AUC7|E9AUC7_LEIMU Putative T-complex protein 1, theta subunit OS=Lei:	7
3.043243807	-0.86984	E9AKJ5	tr|E9AKJ5|E9AKJ5_LEIMU Uncharacterized protein OS=Leishmania mexicana (st	2
3.3024831	-0.86986	E9AW77	tr|E9AW77|E9AW77_LEIMU Uncharacterized protein OS=Leishmania mexicana	2
3.806200723	-0.86995	E9AJN9	tr|E9AJN9|E9AJN9_LEIMU D-3-phosphoglycerate dehydrogenase OS=Leishman	3
3.365083432	-0.87152	Q71PB4;E9E	tr|Q71PB4|Q71PB4_LEIME Broad specificity aminotransferase (Fragment) OS=L	6
2.804657374	-0.87257	E9AK97	tr|E9AK97|E9AK97_LEIMU Putative fructose-1,6-bisphosphatase, cytosolic OS=	2
3.51275574	-0.87313	E9AQ99;E9E	tr|E9AQ99|E9AQ99_LEIMU 60S ribosomal protein L6 OS=Leishmania mexicana	4
2.823431743	-0.87542	E9AQS9	tr|E9AQS9|E9AQS9_LEIMU Coatomer subunit delta OS=Leishmania mexicana (s	5
3.493887141	-0.87582	E9AXL3	tr|E9AXL3|E9AXL3_LEIMU Uncharacterized protein OS=Leishmania mexicana (s	2
2.730072383	-0.87855	E9B0G6	tr|E9B0G6|E9B0G6_LEIMU WD-40 repeat protein OS=Leishmania mexicana (str	2
3.373373457	-0.88037	E9ARI2	tr|E9ARI2|E9ARI2_LEIMU Carboxypeptidase OS=Leishmania mexicana (strain M	2
3.238225828	-0.88472	E9B0D7	tr|E9B0D7|E9B0D7_LEIMU Alkyl dihydroxyacetonephosphate synthase OS=Leis	4
3.349846025	-0.88544	E9B1B3	tr|E9B1B3|E9B1B3_LEIMU Phosphoglycerate kinase OS=Leishmania mexicana (	4
2.134170981	-0.88597	E9AWD4	tr|E9AWD4|E9AWD4_LEIMU Putative coronin OS=Leishmania mexicana (strain	2
3.424356292	-0.88944	E9B2N1	tr|E9B2N1|E9B2N1_LEIMU Putative dynein OS=Leishmania mexicana (strain M	6
3.190282269	-0.89035	E9AVA9	tr|E9AVA9|E9AVA9_LEIMU Putative adenylate kinase OS=Leishmania mexicana	4
2.370287399	-0.89198	E9B2P1	tr|E9B2P1|E9B2P1_LEIMU Elongation of fatty acids protein OS=Leishmania me:	2
3.018035205	-0.89229	E9AP13	tr|E9AP13|E9AP13_LEIMU NADH:flavin oxidoreductase/NADH oxidase,putative	7

2.590595778	-0.89323	Q86PL7;E9A	tr|Q86PL7|Q86PL7_LEIME Putative small GTP-binding protein OS=Leishmania n	4
3.304611872	-0.89528	Q95NJ3;E8N	tr|Q95NJ3|Q95NJ3_LEIAM Activated C kinase protein OS=Leishmania amazone	11
2.849892963	-0.89707	E9ALD1	tr|E9ALD1|E9ALD1_LEIMU Proteasome regulatory non-ATP-ase subunit,putativ	2
2.72868711	-0.89857	E9ATU8	tr|E9ATU8|E9ATU8_LEIMU Uncharacterized protein OS=Leishmania mexicana (	2
3.338298294	-0.89862	E9ARF2	tr|E9ARF2|E9ARF2_LEIMU Serine/threonine protein phosphatase type 5,putati	3
3.751666817	-0.90047	E8NHI0	tr|E8NHI0|E8NHI0_LEIMU 40S ribosomal protein S3a OS=Leishmania mexicana	9
3.504812424	-0.90197	E9AZV4	tr|E9AZV4|E9AZV4_LEIMU Haloacid dehalogenase-like hydrolase-like protein O	2
3.548165627	-0.9027	E9AV93	tr|E9AV93|E9AV93_LEIMU T-complex protein 1 subunit delta OS=Leishmania m	14
3.968011574	-0.90367	V5RIA5;E9A	tr|V5RIA5|V5RIA5_LEIAM Calmodulin-like protein OS=Leishmania amazonensis	8
3.042399584	-0.90446	E9B3G5	tr|E9B3G5|E9B3G5_LEIMU Putative myosin heavy chain OS=Leishmania mexica	6
3.268641779	-0.90524	E9ARW6	tr|E9ARW6|E9ARW6_LEIMU Uncharacterized protein OS=Leishmania mexicana	3
3.529329671	-0.90532	E9AQL8;Q9I	tr|E9AQL8|E9AQL8_LEIMU Putative carbamoyl-phosphate synthase OS=Leishm	26
2.941353717	-0.90589	E9B4H4;Q8I	tr|E9B4H4|E9B4H4_LEIMU Glucose-6-phosphate 1-dehydrogenase OS=Leishma	3
3.719224335	-0.90632	E9APR4;E9A	tr|E9APR4|E9APR4_LEIMU Elongation of fatty acids protein OS=Leishmania me	2
4.135814176	-0.91119	E9AM00	tr|E9AM00|E9AM00_LEIMU Putative U-box domain protein OS=Leishmania me	3
3.09657941	-0.91192	E9AU74	tr|E9AU74|E9AU74_LEIMU Uncharacterized protein OS=Leishmania mexicana (	2
3.364003224	-0.91225	E9B1F7	tr|E9B1F7|E9B1F7_LEIMU Uncharacterized protein OS=Leishmania mexicana (s	4
2.750587284	-0.91368	E9ATI7	tr|E9ATI7|E9ATI7_LEIMU Uncharacterized protein OS=Leishmania mexicana (st	6
2.939300053	-0.9147	E9AJN3	tr|E9AJN3|E9AJN3_LEIMU Putative ATP-dependent Clp protease subunit, heat s	5
3.691795838	-0.91517	E9AZ73	tr|E9AZ73|E9AZ73_LEIMU Putative eukaryotic translation release factor OS=Lei	10
2.901030849	-0.91562	E9B584	tr|E9B584|E9B584_LEIMU Uncharacterized protein OS=Leishmania mexicana (s	3
4.151347632	-0.92194	E9ATN5;E9A	tr|E9ATN5|E9ATN5_LEIMU Putative 60S ribosomal protein L18 OS=Leishmania	3
3.05079118	-0.92199	E9AWH3	tr|E9AWH3|E9AWH3_LEIMU Uncharacterized protein OS=Leishmania mexicana	2
3.702740494	-0.92325	E9ANB8	tr|E9ANB8|E9ANB8_LEIMU Uncharacterized protein OS=Leishmania mexicana (	5
2.989584698	-0.92399	E9ATU0	tr|E9ATU0|E9ATU0_LEIMU Uncharacterized protein OS=Leishmania mexicana (	2
3.60141388	-0.92434	E9AZR7	tr|E9AZR7|E9AZR7_LEIMU Putative ribosomal protein S20 OS=Leishmania mexi	3
2.857571628	-0.92472	E9AQX3	tr|E9AQX3|E9AQX3_LEIMU Uncharacterized protein OS=Leishmania mexicana (	4
2.961644049	-0.92571	E9AJN6	tr|E9AJN6|E9AJN6_LEIMU Putative peptidyl dipeptidase OS=Leishmania mexica	4
3.210250697	-0.92601	E9AJQ8	tr|E9AJQ8|E9AJQ8_LEIMU Putative long chain fatty Acyl CoA synthetase OS=Le	3
3.544067558	-0.92707	E9AUB1	tr|E9AUB1|E9AUB1_LEIMU Uncharacterized protein OS=Leishmania mexicana (	7
3.268216352	-0.92773	E9AJV2	tr|E9AJV2|E9AJV2_LEIMU Uncharacterized protein OS=Leishmania mexicana (s	12
3.121840783	-0.92821	E9AMD5	tr|E9AMD5|E9AMD5_LEIMU Translation initiation factor-like protein OS=Leishr	3
3.451974193	-0.92831	E9AWK3	tr|E9AWK3|E9AWK3_LEIMU Ankyrin/TPR repeat protein OS=Leishmania mexica	4
3.403088258	-0.92997	E9AQ24	tr|E9AQ24|E9AQ24_LEIMU Lysine--tRNA ligase OS=Leishmania mexicana (strain	9
3.345778068	-0.93419	E9ATN0	tr|E9ATN0|E9ATN0_LEIMU Uncharacterized protein OS=Leishmania mexicana (	3
3.557186738	-0.93437	E9APU4	tr|E9APU4|E9APU4_LEIMU ADP/ATP mitochondrial carrier-like protein OS=Leis	3
3.619157585	-0.93489	E9B2Z5	tr|E9B2Z5|E9B2Z5_LEIMU Uncharacterized protein OS=Leishmania mexicana (s	9
3.511043581	-0.93494	E9B3B4	tr|E9B3B4|E9B3B4_LEIMU Dihydrolipoyl dehydrogenase OS=Leishmania mexica	11
3.076540455	-0.93641	E9ATV9	tr|E9ATV9|E9ATV9_LEIMU Uncharacterized protein OS=Leishmania mexicana (s	2
2.8225642	-0.93783	E9B694	tr|E9B694|E9B694_LEIMU Uncharacterized protein OS=Leishmania mexicana (s	4
3.160404726	-0.9416	E9B2L9	tr|E9B2L9|E9B2L9_LEIMU Uncharacterized protein OS=Leishmania mexicana (s	10
3.471285753	-0.94263	E9B0V2	tr|E9B0V2|E9B0V2_LEIMU Putative queuine tRNA-ribosyltransferase OS=Leishr	3
3.077134579	-0.94395	E9AXG0	tr|E9AXG0|E9AXG0_LEIMU Prefoldin subunit 4 OS=Leishmania mexicana (strain	3
3.166927342	-0.9443	E9AJD6	tr|E9AJD6|E9AJD6_LEIMU Putative long-chain-fatty-acid-CoA ligase OS=Leishm	4
2.926331248	-0.94908	E9ASZ2	tr|E9ASZ2|E9ASZ2_LEIMU Putative COP-coated vesicle membrane protein erv2	2
3.273045502	-0.95039	E9AYF0	tr|E9AYF0|E9AYF0_LEIMU Putative cytochrome c oxidase subunit V OS=Leishm	3
2.847204698	-0.95232	E9AJY0	tr|E9AJY0|E9AJY0_LEIMU Uncharacterized protein OS=Leishmania mexicana (st	2
3.413591976	-0.95234	E9AXJ6;E9A	tr|E9AXJ6|E9AXJ6_LEIMU ATP synthase subunit beta OS=Leishmania mexicana	21
3.29516347	-0.95393	E9B1W8	tr|E9B1W8|E9B1W8_LEIMU Uncharacterized protein OS=Leishmania mexicana	2
3.538406898	-0.95417	E9AX01	tr|E9AX01|E9AX01_LEIMU Uncharacterized protein OS=Leishmania mexicana (s	9
3.187864878	-0.95439	E9ATU7	tr|E9ATU7|E9ATU7_LEIMU Uncharacterized protein OS=Leishmania mexicana (	4
3.454239765	-0.95448	E9AST4	tr|E9AST4|E9AST4_LEIMU Clathrin heavy chain OS=Leishmania mexicana (strain	29
3.965116735	-0.95719	E9APS5	tr|E9APS5|E9APS5_LEIMU Uncharacterized protein OS=Leishmania mexicana (s	3
3.592093868	-0.95773	E9AT13	tr|E9AT13|E9AT13_LEIMU Putative tyrosine aminotransferase OS=Leishmania r	9
3.453420337	-0.9579	E9AT36	tr|E9AT36|E9AT36_LEIMU Putative membrane-bound acid phosphatase 2 OS=L	7
3.34726664	-0.958	E9B2A8	tr|E9B2A8|E9B2A8_LEIMU Putative peptidase OS=Leishmania mexicana (strain	5
3.385323236	-0.95838	E9ALK7	tr|E9ALK7|E9ALK7_LEIMU Rab GDP dissociation inhibitor OS=Leishmania mexic	4
3.919303703	-0.95945	E9AXM1	tr|E9AXM1|E9AXM1_LEIMU GTP-binding nuclear protein OS=Leishmania mexic	6
3.203802945	-0.96051	E9B073	tr|E9B073|E9B073_LEIMU Putative acyl-CoA dehydrogenase OS=Leishmania me	7
4.011858442	-0.96152	E9B1H5	tr|E9B1H5|E9B1H5_LEIMU Putative ATP-dependent RNA helicase OS=Leishmar	4
3.400888201	-0.96328	E9B544	tr|E9B544|E9B544_LEIMU Uncharacterized protein OS=Leishmania mexicana (s	3
3.021561046	-0.96685	E9ASF3	tr|E9ASF3|E9ASF3_LEIMU ER membrane protein complex subunit 3 OS=Leishm	2
3.572358112	-0.9669	Q8MTW1;E9	tr|Q8MTW1|Q8MTW1_LEIAM Meta 2 protein OS=Leishmania amazonensis OX	3

3.577778811	-0.96829	E9B146	tr|E9B146|E9B146_LEIMU Conserved TPR domain protein OS=Leishmania mexi	8
3.572405083	-0.96881	E9B473	tr|E9B473|E9B473_LEIMU Succinyl-CoA:3-ketoacid-coenzyme A transferase OS	5
3.457233998	-0.96973	E9AU22	tr|E9AU22|E9AU22_LEIMU Uncharacterized protein OS=Leishmania mexicana (	6
3.979712284	-0.96975	E9ALU2	tr|E9ALU2|E9ALU2_LEIMU Putative carnitine/choline acetyltransferase OS=Leis	7
2.795584929	-0.97198	E9ALJ9	tr|E9ALJ9|E9ALJ9_LEIMU Aminopeptidase OS=Leishmania mexicana (strain MH	4
3.63666725	-0.97209	E9ARA1	tr|E9ARA1|E9ARA1_LEIMU Putative translation initiation factor OS=Leishmania	8
3.535782139	-0.97519	E9B183	tr|E9B183|E9B183_LEIMU Uncharacterized protein OS=Leishmania mexicana (s	9
3.168053071	-0.97542	E9B0I1	tr|E9B0I1|E9B0I1_LEIMU Uncharacterized protein OS=Leishmania mexicana (st	3
3.955560257	-0.97903	E9AYK8	tr|E9AYK8|E9AYK8_LEIMU Putative nitrilase OS=Leishmania mexicana (strain M	2
3.487427008	-0.98274	E9B6N5	tr|E9B6N5|E9B6N5_LEIMU Uncharacterized protein OS=Leishmania mexicana (	3
3.232207588	-0.98326	E9AXK0	tr|E9AXK0|E9AXK0_LEIMU Uncharacterized protein OS=Leishmania mexicana (s	3
3.785060118	-0.98665	E9AVG6	tr|E9AVG6|E9AVG6_LEIMU Proteasome subunit alpha type OS=Leishmania me:	4
3.464590906	-0.9889	E9AYB7	tr|E9AYB7|E9AYB7_LEIMU Prefoldin subunit 3 OS=Leishmania mexicana (strain	2
3.39171761	-0.99264	E9AMV6	tr|E9AMV6|E9AMV6_LEIMU Phospholipid:diacylglycerol acyltransferase,putati	5
2.818002818	-0.99369	E9AK40;A0A	tr|E9AK40|E9AK40_LEIMU Putative spermidine synthase OS=Leishmania mexic	2
3.958564008	-0.99569	E9AZX3	tr|E9AZX3|E9AZX3_LEIMU Putative DEAD box RNA helicase OS=Leishmania me:	2
3.009776356	-0.99581	E9B276	tr|E9B276|E9B276_LEIMU Uncharacterized protein OS=Leishmania mexicana (s	3
3.183974789	-0.9963	E9ANC1	tr|E9ANC1|E9ANC1_LEIMU Uncharacterized protein OS=Leishmania mexicana (	5
3.727862067	-0.99701	E9AQ01	tr|E9AQ01|E9AQ01_LEIMU Uncharacterized protein OS=Leishmania mexicana (	3
3.549176547	-0.99757	E9B476	tr|E9B476|E9B476_LEIMU Uncharacterized protein OS=Leishmania mexicana (s	4
2.937258144	-1.00051	E9AYM2	tr|E9AYM2|E9AYM2_LEIMU Uncharacterized protein OS=Leishmania mexicana	2
3.61774826	-1.00253	E9B187	tr|E9B187|E9B187_LEIMU Putative valyl-tRNA synthetase OS=Leishmania mexi	3
3.750685071	-1.00434	E9B5F8	tr|E9B5F8|E9B5F8_LEIMU Vacuolar ATP synthase catalytic subunit A,putative C	13
3.529083694	-1.00714	E9B5Z7;E9B	tr|E9B5Z7|E9B5Z7_LEIMU Putative NADH-dependent fumarate reductase OS=L	15
3.491633534	-1.00831	E9ASL3	tr|E9ASL3|E9ASL3_LEIMU Putative 40S ribosomal protein S18 OS=Leishmania n	8
3.563270617	-1.01079	E9AK51	tr|E9AK51|E9AK51_LEIMU Putative ubiquitin-conjugating enzyme e2 OS=Leishn	2
2.727808636	-1.01241	E9ASG7	tr|E9ASG7|E9ASG7_LEIMU Putative DNAJ domain protein OS=Leishmania mexi	2
3.855207768	-1.01403	E9AU41	tr|E9AU41|E9AU41_LEIMU Uncharacterized protein OS=Leishmania mexicana (	9
3.481139026	-1.01927	E9ATX3	tr|E9ATX3|E9ATX3_LEIMU Putative NADH dehydrogenase OS=Leishmania mexi	5
3.363973293	-1.01999	E9AUU5	tr|E9AUU5|E9AUU5_LEIMU Phosphatase-like protein OS=Leishmania mexicana	6
3.4388485	-1.02676	E9AW79	tr|E9AW79|E9AW79_LEIMU Uncharacterized protein OS=Leishmania mexicana	2
2.013182595	-1.02797	E9AZU7	tr|E9AZU7|E9AZU7_LEIMU Putative phenylalanine-4-hydroxylase OS=Leishman	2
4.18611656	-1.02845	E9AWN2	tr|E9AWN2|E9AWN2_LEIMU Ubiquitin carboxyl-terminal hydrolase OS=Leishm	2
3.87040637	-1.02847	E9ANM6	tr|E9ANM6|E9ANM6_LEIMU Uncharacterized protein OS=Leishmania mexicana	8
3.496917442	-1.03	E9ANB6	tr|E9ANB6|E9ANB6_LEIMU Uncharacterized protein OS=Leishmania mexicana (	2
3.914680574	-1.03019	E9ALZ2	tr|E9ALZ2|E9ALZ2_LEIMU High mobility group protein homolog tdp-1,putative	3
3.015402825	-1.03284	E9AP30	tr|E9AP30|E9AP30_LEIMU Uncharacterized protein OS=Leishmania mexicana (s	2
3.101708531	-1.03427	E9ASS2	tr|E9ASS2|E9ASS2_LEIMU Uncharacterized protein OS=Leishmania mexicana (s	4
3.890786407	-1.03493	E9B189	tr|E9B189|E9B189_LEIMU Uncharacterized protein OS=Leishmania mexicana (s	3
3.932815306	-1.03519	E9AW40	tr|E9AW40|E9AW40_LEIMU Putative NADP-dependent alcohol dehydrogenase	8
4.143957865	-1.03521	E9ARM0	tr|E9ARM0|E9ARM0_LEIMU Periodic tryptophan protein 2-like protein OS=Leis	2
3.571408576	-1.03568	E9AXQ3	tr|E9AXQ3|E9AXQ3_LEIMU Pyruvate dehydrogenase E1 component subunit be	8
3.655471109	-1.0362	E9AQ06	tr|E9AQ06|E9AQ06_LEIMU Heat shock protein HslVU, ATPase subunit HslU,put	3
3.690253787	-1.03817	E9B3G8	tr|E9B3G8|E9B3G8_LEIMU Putative 60S ribosomal protein L2 OS=Leishmania m	5
3.892677191	-1.04042	E9ARJ0	tr|E9ARJ0|E9ARJ0_LEIMU Uncharacterized protein OS=Leishmania mexicana (s	2
3.138641212	-1.04056	E9AMD0	tr|E9AMD0|E9AMD0_LEIMU Putative ribose-phosphate pyrophosphokinase OS	2
3.603440154	-1.04163	E9AKL7	tr|E9AKL7|E9AKL7_LEIMU Uncharacterized protein OS=Leishmania mexicana (s	2
4.201873927	-1.04179	E9B1E9	tr|E9B1E9|E9B1E9_LEIMU Putative 5-methyltetrahydropteroyltriglutamate--ho	4
3.742907919	-1.04238	E9ASW8;Q8	tr|E9ASW8|E9ASW8_LEIMU Putative nucleoside transporter 1 OS=Leishmania r	2
3.461430488	-1.04306	E9ARX5	tr|E9ARX5|E9ARX5_LEIMU Uncharacterized protein OS=Leishmania mexicana (	4
3.706949092	-1.04384	E9AZ37	tr|E9AZ37|E9AZ37_LEIMU Putative arginyl-tRNA synthetase OS=Leishmania me	19
3.390432137	-1.04451	E9B755	tr|E9B755|E9B755_LEIMU p21 antigen protein OS=Leishmania mexicana (strair	2
4.052930283	-1.04694	E9AZA7;E9A	tr|E9AZA7|E9AZA7_LEIMU Branched-chain-amino-acid aminotransferase OS=Le	6
3.51931464	-1.04849	E9B350;E9B	tr|E9B350|E9B350_LEIMU Putative ribosomal protein L27 OS=Leishmania mexi	6
3.277159157	-1.0494	E9AWR7	tr|E9AWR7|E9AWR7_LEIMU Malic enzyme OS=Leishmania mexicana (strain MH	7
3.402191843	-1.05012	E9AJJ4	tr|E9AJJ4|E9AJJ4_LEIMU Uncharacterized protein OS=Leishmania mexicana (str	7
3.327501188	-1.05104	E9AK26	tr|E9AK26|E9AK26_LEIMU Putative calpain-like cysteine peptidase OS=Leishma	2
3.705696213	-1.05113	E9ARL9	tr|E9ARL9|E9ARL9_LEIMU Uncharacterized protein OS=Leishmania mexicana (s	22
3.461294225	-1.05155	E9AQ39	tr|E9AQ39|E9AQ39_LEIMU Putative 60S acidic ribosomal protein OS=Leishman	2
3.982410778	-1.05263	E9AJV8	tr|E9AJV8|E9AJV8_LEIMU Uncharacterized protein OS=Leishmania mexicana (s	2
4.384735163	-1.05365	E9AYS6	tr|E9AYS6|E9AYS6_LEIMU Kinetoplast-associated protein-like protein OS=Leish	3
3.796174915	-1.05444	E9AU26	tr|E9AU26|E9AU26_LEIMU Uncharacterized protein OS=Leishmania mexicana (	3
3.628965545	-1.0568	E9AVZ3	tr|E9AVZ3|E9AVZ3_LEIMU Uncharacterized protein OS=Leishmania mexicana (s	2

3.303230999	-1.05752	E9B0P1	tr|E9B0P1|E9B0P1_LEIMU Importin subunit alpha OS=Leishmania mexicana (st	7
3.342026203	-1.06015	E9AV30	tr|E9AV30|E9AV30_LEIMU Putative phosphoglucomutase OS=Leishmania mexi	2
3.499892442	-1.06367	E9B687	tr|E9B687|E9B687_LEIMU Putative kinesin OS=Leishmania mexicana (strain MH	6
3.10507818	-1.06414	E9ANI1	tr|E9ANI1|E9ANI1_LEIMU Putative 14-3-3 protein OS=Leishmania mexicana (st	6
2.98123483	-1.06551	E9B2X2	tr|E9B2X2|E9B2X2_LEIMU Putative heat shock protein-like protein OS=Leishma	2
3.456495441	-1.06699	E9ATF7	tr|E9ATF7|E9ATF7_LEIMU Putative 40S ribosomal protein S27-1 OS=Leishmania	4
3.715439841	-1.0676	E9AMH0	tr|E9AMH0|E9AMH0_LEIMU Uncharacterized protein OS=Leishmania mexicana	3
3.61877565	-1.06772	E9ARK4	tr|E9ARK4|E9ARK4_LEIMU Uncharacterized protein OS=Leishmania mexicana (	6
3.49113934	-1.07014	E9AZE0	tr|E9AZE0|E9AZE0_LEIMU Obg-like ATPase 1 OS=Leishmania mexicana (strain M	4
3.386719267	-1.07354	E9APD4;A0A	tr|E9APD4|E9APD4_LEIMU Putative leucyl-tRNA synthetase OS=Leishmania me	5
2.964556971	-1.07421	E9AZK7	tr|E9AZK7|E9AZK7_LEIMU Uncharacterized protein OS=Leishmania mexicana (s	2
3.247146437	-1.07494	E9B343	tr|E9B343|E9B343_LEIMU Putative cystathionine beta-lyase OS=Leishmania me	3
3.542392183	-1.07897	E9AT02;A0A	tr|E9AT02|E9AT02_LEIMU Developmentally regulated GTP-binding protein 1,pu	7
4.868940803	-1.08055	E9ATC6	tr|E9ATC6|E9ATC6_LEIMU 2-oxoglutarate dehydrogenase E1 component,putat	6
3.830595979	-1.08195	E9ALX2	tr|E9ALX2|E9ALX2_LEIMU Uncharacterized protein OS=Leishmania mexicana (s	5
4.06702801	-1.0851	E9AV48	tr|E9AV48|E9AV48_LEIMU Uncharacterized protein OS=Leishmania mexicana (	15
3.203999899	-1.08727	E9AM02	tr|E9AM02|E9AM02_LEIMU Putative lipophosphoglycan biosynthetic protein C	9
4.056949533	-1.08821	E9AWI3	tr|E9AWI3|E9AWI3_LEIMU V-type proton ATPase subunit a OS=Leishmania me	3
4.071449505	-1.08968	E9AWB4	tr|E9AWB4|E9AWB4_LEIMU Uncharacterized protein OS=Leishmania mexicana	5
3.122671232	-1.09053	E9ASF4	tr|E9ASF4|E9ASF4_LEIMU Phosphatidylinositol-4-phosphate 5-kinase-like prote	2
3.289585207	-1.09832	E9AQW6	tr|E9AQW6|E9AQW6_LEIMU Putative endoplasmic reticulum oxidoreductin OS	2
3.664257185	-1.10455	E9APW3;Q3	tr|E9APW3|E9APW3_LEIMU Enolase OS=Leishmania mexicana (strain MHOM/(	17
3.386335124	-1.1049	E9AXC0	tr|E9AXC0|E9AXC0_LEIMU Uncharacterized protein OS=Leishmania mexicana (	5
3.801480869	-1.10685	E9AUL9	tr|E9AUL9|E9AUL9_LEIMU Putative vesicle-fusing ATPase OS=Leishmania mexi	6
3.40470862	-1.10714	E9AJD9	tr|E9AJD9|E9AJD9_LEIMU Putative long-chain-fatty-acid-CoA ligase OS=Leishm	6
3.779276353	-1.1076	E9AVY6	tr|E9AVY6|E9AVY6_LEIMU Alanine--tRNA ligase OS=Leishmania mexicana (strai	13
3.713769714	-1.10798	E9B713	tr|E9B713|E9B713_LEIMU Proteasome subunit alpha type OS=Leishmania mex	5
4.107362501	-1.10877	E9APZ6	tr|E9APZ6|E9APZ6_LEIMU Putative glutathione-S-transferase/glutaredoxin OS=	4
3.799023904	-1.11051	E9B297	tr|E9B297|E9B297_LEIMU Putative acetyl-CoA carboxylase OS=Leishmania mex	27
3.525441875	-1.11197	E9AT43	tr|E9AT43|E9AT43_LEIMU Dihydrolipoamide acetyltransferase component of p	9
3.182108133	-1.1142	E9ALX8	tr|E9ALX8|E9ALX8_LEIMU Uncharacterized protein OS=Leishmania mexicana (s	2
3.609555068	-1.11607	E9APH2	tr|E9APH2|E9APH2_LEIMU Putative Ran-binding protein 1 OS=Leishmania mex	2
3.564625425	-1.11718	E9AWE0	tr|E9AWE0|E9AWE0_LEIMU T-complex protein 1 subunit gamma OS=Leishman	14
4.259858866	-1.11923	E9AM16	tr|E9AM16|E9AM16_LEIMU Putative ABC transporter OS=Leishmania mexicana	6
3.554326064	-1.11983	E9B578;E9B	tr|E9B578|E9B578_LEIMU Putative ribosomal protein L3 OS=Leishmania mexic	9
4.1573128	-1.12368	E9B4H8	tr|E9B4H8|E9B4H8_LEIMU Putative adenylate kinase OS=Leishmania mexicana	4
3.508131685	-1.13183	Q25319;E9A	tr|Q25319|Q25319_LEIME Cathepsin B-like enzyme OS=Leishmania mexicana (	3
3.331893641	-1.13519	E9B215	tr|E9B215|E9B215_LEIMU Prostaglandin f2-alpha synthase OS=Leishmania mex	6
3.606521756	-1.13569	E9B1X5	tr|E9B1X5|E9B1X5_LEIMU Nucleosome assembly protein-like protein OS=Leish	6
2.935362956	-1.13633	E9B733	tr|E9B733|E9B733_LEIMU Uncharacterized protein OS=Leishmania mexicana (s	2
3.994409109	-1.1365	E9AXP2	tr|E9AXP2|E9AXP2_LEIMU Uncharacterized protein OS=Leishmania mexicana (	5
3.04770704	-1.13707	E9AS55	tr|E9AS55|E9AS55_LEIMU Putative phenylalanyl-tRNA synthetase OS=Leishma	2
3.628401677	-1.13804	E9AN99	tr|E9AN99|E9AN99_LEIMU Peptidylprolyl isomerase OS=Leishmania mexicana	2
2.217274808	-1.14029	E9AK63	tr|E9AK63|E9AK63_LEIMU Uncharacterized protein OS=Leishmania mexicana (	2
3.708145963	-1.14065	E9B6R4	tr|E9B6R4|E9B6R4_LEIMU T-complex protein 1 subunit eta OS=Leishmania me	11
3.21767906	-1.14197	E9AN31	tr|E9AN31|E9AN31_LEIMU Uncharacterized protein OS=Leishmania mexicana (	2
3.36716128	-1.14251	E9AZS3	tr|E9AZS3|E9AZS3_LEIMU Uncharacterized protein OS=Leishmania mexicana (s	4
3.679857192	-1.14478	E9B400	tr|E9B400|E9B400_LEIMU Peptidyl-prolyl cis-trans isomerase OS=Leishmania m	4
2.997514723	-1.14591	E9B6Q7	tr|E9B6Q7|E9B6Q7_LEIMU Putative 60S ribosomal protein L23 OS=Leishmania	4
3.49579952	-1.14619	E9AVS5;E9A	tr|E9AVS5|E9AVS5_LEIMU Uncharacterized protein OS=Leishmania mexicana (	2
3.527929595	-1.14835	E9APT8	tr|E9APT8|E9APT8_LEIMU Putative immunodominant antigen (Tc40 antigen-lik	7
3.876715408	-1.15018	E9ANP7	tr|E9ANP7|E9ANP7_LEIMU Uncharacterized protein OS=Leishmania mexicana (	6
4.112692685	-1.15486	O76285;E9A	tr|O76285|O76285_LEIAM Ribonucleotide reductase M2 subunit OS=Leishman	7
3.250100565	-1.15489	E9AR70	tr|E9AR70|E9AR70_LEIMU Uncharacterized protein OS=Leishmania mexicana (	4
2.513548199	-1.15536	E9ARF8	tr|E9ARF8|E9ARF8_LEIMU Uncharacterized protein OS=Leishmania mexicana (s	3
3.230108821	-1.15701	Q0QW09;E9	tr|Q0QW09|Q0QW09_LEIME Malate dehydrogenase OS=Leishmania mexicana	2
4.068462942	-1.15834	E9ANA1	tr|E9ANA1|E9ANA1_LEIMU Putative small GTP-binding protein Rab11 OS=Leish	4
3.175967319	-1.16078	E9AWJ4	tr|E9AWJ4|E9AWJ4_LEIMU Putative 60S ribosomal protein L17 OS=Leishmania	4
3.849268312	-1.16176	E9B6U3	tr|E9B6U3|E9B6U3_LEIMU Polyadenylate-binding protein OS=Leishmania mexi	15
2.756193399	-1.16366	E9B3U1	tr|E9B3U1|E9B3U1_LEIMU Uncharacterized protein OS=Leishmania mexicana (	2
3.836477571	-1.16427	E9AUG4	tr|E9AUG4|E9AUG4_LEIMU Uncharacterized protein OS=Leishmania mexicana	2
3.653941316	-1.16469	P16126;E9A	sp|P16126|DRTS_LEIAM Bifunctional dihydrofolate reductase-thymidylate synt	6
3.201462831	-1.16726	Q25318;E8N	tr|Q25318|Q25318_LEIME LmCPb19 (Fragment) OS=Leishmania mexicana OX=	3

3.208479934	-1.16803	E9AS32	tr|E9AS32|E9AS32_LEIMU Putative ABC transporter OS=Leishmania mexicana (	2
3.870524323	-1.16974	E9B0A4	tr|E9B0A4|E9B0A4_LEIMU Putative heat shock protein 70 OS=Leishmania mexi	3
3.912986381	-1.17286	E9AVH9	tr|E9AVH9|E9AVH9_LEIMU Proteasome subunit alpha type OS=Leishmania me:	4
4.33845807	-1.17327	E9B5Q7	tr|E9B5Q7|E9B5Q7_LEIMU Uncharacterized protein OS=Leishmania mexicana (	2
4.142818058	-1.17611	E9APG0	tr|E9APG0|E9APG0_LEIMU Uncharacterized protein OS=Leishmania mexicana (	12
4.083054983	-1.17798	E9AQY9	tr|E9AQY9|E9AQY9_LEIMU Putative peptidase t (Peptidase t, putative, metallo-	4
3.775678432	-1.18167	E9ALD8;E9B	tr|E9ALD8|E9ALD8_LEIMU 40S ribosomal protein S19-like protein OS=Leishmar	4
3.20441203	-1.18332	E9AZE8	tr|E9AZE8|E9AZE8_LEIMU Putative TPR-repeat protein OS=Leishmania mexicar	3
4.071001735	-1.18414	E9ANK8;E9/	tr|E9ANK8|E9ANK8_LEIMU Putative aminopeptidase (Metallo-peptidase, clan r	5
3.662475176	-1.18613	E9AW01	tr|E9AW01|E9AW01_LEIMU Uncharacterized protein OS=Leishmania mexicana	4
3.754671878	-1.18958	E9API2	tr|E9API2|E9API2_LEIMU Ubiquitin-conjugating enzyme-like protein OS=Leishm	3
4.078401722	-1.19094	E9ALA2	tr|E9ALA2|E9ALA2_LEIMU Putative ubiquitin-conjugating enzyme e2 (Putative	2
3.115726183	-1.19326	E9AM67	tr|E9AM67|E9AM67_LEIMU Proteasome regulatory non-ATPase subunit,putati	8
3.046112434	-1.19603	E9APY0	tr|E9APY0|E9APY0_LEIMU Serine hydroxymethyltransferase OS=Leishmania me	2
3.658288343	-1.19685	Q9GU60;E9	tr|Q9GU60|Q9GU60_LEIAM Kinetoplast membrane protein 11 OS=Leishmania	4
4.262391184	-1.20156	E9B2G0	tr|E9B2G0|E9B2G0_LEIMU Proteasome regulatory non-ATP-ase subunit 8,puta	4
3.995297872	-1.202	E9AZ29	tr|E9AZ29|E9AZ29_LEIMU Putative T-complex protein 1, beta subunit OS=Leish	9
3.232597518	-1.20597	E9B430	tr|E9B430|E9B430_LEIMU Putative phosphoribosylpyrophosphate synthetase (	2
4.339269374	-1.20736	E9B6J0	tr|E9B6J0|E9B6J0_LEIMU Uncharacterized protein OS=Leishmania mexicana (st	3
3.738727466	-1.20759	E9AMI1	tr|E9AMI1|E9AMI1_LEIMU Stress-induced protein sti1 OS=Leishmania mexican	7
3.869450671	-1.21159	E9B0J8	tr|E9B0J8|E9B0J8_LEIMU GrpE protein homolog OS=Leishmania mexicana (stra	6
4.485869819	-1.21954	E9AV05	tr|E9AV05|E9AV05_LEIMU Uncharacterized protein OS=Leishmania mexicana (	2
3.534347814	-1.22753	E9AJK0	tr|E9AJK0|E9AJK0_LEIMU Proteasome regulatory non-ATPase subunit 6,putativ	5
3.237571577	-1.22884	Q7Z031;E9A	tr|Q7Z031|Q7Z031_LEIAM Acidocalcisomal pyrophosphatase OS=Leishmania ai	3
3.870530563	-1.22931	E9ASM3	tr|E9ASM3|E9ASM3_LEIMU Uncharacterized protein OS=Leishmania mexicana	2
3.35626686	-1.23065	E9ATH0	tr|E9ATH0|E9ATH0_LEIMU Eukaryotic translation initiation factor 3 subunit I O:	6
3.905378894	-1.23193	Q5QHV5;E9	tr|Q5QHV5|Q5QHV5_LEIAM Rbp38p OS=Leishmania amazonensis OX=5659 PE	5
3.699268868	-1.23197	E9ALY7	tr|E9ALY7|E9ALY7_LEIMU Uncharacterized protein OS=Leishmania mexicana (s	3
3.639921181	-1.23383	E9ATR2	tr|E9ATR2|E9ATR2_LEIMU Uncharacterized protein OS=Leishmania mexicana (:	2
4.478035991	-1.23532	E9B6X2	tr|E9B6X2|E9B6X2_LEIMU Putative mitochondrial phosphate transporter OS=Lı	5
3.371229352	-1.23805	E9ALW3	tr|E9ALW3|E9ALW3_LEIMU Uncharacterized protein OS=Leishmania mexicana	3
3.866464041	-1.23893	E9AV49	tr|E9AV49|E9AV49_LEIMU Putative methionyl-tRNA synthetase OS=Leishmania	3
3.926417093	-1.24161	E9ARZ1	tr|E9ARZ1|E9ARZ1_LEIMU Putative 40S ribosomal protein S13 OS=Leishmania ı	4
3.560997187	-1.24593	Q9BLW4;E9	tr|Q9BLW4|Q9BLW4_LEIME GDP-mannose pyrophosphorylase OS=Leishmania	3
4.527564113	-1.2463	Q95ZD7;E9/	tr|Q95ZD7|Q95ZD7_LEIME Phosphomannomutase OS=Leishmania mexicana O:	4
4.272262353	-1.24706	E9ALE8	tr|E9ALE8|E9ALE8_LEIMU Uncharacterized protein OS=Leishmania mexicana (s	2
3.868706441	-1.24833	E9B228	tr|E9B228|E9B228_LEIMU Uncharacterized protein OS=Leishmania mexicana (s	2
3.918068118	-1.24936	E9ATT5;E9A	tr|E9ATT5|E9ATT5_LEIMU 40S ribosomal protein SA OS=Leishmania mexicana (	8
3.75528803	-1.25163	E9B3B0	tr|E9B3B0|E9B3B0_LEIMU Putative chaperonin alpha subunit OS=Leishmania m	11
3.294222872	-1.2553	E9APB1	tr|E9APB1|E9APB1_LEIMU Mitochondrial processing peptidase alpha subunit,p	3
3.76140115	-1.25576	E9ATA6	tr|E9ATA6|E9ATA6_LEIMU Putative 60S ribosomal protein L22 OS=Leishmania ı	3
3.925559401	-1.26101	E9AM03	tr|E9AM03|E9AM03_LEIMU Putative U3 small nucleolar ribonucleoprotein pro	3
3.562573571	-1.26455	E9B110	tr|E9B110|E9B110_LEIMU ADP-ribosylation factor-like protein OS=Leishmania ı	3
3.964633525	-1.26757	E9AL77	tr|E9AL77|E9AL77_LEIMU Uncharacterized protein OS=Leishmania mexicana (s	3
4.081421532	-1.2697	E9AWH9	tr|E9AWH9|E9AWH9_LEIMU Uncharacterized protein OS=Leishmania mexicana	3
3.90945519	-1.27069	E9ASQ6	tr|E9ASQ6|E9ASQ6_LEIMU Transitional endoplasmic reticulum ATPase,putative	14
3.835376396	-1.2741	E9B5M9	tr|E9B5M9|E9B5M9_LEIMU Proteasome subunit beta OS=Leishmania mexicana	2
4.089637153	-1.28373	E9B6Z8	tr|E9B6Z8|E9B6Z8_LEIMU Uncharacterized protein OS=Leishmania mexicana (s	2
4.099214945	-1.28603	E9B5D9	tr|E9B5D9|E9B5D9_LEIMU RuvB-like helicase OS=Leishmania mexicana (strain	4
3.960556994	-1.28814	E9B3W9	tr|E9B3W9|E9B3W9_LEIMU Cysteine conjugate beta-lyase,aminotransferase-li	7
3.947017338	-1.28923	E9B4N4	tr|E9B4N4|E9B4N4_LEIMU Proteasome regulatory non-ATP-ase subunit 11,put	2
5.24809209	-1.28999	E9AK60;E9A	tr|E9AK60|E9AK60_LEIMU Uncharacterized protein OS=Leishmania mexicana (:	3
4.234343271	-1.29284	E9AVJ3	tr|E9AVJ3|E9AVJ3_LEIMU Putative heat shock protein DNAJ OS=Leishmania me	2
3.494803774	-1.29314	E9B001;Q5I	tr|E9B001|E9B001_LEIMU Replication protein A subunit OS=Leishmania mexica	7
4.061064701	-1.29348	E8NHJ5;E9A	tr|E8NHJ5|E8NHJ5_LEIMU WGS CADB00000000 data, contig 43 OS=Leishmania	5
3.588941047	-1.2966	E9AN84	tr|E9AN84|E9AN84_LEIMU Uncharacterized protein OS=Leishmania mexicana (	2
3.799766945	-1.29792	E9AZQ5	tr|E9AZQ5|E9AZQ5_LEIMU Ribonucleoside-diphosphate reductase OS=Leishma	3
3.262120875	-1.30034	E9AW05	tr|E9AW05|E9AW05_LEIMU Peptidyl-prolyl cis-trans isomerase OS=Leishmania	3
5.08274601	-1.30178	E9B2Q1	tr|E9B2Q1|E9B2Q1_LEIMU Uncharacterized protein OS=Leishmania mexicana (	2
3.084294424	-1.31503	E9AQ80	tr|E9AQ80|E9AQ80_LEIMU Uncharacterized protein OS=Leishmania mexicana (	2
4.881682014	-1.32179	E9AXI7	tr|E9AXI7|E9AXI7_LEIMU Uncharacterized protein OS=Leishmania mexicana (st	2
3.773948626	-1.32445	E9B6B5	tr|E9B6B5|E9B6B5_LEIMU Putative aminopeptidase P OS=Leishmania mexicana	3
3.718820844	-1.32464	E9AQE0	tr|E9AQE0|E9AQE0_LEIMU Proliferating cell nuclear antigen OS=Leishmania me	5

3.868458525	-1.33018	E9B5M6	tr|E9B5M6|E9B5M6_LEIMU Coatomer subunit alpha OS=Leishmania mexicana	8
3.607560899	-1.33215	E9AKK2	tr|E9AKK2|E9AKK2_LEIMU Dipeptidyl peptidase 3 OS=Leishmania mexicana (str	11
3.459012413	-1.33275	E9B2C5	tr|E9B2C5|E9B2C5_LEIMU Uncharacterized protein OS=Leishmania mexicana (s	3
2.992868611	-1.33377	E9AM51	tr|E9AM51|E9AM51_LEIMU Uncharacterized protein OS=Leishmania mexicana	2
2.980523204	-1.33775	E9B2R2	tr|E9B2R2|E9B2R2_LEIMU Uncharacterized protein OS=Leishmania mexicana (s	3
3.911236204	-1.33974	E9ASD6	tr|E9ASD6|E9ASD6_LEIMU Elongation factor 2 OS=Leishmania mexicana (strain	31
4.338912657	-1.34343	E9AZN6	tr|E9AZN6|E9AZN6_LEIMU Uncharacterized protein OS=Leishmania mexicana (	2
4.30923025	-1.3479	E9AZN0	tr|E9AZN0|E9AZN0_LEIMU Serine/threonine-protein phosphatase OS=Leishma	2
4.066817693	-1.35372	E9B4G0	tr|E9B4G0|E9B4G0_LEIMU Coatomer subunit beta OS=Leishmania mexicana (s	4
3.487871616	-1.35916	E9AVP4	tr|E9AVP4|E9AVP4_LEIMU Putative proteasome regulatory ATPase subunit 5 O	6
3.358743008	-1.35949	E9APP0	tr|E9APP0|E9APP0_LEIMU Uncharacterized protein OS=Leishmania mexicana (s	5
3.684131842	-1.36809	E9ARC2	tr|E9ARC2|E9ARC2_LEIMU Uncharacterized protein OS=Leishmania mexicana (	12
4.065523885	-1.36964	E9APQ1	tr|E9APQ1|E9APQ1_LEIMU Uncharacterized protein OS=Leishmania mexicana (	2
4.331747372	-1.37065	E8NHJ8;E8N	tr|E8NHJ8|E8NHJ8_LEIMU 60S acidic ribosomal protein P0 OS=Leishmania mex	7
3.883168115	-1.37148	E9AW86;E9.	tr|E9AW86|E9AW86_LEIMU Acetyl-coenzyme A synthetase OS=Leishmania me	12
4.000227034	-1.38074	E9AST1	tr|E9AST1|E9AST1_LEIMU Proteasome subunit alpha type OS=Leishmania mexi	4
3.545972088	-1.38296	E9ATG0	tr|E9ATG0|E9ATG0_LEIMU Uncharacterized protein OS=Leishmania mexicana (	2
3.748757394	-1.38733	E9ANS8	tr|E9ANS8|E9ANS8_LEIMU Proteasome subunit beta OS=Leishmania mexicana	4
4.264179554	-1.38877	E9APT0	tr|E9APT0|E9APT0_LEIMU Putative calpain-like cysteine peptidase (Putative cy	4
4.155956572	-1.39616	E9AYS1	tr|E9AYS1|E9AYS1_LEIMU Proteasome endopeptidase complex OS=Leishmania	3
4.449784424	-1.39902	E9AL33	tr|E9AL33|E9AL33_LEIMU Fe-S cluster assembly protein DRE2 OS=Leishmania r	2
3.86341608	-1.39923	E9ANW2	tr|E9ANW2|E9ANW2_LEIMU Uncharacterized protein OS=Leishmania mexican	2
3.739918128	-1.40352	E9AS20;E9A	tr|E9AS20|E9AS20_LEIMU Putative kinesin OS=Leishmania mexicana (strain MH	3
4.291936077	-1.40869	E9AXF0	sp|E9AXF0|IF5A_LEIMU Eukaryotic translation initiation factor 5A OS=Leishmar	6
3.65097177	-1.41293	E9B4B4	tr|E9B4B4|E9B4B4_LEIMU Putative translation initiation factor IF-2 OS=Leishm	3
4.587904969	-1.41859	E9B5G5	tr|E9B5G5|E9B5G5_LEIMU Serine palmitoyltransferase-like protein OS=Leishm	2
3.777972675	-1.42121	E9AKN1;Q6	tr|E9AKN1|E9AKN1_LEIMU Uncharacterized protein OS=Leishmania mexicana (	9
3.971390801	-1.42456	E9AJK8	tr|E9AJK8|E9AJK8_LEIMU Uncharacterized protein OS=Leishmania mexicana (s	2
3.365545254	-1.42933	E9B085	tr|E9B085|E9B085_LEIMU Coatomer subunit gamma OS=Leishmania mexicana	3
5.229502631	-1.43078	E9AQZ5	tr|E9AQZ5|E9AQZ5_LEIMU Uncharacterized protein OS=Leishmania mexicana (	2
4.357432327	-1.43121	E9AZI2	tr|E9AZI2|E9AZI2_LEIMU Histone H2B OS=Leishmania mexicana (strain MHOM,	2
3.6050811	-1.43281	E9B609	tr|E9B609|E9B609_LEIMU Putative ubiquitin-conjugating enzyme e2 OS=Leishr	2
4.229286681	-1.43439	E9B2W9	tr|E9B2W9|E9B2W9_LEIMU Uncharacterized protein OS=Leishmania mexicana	4
3.68904346	-1.45103	E9B2J9	tr|E9B2J9|E9B2J9_LEIMU Putative RNA binding protein OS=Leishmania mexicar	6
4.226519684	-1.45292	E9B714	tr|E9B714|E9B714_LEIMU Uncharacterized protein OS=Leishmania mexicana (s	2
4.027673738	-1.46063	E9ATD8	tr|E9ATD8|E9ATD8_LEIMU Putative cysteine synthase OS=Leishmania mexican	6
3.477876935	-1.46133	E9AKI9	tr|E9AKI9|E9AKI9_LEIMU S-methyl-5-thioadenosine phosphorylase OS=Leishm	2
4.137593232	-1.46401	E9AXT3	tr|E9AXT3|E9AXT3_LEIMU Uncharacterized protein OS=Leishmania mexicana (s	2
4.095830023	-1.46732	E9AV90;Q8	tr|E9AV90|E9AV90_LEIMU Putative 40S ribosomal protein S23 OS=Leishmania	2
4.167742501	-1.4728	E9B2H5	tr|E9B2H5|E9B2H5_LEIMU Profilin OS=Leishmania mexicana (strain MHOM/GT	5
4.071874818	-1.47389	E9AKN6;E9	tr|E9AKN6|E9AKN6_LEIMU Putative ATP-binding cassette protein subfamily G,	2
3.802459022	-1.47735	E9AQ94	tr|E9AQ94|E9AQ94_LEIMU Putative 40S ribosomal protein S3 OS=Leishmania r	7
5.013432587	-1.48527	E9B6I5	tr|E9B6I5|E9B6I5_LEIMU Putative ubiquitin-activating enzyme e1 OS=Leishmar	4
4.218324409	-1.48761	E9AXZ5	tr|E9AXZ5|E9AXZ5_LEIMU Adenine phosphoribosyltransferase OS=Leishmania	2
3.987452461	-1.50694	E9B226	tr|E9B226|E9B226_LEIMU Putative 3,2-trans-enoyl-CoA isomerase, mitochondı	5
4.130174372	-1.51784	E9AQB1	tr|E9AQB1|E9AQB1_LEIMU 60S acidic ribosomal protein P2 OS=Leishmania me	5
4.114964527	-1.53904	E9AQI0	tr|E9AQI0|E9AQI0_LEIMU Protein tyrosine phosphatase-like protein OS=Leishn	3
3.534382306	-1.54963	O61059;E9A	tr|O61059|O61059_LEIME Glucose transporter OS=Leishmania mexicana OX=5	3
4.218224844	-1.55213	E9AQV4	tr|E9AQV4|E9AQV4_LEIMU Uncharacterized protein OS=Leishmania mexicana	5
3.732425156	-1.55384	E9B730	tr|E9B730|E9B730_LEIMU Polyadenylate-binding protein OS=Leishmania mexic	10
3.798084581	-1.55847	E9AVM6	tr|E9AVM6|E9AVM6_LEIMU Putative 40S ribosomal protein S15 OS=Leishmani	2
3.448509225	-1.57037	E9B2Z9	tr|E9B2Z9|E9B2Z9_LEIMU Uncharacterized protein OS=Leishmania mexicana (s	6
3.856100067	-1.58519	E9B669;E9A	tr|E9B669|E9B669_LEIMU Putative 60S Ribosomal protein L36 OS=Leishmania	3
4.409368639	-1.5938	E9B3L1	tr|E9B3L1|E9B3L1_LEIMU Putative ATP-binding cassette protein OS=Leishmani	2
4.077745935	-1.60274	E9ARG7	tr|E9ARG7|E9ARG7_LEIMU Uncharacterized protein OS=Leishmania mexicana (	4
4.397982956	-1.61128	E9ALW5;E9	tr|E9ALW5|E9ALW5_LEIMU Putative ribosomal protein L1a OS=Leishmania me	9
3.324012676	-1.6195	E9B2A6;E9B	tr|E9B2A6|E9B2A6_LEIMU Iron/zinc transporter protein-like protein OS=Leishn	3
4.548176512	-1.62209	E9B4X7	tr|E9B4X7|E9B4X7_LEIMU Uncharacterized protein OS=Leishmania mexicana (s	3
4.267852827	-1.62722	E9AV22	tr|E9AV22|E9AV22_LEIMU Dihydrolipoamide acetyltransferaselike protein OS=	2
3.899527077	-1.63223	E9AUK0	tr|E9AUK0|E9AUK0_LEIMU CTP synthase OS=Leishmania mexicana (strain MHC	6
3.64564411	-1.64671	E9AZ74	tr|E9AZ74|E9AZ74_LEIMU Uncharacterized protein OS=Leishmania mexicana (s	2
4.044260604	-1.65235	E9B6X6	tr|E9B6X6|E9B6X6_LEIMU Uncharacterized protein OS=Leishmania mexicana (s	2
3.200683635	-1.65985	E9ATF9	tr|E9ATF9|E9ATF9_LEIMU Nascent polypeptide-associated complex subunit be	2

4.14615152	-1.6701	E9ASR3	tr|E9ASR3|E9ASR3_LEIMU Putative translation elongation factor 1-beta OS=Lei	4
4.587616628	-1.70519	E9B547	tr|E9B547|E9B547_LEIMU Uncharacterized protein OS=Leishmania mexicana (s	5
3.334627316	-1.75256	E9AXI8	tr|E9AXI8|E9AXI8_LEIMU Uncharacterized protein OS=Leishmania mexicana (st	2
4.232135021	-1.75652	E9ANR0	tr|E9ANR0|E9ANR0_LEIMU Putative S-phase kinase-associated protein (Putativ	2
4.084118809	-1.78819	E9AUR8	tr|E9AUR8|E9AUR8_LEIMU Putative calpain-like cysteine peptidase OS=Leishm	3
4.593450888	-1.7954	E9B1E7;E9B	tr|E9B1E7|E9B1E7_LEIMU Putative 60S acidic ribosomal protein P2 OS=Leishma	5
4.20057321	-1.81028	E9B6Q4	tr|E9B6Q4|E9B6Q4_LEIMU Putative 60S ribosomal protein L27A/L29 OS=Leishr	3
4.132660887	-1.88545	E9AUQ8;E9A	tr|E9AUQ8|E9AUQ8_LEIMU Putative calpain-like cysteine peptidase OS=Leishm	5
4.813952387	-1.90777	E9B072	tr|E9B072|E9B072_LEIMU Eukaryotic translation initiation factor,putative OS=L	2
4.307801667	-2.02918	E9B533;E9A	tr|E9B533|E9B533_LEIMU Putative ribosomal protein l35a OS=Leishmania mex	2
3.954283814	-2.03819	O46368;E9E	tr|O46368|O46368_LEIME Surface protein amastin OS=Leishmania mexicana O	2
4.376294359	-2.16828	Q9NL78;Q9I	tr|Q9NL78|Q9NL78_LEIAM Histone H3 OS=Leishmania amazonensis OX=5659 (	2
4.150676902	-2.33644	E8NHK6;E8I	tr|E8NHK6|E8NHK6_LEIMU WGS CADB00000000 data, contig 52 (Fragment) OS	2

Table S8. High-confidence tanespimycin affected RBPs of *L.mexicana* amastigote.

[-Log(P-value)]	Log2 FC	Protein IDs	Fasta headers	Unique peptides
4.121399371	1.12651	E9B4I0	tr|E9B4I0|E9B4I0_LEIMU Malate dehydrogenase OS=Leishmania mexicana	4
3.540031701	1.06091	E9ALE3	tr|E9ALE3|E9ALE3_LEIMU Uncharacterized protein OS=Leishmania mexican	2
2.938793625	0.83964	E9AUQ1	tr|E9AUQ1|E9AUQ1_LEIMU Putative phosphatidylinositol 3-kinase OS=Leisl	2
2.930543953	0.82965	E9AUW3	tr|E9AUW3|E9AUW3_LEIMU Uncharacterized protein OS=Leishmania mexi	2
2.896650685	0.82755	E9AY81	tr|E9AY81|E9AY81_LEIMU Putative dynein heavy chain OS=Leishmania mex	2
2.865852205	0.75888	E9ATU9	tr|E9ATU9|E9ATU9_LEIMU Uncharacterized protein OS=Leishmania mexica	2
2.198287548	0.65356	E9AR86	tr|E9AR86|E9AR86_LEIMU Uncharacterized protein OS=Leishmania mexica	2
2.495171932	0.64891	Q95PT4;E9	tr|Q95PT4|Q95PT4_LEIME Myo-inositol-1-phosphate synthase OS=Leishma	2
1.119272661	0.61108	E9AMS5	tr|E9AMS5|E9AMS5_LEIMU Putative ubiquitin ligase OS=Leishmania mexica	2
2.562821094	0.60033	E9AKA3	tr|E9AKA3|E9AKA3_LEIMU Uncharacterized protein OS=Leishmania mexica	2
1.745011307	0.48098	E9AMC2	tr|E9AMC2|E9AMC2_LEIMU Uncharacterized protein OS=Leishmania mexic	2
2.19097214	0.4708	E9B5Z0	tr|E9B5Z0|E9B5Z0_LEIMU Uncharacterized protein OS=Leishmania mexican	2
2.137301507	0.46352	E9AQY7	tr|E9AQY7|E9AQY7_LEIMU Uncharacterized protein OS=Leishmania mexica	2
1.884222345	0.45042	E9AVX0	tr|E9AVX0|E9AVX0_LEIMU Protein MAK16 homolog OS=Leishmania mexica	2
2.305341613	0.43973	E9AL32	tr|E9AL32|E9AL32_LEIMU Uncharacterized protein OS=Leishmania mexican	3
1.444573131	0.43296	E9AT88	tr|E9AT88|E9AT88_LEIMU Putative ATP synthase OS=Leishmania mexicana	2
1.805043111	0.40824	E9B5V1	tr|E9B5V1|E9B5V1_LEIMU Uncharacterized protein OS=Leishmania mexica	2
1.843792585	0.39522	E9B552	tr|E9B552|E9B552_LEIMU RuvB-like helicase OS=Leishmania mexicana (stra	4
1.771679273	0.35515	E9B028	tr|E9B028|E9B028_LEIMU Putative mitochondrial DEAD box protein OS=Lei	6
1.915049112	-0.3448	E9AZ84	tr|E9AZ84|E9AZ84_LEIMU Uncharacterized protein OS=Leishmania mexican	8
1.89636695	-0.3456	E9B2N1	tr|E9B2N1|E9B2N1_LEIMU Putative dynein OS=Leishmania mexicana (strain	3
2.154104548	-0.3463	E9B2Z9	tr|E9B2Z9|E9B2Z9_LEIMU Uncharacterized protein OS=Leishmania mexican	3
1.827549658	-0.3482	E9AV16	tr|E9AV16|E9AV16_LEIMU Putative DnaJ protein OS=Leishmania mexicana	4
1.773323485	-0.3504	E9AXQ3	tr|E9AXQ3|E9AXQ3_LEIMU Pyruvate dehydrogenase E1 component subuni	6
1.664253881	-0.3505	E9APF9	tr|E9APF9|E9APF9_LEIMU Uncharacterized protein OS=Leishmania mexican	4
2.15416224	-0.3511	E9B677	tr|E9B677|E9B677_LEIMU Uncharacterized protein OS=Leishmania mexican	8
2.572185919	-0.3523	E9B3G3	tr|E9B3G3|E9B3G3_LEIMU Uncharacterized protein OS=Leishmania mexica	2
1.640081017	-0.3524	E9AUS9	tr|E9AUS9|E9AUS9_LEIMU Putative axoneme central apparatus protein OS	6
2.081947748	-0.3524	E9ASZ0	tr|E9ASZ0|E9ASZ0_LEIMU Putative DEAD box RNA helicase OS=Leishmania	7
2.173498831	-0.3527	E9B2F7	tr|E9B2F7|E9B2F7_LEIMU Uncharacterized protein OS=Leishmania mexican	4
1.545839131	-0.3528	E9AVE3	tr|E9AVE3|E9AVE3_LEIMU Uncharacterized protein OS=Leishmania mexica	2
1.680211342	-0.3532	E9AKL0;Q8	tr|E9AKL0|E9AKL0_LEIMU Stomatin-like protein OS=Leishmania mexicana (	2
3.23319966	-0.354	E9AN22	tr|E9AN22|E9AN22_LEIMU Uncharacterized protein OS=Leishmania mexica	2
1.531524072	-0.3555	E9AS50;E9	tr|E9AS50|E9AS50_LEIMU 4-coumarate:coa ligase-like protein OS=Leishma	2
1.790662306	-0.3563	E9AWI7	tr|E9AWI7|E9AWI7_LEIMU Uncharacterized protein OS=Leishmania mexica	2
1.792264487	-0.3567	E9B478	tr|E9B478|E9B478_LEIMU Putative heat shock protein OS=Leishmania mex	17
1.466089739	-0.3569	E9AT87	tr|E9AT87|E9AT87_LEIMU Uncharacterized protein OS=Leishmania mexican	2
1.814827489	-0.3571	E9ALK3	tr|E9ALK3|E9ALK3_LEIMU Putative GTP-binding protein OS=Leishmania me	16
1.862434676	-0.3571	E9B6L3;Q8	tr|E9B6L3|E9B6L3_LEIMU 6-phosphogluconate dehydrogenase, decarboxyl	2
2.672765154	-0.3575	E9AJM8	tr|E9AJM8|E9AJM8_LEIMU Uncharacterized protein OS=Leishmania mexica	4
1.707037313	-0.3577	E9B099;Q0	tr|E9B099|E9B099_LEIMU Putative heat-shock protein hsp70 OS=Leishman	22
1.962319981	-0.3593	E9B5Q7	tr|E9B5Q7|E9B5Q7_LEIMU Uncharacterized protein OS=Leishmania mexica	4
1.52532595	-0.3593	E9B4E9	tr|E9B4E9|E9B4E9_LEIMU Uncharacterized protein OS=Leishmania mexican	5
1.765286769	-0.3597	E9ATT5;E9	tr|E9ATT5|E9ATT5_LEIMU 40S ribosomal protein SA OS=Leishmania mexica	9
1.643608096	-0.3609	E9AS67	tr|E9AS67|E9AS67_LEIMU Uncharacterized protein OS=Leishmania mexican	5
2.116021901	-0.3614	E9AVL4	tr|E9AVL4|E9AVL4_LEIMU Uncharacterized protein OS=Leishmania mexican	8
1.402744716	-0.3617	E9B226	tr|E9B226|E9B226_LEIMU Putative 3,2-trans-enoyl-CoA isomerase, mitoch	2
1.705562638	-0.3618	E9B1G1	tr|E9B1G1|E9B1G1_LEIMU Uncharacterized protein OS=Leishmania mexica	2
1.416708995	-0.3624	E9B3P7	tr|E9B3P7|E9B3P7_LEIMU Uncharacterized protein OS=Leishmania mexican	3
1.663534207	-0.3626	E9B620	tr|E9B620|E9B620_LEIMU Putative threonyl-tRNA synthetase OS=Leishmar	9
1.811150265	-0.3626	E9AKR8	tr|E9AKR8|E9AKR8_LEIMU Putative 60S ribosomal protein L19 OS=Leishma	3
1.854888435	-0.3628	E9AVB8	tr|E9AVB8|E9AVB8_LEIMU Structural maintenance of chromosomes protei	2
1.815613012	-0.3632	E9B422	tr|E9B422|E9B422_LEIMU Putative ABC transporter OS=Leishmania mexica	3

1.238760631	-0.3641	E9AW22;E!	tr|E9AW22|E9AW22_LEIMU Putative ATP-binding cassette protein subfami	2
2.104600108	-0.3647	E9AP81	tr|E9AP81|E9AP81_LEIMU 40S ribosomal protein S12 OS=Leishmania mexic	6
1.515354636	-0.3647	E9AJK7	tr|E9AJK7|E9AJK7_LEIMU Uncharacterized protein OS=Leishmania mexican	4
1.319079318	-0.3647	E9AMI1	tr|E9AMI1|E9AMI1_LEIMU Stress-induced protein sti1 OS=Leishmania mexi	3
1.633972478	-0.3647	E9AZQ5	tr|E9AZQ5|E9AZQ5_LEIMU Ribonucleoside-diphosphate reductase OS=Leisl	3
1.832107105	-0.3652	E9B085	tr|E9B085|E9B085_LEIMU Coatomer subunit gamma OS=Leishmania mexic	4
2.269371878	-0.3655	E9AYZ5	tr|E9AYZ5|E9AYZ5_LEIMU Putative isovaleryl-coA dehydrogenase OS=Leish	4
2.09971106	-0.3656	E9ATM0	tr|E9ATM0|E9ATM0_LEIMU Proteasome regulatory ATPase subunit OS=Lei:	2
1.408910828	-0.3664	E9ATL1	tr|E9ATL1|E9ATL1_LEIMU Uncharacterized protein OS=Leishmania mexican	4
1.543530567	-0.3671	E9B1A6	tr|E9B1A6|E9B1A6_LEIMU Uncharacterized protein OS=Leishmania mexicai	4
2.107568945	-0.3679	P16126;E9	sp|P16126|DRTS_LEIAM Bifunctional dihydrofolate reductase-thymidylate s	7
1.355158093	-0.3684	E9B2B7	tr|E9B2B7|E9B2B7_LEIMU Uncharacterized protein OS=Leishmania mexicai	2
1.928182159	-0.3687	E9AVY6	tr|E9AVY6|E9AVY6_LEIMU Alanine--tRNA ligase OS=Leishmania mexicana (s	16
1.396063457	-0.3687	E9B760	tr|E9B760|E9B760_LEIMU Uncharacterized protein OS=Leishmania mexicar	6
1.952197357	-0.3689	E9AQV6;H(	tr|E9AQV6|E9AQV6_LEIMU Paraflagellar rod protein 2C OS=Leishmania me	2
1.76262146	-0.369	E9ARS1	tr|E9ARS1|E9ARS1_LEIMU Putative heat shock protein OS=Leishmania mex	27
1.856091594	-0.3691	E9B430	tr|E9B430|E9B430_LEIMU Putative phosphoribosylpyrophosphate syntheta	3
1.699823524	-0.3697	A0A0R6YB	tr|A0A0R6YBU4|A0A0R6YBU4_LEIME Thiamine biosynthesis-like protein (Fı	6
1.722422275	-0.37	E9AZ36	tr|E9AZ36|E9AZ36_LEIMU Uncharacterized protein OS=Leishmania mexicar	9
1.804425065	-0.3704	E9B3B0	tr|E9B3B0|E9B3B0_LEIMU Putative chaperonin alpha subunit OS=Leishman	12
2.09864229	-0.3712	E9ANM5	tr|E9ANM5|E9ANM5_LEIMU Uncharacterized protein OS=Leishmania mexi	2
2.261413127	-0.372	E9AL41	tr|E9AL41|E9AL41_LEIMU Uncharacterized protein OS=Leishmania mexicar	3
1.924750278	-0.3725	E9ATW8	tr|E9ATW8|E9ATW8_LEIMU Uncharacterized protein OS=Leishmania mexic	8
1.652463486	-0.3725	E9B0L7	tr|E9B0L7|E9B0L7_LEIMU Putative adenosine kinase OS=Leishmania mexica	7
1.658760464	-0.3726	E9B074	tr|E9B074|E9B074_LEIMU Uncharacterized protein OS=Leishmania mexicar	2
1.329856808	-0.3728	E9B6S2	tr|E9B6S2|E9B6S2_LEIMU Uncharacterized protein OS=Leishmania mexicar	2
1.294032687	-0.3731	E9ALL7	tr|E9ALL7|E9ALL7_LEIMU Uncharacterized protein OS=Leishmania mexican	3
1.921006889	-0.3735	E9ARK0	tr|E9ARK0|E9ARK0_LEIMU Putative 60S ribosomal protein L10a OS=Leishm	4
1.659249062	-0.3742	E9AWK3	tr|E9AWK3|E9AWK3_LEIMU Ankyrin/TPR repeat protein OS=Leishmania me	4
1.794161876	-0.3753	E9ATK7	tr|E9ATK7|E9ATK7_LEIMU Uncharacterized protein OS=Leishmania mexicar	14
1.498014614	-0.3753	E9ANI7	tr|E9ANI7|E9ANI7_LEIMU Ribosome biogenesis protein BOP1 homolog OS=	3
1.60726501	-0.3758	E9AZU7	tr|E9AZU7|E9AZU7_LEIMU Putative phenylalanine-4-hydroxylase OS=Leish	3
1.565655863	-0.3762	E9B1J0	tr|E9B1J0|E9B1J0_LEIMU Putative calpain-like cysteine peptidase OS=Leish	2
1.582704213	-0.3775	E9AMX3	tr|E9AMX3|E9AMX3_LEIMU Putative AAA family ATPase OS=Leishmania me	10
1.759494943	-0.3776	E9APJ0	tr|E9APJ0|E9APJ0_LEIMU Putative chaperonin TCP20 OS=Leishmania mexic	10
1.585396934	-0.3779	E9ASH6;O(	tr|E9ASH6|E9ASH6_LEIMU Elongation factor G, mitochondrial OS=Leishmar	7
2.147832464	-0.3796	Q4KXA7;E!	tr|Q4KXA7|Q4KXA7_LEIME Cytoplasmic dynein heavy chain 2.2 OS=Leishma	4
1.426423631	-0.3797	E9ARZ6	tr|E9ARZ6|E9ARZ6_LEIMU Putative nucleosome assembly protein OS=Leish	4
2.230489179	-0.3799	E9AZA2	tr|E9AZA2|E9AZA2_LEIMU Putative rRNA methyltransferase OS=Leishmania	3
1.643620298	-0.3808	E9API9	tr|E9API9|E9API9_LEIMU Putative dynein heavy chain OS=Leishmania mexi	38
1.289449376	-0.3809	E9AL69	tr|E9AL69|E9AL69_LEIMU Uncharacterized protein OS=Leishmania mexicar	3
2.190279014	-0.381	E9AJZ0	tr|E9AJZ0|E9AJZ0_LEIMU DNA topoisomerase type IB small subunit OS=Leis	3
2.162105353	-0.3815	E9AU29	tr|E9AU29|E9AU29_LEIMU Uncharacterized protein OS=Leishmania mexica	2
1.560771331	-0.3816	E9AZB9	tr|E9AZB9|E9AZB9_LEIMU Uncharacterized protein OS=Leishmania mexicar	2
1.811192571	-0.3817	E9ATD8	tr|E9ATD8|E9ATD8_LEIMU Putative cysteine synthase OS=Leishmania mexi	5
2.862305743	-0.3823	E9AQW0;E	tr|E9AQW0|E9AQW0_LEIMU Putative kinesin OS=Leishmania mexicana (str	2
1.227398924	-0.3829	E9APH6	tr|E9APH6|E9APH6_LEIMU Uncharacterized protein OS=Leishmania mexica	2
1.778770582	-0.3831	E9B1L5	tr|E9B1L5|E9B1L5_LEIMU Uncharacterized protein OS=Leishmania mexican	2
1.679561733	-0.3842	E9B2V1	tr|E9B2V1|E9B2V1_LEIMU Uncharacterized protein OS=Leishmania mexicai	6
1.556065107	-0.3843	E9AQV1	tr|E9AQV1|E9AQV1_LEIMU Uncharacterized protein OS=Leishmania mexica	2
1.685382259	-0.3844	E9B2C5	tr|E9B2C5|E9B2C5_LEIMU Uncharacterized protein OS=Leishmania mexicar	3
2.475911134	-0.3847	E9B1B7	tr|E9B1B7|E9B1B7_LEIMU Uncharacterized protein OS=Leishmania mexicai	3
2.054036985	-0.3857	E9B6I5	tr|E9B6I5|E9B6I5_LEIMU Putative ubiquitin-activating enzyme e1 OS=Leish	7
2.154003631	-0.3865	E9ASE2	tr|E9ASE2|E9ASE2_LEIMU eIF3-interacting protein-like protein OS=Leishma	4
2.231280665	-0.3866	E9B038	tr|E9B038|E9B038_LEIMU Uncharacterized protein OS=Leishmania mexicar	10
1.952908261	-0.3871	E9APW3;Q	tr|E9APW3|E9APW3_LEIMU Enolase OS=Leishmania mexicana (strain MHO	11
1.552040097	-0.3876	E9AWL4	tr|E9AWL4|E9AWL4_LEIMU Putative ATP-dependent RNA helicase OS=Leisl	2

2.247789261	-0.3879	E9AZZ2	tr|E9AZZ2|E9AZZ2_LEIMU Proteasome regulatory non-ATP-ase subunit 2,pu	4
1.836934335	-0.3886	E9AL37	tr|E9AL37|E9AL37_LEIMU Acetylornithine deacetylase-like protein OS=Leisl	4
1.2685231	-0.3887	E9B6S3	tr|E9B6S3|E9B6S3_LEIMU Putative PRP8 protein homologue (U5 snrna-assc	2
1.552398064	-0.3895	E9AV82	tr|E9AV82|E9AV82_LEIMU Uncharacterized protein OS=Leishmania mexica	8
1.519890473	-0.39	E9ALG1	tr|E9ALG1|E9ALG1_LEIMU Uncharacterized protein OS=Leishmania mexica	11
1.957719176	-0.3901	E9AJQ5;F5	tr|E9AJQ5|E9AJQ5_LEIMU Delta-1-pyrroline-5-carboxylate dehydrogenase,	12
1.946101004	-0.3903	E9AQ24	tr|E9AQ24|E9AQ24_LEIMU Lysine--tRNA ligase OS=Leishmania mexicana (s	7
1.996981963	-0.3904	E9B1E2	tr|E9B1E2|E9B1E2_LEIMU Uncharacterized protein OS=Leishmania mexicar	12
1.904474236	-0.3906	E9B4B4	tr|E9B4B4|E9B4B4_LEIMU Putative translation initiation factor IF-2 OS=Leis	4
1.334028013	-0.3913	E9B235	tr|E9B235|E9B235_LEIMU Uncharacterized protein OS=Leishmania mexicar	4
1.662673496	-0.3922	E9B1K4	tr|E9B1K4|E9B1K4_LEIMU Putative ATP-binding cassette protein subfamily	3
1.292464451	-0.3927	E9AQP7	tr|E9AQP7|E9AQP7_LEIMU Uncharacterized protein OS=Leishmania mexica	2
2.023648749	-0.393	E9AVE1	tr|E9AVE1|E9AVE1_LEIMU Putative RNA helicase OS=Leishmania mexicana	7
1.840995874	-0.393	E9B5J1	tr|E9B5J1|E9B5J1_LEIMU Uncharacterized protein OS=Leishmania mexican	2
1.878083131	-0.3931	E9AV50	tr|E9AV50|E9AV50_LEIMU Uncharacterized protein OS=Leishmania mexica	2
2.147167062	-0.3939	E9AXJ1	tr|E9AXJ1|E9AXJ1_LEIMU Aldehyde dehydrogenase, mitochondrial OS=Leis	2
2.154969792	-0.3971	E9B2L3	tr|E9B2L3|E9B2L3_LEIMU Ethanolamine-phosphate cytidylyltransferase,pu	2
1.358134418	-0.3977	E9AUA1	tr|E9AUA1|E9AUA1_LEIMU 2,3-bisphosphoglycerate-independent phosphc	2
1.94764322	-0.3978	E9B0H1;E9	tr|E9B0H1|E9B0H1_LEIMU Putative aspartyl-tRNA synthetase OS=Leishmar	6
1.18593249	-0.398	E9AXD9	tr|E9AXD9|E9AXD9_LEIMU DNA-directed RNA polymerase subunit beta OS	3
2.34467571	-0.3995	E9B4H8	tr|E9B4H8|E9B4H8_LEIMU Putative adenylate kinase OS=Leishmania mexic	3
1.591723176	-0.3996	E9AMI0	tr|E9AMI0|E9AMI0_LEIMU Uncharacterized protein OS=Leishmania mexica	5
1.74968361	-0.3999	E9AUF0;E9	tr|E9AUF0|E9AUF0_LEIMU Phosphoglycerate kinase OS=Leishmania mexica	9
1.806155654	-0.4004	E9AY14	tr|E9AY14|E9AY14_LEIMU C-1-tetrahydrofolate synthase, cytoplasmic,puta	3
2.598989687	-0.401	E9B4I7	tr|E9B4I7|E9B4I7_LEIMU Uncharacterized protein OS=Leishmania mexican	2
1.845974558	-0.4011	E9ALV8;A5	tr|E9ALV8|E9ALV8_LEIMU Putative tryparedoxin OS=Leishmania mexicana	2
1.767789404	-0.4011	E9AW84	tr|E9AW84|E9AW84_LEIMU 3-ketoacyl-CoA thiolase-like protein OS=Leishr	9
1.841356866	-0.402	E9ATZ6	tr|E9ATZ6|E9ATZ6_LEIMU Putative isoleucyl-tRNA synthetase OS=Leishmar	7
1.729680746	-0.4021	E9AT13	tr|E9AT13|E9AT13_LEIMU Putative tyrosine aminotransferase OS=Leishma	11
2.098210386	-0.4023	E9AQX9	tr|E9AQX9|E9AQX9_LEIMU Uncharacterized protein OS=Leishmania mexica	6
1.84140246	-0.4037	E9B2B3	tr|E9B2B3|E9B2B3_LEIMU Methylcrotonoyl-coa carboxylase biotinylated su	5
2.03505178	-0.4037	E9AZ73	tr|E9AZ73|E9AZ73_LEIMU Putative eukaryotic translation release factor OS	12
2.075247966	-0.4046	E9ASQ6	tr|E9ASQ6|E9ASQ6_LEIMU Transitional endoplasmic reticulum ATPase,puta	13
1.325059704	-0.4051	E9AZA7;E9	tr|E9AZA7|E9AZA7_LEIMU Branched-chain-amino-acid aminotransferase O	2
2.056526362	-0.4058	E9AVZ4	tr|E9AVZ4|E9AVZ4_LEIMU Uncharacterized protein OS=Leishmania mexica	3
1.825295095	-0.4073	E9B5X5	tr|E9B5X5|E9B5X5_LEIMU Uncharacterized protein OS=Leishmania mexicar	2
1.691728553	-0.4076	E9ARA9	tr|E9ARA9|E9ARA9_LEIMU Putative L-gulonolactone oxidase OS=Leishmani	2
1.864282969	-0.4082	E9AQD3	tr|E9AQD3|E9AQD3_LEIMU Putative nucleolar RNA binding protein OS=Leis	13
1.432211642	-0.409	E9AXL3	tr|E9AXL3|E9AXL3_LEIMU Uncharacterized protein OS=Leishmania mexicar	2
1.977867099	-0.4092	E9AZT2	tr|E9AZT2|E9AZT2_LEIMU Putative electron-transfer-flavoprotein, alpha po	4
2.040910603	-0.4095	E9AWL7	tr|E9AWL7|E9AWL7_LEIMU Dynein intermediate-chain-like protein OS=Leis	3
2.57903217	-0.4095	E9B0V3	tr|E9B0V3|E9B0V3_LEIMU Uncharacterized protein OS=Leishmania mexica	2
2.000701799	-0.4098	E9AQL8;Q	tr|E9AQL8|E9AQL8_LEIMU Putative carbamoyl-phosphate synthase OS=Lei	30
2.635182373	-0.4102	E9AL58	tr|E9AL58|E9AL58_LEIMU Uncharacterized protein OS=Leishmania mexicar	5
1.620338573	-0.4109	E9ALW5;E	tr|E9ALW5|E9ALW5_LEIMU Putative ribosomal protein L1a OS=Leishmania	9
1.782256039	-0.4113	E9AZK7	tr|E9AZK7|E9AZK7_LEIMU Uncharacterized protein OS=Leishmania mexicar	2
1.419998163	-0.4146	E9AV49	tr|E9AV49|E9AV49_LEIMU Putative methionyl-tRNA synthetase OS=Leishm	3
1.965473146	-0.4156	E9B473	tr|E9B473|E9B473_LEIMU Succinyl-CoA:3-ketoacid-coenzyme A transferase	8
1.705057189	-0.4163	E9AM02	tr|E9AM02|E9AM02_LEIMU Putative lipophosphoglycan biosynthetic prote	4
2.03240035	-0.4164	E9AZ25	tr|E9AZ25|E9AZ25_LEIMU Uncharacterized protein OS=Leishmania mexicar	5
2.088199151	-0.4167	E9B6I9	tr|E9B6I9|E9B6I9_LEIMU Putative ATP-dependent RNA helicase OS=Leishm	23
1.918356361	-0.4169	E9AXM1	tr|E9AXM1|E9AXM1_LEIMU GTP-binding nuclear protein OS=Leishmania m	7
1.490442231	-0.4175	E9AMH0	tr|E9AMH0|E9AMH0_LEIMU Uncharacterized protein OS=Leishmania mexi	3
2.078742255	-0.4188	E9AK58	tr|E9AK58|E9AK58_LEIMU Putative 60S ribosomal protein L10 OS=Leishma	7
2.380684405	-0.4193	E9AM77	tr|E9AM77|E9AM77_LEIMU Transcription factor-like protein OS=Leishmani	10
1.908821284	-0.4194	E9B296	tr|E9B296|E9B296_LEIMU Putative serine/threonine-protein kinase OS=Lei	2
1.319108248	-0.4194	E9ASK9	tr|E9ASK9|E9ASK9_LEIMU Eukaryotic translation initiation factor 6 OS=Leisl	2

1.977874253	-0.4195	E9AJV2	tr|E9AJV2|E9AJV2_LEIMU Uncharacterized protein OS=Leishmania mexican	11
1.814039246	-0.4202	E9AV10	tr|E9AV10|E9AV10_LEIMU Uncharacterized protein OS=Leishmania mexica	8
2.285151832	-0.4213	E9ANZ0	tr|E9ANZ0|E9ANZ0_LEIMU Putative alanine aminotransferase OS=Leishmar	4
1.208157027	-0.4217	E9B6P8	tr|E9B6P8|E9B6P8_LEIMU Putative Gim5A protein (Glycosomal membrane	4
1.796583897	-0.4219	E9B2M5	tr|E9B2M5|E9B2M5_LEIMU Chaperonin containing t-complex protein,puta	5
2.355470388	-0.4234	E9AJX2	tr|E9AJX2|E9AJX2_LEIMU Uncharacterized protein OS=Leishmania mexican	2
2.352508603	-0.4238	E9AWE0	tr|E9AWE0|E9AWE0_LEIMU T-complex protein 1 subunit gamma OS=Leishr	14
2.062494743	-0.4249	E9AZ29	tr|E9AZ29|E9AZ29_LEIMU Putative T-complex protein 1, beta subunit OS=L	8
1.839186303	-0.425	E9AKA4;C6	tr|E9AKA4|E9AKA4_LEIMU Actin OS=Leishmania mexicana (strain MHOM/G	7
2.015696358	-0.4252	E9AR10	tr|E9AR10|E9AR10_LEIMU Uncharacterized protein OS=Leishmania mexica	2
1.917651807	-0.4254	E9AYU4	tr|E9AYU4|E9AYU4_LEIMU Ribokinase OS=Leishmania mexicana (strain MH	2
2.188761201	-0.4264	E9AX84	tr|E9AX84|E9AX84_LEIMU Putative poly(A)-binding protein OS=Leishmania	9
1.786883408	-0.4269	E9B5U7	tr|E9B5U7|E9B5U7_LEIMU Uncharacterized protein OS=Leishmania mexica	8
1.870730518	-0.4275	E9AQG7	tr|E9AQG7|E9AQG7_LEIMU Uncharacterized protein OS=Leishmania mexic	4
2.106055502	-0.428	E9AJY2;A0	tr|E9AJY2|E9AJY2_LEIMU Elongation initiation factor 2 alpha subunit,putati	7
1.737237694	-0.4281	E9APS7	tr|E9APS7|E9APS7_LEIMU Uncharacterized protein OS=Leishmania mexicar	2
1.73536773	-0.4283	E9B2Z5	tr|E9B2Z5|E9B2Z5_LEIMU Uncharacterized protein OS=Leishmania mexicar	5
1.886602311	-0.4286	E9AX01	tr|E9AX01|E9AX01_LEIMU Uncharacterized protein OS=Leishmania mexicar	7
1.638941551	-0.4289	E9AY42	tr|E9AY42|E9AY42_LEIMU Uncharacterized protein OS=Leishmania mexicar	3
2.039929481	-0.4291	E9AMY4	tr|E9AMY4|E9AMY4_LEIMU Putative paraflagellar rod component OS=Leish	5
1.992424012	-0.4294	E9AXI8	tr|E9AXI8|E9AXI8_LEIMU Uncharacterized protein OS=Leishmania mexican	2
2.032429416	-0.4296	E9B170;A0	tr|E9B170|E9B170_LEIMU Glyceraldehyde-3-phosphate dehydrogenase OS	7
1.852298444	-0.4305	E9ALU2	tr|E9ALU2|E9ALU2_LEIMU Putative carnitine/choline acetyltransferase OS=	3
1.575635804	-0.4307	E9B5Z9	tr|E9B5Z9|E9B5Z9_LEIMU Putative pre-mRNA splicing factor ATP-depender	3
2.56978477	-0.4309	E9B549	tr|E9B549|E9B549_LEIMU Uncharacterized protein OS=Leishmania mexicar	5
2.115036757	-0.4316	E9AUD1;A	tr|E9AUD1|E9AUD1_LEIMU Protein disulfide-isomerase OS=Leishmania me	11
2.289335738	-0.4319	E9AZ08	tr|E9AZ08|E9AZ08_LEIMU Putative cysteine desulfurase OS=Leishmania me	6
1.998446883	-0.4335	E9AV48	tr|E9AV48|E9AV48_LEIMU Uncharacterized protein OS=Leishmania mexica	8
2.291308854	-0.4339	E9B2C2	tr|E9B2C2|E9B2C2_LEIMU Uncharacterized protein OS=Leishmania mexicar	9
1.741549294	-0.4341	E9AZ50	tr|E9AZ50|E9AZ50_LEIMU Uncharacterized protein OS=Leishmania mexicar	5
1.660609949	-0.4355	E9B4N7	tr|E9B4N7|E9B4N7_LEIMU Uncharacterized protein OS=Leishmania mexica	2
2.481699444	-0.4368	E9ATM4	tr|E9ATM4|E9ATM4_LEIMU ATP-dependent RNA helicase-like protein OS=L	3
2.685860097	-0.4374	E8NHK6;E8	tr|E8NHK6|E8NHK6_LEIMU WGS CADB00000000 data, contig 52 (Fragment	2
1.438666405	-0.4377	E9ALW3	tr|E9ALW3|E9ALW3_LEIMU Uncharacterized protein OS=Leishmania mexic	3
1.742526206	-0.4379	E9AYA3;Q2	tr|E9AYA3|E9AYA3_LEIMU Heat shock protein 70-related protein OS=Leishr	11
1.203497554	-0.4392	E9ALI0	tr|E9ALI0|E9ALI0_LEIMU Phosphodiesterase OS=Leishmania mexicana (stra	2
2.110721198	-0.4399	E9ATX3	tr|E9ATX3|E9ATX3_LEIMU Putative NADH dehydrogenase OS=Leishmania n	8
1.918672914	-0.4403	E9ATU8	tr|E9ATU8|E9ATU8_LEIMU Uncharacterized protein OS=Leishmania mexica	2
2.217863972	-0.4407	E9ARW1	tr|E9ARW1|E9ARW1_LEIMU Putative fibrillarin OS=Leishmania mexicana (s	5
2.392084199	-0.4407	E9AZE9	tr|E9AZE9|E9AZE9_LEIMU Putative heat shock protein DNAJ OS=Leishmania	7
1.78843203	-0.4412	E8NHG6;E8	tr|E8NHG6|E8NHG6_LEIMU Uncharacterized protein LmxM_30_0440_1 OS	14
1.698365288	-0.4414	E9ALH0	tr|E9ALH0|E9ALH0_LEIMU Uncharacterized protein OS=Leishmania mexicar	2
1.729939874	-0.442	E9AUC7	tr|E9AUC7|E9AUC7_LEIMU Putative T-complex protein 1, theta subunit OS=	8
2.264306637	-0.4428	E9B1C6	tr|E9B1C6|E9B1C6_LEIMU S-adenosylmethionine synthase OS=Leishmania	9
1.820190241	-0.4428	E9AQ77	tr|E9AQ77|E9AQ77_LEIMU Uncharacterized protein OS=Leishmania mexica	3
2.588998072	-0.4452	E9B5D2	tr|E9B5D2|E9B5D2_LEIMU Cleavage and polyadenylation specificity factor,	2
2.221899377	-0.4455	E9AQA3	tr|E9AQA3|E9AQA3_LEIMU Tryparedoxin peroxidase OS=Leishmania mexic	2
1.368315708	-0.446	E9ATR2	tr|E9ATR2|E9ATR2_LEIMU Uncharacterized protein OS=Leishmania mexicar	2
1.957812016	-0.446	E9ANP4	tr|E9ANP4|E9ANP4_LEIMU Pyruvate, phosphate dikinase OS=Leishmania m	24
2.701480672	-0.4461	E9AQS3	tr|E9AQS3|E9AQS3_LEIMU Tyrosyl or methionyl-tRNA synthetase-like prote	3
2.012227369	-0.4467	E9ANQ5	tr|E9ANQ5|E9ANQ5_LEIMU Uncharacterized protein OS=Leishmania mexic	4
1.989886349	-0.447	E9AKB8	tr|E9AKB8|E9AKB8_LEIMU Putative nucleolar RNA helicase II OS=Leishmani	9
1.668500129	-0.4473	E9AQ03	tr|E9AQ03|E9AQ03_LEIMU Uncharacterized protein OS=Leishmania mexica	2
2.187068835	-0.4487	E9AN73	tr|E9AN73|E9AN73_LEIMU Flagellar glycoprotein-like protein OS=Leishman	2
1.466267042	-0.4489	E9B163	tr|E9B163|E9B163_LEIMU Putative aldehyde dehydrogenase OS=Leishmani	2
2.130701837	-0.4492	E9AX53	tr|E9AX53|E9AX53_LEIMU 40S ribosomal protein S8 OS=Leishmania mexica	4
1.682874962	-0.4496	Q2TUW2;E	tr|Q2TUW2|Q2TUW2_LEIAM Inosine-5-monophosphate dehydrogenase OS	4

1.999049372	-0.45	E9ATU4	tr|E9ATU4|E9ATU4_LEIMU Uncharacterized protein OS=Leishmania mexica	12
1.964436622	-0.4503	E9B6K5	tr|E9B6K5|E9B6K5_LEIMU Uncharacterized protein OS=Leishmania mexicar	2
1.680750804	-0.4508	E9B0H0	tr|E9B0H0|E9B0H0_LEIMU Eukaryotic translation initiation factor 4e,putati	2
1.905752562	-0.451	E9AU22	tr|E9AU22|E9AU22_LEIMU Uncharacterized protein OS=Leishmania mexica	6
2.266006879	-0.4512	E9AUD5	tr|E9AUD5|E9AUD5_LEIMU Putative eukaryotic translation initiation factor	15
1.931200101	-0.4518	E9ANA0	tr|E9ANA0|E9ANA0_LEIMU Uncharacterized protein OS=Leishmania mexica	2
2.087406424	-0.4522	E9AT85	tr|E9AT85|E9AT85_LEIMU Fibrillarin OS=Leishmania mexicana (strain MHO	3
1.891540473	-0.4524	E9ASC2	tr|E9ASC2|E9ASC2_LEIMU Putative PUF1 OS=Leishmania mexicana (strain M	3
2.013892852	-0.453	E9ALH3	tr|E9ALH3|E9ALH3_LEIMU ATP-dependent 6-phosphofructokinase OS=Leisl	6
2.407981893	-0.4549	E9APT8	tr|E9APT8|E9APT8_LEIMU Putative immunodominant antigen (Tc40 antiger	6
1.959697779	-0.4554	Q95NJ3;E8	tr|Q95NJ3|Q95NJ3_LEIAM Activated C kinase protein OS=Leishmania amaz	10
2.340272519	-0.4554	E9B178	tr|E9B178|E9B178_LEIMU Eukaryotic translation initiation factor 3 subunit	3
2.1435827	-0.4565	E9B3L2;P2	tr|E9B3L2|E9B3L2_LEIMU Heat shock protein 83-1 OS=Leishmania mexican	32
1.976592231	-0.4565	E9B474;E9	tr|E9B474|E9B474_LEIMU Uncharacterized protein OS=Leishmania mexicar	2
1.702866967	-0.4567	E9AV88	tr|E9AV88|E9AV88_LEIMU Putative kinesin OS=Leishmania mexicana (strain	2
1.99323355	-0.4573	E9AS32	tr|E9AS32|E9AS32_LEIMU Putative ABC transporter OS=Leishmania mexica	4
2.296517644	-0.4578	E9B4K3	tr|E9B4K3|E9B4K3_LEIMU Eukaryotic translation initiation factor 5,putative	4
2.044453967	-0.4588	E9B492	tr|E9B492|E9B492_LEIMU Uncharacterized protein OS=Leishmania mexicar	4
2.038054041	-0.4589	E9AZ37	tr|E9AZ37|E9AZ37_LEIMU Putative arginyl-tRNA synthetase OS=Leishmania	16
2.155037347	-0.4589	E9AKB4	tr|E9AKB4|E9AKB4_LEIMU Serine/threonine-protein phosphatase OS=Leish	5
1.96887799	-0.4591	E9B544	tr|E9B544|E9B544_LEIMU Uncharacterized protein OS=Leishmania mexicar	2
2.209220126	-0.4594	E9B699	tr|E9B699|E9B699_LEIMU Putative RNA-binding protein OS=Leishmania me	6
2.223853809	-0.4601	E9ARK6;E9	tr|E9ARK6|E9ARK6_LEIMU Citrate synthase OS=Leishmania mexicana (strai	8
1.73713183	-0.4616	E9B5Y1	tr|E9B5Y1|E9B5Y1_LEIMU Putative casein kinase OS=Leishmania mexicana	3
2.466055516	-0.4618	E9AUS4	tr|E9AUS4|E9AUS4_LEIMU Coatomer subunit beta OS=Leishmania mexican	9
2.161893223	-0.4632	E9ALR5	tr|E9ALR5|E9ALR5_LEIMU Uncharacterized protein OS=Leishmania mexicar	2
1.679563477	-0.4637	E9AN72	tr|E9AN72|E9AN72_LEIMU Uncharacterized protein OS=Leishmania mexica	2
1.533900136	-0.4637	E9AM65	tr|E9AM65|E9AM65_LEIMU DNA-directed RNA polymerase I-like protein O	2
1.699218057	-0.4641	E9AR96	tr|E9AR96|E9AR96_LEIMU Uncharacterized protein OS=Leishmania mexica	3
1.601873126	-0.4641	E9AJW0;E9	tr|E9AJW0|E9AJW0_LEIMU Uncharacterized protein OS=Leishmania mexica	3
1.30663076	-0.4646	E9ARB0	tr|E9ARB0|E9ARB0_LEIMU Protein SDA1 OS=Leishmania mexicana (strain M	3
2.247077379	-0.4662	E9ALV7	tr|E9ALV7|E9ALV7_LEIMU Tryparedoxin OS=Leishmania mexicana (strain M	3
1.642346789	-0.4662	E9AUZ2;Q4	tr|E9AUZ2|E9AUZ2_LEIMU Phosphotransferase OS=Leishmania mexicana (s	13
1.525915639	-0.4663	E9AZQ1	tr|E9AZQ1|E9AZQ1_LEIMU DNA helicase OS=Leishmania mexicana (strain M	2
1.776451006	-0.4664	E9ASM7	tr|E9ASM7|E9ASM7_LEIMU Putative ribosomal protein L24 OS=Leishmania	4
2.261220414	-0.4671	E9AV93	tr|E9AV93|E9AV93_LEIMU T-complex protein 1 subunit delta OS=Leishman	13
1.830831313	-0.4674	E9B4Q3;E9	tr|E9B4Q3|E9B4Q3_LEIMU Elongation factor 1-beta OS=Leishmania mexica	3
1.388738679	-0.4682	E9AV20	tr|E9AV20|E9AV20_LEIMU Uncharacterized protein OS=Leishmania mexica	2
2.00470166	-0.4688	E9AZ83;E9	tr|E9AZ83|E9AZ83_LEIMU Glycosomal phosphoenolpyruvate carboxykinase	10
1.863807148	-0.4694	E9AQ94	tr|E9AQ94|E9AQ94_LEIMU Putative 40S ribosomal protein S3 OS=Leishmar	8
2.530691614	-0.47	E9ANY1;P4	tr|E9ANY1|E9ANY1_LEIMU Glucose-6-phosphate isomerase OS=Leishmania	3
2.332902829	-0.4703	E9AJC9	tr|E9AJC9|E9AJC9_LEIMU 40S ribosomal protein S7 OS=Leishmania mexicar	7
1.846696818	-0.4706	E9ANQ6	tr|E9ANQ6|E9ANQ6_LEIMU Putative eukaryotic release factor 3 OS=Leishm	6
2.103536869	-0.4709	E9AYX8;E9	tr|E9AYX8|E9AYX8_LEIMU Putative small GTP-binding protein Rab1 OS=Leis	7
1.963434616	-0.4713	E9B258	tr|E9B258|E9B258_LEIMU Uncharacterized protein OS=Leishmania mexicar	2
1.952709983	-0.4714	E9B1K6	tr|E9B1K6|E9B1K6_LEIMU Mevalonate kinase OS=Leishmania mexicana (str	3
2.005169465	-0.4719	E9AM37	tr|E9AM37|E9AM37_LEIMU Uncharacterized protein OS=Leishmania mexic	2
2.831819408	-0.4739	E9AMU6	tr|E9AMU6|E9AMU6_LEIMU Uncharacterized protein OS=Leishmania mexi	2
1.987487643	-0.4742	E9AS17	tr|E9AS17|E9AS17_LEIMU Uncharacterized protein OS=Leishmania mexicar	5
2.144273486	-0.4742	E9B4G7	tr|E9B4G7|E9B4G7_LEIMU Uncharacterized protein OS=Leishmania mexica	10
2.129645918	-0.4756	E9AMV9;E	tr|E9AMV9|E9AMV9_LEIMU Putative eukaryotic translation initiation facto	10
2.247387511	-0.4761	E9AYH6	tr|E9AYH6|E9AYH6_LEIMU Uncharacterized protein OS=Leishmania mexica	20
2.228282427	-0.4785	E9AP13	tr|E9AP13|E9AP13_LEIMU NADH:flavin oxidoreductase/NADH oxidase,puta	3
1.931117212	-0.4788	E9B3H7	tr|E9B3H7|E9B3H7_LEIMU Putative GIPL galf transferase OS=Leishmania m	2
2.124537101	-0.479	E9B384	tr|E9B384|E9B384_LEIMU Uncharacterized protein OS=Leishmania mexicar	15
2.076635889	-0.4805	E9B5R2	tr|E9B5R2|E9B5R2_LEIMU 60S ribosomal protein L30 OS=Leishmania mexic	4
1.860908777	-0.4814	E9AXE4	tr|E9AXE4|E9AXE4_LEIMU Putative epsin OS=Leishmania mexicana (strain M	2

2.476046265	-0.482	W6A238;E	tr|W6A238|W6A238_LEIME Elongation factor 1-alpha (Fragment) OS=Leish	20
2.047673584	-0.4822	E9ARI8	tr|E9ARI8|E9ARI8_LEIMU Aconitate hydratase OS=Leishmania mexicana (st	13
2.633877882	-0.4835	E9B1B3	tr|E9B1B3|E9B1B3_LEIMU Phosphoglycerate kinase OS=Leishmania mexica	3
2.293190128	-0.484	E9AT73	tr|E9AT73|E9AT73_LEIMU Succinate--CoA ligase [ADP-forming] subunit bet	13
1.970324929	-0.4853	E9AZX3	tr|E9AZX3|E9AZX3_LEIMU Putative DEAD box RNA helicase OS=Leishmania	5
2.463736519	-0.4858	E9AV28	tr|E9AV28|E9AV28_LEIMU Putative RNA helicase OS=Leishmania mexicana	5
2.036853763	-0.4872	E9B1G4	tr|E9B1G4|E9B1G4_LEIMU DNA-directed RNA polymerase subunit beta OS=	5
1.776317052	-0.4879	E9B423	tr|E9B423|E9B423_LEIMU Nucleolar GTP-binding protein 1 OS=Leishmania	6
1.914971276	-0.488	E9B3U9	tr|E9B3U9|E9B3U9_LEIMU Putative pumilio protein OS=Leishmania mexica	3
2.212540456	-0.4886	E9B193	tr|E9B193|E9B193_LEIMU 3-hydroxy-3-methylglutaryl coenzyme A reducta	4
2.262235372	-0.4897	E9AQA0	tr|E9AQA0|E9AQA0_LEIMU NAD-specific glutamate dehydrogenase OS=Lei	23
1.834883151	-0.4907	E9B0K3	tr|E9B0K3|E9B0K3_LEIMU Uncharacterized protein OS=Leishmania mexican	4
2.620033107	-0.4909	E9AT99	tr|E9AT99|E9AT99_LEIMU 14-3-3 protein-like protein OS=Leishmania mexic	2
1.709667122	-0.491	E9AZV5	tr|E9AZV5|E9AZV5_LEIMU Haloacid dehalogenase-like hydrolase-like prote	2
2.276528537	-0.4918	E9AST4	tr|E9AST4|E9AST4_LEIMU Clathrin heavy chain OS=Leishmania mexicana (s	35
1.762578368	-0.4918	E9B215	tr|E9B215|E9B215_LEIMU Prostaglandin f2-alpha synthase OS=Leishmania	3
1.877062464	-0.492	E9AV21	tr|E9AV21|E9AV21_LEIMU Putative la RNA binding protein OS=Leishmania	2
2.037865943	-0.4922	E9AMW3	tr|E9AMW3|E9AMW3_LEIMU Mitochondrial RNA binding protein 2 OS=Lei	2
2.233995652	-0.4922	E9B494	tr|E9B494|E9B494_LEIMU Isocitrate dehydrogenase [NADP] OS=Leishmania	3
2.160781169	-0.4924	E9AP62;E8	tr|E9AP62|E9AP62_LEIMU Tubulin alpha chain OS=Leishmania mexicana (st	18
1.964146752	-0.4927	E9B6F3	tr|E9B6F3|E9B6F3_LEIMU Galactokinase-like protein OS=Leishmania mexic	3
2.18818493	-0.4931	E9ARA1	tr|E9ARA1|E9ARA1_LEIMU Putative translation initiation factor OS=Leishm	11
3.316516831	-0.4935	E9B1T6	tr|E9B1T6|E9B1T6_LEIMU Uncharacterized protein OS=Leishmania mexican	4
2.239750078	-0.494	E9AX88	tr|E9AX88|E9AX88_LEIMU Putative electron transfer flavoprotein OS=Leish	5
2.312056136	-0.4947	E9AZE0	tr|E9AZE0|E9AZE0_LEIMU Obg-like ATPase 1 OS=Leishmania mexicana (stra	3
2.049587323	-0.4951	E9B297	tr|E9B297|E9B297_LEIMU Putative acetyl-CoA carboxylase OS=Leishmania	36
1.458304005	-0.4952	E9AXC0	tr|E9AXC0|E9AXC0_LEIMU Uncharacterized protein OS=Leishmania mexica	2
1.824894004	-0.4952	Q9NGJ9;O	tr|Q9NGJ9|Q9NGJ9_LEIAM Ribonucleotide reductase small subunit M2 OS=	2
1.658003892	-0.4952	E9AVH3	tr|E9AVH3|E9AVH3_LEIMU Putative ATP synthase F1 subunit gamma prote	4
3.032507542	-0.496	E9ALK4	tr|E9ALK4|E9ALK4_LEIMU Uncharacterized protein OS=Leishmania mexican	5
2.800176269	-0.4962	E9AY75	tr|E9AY75|E9AY75_LEIMU 60S ribosomal export protein NMD3 OS=Leishma	5
2.430941868	-0.4962	Q9BLW4;E	tr|Q9BLW4|Q9BLW4_LEIME GDP-mannose pyrophosphorylase OS=Leishma	2
2.02753642	-0.4967	E9AUQ7;E	tr|E9AUQ7|E9AUQ7_LEIMU Calpain-like cysteine peptidase OS=Leishmania	5
2.255609432	-0.4971	E9AN04	tr|E9AN04|E9AN04_LEIMU Uncharacterized protein OS=Leishmania mexica	5
1.969101954	-0.498	E9ASG1	tr|E9ASG1|E9ASG1_LEIMU Uncharacterized protein OS=Leishmania mexica	3
2.043521532	-0.4981	E9AR24	tr|E9AR24|E9AR24_LEIMU Putative RNA-binding protein OS=Leishmania m	3
2.531439631	-0.4981	E9AZA6	tr|E9AZA6|E9AZA6_LEIMU D-lactate dehydrogenase-like protein OS=Leishn	4
1.814635672	-0.4989	E9AVW4	tr|E9AVW4|E9AVW4_LEIMU Uncharacterized protein OS=Leishmania mexic	4
2.12932197	-0.4994	E9AVR1	tr|E9AVR1|E9AVR1_LEIMU Putative NADH-cytochrome b5 reductase OS=Le	3
2.44133857	-0.4996	E9AQY9	tr|E9AQY9|E9AQY9_LEIMU Putative peptidase t (Peptidase t, putative, met	7
2.290867239	-0.4998	Q86PL4;E9	tr|Q86PL4|Q86PL4_LEIME Paraflagellar rod protein 1D OS=Leishmania mex	15
2.227762844	-0.5001	E9B3W9	tr|E9B3W9|E9B3W9_LEIMU Cysteine conjugate beta-lyase,aminotransferas	3
1.860647421	-0.5017	E9B007	tr|E9B007|E9B007_LEIMU Uncharacterized protein OS=Leishmania mexican	4
2.235649985	-0.5028	E9AMU9	tr|E9AMU9|E9AMU9_LEIMU Elongation factor-1 gamma OS=Leishmania m	7
2.461380759	-0.5029	E9ASR3	tr|E9ASR3|E9ASR3_LEIMU Putative translation elongation factor 1-beta OS=	4
2.134949711	-0.5047	E9B6R4	tr|E9B6R4|E9B6R4_LEIMU T-complex protein 1 subunit eta OS=Leishmania	10
1.754151643	-0.5051	E9B5P3	tr|E9B5P3|E9B5P3_LEIMU Putative 2-oxoisovalerate dehydrogenase beta s	2
2.322345937	-0.5056	E9ASD6	tr|E9ASD6|E9ASD6_LEIMU Elongation factor 2 OS=Leishmania mexicana (st	27
2.330591046	-0.5058	E9AMV3	tr|E9AMV3|E9AMV3_LEIMU Uncharacterized protein OS=Leishmania mexic	11
2.2039599	-0.5061	E9AV90;Q8	tr|E9AV90|E9AV90_LEIMU Putative 40S ribosomal protein S23 OS=Leishma	2
2.553642915	-0.5065	Q8I911;E9	tr|Q8I911|Q8I911_LEIAM Glucose-6-phosphate 1-dehydrogenase OS=Leish	2
1.89430832	-0.5071	E9B5M6	tr|E9B5M6|E9B5M6_LEIMU Coatomer subunit alpha OS=Leishmania mexic	11
2.166161316	-0.5142	E9B578;E9	tr|E9B578|E9B578_LEIMU Putative ribosomal protein L3 OS=Leishmania m	11
2.070776165	-0.5147	E9ANI6	tr|E9ANI6|E9ANI6_LEIMU Tubulin-tyrsoine ligase-like protein OS=Leishman	4
2.428978349	-0.515	E9AYS6	tr|E9AYS6|E9AYS6_LEIMU Kinetoplast-associated protein-like protein OS=L	2
1.937011002	-0.5166	E9B5Z7;E9	tr|E9B5Z7|E9B5Z7_LEIMU Putative NADH-dependent fumarate reductase C	18
2.310477749	-0.5173	E9AJD9;E9	tr|E9AJD9|E9AJD9_LEIMU Putative long-chain-fatty-acid-CoA ligase OS=Leis	4

2.411264447	-0.5174	E9AJJ4	tr|E9AJJ4|E9AJJ4_LEIMU Uncharacterized protein OS=Leishmania mexicana	7
2.277228256	-0.5176	E9B573	tr|E9B573|E9B573_LEIMU Regulatory subunit of protein kinase a-like prote	12
2.15944296	-0.5177	Q25327;E9	tr|Q25327|Q25327_LEIME Membrane-bound acid phosphatase OS=Leishm	3
1.588160005	-0.518	E9B5I1	tr|E9B5I1|E9B5I1_LEIMU Putative dynein heavy chain OS=Leishmania mexi	5
1.945805921	-0.5195	E9B1L9	tr|E9B1L9|E9B1L9_LEIMU Uncharacterized protein OS=Leishmania mexican	3
2.350152326	-0.5211	E9AXX5	tr|E9AXX5|E9AXX5_LEIMU Uncharacterized protein OS=Leishmania mexica	8
1.563188721	-0.5242	E9B2J9	tr|E9B2J9|E9B2J9_LEIMU Putative RNA binding protein OS=Leishmania mex	3
2.435432666	-0.5248	E9B638	tr|E9B638|E9B638_LEIMU Uncharacterized protein OS=Leishmania mexicar	2
1.821574683	-0.5248	E9B5S5	tr|E9B5S5|E9B5S5_LEIMU Putative ATP-dependent DEAD-box RNA helicase	8
2.686448563	-0.5278	E9AL95	tr|E9AL95|E9AL95_LEIMU Flavoprotein subunit-like protein OS=Leishmania	2
2.123334266	-0.5281	E9ANJ4	tr|E9ANJ4|E9ANJ4_LEIMU Uncharacterized protein OS=Leishmania mexicar	3
2.761960008	-0.5293	E9AL83	tr|E9AL83|E9AL83_LEIMU Topoisomerase-related function protein-like prot	2
2.213187142	-0.5305	E9AW86;E	tr|E9AW86|E9AW86_LEIMU Acetyl-coenzyme A synthetase OS=Leishmania	9
1.64336472	-0.5308	E9AZN4	tr|E9AZN4|E9AZN4_LEIMU Uncharacterized protein OS=Leishmania mexica	3
2.145343857	-0.5317	E9AQM8	tr|E9AQM8|E9AQM8_LEIMU Uncharacterized protein OS=Leishmania mexi	3
3.022342787	-0.5343	E9ALD8;E9	tr|E9ALD8|E9ALD8_LEIMU 40S ribosomal protein S19-like protein OS=Leish	2
1.846561866	-0.5344	E9B0C5	tr|E9B0C5|E9B0C5_LEIMU Uncharacterized protein LMXM_28_3030 OS=Le	2
1.93180057	-0.5345	E9AP69	tr|E9AP69|E9AP69_LEIMU Uncharacterized protein OS=Leishmania mexica	6
2.295594214	-0.5366	E9AUZ5	tr|E9AUZ5|E9AUZ5_LEIMU Uncharacterized protein OS=Leishmania mexica	2
2.010000105	-0.5366	E9B5Y8	tr|E9B5Y8|E9B5Y8_LEIMU Uncharacterized protein OS=Leishmania mexicar	3
1.73409018	-0.5375	E9ARW5	tr|E9ARW5|E9ARW5_LEIMU Uncharacterized protein OS=Leishmania mexic	3
1.55952177	-0.5381	E9ARX0	tr|E9ARX0|E9ARX0_LEIMU Uncharacterized protein OS=Leishmania mexica	2
2.633686486	-0.5392	E9AVQ7	tr|E9AVQ7|E9AVQ7_LEIMU Uncharacterized protein OS=Leishmania mexica	11
2.202272878	-0.5392	E9AM00	tr|E9AM00|E9AM00_LEIMU Putative U-box domain protein OS=Leishmania	2
2.107992159	-0.5398	Q8MPM3;I	tr|Q8MPM3|Q8MPM3_LEIME Transketolase OS=Leishmania mexicana mex	5
2.471528628	-0.5398	E9B3G8	tr|E9B3G8|E9B3G8_LEIMU Putative 60S ribosomal protein L2 OS=Leishman	3
2.480940049	-0.5415	E9B520	tr|E9B520|E9B520_LEIMU Putative asparaginyl-tRNA synthetase OS=Leishn	5
2.671845993	-0.5437	E9B001;Q5	tr|E9B001|E9B001_LEIMU Replication protein A subunit OS=Leishmania me	5
3.137654551	-0.544	E9AX57	tr|E9AX57|E9AX57_LEIMU Uncharacterized protein OS=Leishmania mexica	2
1.963091246	-0.5444	E9AQ78	tr|E9AQ78|E9AQ78_LEIMU Peroxisomal membrane protein PEX16 OS=Leisl	2
1.845409075	-0.5459	E9ARW7	tr|E9ARW7|E9ARW7_LEIMU Putative aminopeptidase OS=Leishmania mexi	3
2.051448301	-0.5463	E9AS66	tr|E9AS66|E9AS66_LEIMU Uncharacterized protein OS=Leishmania mexicar	3
2.35963711	-0.5468	E9B2G1	tr|E9B2G1|E9B2G1_LEIMU Putative ATP-dependent RNA helicase OS=Leish	20
2.256244261	-0.5479	E9AKX3	tr|E9AKX3|E9AKX3_LEIMU Putative glucosamine-fructose-6-phosphate ami	3
1.901028694	-0.5497	E9AZK8	tr|E9AZK8|E9AZK8_LEIMU Carbonic anhydrase-like protein OS=Leishmania	2
1.726491419	-0.5516	E9B6T5	tr|E9B6T5|E9B6T5_LEIMU Uncharacterized protein OS=Leishmania mexicar	2
2.558114035	-0.5517	E9B3C2	tr|E9B3C2|E9B3C2_LEIMU Uncharacterized protein OS=Leishmania mexica	2
2.20409899	-0.5521	E9B4J8	tr|E9B4J8|E9B4J8_LEIMU Uncharacterized protein OS=Leishmania mexican	5
2.321027105	-0.5559	E9AQL3;Q	tr|E9AQL3|E9AQL3_LEIMU Putative orotidine-5-phosphate decarboxylase/	16
1.744022443	-0.5564	E9AXG9	tr|E9AXG9|E9AXG9_LEIMU Peptidyl-prolyl cis-trans isomerase OS=Leishma	6
2.913780804	-0.5572	E9B5X7;E9	tr|E9B5X7|E9B5X7_LEIMU Putative aldose 1-epimerase OS=Leishmania me	2
1.98330424	-0.5574	E9AXU0	tr|E9AXU0|E9AXU0_LEIMU Elongation factor 2-like protein OS=Leishmania	2
2.443076837	-0.5598	E9ASP5;E9	tr|E9ASP5|E9ASP5_LEIMU Putative 40S ribosomal protein S9 OS=Leishmani	6
1.696596057	-0.5604	E9AJR6	tr|E9AJR6|E9AJR6_LEIMU Uncharacterized protein OS=Leishmania mexican	3
2.308591287	-0.5636	E9APE7;E9	tr|E9APE7|E9APE7_LEIMU 40S ribosomal protein S4 OS=Leishmania mexica	11
2.016828375	-0.5652	E9AKK2	tr|E9AKK2|E9AKK2_LEIMU Dipeptidyl peptidase 3 OS=Leishmania mexicana	3
2.240642273	-0.5687	E9B466	tr|E9B466|E9B466_LEIMU Uncharacterized protein OS=Leishmania mexicar	2
1.926148836	-0.5706	E9ASB9;E9	tr|E9ASB9|E9ASB9_LEIMU Histone H4 OS=Leishmania mexicana (strain MH	3
2.32866607	-0.5711	E9AZJ4	tr|E9AZJ4|E9AZJ4_LEIMU Putative rrp44p homologue OS=Leishmania mexic	2
2.374996131	-0.5716	E9AWU0	tr|E9AWU0|E9AWU0_LEIMU Putative replication factor C, subunit 1 OS=Lei	3
1.810645973	-0.5741	E9AUG6	tr|E9AUG6|E9AUG6_LEIMU Midasin OS=Leishmania mexicana (strain MHO	7
2.769189154	-0.5748	E9AY66;Q	tr|E9AY66|E9AY66_LEIMU Putative 40S ribosomal protein S16 OS=Leishma	4
2.269540182	-0.5764	E9AMR1	tr|E9AMR1|E9AMR1_LEIMU Putative cyclin 1 OS=Leishmania mexicana (str	3
1.79453863	-0.5787	E9B3L1	tr|E9B3L1|E9B3L1_LEIMU Putative ATP-binding cassette protein OS=Leishm	2
1.826839053	-0.5806	E9AWQ4	tr|E9AWQ4|E9AWQ4_LEIMU Kinesin-like protein OS=Leishmania mexicana	4
2.39283348	-0.581	E9B545	tr|E9B545|E9B545_LEIMU Uncharacterized protein OS=Leishmania mexicar	8
2.268127118	-0.583	E9AVH4	tr|E9AVH4|E9AVH4_LEIMU 40S ribosomal protein S6 OS=Leishmania mexic	3

2.674957419	-0.5841	E9B4N8	tr|E9B4N8|E9B4N8_LEIMU Uncharacterized protein OS=Leishmania mexica	4
2.316396299	-0.5847	E9B700	tr|E9B700|E9B700_LEIMU Uncharacterized protein OS=Leishmania mexicar	9
2.8817374	-0.5883	E9AZH2	tr|E9AZH2|E9AZH2_LEIMU Proteasome subunit beta OS=Leishmania mexic	2
2.512802479	-0.591	E9AR70	tr|E9AR70|E9AR70_LEIMU Uncharacterized protein OS=Leishmania mexicar	2
2.747267103	-0.5945	E9AQU5	tr|E9AQU5|E9AQU5_LEIMU Uncharacterized protein OS=Leishmania mexic	2
2.426579341	-0.5954	E9AV89	tr|E9AV89|E9AV89_LEIMU Putative 60S ribosomal protein L9 OS=Leishman	8
2.676830399	-0.5963	E9AMM1	tr|E9AMM1|E9AMM1_LEIMU Uncharacterized protein OS=Leishmania mex	2
2.312373413	-0.6029	E9B4X4	tr|E9B4X4|E9B4X4_LEIMU Uncharacterized protein OS=Leishmania mexicar	2
1.798888126	-0.603	E9AYA4	tr|E9AYA4|E9AYA4_LEIMU Uncharacterized protein OS=Leishmania mexica	2
2.656122977	-0.6046	E9AKP0	tr|E9AKP0|E9AKP0_LEIMU Peptidyl-prolyl cis-trans isomerase OS=Leishmar	2
2.271558915	-0.6154	E9AQE5;E9	tr|E9AQE5|E9AQE5_LEIMU Phosphodiesterase OS=Leishmania mexicana (st	10
1.923715346	-0.6159	E9AK98	tr|E9AK98|E9AK98_LEIMU Putative RNA-binding protein OS=Leishmania me	4
2.396942366	-0.6174	E9AK64	tr|E9AK64|E9AK64_LEIMU Pescadillo homolog OS=Leishmania mexicana (st	4
2.536082083	-0.619	E9AZL5	tr|E9AZL5|E9AZL5_LEIMU 40S ribosomal protein S26 OS=Leishmania mexic	2
2.15966167	-0.6214	E9ARS2	tr|E9ARS2|E9ARS2_LEIMU Putative pyruvate dehydrogenase E1 componen	4
2.655887816	-0.6216	E9AQK4	tr|E9AQK4|E9AQK4_LEIMU Putative 60S ribosomal protein L21 OS=Leishma	4
2.810070213	-0.6249	E9AYV1;E9	tr|E9AYV1|E9AYV1_LEIMU Putative calpain-like cysteine peptidase (Fragme	4
2.632820856	-0.6252	E9AK29	tr|E9AK29|E9AK29_LEIMU 60S ribosomal protein L11 (L5, L16) OS=Leishma	6
2.397180923	-0.6266	E9AZU1	tr|E9AZU1|E9AZU1_LEIMU Uncharacterized protein OS=Leishmania mexica	2
2.042329103	-0.6273	E9AN41	tr|E9AN41|E9AN41_LEIMU Protein transport protein SEC23 OS=Leishmania	2
2.683062915	-0.6291	E9B350;E9	tr|E9B350|E9B350_LEIMU Putative ribosomal protein L27 OS=Leishmania n	5
2.244838157	-0.6348	E9ANK8	tr|E9ANK8|E9ANK8_LEIMU Putative aminopeptidase (Metallo-peptidase, cl	2
2.694756695	-0.6363	E9AJQ8	tr|E9AJQ8|E9AJQ8_LEIMU Putative long chain fatty Acyl CoA synthetase OS	3
1.832485833	-0.6371	E9AMS7	tr|E9AMS7|E9AMS7_LEIMU Uncharacterized protein OS=Leishmania mexic	2
2.104452134	-0.6373	E9AZX7	tr|E9AZX7|E9AZX7_LEIMU Putative hydrolase, alpha/beta fold family OS=Le	2
3.065744437	-0.64	E9B722	tr|E9B722|E9B722_LEIMU Uncharacterized protein OS=Leishmania mexicar	2
2.088917307	-0.6431	E9AUU6	tr|E9AUU6|E9AUU6_LEIMU Putative 40S ribosomal protein S11 OS=Leishm	5
2.764636548	-0.646	E9B547	tr|E9B547|E9B547_LEIMU Uncharacterized protein OS=Leishmania mexicar	5
2.894539653	-0.6472	E9AVH2	tr|E9AVH2|E9AVH2_LEIMU Centromere/microtubule binding protein cbf5,p	4
2.255832537	-0.6496	E9B483	tr|E9B483|E9B483_LEIMU Uncharacterized protein OS=Leishmania mexicar	5
2.604107604	-0.6519	E9ANP0	tr|E9ANP0|E9ANP0_LEIMU 40S ribosomal protein S5 OS=Leishmania mexic	2
2.228104749	-0.6544	E9B1X5	tr|E9B1X5|E9B1X5_LEIMU Nucleosome assembly protein-like protein OS=Le	3
2.576865079	-0.6558	E9ARL1	tr|E9ARL1|E9ARL1_LEIMU Putative elongation factor Tu OS=Leishmania me	12
2.770320883	-0.6604	E9B183	tr|E9B183|E9B183_LEIMU Uncharacterized protein OS=Leishmania mexicar	2
2.639049249	-0.6637	E9AXZ8;E9	tr|E9AXZ8|E9AXZ8_LEIMU Putative 60S ribosomal protein L7 OS=Leishmani	6
2.409655197	-0.6713	E9AQ75	tr|E9AQ75|E9AQ75_LEIMU Uncharacterized protein OS=Leishmania mexica	2
2.143752764	-0.6713	E9AUQ0	tr|E9AUQ0|E9AUQ0_LEIMU Uncharacterized protein OS=Leishmania mexic	3
3.589261353	-0.6745	E9AKN1;Q(	tr|E9AKN1|E9AKN1_LEIMU Uncharacterized protein OS=Leishmania mexica	4
2.839542445	-0.6757	E9AU20	tr|E9AU20|E9AU20_LEIMU Uncharacterized protein OS=Leishmania mexica	5
2.412920123	-0.6779	E9AW79	tr|E9AW79|E9AW79_LEIMU Uncharacterized protein OS=Leishmania mexic	2
2.968603918	-0.6785	E9B6K7	tr|E9B6K7|E9B6K7_LEIMU Putative 60S ribosomal subunit protein L31 OS=L	2
2.237888504	-0.6787	E9AXM2	tr|E9AXM2|E9AXM2_LEIMU Uncharacterized protein OS=Leishmania mexic	2
3.226201619	-0.6788	E9ATF7	tr|E9ATF7|E9ATF7_LEIMU Putative 40S ribosomal protein S27-1 OS=Leishm	3
3.018653281	-0.684	E9AKC3	tr|E9AKC3|E9AKC3_LEIMU Uncharacterized protein OS=Leishmania mexica	2
2.739512058	-0.6849	E9B3B4	tr|E9B3B4|E9B3B4_LEIMU Dihydrolipoyl dehydrogenase OS=Leishmania me	4
3.226097339	-0.6876	E9AMC1	tr|E9AMC1|E9AMC1_LEIMU Uncharacterized protein OS=Leishmania mexic	2
4.149994056	-0.6898	E9ATA6	tr|E9ATA6|E9ATA6_LEIMU Putative 60S ribosomal protein L22 OS=Leishma	2
2.31575226	-0.6902	E9ANH0	tr|E9ANH0|E9ANH0_LEIMU Proteasome subunit alpha type OS=Leishmania	2
3.010727622	-0.6953	E9ASL3	tr|E9ASL3|E9ASL3_LEIMU Putative 40S ribosomal protein S18 OS=Leishman	5
2.981559008	-0.6988	E9APZ2	tr|E9APZ2|E9APZ2_LEIMU Uncharacterized protein OS=Leishmania mexicar	12
3.077808522	-0.7004	E9ARG5	tr|E9ARG5|E9ARG5_LEIMU Uncharacterized protein OS=Leishmania mexica	2
2.69540737	-0.708	E8NHI0	tr|E8NHI0|E8NHI0_LEIMU 40S ribosomal protein S3a OS=Leishmania mexic	7
1.968427351	-0.7094	Q9GU60;E9	tr|Q9GU60|Q9GU60_LEIAM Kinetoplast membrane protein 11 OS=Leishma	2
1.687094262	-0.7122	E9B303	tr|E9B303|E9B303_LEIMU Uncharacterized protein OS=Leishmania mexicar	2
3.208044268	-0.718	E9B6Q4	tr|E9B6Q4|E9B6Q4_LEIMU Putative 60S ribosomal protein L27A/L29 OS=Le	3
2.963277625	-0.7199	E9B078	tr|E9B078|E9B078_LEIMU Putative 40S ribosomal protein S17 OS=Leishmar	4
2.763260098	-0.7228	E9AQ29	tr|E9AQ29|E9AQ29_LEIMU Ribonucleoprotein p18, mitochondrial,putative	5

2.794542651	-0.7242	E9ART6	tr|E9ART6|E9ART6_LEIMU Plasma membrane ATPase OS=Leishmania mexi	6
0.5922187	-0.733	E9B3Y5	tr|E9B3Y5|E9B3Y5_LEIMU Uncharacterized protein OS=Leishmania mexicar	2
2.408716407	-0.7389	E9APV6	tr|E9APV6|E9APV6_LEIMU Putative kinesin K39 OS=Leishmania mexicana (s	3
2.771166667	-0.739	E9AXF0	sp|E9AXF0|IF5A_LEIMU Eukaryotic translation initiation factor 5A OS=Leish	3
3.242759286	-0.7463	E9B665	tr|E9B665|E9B665_LEIMU Putative 60S ribosomal protein L5 OS=Leishmani	5
2.049888327	-0.7515	E9ARW8	tr|E9ARW8|E9ARW8_LEIMU Uncharacterized protein OS=Leishmania mexi	3
2.579853419	-0.7532	E9AK86	tr|E9AK86|E9AK86_LEIMU Acyltransferase-like protein, copy 2 OS=Leishma	3
3.081333764	-0.759	E9AW42	tr|E9AW42|E9AW42_LEIMU ABC transporter-like protein OS=Leishmania m	2
3.513532775	-0.7731	V5RIA5;E9	tr|V5RIA5|V5RIA5_LEIAM Calmodulin-like protein OS=Leishmania amazone	3
2.2647001	-0.7804	E9B012	tr|E9B012|E9B012_LEIMU Putative zinc transporter OS=Leishmania mexica	2
2.849177434	-0.7848	E9ALH7	tr|E9ALH7|E9ALH7_LEIMU Putative 60S ribosomal protein L13 OS=Leishma	6
2.710283511	-0.7965	E9APB2	tr|E9APB2|E9APB2_LEIMU Uncharacterized protein OS=Leishmania mexica	4
1.932135395	-0.7993	E9ANG1	tr|E9ANG1|E9ANG1_LEIMU Uncharacterized protein OS=Leishmania mexic	2
2.786816701	-0.8065	E8NHE3	tr|E8NHE3|E8NHE3_LEIMU Putative 60S ribosomal protein L28 OS=Leishma	3
3.105880367	-0.8066	E9ANF3	tr|E9ANF3|E9ANF3_LEIMU Uncharacterized protein OS=Leishmania mexica	6
2.515170467	-0.8117	E9AXX0	tr|E9AXX0|E9AXX0_LEIMU Uncharacterized protein OS=Leishmania mexica	2
2.579168861	-0.8181	E9AQ99;E9	tr|E9AQ99|E9AQ99_LEIMU 60S ribosomal protein L6 OS=Leishmania mexic	3
2.496890037	-0.8264	E9B1D2	tr|E9B1D2|E9B1D2_LEIMU Putative serine/threonine-protein kinase OS=Le	4
2.381457864	-0.8271	E9B6Q7	tr|E9B6Q7|E9B6Q7_LEIMU Putative 60S ribosomal protein L23 OS=Leishma	4
2.569812272	-0.828	E9AY41	tr|E9AY41|E9AY41_LEIMU Putative 10 kDa heat shock protein OS=Leishmar	3
3.107465515	-0.8382	E9AXD1	tr|E9AXD1|E9AXD1_LEIMU Uncharacterized protein OS=Leishmania mexica	4
3.243822995	-0.8445	E9AVK9	tr|E9AVK9|E9AVK9_LEIMU Uncharacterized protein OS=Leishmania mexica	2
3.03016063	-0.8636	E9AVK4	tr|E9AVK4|E9AVK4_LEIMU Uncharacterized protein OS=Leishmania mexica	6
3.485903733	-0.8675	Q9NGK2;E	tr|Q9NGK2|Q9NGK2_LEIAM 40S ribosomal protein S24 OS=Leishmania ama	5
3.337043348	-0.8948	E9ALB6	tr|E9ALB6|E9ALB6_LEIMU Putative nucleolar RNA-binding protein OS=Leish	3
2.343149798	-0.8965	E9AWJ4	tr|E9AWJ4|E9AWJ4_LEIMU Putative 60S ribosomal protein L17 OS=Leishma	3
2.828047642	-0.9207	E9B047	tr|E9B047|E9B047_LEIMU Putative glycosomal membrane protein OS=Leish	2
3.187440576	-0.9354	E9AQB1	tr|E9AQB1|E9AQB1_LEIMU 60S acidic ribosomal protein P2 OS=Leishmania	2
2.886106385	-0.9542	E9B1H5	tr|E9B1H5|E9B1H5_LEIMU Putative ATP-dependent RNA helicase OS=Leish	2
2.852072816	-0.9899	E9AQ19	tr|E9AQ19|E9AQ19_LEIMU Putative 60S ribosomal protein L13a OS=Leishm	5
3.330694102	-1.0339	E9B376;A0	tr|E9B376|E9B376_LEIMU Nucleoside diphosphate kinase OS=Leishmania n	2
3.189458665	-1.0367	E9B2E2	tr|E9B2E2|E9B2E2_LEIMU Uncharacterized protein OS=Leishmania mexicar	7
2.960857464	-1.053	E9AK81	tr|E9AK81|E9AK81_LEIMU Uncharacterized protein OS=Leishmania mexica	5
2.924042385	-1.0652	E9AXC7;E9	tr|E9AXC7|E9AXC7_LEIMU RNA-binding protein, putative, UPB2 OS=Leishm	3
3.700379913	-1.0759	E9AYA9	tr|E9AYA9|E9AYA9_LEIMU Uncharacterized protein OS=Leishmania mexica	3
2.730545714	-1.0897	E9AKU9	tr|E9AKU9|E9AKU9_LEIMU Uncharacterized protein OS=Leishmania mexica	2
2.613171777	-1.0988	E9AM28	tr|E9AM28|E9AM28_LEIMU Cofilin-like protein OS=Leishmania mexicana (s	2
2.560763147	-1.1032	E9B1E7;E9	tr|E9B1E7|E9B1E7_LEIMU Putative 60S acidic ribosomal protein P2 OS=Leis	2
3.979739454	-1.1132	E9ANM6	tr|E9ANM6|E9ANM6_LEIMU Uncharacterized protein OS=Leishmania mexi	3
2.842654209	-1.1285	E9B5U1;E9	tr|E9B5U1|E9B5U1_LEIMU 60S ribosomal protein L18a OS=Leishmania mex	3
3.303875053	-1.2193	E9ALB7	tr|E9ALB7|E9ALB7_LEIMU RNA binding protein-like protein OS=Leishmania	3
3.243615272	-1.2429	E9B2E8	tr|E9B2E8|E9B2E8_LEIMU Uncharacterized protein OS=Leishmania mexicar	6
3.299144753	-1.4505	E9ALA4	tr|E9ALA4|E9ALA4_LEIMU Splicing factor ptsr1-like protein OS=Leishmania	7
1.562636673	-1.5019	E9AKF9	tr|E9AKF9|E9AKF9_LEIMU Kinetoplast-associated protein-like protein OS=L	2

Table S9. GO Terms enriched in the tanespimycin-affected *L. mexicana* RBPs

Top10 MF GO-Terms; Tanespimycin-affected RBPs of *L. mexicana* promastigotes

ID	Name	Result count	P-value	Benjamini
GO:0003735	structural constituent of ribosome	98	1.14E-37	3.46E-35
GO:1901363	heterocyclic compound binding	313	1.88E-14	2.28E-12
GO:0097159	organic cyclic compound binding	313	1.88E-14	2.28E-12
GO:0003723	RNA binding	119	3.60E-13	3.11E-11
GO:0016875	ligase activity, forming carbon-oxygen bonds	18	3.27E-10	2.20E-08
GO:0004812	aminoacyl-tRNA ligase activity	18	3.27E-10	2.20E-08
GO:0003729	mRNA binding	45	2.12E-09	1.29E-07
GO:0051082	unfolded protein binding	20	2.46E-09	1.36E-07
GO:0003676	nucleic acid binding	155	3.97E-09	2.00E-07
GO:0016874	ligase activity	39	5.82E-07	2.71E-05

Top10 CC GO-Terms; Tanespimycin-affected RBPs of *L. mexicana* promastigotes

ID	Name	Result count	P-value	Benjamini
GO:0005840	ribosome	125	4.00E-23	6.56E-21
GO:0043232	intracellular non-membrane-bounded organelle	270	7.51E-21	4.93E-19
GO:0032991	protein-containing complex	192	1.46E-16	6.85E-15
GO:0005730	nucleolus	90	3.00E-14	1.23E-12
GO:0031981	nuclear lumen	201	3.83E-10	1.25E-08
GO:0005737	cytoplasm	527	9.56E-10	2.85E-08
GO:0005634	nucleus	283	5.18E-09	1.42E-07
GO:0000502	proteasome complex	20	9.67E-09	2.44E-07
GO:0044428	obsolete nuclear part	134	9.29E-08	2.18E-06
GO:1905369	endopeptidase complex	20	1.02E-07	2.24E-06

Top10 BP GO-Terms; Tanespimycin-affected RBPs of *L. mexicana* promastigotes

ID	Name	Result count	P-value	Benjamini
GO:0006412	translation	130	2.94E-47	1.44E-44
GO:0043043	peptide biosynthetic process	130	9.76E-47	3.19E-44
GO:0043603	cellular amide metabolic process	136	1.39E-46	3.42E-44
GO:0006518	peptide metabolic process	132	6.45E-46	1.26E-43
GO:1901566	organonitrogen compound biosynthetic process	167	1.70E-41	2.78E-39
GO:0009058	biosynthetic process	200	1.45E-31	1.42E-29
GO:0010467	gene expression	173	2.90E-29	2.03E-27
GO:0034645	cellular macromolecule biosynthetic process	141	6.73E-29	4.40E-27
GO:1901564	organonitrogen compound metabolic process	260	3.03E-28	1.86E-26
GO:0008152	metabolic process	399	7.00E-22	3.81E-20

Top10 MF GO-Terms; Tanespimycin-affected RBPs of *L. mexicana* amastigotes

ID	Name	Result count	P-value	Benjamini
GO:0003735	structural constituent of ribosome	82	4.50E-42	9.45E-40
GO:1901363	heterocyclic compound binding	235	1.39E-26	1.17E-24
GO:0097159	organic cyclic compound binding	235	1.39E-26	1.17E-24
GO:0003723	RNA binding	104	2.43E-24	1.70E-22
GO:0003676	nucleic acid binding	132	6.53E-21	3.92E-19
GO:0003729	mRNA binding	44	4.65E-17	2.17E-15
GO:0036094	small molecule binding	137	1.14E-11	4.81E-10
GO:0008135	translation factor activity, RNA binding	26	1.61E-11	5.20E-10
GO:0090079	translation regulator activity, nucleic acid binding	26	1.61E-11	5.20E-10
GO:0043168	anion binding	130	4.17E-11	1.25E-09

Top10 CC GO-Terms; Tanespimycin-affected RBPs of *L. mexicana* amastigotes

ID	Name	Result count	P-value	Benjamini
GO:0005840	ribosome	92	1.43E-23	1.24E-21

GO:0005622	intracellular anatomical structure	431	1.48E-20	9.61E-19
GO:0005730	nucleolus	75	2.30E-20	1.20E-18
GO:0031981	nuclear lumen	136	2.08E-11	6.77E-10
GO:0022626	cytosolic ribosome	11	1.45E-09	3.78E-08
GO:0005829	cytosol	28	1.99E-09	4.70E-08
GO:0005634	nucleus	179	1.45E-08	3.15E-07
GO:0031974	membrane-enclosed lumen	141	2.36E-08	4.10E-07
GO:0043233	organelle lumen	141	2.36E-08	4.10E-07
GO:0070013	intracellular organelle lumen	141	2.36E-08	4.10E-07

Top10 BP GO-Terms; Tanespimycin-affected RBPs of *L. mexicana* amastigotes

ID	Name	Result count	P-value	Benjamini
GO:0006412	translation	105	4.08E-50	1.11E-47
GO:0043043	peptide biosynthetic process	105	1.05E-49	2.14E-47
GO:0006518	peptide metabolic process	106	1.13E-48	1.84E-46
GO:0044271	cellular nitrogen compound biosynthetic process	121	8.28E-40	1.12E-37
GO:0034645	cellular macromolecule biosynthetic process	113	2.84E-35	2.89E-33
GO:0009058	biosynthetic process	149	4.11E-35	3.34E-33
GO:0010467	gene expression	132	9.59E-34	7.10E-32
GO:0034641	cellular nitrogen compound metabolic process	165	1.34E-29	7.81E-28
GO:0044267	cellular protein metabolic process	126	1.04E-18	4.48E-17
GO:0044237	cellular metabolic process	217	6.49E-18	2.52E-16

Table S10. Effect of tanespimycin on the RBPs of*L. mexicana* positively (highlighted in blue) and negatively (highlighted in orange) correlated with the synthesis of the proteins.

Log2 FC (Tan-noTan)	Protein IDs	Protein name
-0.900472641	E8NHI0	40S ribosomal protein S3a
-0.572738647	E9AJ91	Putative carboxylase
-0.867225011	E9AK58	Putative 60S ribosomal protein L10
-0.872570038	E9AK97	Putative fructose-1,6-bisphosphatase, cytosolic
-0.752810796	E9AKE4	Putative microtubule-associated protein (Fragment)
-0.666592916	E9AL62;E9AL63	Putative 60S ribosomal protein L7a
-1.181669235	E9ALD8;E9B569	40S ribosomal protein S19-like protein
-0.549959819	E9ALG7	Putative serine/threonine-protein kinase
-0.837327321	E9ALH7	Putative 60S ribosomal protein L13
-0.65726916	E9ALL3	Uncharacterized protein
-1.193260829	E9AM67	Proteasome regulatory non-ATPase subunit,putative
-0.435370763	E9AP62;E8NHR0	Tubulin alpha chain
1.602411906	E9AP69	Uncharacterized protein
0.581494013	E9APB2	Uncharacterized protein
-0.74956131	E9APE7;E9APE6	40S ribosomal protein S4
-1.189582825	E9API2	Ubiquitin-conjugating enzyme-like protein
-0.767786662	E9APJ0	Putative chaperonin TCP20
-1.388770421	E9APT0	Putative calpain-like cysteine peptidase
-0.691284815	E9AQ19	Putative 60S ribosomal protein L13a
0.741272608	E9AQ29	Ribonucleoprotein p18, mitochondrial,putative
-1.477350871	E9AQ94	Putative 40S ribosomal protein S3
-0.873127619	E9AQ99;E9B3Q4	60S ribosomal protein L6
-1.517837524	E9AQB1	60S acidic ribosomal protein P2
0.956509908	E9AQK4	Putative 60S ribosomal protein L21
-0.972090403	E9ARA1	Putative translation initiation factor
-1.24161466	E9ARZ1	Putative 40S ribosomal protein S13
-0.429400126	E9AS67	Uncharacterized protein
-0.500628153	E9ASB9	Histone H4
-1.339741389	E9ASD6	Elongation factor 2
-1.008309682	E9ASL3	Putative 40S ribosomal protein S18
-0.536635717	E9ASM7	Putative ribosomal protein L24
-0.799114863	E9ASP5;E9AL81	Putative 40S ribosomal protein S9
-0.954479853	E9AST4	Clathrin heavy chain
-0.957728068	E9AT13	Putative tyrosine aminotransferase
-1.111968358	E9AT43	Dihydrolipoamide acetyltransferase component of pyruvate dehydrogenase complex
-0.815184911	E9AT73	Succinate--CoA ligase [ADP-forming] subunit beta, mitochondrial
-0.428725561	E9AT99	14-3-3 protein-like protein
-1.460632324	E9ATD8	Putative cysteine synthase
-0.757368088	E9ATK7	Uncharacterized protein
-0.934185028	E9ATN0	Uncharacterized protein
-0.921944936	E9ATN5;E9AP80	Putative 60S ribosomal protein L18
0.74280866	E9ATU4	Uncharacterized protein
-0.441877365	E9ATZ6	Putative isoleucyl-tRNA synthetase
-0.902696609	E9AV93	T-complex protein 1 subunit delta
-0.822966894	E9AVE1	Putative RNA helicase
-0.850364685	E9AVE2	Uncharacterized protein
-0.986647924	E9AVG6	Proteasome subunit alpha type
-0.793238958	E9AVH4	40S ribosomal protein S6
0.622889837	E9AVQ7	Uncharacterized protein
-0.761469523	E9AVR1	Putative NADH-cytochrome b5 reductase
-0.847902298	E9AVX8	I/6 autoantigen-like protein
-1.10760053	E9AVY6	Alanine--tRNA ligase
-0.61144956	E9AW08	Uncharacterized protein
-0.865780512	E9AW84	3-ketoacyl-CoA thiolase-like protein
-1.117176692	E9AWE0	T-complex protein 1 subunit gamma
-1.088214874	E9AWI3	V-type proton ATPase subunit a
-1.049395879	E9AWR7	Malic enzyme
-0.954166412	E9AX01	Uncharacterized protein
-0.425351461	E9AXD1	Uncharacterized protein
-0.489401499	E9AXF3	Uncharacterized protein
0.462238948	E9AXG9	Peptidyl-prolyl cis-trans isomerase
-1.46401151	E9AXT3	Uncharacterized protein
-1.487605413	E9AXZ5	Adenine phosphoribosyltransferase

-0.64060084	E9AY60;E9AY59	Glutathione peroxidase
-1.05365181	E9AYS6	Kinetoplast-associated protein-like protein
-1.070138295	E9AZE0	Obg-like ATPase 1
-0.960509618	E9B073	Putative acyl-CoA dehydrogenase
-0.779999415	E9B078	Putative 40S ribosomal protein S17
-1.041792552	E9B1E9	Putative 5-methyltetrahydropteroyltriglutamate--homocysteine methyltransferase
-0.675694784	E9B1S0	Putative vacuolar-type proton translocating pyrophosphatase 1
-0.855739594	E9B1W4;E9B1W3	Thiolase protein-like protein
-1.110507965	E9B297	Putative acetyl-CoA carboxylase
-0.941595713	E9B2L9	Uncharacterized protein
-1.301782608	E9B2Q1	Uncharacterized protein
-1.048489253	E9B350;E9B348	Putative ribosomal protein L27
-0.773775736	E9B384	Uncharacterized protein
-0.93494161	E9B3B4	Dihydrolipoyl dehydrogenase
-0.904457728	E9B3G5	Putative myosin heavy chain
0.418612162	E9B3L2;P27741	Heat shock protein 83-1
0.415847143	E9B3P3	Uncharacterized protein
-1.288141886	E9B3W9	Cysteine conjugate beta-lyase,aminotransferase-like protein
1.074305216	E9B3Z3	Ribulose-phosphate 3-epimerase
-0.599527359	E9B478	Putative heat shock protein
-0.723982493	E9B493	Putative carboxypeptidase
-0.963277817	E9B544	Uncharacterized protein
-1.119827906	E9B578;E9B396	Putative ribosomal protein L3
-0.740579605	E9B5F7	Transmembrane 9 superfamily member
-0.815516154	E9B5P1;Q27686	Pyruvate kinase
-0.859295527	E9B5S5	Putative ATP-dependent DEAD-box RNA helicase
-1.007140477	E9B5Z7	Putative NADH-dependent fumarate reductase
-0.737489065	E9B665	Putative 60S ribosomal protein L5
-1.585186005	E9B669;E9AV40	Putative 60S Ribosomal protein L36
-0.93783315	E9B694	Uncharacterized protein
-0.544847488	E9B6K2	Putative cystathione gamma lyase
-1.810284932	E9B6Q4	Putative 60S ribosomal protein L27A/L29
-1.145910263	E9B6Q7	Putative 60S ribosomal protein L23
-1.161759059	E9B6U3	Polyadenylate-binding protein
-1.235321681	E9B6X2	Putative mitochondrial phosphate transporter
-0.488818487	Q8MPM3;E9AX52	Transketolase
-1.245927175	Q9BLW4;E9AW11	GDP-mannose pyrophosphorylase
-0.756830215	Q9NGK2;E9AT64	40S ribosomal protein S24
-0.431416194	E8NHE3	Putative 60S ribosomal protein L28
-1.293479284	E8NHJ5;E9AWZ6	IgE-dependent histamine-releasing factor,putative
1.556575139	E9AJC9	40S ribosomal protein S7
-1.424559911	E9AJK8	Uncharacterized protein
-0.63649559	E9AKA4;C6KJE2;C6KJD6	Actin
-1.332153956	E9AKK2	Dipeptidyl peptidase 3
-0.814347585	E9AKP0	Peptidyl-prolyl cis-trans isomerase
1.156283696	E9ALA4	Splicing factor ptsr1-like protein
-0.958375295	E9ALK7	Rab GDP dissociation inhibitor
-0.804760615	E9ALM8	Putative fumarate hydratase (Fragment)
-0.969751994	E9ALU2	Putative carnitine/choline acetyltransferase
-1.61127917	E9ALW5;E9ALW4	Putative ribosomal protein L1a
-0.456759771	E9ALY6;Q9U4E3	Putative ADP ribosylation factor 3
1.952315648	E9AM47	Uncharacterized protein
2.419125239	E9AMA8	Superoxide dismutase
-1.158338547	E9ANA1	Putative small GTP-binding protein Rab11
-0.825245539	E9ANH0	Proteasome subunit alpha type
-1.064140956	E9ANI1	Putative 14-3-3 protein
-1.150180817	E9ANP7	Uncharacterized protein
-0.767587026	E9ANV2	Putative cysteinyl-tRNA synthetase
-0.720156988	E9ANZ0	Putative alanine aminotransferase
0.57394282	E9APL2	Uncharacterized protein
-1.104552587	E9APW3;Q3HL75	Enolase
0.999807358	E9APZ2	Uncharacterized protein
-0.53196907	E9AQA0	NAD-specific glutamate dehydrogenase
-0.528673808	E9AQN5	Transaldolase
-1.368091583	E9ARC2	Uncharacterized protein
-0.796550115	E9ARI8	Aconitate hydratase
-0.704473495	E9ARN6	UTP--glucose-1-phosphate uridylyltransferase

-0.679398855	E9ASC5	Stress-inducible protein STI1 homolog
-1.270690918	E9ASQ6	Transitional endoplasmic reticulum ATPase,putative
-1.034269969	E9ASS2	Uncharacterized protein
1.659996033	E9AT16;E9AT15;C9E262	Methyltransferase
0.976696014	E9ATF6;E9ARS4	Putative 60S ribosomal protein L34
-0.525150935	E9ATH3	Adenosylhomocysteinase
1.634650548	E9AU20	Uncharacterized protein
-1.054442088	E9AU26	Uncharacterized protein
-0.619620641	E9AUA1	2,3-bisphosphoglycerate-independent phosphoglycerate mutase
1.099084218	E9AUU6	Putative 40S ribosomal protein S11
-0.820654551	E9AUZ2;E9AUZ1;Q4VT6	Phosphotransferase
-1.627217611	E9AV22	Dihydrolipoamide acetyltransferaselike protein
-0.775581996	E9AV44	Proteasome regulatory non-ATP-ase subunit 5,putative
-0.63511912	E9AVH3	Putative ATP synthase F1 subunit gamma protein
-0.559890111	E9AW21	Putative endoribonuclease L-PSP (Pb5)
-1.37147967	E9AW86;E9AW57	Acetyl-coenzyme A synthetase
0.434722265	E9AWR8	Putative malic enzyme
0.593442917	E9AX53	40S ribosomal protein S8
-0.604983648	E9AX57	Uncharacterized protein
-0.575230281	E9AXJ1	Aldehyde dehydrogenase, mitochondria
-0.959449132	E9AXM1	GTP-binding nuclear protein
-1.035684586	E9AXQ3	Pyruvate dehydrogenase E1 component subunit beta
-0.688212713	E9AYD6	Putative thimet oligopeptidase
-0.474522273	E9AZ67	Uncharacterized protein
-0.739544551	E9AZ89;A5JV94	Putative trypanothione synthetase
-0.924343109	E9AZR7	Putative ribosomal protein S20
-0.588586807	E9B047	Putative glycosomal membrane protein
-0.510678609	E9B1C6	S-adenosylmethionine synthase
-1.135185877	E9B215	Prostaglandin f2-alpha synthase
-1.45102946	E9B2J9	Putative RNA binding protein
-0.479981105	E9B2V9;E9B2W0	Superoxide dismutase
-0.738406499	E9B307	Uncharacterized protein
-0.48428599	E9B376	Nucleoside diphosphate kinase
-1.251626968	E9B3B0	Putative chaperonin alpha subunit
-0.968808492	E9B473	Succinyl-CoA:3-ketoacid-coenzyme A transferase
-1.622092565	E9B4X7	Uncharacterized protein
-1.324446996	E9B6B5	Putative aminopeptidase
2.924128215	E9B6K7	Putative 60S ribosomal subunit protein L31
-0.982737223	E9B6N5	Uncharacterized protein
-1.14064916	E9B6R4	T-complex protein 1 subunit eta
-1.107983271	E9B713	Proteasome subunit alpha type
-0.966897329	E9AR60	Uncharacterized protein
-0.591276169	E9APY4	Myo-inositol-1-phosphate synthase
